# Transition Metal Mimetic π-Activation by Cationic Bismuth(III) Catalysts for Allylic C–H Functionalization of Olefins Using C=O and C=N Electrophiles

**DOI:** 10.1021/jacs.4c06235

**Published:** 2024-08-05

**Authors:** Ruihan Wang, Sebastián Martínez, Johannes Schwarzmann, Christopher Z. Zhao, Jacqueline Ramler, Crispin Lichtenberg, Yi-Ming Wang

**Affiliations:** † Department of Chemistry, University of Pittsburgh, Pittsburgh, Pennsylvania 15260, United States; § Department of Chemistry, Philipps-University Marburg, Hans-Meerwein-Str. 4, 35032 Marburg, Germany

## Abstract

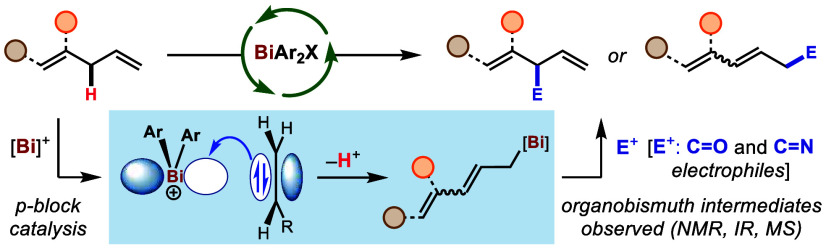

The discovery and utilization of main-group element catalysts that behave similarly to transition metal (TM) complexes have become increasingly active areas of investigation in recent years. Here, we report a series of Lewis acidic bismuth(III) complexes that allow for the catalytic allylic C(sp^3^)–H functionalization of olefins via an organometallic complexation-assisted deprotonation mechanism to generate products containing new C–C bonds. This heretofore unexplored mode of main-group reactivity was applied to the regioselective functionalization of 1,4-dienes and allylbenzene substrates. Experimental and computational mechanistic studies support the key steps of the proposed catalytic cycle, including the intermediacy of elusive Bi–olefin complexes and allylbismuth species.

The development of new main-group based catalysts that emulate the behavior of transition metal (TM) complexes has garnered significant attention in recent years, driven by both interest in fundamental reactivity and the demand for more sustainable alternatives to noble metal catalysts.^1^ Remarkable progress in this context has been made in the catalytic chemistry of alkali and alkaline earth metals,^2^ frustrated Lewis pairs,^3^ organopnictogens,^1c,1d,4^ organoselenides,^5^ and hypervalent iodine compounds.^6^ Despite these achievements, a number of desirable reactivity modes characteristic of TM catalysis remain underexplored or unavailable for main-group complexes. Notably, although the TM-catalyzed functionalization of C–H bonds has emerged as an effective tool for streamlining chemical synthesis and accessing novel molecular structures,^7^ main-group based catalytic systems that can emulate the requisite elementary steps are rare. In a seminal report, Fontaine and co-workers disclosed an ambiphilic aminoborane catalyst that could mimic a concerted metalation deprotonation step commonly proposed for TM-catalyzed C(sp^2^)–H functionalization.^8^ However, a TM-mimetic C(sp^3^)–H functionalization reaction had remained elusive. The handful of reported main-group catalyzed processes all proceed through prototypical main-group reactivity patterns.^2b,5b,9^

Simple unsaturated hydrocarbons (i.e., alkenes and alkynes) are attractive targets for the development of C(sp^3^)–H functionalization, due to their synthetic importance and wide availability.^10^ For two-electron processes, the coordination of a transition metal to the π-bond is typically a critical recognition and activation event. With respect to main-group elements, which lack energetically accessible, partially filled d orbitals, their interactions with C–C multiple bonds are relatively weak and remain poorly understood.^11^ Nevertheless, these interactions have been invoked in an array of main-group catalyzed hydrofunctionalization reactions.^2b,12^ We wondered whether the π-activating property of main-group elements could be extended to the role of assisting in the cleavage of neighboring C(sp^3^)–H bonds for TM-like catalytic C–H functionalization.

One of our groups (Y.-M.W.) and others have recently developed an approach for TM-catalyzed C–H functionalization which exploits the coordination between cationic metal centers and π-bonds to facilitate the heterolytic cleavage of α-C(sp^3^)–H bonds.^13^ Here, we report the development of a mechanistically analogous bismuth(III)-based catalytic system for allylic C–H functionalization of alkenes, enabling TM-like reactivity for C–C bond formation reactions with carbonyl and iminium electrophiles. Notably, this system was amenable to a range of 1,4-dienes, which are common substructures in bioactive molecules and important intermediates for organic synthesis.^14^ While previously reported metal-catalyzed allylic functionalization protocols generally provide double-bond isomerized conjugated diene products (α-selectivity),^15^ the Bi-catalyzed process reported here affords access to products that retain the 1,4-diene substructure (γ-selectivity, Scheme 1B) for the reaction of 1,4-dienes with two general classes of electrophilic coupling partners.

**Scheme 1:**
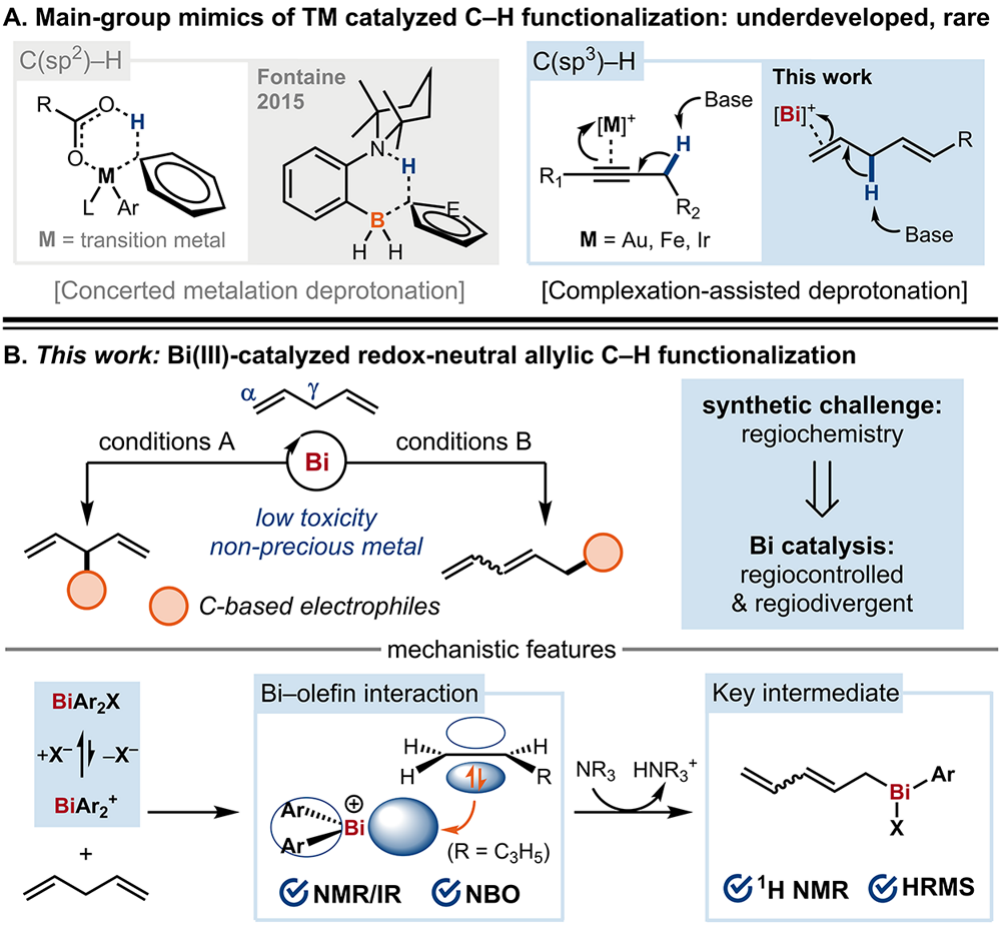
General Context and Features of Bi-Catalyzed Allylic C–H Functionalization

At the outset, we selected 1,4-pentadiene, a simple hydrocarbon derived from renewable feedstocks,^16^ as the target of allylic C–H functionalization, using 4-bromobenzaldehyde (**1a**) as the model electrophilic coupling partner.^13c,13d,13g^ A range of commercially available main-group Lewis acids were tested for their catalytic reactivity in allylic C–H functionalization. Pleasingly, heavy *p*-block metals In and Bi were found to be reactive, giving a mixture of linear (α) and branched (γ) carbonyl allylation products (see Supporting Information). We thus focused our attention on Bi, a stable, nontoxic, inexpensive and Earth-abundant main-group element.^17^ Recent reports suggest a strong tendency for cationic Bi(III) species to interact with soft Lewis bases, as evidenced by the retention of their thiophilicity in the presence of hard Lewis bases (e.g., THF and pyridine).^18^ The soft character of Bi is also suggested by its high tolerance of (hard) oxygen/nitrogen-based functional groups observed by others in the recent renaissance of Bi chemistry in organic synthesis.^19^ These traits boded well for the application of Bi as a late TM surrogate for olefin π-activation, prompting us to further explore Bi(III) complexes for allylic C–H functionalization.

To optimize this Bi(III)-catalyzed coupling process, we examined the performance of a range of readily accessible cationic Bi(III) compounds with diverse ligand structures and identified **Bi1** as an effective catalyst that gave the branched isomer **1** in 49% yield with high regioselectivity (Table 1, entry 3). By comparison, our group’s previously developed cyclopentadienyliron catalysts,^13c^ though providing good overall yield, were unable to control the regioselectivity (Table 1, top right). The addition of 10 mol % of LiNTf_2_ further improved the yield (Table 1, top left).^13d^ Interestingly, switching the Lewis acid from BF_3_·Et_2_O to TMSOTf led to opposite regioselective outcomes (entry 4). Several Bi(III) complexes bearing more elaborate ligand scaffolds exhibited lower reactivity or selectivity compared to **Bi1** (entries 7–9). Control experiments indicated that the main reaction pathway is unlikely to be an ene-type reaction or a one-electron process (entries 1–2).

**Table 1:**
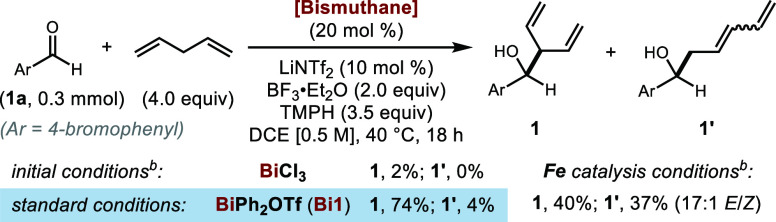

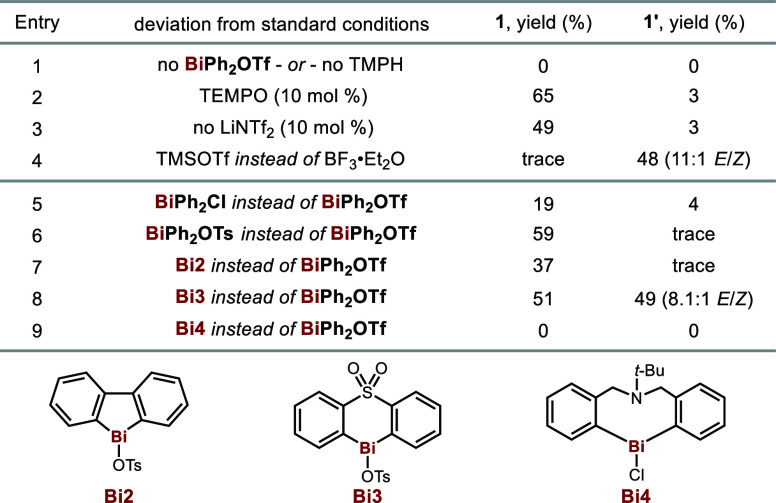
Optimization of the Bi(III)-Catalyzed Allylic C–H Functionalization^a^

a Yields were determined by ^1^H NMR spectroscopy using 2,4-dinitrotoluene as the internal standard. TMPH = 2,2,6,6-tetramethylpiperidine.

b See Supporting Information.

With the optimized conditions in hand, we investigated the generality of the Bi(III)-catalyzed allylic C–H functionalization with respect to the electrophilic coupling partner. As shown in Table 2, this protocol is applicable to a variety of carbonyl and iminium electrophiles, including aldehydes, α-keto esters, *N*-sulfonyl ketimines and *N*,*O*-acetals. The formation of linear or branched allylic functionalization products is dependent on the structure of the electrophiles as well as the choice of Lewis acid used. In addition, we found that **Bi2** stood out as an exceptional catalyst for α-keto ester substrates (see Supporting Information).

**Table 2:**
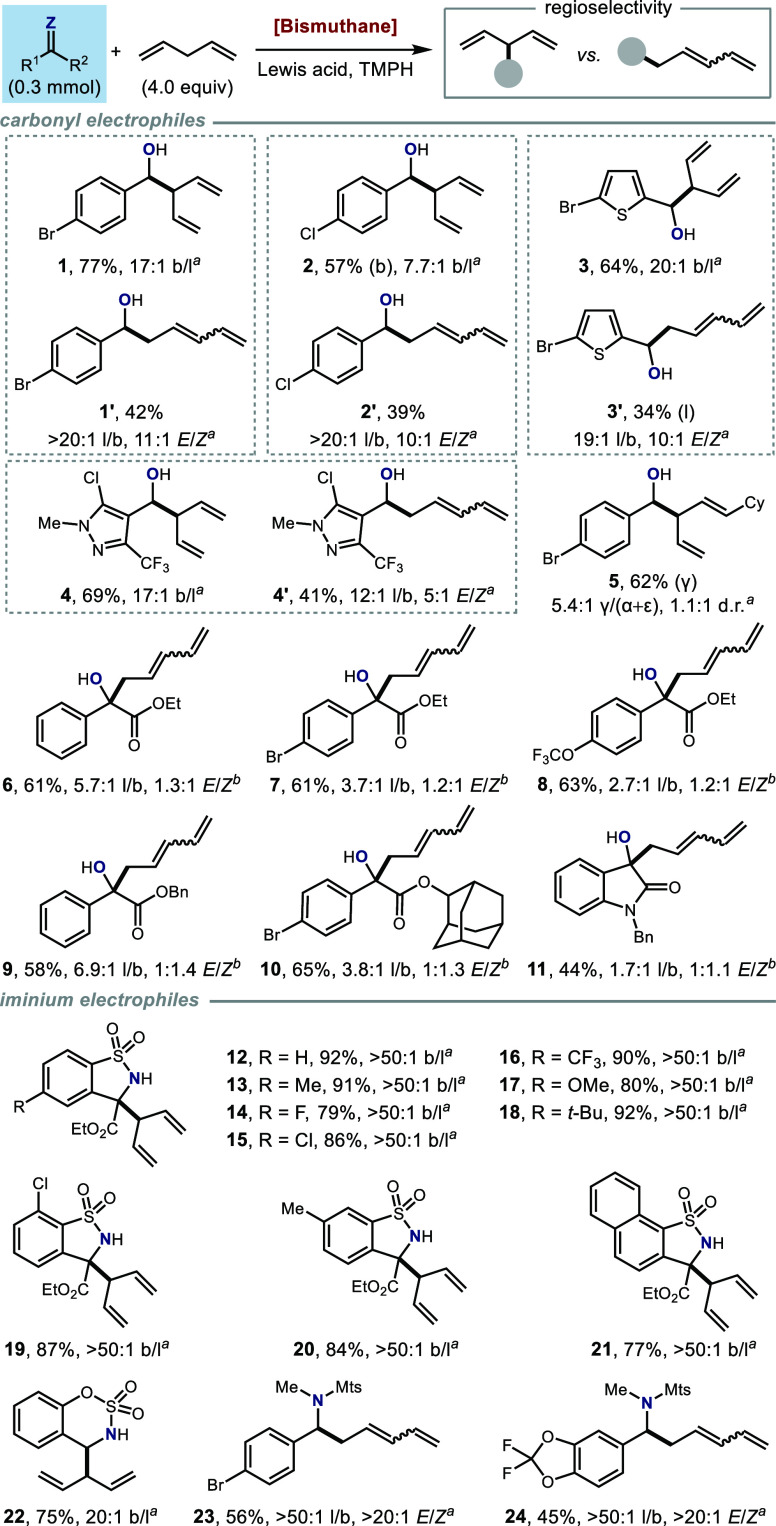
Scope of the Electrophiles

Isolated yields reported. *^a^***Bi1** was used as the catalyst.

b **Bi2** was used as the catalyst. Detailed reaction conditions provided in Supporting Information.

Subsequently, the scope of the olefins was examined with the employment of ketimine **19a** as the coupling partner (Table 3). In general, the reaction proceeded efficiently with high regioselectivity and diastereoselectivity (>9:1 b/l, >5:1 d.r.). A range of electronically distinct allylarenes were well tolerated (**25**–**31**), as were olefins containing heteroarenes (**32**–**35**). Unactivated alkenes (**36**, **37**) could also be functionalized regioselectively, though in low to modest yields. Several 1,4-dienes bearing alkyl substituents were also found to be compatible substrates, providing consistently high levels of regioselectivity (**38**–**42**). X-ray crystallographic analysis of compounds **25** and **42** was conducted to confirm the relative configuration of the major diastereomer. Moreover, the synthetic value of this method was demonstrated through the transformation of products into derivatives of higher molecular complexity (**43**, **45**).

**Table 3:**
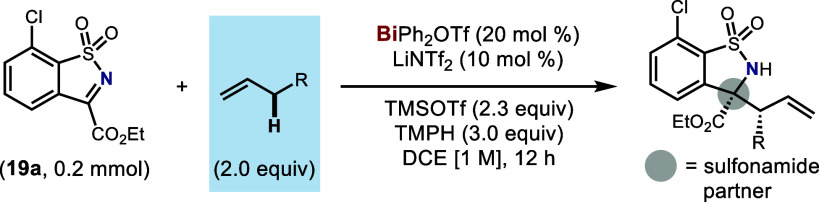

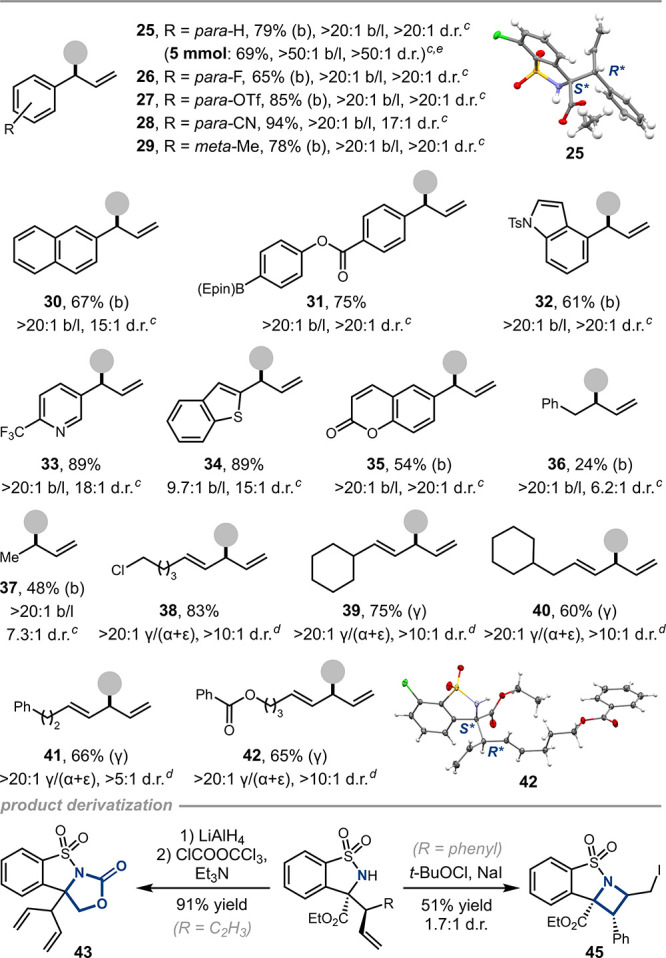
Scope of the Olefins

Isolated yields reported.

c Temperature 70 °C.

d Temperature 40 °C.

e 18 h, further purified by recrystallization. For cases **38**–**42**, >20:1 *E*/*Z*. For cases **30**, **39**–**42**, isolated yields of major diastereomers reported. For case **37**, excess 1-butene (5.5 equiv) was used.

Experimental and theoretical evidence of a substantial intermolecular Bi–olefin coordination has remained elusive to date,^20^ and weak intramolecular interactions have only recently been discovered.^18c,21^ Hence, we conducted systematic studies on the interactions between cationic Bi(III) compounds and simple olefins in the context of mechanistic investigations of this catalytic process. First, the relevant Bi(III) compound was mixed with an olefin in CD_2_Cl_2_ (1 equiv each) and the change in chemical shift of their NMR spectroscopic signals was recorded. BiMe_2_SbF_6_ and BiPh_2_SbF_6_ were used as the Bi source,^22^ due to their low steric hindrance and good solubility in weakly coordinating solvents. Among the tested olefins, cyclopentene exhibited the most significant interaction with Bi, resulting in a downfield shift in the resonance of its vinyl protons by 0.25–0.26 ppm (Scheme 2A). Additionally, red-shift of IR frequencies of the C=C bonds was observed, and the Bi–olefin adducts were detected by HRMS (Scheme 2B). 1,4-Pentadiene and allylbenzene, which were used as substrates in catalytic reactions, were also found to bind to Bi, albeit more weakly. These are the first examples of intermolecular Bi–olefin interactions unequivocally established in solution. We were thus interested in understanding the nature and strength of such interactions. By means of Natural Bond Orbital (NBO) and Intrinsic Bond Orbital (IBO) analysis we found a significant interaction between the olefin π-bond and the lone pair* orbital of the Bi center, which is associated with interaction energies of 15.6–20.6 kcal/mol and varies depending on the olefin and the Bi coordination environment (Scheme 2C). A frontier orbital analysis revealed bonding and antibonding Bi–olefin interactions in the HOMO and the LUMO, respectively, which show considerable contributions by *p*(Bi) and π(C=C) orbitals, exemplarily shown for 1,4-pentadiene and BiMe_2_SbF_6_ in Scheme 2D (see Supporting Information for additional details).

**Scheme 2:**
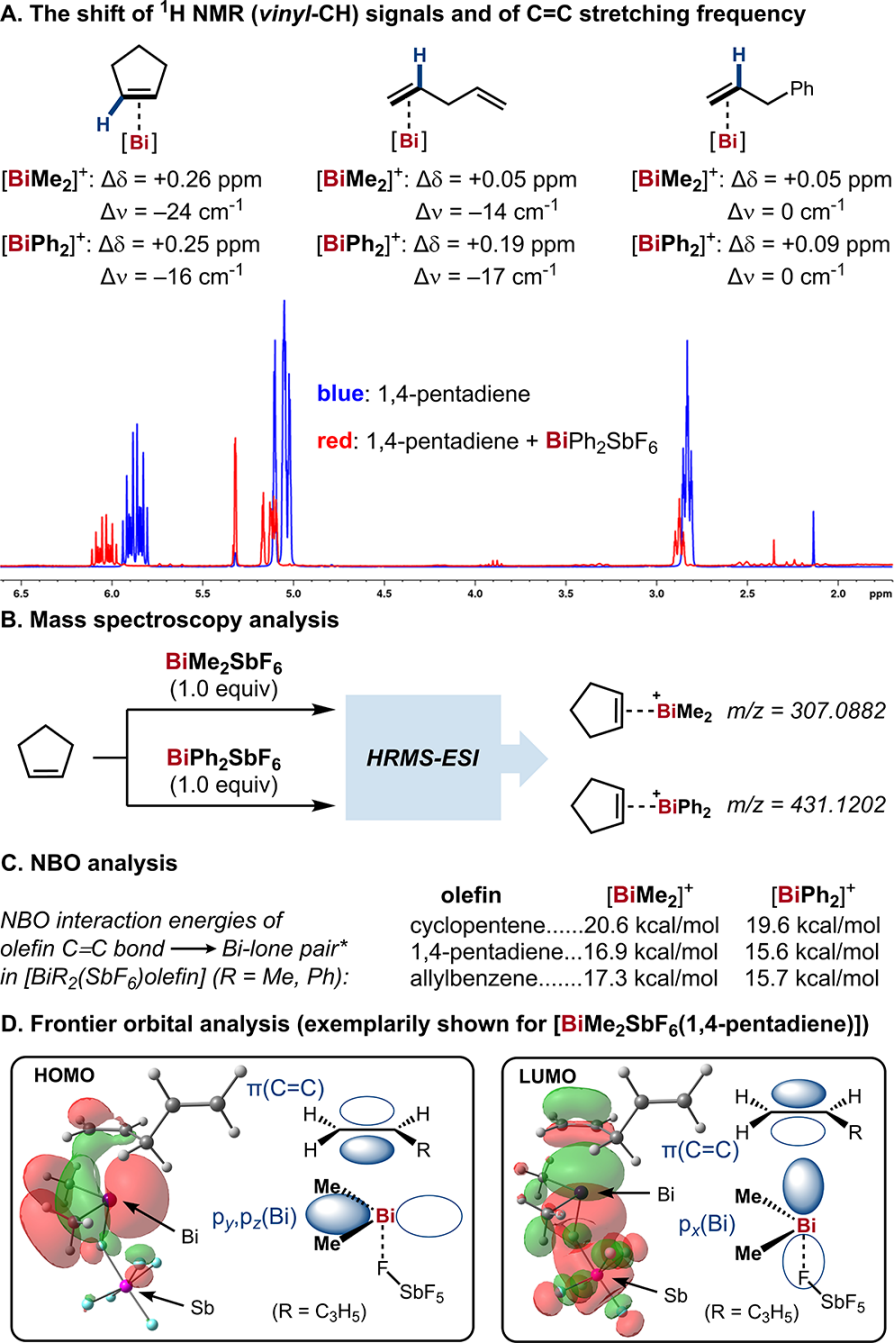
Identification of Bi–Olefin Interactions

We further performed investigations to determine whether our catalytic reactions proceed through a C–H deprotonation process. To begin with, the kinetic isotope effect was examined through independent rate measurements (*k*_H_/*k*_D_ = 4.1) and a competition experiment (*P*_H_/*P*_D_ = 4.9), indicating that C–H bond cleavage is likely involved in a rate-determining step (Scheme 3A). We then conducted stoichiometric reactions to test our hypothesis regarding the formation of an allylbismuth intermediate mediated by the amine base (Scheme 3B). Pleasingly, a small amount of new olefinic species was detected by NMR spectroscopy after the reaction with 1,4-pentadiene had progressed for 1 h (see Supporting Information). Subsequently, the reaction mixture was analyzed by HRMS, and a peak corresponding to the pentadienylbismuth cation with coordinated TMPH was observed. Allylbismuth derivatives are known to be unstable and have been suggested to readily undergo decomposition via radical pathways.^23^ During the course of the stoichiometric reaction, we observed by NMR spectroscopy the formation of deca-1,3,7,9-tetraene concomitant with a gradual attenuation of the new olefin signals. In addition, a radical trap, *N*-*tert*-butyl-α-phenylnitrone (PBN), was able to intercept the pentadienyl radical in the reaction mixture, as evidenced by EPR spectroscopy (Scheme 3C and Supporting Information). Both observations evinced the typical radical leaving group character of an allylic ligand on Bi, thus providing complementary evidence for the key proposed intermediate.

**Scheme 3:**
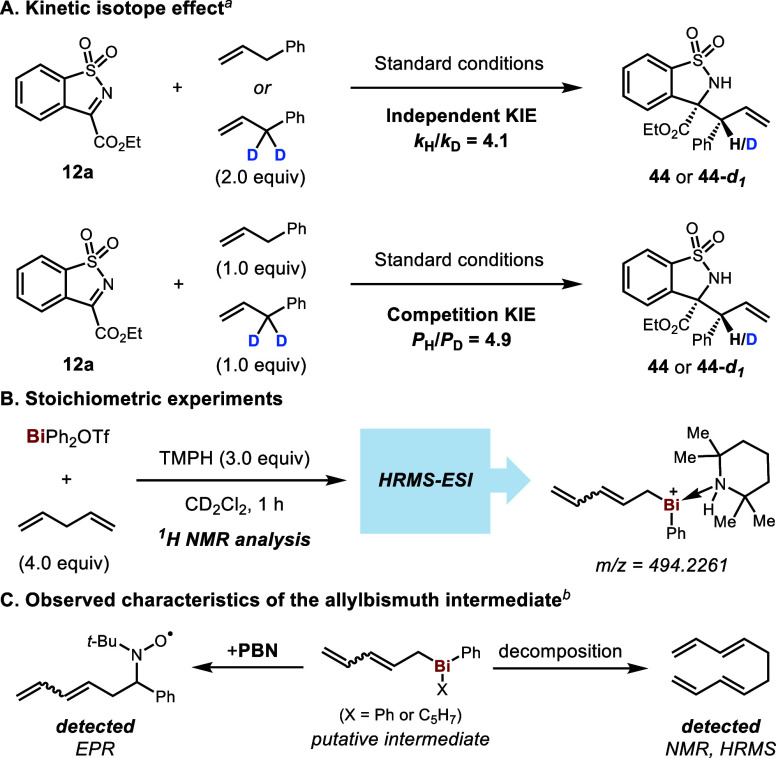
Mechanistic Insights into the C–H Deprotonation Step Standard conditions: BiPh_2_OTf (20 mol %), TMSOTf (2.3 equiv), TMPH (3.0 equiv), DCE (1.0 M), 70 °C. See Supporting Information for conditions.

We conducted a theoretical investigation to shed light on the most plausible reaction mechanism. Our study suggests that the reaction occurs via a Bi(III)-pathway involving an allylbismuth species. This species is proposed to be formed by TMPH-mediated deprotonation of the olefin upon coordination with the soft Bi(III) center. The subsequent process leading to the C–C coupling product is predicted to occur through attack of the electrophilic substrate (S_E_2′) via a closed transition state. Other pathways involving outer-sphere attack of the electrophile, insertion into the Bi–C bond, or the addition of a free radical to the carbonyl/iminium substrate were found to have higher overall free energy barriers (and are inconsistent with the observed regioselectivity) and are therefore unlikely scenarios (see Supporting Information).

In Figure 1 we present the computed free energy surface for the formation of **13** via the most plausible reaction mechanism. The reaction commences by coordination of TMPH to BiPh_2_OTf to produce **Int-1** in an exergonic process (*ΔG* = −5.4 kcal/mol). Next, ligand exchange between the triflate and olefin substrate via **Int-2** leads to the formation of **Int-3**, in which the key Bi–olefin interactions take place. Subsequently, **Int-3** undergoes deprotonation by TMPH via **TS-1**, overcoming an overall free energy barrier of +23.5 kcal/mol. Concomitant formation of TMPH_2_^+^ leads to the formation of the thermodynamically favored and experimentally validated allylbismuth species **Int-4** (*ΔG*_*rel*_ = +2.8 kcal/mol).

**Figure 1:**
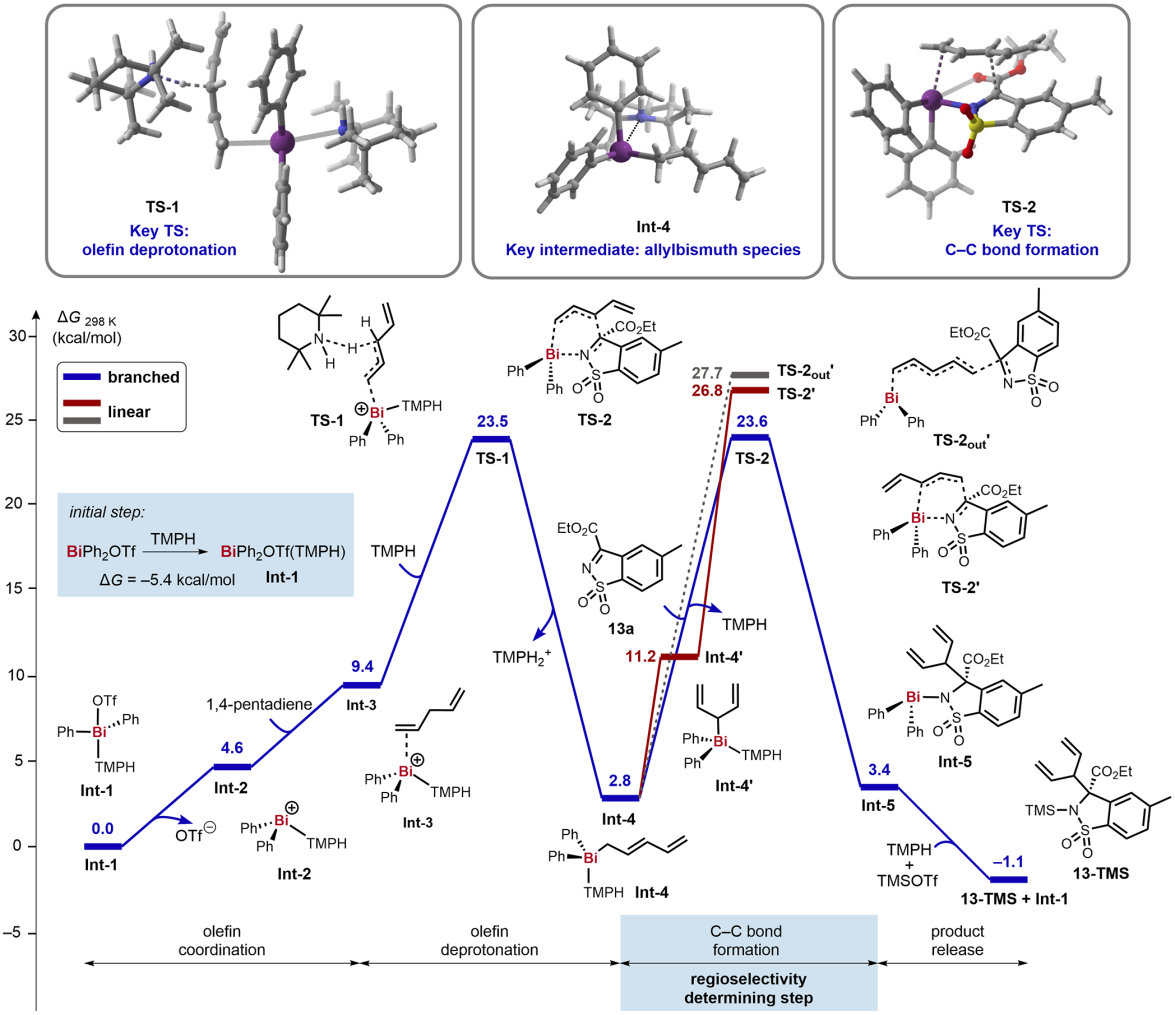
Computational mechanistic studies. Calculations were performed at the M06-L+GD3/def2-TZVP(C,H,N,O,S,F)/LanL2DZ(Bi,Si) (DCE,SMD)//M06-L+GD3/def2-SVP/LanL2DZ(Bi,Si) level of theory. Int, intermediate, TS, transition state.

Electrophilic substrate **13a** is predicted to react with **Int-4** to generate **Int-5** via closed transition state **TS-2**, accounting for an overall free energy barrier of +23.6 kcal/mol. This barrier is very close to that of the previous deprotonation step (+23.5 kcal/mol overall), and therefore, we propose that both of these events are partially rate determining. In addition, computed inner- and outer-sphere transition states that lead to the linear allylation product were found to have higher activation barriers (**TS-2′** and **TS-2**_**out**_**′**), in line with the experimentally observed >50:1 b/l regioselectivity. Finally, TMSOTf reacts with **Int-5** to afford product **13-TMS** and to regenerate active catalytic species **Int-1** upon coordination with TMPH.^24^

In summary, a novel Bi(III)-based catalytic system for C(sp^3^)–H functionalization has been established. This transformation represents a distinct method for the coupling of olefins at the allylic position with diverse electrophilic reagents to give products with unique selectivities. In-depth spectroscopic and computational studies revealed the presence and nature of heretofore elusive Bi–olefin interactions. The key elementary step involving base-mediated C–H deprotonation within the catalytic cycle was also elucidated through experiments and calculations. Further efforts using main-group catalysts to address longstanding selectivity challenges in C–H functionalization are underway and will be reported in due course.

## Supporting Information Available

Experimental procedures, characterization of new compounds, X-ray crystallographic data, computational details and results, and copies of NMR spectra. (PDF)
