# Supplementary material for: Transition Metal Mimetic π-Activation by Cationic Bismuth(III) Catalysts for Allylic C–H Functionalization of Olefins Using C=O and C=N Electrophiles
Source: J Am Chem Soc. 2024 Aug 5;146(32):22122–8. doi: 10.1021/jacs.4c06235 (PMC11328129; doi:10.1021/jacs.4c06235)
Supplement: Supplementary file 1 — ja4c06235_si_001.pdf [file ja4c06235_si_001.pdf]

Supporting Information for

Transition Metal Mimetic π-Activation by Cationic Bismuth(III) Catalysts for Allylic C−H Functionalization of Olefins Using C=O and C=N Electrophiles

Ruihan Wang,[a] Sebastián Martínez,[b]‡ Johannes Schwarzmann,[b]‡ Christopher Z. Zhao,[a] Jacqueline Ramler,[b] Crispin Lichtenberg,[b]* and Yi-Ming Wang[a]*

[a] Department of Chemistry, University of Pittsburgh, Pittsburgh, Pennsylvania 15260, United States;

[b] Department of Chemistry, Philipps-University Marburg, Hans-Meerwein-Str. 4, 35032 Marburg, Germany

Table of Contents

General information 3
Initial investigations 4
Reaction optimization 5
Control experiments 10
General procedures for catalytic reactions 12
Characterization data for products 16
Product derivatization 37
Identification of bismuth—olefin interactions 45
Part I: Analysis with [$BiMe_2(SbF_6)$] 45
NMR-Spectroscopy 45
IR Spectroscopy 52
Mass spectrometry 57
Part II: Analysis with [$BiPh_2(SbF_6)$] 58
NMR Spectroscopy 58
IR Spectroscopy 62
Mass spectrometry 66
Part III: Analysis with [$BiPh_2OTf$] 68
NMR Spectroscopy 68
IR Spectroscopy 68
Kinetic isotope effect experiments 69
Stoichiometric experiments 71
Computational Mechanistic Studies 76
Synthesis of cationic bismuth complexes 92
Procedures for substrate synthesis and characterization data 96
X-ray structures 102
Copies of NMR spectra 107

Cartesian coordinates (Å) for DFT calculations 185
References 218

General information

General Reagent Information: Anhydrous 1,2-dichloroethane (DCE, Aldrich, Sure/Seal packaging), anhydrous chlorobenzene (Acros, AcroSeal packaging), anhydrous toluene (Aldrich, Sure/Seal packaging) and anisole (Fluka) were obtained from commercial suppliers and were deoxygenated by sparging with nitrogen, transferred into an argon-filled glovebox, and used without further purification. 1,4-Pentadiene, allylbenzene and trimethylsilyl trifluoromethanesulfonate (TMSOTf) were purchased from Thermo Scientific Chemicals. Boron trifluoride etherate was purchased from Sigma Aldrich. 2,2,6,6-Tetramethylpiperidine (TMPH) was purchased from Chem-Impex Int'l. Inc. and was redistilled and obtained as a pale yellow liquid before use. (*Note:* Distilled TMPH used for Bi-catalyzed reactions must be stored under inert atmosphere.) All other reagents were purchased from TCI, Thermo Scientific Chemicals, Sigma Aldrich or Combi-Blocks and were used as received. Compounds were purified by flash column chromatography using SiliCycle *SiliaFlash® F60* silica gel, unless otherwise indicated. Gaseous reactions were performed in pressure tubes (102 mm × 13 mm, Ace glass, part# 8648-61) according to literature procedure[1].

General Analytical Information: New compounds were characterized by ^{1}H NMR, ^{13}C NMR, HRMS, and, where appropriate, other analytical techniques as indicated. Copies of the ^{1}H NMR and ^{13}C NMR spectra can be found at the end of the Supporting Information. ^{1}H, ^{19}F and ^{13}C NMR spectra were recorded on Bruker 300 MHz, 400 MHz or 500 MHz instruments. All ^{1}H NMR data are reported in δ units, parts per million (ppm), and were measured relative to the residual proton signal in the deuterated solvent at 7.26 ppm ($CDCl_3$), 5.32 ppm (CD_2Cl_2), 1.94 (CD_3CN) or 3.31 (CD_3OD). All ^{13}C NMR spectra are ^{1}H decoupled and reported in ppm relative to the solvent signal at 77.16 ppm ($CDCl_3$), 53.84 ppm (CD_2Cl_2), 1.32/118.26 ppm (CD_3CN) or 49.00 (CD_3OD). 2,4-Dinitrotoluene was used as the internal standard for yields determined by ^{1}H NMR spectroscopy. High resolution mass spectra were obtained on a Bruker Daltonics, Inc. APEXIII 7.0 TESLA FTMS instrument (ESI) or Waters Micromass GCT Premier instrument (EI). Thin-layer chromatography (TLC) was performed on Silicycle 250 μm (analytical) or 1000 μm (preparative) silica gel plates. Compounds were visualized by irradiation with UV light, or by staining with iodine/silica gel, potassium permanganate, or phosphomolybdic acid (PMA). Yields refer to isolated compounds, unless otherwise indicated.

Initial investigations

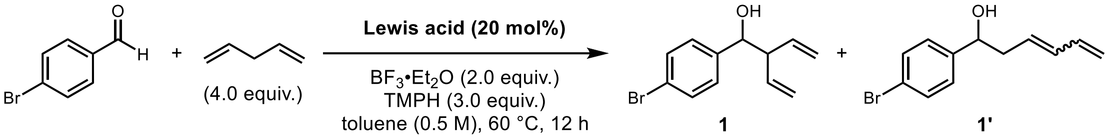

To an oven-dried reaction tube (100 mm × 13 mm, Fisherbrand, part# 14-959-35C) charged with a magnetic stir bar, Lewis acid (20 mol%), 4-bromobenzaldehyde (18.5 mg, 0.1 mmol, 1.0 equiv.), dry toluene (0.2 mL), 1,4-pentadiene (41.3 µL, 0.4 mmol, 4.0 equiv.), $BF_3{\cdot}Et_2O$ (24.7 µL, 0.2 mmol, 2.0 equiv.), TMPH (50.6 µL, 0.3 mmol, 3.0 equiv.) were added in succession in an argon-filled glovebox. The tube was capped with a Teflon/silicone septum (Thermo/National part# C4015-66A) screw cap and placed in an oil bath preheated at 60 °C with vigorous stirring. After 12 h, the tube was allowed to cool to room temperature. Yields were determined by ^{1}H NMR spectroscopy using 2,4-dinitrotoluene as the internal standard.

Entry	Lewis acid	% yield of **1**	% yield of **1'**
1	LiOTf	0	0
2	NaOTf	0	0
3	$Mg(OTf)_2$	0	0
4	$Ca(OTf)_2$	0	0
5	$Ba(OTf)_2$	0	0
6	BPh_3	0	0
7	$AlCl_3$	0	0
8	$Ga(OTf)_3$	0	0
9	$In(OTf)_3$	15	14 (13:1 *E*/*Z*)
10	$Sn(OTf)_2$	0	0
11	$Sn(NTf_2)_4$	0	0
12	$SbCl_3$	0	0
13	$BiCl_3$	2	0

Reaction optimization

The condition optimization experiments were performed based on the result of entry 13 of the table above (initial investigations). The procedures are the same as those of the initial investigations. Yields were determined by ^{1}H NMR spectroscopy using 2,4-dinitrotoluene as the internal standard.

1. Aldehyde substrate

1.1. Solvent test

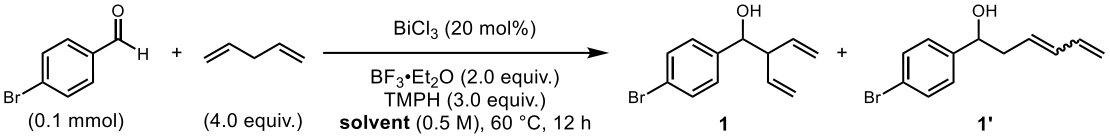

Entry	Solvent	% yield of **1**	% yield of **1’**
1	DCE	8	0
2	$PhCF_3$	0	0
3	PhCl	6	0

1.2. Test for Bi(III) complexes

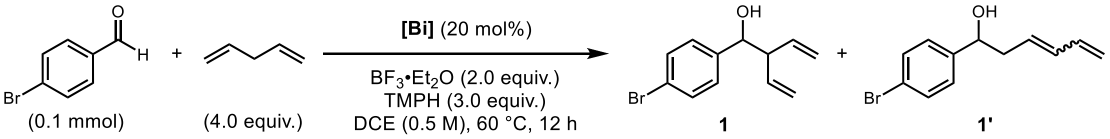

Entry	bismuthane	% yield of **1**	% yield of **1’**
1	$BiBr_3$	8	0
2	$Bi(OTf)_3$	0	0
3	$BiPh_2Cl$	6	0
4	$BiPhCl_2$	9	0
5	$BiPh_2Br$	6	Trace
6	$BiPh_2OTs$	8	0
7	$BiPh_2OTf$	51	13 (2.5:1 *Z*/*E*)
8	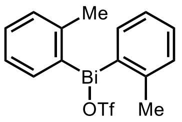	16	1
9	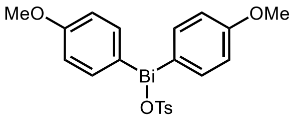	22	Trace
10	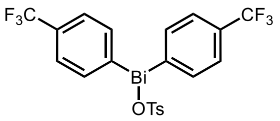	9	0

1.3. Other conditions

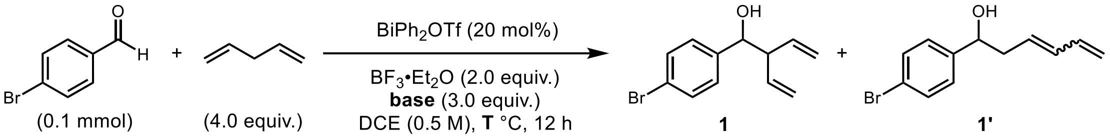

Entry	base	temp. °C	% yield of **1**	% yield of **1’**
1	Et_3N	60	0	0
2	*sym*-collidine	60	3	0
3	TMPH	40	42	3

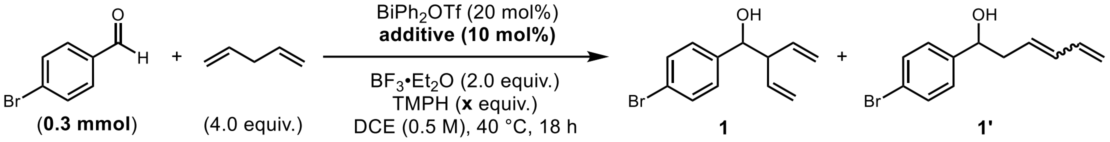

Entry	**x** equiv.	additive	% yield of **1**	% yield of **1’**
1	3.0	—	54	6
2	3.5	—	49	3
3	3.5	$LiNTf_2$	74	4

The increased yield in the presence of the additive $LiNTf_2$ (entry 3 in the table above) suggests that ligand exchange between $LiNTf_2$ and $BiPh_2OTf$ to give LiOTf and the more Lewis-acidic $BiPh_2NTf_2$ is viable under the given conditions.

1.4. TMSOTf instead of $BF_3 \cdot Et_2O$

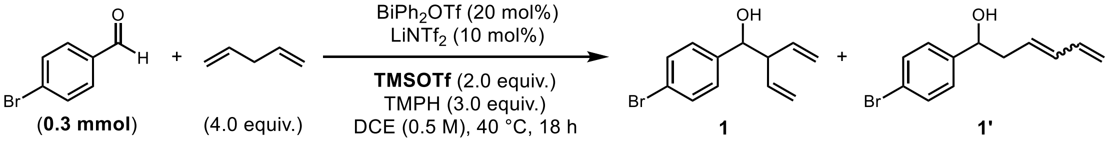

% yield of **1**: trace
% yield of **1’**: 48 (11:1 *E*/*Z*)

1.5. Test for Bi(III) complexes with complex ligand scaffolds

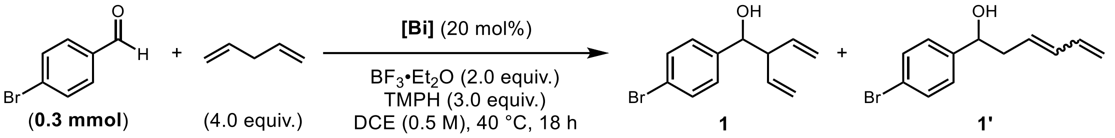

Entry	Bismuthane	% yield of **1**	% yield of **1'**
1	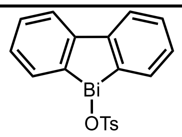	37	trace
2	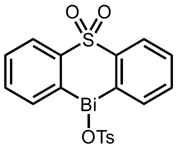	51	49 (8.1:1 *E*/*Z*)
3	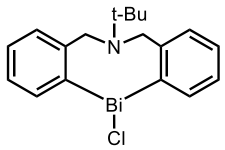	0	0

1.6. Test for Fe(II) catalyzed conditions

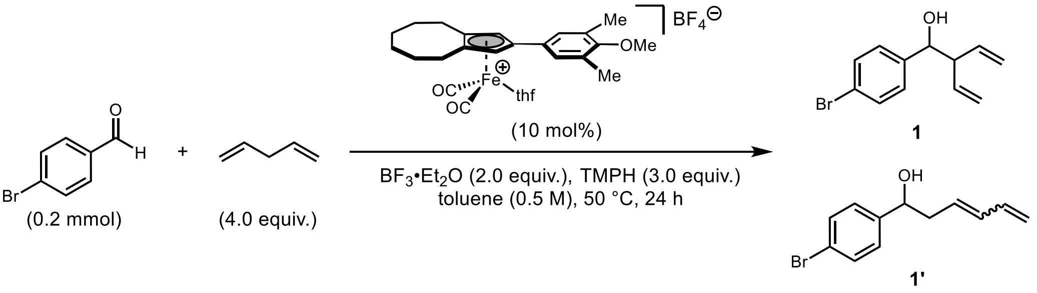

To an oven-dried reaction tube (100 mm × 13 mm, Fisherbrand, part# 14-959-35C) charged with a magnetic stir bar, Fe(II) catalyst[1] (11.0 mg, 10 mol%), 4-bromobenzaldehyde (37 mg, 0.2 mmol, 1.0 equiv), 1,4-pentadiene (83 μL, 0.8 mmol, 4.0 equiv), dry toluene (0.4 mL), $BF_3·Et_2O$ (49 μL, 0.4 mmol, 2.0 equiv), TMPH (101 μL, 0.6 mmol, 3.0 equiv) were added in succession in an argon-filled glovebox. The tube was capped with a Teflon/silicone septum (Thermo/National part# C4015-66A) screw cap and placed in an oil bath preheated at 50 °C with vigorous stirring. After 24 h, the tube was allowed to cool to room temperature. The crude mixture was concentrated *in vacuo* and purified by flash column chromatography (eluent: 15:1 → 10:1 hexanes/EtOAc) on silica gel to obtain the desired products.

% yield of **1**: 40%
% yield of **1'**: 37% (17:1 *E*/*Z*)

2. α-Keto ester substrate

2.1. General conditions

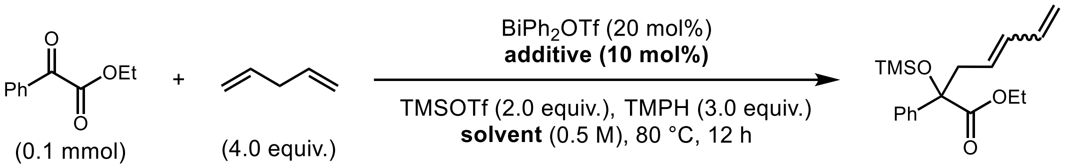

Entry	solvent	additive	% yield of linear product	r.r. (α/γ)	*E*/*Z*
1	DCE	—	39	16:1	1:1.5
2	$PhCF_3$	—	29	15:1	1:1.3
3	Toluene	—	32	>20:1	1:1.2
4	PhCl	—	39	>30:1	1:1.4
5	PhCl	$LiNTf_2$	46	12:1	1:1.1

2.2. Test for Bi(III) complexes

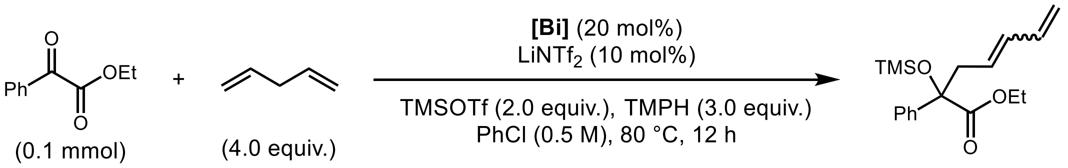

Entry	Bismuthane	% yield of linear product	r.r. (α/γ)	*E*/*Z*
1	$BiPh_2Cl$	33	7.7:1	1.3:1
2	$BiPhCl_2$	36	9.8:1	1.5:1
3	$BiPh_2Br$	16	3.8:1	1.5:1
4	$BiPh_2OTs$	43	15:1	1.0:1
5	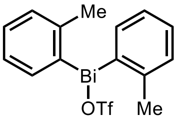	52	16:1	1.7:1
6	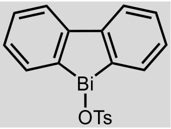	63	4.9:1	1.3:1

3. Ketimine substrate

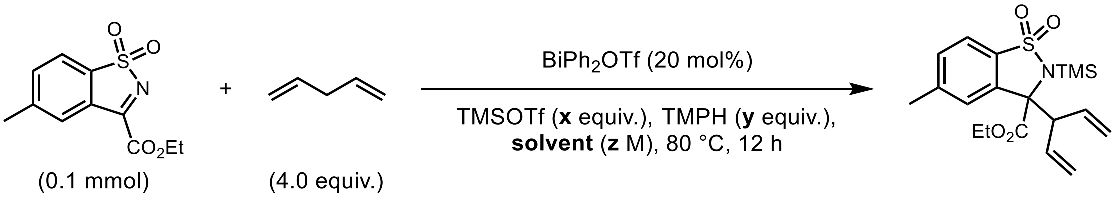

Entry	**x** equiv.	**y** equiv.	**z** M	Solvent	% yield	r.r. (γ/α)
1	2	3	0.5	PhBr	46	13:1
2	2	3	0.5	PhCl	47	12:1
3	2	3	0.5	PhF	41	8.3:1
4	2	3	0.5	DCE	58	>20:1
5	2	3	1.0	DCE	91	20:1
6	2.5	3	1.0	DCE	91	32:1
7	2.8	2	1.0	DCE	91	43:1
8	2	2	1.0	DCE	82	>20:1

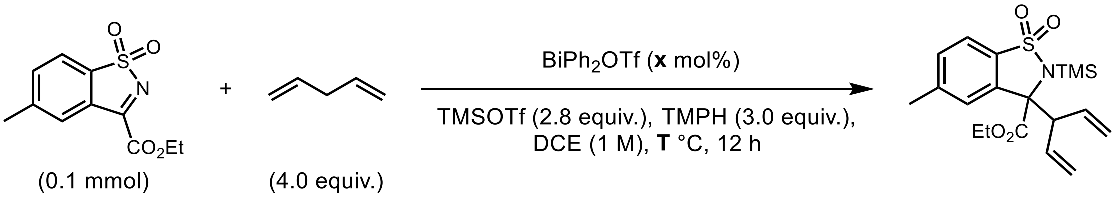

Entry	**x** mol%	temp. °C	% yield	r.r. (γ/α)
1	10	80	78	44:1
2	20	70	99	>50:1
3	15	70	98	>50:1
4	10	70	80	>50:1

Control experiments

1. Test for ene-type reaction

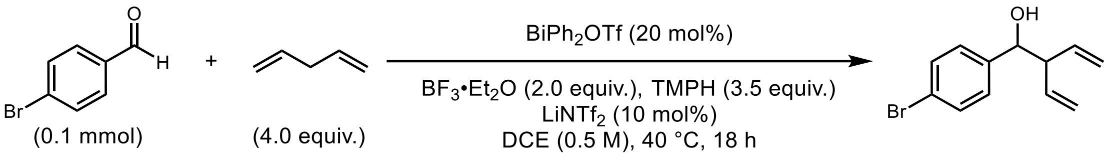

Entry	$BiPh_2OTf$	$BF_3{\cdot}Et_2O$	TMPH	$LiNTf_2$	% Yield
1		✓	✓	✓	0
2		✓		✓	0
3	✓		✓		0
4	✓				0
5	✓	✓		✓	0

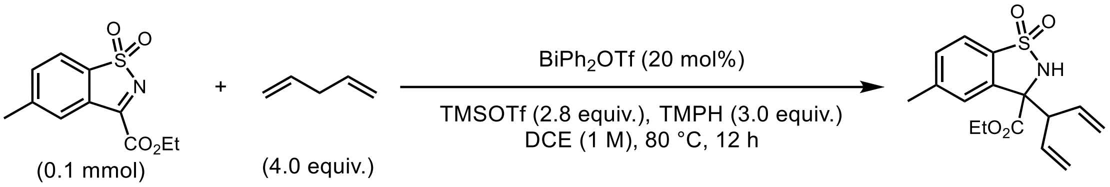

Entry	$BiPh_2OTf$	TMSOTf[a]	TMPH	% Yield
1		✓	✓	0
2	✓	✓		0
3	✓		✓	1
4	✓			0

For a mechanistic investigation of the reaction described in this table, see main text. [a]According to mechanistic studies, TMSOTf helps to thermodynamically drive the reaction and re-generates the bismuth catalyst (see main text and computational part).

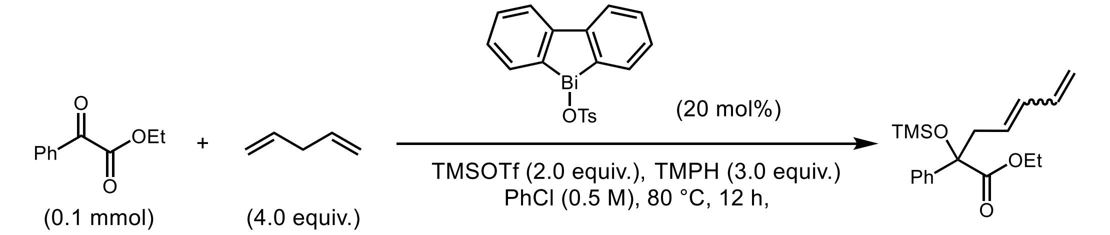

Entry	[Bi]	TMSOTf	TMPH	% Yield
1	✓			0
2		✓	✓	0
3		✓		0

2. Test for radical process

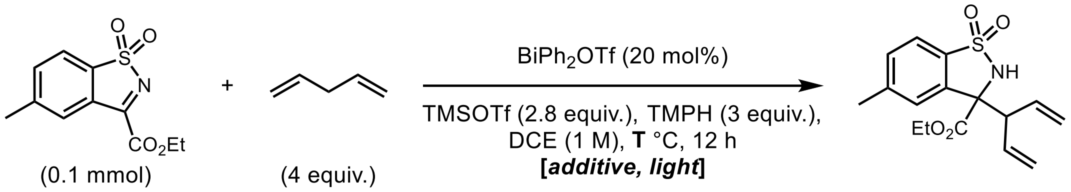

Entry	Temp. °C	Additive	Light	% Yield	r.r. (b/l)
1	80	—	—	92	>20:1
2	80	BHT (0.2 equiv.)	—	79	>99:1
3	80	dihydroanthracene (0.5 equiv.)	—	92	>20:1
4	22	—	white light[a]	50	7.4:1
5	22	—	dark	41	>99:1

[a]85 W fluorescent lamp; borosilicate glass.

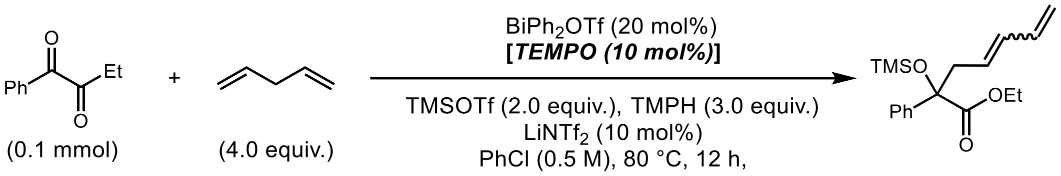

Entry	TEMPO	% Yield	*E*/*Z*
1	—	46	1.1:1
2	✓	39	1.0:1

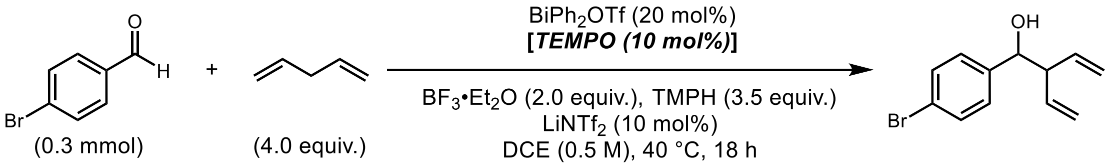

Entry	TEMPO	% Yield
1	—	74
2	✓	65

General procedures for catalytic reactions

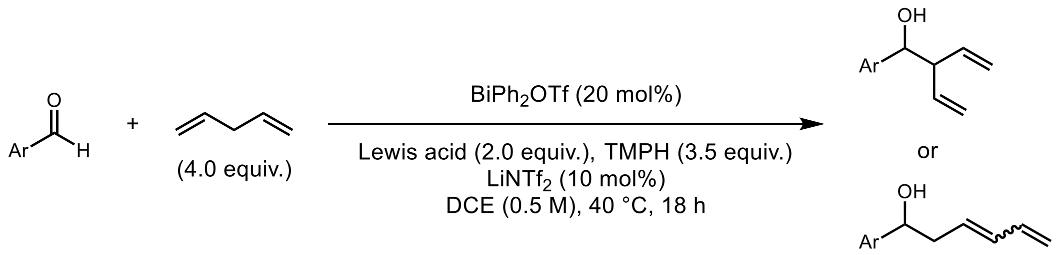

General procedure A1: To an oven-dried reaction tube (100 mm × 13 mm, Fisherbrand, part# 14-959-35C) charged with a magnetic stir bar, $BiPh_2OTf$ (30.7 mg, 20 mol%), $LiNTf_2$ (8.6 mg, 10 mol%), aldehyde (0.3 mmol, 1.0 equiv.), dry DCE (0.6 mL), 1,4-pentadiene (123 µL, 1.2 mmol, 4.0 equiv.), $BF_3{\cdot}Et_2O$ (74.0 µL, 0.6 mmol, 2.0 equiv.) or TMSOTf (108 µL, 0.6 mmol, 2.0 equiv.), TMPH (177 µL, 1.1 mmol, 3.5 equiv.) were added in succession in an argon-filled glovebox. The tube was capped with a Teflon/silicone septum (Thermo/National part# C4015-66A) screw cap and placed in an oil bath preheated at 40 °C with vigorous stirring. After 18 h, the tube was allowed to cool to room temperature. The crude mixture was filtered through a short pad of silica to remove insoluble materials, washed with CH_2Cl_2, and concentrated *in vacuo*. The mixture was purified by flash column chromatography on silica gel to obtain the desired products as the mixture of branched and linear isomers. The overall yields were reported, unless otherwise indicated. The r.r. and *E*/*Z* ratios were determined by ^{1}H NMR spectroscopic analysis of the crude material.

Work-up procedure of the reaction with TMSOTf as Lewis acid: After completion of the catalytic reaction, the crude mixture was filtered through a short pad of silica to remove insoluble materials, and was washed with EtOAc (10 mL). Then TBAF (0.4 mL, 1 M in THF) was added, and the mixture was stirred at room temperature overnight. Then the mixture was washed with brine (5 mL × 2), the organic layer was collected, dried over magnesium sulfate and concentrated *in vacuo*. The product was obtained by purification by flash column chromatography on silica gel.

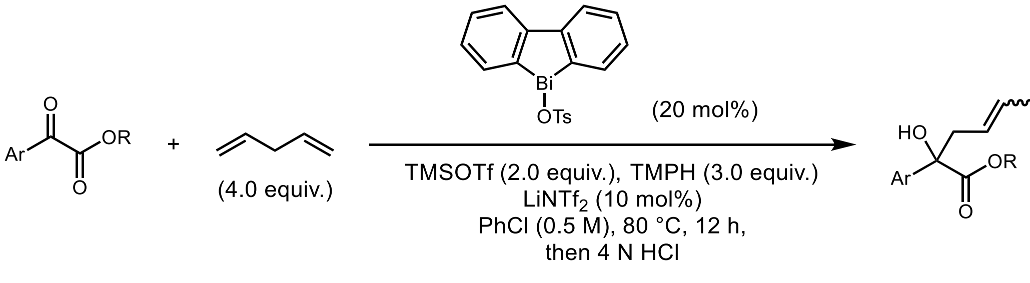

General procedure A2: To an oven-dried reaction tube (100 mm × 13 mm, Fisherbrand, part# 14-959-35C) charged with a magnetic stir bar, bismuth catalyst (31.9 mg, 20 mol%), $LiNTf_2$ (8.6 mg, 10 mol%), α-keto ester (0.3 mmol, 1.0 equiv.), dry PhCl (0.6 mL), 1,4-pentadiene (123 µL, 1.2 mmol, 4.0 equiv.), TMSOTf (108 µL, 0.6 mmol, 2.0 equiv.), TMPH (152 µL, 0.9 mmol, 3.0 equiv.) were added in succession in an argon-filled glovebox. The tube was capped with a Teflon/silicone septum (Thermo/National part# C4015-66A) screw cap and placed in an oil bath preheated at 80 °C with vigorous stirring. After 12 h, the tube was allowed to cool to room temperature. The crude mixture was filtered through a short pad of silica to remove insoluble materials, washed with CH_2Cl_2 (10 mL), and concentrated *in vacuo*. The

resulting mixture was diluted with CH_2Cl_2 (2 mL) and was treated with 4 N HCl in dioxane (0.5 mL) to completely remove the TMS group from the product. After stirring at room temperature for 4 h, the mixture was purified by flash column chromatography on silica gel to obtain the desired products as the mixture of linear and branched isomers. The two regio-isomers are separable, and the overall yields were reported. The r.r. and *E*/*Z* ratios were determined by ^{1}H NMR spectroscopic analysis of the crude material.

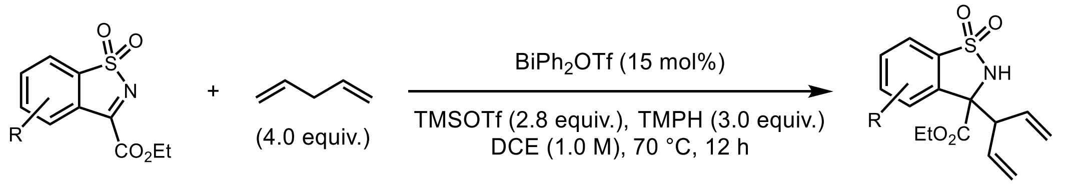

General procedure A3: To an oven-dried reaction tube (100 mm × 13 mm, Fisherbrand, part# 14-959-35C) charged with a magnetic stir bar, $BiPh_2OTf$ (23.1 mg, 15 mol%), *N*-sulfonyl ketimine (0.3 mmol, 1.0 equiv.), dry DCE (0.3 mL), 1,4-pentadiene (123 µL, 1.2 mmol, 4.0 equiv.), TMSOTf (152 µL, 0.84 mmol, 2.8 equiv.), TMPH (152 µL, 0.9 mmol, 3.0 equiv.) were added in succession in an argon-filled glovebox. The tube was capped with a Teflon/silicone septum (Thermo/National part# C4015-66A) screw cap and placed in an oil bath preheated at 70 °C with vigorous stirring. After 12 h, the tube was allowed to cool to room temperature. The crude mixture was filtered through a short pad of silica to remove insoluble materials, washed with EtOAc (10 mL), and concentrated *in vacuo*. The resulting mixture was diluted with CH_2Cl_2 (2 mL) and was treated with silica gel (500 mg) to completely remove the TMS group from the product. After stirring at room temperature for 1.5 h, the mixture was purified by flash column chromatography on silica gel to obtain the desired products. Unless otherwise stated, the products were generated with exclusive regioselectivity (>50:1 b/l) as determined by ^{1}H NMR spectroscopic analysis of the crude material. (*Note*: the $BiPh_2OTf$ catalyst decomposed to $BiPh_3$ which can be recovered by column chromatography after reaction. For example, 9% of $BiPh_3$ was recovered in the reaction to generate compound **13**.)

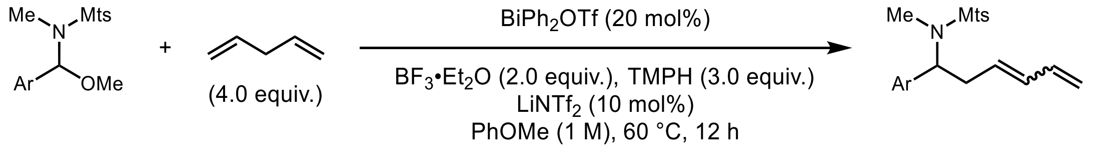

General procedure A4: To an oven-dried reaction tube (100 mm × 13 mm, Fisherbrand, part# 14-959-35C) charged with a magnetic stir bar, $BiPh_2OTf$ (20.5 mg, 20 mol%), $LiNTf_2$ (5.7 mg, 10 mol%), *N*,*O*-acetal (0.2 mmol, 1.0 equiv.), PhOMe (0.2 mL), 1,4-pentadiene (82.6 µL, 0.8 mmol, 4.0 equiv.), $BF_3{\cdot}Et_2O$ (49.4 µL, 0.4 mmol, 2.0 equiv.), TMPH (101 µL, 0.6 mmol, 3.0 equiv.) were added in succession in an argon-filled glovebox. The tube was capped with a Teflon/silicone septum (Thermo/National part# C4015-66A) screw cap and placed in an oil bath preheated at 60 °C with vigorous stirring. After 12 h, the tube was allowed to cool to room temperature. The crude mixture was filtered through a short pad of silica to remove insoluble materials, washed with CH_2Cl_2 (10 mL), and concentrated *in vacuo*. The mixture was purified by flash column chromatography on silica gel to obtain the desired products with high regioselectivity (>50:1 l/b) and high *E*/*Z* ratio (>20:1) as determined by ^{1}H NMR spectroscopic analysis of the crude material. The overall yields were reported.

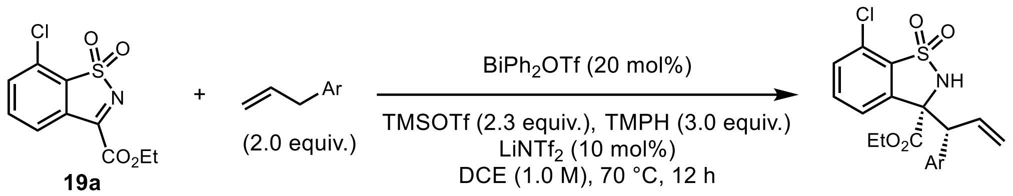

General procedure A5: To an oven-dried reaction tube (100 mm × 13 mm, Fisherbrand, part# 14-959-35C) charged with a magnetic stir bar, $BiPh_2OTf$ (20.5 mg, 20 mol%), $LiNTf_2$ (5.7 mg, 10 mol%), *N*-sulfonyl ketimine **19a** (54.7 mg, 0.2 mmol, 1.0 equiv.), dry DCE (0.2 mL), allyl arene (0.4 mmol, 2.0 equiv.), TMSOTf (83.1 µL, 0.46 mmol, 2.3 equiv.), TMPH (101 µL, 0.6 mmol, 3.0 equiv.) were added in succession in an argon-filled glovebox. The tube was capped with a Teflon/silicone septum (Thermo/National part# C4015-66A) screw cap and placed in an oil bath preheated at 70 °C with vigorous stirring. After 12 h, the tube was allowed to cool to room temperature. The crude mixture was filtered through a short pad of silica to remove insoluble materials, washed with EtOAc (10 mL), and concentrated *in vacuo*. The resulting mixture was diluted with CH_2Cl_2 (2 mL) and was treated with silica gel (500 mg) to completely remove the TMS group from the product. After stirring at room temperature for 30 min, the mixture was purified by flash column chromatography on silica gel to obtain the desired products. Unless otherwise stated, the products were generated with high regioselectivity (>20:1 b/l) as determined by ^1H NMR spectroscopic analysis of the crude material. The yields of the branched products were reported unless otherwise stated.

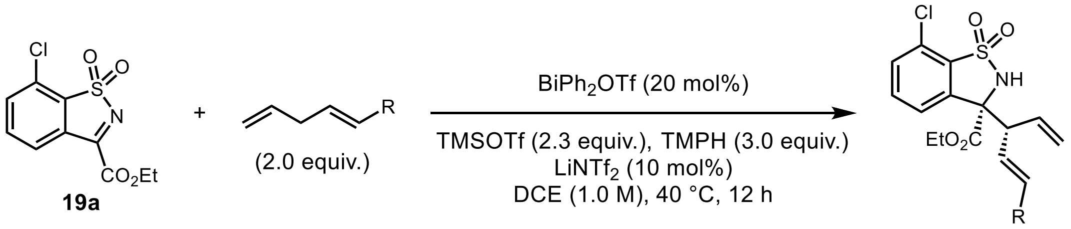

General procedure A6: To an oven-dried reaction tube (100 mm × 13 mm, Fisherbrand, part# 14-959-35C) charged with a magnetic stir bar, $BiPh_2OTf$ (20.5 mg, 20 mol%), $LiNTf_2$ (5.7 mg, 10 mol%), *N*-sulfonyl ketimine **19a** (54.7 mg, 0.2 mmol, 1.0 equiv.), dry DCE (0.2 mL), 1,4-diene (0.4 mmol, 2.0 equiv.), TMSOTf (83.1 µL, 0.46 mmol, 2.3 equiv.), TMPH (101 µL, 0.6 mmol, 3.0 equiv.) were added in succession in an argon-filled glovebox. The tube was capped with a Teflon/silicone septum (Thermo/National part# C4015-66A) screw cap and placed in an oil bath preheated at 40 °C with vigorous stirring. After 12 h, the tube was allowed to cool to room temperature. The crude mixture was filtered through a short pad of silica to remove insoluble materials, washed with EtOAc (10 mL), and concentrated *in vacuo*. The resulting mixture was diluted with CH_2Cl_2 (2 mL) and was treated with silica gel (500 mg) to completely remove the TMS group from the product. After stirring at room temperature for 1.5 h, the mixture was purified by flash column chromatography on silica gel to obtain the desired products. All the products were generated with high regioselectivity (>20:1 $\gamma/(\alpha+\varepsilon)$) and *E*/*Z* selectivity (>20:1 *E*/*Z*) as determined by ^1H NMR spectroscopic analysis of the crude material. The ratios of diastereoselectivity could not be determined accurately from crude ^1H NMR spectra, so the estimated values were reported. The diastereomers are separable, and the yields of the major diastereomer of the γ regio-isomer were reported unless otherwise stated.

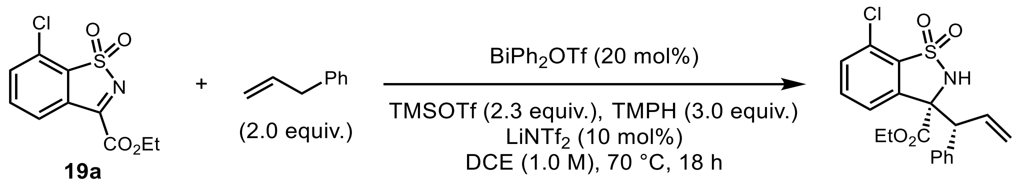

Gram-scale synthesis: To an oven-dried 25 mL round bottom flask charged with a magnetic stir bar, $BiPh_2OTf$ (512 mg, 20 mol%), $LiNTf_2$ (143 mg, 10 mol%), *N*-sulfonyl ketimine **19a** (1.37 g, 5.0 mmol, 1.0 equiv.), dry DCE (5.0 mL), allylbenzene (1.3 mL, 10.0 mmol, 2.0 equiv.), TMSOTf (2.1 mL, 11.5 mmol, 2.3 equiv.), TMPH (2.5 mL, 15.0 mmol, 3.0 equiv.) were added in succession in an argon-filled glovebox. The flask was sealed with a rubber septum and placed in an oil bath preheated at 70 °C with vigorous stirring. After 18 h, the flask was allowed to cool to room temperature. The crude mixture was treated with silica gel (3 g) to completely remove the TMS group from the product. After stirring at room temperature for 30 min, the mixture was purified by flash column chromatography on silica gel (gradient elution: 3.5:1 → 2:1 hexanes/EtOAc) followed by reprecipitating by dissolving in $CHCl_3$ (5 mL) and agitating after adding Et_2O (20 mL). The product was obtained in 69% yield (1.35 g) as a single regio- and diastereoisomer (>50:1 b/l, >50:1 d.r.).

Characterization data for products

(Signals indicated by * correspond to those from one isomer of a pair of diastereomers or *E*/*Z* isomers.)

1. 1-(4-Bromophenyl)-2-vinylbut-3-en-1-ol (1, wrh-08-109)

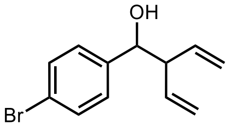

Compound **1** was prepared as a pale yellow oil (58.3 mg, 77% yield, 17:1 b/l, eluent: 10:1 hexanes/EtOAc) from 4-bromobenzaldehyde **1a** according to general procedure A1 using BF_3·Et_2O as Lewis acid.
^{1}H NMR (400 MHz, $CDCl_3$) δ 7.46 (d, *J* = 8.2 Hz, 2H), 7.19 (d, *J* = 8.1 Hz, 2H), 5.88 – 5.76 (m, 1H), 5.66 (ddd, *J* = 17.4, 10.3, 7.3 Hz, 1H), 5.24 (d, *J* = 10.3 Hz, 1H), 5.17 (d, *J* = 17.3 Hz, 1H), 5.07 (d, *J* = 10.4 Hz, 1H), 5.01 (d, *J* = 17.3 Hz, 1H), 4.55 (d, *J* = 6.5 Hz, 1H), 3.04 (q, *J* = 7.4 Hz, 1H), 2.30 – 2.19 (m, 1H).
^{13}C NMR (100 MHz, $CDCl_3$) δ 140.9, 136.45, 136.43, 131.3, 128.7, 121.6, 118.8, 117.6, 75.6, 56.3.
HRMS (ESI) calcd for $C_{12}H_{12}Br$ $[M−OH]^+$: 235.0117, found: 235.0122.

2. 1-(4-Bromophenyl)hexa-3,5-dien-1-ol (1’, wrh-08-120A)

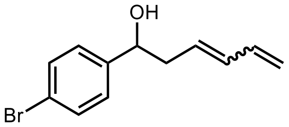

Compound **1’** was prepared as a pale yellow oil (31.7 mg, 42% yield, >20:1 l/b, 11:1 *E*/*Z*, eluent: 10:1 → 8:1 hexanes/EtOAc) from 4-bromobenzaldehyde **1a** according to general procedure A1 using TMSOTf as Lewis acid.
^{1}H NMR (400 MHz, $CDCl_3$) δ 7.51 – 7.42 (m, 2H), 7.25 – 7.19 (m, 2H), 6.31 (dt, *J* = 16.9, 10.2 Hz, 1H), 6.16 (dd, *J* = 15.3, 10.3 Hz, 1H), 5.64 (dt, *J* = 14.9, 7.4 Hz, 1H), 5.16 (dd, *J* = 16.8, 1.7 Hz, 1H), 5.04 (dd, *J* = 10.1, 1.6 Hz, 1H), 4.75 – 4.66 (m, 1H), 2.55 – 2.42 (m, 2H), 2.03 (d, *J* = 3.0 Hz, 1H).
^{13}C NMR (100 MHz, $CDCl_3$) δ 142.9, 136.7, 135.0, 131.7, 129.5, 127.7, 121.5, 116.7, 73.1, 42.8.
HRMS (ESI) calcd for $C_{12}H_{12}Br$ $[M−OH]^+$: 235.0117, found: 235.0123.

3. 1-(4-Chlorophenyl)-2-vinylbut-3-en-1-ol (2, wrh-08-164A)

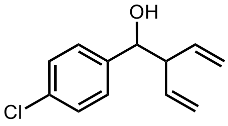

Compound **2** was prepared as a pale yellow oil (35.9 mg, 57% yield (branched isomer), 7.7:1 b/l, eluent: 6:1 → 5:1 hexanes/EtOAc) from 4-chlorobenzaldehyde **2a** according to general procedure A1 using BF_3·Et_2O as Lewis acid.
^{1}H NMR (400 MHz, $CDCl_3$) δ 7.34 – 7.27 (m, 2H), 7.27 – 7.21 (m, 2H), 5.82 (ddd, *J* = 17.1, 10.3, 8.3 Hz, 1H), 5.66 (ddd, *J* = 17.5, 10.4, 7.2 Hz, 1H), 5.24 (d, *J* = 10.3 Hz, 1H), 5.17 (d, *J* = 17.3, 1H), 5.06 (d, *J* = 10.4, 1H), 5.00 (d, *J* = 17.3, 1H), 4.56 (dd, *J* = 7.1, 2.6 Hz, 1H), 3.04 (q, *J* = 7.6 Hz, 1H), 2.23 (d, *J* = 2.9 Hz, 1H).
^{13}C NMR (100 MHz, $CDCl_3$) δ 140.4, 136.50, 136.47, 133.4, 128.40, 128.37, 118.8, 117.6, 75.6, 56.4.

HRMS (ESI) calcd for $C_{12}H_{12}Cl$ [M−OH]$^+$: 191.0622, found: 191.0627.

4. 1-(4-Chlorophenyl)hexa-3,5-dien-1-ol (2’, wrh-08-163A)

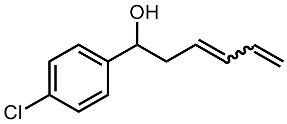

Compound **2’** was prepared as a pale yellow oil (24.2 mg, 39% yield, >20:1 l/b, 10:1 *E*/*Z*, eluent: 15:1 hexanes/EtOAc) from 4-chlorobenzaldehyde **2a** according to general procedure A1 using TMSOTf as Lewis acid.

^{1}H NMR (400 MHz, $CDCl_3$) δ 7.34 – 7.26 (m, 4H), 6.30 (dt, *J* = 16.9, 10.2 Hz, 1H), 6.15 (dd, *J* = 15.3, 10.4 Hz, 1H), 5.64 (dt, *J* = 14.9, 7.4 Hz, 1H), 5.15 (d, *J* = 16.6 Hz, 1H), 5.04 (d, *J* = 10.0 Hz, 1H), 4.71 (t, *J* = 6.3 Hz, 1H), 2.55 – 2.42 (m, 2H), 2.07 (s, 1H).

^{13}C NMR (100 MHz, $CDCl_3$) δ 142.4, 136.7, 134.9, 133.3, 129.6, 128.7, 127.3, 116.7, 73.0, 42.8.

HRMS (ESI) calcd for $C_{12}H_{12}Cl$ [M−OH]$^+$: 191.0622, found: 191.0626.

5. 1-(5-Bromothiophen-2-yl)-2-vinylbut-3-en-1-ol (3, wrh-08-171B)

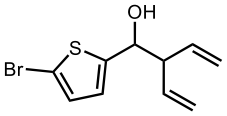

Compound **3** was prepared as a yellow oil (50.1 mg, 64% yield, 20:1 b/l, eluent: 15:1 hexanes/EtOAc) from 5-bromothiophene-2-carbaldehyde **3a** according to general procedure A1 using $BF_3{\cdot}Et_2O$ as Lewis acid.

^{1}H NMR (500 MHz, $CDCl_3$) δ 6.89 (d, *J* = 3.7 Hz, 1H), 6.74 – 6.66 (m, 1H), 5.84 (ddd, *J* = 17.1, 10.4, 8.0 Hz, 1H), 5.75 (ddd, *J* = 17.5, 10.4, 7.3 Hz, 1H), 5.32 – 5.19 (m, 2H), 5.19 – 5.07 (m, 2H), 4.77 (dd, *J* = 7.1, 3.2 Hz, 1H), 3.09 (q, *J* = 7.4 Hz, 1H), 2.40 (d, *J* = 3.7 Hz, 1H).

^{13}C NMR (125 MHz, $CDCl_3$) δ 147.4, 136.2, 135.9, 129.3, 125.2, 119.2, 118.2, 111.8, 72.6, 56.2.

HRMS (ESI) calcd for $C_{10}H_{10}BrS$ [M−OH]$^+$: 240.9681, found: 240.9684.

6. 1-(5-Bromothiophen-2-yl)hexa-3,5-dien-1-ol (3’, wrh-08-173)

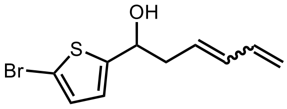

Compound **3’** was prepared as a yellow oil (26.4 mg, 34% yield (linear isomer), 19:1 l/b, 10:1 *E*/*Z*, eluent: 20:1 → 10:1 hexanes/EtOAc) from 5-bromothiophene-2-carbaldehyde **3a** according to general procedure A1 using TMSOTf as Lewis acid.

^{1}H NMR (500 MHz, $CDCl_3$) δ 6.91 (d, *J* = 3.7 Hz, 1H), 6.72 (d, *J* = 3.8 Hz, 1H), 6.32 (dt, *J* = 16.8, 10.2 Hz, 1H), 6.19 (dd, *J* = 15.0, 10.4 Hz, 1H), 5.67 (dt, *J* = 14.9, 7.3 Hz, 1H), 5.17 (d, *J* = 16.8 Hz, 1H), 5.05 (d, *J* = 10.0 Hz, 1H), 4.94 – 4.86 (m, 1H), 2.67 – 2.53 (m, 2H), 2.14 (d, *J* = 4.1 Hz, 1H).

^{13}C NMR (100 MHz, $CDCl_3$) δ 149.5, 136.6, 135.3, 129.5, 128.8, 124.1, 116.9, 111.6, 69.9, 42.4.

HRMS (ESI) calcd for $C_{10}H_{10}BrS$ [M−OH]$^+$: 240.9681, found: 240.9676.

7. 1-(5-Chloro-1-methyl-3-(trifluoromethyl)-1*H*-pyrazol-4-yl)-2-vinylbut-3-en-1-ol (4, wrh-08-110A)

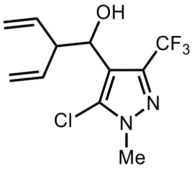

Compound **4** was prepared as a colorless oil (58.5 mg, 69% yield, 17:1 b/l, eluent: 5:1 → 4:1 hexanes/EtOAc) from 5-chloro-1-methyl-3-(trifluoromethyl)-1*H*-pyrazole-4-carbaldehyde **4a** according to general procedure A1 using $BF_3 \cdot Et_2O$ as Lewis acid.

^{1}H NMR (400 MHz, $CDCl_3$) δ 5.90 (ddd, *J* = 17.0, 10.4, 8.5 Hz, 1H), 5.57 (ddd, *J* = 17.7, 9.7, 8.1 Hz, 1H), 5.35 – 5.24 (m, 2H), 5.04 – 4.94 (m, 2H), 4.64 (dd, *J* = 9.5, 3.2 Hz, 1H), 3.87 (s, 3H), 3.32 (q, *J* = 8.5 Hz, 1H), 2.24 (d, *J* = 3.3 Hz, 1H).

^{13}C NMR (100 MHz, $CDCl_3$) δ 139.4 (q, *J* = 37.3 Hz), 137.2, 135.8, 127.9, 121.1 (q, *J* = 268 Hz), 119.1, 117.6, 117.2, 67.5, 54.9, 37.1.

^{19}F NMR (376 MHz, $CDCl_3$) δ –60.21.

HRMS (ESI) calcd for $C_{11}H_{13}ON_2ClF_3$ $[M+H]^+$: 281.0663, found: 281.0661.

8. 1-(5-Chloro-1-methyl-3-(trifluoromethyl)-1*H*-pyrazol-4-yl)hexa-3,5-dien-1-ol (4', wrh-08-165)

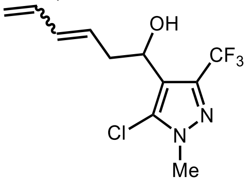

Compound **4'** was prepared as a pale yellow oil (34.7 mg, 41% yield, 12:1 l/b, 5:1 *E*/*Z*, eluent: 5:1 hexanes/EtOAc) from 5-chloro-1-methyl-3-(trifluoromethyl)-1*H*-pyrazole-4-carbaldehyde **4a** according to general procedure A1 using TESOTf (136 µL, 0.6 mmol, 2.0 equiv.) as Lewis acid, the reaction was conducted at 80 °C.

^{1}H NMR (400 MHz, $CDCl_3$) δ 6.30 (dt, *J* = 16.8, 10.2 Hz, 1H), 6.16 (dd, *J* = 15.1, 10.4 Hz, 1H), 5.64 (dt, *J* = 15.0, 7.4 Hz, 1H), 5.20 – 5.12 (m, 1H), 5.06 – 5.02 (m, 1H), 4.92 – 4.83 (m, 1H), 3.88 (s, 3H), 2.76 – 2.65 (m, 1H), 2.60 – 2.51 (m, 1H), 2.08 (d, *J* = 4.5 Hz, 1H).

^{13}C NMR (100 MHz, $CDCl_3$) δ 138.8 (q, *J* = 37.7 Hz), 136.7, 134.9, 133.0, 131.7, 129.1, 127.5, 126.3, 121.1 (q, *J* = 268 Hz), 118.9, 118.8, 116.8, 65.4, 40.3, 37.0. (Complexity of the spectrum is due to the presence of isomers and C−F coupling. Some C signals could not be unambiguously assigned.)

^{19}F NMR (376 MHz, $CDCl_3$) δ –60.49 (–60.21, –60.45 for other isomers).

HRMS (ESI) calcd for $C_{11}H_{13}ON_2ClF_3$ $[M+H]^+$: 281.0663, found: 281.0670.

9. (*E*)-1-(4-Bromophenyl)-4-cyclohexyl-2-vinylbut-3-en-1-ol (5, wrh-08-130A)

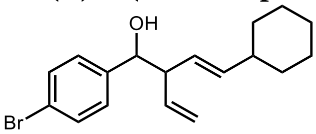

Compound **5** was prepared as a colorless oil (71.4 mg, 62% yield (γ isomer), containing 4% isomer which is likely the *Z* isomer, 5.4:1 γ/(α+ε), 1.1:1 d.r., eluent: 30:1 hexanes/EtOAc) from (*E*)-penta-1,4-dien-1-ylcyclohexane **39b** (2.0 equiv.) according to general procedure A1 using $BF_3 \cdot Et_2O$ as Lewis acid.

^{1}H NMR (500 MHz, $CDCl_3$) δ 7.48 – 7.41 (m, 2H), 7.23 – 7.12 (m, 2H), 5.79/5.64* (ddd, *J* = 17.3, 10.3, 8.2 Hz/7.0 Hz*, 1H), 5.54 (dd, *J* = 15.6, 6.6 Hz, 0.5H), 5.38 – 5.26

(m, 1H), 5.25 – 5.11 (m, 1.5H), 5.02*/4.97 (dt, J = 10.4, 1.4 Hz/17.2, 1.5 Hz, 1H), 4.51 – 4.44 (m, 1H), 2.98 – 2.89 (m, 1H), 2.26*/2.24 (s, 1H), 2.03 – 1.94*/1.89 – 1.80 (m, 1H), 1.76 – 1.51 (m, 5H), 1.33 – 0.87 (m, 5H).
^{13}C NMR (125 MHz, $CDCl_3$) δ 141.7, 141.1/141.0*, 140.1, 137.5/137.2*, 131.2*/131.1, 128.9*/128.8, 125.11/125.07*, 121.4*/121.3, 118.1/117.1*, 75.9/75.8*, 55.6/55.5*, 40.9*/40.8, 33.2/33.1*, 33.0/32.9*, 26.2, 26.11*/26.06.
HRMS (ESI) calcd for $C_{18}H_{22}Br$ $[M–OH]^+$: 317.0899, found: 317.0893.

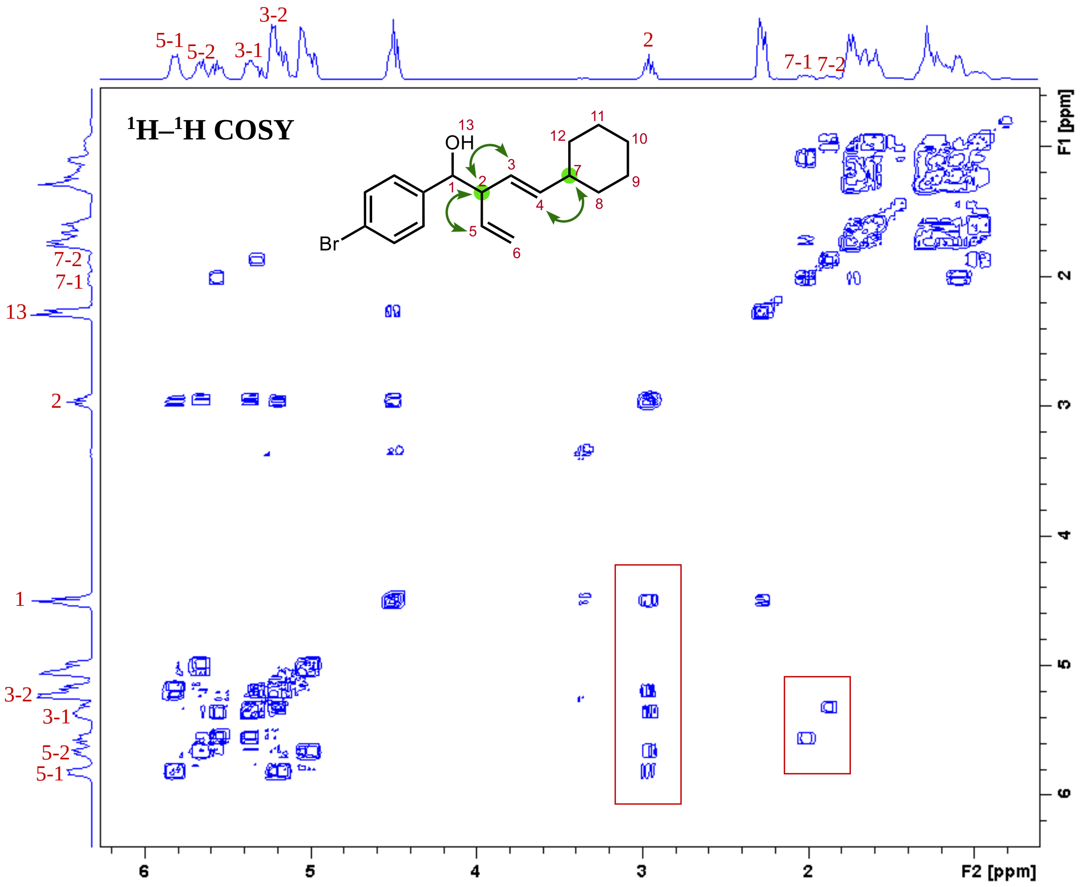

10. Ethyl 2-hydroxy-2-phenylhepta-4,6-dienoate (6, wrh-08-85A)

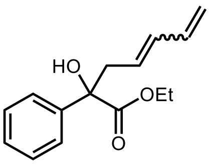

Compound **6** was prepared as a colorless oil (44.9 mg, 61% yield, 5.7:1 l/b, 1.3:1 *E*/*Z*, eluent: CH_2Cl_2) from ethyl benzoylformate **6a** according to general procedure A2.
^{1}H NMR (500 MHz, $CDCl_3$, linear) δ 7.66 – 7.58 (m, 2H), 7.39 – 7.34 (m, 2H), 7.33 – 7.28 (m, 1H), 6.73 – 6.63/6.29* (m/dt, J = 16.8, 10.3 Hz, 1H), 6.21 – 6.12 (m, 1H), 5.68*/5.50 (dt, J = 14.8, 7.3 Hz*/10.0, 7.5 Hz, 1H), 5.23 (d, J = 15.5 Hz, 0.5H), 5.18 – 5.09 (m, 1H), 5.02* (d, J = 10.5 Hz, 0.5H), 4.33 – 4.14 (m, 2H), 3.79/3.76* (s, 1H), 3.20/3.00* (dd, J = 14.6, 8.5 Hz/14.1, 8.0 Hz*, 1H), 2.86 – 2.75 (m, 1H), 1.30 – 1.24 (m, 3H).
^{1}H NMR (500 MHz, $CDCl_3$, branched) δ 7.68 – 7.64 (m, 2H), 7.35 (t, J = 7.7 Hz, 2H), 7.31 – 7.27 (m, 1H), 5.96 (ddd, J = 17.1, 10.3, 8.6 Hz, 1H), 5.66 (ddd, J = 17.4, 10.5,

7.0 Hz, 1H), 5.26 – 5.18 (m, 2H), 4.99 – 4.91 (m, 2H), 4.29 – 4.13 (m, 2H), 3.86 (s, 1H), 3.71 (t, *J* = 7.8 Hz, 1H), 1.29 (t, *J* = 7.1 Hz, 3H).
^{13}C NMR (125 MHz, $CDCl_3$, linear) δ 174.8/174.7*, 141.63/141.59*, 136.9/135.4*, 132.8*/132.3, 128.42/128.41*, 128.0, 127.98/127.95*, 125.7/125.6*, 125.5, 118.4/116.5*, 78.27*/78.25, 62.74/62.65*, 43.2*/38.2, 14.3*/14.2.
HRMS (ESI) calcd for $C_{15}H_{17}O_2$ $[M–OH]^+$: 229.1223, found: 229.1225.

11. Ethyl 2-(4-bromophenyl)-2-hydroxyhepta-4,6-dienoate (7, wrh-08-89)

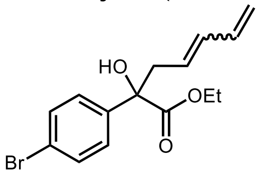

Compound **7** was prepared as a colorless oil (59.3 mg, 61% yield, 3.7:1 l/b, 1.2:1 *E*/*Z*, eluent: 1:1 → 1:1.5 hexanes/CH_2Cl_2) from ethyl 2-(4-bromophenyl)-2-oxoacetate **7a** according to general procedure A2.
^{1}H NMR (400 MHz, $CDCl_3$, linear) δ 7.55 – 7.45 (m, 4H), 6.71 – 6.59/6.28* (m/dt, *J* = 16.7, 10.2 Hz*, 1H), 6.20 – 6.10 (m, 1H), 5.63*/5.50 – 5.41 (dt, *J* = 14.8, 7.4 Hz*/m, 1H), 5.24 (d, *J* = 16.8 Hz, 0.5H), 5.20 – 5.10 (m, 1H), 5.03* (d, *J* = 9.5 Hz, 0.5H), 4.35 – 4.12 (m, 2H), 3.81/3.78* (s, 1H), 3.13/2.94* (dd, *J* = 14.6, 8.4 Hz/14.1, 7.9 Hz*, 1H), 2.83 – 2.69 (m, 1H), 1.27 (t, *J* = 7.2 Hz, 3H).
^{1}H NMR (400 MHz, $CDCl_3$, branched) δ 7.53 (d, *J* = 8.5 Hz, 2H), 7.47 (d, *J* = 8.5 Hz, 2H), 5.92 (dt, *J* = 18.0, 9.0 Hz, 1H), 5.63 (ddd, *J* = 17.5, 10.3, 7.4 Hz, 1H), 5.26 – 5.15 (m, 2H), 5.02 – 4.88 (m, 2H), 4.30 – 4.13 (m, 2H), 3.87 (s, 1H), 3.64 (t, *J* = 7.8 Hz, 1H), 1.28 (t, *J* = 7.1 Hz, 3H).
^{13}C NMR (100 MHz, $CDCl_3$, linear) δ 174.4/174.3*, 140.61/140.58*, 136.7/135.7*, 133.1*/132.1, 131.5, 127.7/127.6*, 127.5*/124.9, 122.20/122.17*, 118.8/116.8*, 77.95*/77.93, 63.0*/62.9, 43.3*/38.2, 14.3*/14.2.
HRMS (ESI) calcd for $C_{15}H_{16}O_2Br$ $[M–OH]^+$: 307.0328, found: 307.0328.

12. Ethyl 2-hydroxy-2-(4-(trifluoromethoxy)phenyl)hepta-4,6-dienoate (8, wrh-08-88)

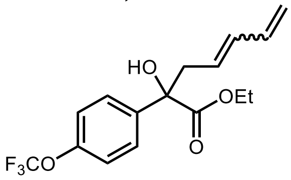

Compound **8** was prepared as a colorless oil (62.6 mg, 63% yield, 2.7:1 l/b, 1.2:1 *E*/*Z*, eluent: 1:1 → 1:1.5 hexanes/CH_2Cl_2) from ethyl 2-oxo-2-(4-(trifluoromethoxy) phenyl)acetate **8a** according to general procedure A2.
^{1}H NMR (400 MHz, $CDCl_3$, linear) δ 7.66 (t, *J* = 8.2 Hz, 2H), 7.20 (d, *J* = 8.1 Hz, 2H), 6.65/6.28* (dt, *J* = 16.8, 10.6 Hz, 1H), 6.21 – 6.11 (m, 1H), 5.65*/5.51 – 5.42 (dt, *J* = 14.9, 7.4 Hz*/m, 1H), 5.24 (d, *J* = 16.7 Hz, 0.5H), 5.19 – 5.09 (m, 1H), 5.03* (d, *J* = 10.0 Hz, 0.5H), 4.34 – 4.14 (m, 2H), 3.85/3.82* (s, 1H), 3.16/2.97* (dd, *J* = 14.5, 8.5 Hz/14.1, 8.0 Hz*, 1H), 2.83 – 2.70 (m, 1H), 1.28 (t, *J* = 7.2 Hz, 3H).
^{1}H NMR (400 MHz, $CDCl_3$, branched) δ 7.69 (d, *J* = 8.8 Hz, 2H), 7.18 (d, *J* = 8.5 Hz, 2H), 5.99 – 5.87 (m, 1H), 5.63 (ddd, *J* = 17.5, 10.4, 7.3 Hz, 1H), 5.25 – 5.17 (m, 2H), 4.95 (dd, *J* = 18.2, 13.9 Hz, 2H), 4.32 – 4.13 (m, 2H), 3.91 (s, 1H), 3.65 (t, *J* = 7.9 Hz, 1H), 1.30 (t, *J* = 7.1 Hz, 3H).

^{13}C NMR (100 MHz, $CDCl_3$, linear) δ 174.4/174.3*, 149.0, 140.2/140.1*, 136.7/135.7*, 133.2*/132.1, 127.44, 127.41/127.38*, 124.9, 120.7, 120.7, 120.6 (q, *J* = 256 Hz), 118.8, 116.8, 77.92*/77.90, 63.0/62.9*, 43.5*/38.4, 14.3*/14.2.
^{19}F NMR (376 MHz, $CDCl_3$, linear) δ –57.83.
HRMS (ESI) calcd for $C_{16}H_{16}O_3F_3$ $[M–OH]^+$: 313.1046, found: 313.1039.

13. Benzyl 2-hydroxy-2-phenylhepta-4,6-dienoate (9, wrh-08-93A)

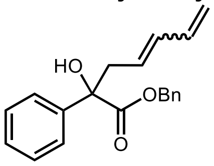

Compound **9** was prepared as a colorless oil (53.3 mg, 58% yield, 6.9:1 l/b, 1:1.4 *E*/*Z*, eluent: CH_2Cl_2) from benzyl 2-oxo-2-phenylacetate **9a** according to general procedure A2.
^{1}H NMR (500 MHz, $CDCl_3$, linear) δ 7.64 – 7.58 (m, 2H), 7.39 – 7.22 (m, 8H), 6.68 – 6.58/6.25* (m/dt, *J* = 16.9, 10.3 Hz*, 1H), 6.17 – 6.06 (m, 1H), 5.63*/5.51 – 5.42 (dt, *J* = 14.8, 7.3 Hz*/m, 1H), 5.28 – 5.19 (m, 1H), 5.17 (d, *J* = 4.5 Hz, 1H), 5.16 – 4.99 (m, 2H), 3.77/3.75*(s, 1H), 3.20/3.02* (dd, *J* = 14.6, 8.2 Hz/14.2, 7.9 Hz*, 1H), 2.87/2.79* (ddd, *J* = 14.6, 6.9, 1.3 Hz/dd, 14.3, 6.7 Hz*, 1H).
^{1}H NMR (500 MHz, $CDCl_3$, branched) δ 7.67 – 7.63 (m, 2H), 7.37 – 7.31 (m, 5H), 7.30 – 7.27 (m, 3H), 5.93 (ddd, *J* = 17.2, 10.3, 8.5 Hz, 1H), 5.65 (ddd, *J* = 17.4, 10.4, 7.0 Hz, 1H), 5.19 – 5.13 (m, 2H), 5.13 – 5.06 (m, 2H), 4.98 – 4.90 (m, 2H), 3.85 (s, 1H), 3.71 (t, *J* = 7.6 Hz, 1H).
^{13}C NMR (125 MHz, $CDCl_3$, linear) δ 174.64/174.57*, 141.34*/141.32, 136.8*/135.5, 135.1*/135.0, 133.0/132.2*, 128.8*/128.71, 128.69*/128.63, 128.44, 128.42*/128.3, 128.1/128.0*, 127.8, 125.74/125.70*, 125.3, 118.6/116.7*, 78.39*/78.38, 68.28/68.26*, 43.1*/38.0.
HRMS (ESI) calcd for $C_{20}H_{19}O_2$ $[M–OH]^+$: 291.1380, found: 291.1370.

14. (1*R*,3*S*,5*r*,7*r*)-Adamantan-2-yl 2-(4-bromophenyl)-2-hydroxyhepta-4,6-dienoate (10, wrh-08-107)

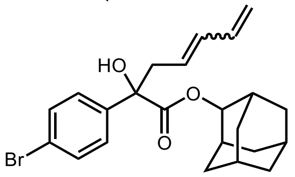

Compound **10** was prepared as a pale yellow oil (83.7 mg, 65% yield, 3.8:1 l/b, 1:1.3 *E*/*Z*, eluent: CH_2Cl_2) from (1*R*,3*S*,5*r*,7*r*)-adamantan-2-yl 2-(4-bromophenyl)-2-oxoacetate **10a** according to general procedure A2.
^{1}H NMR (400 MHz, $CDCl_3$, linear) δ 7.57 – 7.51 (m, 2H), 7.50 – 7.44 (m, 2H), 6.64/6.27* (dt, *J* = 16.9, 10.6 Hz/16.6, 10.1 Hz*, 1H), 6.21 – 6.10 (m, 1H), 5.65*/5.49 – 5.38 (dt, *J* = 14.7, 7.3 Hz*/m, 1H), 5.23 (d, *J* = 16.6 Hz, 0.5H), 5.18 – 5.07 (m, 1H), 5.05 – 4.93 (m, 1.5H), 3.92/3.91* (s, 1H), 3.15/2.99* (dd, *J* = 14.7, 7.8 Hz, 1H), 2.88/2.79* (dd, *J* = 14.7, 7.0 Hz/*J* = 14.2, 6.7 Hz*, 1H), 2.10 – 1.93 (m, 2H), 1.91 – 1.81 (m, 4H), 1.81 – 1.67 (m, 6H), 1.61 (t, *J* = 11.7 Hz, 1H), 1.51 (t, *J* = 11.5 Hz, 1H).
^{1}H NMR (400 MHz, $CDCl_3$, branched) δ 7.57 (d, *J* = 8.7 Hz, 2H), 7.47 (d, *J* = 8.7 Hz, 2H), 6.00 – 5.87 (m, 1H), 5.65 (ddd, *J* = 17.4, 10.5, 7.3 Hz, 1H), 5.24 – 5.15 (m, 2H), 5.03 – 4.93 (m, 3H), 3.99 (s, 1H), 3.70 (t, *J* = 7.7 Hz, 1H), 2.13 – 1.99 (m, 2H), 1.92 – 1.81 (m, 4H), 1.80 – 1.70 (m, 6H), 1.70 – 1.62 (m, 1H), 1.54 – 1.45 (m, 1H).

^{13}C NMR (100 MHz, $CDCl_3$, linear) δ 173.94/173.88*, 140.8, 136.8*/135.6, 133.0/132.1*, 131.42/131.41*, 127.81/127.77*, 127.5*/124.9, 122.12/122.08*, 118.7/116.7*, 80.4/80.3*, 77.8*/77.7, 43.2, 38.1, 37.3/36.4*, 36.29*/36.27, 32.0, 31.98*/31.94, 31.89*/31.84, 31.77*/31.71, 27.19*/27.16, 26.9.
HRMS (ESI) calcd for $C_{23}H_{28}O_3Br$ $[M+H]^+$: 431.1216, found: 431.1213.

15. (*E*)-1-Benzyl-3-hydroxy-3-(penta-2,4-dien-1-yl)indolin-2-one (11, wrh-08-120C)

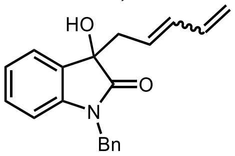

Compound **11** was prepared as a pale yellow solid (27.2 mg, 44% yield, 1.7:1 l/b, 1:1.1 *E*/*Z*, eluent: 30:1 → 20:1 CH_2Cl_2/EtOAc) from 1-benzylisatin **11a** according to general procedure A2.
^{1}H NMR (500 MHz, $CDCl_3$, linear) δ 7.46 – 7.38 (m, 1H), 7.34 – 7.23 (m, 5H), 7.21 (td, *J* = 7.8, 1.3 Hz, 1H), 7.10 – 7.03 (m, 1H), 6.70 (dd, *J* = 15.1, 7.8 Hz, 1H), 6.66 – 6.57/6.25 – 6.18* (m, 1H), 6.18 – 6.05 (m, 1H), 5.43 (ddd, *J* = 14.8, 9.0, 6.1 Hz, 0.5H), 5.28 – 5.18 (m, 1H), 5.17 – 5.09 (m, 1.5H), 5.08 – 5.01 (m, 1H), 4.70/4.63* (d, *J* = 15.6 Hz/15.8 Hz*, 1H), 3.21 (d, *J* = 10.9 Hz, 1H), 3.04 – 2.96 (m, 0.5H), 2.92 – 2.83 (m, 1H), 2.80 – 2.72 (m, 0.5H).
^{1}H NMR (500 MHz, $CDCl_3$, branched) δ 7.38 (dd, *J* = 7.4, 1.3 Hz, 1H), 7.34 – 7.24 (m, 5H), 7.21 (td, *J* = 7.8, 1.3 Hz, 1H), 7.05 (td, *J* = 7.5, 1.0 Hz, 1H), 6.68 (d, *J* = 7.9 Hz, 1H), 5.95 (ddd, *J* = 17.2, 10.4, 6.7 Hz, 1H), 5.68 – 5.57 (m, 1H), 5.29 (dd, *J* = 17.1, 1.7 Hz, 1H), 5.22 – 5.16 (m, 2H), 5.14 (dt, *J* = 17.3, 1.5 Hz, 1H), 5.04 (d, *J* = 15.7 Hz, 1H), 4.66 (d, *J* = 15.7 Hz, 1H), 3.47 – 3.40 (m, 1H), 3.09 (s, 1H).
^{13}C NMR (125 MHz, $CDCl_3$, linear) δ 177.9, 142.6, 136.6/136.4*, 135.6/135.5*, 133.9/131.7*, 129.83/129.82*, 129.68/129.65*, 129.0*/128.9, 127.82/127.76*, 127.5/127.3*, 125.9, 124.23/124.20*, 123.3/123.2*, 119.1/117.2*, 109.7*/109.6, 76.3*/76.1, 44.03/44.00*, 42.1*/37.0.
HRMS (ESI) calcd for $C_{20}H_{20}O_2N$ $[M+H]^+$: 306.1489, found: 306.1484.
m.p. (linear) = 115 – 116 °C; **m.p.** (branched) = 106 – 107 °C

16. Ethyl 3-(penta-1,4-dien-3-yl)-2,3-dihydrobenzo[*d*]isothiazole-3-carboxylate 1, 1-dioxide (12, wrh-07-165A)

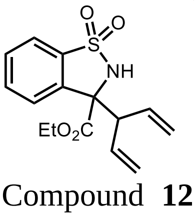

Compound **12** was prepared as a white solid (84.6 mg, 92% yield, eluent: 2:1 hexanes/EtOAc) from ethyl benzo[*d*]isothiazole-3-carboxylate 1,1-dioxide **12a** according to general procedure A3.
^{1}H NMR (400 MHz, $CDCl_3$) δ 7.79 – 7.70 (m, 2H), 7.67 (t, *J* = 7.5 Hz, 1H), 7.61 – 7.53 (m, 1H), 5.89 – 5.78 (m, 1H), 5.72 (s, 1H), 5.66 (ddd, *J* = 17.2, 10.2, 8.4 Hz, 1H), 5.26 – 5.17 (m, 2H), 4.98 – 4.84 (m, 2H), 4.36 – 4.22 (m, 2H), 3.66 (t, *J* = 8.4 Hz, 1H), 1.34 (t, *J* = 7.1 Hz, 3H).
^{13}C NMR (100 MHz, $CDCl_3$) δ 169.6, 136.0, 135.7, 134.9, 133.6, 133.2, 130.6, 125.6, 121.7, 119.7, 119.2, 72.3, 63.8, 57.0, 14.2.
HRMS (ESI) calcd for $C_{15}H_{18}O_4NS$ $[M+H]^+$: 308.0951, found: 308.0952.

m.p. = 113 – 114 ℃

17. Ethyl 5-methyl-3-(penta-1,4-dien-3-yl)-2,3-dihydrobenzo[*d*]isothiazole-3-carboxylate 1,1-dioxide (13, wrh-07-164)

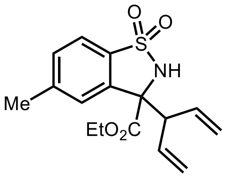

Compound **13** was prepared as a white solid (87.9 mg, 91% yield, eluent: 3:1 hexanes/EtOAc) from ethyl 5-methylbenzo[*d*]isothiazole-3-carboxylate 1,1-dioxide **13a** according to general procedure A3.
^{1}H NMR (500 MHz, $CDCl_3$) δ 7.60 (d, J = 8.0 Hz, 1H), 7.51 (s, 1H), 7.40 – 7.33 (m, 1H), 5.87 – 5.77 (m, 1H), 5.71 – 5.60 (m, 2H), 5.26 – 5.17 (m, 2H), 4.98 – 4.87 (m, 2H), 4.34 – 4.23 (m, 2H), 3.64 (t, J = 8.3 Hz, 1H), 2.48 (s, 3H), 1.33 (t, J = 7.1 Hz, 3H).
^{13}C NMR (125 MHz, $CDCl_3$) δ 169.6, 144.7, 136.3, 134.9, 133.3, 133.1, 131.6, 125.6, 121.3, 119.6, 119.2, 72.1, 63.7, 56.9, 22.1, 14.2.
HRMS (ESI) calcd for $C_{16}H_{20}O_4NS$ $[M+H]^+$: 322.1108, found: 322.1099.
m.p. = 128 – 129 ℃

18. Ethyl 5-fluoro-3-(penta-1,4-dien-3-yl)-2,3-dihydrobenzo[*d*]isothiazole-3-carboxylate 1,1-dioxide (14, wrh-07-169C)

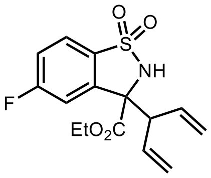

Compound **14** was prepared as a white solid (77.5 mg, 79% yield, eluent: 3:1 hexanes/EtOAc) from ethyl 5-fluorobenzo[*d*]isothiazole-3-carboxylate 1,1-dioxide **14a** according to general procedure A3.
^{1}H NMR (500 MHz, $CDCl_3$) δ 7.73 (dd, *J* = 8.5, 4.7 Hz, 1H), 7.41 (dd, *J* = 8.5, 1.9 Hz, 1H), 7.31 – 7.23 (m, 1H), 5.87 – 5.73 (m, 2H), 5.73 – 5.63 (m, 1H), 5.27 – 5.18 (m, 2H), 5.03 – 4.90 (m, 2H), 4.38 – 4.25 (m, 2H), 3.59 (t, *J* = 8.4 Hz, 1H), 1.36 (t, *J* = 7.1 Hz, 3H).
^{13}C NMR (125 MHz, $CDCl_3$) δ 169.0, 165.8 (d, *J* = 255 Hz), 139.2 (d, *J* = 9.6 Hz), 134.5, 132.9, 131.8 (d, *J* = 2.6 Hz), 123.9 (d, *J* = 10.0 Hz), 120.0, 119.5, 118.8 (d, *J* = 24.2 Hz), 112.7 (d, *J* = 25.0 Hz), 71.8 (d, *J* = 2.1 Hz), 64.1, 57.2, 14.2.
^{19}F NMR (471 MHz, $CDCl_3$) δ –103.3.
HRMS (ESI) calcd for $C_{15}H_{17}O_4NFS$ $[M+H]^+$: 326.0857, found: 326.0845.
m.p. = 103 – 104 ℃

19. Ethyl 5-chloro-3-(penta-1,4-dien-3-yl)-2,3-dihydrobenzo[*d*]isothiazole-3-carboxylate 1,1-dioxide (15, wrh-07-165B)

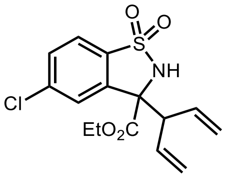

Compound **15** was prepared as a white solid on the scale of 0.2 mmol (58.8 mg, 86% yield, eluent: 4:1 hexanes/EtOAc) from ethyl 5-chlorobenzo[*d*]isothiazole-3-carboxylate 1,1-dioxide **15a** according to general procedure A3.
^{1}H NMR (400 MHz, $CDCl_3$) δ 7.72 (d, *J* = 1.3 Hz, 1H), 7.66 (d, *J* = 8.3 Hz, 1H), 7.54 (dd, *J* = 8.3, 1.5 Hz, 1H), 5.87 – 5.73 (m, 2H), 5.66 (ddd, *J* = 17.3, 10.2, 8.5 Hz, 1H), 5.28 – 5.17 (m, 2H), 4.96 (dd, *J* = 17.5, 13.8 Hz, 2H), 4.38 – 4.25 (m, 2H), 3.60 (t, *J* = 8.4 Hz, 1H), 1.36 (t, *J* = 7.1 Hz, 3H).
^{13}C NMR (100 MHz, $CDCl_3$) δ 169.0, 140.1, 138.0, 134.5, 134.3, 132.9, 131.2, 125.8, 122.9, 120.1, 119.5, 71.9, 64.1, 57.1, 14.2.
HRMS (ESI) calcd for $C_{15}H_{17}O_4NClS$ $[M+H]^+$: 342.0561, found: 342.0564.
m.p. = 128 – 129 °C

20. Ethyl 3-(penta-1,4-dien-3-yl)-5-(trifluoromethyl)-2,3-dihydrobenzo[*d*]isothiazole-3-carboxylate 1,1-dioxide (16, wrh-07-169B)

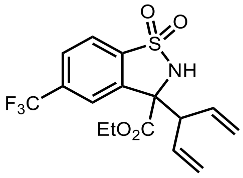

Compound **16** was prepared as a white solid (102 mg, 90% yield, eluent: 5:1 hexanes/EtOAc) from ethyl 5-(trifluoromethyl)benzo[*d*]isothiazole-3-carboxylate 1,1-dioxide **16a** according to general procedure A3.
^{1}H NMR (400 MHz, $CDCl_3$) δ 8.01 (s, 1H), 7.90 – 7.81 (m, 2H), 5.90 – 5.77 (m, 2H), 5.66 (ddd, *J* = 17.2, 10.2, 8.6 Hz, 1H), 5.29 – 5.21 (m, 2H), 5.01 – 4.85 (m, 2H), 4.41 – 4.25 (m, 2H), 3.66 (t, *J* = 8.5 Hz, 1H), 1.35 (t, *J* = 7.1 Hz, 3H).
^{13}C NMR (100 MHz, $CDCl_3$) δ 168.8, 138.9, 137.1, 135.7 (q, *J* = 33.2 Hz), 134.3, 132.8, 127.9 (q, *J* = 3.3 Hz), 124.5, 123.2 (q, *J* = 4.0 Hz), 122.7, 121.8, 120.3, 119.7, 72.2, 64.3, 57.2, 14.2.
^{19}F NMR (376 MHz, $CDCl_3$) δ –62.88.
HRMS (ESI) calcd for $C_{16}H_{17}O_4NF_3S$ $[M+H]^+$: 376.0825, found: 376.0810.
m.p. = 152 – 153 °C

21. Ethyl 5-methoxy-3-(penta-1,4-dien-3-yl)-2,3-dihydrobenzo[*d*]isothiazole-3-carboxylate 1,1-dioxide (17, wrh-07-174A)

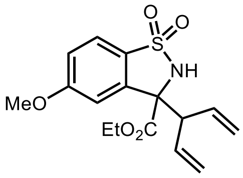

Compound **17** was prepared as a white solid (81.4 mg, 80% yield, eluent: 2.5:1 hexanes/EtOAc) from ethyl 5-methoxybenzo[*d*]isothiazole-3-carboxylate 1,1-dioxide **17a** according to general procedure A3.
^{1}H NMR (500 MHz, $CDCl_3$) δ 7.63 (d, *J* = 8.6 Hz, 1H), 7.15 (d, *J* = 2.2 Hz, 1H), 7.06 (dd, *J* = 8.6, 2.2 Hz, 1H), 5.87 – 5.77 (m, 1H), 5.73 – 5.63 (m, 2H), 5.26 – 5.17 (m, 2H), 4.95 (dd, *J* = 18.2, 13.7 Hz, 2H), 4.35 – 4.24 (m, 2H), 3.90 (s, 3H), 3.61 (t, *J* = 8.3 Hz, 1H), 1.34 (t, *J* = 7.1 Hz, 3H).
^{13}C NMR (125 MHz, $CDCl_3$) δ 169.5, 164.0, 138.6, 134.9, 133.2, 127.9, 123.1, 119.7, 119.2, 117.1, 109.8, 71.9, 63.8, 57.1, 56.1, 14.3.
HRMS (ESI) calcd for $C_{16}H_{18}O_5NS$ $[M–H]^–$: 336.0911, found: 336.0906.
m.p. = 161 – 163 °C

22. Ethyl 5-(*tert*-butyl)-3-(penta-1,4-dien-3-yl)-2,3-dihydrobenzo[*d*]isothiazole-3-carboxylate 1,1-dioxide (18, wrh-07-174B)

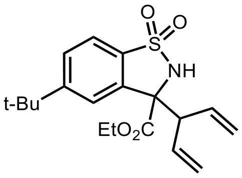

Compound **18** was prepared as a colorless oil (100 mg, 92% yield, eluent: 4:1 hexanes/EtOAc) from ethyl 5-(*tert*-butyl)benzo[*d*]isothiazole-3-carboxylate 1,1-dioxide **18a** according to general procedure A3.

^{1}H NMR (500 MHz, $CDCl_3$) δ 7.72 (d, *J* = 1.2 Hz, 1H), 7.65 (d, *J* = 8.2 Hz, 1H), 7.59 (dd, *J* = 8.3, 1.5 Hz, 1H), 5.84 (ddd, *J* = 17.2, 10.2, 8.6 Hz, 1H), 5.73 – 5.61 (m, 2H), 5.27 – 5.17 (m, 2H), 4.93 (d, *J* = 10.3 Hz, 1H), 4.87 (d, *J* = 17.1 Hz, 1H), 4.33 (dq, *J* = 10.8, 7.1 Hz, 1H), 4.24 (dq, *J* = 10.8, 7.1 Hz, 1H), 3.66 (t, *J* = 8.4 Hz, 1H), 1.40 – 1.30 (m, 12H).

^{13}C NMR (125 MHz, $CDCl_3$) δ 169.7, 157.9, 136.0, 135.0, 133.4, 132.9, 128.0, 122.4, 121.1, 119.5, 119.1, 72.3, 63.6, 57.0, 35.6, 31.3, 14.3.

HRMS (ESI) calcd for $C_{19}H_{24}O_4NS$ $[M–H]^-$: 362.1432, found: 362.1425.

23. Ethyl 7-chloro-3-(penta-1,4-dien-3-yl)-2,3-dihydrobenzo[*d*]isothiazole-3-carboxylate 1,1-dioxide (19, wrh-07-169A)

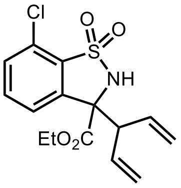

Compound **19** was prepared as a white solid (89.7 mg, 87% yield, eluent: 3:1 hexanes/EtOAc) from ethyl 7-chlorobenzo[*d*]isothiazole-3-carboxylate 1,1-dioxide **19a** according to general procedure A3.

^{1}H NMR (400 MHz, $CDCl_3$) δ 7.65 (d, *J* = 7.8 Hz, 1H), 7.59 (t, *J* = 7.8 Hz, 1H), 7.49 (d, *J* = 7.7 Hz, 1H), 5.88 – 5.75 (m, 2H), 5.75 – 5.61 (m, 1H), 5.27 – 5.17 (m, 2H), 5.01 – 4.85 (m, 2H), 4.29 (q, *J* = 7.1 Hz, 2H), 3.63 (t, *J* = 8.4 Hz, 1H), 1.34 (t, *J* = 7.1 Hz, 3H).

^{13}C NMR (100 MHz, $CDCl_3$) δ 169.1, 138.7, 134.7, 134.6, 133.7, 133.0, 131.4, 129.3, 123.9, 120.0, 119.4, 71.3, 64.1, 57.1, 14.2.

HRMS (ESI) calcd for $C_{15}H_{17}O_4NClS$ $[M+H]^+$: 342.0561, found: 342.0549.

m.p. = 161 – 162 °C

24. Ethyl 6-methyl-3-(penta-1,4-dien-3-yl)-2,3-dihydrobenzo[*d*]isothiazole-3-carboxylate 1,1-dioxide (20, wrh-07-173A)

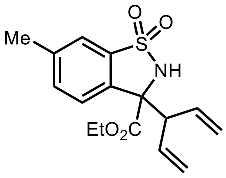

Compound **20** was prepared as a white solid (81.2 mg, 84% yield, eluent: 3:1 hexanes/EtOAc) from ethyl 6-methylbenzo[*d*]isothiazole-3-carboxylate 1,1-dioxide **20a** according to general procedure A3.

^{1}H NMR (500 MHz, $CDCl_3$) δ 7.60 (d, *J* = 8.1 Hz, 1H), 7.52 (s, 1H), 7.46 (d, *J* = 8.1 1H), 5.88 – 5.76 (m, 1H), 5.74 – 5.60 (m, 2H), 5.27 – 5.15 (m, 2H), 4.99 – 4.85 (m, 2H), 4.34 – 4.21 (m, 2H), 3.63 (t, *J* = 8.3 Hz, 1H), 2.44 (s, 3H), 1.33 (t, *J* = 7.1 Hz, 3H).
^{13}C NMR (125 MHz, $CDCl_3$) δ 169.7, 141.4, 135.7, 135.0, 134.8, 133.3, 133.2, 125.3, 121.5, 119.6, 119.1, 72.1, 63.7, 56.9, 21.3, 14.2.
HRMS (ESI) calcd for $C_{16}H_{20}O_4NS$ $[M+H]^+$: 322.1108, found: 322.1102.
m.p. = 115 – 116 ℃

25. Ethyl 3-(penta-1,4-dien-3-yl)-2,3-dihydronaphtho[2,1-*d*]isothiazole-3-carboxylate 1,1-dioxide (21, wrh-07-173B)

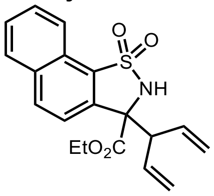

Compound **21** was prepared as a white solid (83.2 mg, 77% yield, eluent: 3:1 hexanes/EtOAc) from ethyl naphtho[2,1-*d*]isothiazole-3-carboxylate 1,1-dioxide **21a** according to general procedure A3.
^{1}H NMR (500 MHz, $CDCl_3$) δ 8.37 (d, *J* = 8.3 Hz, 1H), 8.10 (d, *J* = 8.6 Hz, 1H), 7.95 (d, *J* = 8.1 Hz, 1H), 7.75 (d, *J* = 8.7 Hz, 1H), 7.73 – 7.68 (m, 1H), 7.68 – 7.62 (m, 1H), 5.94 – 5.84 (m, 2H), 5.70 (ddd, *J* = 17.1, 10.2, 8.3 Hz, 1H), 5.30 – 5.22 (m, 2H), 4.97 – 4.86 (m, 2H), 4.37 – 4.25 (m, 2H), 3.78 (t, *J* = 8.4 Hz, 1H), 1.35 (t, *J* = 7.1 Hz, 3H).
^{13}C NMR (125 MHz, $CDCl_3$) δ 169.5, 135.1, 135.0, 134.6, 134.0, 133.2, 131.2, 129.4, 128.6, 128.4, 125.4, 123.4, 121.0, 119.7, 119.2, 72.3, 63.9, 56.5, 14.3.
HRMS (ESI) calcd for $C_{19}H_{20}O_4NS$ $[M+H]^+$: 358.1108, found: 358.1110.
m.p. = 132 – 133 ℃

26. 4-(Penta-1,4-dien-3-yl)-3,4-dihydrobenzo[*e*][1,2,3]oxathiazine 2,2-dioxide (22, wrh-07-173C)

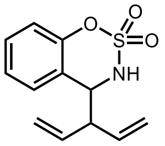

Compound **22** was prepared as a colorless oil (56.6 mg, 75% yield, 20:1 b/l, eluent: 10:1 → 8:1 hexanes/EtOAc) from benzo[*e*][1,2,3]oxathiazine 2,2-dioxide **22a** according to general procedure A3.
^{1}H NMR (500 MHz, $CDCl_3$) δ 7.37 (d, *J* = 7.8 Hz, 1H), 7.35 – 7.30 (m, 1H), 7.21 (td, *J* = 7.6, 1.2 Hz, 1H), 7.03 (dd, *J* = 8.2, 1.1 Hz, 1H), 5.96 (ddd, *J* = 17.3, 10.4, 7.7 Hz, 1H), 5.75 (ddd, *J* = 17.3, 10.6, 5.3 Hz, 1H), 5.40 – 5.29 (m, 3H), 5.27 – 5.21 (m, 1H), 4.97 (dd, *J* = 9.1, 3.6 Hz, 1H), 4.56 (d, *J* = 9.1 Hz, 1H), 3.76 – 3.68 (m, 1H).
^{13}C NMR (125 MHz, $CDCl_3$) δ 152.0, 134.3, 133.7, 129.8, 126.4, 125.5, 120.93, 120.89, 119.9, 119.3, 59.5, 49.2.
HRMS (ESI) calcd for $C_{12}H_{12}O_3NS$ $[M–H]^–$: 250.0543, found: 250.0539.

27. (*E*)-*N*-(1-(4-Bromophenyl)hexa-3,5-dien-1-yl)-*N*,2,4,6-tetramethylbenzenesulfonamide (23, wrh-08-118A)

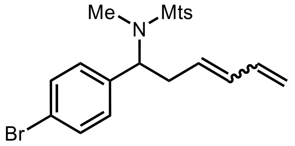

Compound **23** was prepared as a colorless oil (49.9 mg, 56% yield, >20:1 *E*/*Z*, eluent: 30:1 hexanes/EtOAc) from *N*-((4-bromophenyl)(methoxy)methyl)-*N*,2,4,6-tetramethylbenzenesulfonamide **23a** according to general procedure A4.

^{1}H NMR (500 MHz, $CDCl_3$) δ 7.45 – 7.41 (m, 2H), 7.07 (d, *J* = 8.4 Hz, 2H), 6.96 (s, 2H), 6.14 – 6.00 (m, 2H), 5.31 (dt, *J* = 14.2, 7.1 Hz, 1H), 5.10 – 5.05 (m, 1H), 5.00 – 4.95 (m, 1H), 4.80 (dd, *J* = 9.2, 6.5 Hz, 1H), 2.84 – 2.76 (m, 1H), 2.73 – 2.66 (m, 1H), 2.62 (s, 3H), 2.58 (s, 6H), 2.32 (s, 3H).

^{13}C NMR (126 MHz, $CDCl_3$) δ 142.7, 140.4, 136.7, 136.6, 133.9, 133.3, 132.2, 131.7, 130.3, 129.7, 122.1, 116.4, 58.5, 33.7, 28.3, 23.2, 21.1.

HRMS (ESI) calcd for $C_{22}H_{26}O_2NBrNaS$ $[M+Na]^+$: 470.0760, found: 470.0771.

28. (*E*)-*N*-(1-(2,2-Difluorobenzo[*d*][1,3]dioxol-5-yl)hexa-3,5-dien-1-yl)-*N*,2,4,6-tetramethylbenzenesulfonamide (24, wrh-08-117C)

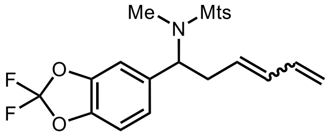

Compound **24** was prepared as a colorless oil (40.3 mg, 45% yield, >20:1 *E*/*Z*, eluent: 30:1 hexanes/EtOAc) from *N*-((2,2-difluorobenzo[*d*][1,3]dioxol-5-yl)(methoxy) methyl)-*N*,2,4,6-tetramethylbenzenesulfonamide **24a** according to general procedure A4.

^{1}H NMR (400 MHz, $CDCl_3$) δ 7.00 – 6.89 (m, 5H), 6.16 – 5.99 (m, 2H), 5.30 (dt, *J* = 14.1, 7.0 Hz, 1H), 5.09 (d, *J* = 16.7 Hz, 1H), 4.99 (d, *J* = 9.9 Hz, 1H), 4.84 (dd, *J* = 8.7, 6.9 Hz, 1H), 2.82 – 2.67 (m, 2H), 2.63 (s, 3H), 2.58 (s, 6H), 2.32 (s, 3H).

^{13}C NMR (100 MHz, $CDCl_3$) δ 144.0, 143.4, 142.8, 140.4, 136.6, 134.1, 133.9, 133.20, 132.25, 129.4, 123.8, 116.6, 109.9, 109.2, 58.7, 34.0, 28.3, 23.2, 21.1.

^{19}F NMR (376 MHz, $CDCl_3$) δ –49.40, –49.66, –49.78, –50.03.

HRMS (ESI) calcd for $C_{23}H_{25}O_4NF_2NaS$ $[M+Na]^+$: 472.1365, found: 472.1375.

29. Ethyl 7-chloro-3-(1-phenylallyl)-2,3-dihydrobenzo[*d*]isothiazole-3-carboxylate 1,1-dioxide (25, wrh-07-185A)

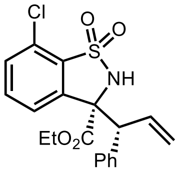

Compound **25** was prepared as a white solid (61.6 mg, 79% yield, >20:1 d.r., eluent: 2.5:1 hexanes/EtOAc) from allylbenzene **25b** according to general procedure A5.

^{1}H NMR (400 MHz, $CDCl_3$) δ 7.80 (d, *J* = 7.8 Hz, 1H), 7.64 (t, *J* = 7.9 Hz, 1H), 7.53 (d, *J* = 7.8 Hz, 1H), 7.44 – 7.37 (m, 2H), 7.37 – 7.27 (m, 3H), 6.09 (dt, *J* = 17.0, 9.8 Hz, 1H), 5.76 (s, 1H), 4.97 (d, *J* = 10.1 Hz, 1H), 4.86 (d, *J* = 16.9 Hz, 1H), 4.18 (d, *J* = 9.3 Hz, 1H), 4.15 – 4.00 (m, 2H), 1.18 (t, *J* = 7.1 Hz, 3H).

^{13}C NMR (100 MHz, $CDCl_3$) δ 168.7, 139.0, 138.0, 134.7, 134.0, 133.8, 131.4, 129.3, 128.9, 128.8, 128.2, 124.0, 120.0, 71.9, 63.9, 58.4, 14.0.

HRMS (ESI) calcd for $C_{19}H_{19}O_4NClS$ $[M+H]^+$: 392.0718, found: 392.0722.

m.p. = 180 – 181 ℃

30. Ethyl 7-chloro-3-(1-(4-fluorophenyl)allyl)-2,3-dihydrobenzo[*d*]isothiazole-3-carboxylate 1,1-dioxide (26, wrh-07-187B)

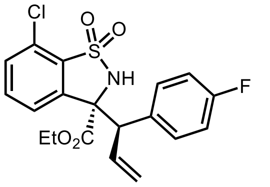

Compound **26** was prepared as a white solid (53.6 mg, 65% yield, >20:1 d.r., eluent: 2.5:1 hexanes/EtOAc) from 1-allyl-4-fluorobenzene **26b** according to general procedure A5.

^{1}H NMR (400 MHz, $CDCl_3$) δ 7.78 (d, *J* = 7.8 Hz, 1H), 7.64 (t, *J* = 7.9 Hz, 1H), 7.53 (d, *J* = 7.8 Hz, 1H), 7.43 – 7.34 (m, 2H), 7.03 (t, *J* = 8.6 Hz, 2H), 6.04 (dt, *J* = 16.9, 9.7 Hz, 1H), 5.75 (s, 1H), 4.97 (d, *J* = 10.2 Hz, 1H), 4.85 (d, *J* = 16.9 Hz, 1H), 4.23 – 4.03 (m, 3H), 1.20 (t, *J* = 7.1 Hz, 3H).

^{13}C NMR (100 MHz, $CDCl_3$) δ 168.7, 162.6 (d, *J* = 247 Hz), 139.0, 134.8, 133.9, 133.8, 131.5, 130.6, 130.5, 129.4, 123.9, 120.1, 115.7 (d, *J* = 21.3 Hz), 71.9, 64.0, 57.6, 14.1.

^{19}F NMR (376 MHz, $CDCl_3$) δ –113.9.

HRMS (ESI) calcd for $C_{19}H_{18}O_4NClFS$ $[M+H]^+$: 410.0624, found: 410.0627.

m.p. = 175 – 177 °C

31. Ethyl 7-chloro-3-(1-(4-(((trifluoromethyl)sulfonyl)oxy)phenyl)allyl)-2,3-dihydrobenzo[*d*]isothiazole-3-carboxylate 1,1-dioxide (27, wrh-08-2A)

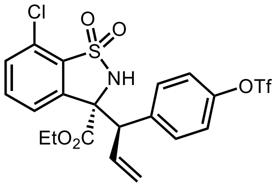

Compound **27** was prepared as a white solid (91.9 mg, 85% yield, >20:1 d.r., eluent: 2:1 hexanes/EtOAc) from 4-allylphenyl trifluoromethanesulfonate **27b** according to general procedure A5.

^{1}H NMR (500 MHz, $CDCl_3$) δ 7.78 (d, *J* = 7.9 Hz, 1H), 7.65 (t, *J* = 7.9 Hz, 1H), 7.58 – 7.48 (m, 3H), 7.30 – 7.22 (m, 2H), 6.04 (dt, *J* = 16.9, 9.7 Hz, 1H), 5.75 (s, 1H), 5.01 (d, *J* = 10.2 Hz, 1H), 4.87 (d, *J* = 16.9 Hz, 1H), 4.22 (d, *J* = 9.2 Hz, 1H), 4.18 – 4.03 (m, 2H), 1.16 (t, *J* = 7.1 Hz, 3H).

^{13}C NMR (125 MHz, $CDCl_3$) δ 168.4, 149.3, 138.8, 138.7, 134.9, 133.9, 133.2, 131.7, 130.9, 129.5, 123.8, 121.7, 120.9, 118.9 (q, *J* = 319 Hz), 71.6, 64.2, 57.8, 14.0.

^{19}F NMR (471 MHz, $CDCl_3$) δ –72.81.

HRMS (ESI) calcd for $C_{20}H_{16}O_7NClF_3S_2$ $[M–H]^–$: 538.0014, found: 537.9994.

m.p. = 131 – 133°C

32. Ethyl 7-chloro-3-(1-(4-cyanophenyl)allyl)-2,3-dihydrobenzo[*d*]isothiazole-3-carboxylate 1,1-dioxide (28, wrh-08-7A)

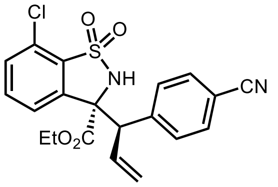

Compound **28** was prepared as a white solid (78.6 mg, 94% yield (including both regioisomers), 17:1 d.r., eluent: 1.5:1 hexanes/EtOAc) from 4-allylbenzonitrile **28b** according to general procedure A5.

^{1}H NMR (500 MHz, $CDCl_3$) δ 7.76 (d, *J* = 7.9 Hz, 1H), 7.69 – 7.61 (m, 3H), 7.59 – 7.50 (m, 3H), 6.04 (dt, *J* = 16.9, 9.7 Hz, 1H), 5.78 (s, 1H), 5.02 (d, *J* = 10.2 Hz, 1H), 4.88 (d, *J* = 16.9 Hz, 1H), 4.23 (d, *J* = 9.3 Hz, 1H), 4.20 – 4.06 (m, 2H), 1.20 (t, *J* = 7.1 Hz, 3H).
^{13}C NMR (125 MHz, $CDCl_3$) δ 168.4, 143.5, 138.6, 135.0, 133.9, 132.9, 132.5, 131.8, 129.8, 129.6, 123.7, 121.2, 118.5, 112.2, 71.4, 64.3, 58.3, 14.1.
HRMS (ESI) calcd for $C_{20}H_{18}O_4N_2ClS$ $[M+H]^+$: 417.0670, found: 417.0686.
m.p. = 189 – 190 °C

33. Ethyl 7-chloro-3-(1-(m-tolyl)allyl)-2,3-dihydrobenzo[*d*]isothiazole-3-carboxylate 1,1-dioxide (29, wrh-07-188)

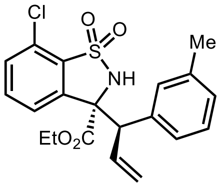

Compound **29** was prepared as a white solid (63.3 mg, 78% yield, >20:1 d.r., eluent: 2.5:1 hexanes/EtOAc) from 1-allyl-3-methylbenzene **29b** according to general procedure A5.
^{1}H NMR (500 MHz, $CDCl_3$) δ 7.79 (d, *J* = 7.9 Hz, 1H), 7.63 (t, *J* = 7.9 Hz, 1H), 7.52 (d, *J* = 7.8 Hz, 1H), 7.24 – 7.16 (m, 3H), 7.09 (d, *J* = 7.1 Hz, 1H), 6.09 (dt, *J* = 16.9, 9.8 Hz, 1H), 5.76 (s, 1H), 4.96 (dd, *J* = 10.2, 0.9 Hz, 1H), 4.86 (d, *J* = 16.9 Hz, 1H), 4.18 – 4.03 (m, 3H), 2.35 (s, 3H), 1.19 (t, *J* = 7.2 Hz, 3H).
^{13}C NMR (100 MHz, $CDCl_3$) δ 168.8, 139.1, 138.4, 137.9, 134.7, 134.1, 133.7, 131.4, 129.5, 129.3, 128.9, 128.7, 125.9, 124.0, 119.9, 71.9, 63.8, 58.3, 21.6, 14.0.
HRMS (ESI) calcd for $C_{20}H_{21}O_4NClS$ $[M+H]^+$: 406.0874, found: 406.0876.
m.p. = 179 – 181 °C

34. Ethyl 7-chloro-3-(1-(naphthalen-2-yl)allyl)-2,3-dihydrobenzo[*d*]isothiazole-3-carboxylate 1,1-dioxide (30, wrh-08-9)

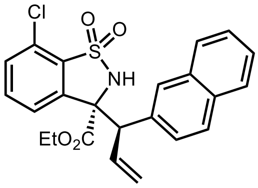

Compound **30** was prepared as a white solid (59.3 mg, 67% yield (major diastereomer), 15:1 d.r., eluent: 2.5:1 hexanes/EtOAc → 100% CH_2Cl_2) from 2-allylnaphthalene **30b** according to general procedure A5.
^{1}H NMR (400 MHz, $CDCl_3$) δ 7.91 – 7.78 (m, 5H), 7.66 (t, *J* = 7.9 Hz, 1H), 7.59 – 7.45 (m, 4H), 6.20 (dt, *J* = 17.0, 9.8 Hz, 1H), 5.84 (s, 1H), 5.01 (d, *J* = 10.2 Hz, 1H), 4.92 (d, *J* = 16.9 Hz, 1H), 4.37 (d, *J* = 9.1 Hz, 1H), 4.14 – 3.99 (m, 2H), 1.14 (t, *J* = 7.1 Hz, 3H).
^{13}C NMR (100 MHz, $CDCl_3$) δ 168.7, 139.1, 135.5, 134.8, 134.0, 133.8, 133.4, 133.0, 131.5, 129.4, 128.5, 128.03, 127.98, 127.8, 126.6, 126.5, 126.4, 124.0, 120.2, 72.1, 64.0, 58.4, 14.0.
HRMS (ESI) calcd for $C_{23}H_{21}O_4NClS$ $[M+H]^+$: 442.0874, found: 442.0860.
m.p. = 188 – 189 °C

35. Ethyl 7-chloro-3-(1-(4-((4-(4,4,5,5-tetraethyl-1,3,2-dioxaborolan-2-yl)phenoxy)carbonyl)phenyl)allyl)-2,3-dihydrobenzo[*d*]isothiazole-3-carboxylate 1,1-dioxide (31, wrh-08-100)

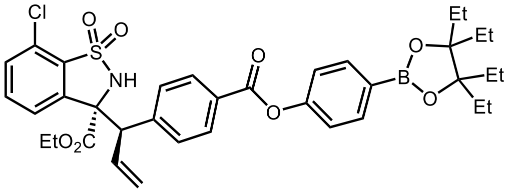

Compound **31** was prepared as a pale yellow oil (105 mg, 75% yield (including both regio-isomers), >20:1 d.r., eluent: 3:1 hexanes/EtOAc) from 4-(4,4,5,5-tetraethyl-1,3,2-dioxaborolan-2-yl)phenyl 4-allylbenzoate **31b** according to general procedure A5.

^{1}H NMR (500 MHz, $CDCl_3$) δ 8.17 (d, *J* = 8.4 Hz, 2H), 7.90 (d, *J* = 8.4 Hz, 2H), 7.80 (d, *J* = 7.8 Hz, 1H), 7.66 (t, *J* = 7.9 Hz, 1H), 7.60 – 7.53 (m, 3H), 7.22 (d, *J* = 8.5 Hz, 2H), 6.11 (dt, *J* = 16.9, 9.9 Hz, 1H), 5.82 (s, 1H), 5.02 (dd, *J* = 10.2, 1.0 Hz, 1H), 4.90 (d, *J* = 16.9 Hz, 1H), 4.28 (d, *J* = 9.2 Hz, 1H), 4.21 – 4.07 (m, 2H), 1.86 – 1.69 (m, 8H), 1.24 (t, *J* = 7.1 Hz, 3H), 0.98 (t, *J* = 7.5 Hz, 12H).

^{13}C NMR (125 MHz, $CDCl_3$) δ 168.5, 164.7, 153.4, 144.0, 138.8, 136.4, 134.9, 133.9, 133.3, 131.6, 130.6, 129.5, 129.4, 129.2, 123.8, 121.1, 120.8, 89.0, 71.6, 64.2, 58.4, 26.6, 14.1, 9.0.

HRMS (ESI) calcd for $C_{36}H_{42}O_8NBClS$ $[M+H]^+$: 694.2407, found: 694.2401.

36. Ethyl 7-chloro-3-(1-(1-tosyl-1*H*-indol-4-yl)allyl)-2,3-dihydrobenzo[*d*]isothiazole-3-carboxylate 1,1-dioxide (32, wrh-08-29A)

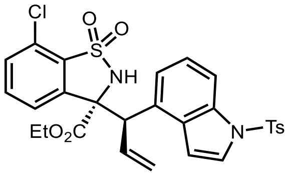

Compound **32** was prepared as a colorless oil (71.8 mg, 61% yield, >20:1 d.r., eluent: 2:1 hexanes/EtOAc) from 4-allyl-1-tosyl-1*H*-indole **32b** according to general procedure A5.

^{1}H NMR (400 MHz, $CDCl_3$) δ 7.94 (d, *J* = 8.3 Hz, 1H), 7.83 – 7.73 (m, 3H), 7.66 – 7.58 (m, 2H), 7.53 (d, *J* = 7.8 Hz, 1H), 7.49 (d, *J* = 7.6 Hz, 1H), 7.31 (t, *J* = 7.9 Hz, 1H), 7.23 (d, *J* = 8.1 Hz, 2H), 6.88 (d, *J* = 3.7 Hz, 1H), 6.11 (dt, *J* = 16.9, 9.6 Hz, 1H), 5.81 (s, 1H), 4.95 (d, *J* = 10.1 Hz, 1H), 4.84 (d, *J* = 16.9 Hz, 1H), 4.51 (d, *J* = 9.1 Hz, 1H), 3.81 (dq, *J* = 10.7, 7.1 Hz, 1H), 3.65 (dq, *J* = 10.8, 7.1 Hz, 1H), 2.34 (s, 3H), 0.78 (t, *J* = 7.1 Hz, 3H).

^{13}C NMR (100 MHz, $CDCl_3$) δ 168.5, 145.3, 139.1, 135.3, 135.1, 134.7, 133.8, 133.7, 131.5, 130.9, 130.1, 129.9, 129.4, 127.0, 126.3, 125.0, 123.9, 123.3, 120.1, 113.2, 107.6, 71.6, 63.7, 55.2, 21.7, 13.5.

HRMS (ESI) calcd for $C_{28}H_{26}O_6N_2ClS_2$ $[M+H]^+$: 585.0915, found: 585.0917.

37. Ethyl 7-chloro-3-(1-(6-(trifluoromethyl)pyridin-3-yl)allyl)-2,3-dihydrobenzo [*d*]isothiazole-3-carboxylate 1,1-dioxide (33, wrh-08-26)

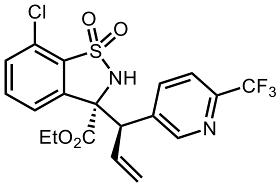

Compound **33** was prepared as a pale yellow oil (82.0 mg, 89% yield (including both regio-isomers), 18:1 d.r., eluent: 2:1 → 1.5:1 hexanes/EtOAc) from 5-allyl-2-(trifluoromethyl)pyridine **33b** according to general procedure A5.
^{1}H NMR (500 MHz, $CDCl_3$) δ 8.65 (s, 1H), 8.11 (d, *J* = 8.1 Hz, 1H), 7.78 (d, *J* = 7.8 Hz, 1H), 7.73 – 7.63 (m, 2H), 7.57 (d, *J* = 7.8 Hz, 1H), 6.05 (dt, *J* = 16.9, 9.7 Hz, 1H), 5.83 (s, 1H), 5.05 (d, *J* = 10.2 Hz, 1H), 4.92 (d, *J* = 16.8 Hz, 1H), 4.31 (d, *J* = 9.3 Hz, 1H), 4.26 – 4.10 (m, 2H), 1.23 (t, *J* = 7.2 Hz, 3H).
^{13}C NMR (125 MHz, $CDCl_3$) δ 168.3, 150.2, 148.0 (q, *J* = 35.0 Hz), 138.4, 138.0, 137.3, 135.1, 134.0, 132.4, 131.9, 129.7, 123.6, 121.7, 121.5 (q, *J* = 272 Hz), 120.6 (q, *J* = 2.5 Hz), 71.3, 64.7, 55.4, 14.1.
^{19}F NMR (471 MHz, $CDCl_3$) δ –67.91.
HRMS (ESI) calcd for $C_{19}H_{15}O_4N_2ClF_3S$ $[M–H]^–$: 459.0399, found: 459.0406.

38. Ethyl 3-(1-(benzo[*b*]thiophen-2-yl)allyl)-7-chloro-2,3-dihydrobenzo[*d*]isothiazole-3-carboxylate 1,1-dioxide (34, wrh-08-19A)

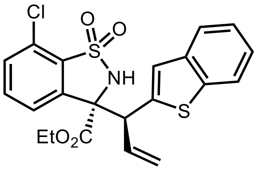

Compound **34** was prepared as a white solid (79.5 mg, 89% yield (including both regio-isomers), 9.7:1 b/l, 15:1 d.r., eluent: 3:1 hexanes/EtOAc) from 2-allylbenzo[*b*]thiophene **34b** according to general procedure A5.
^{1}H NMR (400 MHz, $CDCl_3$) δ 7.83 – 7.76 (m, 2H), 7.73 (d, *J* = 7.3 Hz, 1H), 7.65 (t, *J* = 7.8 Hz, 1H), 7.54 (d, *J* = 7.8 Hz, 1H), 7.38 – 7.28 (m, 3H), 6.04 (dt, *J* = 17.1, 9.6 Hz, 1H), 5.96 (s, 1H), 5.02 (d, *J* = 10.1 Hz, 1H), 4.95 (d, *J* = 16.9 Hz, 1H), 4.58 (d, *J* = 9.1 Hz, 1H), 4.27 – 4.13 (m, 2H), 1.23 (t, *J* = 7.1 Hz, 3H).
^{13}C NMR (100 MHz, $CDCl_3$) δ 168.6, 140.6, 139.6, 139.3, 138.3, 134.9, 133.8, 133.4, 131.7, 129.5, 124.6, 123.7, 123.6, 123.3, 122.3, 120.6, 71.6, 64.3, 54.6, 14.0.
HRMS (ESI) calcd for $C_{21}H_{17}O_4NClS_2$ $[M–H]^–$: 446.0293, found: 446.0284.
m.p. = 171 – 173 °C

39. Ethyl 7-chloro-3-(1-(2-oxo-2*H*-chromen-6-yl)allyl)-2,3-dihydrobenzo[*d*]isothiazole-3-carboxylate 1,1-dioxide (35, wrh-08-42)

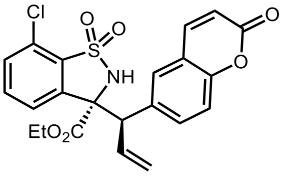

Compound **35** was prepared as a pale yellow oil (49.7 mg, 54% yield, >20:1 d.r., eluent: 1.5:1 → 1:1 hexanes/EtOAc) from 6-allyl-2*H*-chromen-2-one **35b** according to general procedure A5.
^{1}H NMR (400 MHz, $CDCl_3$) δ 7.78 (d, *J* = 7.7 Hz, 1H), 7.74 (d, *J* = 9.6 Hz, 1H), 7.64 – 7.60 (m, 1H), 7.63 (d, *J* = 1.8 Hz, 1H), 7.55 (d, *J* = 7.7 Hz, 1H), 7.52 (dd, *J* = 8.6, 1.9 Hz, 1H), 7.29 (d, *J* = 8.5 Hz, 1H), 6.46 (d, *J* = 9.5 Hz, 1H), 6.07 (dt, *J* = 16.9, 9.8 Hz, 1H), 5.83 (s, 1H), 5.00 (d, *J* = 10.2 Hz, 1H), 4.88 (d, *J* = 16.9 Hz, 1H), 4.25 (d, *J* = 9.2 Hz, 1H), 4.13 (qq, *J* = 10.8, 7.1 Hz, 2H), 1.20 (t, *J* = 7.1 Hz, 3H).
^{13}C NMR (100 MHz, $CDCl_3$) δ 168.6, 160.5, 153.7, 143.3, 138.8, 135.0, 134.6, 133.9, 133.5, 132.5, 131.7, 129.5, 128.1, 123.7, 120.6, 119.2, 117.4, 117.2, 71.7, 64.2, 57.5, 14.2.

HRMS (ESI) calcd for $C_{22}H_{19}O_6NClS$ $[M+H]^+$: 460.0616, found: 460.0610.

40. Ethyl 7-chloro-3-(1-phenylbut-3-en-2-yl)-2,3-dihydrobenzo[*d*]isothiazole-3-carboxylate 1,1-dioxide (36, wrh-08-170B)

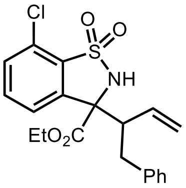

Compound **36** was prepared as a white solid (19.5 mg, 24% yield, 6.2:1 d.r., eluent: 3:1 hexanes/EtOAc) from 4-phenyl-1-butene **36b** according to general procedure A5.
^{1}H NMR (400 MHz, $CDCl_3$) δ 7.62 – 7.50 (m, 2H), 7.47 (dd, *J* = 7.5, 1.3 Hz, 1H), 7.30 – 7.22 (m, 2H), 7.22 – 7.14 (m, 1H), 7.12 – 7.08/6.95 – 6.90* (m, 2H), 5.95/5.92* (s, 1H), 5.68*/5.56 (dt, *J* = 17.1, 9.9 Hz, 1H), 5.09*/4.78 (dd, *J* = 10.3, 1.7 Hz, 1H), 4.91*/4.49 (dd, *J* = 17.1, 1.7 Hz, 1H), 4.33 – 4.19 (m, 2H), 3.28 – 3.18 (m, 1H), 2.81 – 2.61/2.47 – 2.27* (m, 2H), 1.37/1.30* (t, *J* = 7.1 Hz, 3H).
^{13}C NMR (100 MHz, $CDCl_3$) δ 169.9, 139.6, 138.5, 134.6, 133.7, 133.3, 131.3, 129.5, 129.3, 128.4, 126.6, 123.7, 120.9, 71.5, 64.2, 54.7, 37.6, 14.3.
HRMS (ESI) calcd for $C_{20}H_{19}O_4NClS$ $[M–H]^–$: 404.0729, found: 404.0712.
m.p. = 157 – 159 °C

41. Ethyl 3-(but-3-en-2-yl)-7-chloro-2,3-dihydrobenzo[*d*]isothiazole-3-carboxylate 1,1-dioxide (37, wrh-08-171A)

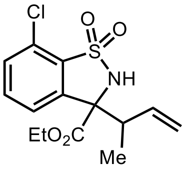

Compound **37** was prepared as a white solid (31.9 mg, 48% yield, 7.3:1 d.r., eluent: 3:1 hexanes/EtOAc) from 1-butene **37b** (27 mL, 1.1 mmol, 5.5 equiv.) according to general procedure A5.
^{1}H NMR (500 MHz, $CDCl_3$) δ 7.67*/7.62 (dd, *J* = 8.0, 1.0 Hz, 1H), 7.57 (t, *J* = 7.8 Hz, 1H), 7.53*/7.48 (dd, *J* = 7.6, 1.1 Hz, 1H), 5.78 (s, 1H), 5.72*/5.58 (ddd, *J* = 17.0, 10.3, 8.7 Hz, 1H), 5.23 – 5.11*/4.91 – 4.80 (m, 2H), 4.34/4.27* (qd, *J* = 7.1, 4.5 Hz, 2H), 3.12 (dq, *J* = 8.8, 6.8 Hz, 1H), 1.36 (t, *J* = 7.2 Hz, 3H), 1.11/0.86* (d, *J* = 6.7 Hz, 3H).
^{13}C NMR (125 MHz, $CDCl_3$) δ 169.8/169.6*, 139.6/138.9*, 137.5*/135.9, 134.8*/134.6, 133.7*/133.4, 131.4*/131.3, 129.4*/129.2, 123.8/123.6*, 118.7/118.2*, 72.0*/71.7, 64.1/63.9*, 47.1/47.0*, 16.3, 14.2/14.1*.
HRMS (ESI) calcd for $C_{14}H_{15}O_4NClS$ $[M–H]^–$: 328.0416, found: 328.0406.
m.p. = 98 – 99 °C

42. Ethyl (*E*)-7-chloro-3-(9-chloronona-1,4-dien-3-yl)-2,3-dihydrobenzo[*d*]isothiazole-3-carboxylate 1,1-dioxide (38, wrh-08-54A)

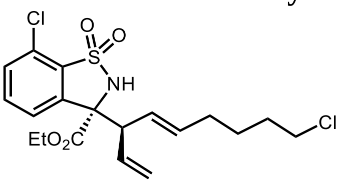

Compound **38** was prepared as a colorless oil (71.6 mg, 83% yield (including all isomers), >10:1 d.r., eluent: 40:1 CH_2Cl_2/Et_2O) from (*E*)-9-chloronona-1,4-diene **38b** according to general procedure A6.
^{1}H NMR (500 MHz, $CDCl_3$) δ 7.64 (d, *J* = 7.9 Hz, 1H), 7.58 (t, *J* = 7.8 Hz, 1H), 7.48 (d, *J* = 7.7 Hz, 1H), 5.81 (s, 1H), 5.70 – 5.57 (m, 2H), 5.45 (dd, *J* = 15.4, 8.5 Hz, 1H), 4.93 (d, *J* = 10.2 Hz, 1H), 4.87 (d, *J* = 17.1 Hz, 1H), 4.28 (q, *J* = 7.1 Hz, 2H), 3.59 (t, *J* = 8.5 Hz, 1H), 3.53 (t, *J* = 6.6 Hz, 2H), 2.12 – 1.99 (m, 2H), 1.79 – 1.70 (m, 2H), 1.54 – 1.46 (m, 2H), 1.33 (t, *J* = 7.1 Hz, 3H).
^{13}C NMR (125 MHz, $CDCl_3$) δ 169.2, 138.8, 134.9, 134.6, 133.7, 133.6, 131.3, 129.3, 126.7, 123.9, 119.5, 71.6, 64.0, 56.1, 44.9, 32.0, 31.9, 26.4, 14.3.
HRMS (ESI) calcd for $C_{19}H_{22}Cl_2NO_4S$ $[M–H]^-$: 430.0652, found: 430.0637.

43. Ethyl (*E*)-7-chloro-3-(1-cyclohexylpenta-1,4-dien-3-yl)-2,3-dihydrobenzo[*d*]-isothiazole-3-carboxylate 1,1-dioxide (39, wrh-08-58A)

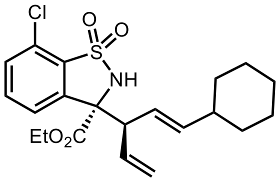

Compound **39** was prepared as a white solid (63.8 mg, 75% yield, >10:1 d.r., eluent: 250:1 CH_2Cl_2/Et_2O) from (*E*)-penta-1,4-dien-1-ylcyclohexane **39b** according to general procedure A6.
^{1}H NMR (500 MHz, $CDCl_3$) δ 7.64 (d, *J* = 7.8 Hz, 1H), 7.57 (t, *J* = 7.8 Hz, 1H), 7.48 (d, *J* = 7.7 Hz, 1H), 5.80 (s, 1H), 5.65 (ddd, *J* = 17.3, 10.2, 8.3 Hz, 1H), 5.57 (dd, *J* = 15.5, 6.8 Hz, 1H), 5.38 (dd, *J* = 15.5, 8.7 Hz, 1H), 4.93 (d, *J* = 10.2 Hz, 1H), 4.86 (d, *J* = 17.1 Hz, 1H), 4.26 (q, *J* = 7.1 Hz, 2H), 3.55 (t, *J* = 8.5 Hz, 1H), 1.99 – 1.89 (m, 1H), 1.75 – 1.61 (m, 5H), 1.34 (t, *J* = 7.1 Hz, 3H), 1.31 – 1.21 (m, 2H), 1.19 – 1.09 (m, 1H), 1.08 – 0.97 (m, 2H).
^{13}C NMR (125 MHz, $CDCl_3$) δ 169.2, 141.6, 138.9, 134.5, 133.8, 133.7, 131.3, 129.3, 123.9, 123.6, 119.4, 71.9, 63.9, 56.2, 40.8, 33.0, 32.9, 26.02, 25.99, 26.0, 14.3.
HRMS (ESI) calcd for $C_{21}H_{27}O_4NClS$ $[M+H]^+$: 424.1344, found: 424.1346.
m.p. = 133 – 134 °C

44. Ethyl (*E*)-7-chloro-3-(6-cyclohexylhexa-1,4-dien-3-yl)-2,3-dihydrobenzo[*d*]-isothiazole-3-carboxylate 1,1-dioxide (40, wrh-08-57B)

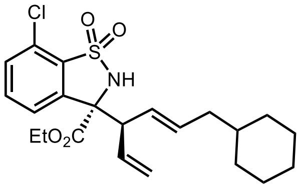

Compound **40** was prepared as a colorless oil (52.5 mg, 60% yield, >10:1 d.r., eluent: 100% CH_2Cl_2 → 5:1 hexanes/EtOAc) from (*E*)-hexa-2,5-dien-1-ylcyclohexane **40b** according to general procedure A6.
^{1}H NMR (400 MHz, $CDCl_3$) δ 7.64 (d, *J* = 7.8 Hz, 1H), 7.58 (t, *J* = 7.8 Hz, 1H), 7.49 (d, *J* = 7.7 Hz, 1H), 5.80 (s, 1H), 5.71 – 5.56 (m, 2H), 5.40 (dd, *J* = 15.3, 8.6 Hz, 1H), 4.94 (d, *J* = 10.2 Hz, 1H), 4.87 (d, *J* = 17.1 Hz, 1H), 4.27 (q, *J* = 7.1 Hz, 2H), 3.60 (t, *J* = 8.4 Hz, 1H), 1.97 – 1.85 (m, 2H), 1.74 – 1.59 (m, 5H), 1.33 (t, *J* = 7.1 Hz, 3H), 1.30 – 1.09 (m, 4H), 0.94 – 0.79 (m, 2H).
^{13}C NMR (100 MHz, $CDCl_3$) δ 169.3, 138.9, 134.55, 134.52, 133.8, 133.7, 131.3, 129.3, 126.9, 123.9, 119.4, 71.7, 63.9, 56.1, 40.7, 37.9, 33.2, 33.1, 26.6, 26.4, 14.2.

HRMS (ESI) calcd for $C_{22}H_{29}O_4NClS$ $[M+H]^+$: 438.1500, found: 438.1501.

45. Ethyl (*E*)-7-chloro-3-(7-phenylhepta-1,4-dien-3-yl)-2,3-dihydrobenzo[*d*]isothiazole-3-carboxylate 1,1-dioxide (41, wrh-08-58B)

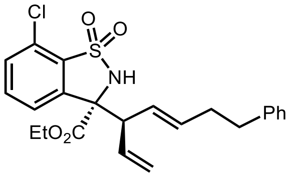

Compound **41** was prepared as a white solid (58.9 mg, 66% yield, >5:1 d.r., eluent: 250:1 CH_2Cl_2/EtOAc) from (*E*)-hepta-3,6-dien-1-ylbenzene **41b** according to general procedure A6.
^{1}H NMR (400 MHz, $CDCl_3$) δ 7.61 (d, *J* = 7.7 Hz, 1H), 7.56 (t, *J* = 7.7 Hz, 1H), 7.48 (d, *J* = 7.5 Hz, 1H), 7.30 (t, *J* = 7.4 Hz, 2H), 7.24 – 7.13 (m, 3H), 5.72 – 5.57 (m, 3H), 5.45 (dd, *J* = 15.4, 8.3 Hz, 1H), 4.94 (d, *J* = 10.2 Hz, 1H), 4.84 (d, *J* = 17.1 Hz, 1H), 4.30 – 4.14 (m, 2H), 3.58 (t, *J* = 8.3 Hz, 1H), 2.75 – 2.61 (m, 2H), 2.42 – 2.31 (m, 2H), 1.29 (t, *J* = 7.1 Hz, 3H).
^{13}C NMR (100 MHz, $CDCl_3$) δ 169.2, 141.4, 138.8, 134.7, 134.5, 133.7, 133.5, 131.3, 129.3, 128.6, 128.5, 126.8, 126.2, 123.9, 119.6, 71.5, 63.9, 55.9, 35.6, 34.2, 14.3.
HRMS (ESI) calcd for $C_{23}H_{25}O_4NClS$ $[M+H]^+$: 446.1187, found: 446.1187.
m.p. = 118 – 120 °C

46. Ethyl (*E*)-3-(8-(benzoyloxy)octa-1,4-dien-3-yl)-7-chloro-2,3-dihydrobenzo[*d*]-isothiazole-3-carboxylate 1,1-dioxide (42, wrh-08-61A)

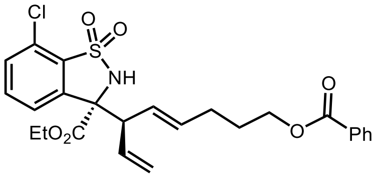

Compound **42** was prepared as a white solid (65.7 mg, 65% yield, >10:1 d.r., eluent: 3:1 → 2:1 hexanes/EtOAc, then recrystallized using CH_2Cl_2 (0.5 mL) and hexanes (3 mL)) from (*E*)-octa-4,7-dien-1-yl benzoate **42b** according to general procedure A6.
^{1}H NMR (400 MHz, $CDCl_3$) δ 8.05 (d, *J* = 7.5 Hz, 2H), 7.63 (d, *J* = 7.7 Hz, 1H), 7.61 – 7.53 (m, 2H), 7.52 – 7.40 (m, 3H), 5.88 (s, 1H), 5.73 – 5.59 (m, 2H), 5.57 – 5.46 (m, 1H), 4.95 (d, *J* = 10.1 Hz, 1H), 4.87 (d, *J* = 17.1 Hz, 1H), 4.39 – 4.23 (m, 4H), 3.61 (t, *J* = 8.3 Hz, 1H), 2.27 – 2.12 (m, 2H), 1.93 – 1.77 (m, 2H), 1.32 (t, *J* = 7.1 Hz, 3H).
^{13}C NMR (100 MHz, $CDCl_3$) δ 169.2, 166.7, 138.8, 134.5, 134.1, 133.7, 133.5, 133.1, 131.3, 130.4, 129.7, 129.3, 128.5, 127.4, 123.9, 119.7, 71.6, 64.1, 64.0, 56.0, 29.1, 28.4, 14.3.
HRMS (ESI) calcd for $C_{25}H_{25}O_6NClS$ $[M-H]^-$: 502.1097, found: 502.1103.
m.p. = 135 – 137 °C

Unsuccessful substrates

Ethyl 3-allyl-7-chloro-2,3-dihydrobenzo[*d*]isothiazole-3-carboxylate 1,1-dioxide (wrh-08-169B)

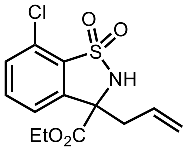

This compound was prepared as a colorless oil (18% NMR yield) from propylene (27 mL, 1.1 mmol, 5.5 equiv.) according to general procedure A5.
^{1}H NMR (400 MHz, $CDCl_3$) δ 7.64 (dd, *J* = 7.8, 1.2 Hz, 1H), 7.59 (t, *J* = 7.7 Hz, 1H), 7.52 (dd, *J* = 7.8, 1.2 Hz, 1H), 5.85 – 5.70 (m, 2H), 5.26 – 5.16 (m, 2H), 4.32 (qd, *J* = 7.2, 1.5 Hz, 2H), 2.94 (dd, *J* = 13.9, 7.8 Hz, 1H), 2.74 (ddt, *J* = 13.8, 6.4, 1.3 Hz, 1H), 1.35 (t, *J* = 7.1 Hz, 3H).
^{13}C NMR (100 MHz, $CDCl_3$) δ 169.2, 140.6, 134.7, 133.8, 131.4, 130.8, 129.4, 123.3, 121.3, 67.9, 63.9, 44.7, 14.3.
HRMS (ESI) calcd for $C_{13}H_{13}O_4NClS$ $[M–H]^–$: 314.0259, found: 314.0253.

1-Phenylocta-5,7-dien-3-ol (wrh-08-172A)

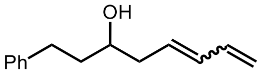

This compound was prepared (5% NMR yield) from 3-phenylpropanal according to general procedure A1 using $BF_3·Et_2O$ as Lewis acid.

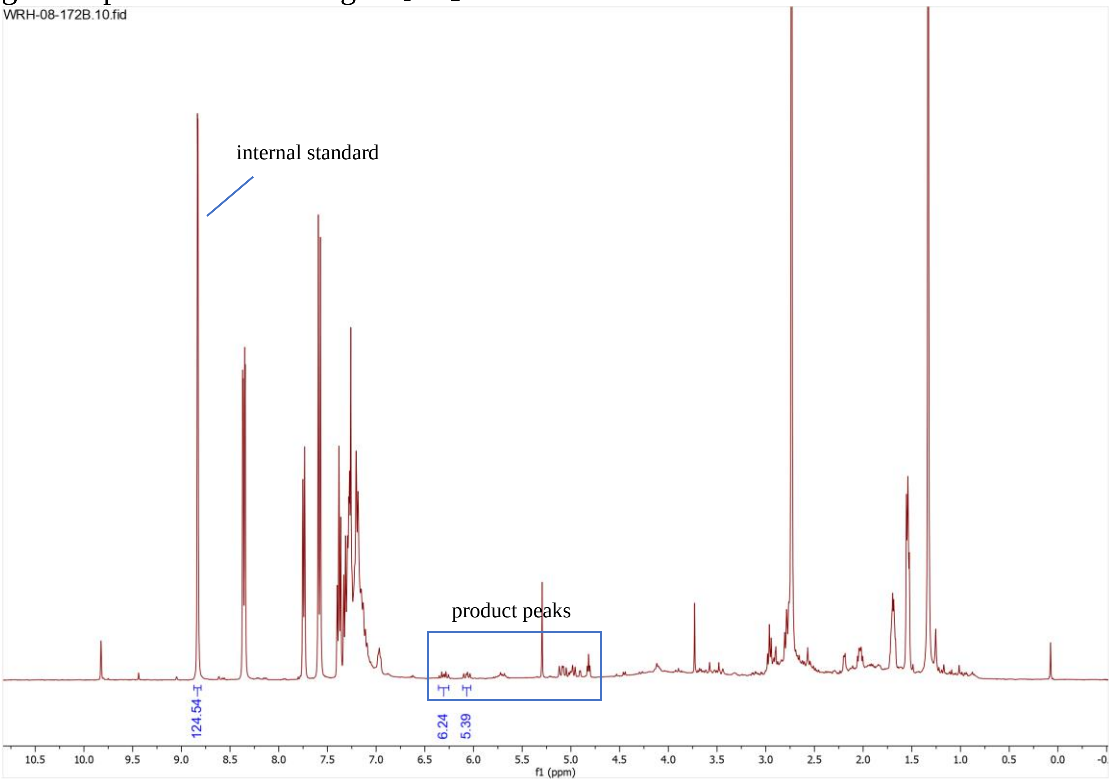

1-(1-Benzylcyclopentyl)hexa-3,5-dien-1-ol (wrh-08-166B)

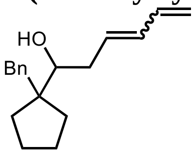

This compound was prepared (16% NMR yield) from 1-benzylcyclopentane-1-carbaldehyde according to general procedure A1 using $BF_3·Et_2O$ as Lewis acid.

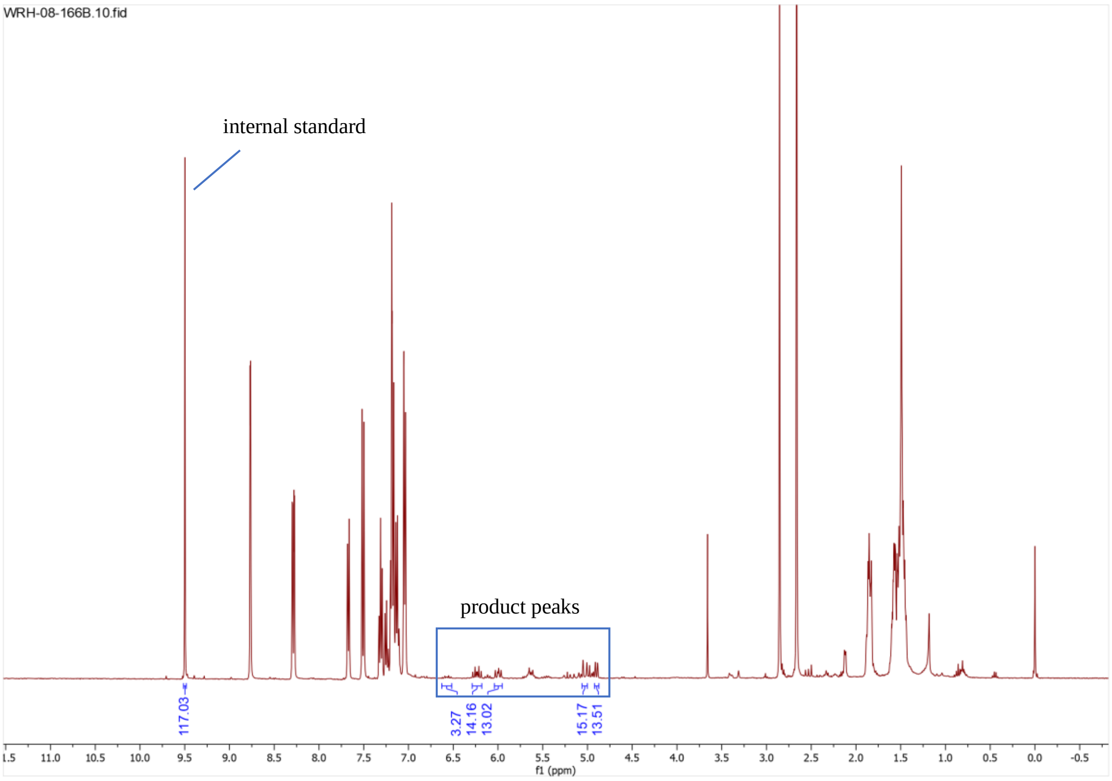
WRH-08-166B.10.fid
internal standard
product peaks
117.03
3.27
14.16
13.02
15.17
13.51
f1 (ppm)

Product derivatization

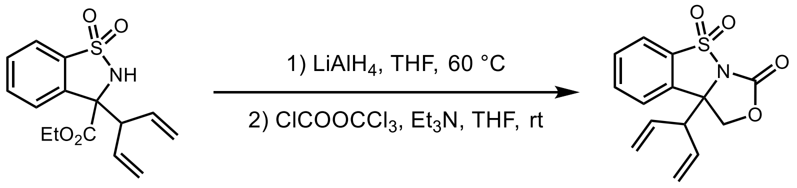

12 **43**

91% yield

This compound was synthesized according to modified literature procedure.[2]

To a flame-dried round bottom flask was added lithium aluminium hydride (17.1 mg, 0.45 mmol, 1.5 equiv.), anhydrous THF (1 mL), and compound **12** (92.2 mg, 0.3 mmol, 1.0 equiv.). The mixture was stirred at 60 °C for 6 h, then cooled to room temperature, quenched with aqueous NH_4Cl solution (2 mL) and extracted with EtOAc (3 × 2 mL). The combined organic layers were dried over magnesium sulfate and concentrated under reduced pressure. The crude product was used in the next step without further purification. Subsequently, the residue was dissolved in THF (5 mL) at 0 °C, and triethylamine (209 µL, 1.5 mmol, 5.0 equiv.) was added. The mixture was stirred for 10 min at 0 °C, then diphosgene (43.2 µL, 0.36 mmol, 1.2 euqiv.) was added over 2 minutes. After stirring at 0 °C for 4 h, the mixture was quenched with H_2O (5 mL) and extracted with EtOAc (3 × 10 mL). The combined organic layers were dried over magnesium sulfate and concentrated under reduced pressure. The residue was purified by flash chromatography on silica gel to afford product **43** (79.7 mg, 91%, eluent: 1:1 hexanes/EtOAc) as a pale yellow solid.

^{1}H NMR (300 MHz, $CDCl_3$) δ 7.87 – 7.78 (m, 1H), 7.78 – 7.59 (m, 2H), 7.33 (d, *J* = 7.7 Hz, 1H), 5.99 – 5.78 (m, 2H), 5.40 (d, *J* = 10.3, 1H), 5.28 (d, *J* = 17.0, 1H), 5.18 (d, *J* = 10.4, 1H), 5.11 (d, *J* = 17.0, 1H), 4.78 (d, *J* = 8.8 Hz, 1H), 4.37 (d, *J* = 8.8 Hz, 1H), 3.31 (t, *J* = 8.3 Hz, 1H).

^{13}C NMR (76 MHz, $CDCl_3$) δ 151.3, 138.3, 136.2, 134.3, 132.6, 132.2, 130.9, 124.2, 122.6, 121.9, 121.0, 71.7, 71.1, 56.9.

HRMS (ESI) calcd for $C_{14}H_{14}O_4NS$ $[M+H]^+$: 292.0638, found: 292.0637.

m.p. = 145 – 147 ℃

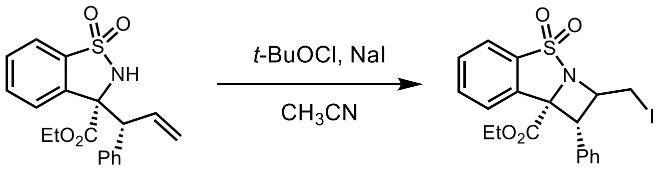

44 **45**

This compound was synthesized according to modified literature procedure.[3]

To a mixture of **44** (71.5 mg, 0.2 mmol, 1.0 equiv.) and NaI (30.0 mg, 0.2 mmol, 1.0 equiv.) in CH_3CN (1.2 mL) was added *t*-BuOCl (22.7 µL, 0.2 mmol, 1.0 equiv.) under an atmosphere of nitrogen. The mixture was allowed to stir in the dark at room temperature for 18 h. The solvent was removed under reduced pressure and the residue was purified by flash column chromatography on silica gel (eluent: 3:1 hexanes/EtOAc) followed by preparative TLC (eluent: 100% CH_2Cl_2) to obtain product **45** as a colorless oil (49.3 mg, 51% yield, 1.7:1 d.r.). The two diastereomers are separable, and their characterization data were reported separately.

Under the guidance of computational analysis, the relative configuration of the diastereomers was proposed based on the NOE signals of H1–H3 coupling. The optimized geometries of the two diastereomers were computed at the B3LYP/LanL2DZ level of theory. As depicted in the 3D models below, H1 and H3 are proximal to each other in the anti-isomer (left), thus a remote coupling is expected; while these nuclei are distant in the syn-isomer (right), therefore the coupling is not likely to be observed. The NOE spectra of the two diastereomers clearly demonstrate the difference of the corresponding signals, which are presented in the following pages.

According to ^{1}H NMR analysis of the reaction mixture, **45-anti** is identified as the major diastereomer.

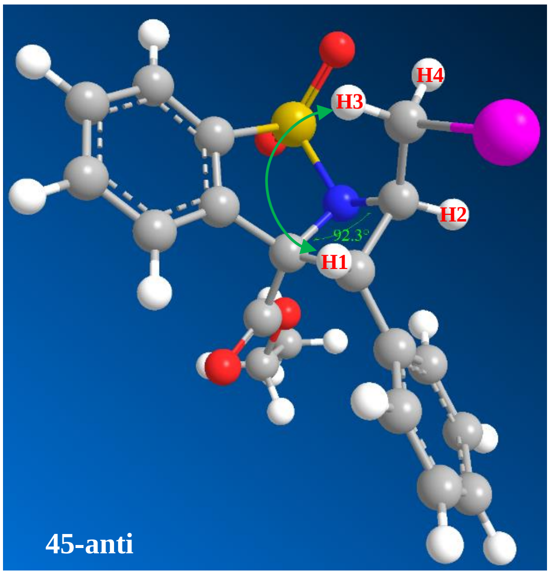

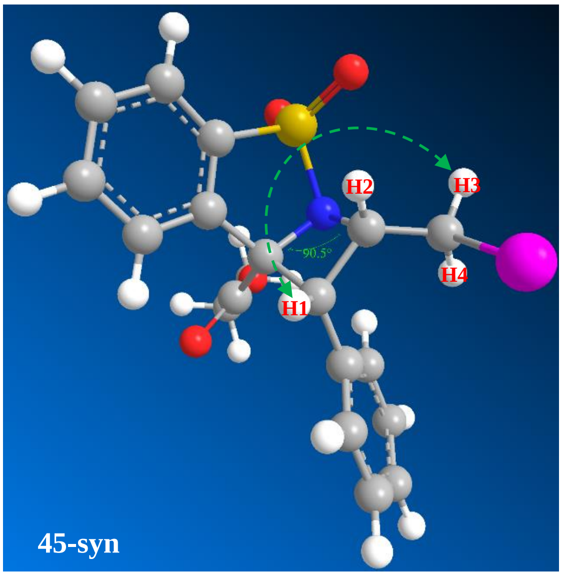

45-syn:

^{1}H NMR (400 MHz, $CDCl_3$) δ 7.88 – 7.81 (m, 2H), 7.79 – 7.72 (m, 1H), 7.71 – 7.64 (m, 1H), 7.56 – 7.48 (m, 2H), 7.39 (dd, *J* = 5.0, 2.0 Hz, 3H), 4.39 (ddd, *J* = 12.1, 8.0, 4.1 Hz, 1H), 4.00 – 3.87 (m, 3H), 3.47 (dd, *J* = 9.6, 4.2 Hz, 1H), 3.23 (dd, *J* = 12.1, 9.6 Hz, 1H), 0.91 (t, *J* = 7.1 Hz, 3H).
^{13}C NMR (100 MHz, $CDCl_3$) δ 166.4, 138.6, 135.1, 134.4, 132.9, 131.4, 130.0, 128.7, 128.6, 126.1, 122.9, 75.1, 63.8, 62.4, 51.4, 13.7, 1.4.
HRMS (ESI) calcd for $C_{19}H_{19}O_4NIS$ $[M+H]^+$: 484.0074, found: 484.0071.

^{1}H–^{1}H COSY

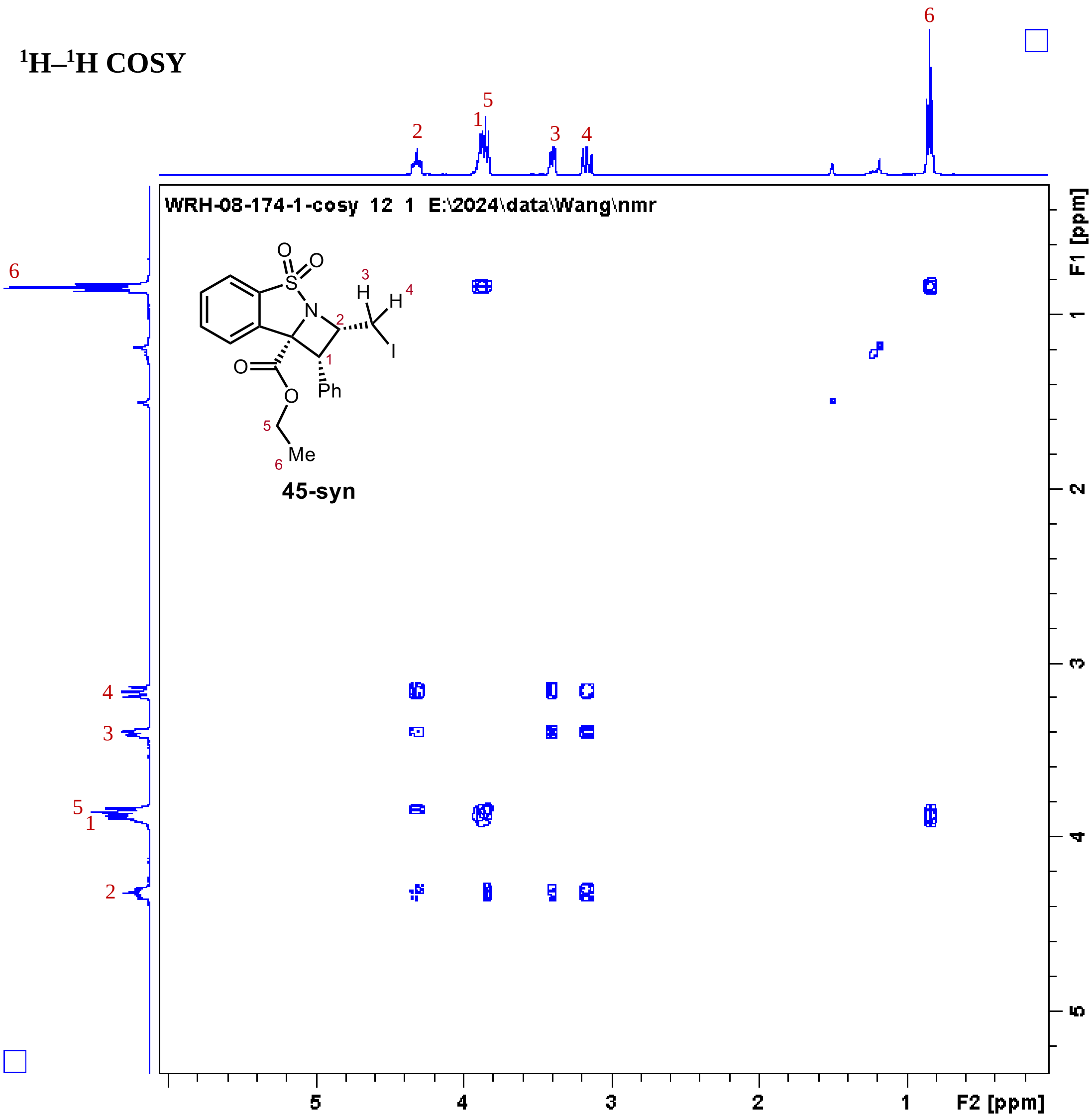
WRH-08-174-1-cosy 12 1 E:\2024\data\Wang\nmr
45-syn
F1 [ppm]
F2 [ppm]
6
5
1
2
3
4

HSQC

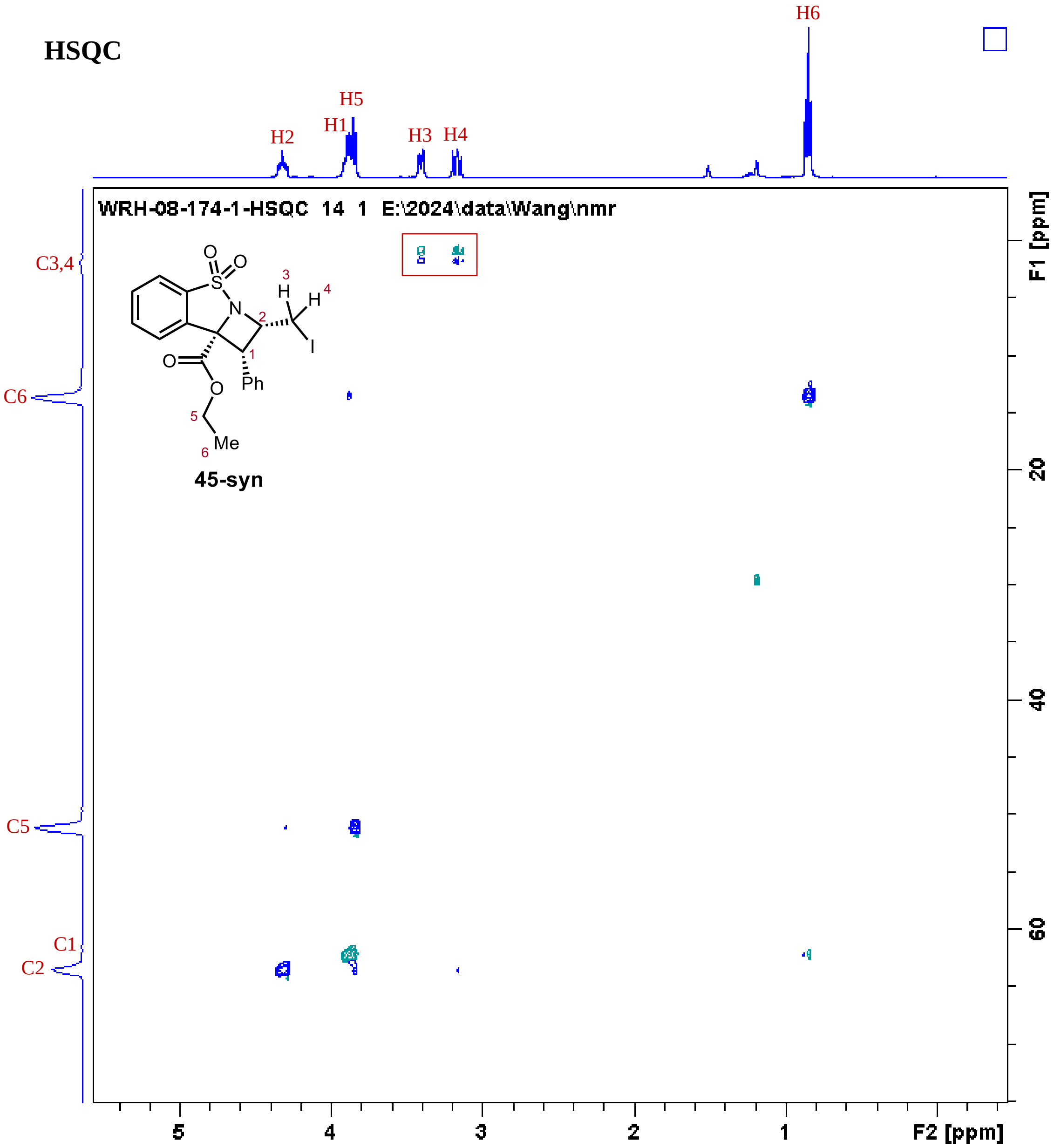
H6
H5
H1
H2
H3
H4
WRH-08-174-1-HSQC 14 1 E:\2024\data\Wang\nmr
C3,4
C6
C5
C1
C2
45-syn
F1 [ppm]
20
40
60
5
4
3
2
1
F2 [ppm]

NOESY

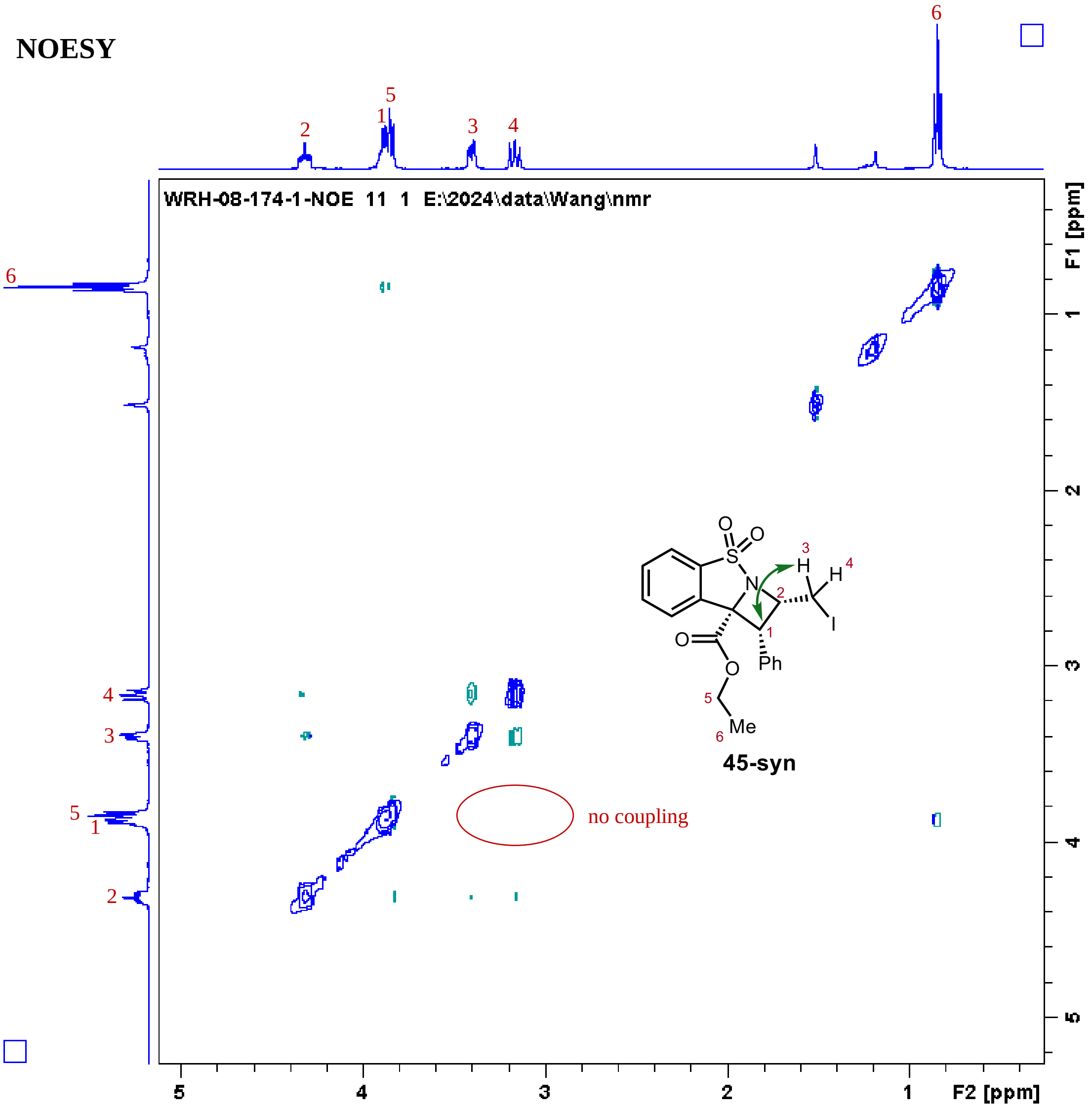

45-anti:

^{1}H NMR (400 MHz, $CDCl_3$) δ 7.86 (d, *J* = 7.8 Hz, 1H), 7.81 – 7.69 (m, 2H), 7.68 – 7.62 (m, 1H), 7.54 – 7.48 (m, 2H), 7.47 – 7.29 (m, 3H), 4.75 (dt, *J* = 13.0, 4.5 Hz, 1H), 4.17 (dd, *J* = 9.8, 4.1 Hz, 1H), 3.82 (q, *J* = 7.2 Hz, 2H), 3.57 (d, *J* = 4.7 Hz, 1H), 2.86 (dd, *J* = 13.0, 9.8 Hz, 1H), 0.86 (t, *J* = 7.1 Hz, 3H).

^{13}C NMR (125 MHz, $CDCl_3$) δ 165.9, 140.7, 138.1, 136.7, 134.2, 131.0, 129.1, 128.6, 128.4, 125.6, 121.4, 76.2, 69.4, 62.4, 57.2, 13.7, 7.9.

HRMS (ESI) calcd for $C_{19}H_{19}O_4NIS$ $[M+H]^+$: 484.0074, found: 484.0072.

^1H–^1H COSY

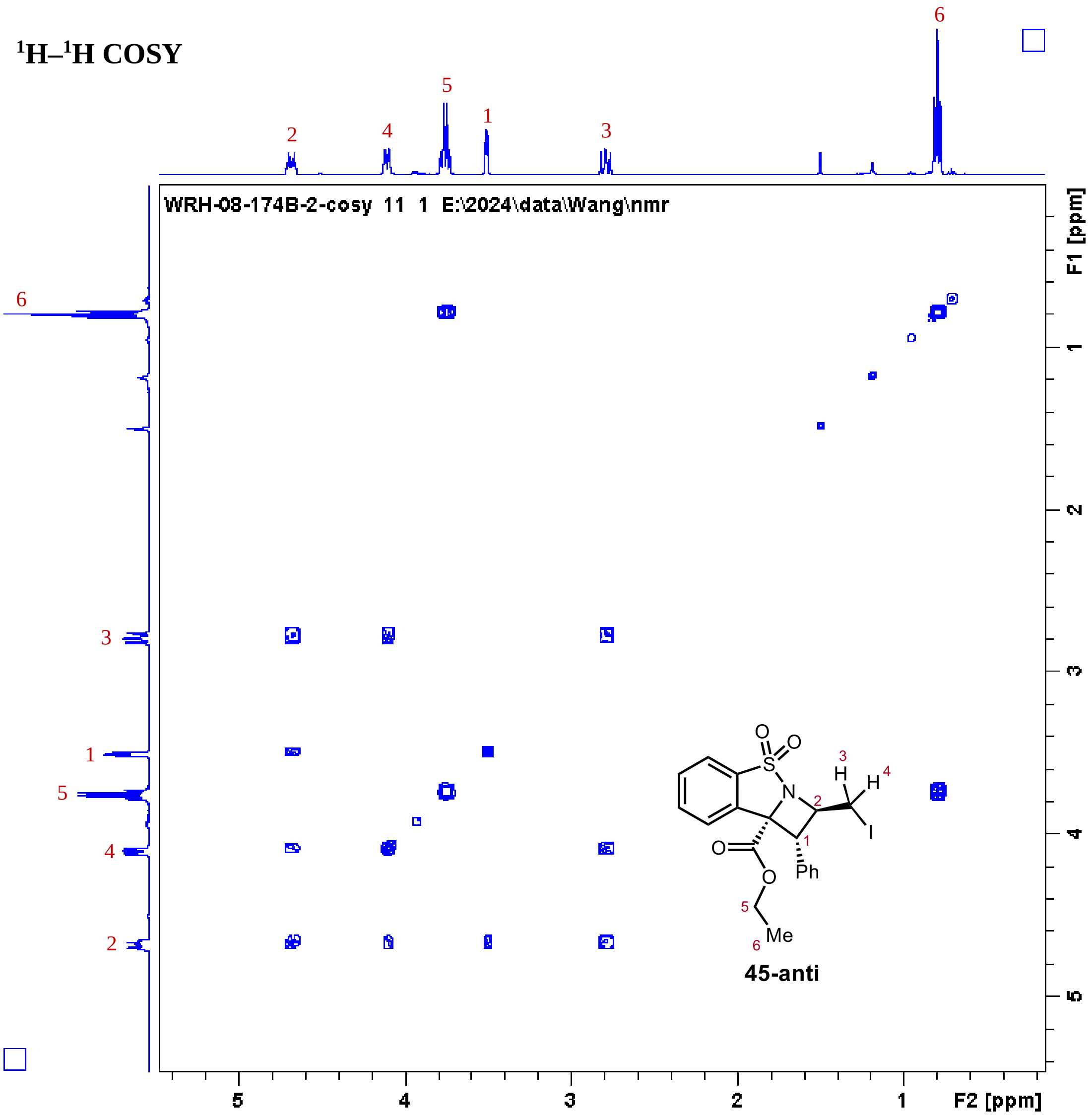
WRH-08-174B-2-cosy 11 1 E:\2024\data\Wang\nmr
F1 [ppm]
F2 [ppm]
1
2
3
4
5
6
45-anti
Ph
Me
O
S
N
H
I

HSQC

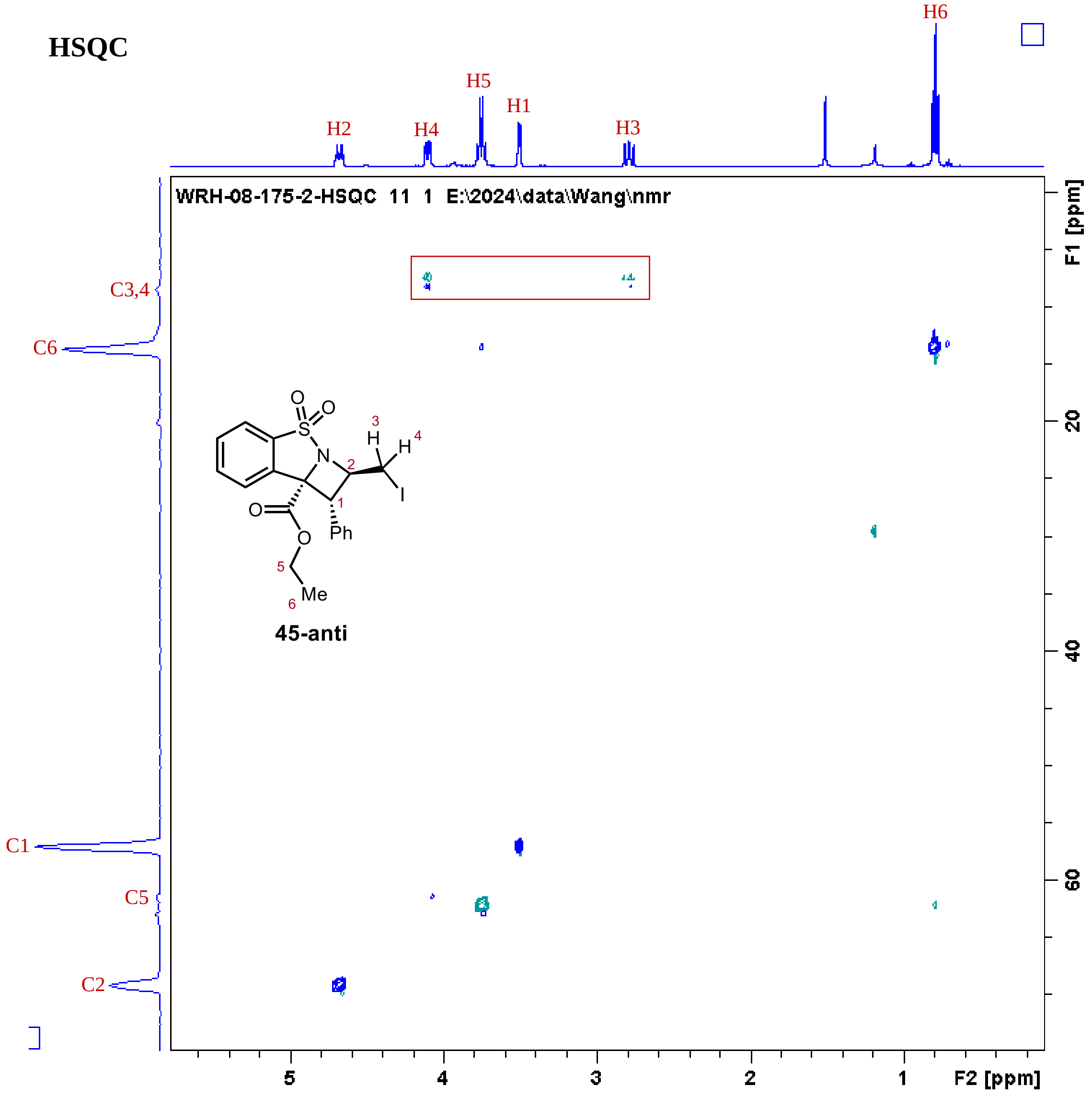

H2
H4
H5
H1
H3
H6
WRH-08-175-2-HSQC 11 1 E:\2024\data\Wang\nmr
C3,4
C6
C1
C5
C2
45-anti
F1 [ppm]
F2 [ppm]

NOESY

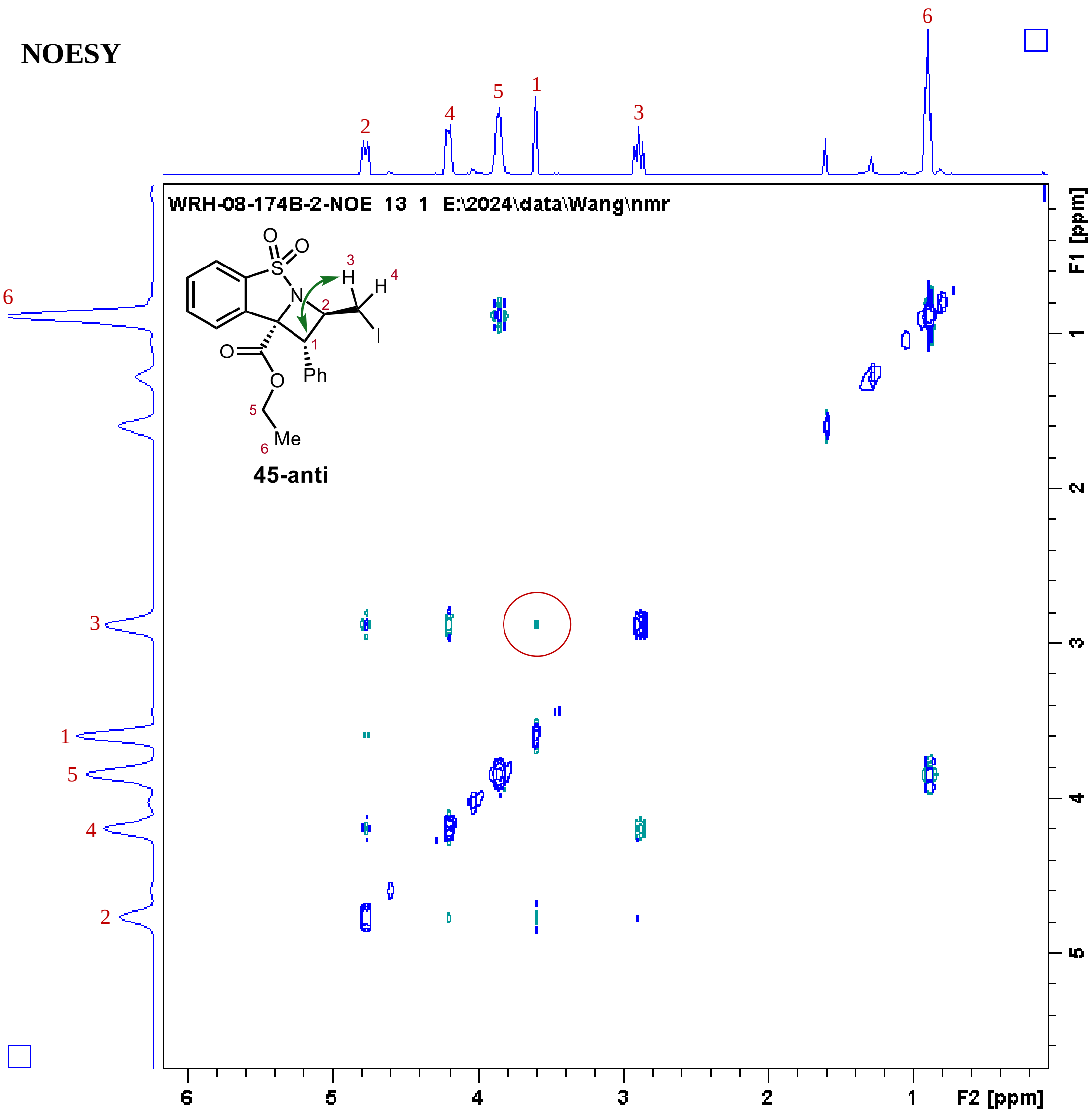

WRH-08-174B-2-NOE 13 1 E:\2024\data\Wang\nmr
45-anti
Ph
Me
F1 [ppm]
F2 [ppm]

Identification of bismuth−olefin interactions

Catalytic reactivities of [$BiMe_2(SbF_6)$] and [$BiPh_2(SbF_6)$]: (Yields were determined by ^{1}H NMR spectroscopy using 2,4-dinitrotoluene as the internal standard.)

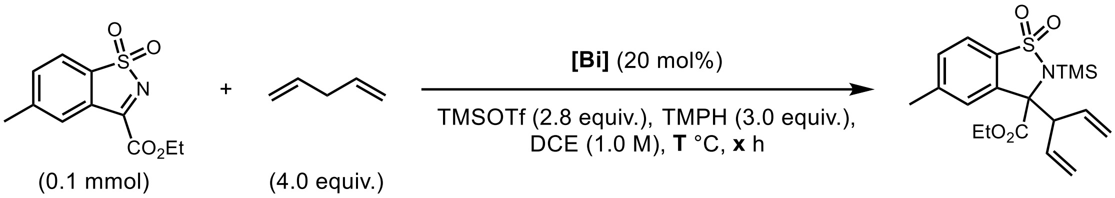

Entry	[Bi]	*T*. °C	*x* h	% yield
1	$BiMe_2(SbF_6)$	80	12	47
2	$BiMe_2(SbF_6)$	22	20	51
3	$BiPh_2(SbF_6)$	80	12	34
4	$BiPh_2(SbF_6)$	22	20	62

Part I: Analysis with [$BiMe_2(SbF_6)$]

NMR-Spectroscopy

General procedure for the *in-situ*-NMR spectroscopic analysis of [$BiMe_2SbF_6$] in the presence of an olefin:
[$BiMe_2(SbF_6)$] (25 mg, 0.053 mmol, 1 eq) was dissolved in CD_2Cl_2 (0.7 mL). The respective olefin (1 eq) was added with a microliter syringe. The samples were analyzed by ^{1}H- and ^{13}C-NMR spectroscopy. An interaction is indicated by a change in the respective NMR chemical shifts of both compounds, without a change in multiplicity.

Olefins used as substrates:

- Cycloheptatriene (5.5 µL, 0.053 mmol, 1 eq.):
  Formation of tropylium cation (NMR)
- Norbornadiene (5.3 µL, 0.053 mmol, 1 eq.):
  broad signals, quick color change and formation of dark solid; $BiMe_3$ detected (NMR)
- Cyclohexadiene (4.9 µL, 0.053 mmol, 1 eq.):
  Formation of benzene, shift of resonance due to [$BiMe_2$]$^+$ observed (NMR), might be coordinated olefin as an intermediate
- Cyclopentene (4.7 µL, 0.053 mmol, 1 eq.):
  coordination (NMR shifts in next section)
- Allylbenzene (7.0 µL, 0.053 mmol, 1 eq.):
  coordination (NMR shifts in next section)
- 1,4-Pentadiene (5.4 µL, 0.053 mmol, 1 eq.):
  coordination (NMR shifts in next section)

NMR-Spectra:

[$BiMe_2(SbF_6)$]

^{1}H-NMR (500 MHz, CD_2Cl_2): δ = 2.28 (s, 6 H) ppm.
^{13}C-NMR (126 MHz, CD_2Cl_2): δ = 64.38 (s) ppm.

Cyclopentene (in the absence of a bismuth compound)

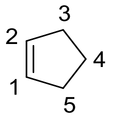

^{1}H-NMR (300 MHz, CD_2Cl_2): δ = 1.82 (quint, 2 H, $^3J_{HH}$=7.50 Hz, H-4), 2.31 (t, 4 H, $^3J_{HH}$=7.43 Hz, H-3, 5), 5.74 (s, 2 H, H-1,2) ppm.
^{13}C-NMR (75 MHz, CD_2Cl_2): δ = 23.19 (s, C-4), 32.80 (s, C-3, 5), 130.99 (s, C-1, 2) ppm.

Cyclopentene (after reaction)

^{1}H-NMR (500 MHz, CD_2Cl_2): δ = 1.84 (quint, 2 H, $^3J_{HH}$=7.45 Hz, H-4), 2.11 (s, 6 H, Bi-CH_3), 2.43 (t, 4 H, $^3J_{HH}$=7.47 Hz, H-3, 5), 6.00 (s, 2H, H-1, 2) ppm.
^{13}C-NMR (126 MHz, CD_2Cl_2): δ = 22.83 (s, C-4), 32.78 (s, C-3, 5), 54.14 (s, Bi-*C*, detected *via* ^{1}H-^{13}C-HSQC spectrum), 133.03 (s, C-1, 2) ppm.

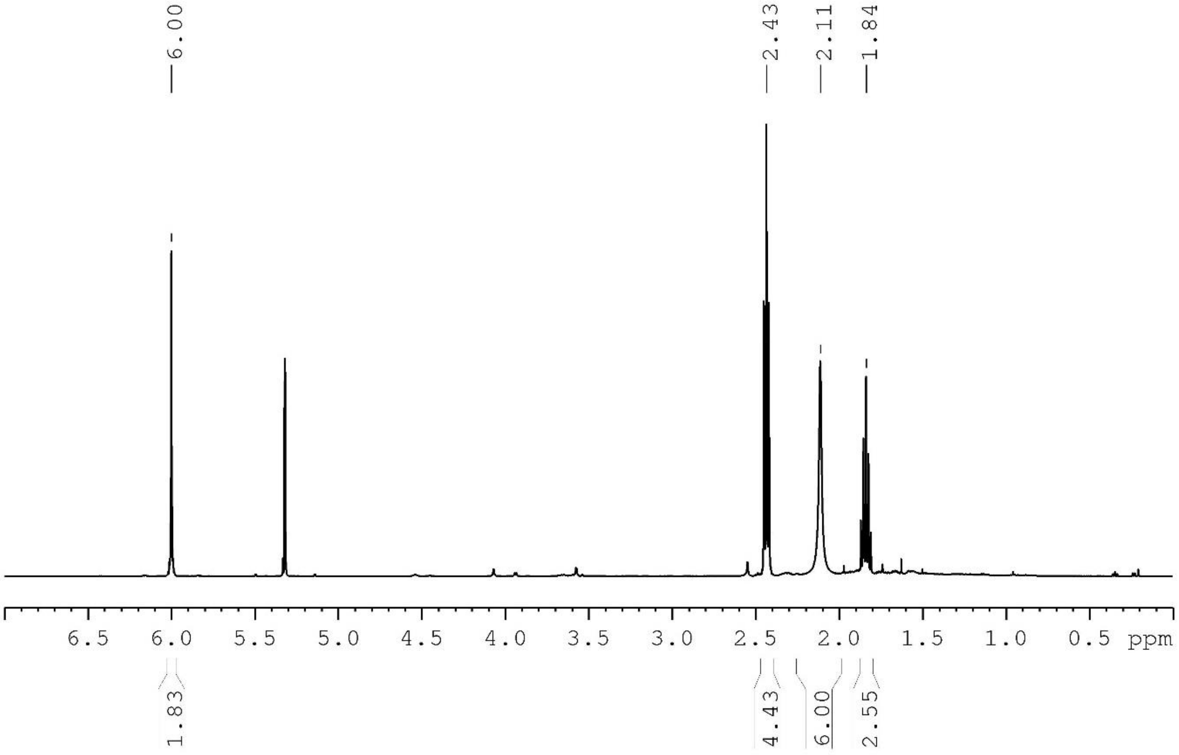

Figure S1. ^{1}H-NMR spectrum of the reaction mixture of [$BiMe_2(SbF_6)$] and cyclopentene.

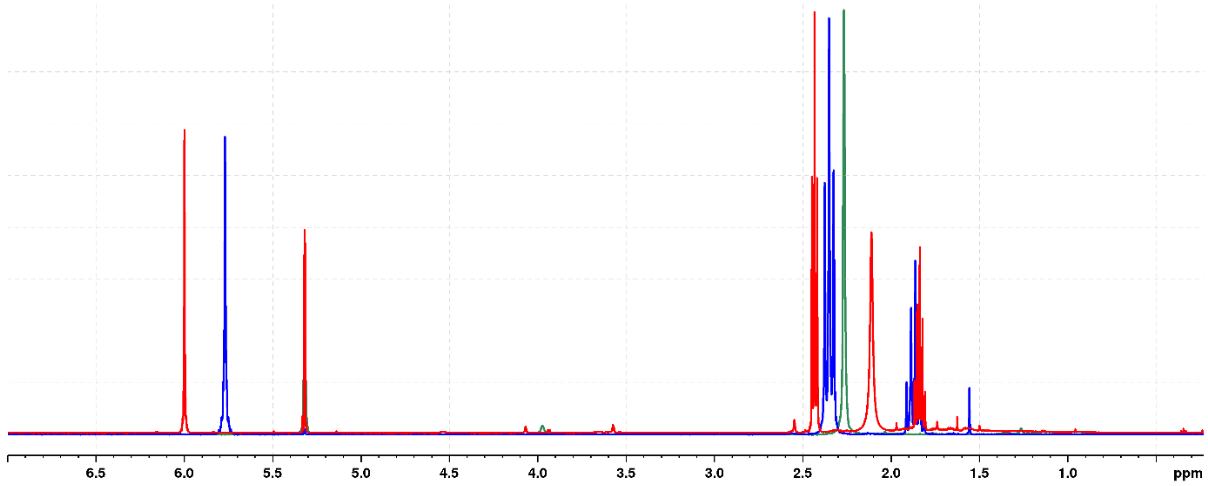

Figure S2. Stacked display of ^{1}H-NMR spectra of reaction mixture and starting materials: green: [$BiMe_2(SbF_6)$], blue: cyclopentene, red: reaction mixture.

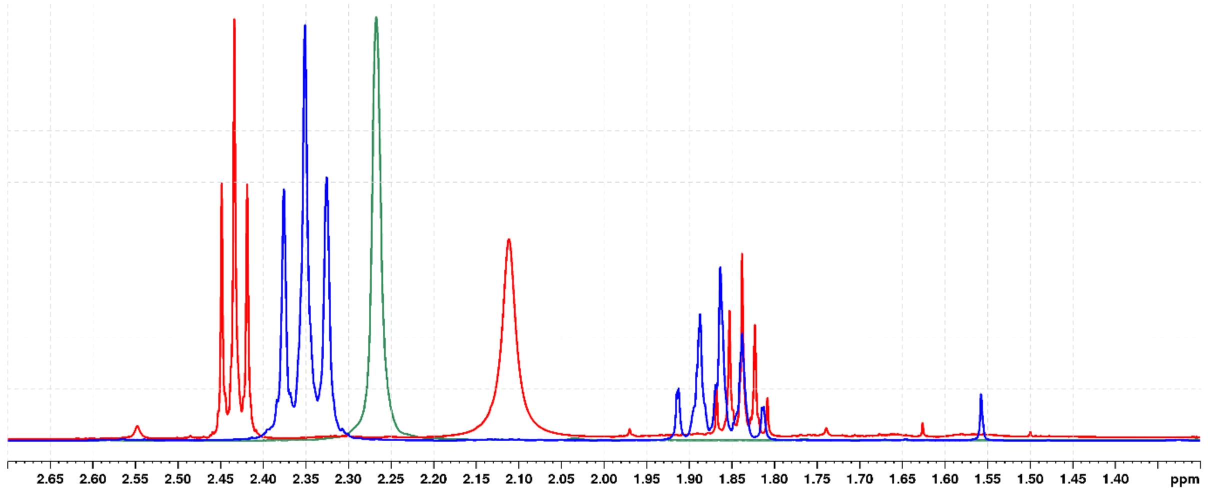

Figure S3. Stacked display of ^{1}H-NMR spectra of the region between 1.3 and 2.8 ppm: green: [$BiMe_2(SbF_6)$], blue: cyclopentene, red: reaction mixture.

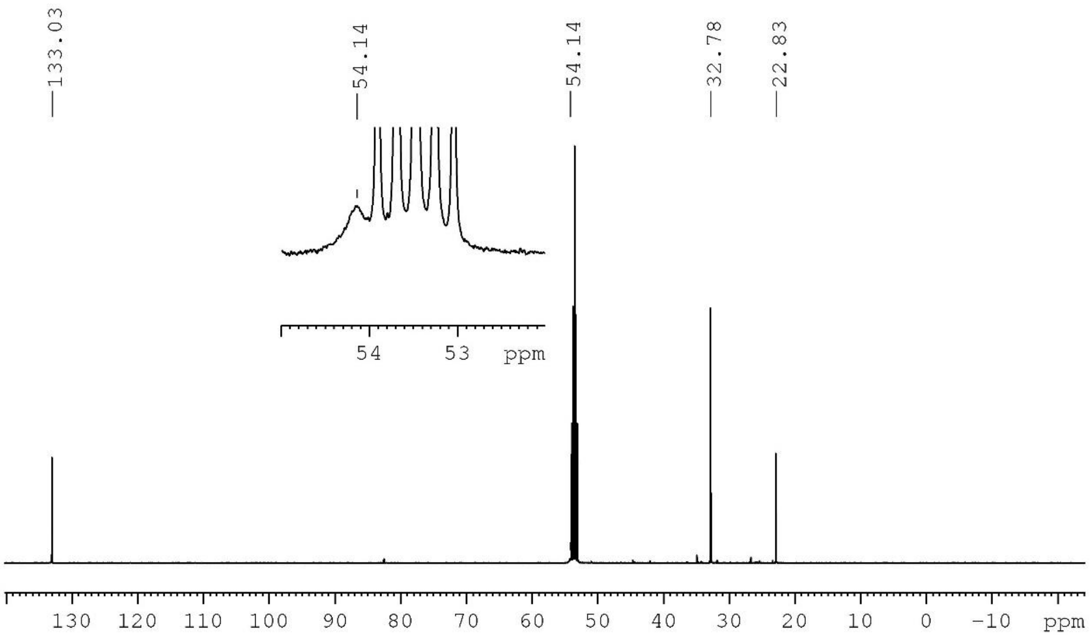

Figure S4. ^{13}C-NMR spectrum of the reaction mixture of [$BiMe_2(SbF_6)$] and cyclopentene. Inset: detailed view around 54 ppm.

Allylbenzene (in the absence of a bismuth compound)

^{1}H-NMR (300 MHz, CD_2Cl_2): δ = 3.42 (d, 2 H, $^3J_{HH}$=6.64 Hz, alkyl-C*H*$_2$), 5.11 (m, 2 H, olefin-C*H*$_2$), 6.01 (m, 1 H, olefin-C*H*), 7.23 (m, 3 H, *meta*-, *para*-C*H*, $^3J_{HH}$=6.90 Hz), 7.32 (t, 2 H, $^3J_{HH}$=7.20 Hz, *ortho*-C*H*) ppm.
^{13}C-NMR (75 MHz, CD_2Cl_2): δ = 40.62 (s, alkyl-*C*H$_2$), 115.85 (s, olefin-*C*H$_2$), 126.44 (s, *para*-*C*H), 128.98 (s, *ortho*-*C*H), 129.01 (s, *meta*-*C*H),138.10 (s, olefin-*C*H), 140.67 (s, *ipso*-*C*) ppm.

Allylbenzene (after reaction)

^{1}H-NMR (300 MHz, CD_2Cl_2): δ = 2.19 (s, 6 H, Bi-C*H*$_3$), 3.41 (d, 2 H, $^3J_{HH}$=6.66 Hz, alkyl-C*H*$_2$,), 5.07 (m, 2 H, olefin-C*H*$_2$), 6.06 (m, 1 H, olefin-C*H*), 7.21 (m, 3 H, *meta*-, *para*-C*H*), 7.32 (t, 2 H, $^3J_{HH}$=7.27 Hz, *ortho*-C*H*,) ppm.
^{13}C-NMR (75 MHz, CD_2Cl_2): δ =40.52 (s, alkyl-*C*H$_2$), 114.14 (s, olefin-*C*H$_2$), 126.73 (s, *para*-*C*H, 128.74 (s, *ortho*-*C*H), 129.25 (s, *meta*-*C*H), 139.62 (s, olefin-*C*H), 140.81 (s, *ipso*-*C*)) ppm.

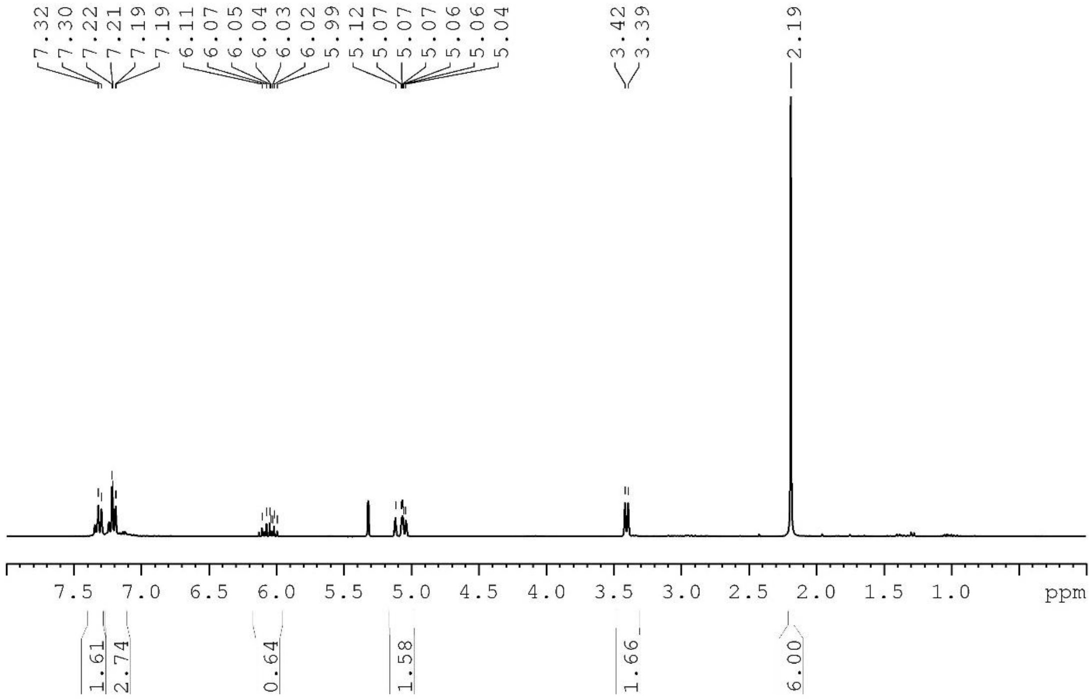

Figure S5. ^{1}H-NMR spectrum of the reaction mixture of [$BiMe_2(SbF_6)$] and allylbenzene.

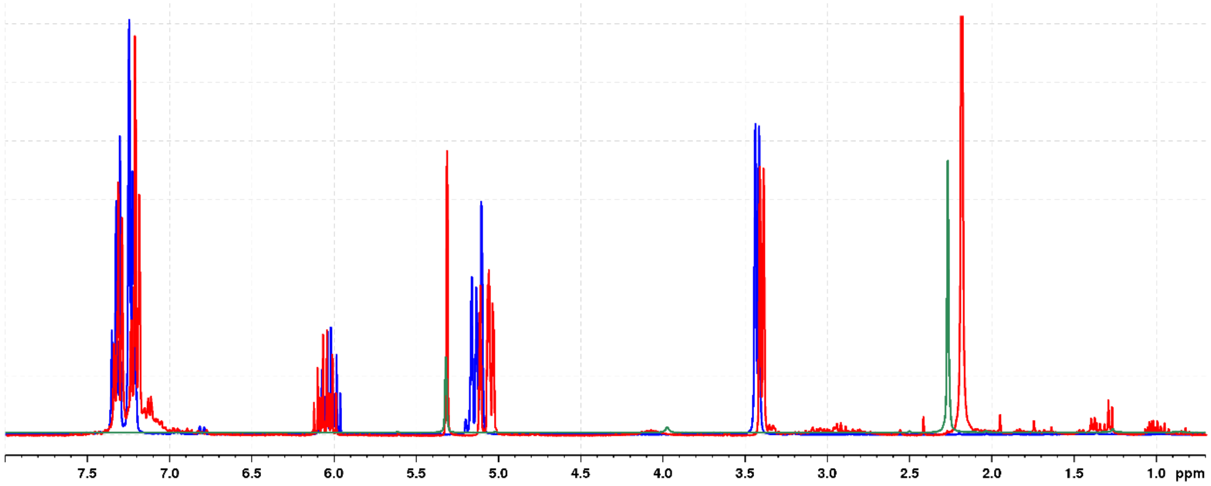

Figure S6. Stacked display of ^{1}H-NMR spectra of reaction mixture and starting materials: green: [$BiMe_2(SbF_6)$], blue: allylbenzene, red: reaction mixture.

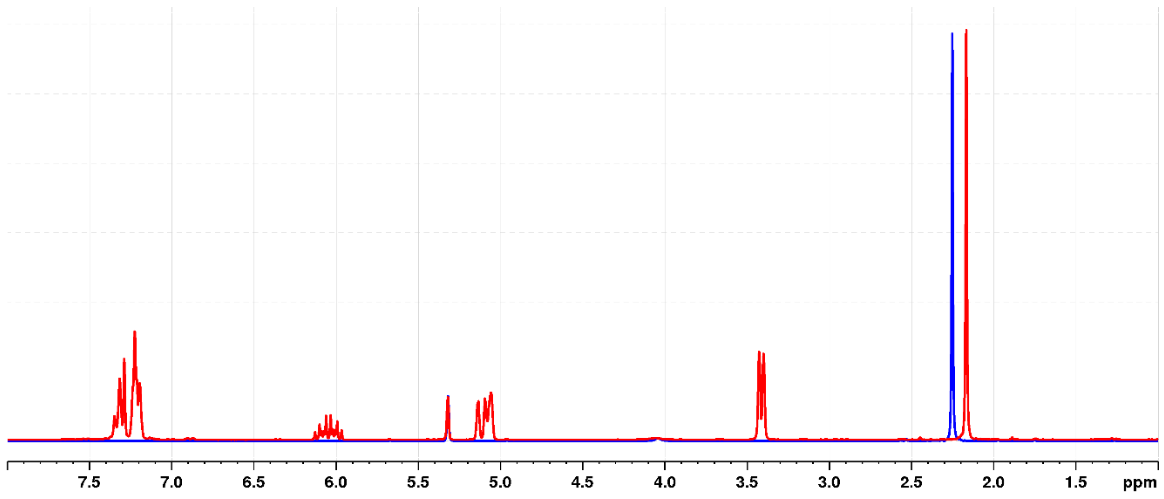

Figure S7. ^{1}H-NMR Spectra of freshly prepared [BiMe$_2$(SbF$_6$)] (blue) and the reaction mixture after allylbenzene was added to the above NMR sample (red).

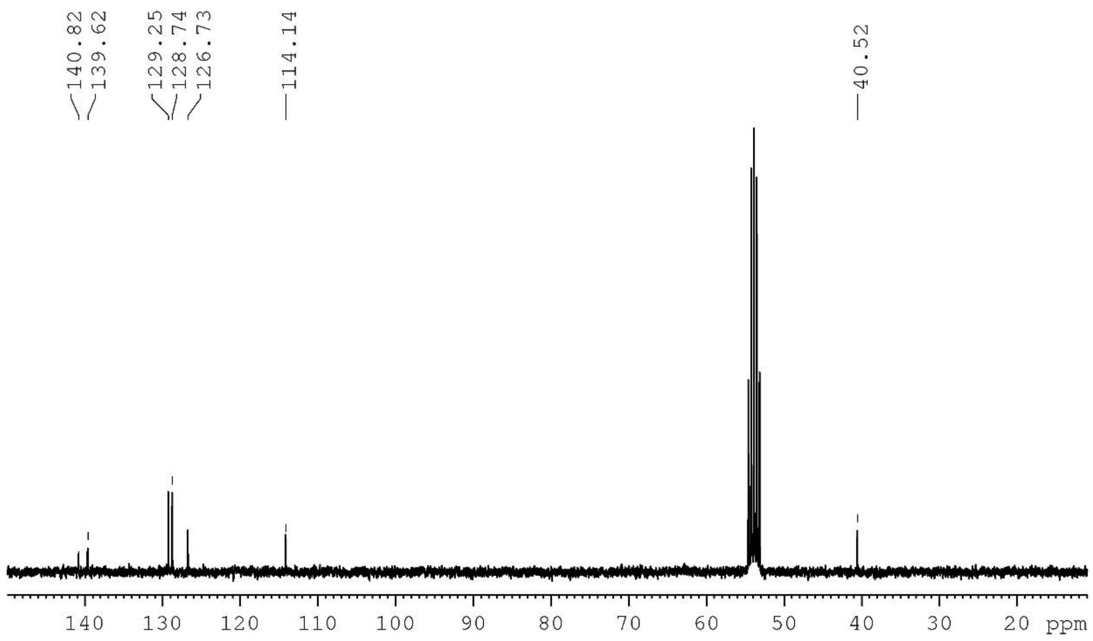

Figure S8. ^{13}C-NMR spectrum of the reaction mixture of [BiMe$_2$(SbF$_6$)] and allylbenzene.

1,4-Pentadiene (in the absence of a bismuth compound)

^{1}H-NMR (300 MHz, CD$_2$Cl$_2$): δ = 2.81 (t, 2 H, ${}^{3}J_{HH}$=5.78 Hz, alkyl-C*H*$_2$), 5.02 (m, 4 H, olefin-C*H*$_2$), 5.85 (m, 2H, C*H*) ppm.
^{13}C-NMR (75 MHz, CD$_2$Cl$_2$): δ = 38.24 (s, alkyl-*C*H$_2$), 115.51 (s, olefin-*C*H$_2$), 136.96 (s, *C*H) ppm.

1,4-Pentadiene (after reaction)

^{1}H-NMR (300 MHz, CD$_2$Cl$_2$): δ = 2.18 (s, 6 H, Bi-C*H*$_3$) 2.84 (t, 2 H, alkyl-C*H*$_2$), 5.07 (m, 4 H, olefin-C*H*$_2$), 5.90 (m, 2H, C*H*) ppm.
^{13}C-NMR (75 MHz, CD$_2$Cl$_2$): δ = 38.27 (s, alkyl-*C*H$_2$), 58.94 (s, Bi-*C*, detected *via* ^{1}H-^{13}C-HSQC spectrum), 115.23 (s, terminal *C*H$_2$), 137.39 (s, *C*H) ppm.

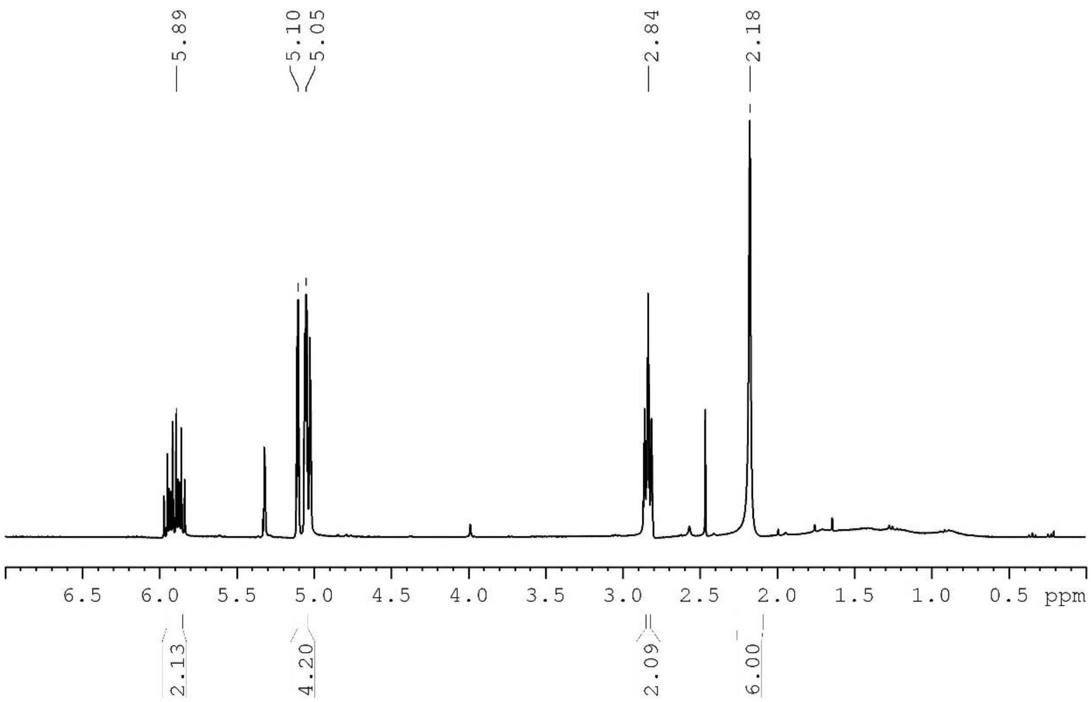

Figure S9. ^{1}H-NMR spectrum of the reaction mixture of [$BiMe_2(SbF_6)$] and pentadiene.

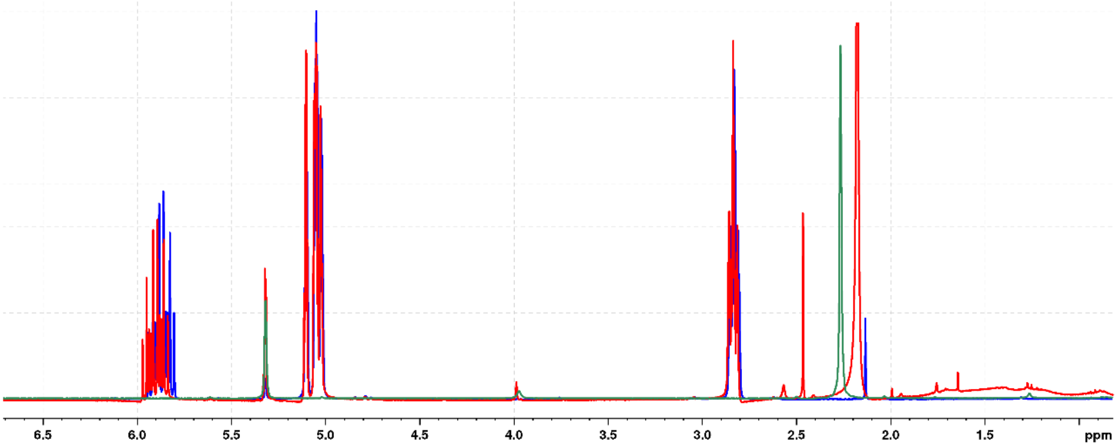

Figure S10. Stacked display of ^{1}H-NMR spectra of the reaction mixture and starting materials: green: [$BiMe_2(SbF_6)$], blue: pentadiene, red: reaction mixture.

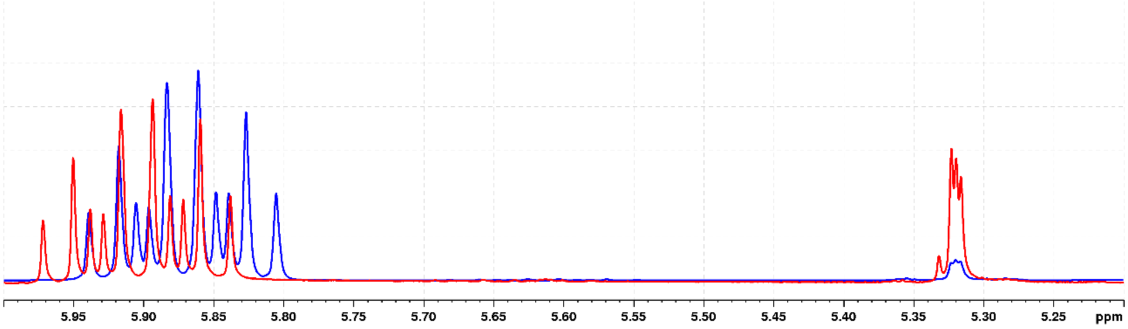

Figure S11. Stacked display of ^{1}H-NMR spectra of the region between 5 and 6 ppm: green: [$BiMe_2(SbF_6)$], blue: pentadiene, red: reaction mixture.

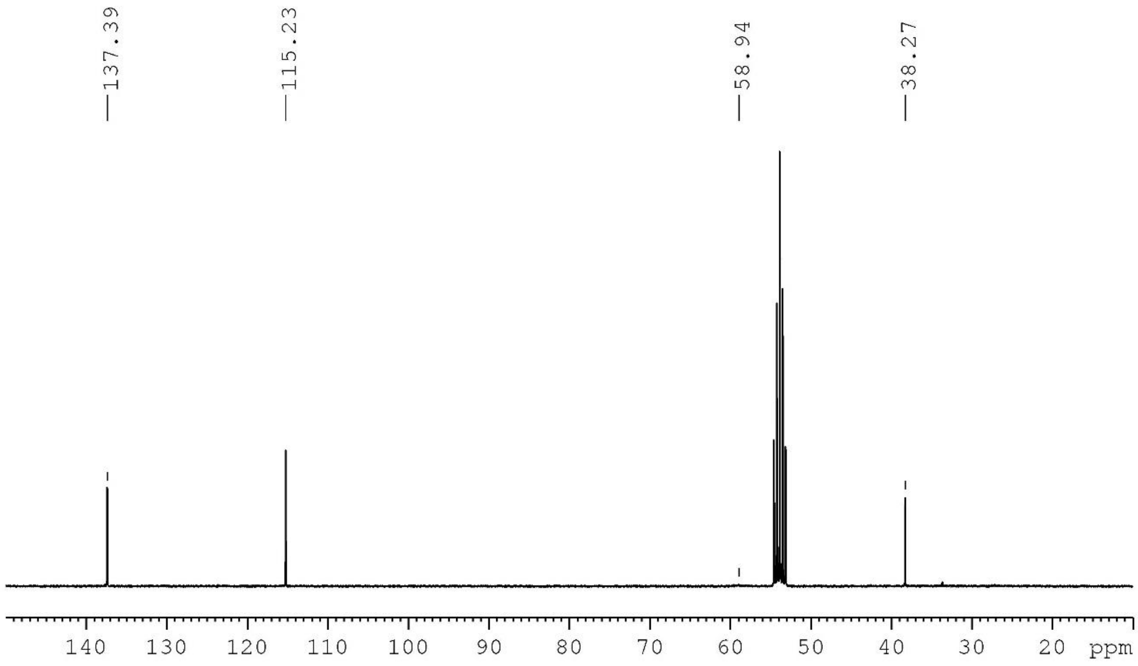

Figure S12. ^{13}C-NMR spectrum of the reaction mixture of [$BiMe_2(SbF_6)$] and pentadiene.

Observations:

- Change in NMR chemical shift for the olefin is bigger when there is less olefin present (very likely due to dynamic ligand exchange in solution).
- Cyclopentene: NMR data clearly indicate the interaction of the olefin with the bismuth compound in solution.
- Allylbenzene and 1,4-pentadiene: ^{1}H-NMR spectra show only minor changes in the chemical shifts of the olefins, slightly more pronounced changes in chemical shift are detected for the $BiMe_2$ unit. That might be due to a rapid equilibrium between i) bound and unbound olefin and ii) the olefin or the aryl unit (in case of allylbenzene, as verified by DFT calculations) or one and the other olefin unit (in case of 1,4-pentadiene) interacting with the bismuth center.

IR Spectroscopy

General procedure for samples analyzed by infrared spectroscopy:
[$BiMe_2(SbF_6)$] (10 mg, 0.021 mmol, 1 eq) was dissolved in CH_2Cl_2 (0.5 mL). The respective olefin (1 eq) was added *via* microliter syringe. The reaction mixture was analyzed *in situ* via IR-spectroscopy.

Olefins used as substrates:

- Cyclopentene (1.9 µL, 0.021 mmol, 1 eq.)
- Allylbenzene (2.3 µL, 0.021 mmol, 1 eq.)
- 1,4-Pentadiene (2.2 µL, 0.021 mmol, 1 eq.)

Cyclopentene

Pure:

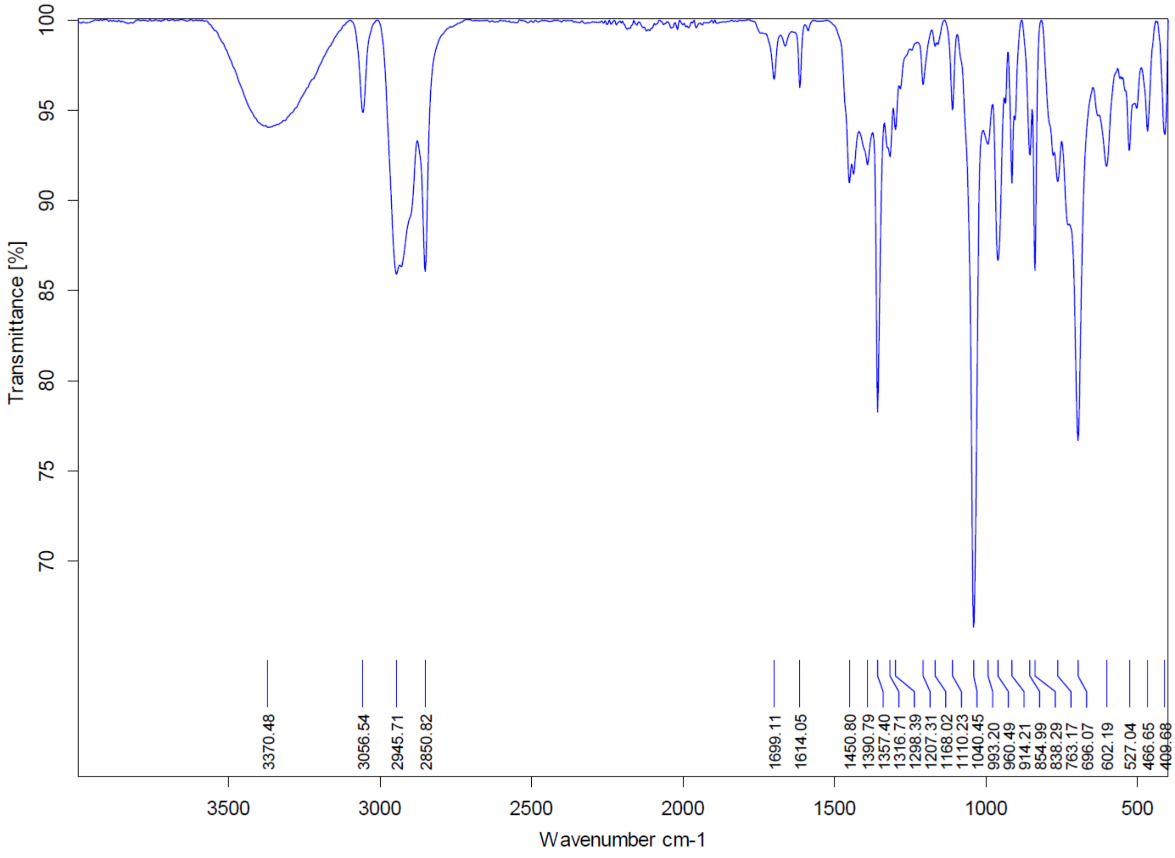

After Reaction:

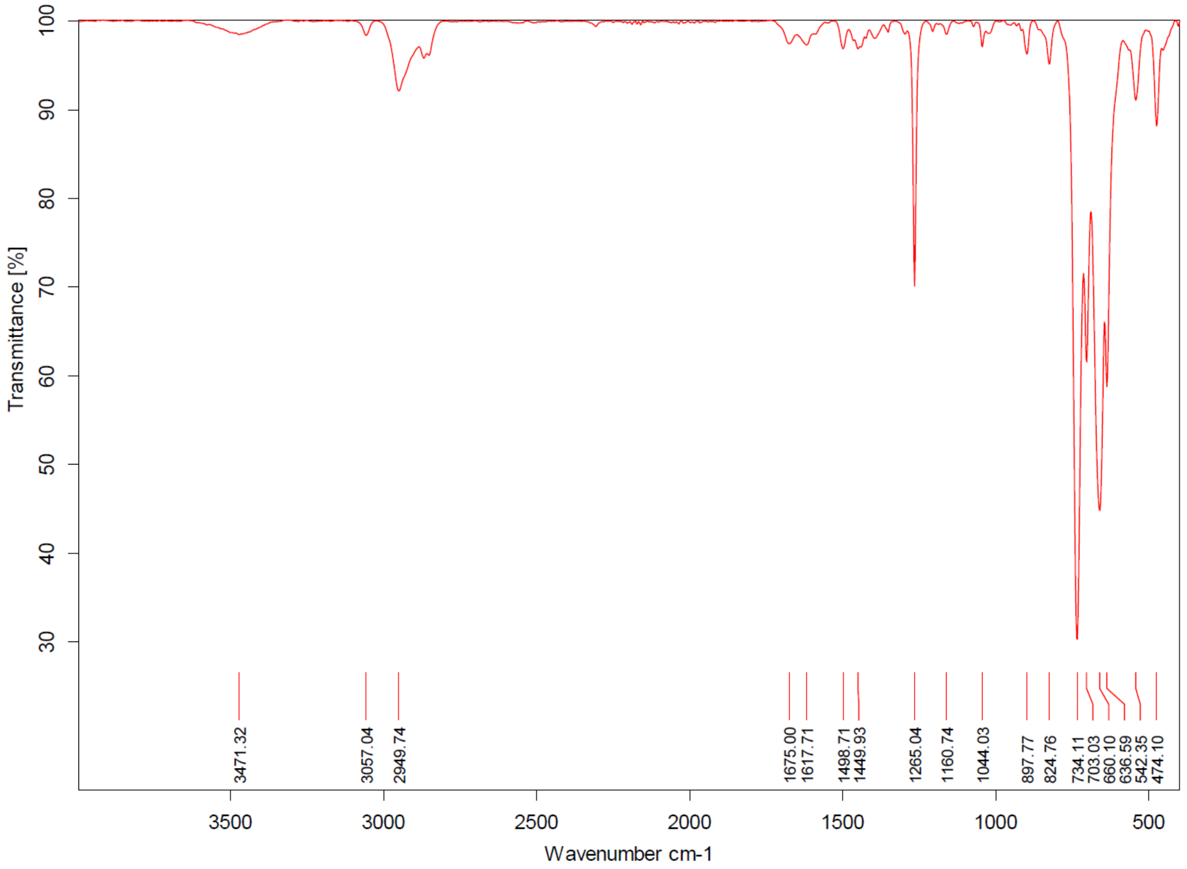

Shift of the olefin signal from 1699 cm^{-1} to 1675 cm^{-1}

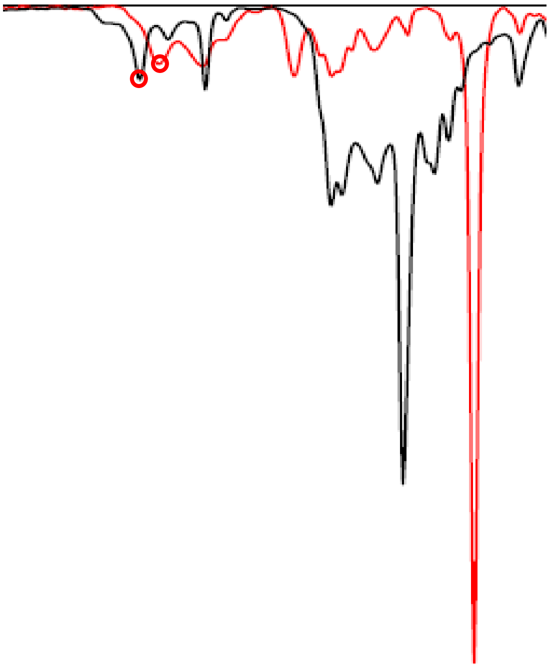

Allylbenzene

Pure:

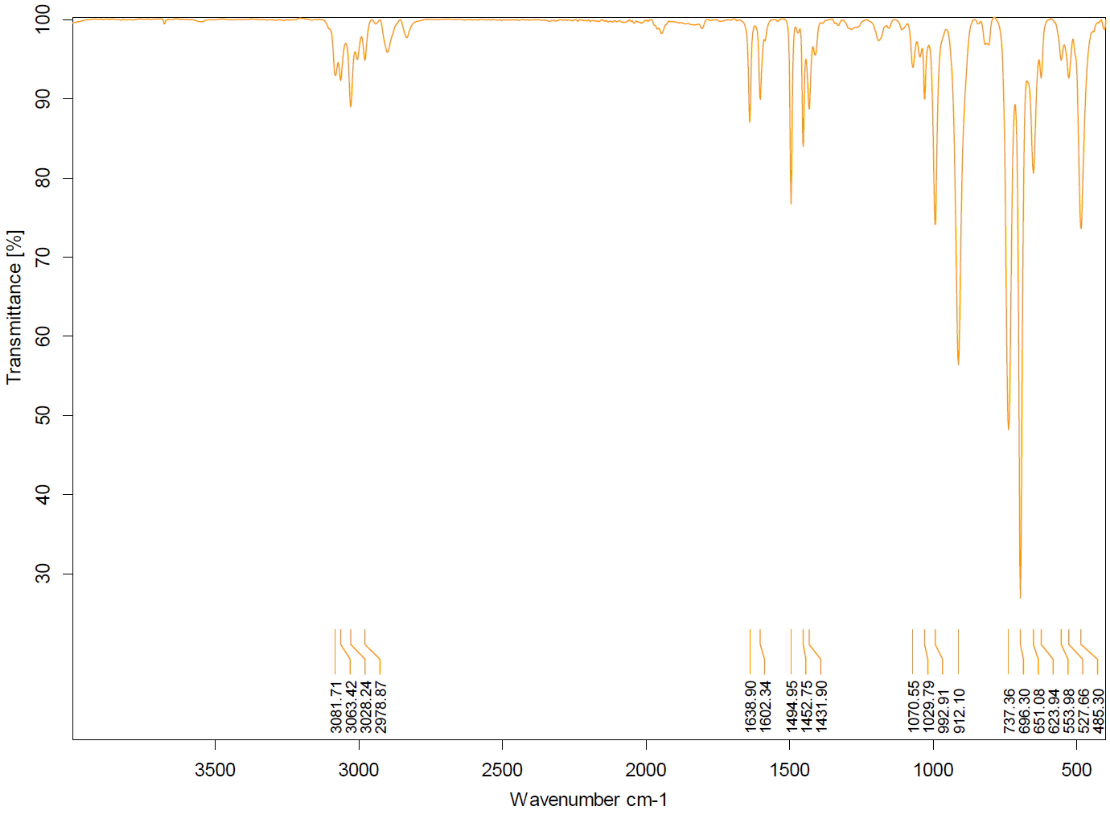

After Reaction:

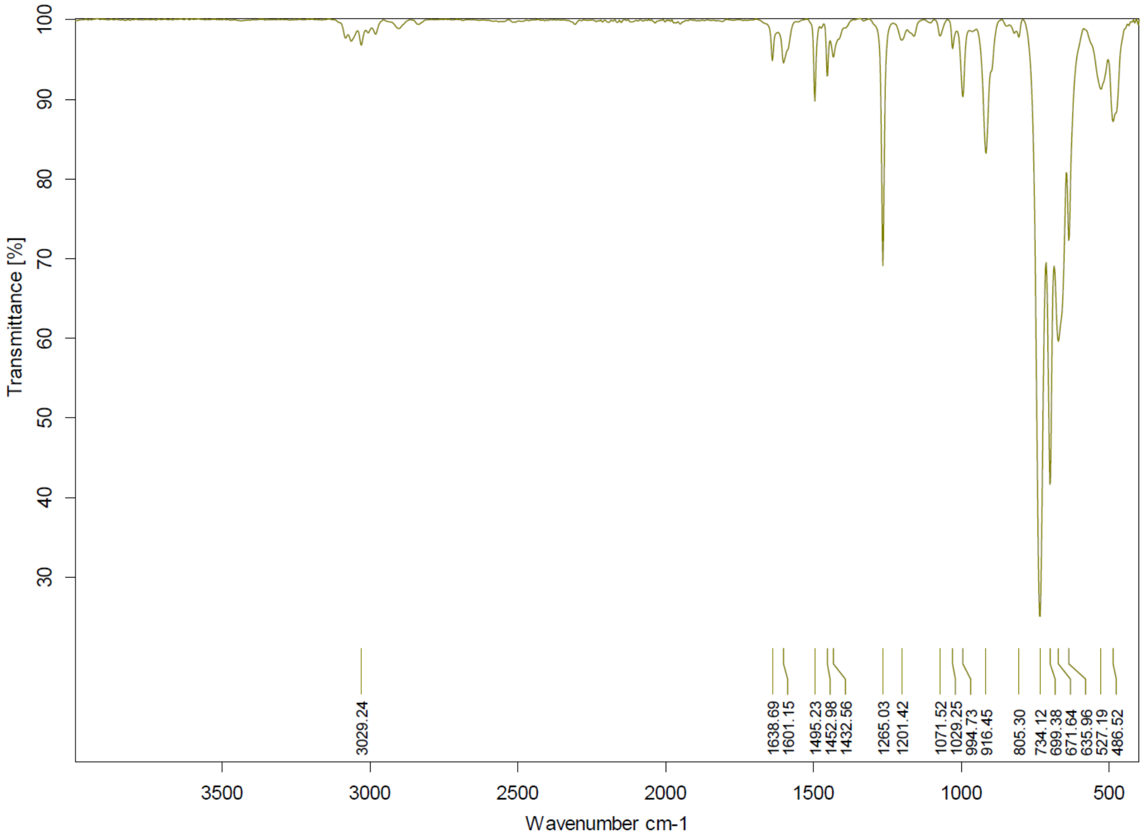

No significant shift of the olefin signal (1639 cm^{-1} and 1601 cm^{-1})

1,4-Pentadiene

Pure:

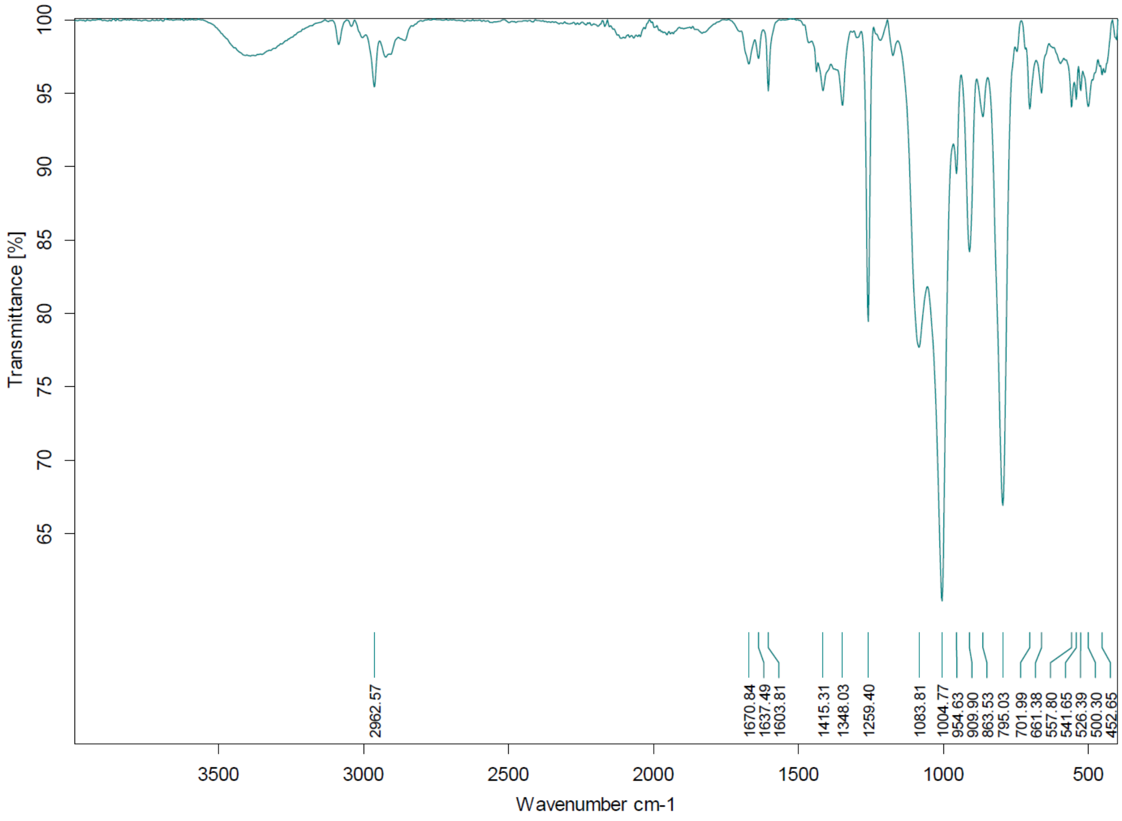

After Reaction:

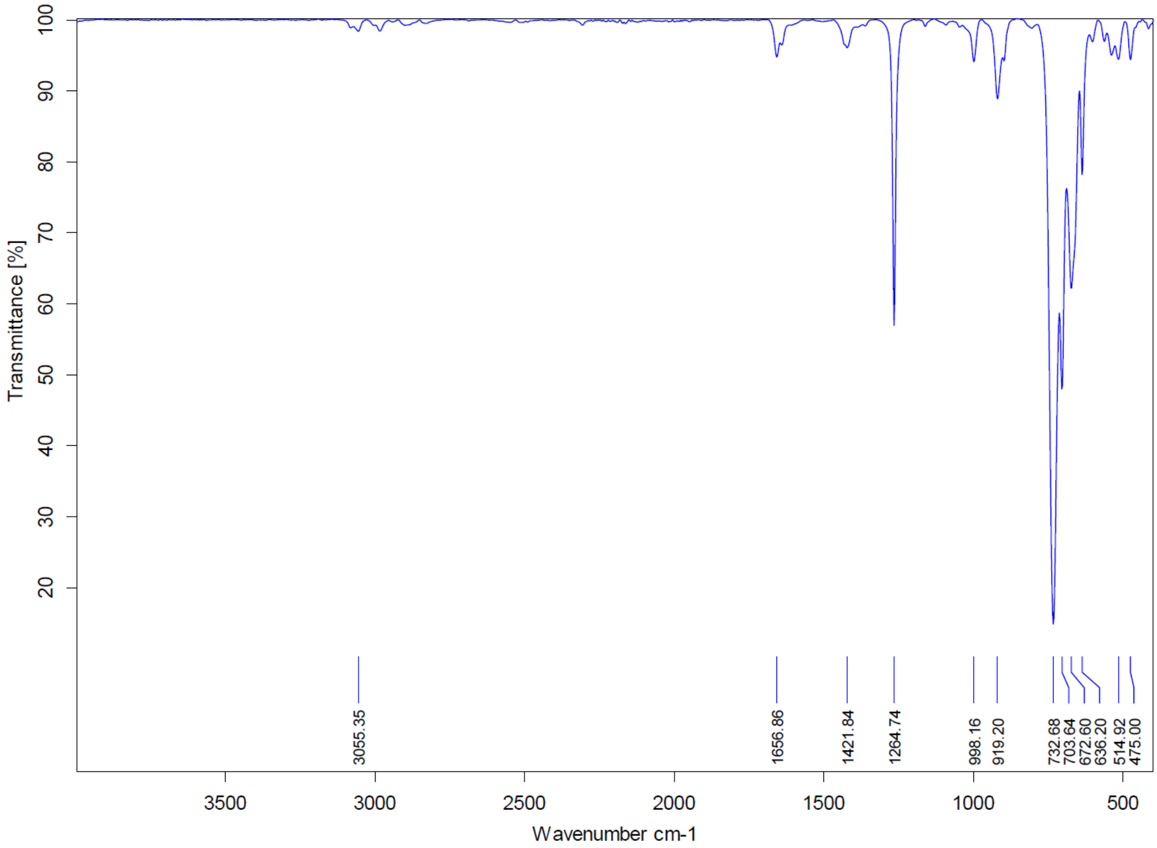

Shift of the olefin signal from 1670 cm^{-1} to 1656 cm^{-1}

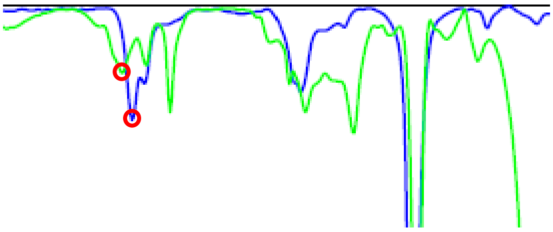

Mass spectrometry

<u>General procedure for samples analyzed by mass spectrometry:</u>
[$BiMe_2(SbF_6)$] (25 mg, 0.053 mmol, 1 eq) was dissolved in CH_2Cl_2 (2 mL). The respective olefin (1 eq) was added *via* microliter syringe. A small portion of the reaction mixture was further diluted with CH_2Cl_2 and analyzed *via* mass spectrometry.

<u>Olefins used as substrates:</u>

- Cyclopentene (4.7 µL, 0.053 mmol, 1 eq.)
- 1,4-Pentadiene (5.4 µL, 0.053 mmol, 1 eq.)

Cyclopentene: (ESI-MS)

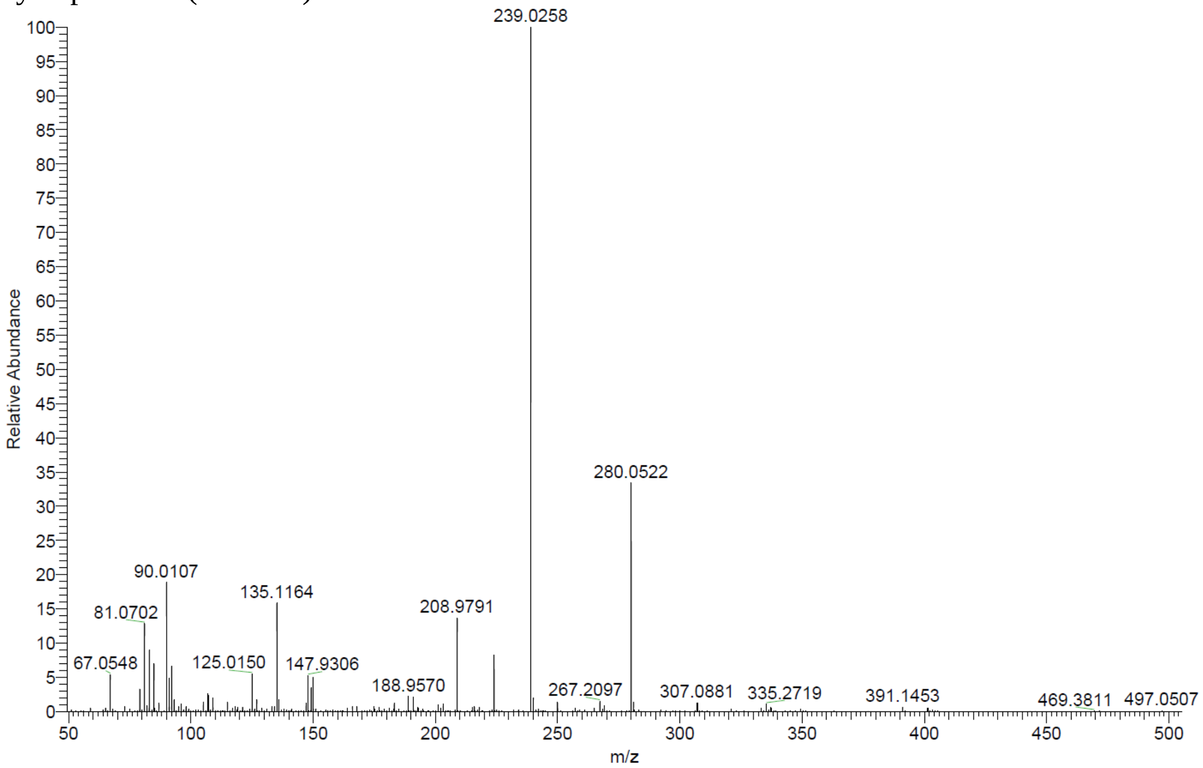

Figure S13. ESI-MS of the mixture of [$BiMe_2(SbF_6)$] and cyclopentene.

m/z =208.9791 (Bi^+: *m/z* = 208.9799)
m/z =239.0258 ($BiMe_2^+$: *m/z* = 239.0269)
m/z = 307.0882 ($BiC_7H_{14}^+$: *m/z* = 307.0894)

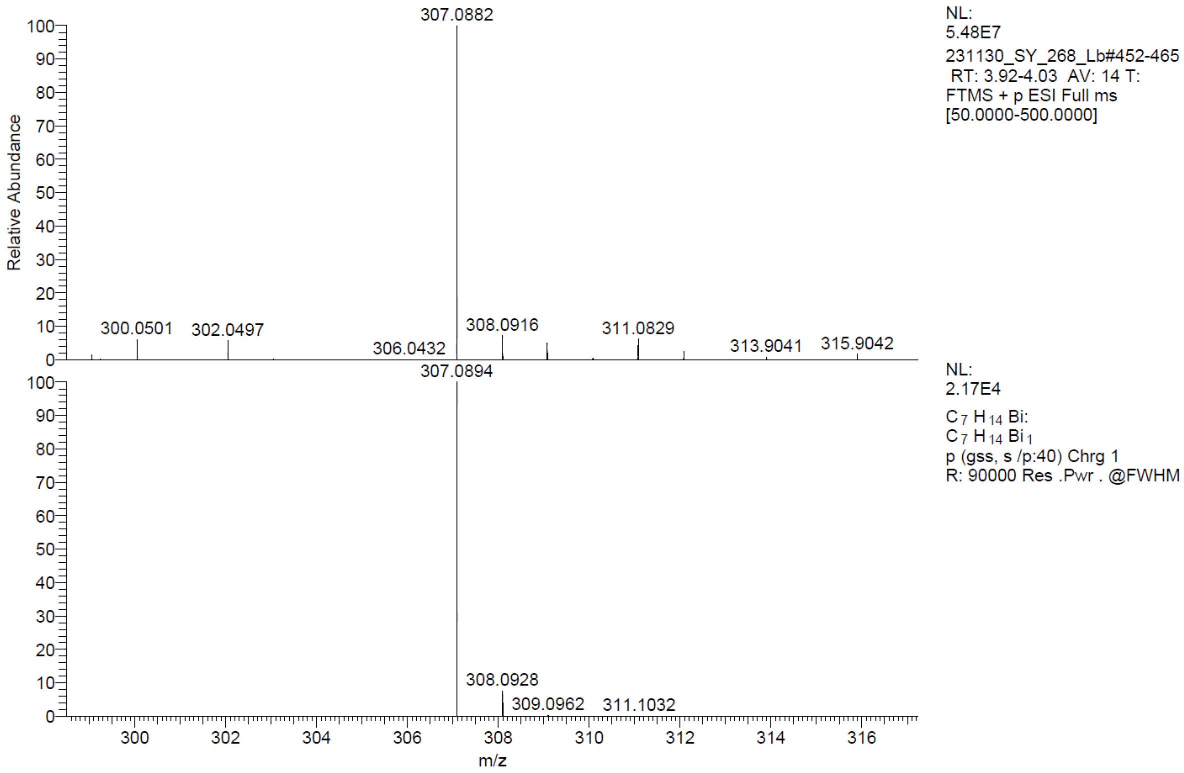

Figure S14. Top: enlarged part of the ESI-MS spectrum. Bottom: calculated isotope pattern of $C_7H_{14}Bi^+$.

Part II: Analysis with [$BiPh_2(SbF_6)$]

NMR Spectroscopy

General procedure for the in-situ-NMR spectroscopic analysis of [$BiPh_2SbF_6$] in the presence of an olefin:

[$BiPh_2(SbF_6)$] (25 mg, 0.042 mmol, 1 eq) was dissolved in CD_2Cl_2 (0.7 mL). The respective olefin (1 eq) was added with a microliter syringe. The samples were analyzed by ^{1}H- and ^{13}C-NMR spectroscopy. An interaction is indicated by a change in the respective NMR chemical shifts of both compounds, without a change in multiplicity.

Olefins used as substrates:

- Cyclopentene (3.7 µL, 0.042 mmol, 1 eq.): coordination (NMR shifts in next section)
- Allylbenzene (5.5 µL, 0.042 mmol, 1 eq.): coordination (NMR shifts in next section)
- 1,4-Pentadiene (4.3 µL, 0.042 mmol, 1 eq.): coordination (NMR shifts in next section)

NMR-Spectra:

[$BiPh_2(SbF_6)$]

^{1}H NMR (400 MHz, CD_2Cl_2): $\delta = 7.63$ (t, 2 H, $^3J_{HH} = 7.3$ Hz, *para*-C_6H_5), 8.08 (t, 4 H, $^3J_{HH} = 7.4$ Hz, *meta*-C_6H_5), 8.55 (d, 4 H, $^3J_{HH} = 7.5$ Hz, *ortho*-C_6H_5) ppm.

^{13}C NMR (101 MHz, CD_2Cl_2): δ = 131.34 (s, *para*-C_6H_5), 134.79 (s, *meta*-C_6H_5), 138.76 (s, *ortho*-C_6H_5), 214.76 (s, *ipso*-C_6H_5, detected by 2D-^1H-^{13}C-HMBC experiments) ppm.
(*Note*: This compound gradually decomposes to benzene in CD_2Cl_2, details of its characterization are included in section "Synthesis of cationic bismuth complexes".)

Cyclopentene (in the absence of a bismuth compound)

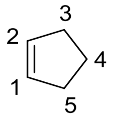

^1H-NMR (300 MHz, CD_2Cl_2): δ = 1.82 (quint, 2 H, $^3J_{HH}$=7.50 Hz, H-4), 2.31 (t, 4 H, $^3J_{HH}$=7.43 Hz, H-3, 5), 5.74 (s, 2 H, H-1,2) ppm.
^{13}C-NMR (75 MHz, CD_2Cl_2): δ = 23.19 (s, C-4), 32.80 (s, C-3, 5), 130.99 (s, C-1, 2) ppm.

Cyclopentene (after reaction)

^1H-NMR (300 MHz, CD_2Cl_2): δ = 1.82 (quint, 2 H, $^3J_{HH}$=7.37 Hz, H-4), 2.37 (t, 4 H, $^3J_{HH}$=7.40 Hz, H-3, 5), 5.99 (s, 2H, H-1, 2) 7.60 (t, 2H, $^3J_{HH}$ = 7.49 Hz, *para*-C_6H_5), 8.02 (t, 4H, $^3J_{HH}$ = 7.66 Hz, *meta*-C_6H_5), 8.49 (d, 4H, $^3J_{HH}$ = 7.16 Hz, *ortho*-C_6H_5) ppm.
^{13}C-NMR (75 MHz, CD_2Cl_2): δ = 23.50 (s, C-4), 33.16 (s, C-3, 5), 131.01 (s, *para*-C_6H_5), 131.86 (s, C-1, 2), 134.37 (s, *meta*-C_6H_5), 138.53 (s, *ortho*-C_6H_5), 205.82 (s, *ipso*-C_6H_5, detected by 2D-^1H-^{13}C-HMBC experiments) ppm.

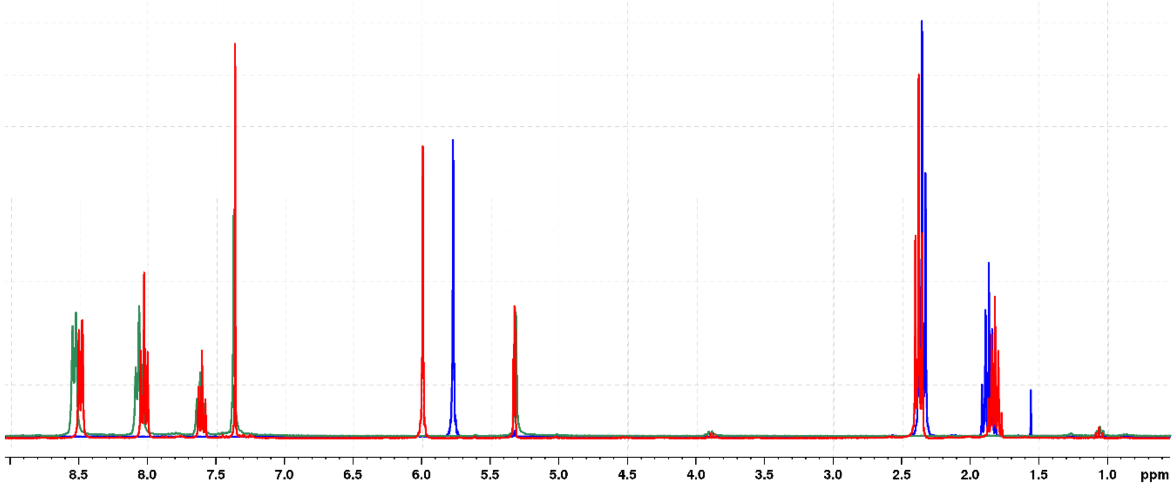

Figure S15. Stacked display of ^1H-NMR spectra of reaction mixture and starting materials: green: [$BiPh_2(SbF_6)$], blue: cyclopentene, red: reaction mixture.

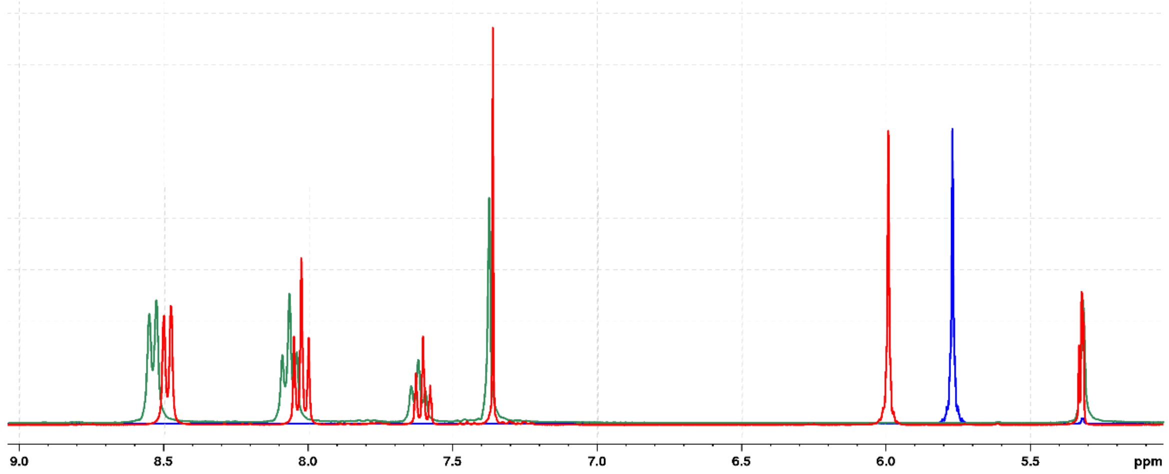

Figure S16. Stacked display of ^{1}H-NMR spectra of the region between 5 and 9 ppm: green: [$BiPh_2(SbF_6)$], blue: cyclopentene, red: reaction mixture.

Note: A change in chemical shifts of the olefin was maintained, when [$BiPh_2(SbF_6)$] and TMSOTf were present in the reaction mixture. No change in the ^{1}H NMR chemical shifts of the olefin was observed in the absence of bismuth component, but with equimolar amounts of TMSOTf or $BF_3{\cdot}OEt_2$ (solvent: CD_2Cl_2).

Allylbenzene (in the absence of a bismuth compound)

^{1}H-NMR (300 MHz, CD_2Cl_2): δ = 3.42 (d, 2 H, $^{3}J_{HH}$=6.64 Hz, alkyl-CH_2), 5.11 (m, 2 H, olefin-CH_2), 6.01 (m, 1 H, olefin-C*H*), 7.23 (m, 3 H, *meta*-, *para*-C*H*, $^{3}J_{HH}$=6.90 Hz), 7.32 (t, 2 H, $^{3}J_{HH}$=7.20 Hz, *ortho*-C*H*) ppm.
^{13}C-NMR (75 MHz, CD_2Cl_2): δ = 40.62 (s, alkyl-CH_2), 115.85 (s, olefin-CH_2), 126.44 (s, *para*-*C*H), 128.98 (s, *ortho*-*C*H), 129.01 (s, *meta*-*C*H),138.10 (s, olefin-*C*H), 140.67 (s, *ipso*-*C*) ppm.

Allylbenzene (after reaction)

^{1}H-NMR (300 MHz, CD_2Cl_2): δ = 3.38 (d, 2 H, $^{3}J_{HH}$=6.83 Hz, alkyl-CH_2,), 5.05 (m, 2 H, olefin-CH_2), 6.10 (m, 1 H, olefin-C*H*), 7.18 (m, 3 H, *meta*-, *para*-C*H*), 7.33 (t, 2 H, $^{3}J_{HH}$=7.42 Hz, *ortho*-C*H*,), 7.57 (s, br, 2H, Bi-*para*-C_6H_5), 7.98 (s, br, 4H, Bi-*meta*-C_6H_5), 8.42 (s, br, 4H, Bi-*ortho*-C_6H_5) ppm.
^{13}C-NMR (75 MHz, CD_2Cl_2): δ =40.48 (s, alkyl-CH_2), 113.32 (s, olefin-CH_2), 126.84 (s, *para*-*C*H), 128.72 (s, *ortho*-*C*H), 129.26 (s, *meta*-*C*H), 130.77 (s, Bi-*para*-C_6H_5), 134.22 (s, Bi-*meta*-C_6H_5), 138.40 (s, Bi-*ortho*-C_6H_5), 139.37 (s, olefin-*C*H), 140.61 (s, *ipso*-*C*)) 208.93 (s, Bi-*ipso*-C_6H_5, detected by 2D-^{1}H-^{13}C-HMBC experiments) ppm.

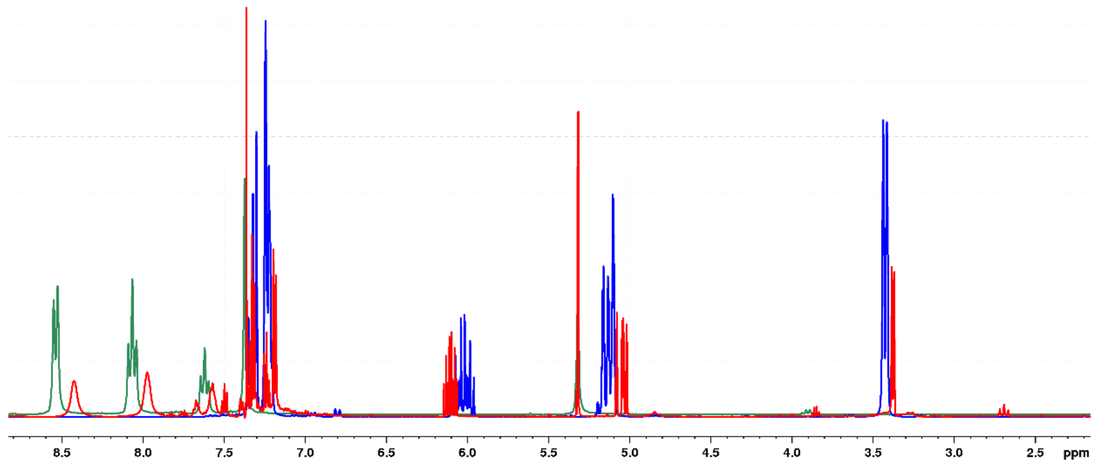

Figure S17. Stacked display of ^{1}H-NMR spectra of reaction mixture and starting materials: green: [$BiPh_2(SbF_6)$], blue: allylbenzene, red: reaction mixture.

1,4-Pentadiene (in the absence of a bismuth compound)

^{1}H-NMR (300 MHz, CD_2Cl_2): δ = 2.81 (t, 2 H, $^3J_{HH}$=5.78 Hz, alkyl-C*H*$_2$), 5.02 (m, 4 H, olefin-C*H*$_2$), 5.85 (m, 2H, C*H*) ppm.
^{13}C-NMR (75 MHz, CD_2Cl_2): δ = 38.24 (s, alkyl-*C*H$_2$), 115.51 (s, olefin-*C*H$_2$), 136.96 (s, *C*H) ppm.

1,4-Pentadiene (after reaction)

^{1}H-NMR (300 MHz, CD_2Cl_2): δ = 2.87 (t, 2 H, $^3J_{HH}$=6.58 Hz, alkyl-C*H*$_2$), 5.13 (m, 4 H, olefin-C*H*$_2$), 6.04 (m, 2H, C*H*), 7.59 (t, 2H, $^3J_{HH}$ = 7.47 Hz, *para*-C_6H_5), 8.01 (t, 4H, $^3J_{HH}$ = 7.62 Hz, *meta*-C_6H_5), 8.48 (d, 4H, $^3J_{HH}$ = 7.42 Hz, *ortho*-C_6H_5) ppm.
^{13}C-NMR (75 MHz, CD_2Cl_2): δ = 38.31 (s, alkyl-*C*H$_2$), 113.54 (s, terminal *C*H$_2$), 131.04 (s, *para*-C_6H_5), 134.40 (s, *meta*-C_6H_5), 137.77 (s, *C*H), 138.46 (s, *ortho*-C_6H_5), 207.57 (s, *ipso*-C_6H_5, detected by 2D-^{1}H-^{13}C-HMBC experiments) ppm.

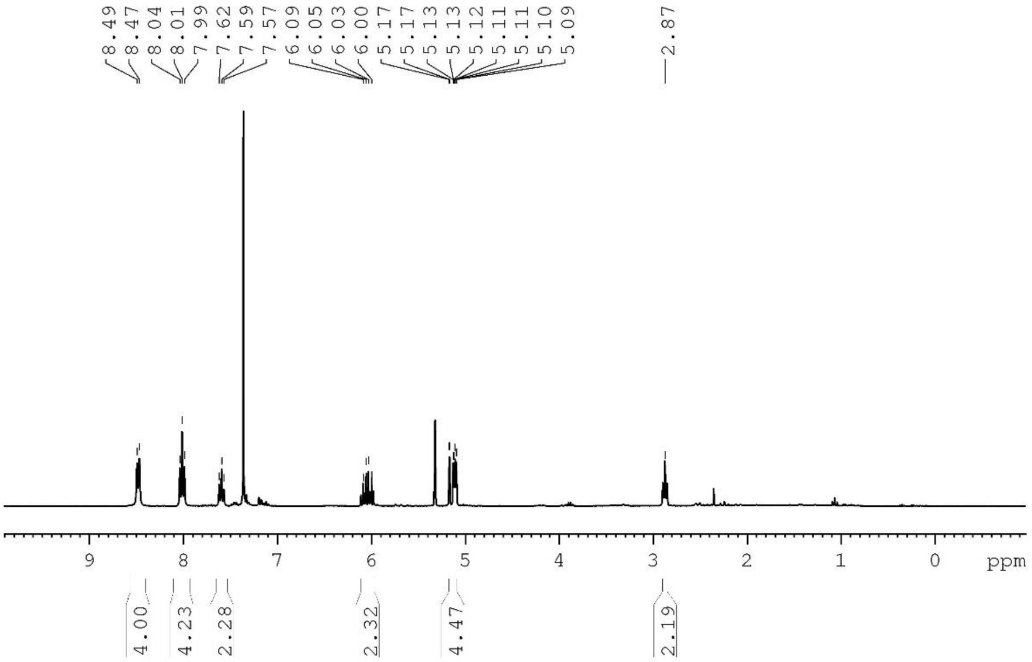

Figure S18. ^{1}H-NMR spectrum of the reaction mixture of [$BiPh_2(SbF_6)$] and pentadiene.

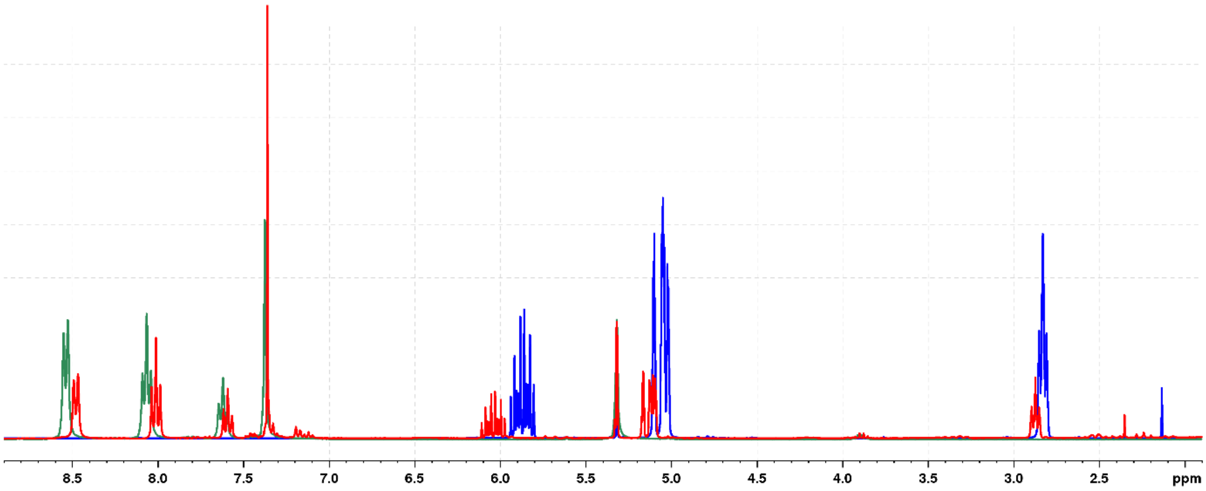

Figure S19. Stacked display of ^{1}H-NMR spectra of the reaction mixture and starting materials: blue: pentadiene, green: $[BiPh_2(SbF_6)]$, red: reaction mixture.

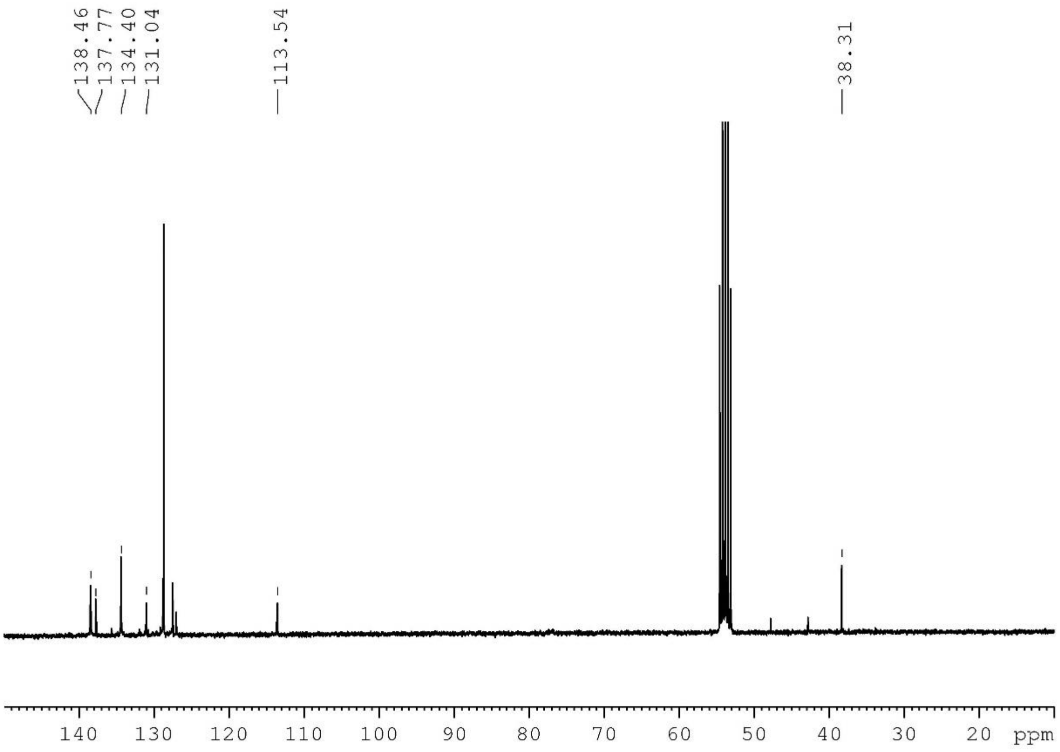

Figure S20. ^{13}C-NMR spectrum of the reaction mixture of $[BiPh_2(SbF_6)]$ and pentadiene.

Note: A change in chemical shifts of the olefin was maintained, when $[BiPh_2(SbF_6)]$ and TMSOTf were present in the reaction mixture. No change in the ^{1}H NMR chemical shifts of the olefin was observed in the absence of bismuth component, but with equimolar amounts of TMSOTf or $BF_3{\cdot}OEt_2$ (solvent: CD_2Cl_2).

IR Spectroscopy

General procedure for samples analyzed by infrared spectroscopy:

$[BiPh_2(SbF_6)]$ (10 mg, 0.021 mmol, 1 eq) was dissolved in CH_2Cl_2 (0.5 mL). The respective olefin (1 eq) was added *via* microliter syringe. The reaction mixture was analyzed *in situ* via IR-spectroscopy.

<u>Olefins used as substrates:</u>

- Cyclopentene (1.9 µL, 0.021 mmol, 1 eq.)
- Allylbenzene (2.3 µL, 0.021 mmol, 1 eq.)
- 1,4-Pentadiene (2.2 µL, 0.021 mmol, 1 eq.)

Cyclopentene
After Reaction:

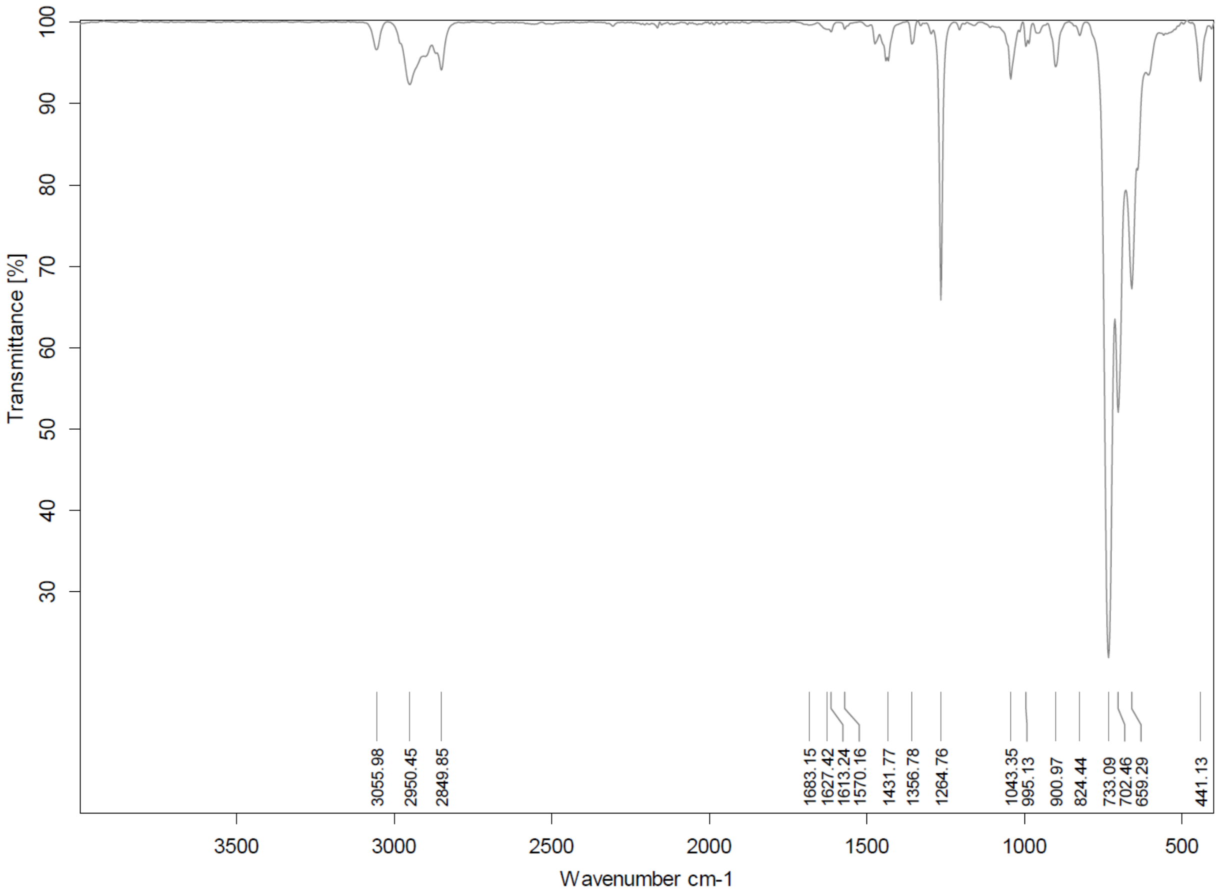

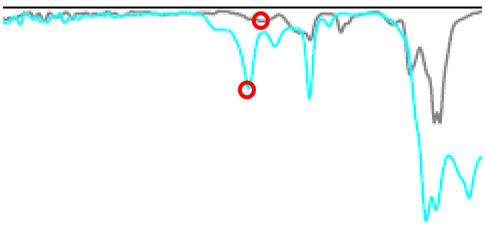

Shift of the olefin signal from 1699 cm^{-1} to 1683 cm^{-1}

Allylbenzene

After Reaction:

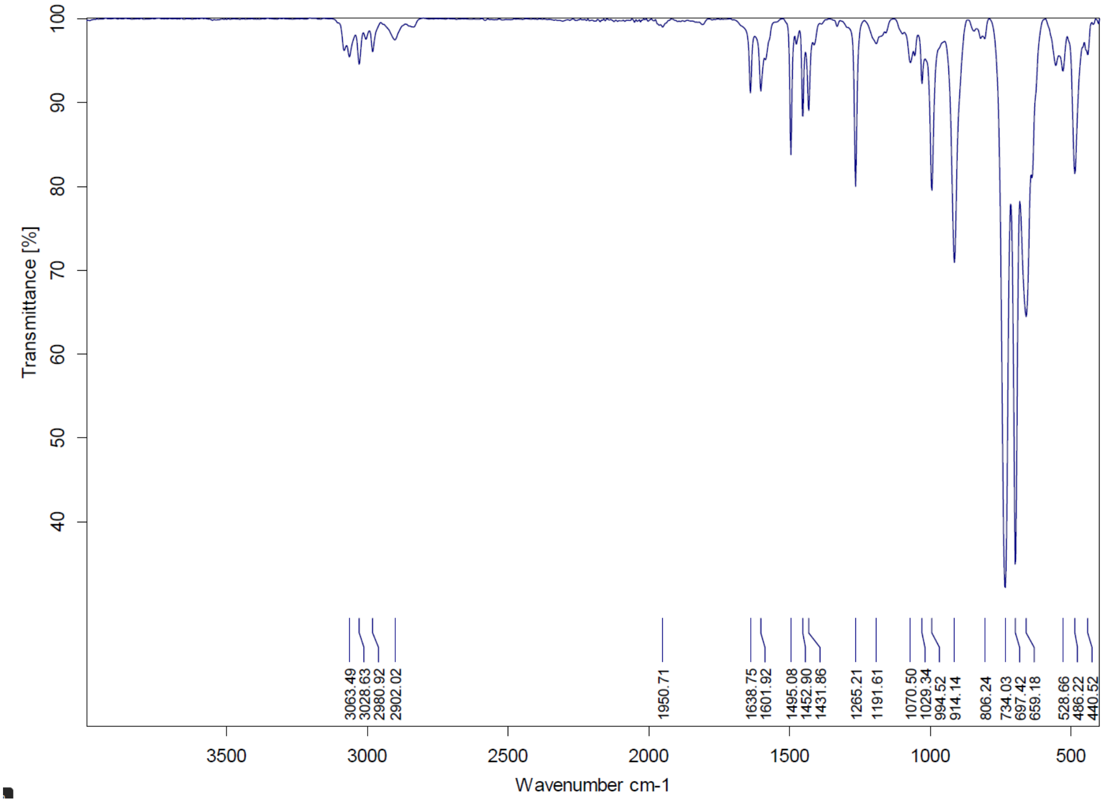

No significant shift of the olefin signal (1639 cm^{-1} and 1601 cm^{-1})

1,4-Pentadiene

After Reaction:

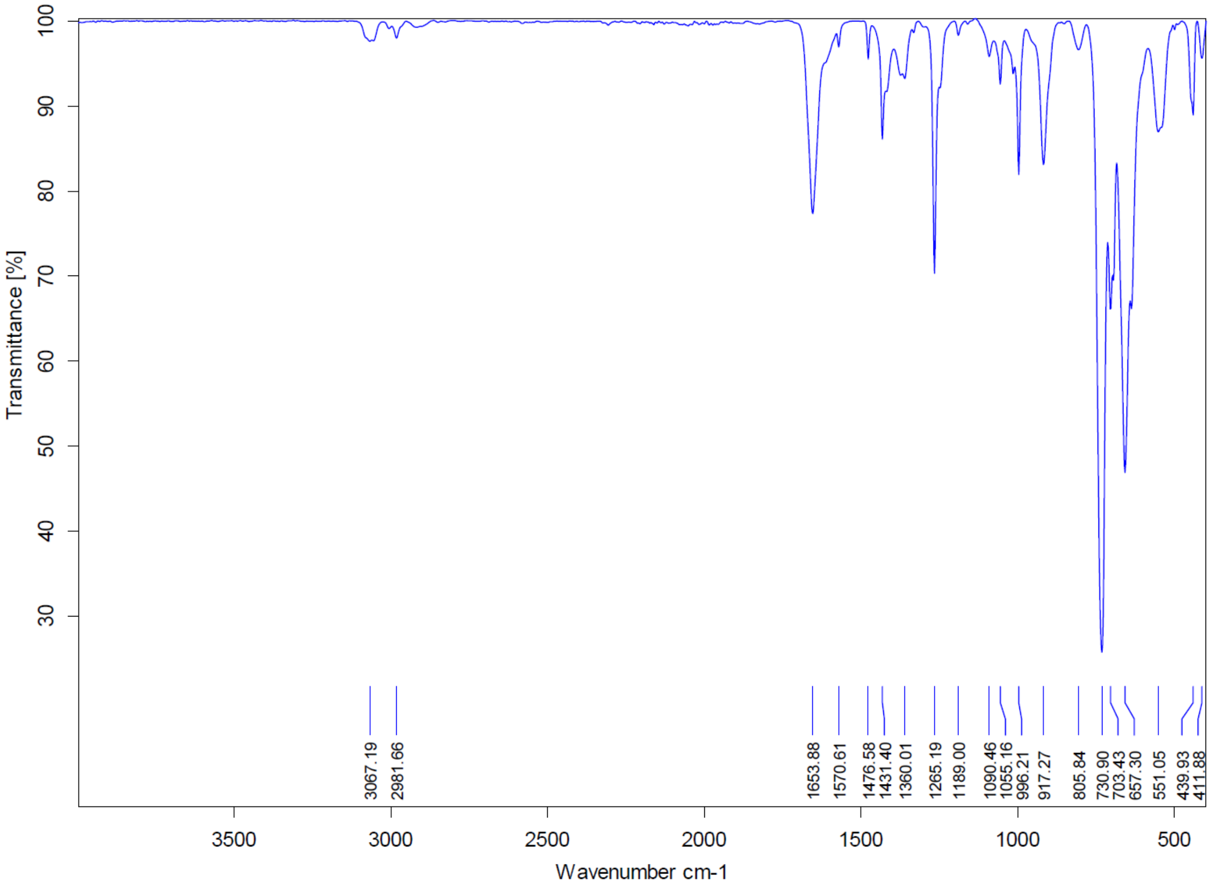

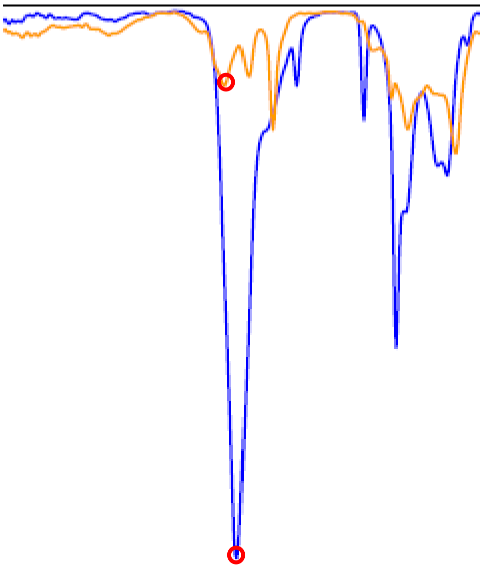

Shift of the olefin signal from 1670 cm^{-1} to 1653 cm^{-1}

Mass spectrometry

<u>General procedure for samples analyzed by mass spectrometry:</u>

[$BiPh_2(SbF_6)$] (25 mg, 0.053 mmol, 1 eq) was dissolved in CH_2Cl_2 (2 mL). Cyclopentene (4.7 µL, 0.053 mmol, 1 eq.) was added *via* microliter syringe. A small portion of the reaction mixture was further diluted with CH_2Cl_2 and analyzed *via* mass spectrometry.

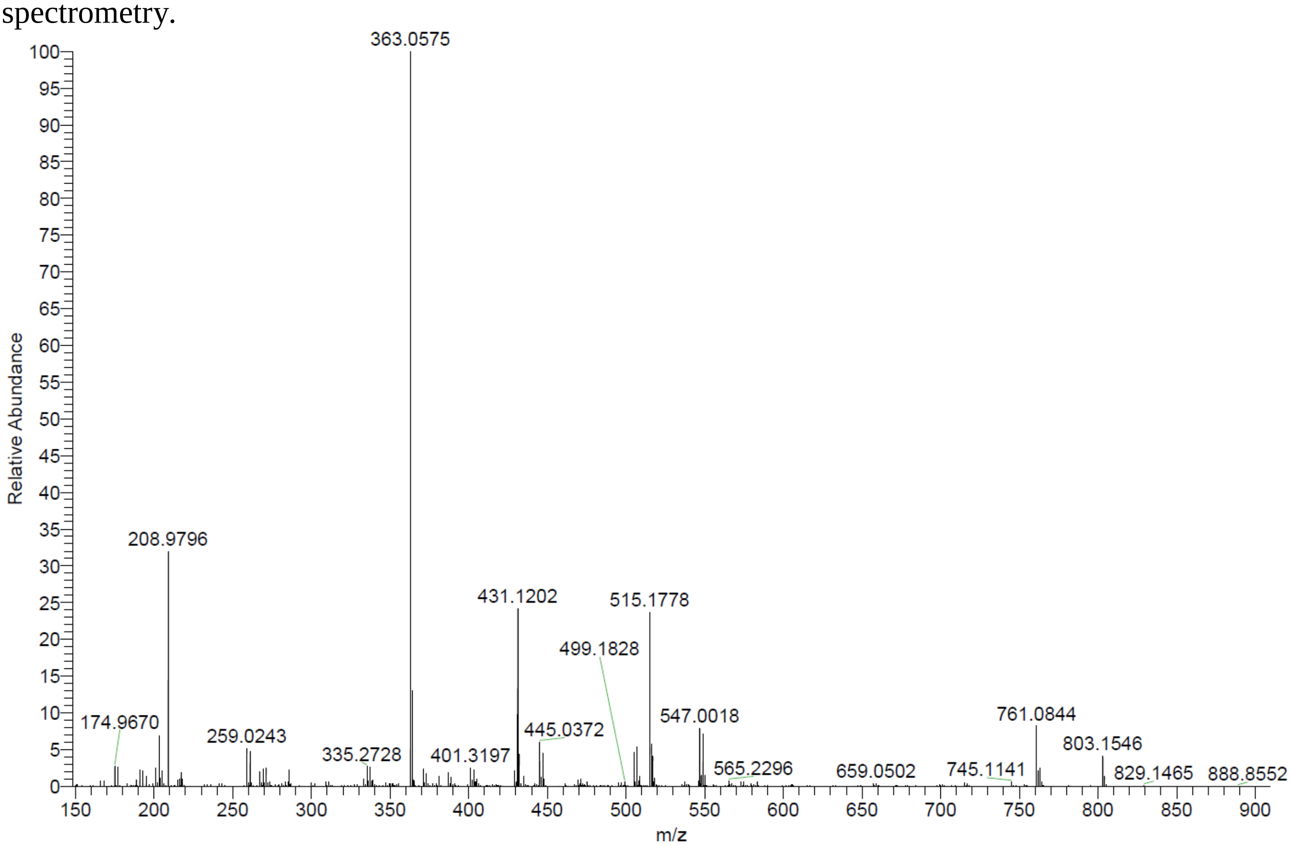

Figure S21. ESI-MS of the mixture of [$BiPh_2(SbF_6)$] and cyclopentene.

m/z =208.9791 (Bi^+: *m/z* = 208.9799)
m/z =363.0575 ($BiPh_2^+$: *m/z* = 363.0581)
m/z = 431.1202 ($BiC_{17}H_{18}^+$: *m/z* = 431.1207)

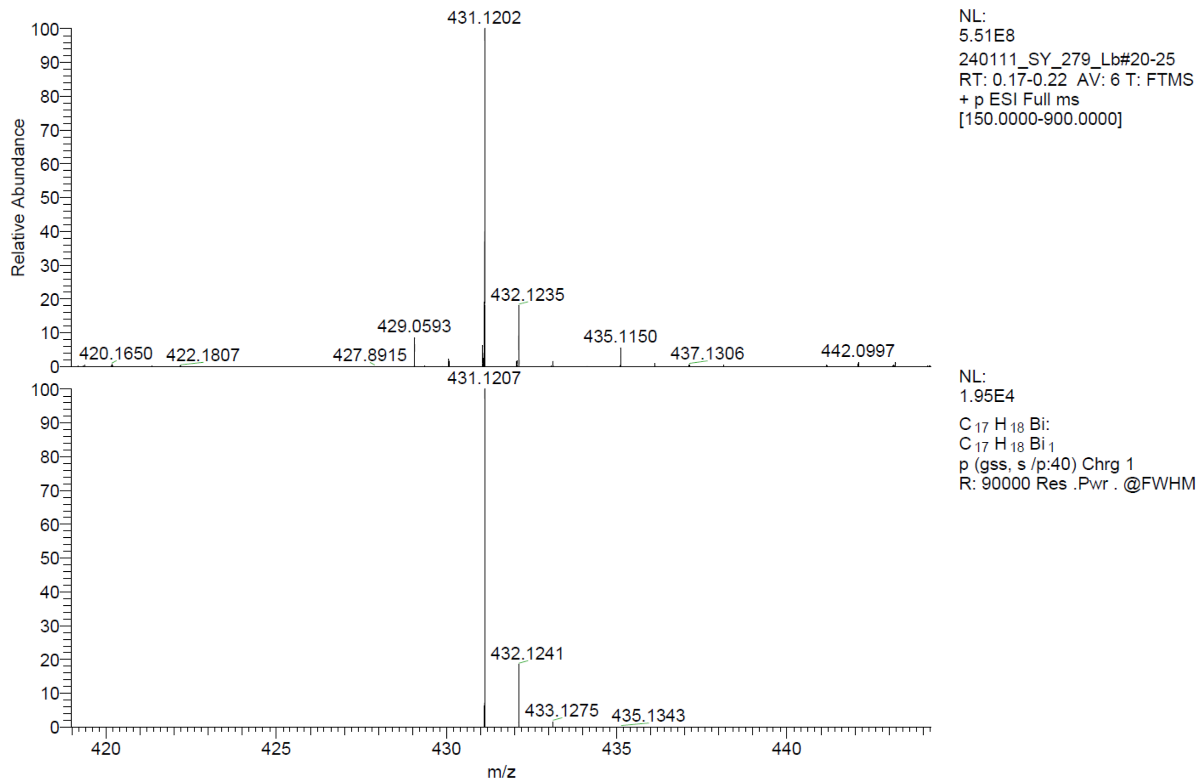

Figure S22. Top: enlarged part of the ESI-MS spectrum. Bottom: calculated isotope pattern of $C_{17}H_{18}Bi^{+}$.

Part III: Analysis with [$BiPh_2OTf$]

NMR Spectroscopy

General procedure for the in-situ-NMR spectroscopic analysis of [$BiPh_2(OTf)$] in the presence of an olefin:

[$BiPh_2(OTf)$] (25 mg, 0.049 mmol, 1 eq) was suspended in CD_2Cl_2 (0.7 mL). The respective olefin (1 eq) was added with a microliter syringe to give colorless suspensions. The samples were analyzed by ^{1}H- and ^{13}C-NMR spectroscopy. Due to the insolubility of [$BiPh_2(OTf)$] in non-coordinating solvents, only the free olefins were observed in the NMR spectra.

Olefins used as substrates:

- Cyclopentene (4.3 µL, 0.049 mmol, 1 eq.)
- Allylbenzene (6.0 µL, 0.049 mmol, 1 eq.)
- 1,4-Pentadiene (5.0 µL, 0.049 mmol, 1 eq.)

IR Spectroscopy

General procedure for samples analyzed by infrared spectroscopy:

[$BiPh_2(OTf)$] (10 mg, 0.020 mmol, 1 eq) was suspended in CH_2Cl_2 (0.5 mL). The respective olefin (1 eq) was added *via* microliter syringe to give a colorless suspension. The reaction mixture was analyzed *in situ* via IR-spectroscopy.

Olefins used as substrates:

- Cyclopentene (1.7 µL, 0.020 mmol, 1 eq.)
- Allylbenzene (2.6 µL, 0.020 mmol, 1 eq.)
- 1,4-Pentadiene (2.0 µL, 0.020 mmol, 1 eq.)

Cyclopentene
Only free olefin (shift of about 1 cm^{-1}) and CH_2Cl_2 were observed.

Allylbenzene
Only free olefin and CH_2Cl_2 were observed.

1,4-Pentadiene
Only free olefin and CH_2Cl_2 were observed.

Free olefin was detected due to the poor solubility of the bismuth compounds under the given conditions.

Kinetic isotope effect experiments

1. Independent KIE results

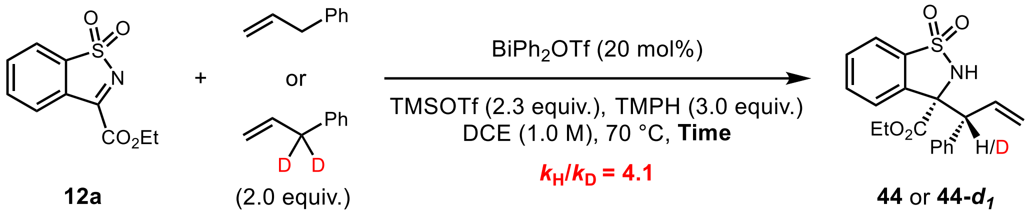

To a flame-dried reaction tube charged with a magnetic stir bar, $BiPh_2OTf$ (10.2 mg, 20 mol%), *N*-sulfonyl ketimine **12a** (23.9 mg, 0.1 mmol, 1.0 equiv.), dry DCE (0.1 mL), allylbenzene (26.5 μL, 0.2 mmol, 2.0 equiv.) or allylbenzene-d_2[4] (24.0 mg, 0.2 mmol, 2.0 equiv.), TMSOTf (41.7 μL, 0.23 mmol, 2.3 equiv.), TMPH (50.6 μL, 0.3 mmol, 3.0 equiv.) were added in succession in an argon-filled glovebox. The tube was sealed and placed in an oil bath preheated at 70 °C with vigorous stirring. Reactions were stopped at corresponding times within 0 min to 60 min. Yields were determined by ^{1}H NMR spectroscopy using 2,4-dinitrotoluene as the internal standard. The average yields of two runs were reported (Table S1) and used for calculation of the KIE value (Chart S1). For the reaction conducted for 12 h, the product was afforded in 63% yield, >20:1 b/l, 13:1 d.r..

Table S1. Original data for independent kinetic experiments (% yield).

	5 min	15 min	30 min	45 min	60 min
allylbenzene	2.2	4.7	8.0	10.7	15.0
allylbenzene-d_2	0.5	1.1	2.1	3.0	3.4

Chart S1. Initial rates with allylbenzene and allylbenzene-d_2.

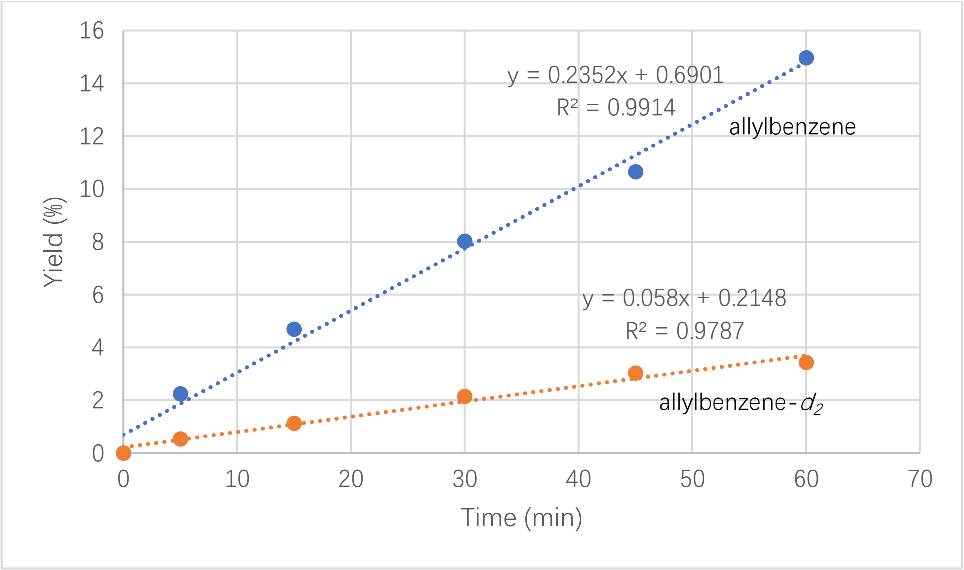

$\boldsymbol{k_H/k_D}$ = 0.2352/0.058 = 4.1

2. Competition KIE results

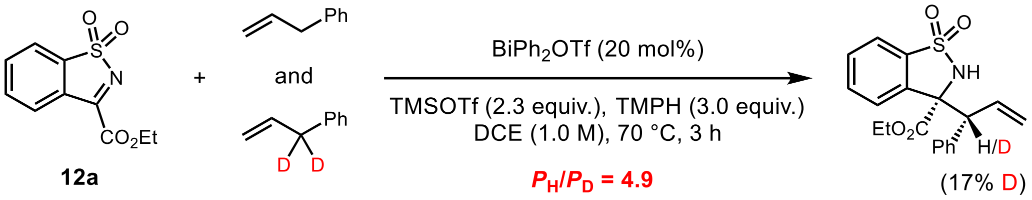

To a flame-dried reaction tube charged with a magnetic stir bar, $BiPh_2OTf$ (30.7 mg, 20 mol%), *N*-sulfonyl ketimine **12a** (71.8 mg, 0.3 mmol, 1.0 equiv.), dry DCE (0.3 mL), allylbenzene (39.7 µL, 0.3 mmol, 1.0 equiv.), allylbenzene-d_2 (36.1 mg, 0.3 mmol, 1.0 equiv.), TMSOTf (125 µL, 0.69 mmol, 2.3 equiv.), TMPH (152 µL, 0.9 mmol, 3.0 equiv.) were added in succession in an argon-filled glovebox. The tube was sealed and placed in an oil bath preheated at 70 °C with vigorous stirring. After 3 h, the tube was allowed to cool to room temperature. The crude mixture was concentrated *in vacuo* and purified by flash column chromatography on silica gel (18.2 mg, 17% yield, >20:1 b/l, >20:1 d.r., eluent: 3:1 hexanes/EtOAc) to obtain a mixture of **44** and **44-d_1**. The pure products were then analyzed by ^{1}H NMR spectroscopy (included in the NMR spectra section), and the KIE value was calculated based on the product ratio.

$P_H/P_D = 0.83/0.17 = 4.9$

Stoichiometric experiments

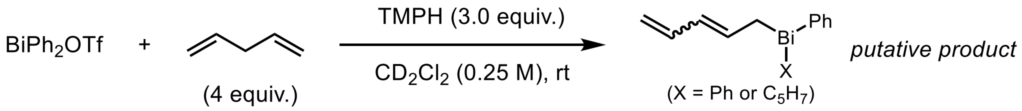

To an oven-dried NMR tube was added $BiPh_2OTf$ (51.2 mg, 0.1 mmol, 1.0 equiv.), CD_2Cl_2 (0.4 mL), 1,4-pentadiene (41.3 µL, 0.4 mmol, 4.0 equiv.) and TMPH (50.6 µL, 0.3 mmol, 3.0 equiv.) in rapid succession. The NMR tube was shaken vigorously to mix all the reagents completely, and was placed in dark for 1 h to initiate the reaction. Then the reaction mixture was subjected to NMR spectroscopy and HRMS.

The complex $BiPh_2OTf$ is only slightly soluble in CD_2Cl_2. Upon the addition of TMPH, the $BiPh_2OTf$ completely dissolved, and the original colorless suspension turned into a clear brown solution. Small amounts of black precipitates were formed at the bottom of the NMR tube over time, which are suggested to be Bi^0 or other unknown low-valent bismuth species generated from the degradation of the alkyl bismuth species.

1. NMR experiment at room temperature

The course of the reaction was monitored through ^1H NMR spectroscopy. New signals at 6.6 ppm and 3.2 ppm were detected with low intensity and broad shape, which are transient and disappeared within 12 h. The compound corresponding to these signals is vacuum-sensitive and could not be isolated to date. We suspected that this newly generated species is the allylbismuth complex, which is known to be unstable and hard to purify. Meanwhile, the homocoupling product deca-1,3,7,9-tetraene was gradually formed and remained stable after 12 h, which is suggested to be the decomposition product of allylbismuth species via radical pathways.[5] This compound was isolated and characterized.

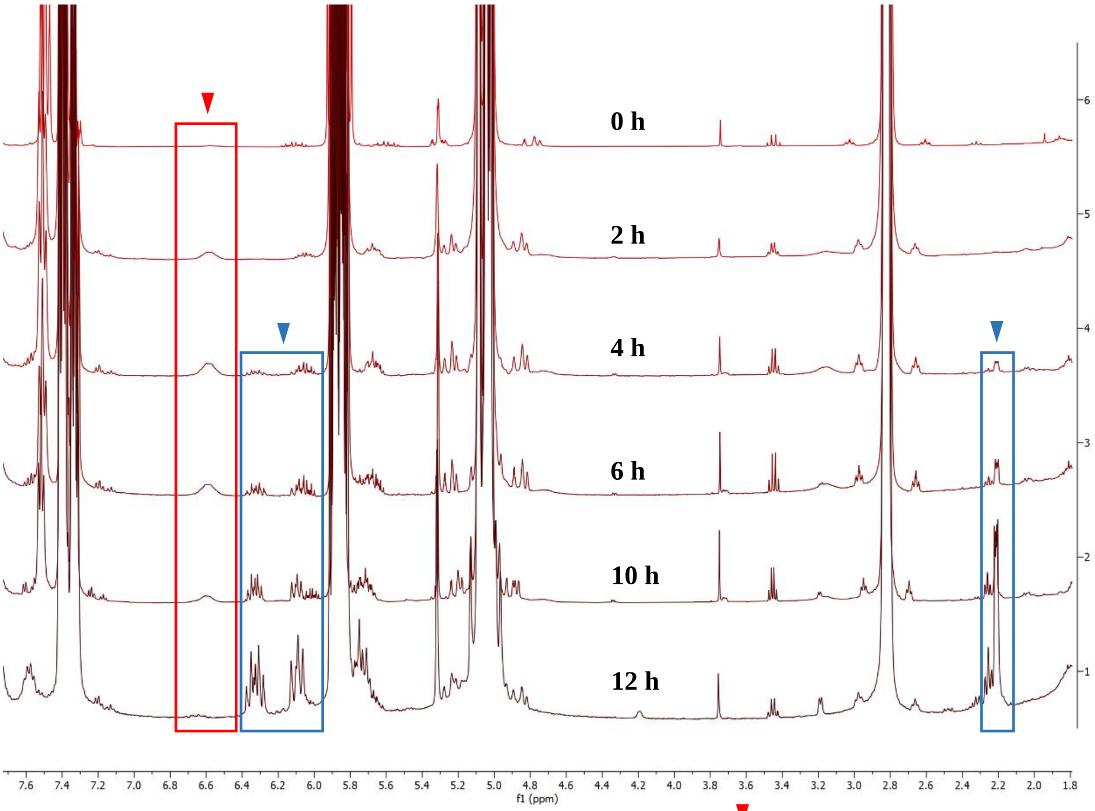

Figure S23. ^1H NMR spectra of the reaction mixture over time. ▼ newly generated transient olefin species (allylbismuth species); ▼ deca-1,3,7,9-tetraene.

Deca-1,3,7,9-tetraene

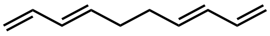

^{1}H NMR (400 MHz, $CDCl_3$) δ 6.38 – 6.28 (m, 2H), 6.14 – 6.05 (m, 2H), 5.78 – 5.68 (m, 2H), 5.13 (d, *J* = 17.3 Hz, 2H), 5.00 (d, *J* = 9.4 Hz, 2H), 2.24 – 2.20 (m, 4H). ^{1}H NMR spectroscopic data were in agreement with the literature.[6]

2. ^{1}H VT NMR experiment

The reaction mixture was analyzed by ^{1}H VT NMR spectroscopy in a temperature range of +23 °C to –25 °C for further observing the behavior of the transient olefin species mentioned above. The olefin peaks appear broad in the spectrum recorded at ambient temperature; however, as the temperature decreases the splitting pattern becomes more distinct. This phenomenon is likely attributed to the allyl exchange behavior typical of allylmetal complexes.

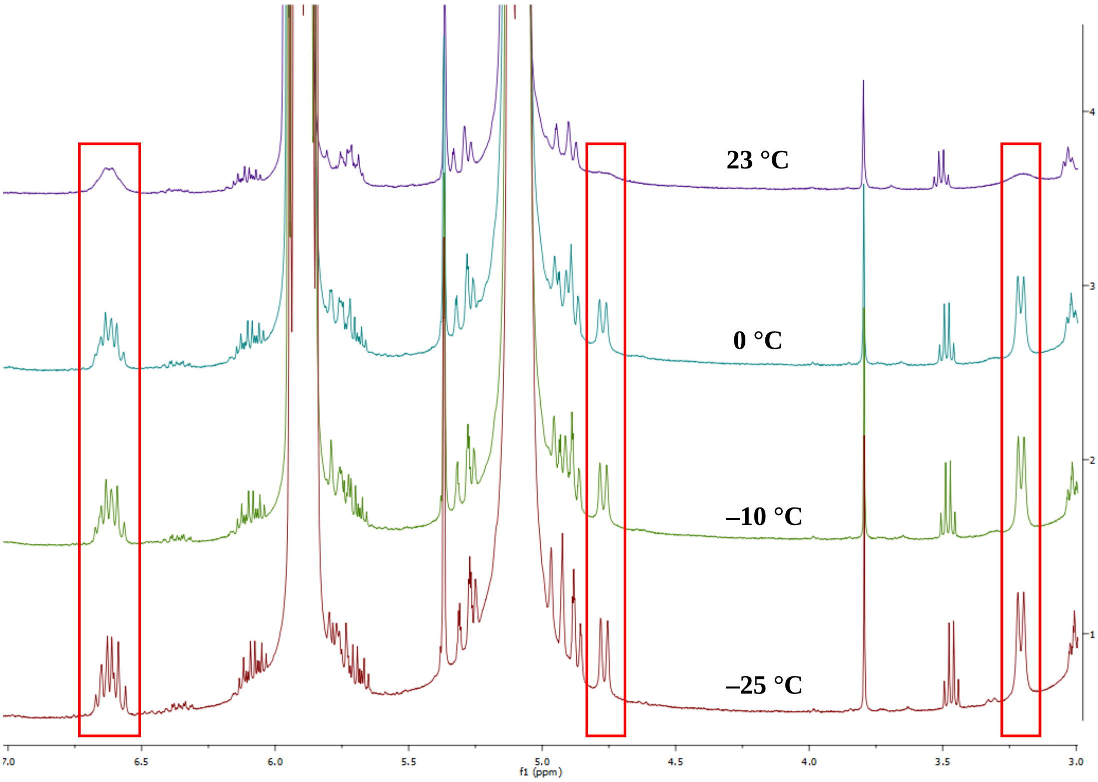

Figure S24. ^{1}H VT NMR spectra of the reaction mixture—region of the newly generated transient olefin species (allylbismuth species).

3. HRMS experiment

The reaction mixture was analyzed by HRMS, the peaks corresponding to pentadienylbismuth complexes and deca-1,3,7,9-tetraene were detected.

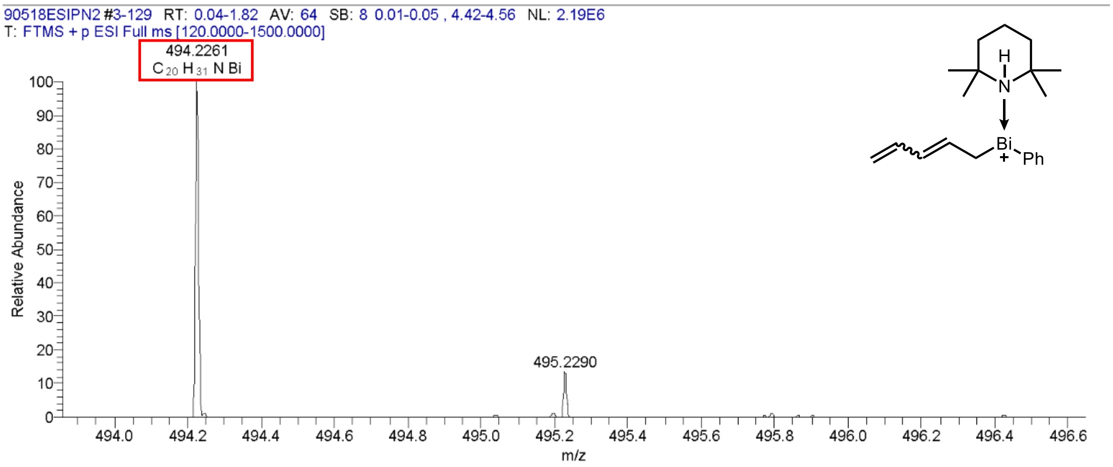

Figure S25. HRMS spectrum of species [BiPh(pentadienyl)(TMPH)]$^+$.

HRMS (ESI) calcd for $C_{20}H_{31}NBi$ [M−C_6H_5+$C_9H_{19}N$]$^+$: 494.2255, found: 494.2261.

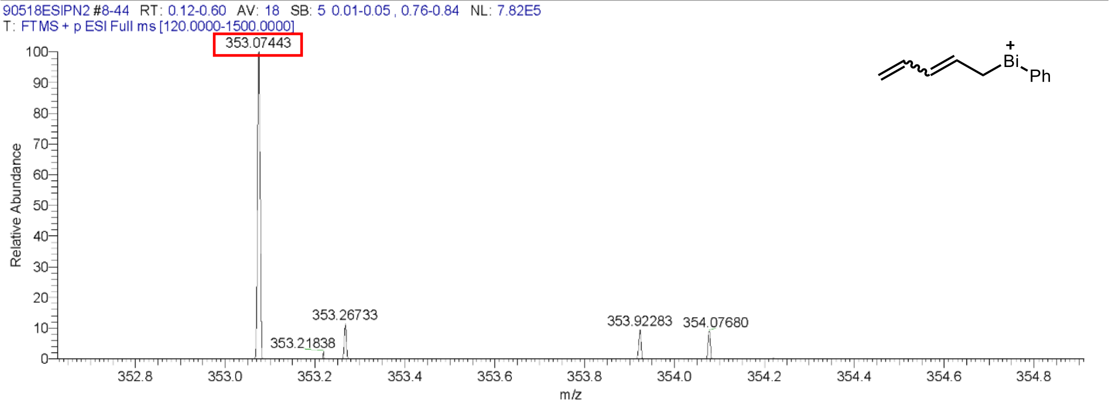

Figure S26. HRMS spectrum of species [BiPh(pentadienyl)]$^+$.

HRMS (ESI) calcd for $C_{11}H_{12}Bi$ [M−C_6H_5]$^+$: 353.0737, found: 353.0744.

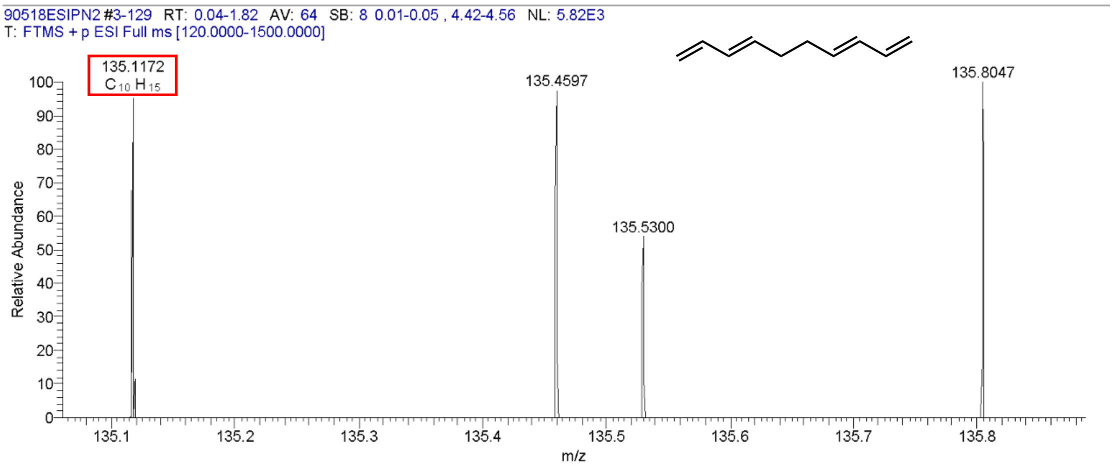

Figure S27. HRMS spectrum of deca-1,3,7,9-tetraene.

HRMS (ESI) calcd for $C_{10}H_{15}$ [M+H]$^+$: 135.1168, found: 135.1172.

4. Monitoring the course of the C−H functionalization reaction through ^{1}H NMR spectroscopy

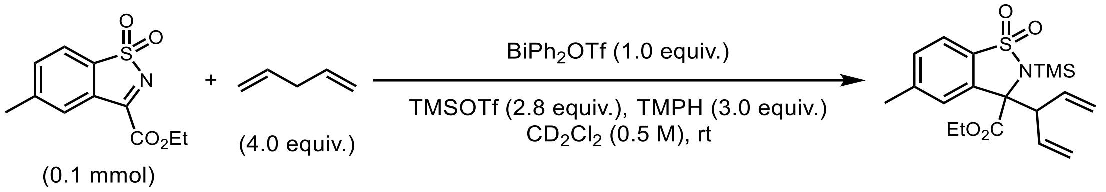

Only the starting materials and the product were observed when the reaction was progressing. Upon completion of the reaction, deca-1,3,7,9-tetraene began to accumulate.

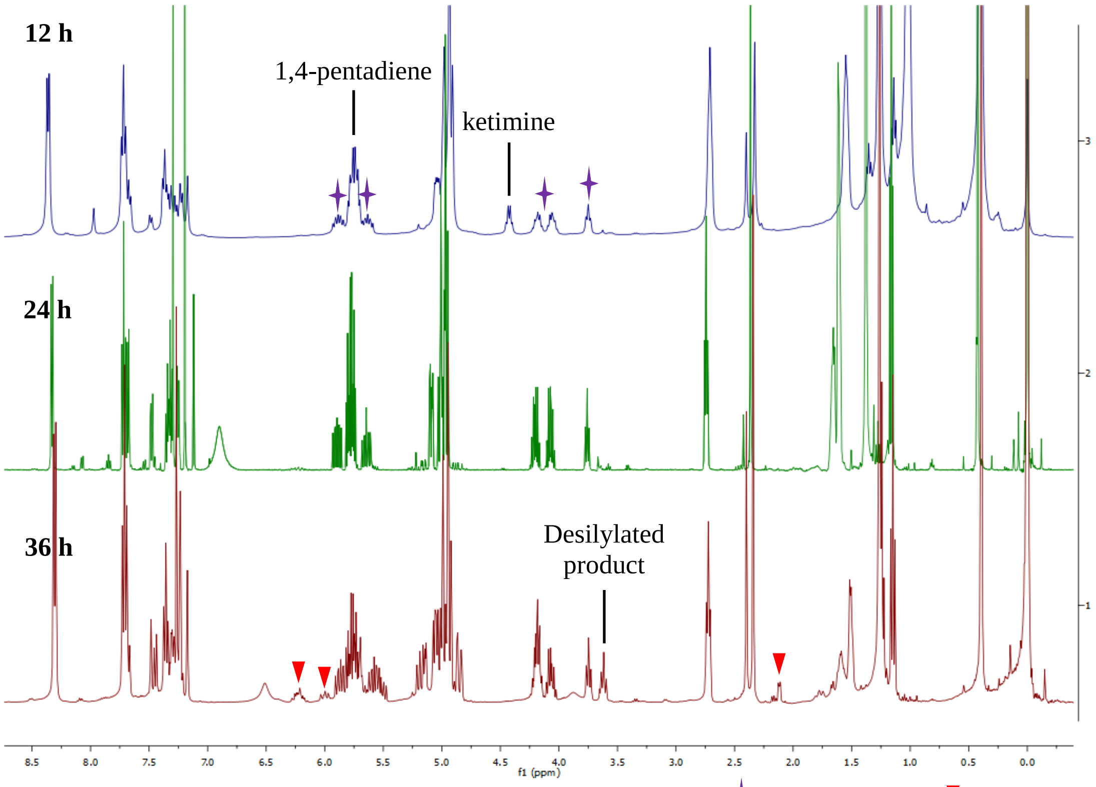

Figure S28. ^{1}H NMR spectra of the C−H functionalization reaction. ✦ reaction product; ▼ deca-1,3,7,9-tetraene.

5. EPR spectroscopic experiment

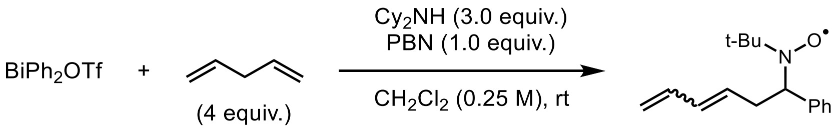

The pentadienyl group in compound $BiPh_2(C_5H_7)$ or $BiPh_2(C_5H_7)$(TMPH) can be expected to show a strong radical leaving group character. Thus, an EPR spectroscopic experiment was performed to check for the formation of the pentadienyl radical.

In a glovebox $BiPh_2OTf$ (13.0 mg, 0.025 mmol, 1.0 equiv.), PBN (4.49 mg, 0.025 mmol, 1.0 equiv.), CH_2Cl_2 (1 mL), 1,4-pentadiene (10.3 µL, 0.1 mmol, 4.0 equiv.) and Cy_2NH (14.9 µL, 0.075 mmol, 3.0 equiv.) were combined in rapid succession. The reagents were mixed thoroughly to initiate the reaction. 0.25 mL of the reaction mixture were transferred to an oven dried EPR tube and subjected to EPR spectroscopy. (*Note*: Cy_2NH was used as the base instead of TMPH in this experiment because the latter

contains trace amounts of TEMPO which interferes with the detection of radicals generated during the reaction. The C−H deprotonation reaction mediated by Cy_2NH proceeded in the same way as that with TMPH, as determined by ^1H NMR spectroscopy and HRMS.)

EPR-spectroscopic reaction monitoring revealed a resonance characteristic of a hydrocarbon radical captured by PBN (Figure S29), which is ascribed to the addition of the pentadienyl radical to PBN.

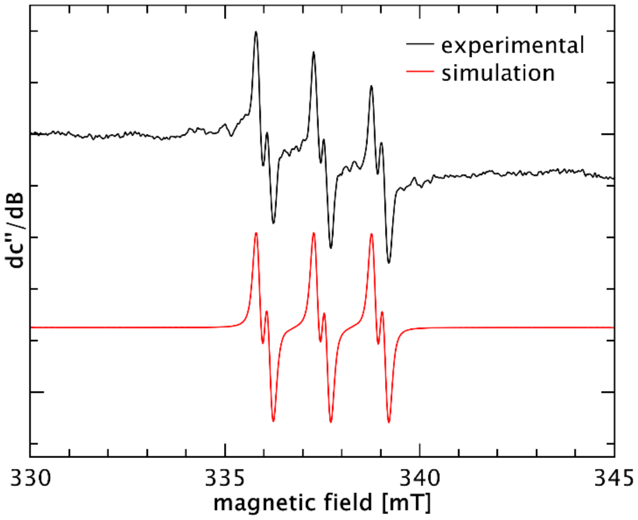

Figure S29. Experimental (black) and simulated (red) continuous-wave (CW) X-band EPR spectra of the degradation of a CH_2Cl_2 solution containing 1 equiv. $BiPh_2OTf$ (c = 0.024 mol/L), 1 equiv. PBN, 3 equiv. $HNCy_2$, and 4 equiv. 1,4-pentadiene.) The observed resonance shows coupling constants of a(^{14}N) = 41.4 MHz (14.7 G), a(^1H) = 6.8 MHz (2.4 G) and a g_{iso} value of 2.0050. Spectrometer settings: microwave frequency = 9.472598 GHz, 0.2 mT modulation amplitude at 100 kHz, microwave power = 10 mW, number of accumulated scans = 10, conversion time = 2 ms. Control experiments in the absence of 1,4-pentadiene indicated the transfer of a pentadienyl group to be responsible for the appearance of the EPR spectroscopic resonance.

Computational Mechanistic Studies

1.1 Computational Details.

All geometry optimizations and single point energy calculations were performed using the Gaussian16[7] suite of programs. at the M06-L+GD3/def2-TZVP$^{(C,H,N,O,S,F)}$/LanL2DZ$^{(Bi,Si)}$$_{(DCE,SMD)}$//M06-L+GD3/def2-SVP/LanL2DZ$^{(Bi,Si)}$ level of theory[8-13]. Natural Bond Orbital (NBO) calculations were carried out using the program NBO 6.0.[14] Intrinsic Bond Orbitals (IBO) derived from Intrinsic Atomic Orbitals (IAOs) were computed using the IBOview (v20150427) program implemented by the Knizia group to directly interpret chemical AOs and chemical bonds.[15] Readable *.molden files for IBOview were generated using the following procedure. The Gaussian *.chk files, which contained information on the targeted wavefunctions, were formatted to *.fchk files using the formchk utility from Gaussian16 program, and then converted to *.molden files using utilities from the program Multiwfn.[16] The generated files were then loaded to IBOview, and the chemical analysis was performed, importing all the wavefunction information calculated at the abovementioned level of theory. Atomic Orbital Contributions were calculated with the Multiwfn program using the output Gaussian files resulted from the NBO calculations. Note: cationic $TMPH_2^+$ or anionic OTf^- species generated during the reaction were considered as solvated non-interacting species. The dimeric form of $[TMPH_2^+][OTf^-]$ was found to be isoenergetic in comparison to the individual species (ΔG = 0.04 kcal/mol).

1.2 General Considerations

In addition to the most feasible reaction pathway presented in Figure 1 of the manuscript, we computed three other pathways that could be considered as potential mechanistic scenarios, taking as a starting point the allylbismuth species: 1) outer-sphere electrophilic attack of the substrate to the allylbismuth complex, 2) a free radical reaction upon homolysis of allylbismuth species and 3) insertion into Bi–C bond of allylbismuth species. All these scenarios are predicted to have higher overall free energy barriers and are therefore unlikely pathways. A detailed discussion is provided in the sections below.

The full reaction profile with all related structures is presented in Figure S30.

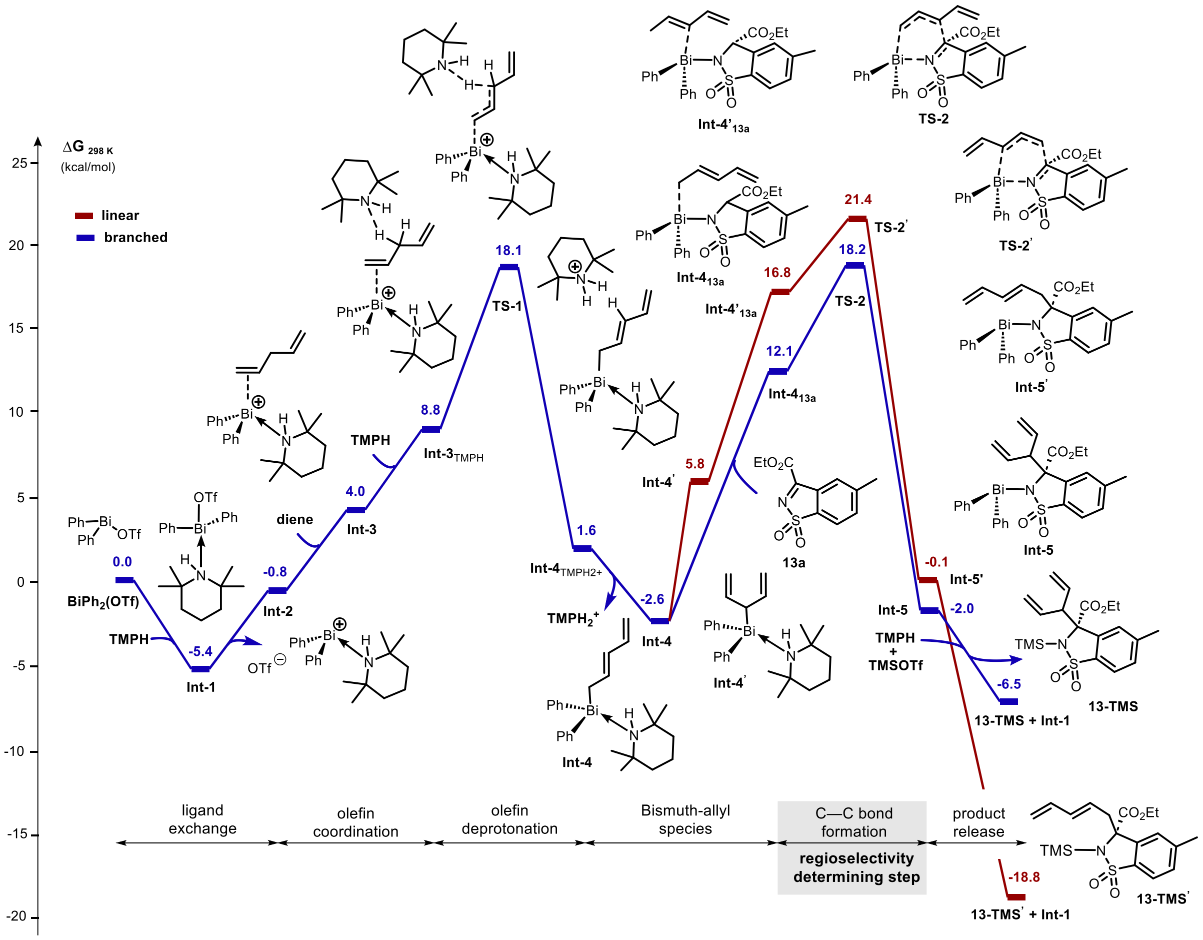

Figure S30. Full reaction profile with all intermediates depicted.

1.2.1 Formation of the linear product

As shown in the manuscript, the preferred reaction pathway for the formation of the main product involves a six-membered transition state. We computed the analogous pathway that leads to the formation of the linear (minor) product to gain insights into the origin of the observed regioselectivity. The computed reaction profile is shown in Figure S31 (see red and blue lines to compare the profiles for linear vs branched product formation, respectively). Starting from the linear allylbismuth species **Int-4**, an isomerization process is required to form the corresponding branched allylbismuth species **Int-4’** in an endergonic process (ΔG = 8.4 kcal/mol). The subsequent C–C bond formation step via **TS-2’** to form the linear product involves a free energy barrier of ΔG = 24.0 kcal/mol, which is 3.2 kcal/mol higher than the barrier for the formation of the branched product. This indicates that the C–C bond formation step is likely to be the regioselectivity-determining step of the reaction.

Finally, formation of **Int-5’** and subsequent release of the product **13-TMS’** account for an overall energy of ΔG = –18.8 kcal/mol, indicating that the linear product is thermodynamically more stable than the branched product.

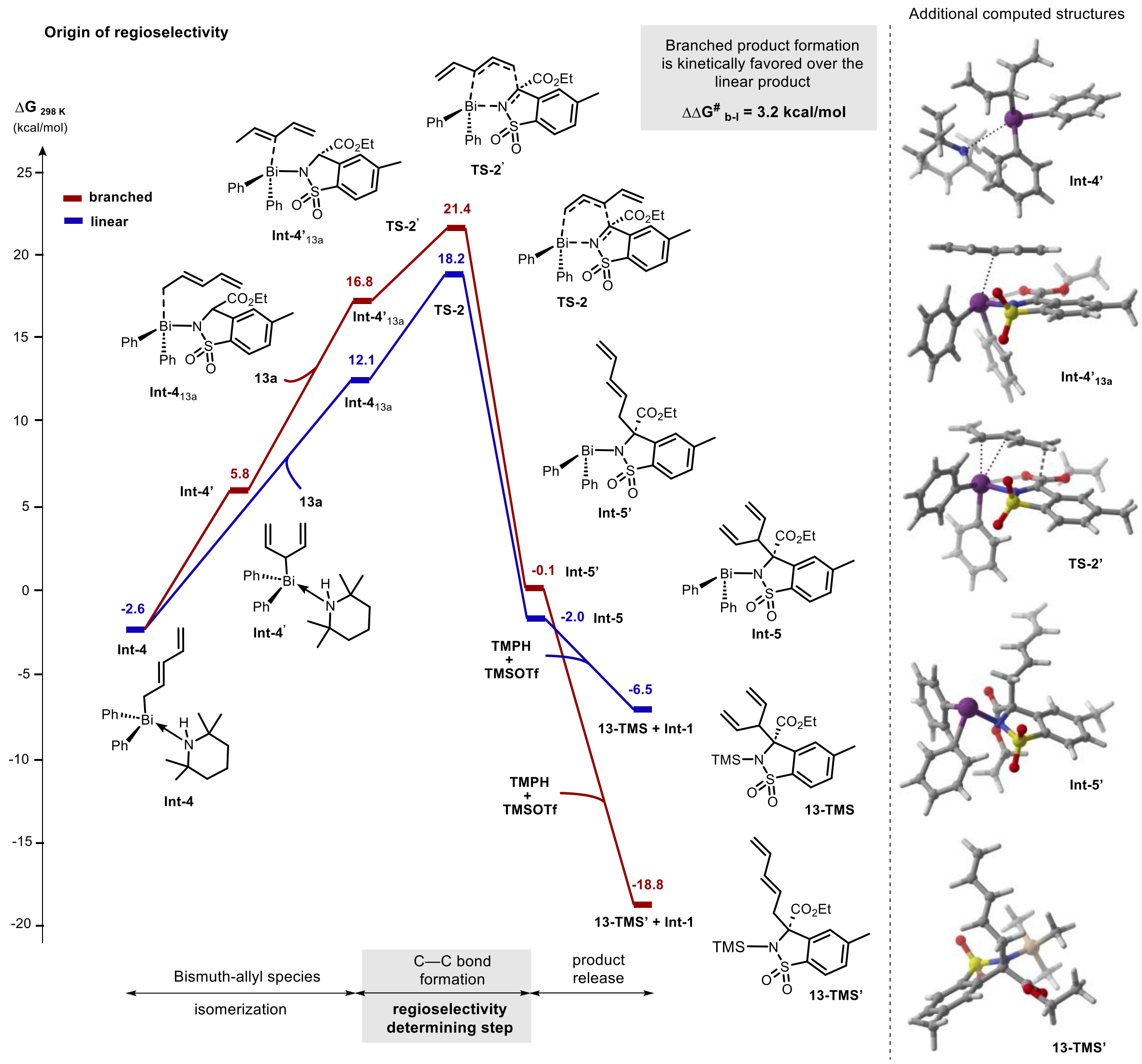

Figure S31. Reaction pathways revealing the origin of regioselectivity. Formation of linear product (red line) vs branched product (blue line) via 6-membered transition state.

1.2.2 Outer-sphere electrophilic attack to allylbismuth species

Another alternative for the C–C bond formation step is an outer-sphere electrophilic attack from the electrophilic substrate to the reacting C-atom of the allylbismuth species, without coordination to the bismuth center. Such a scenario occurring via **TS-2^{out}** has a high energy barrier ($\Delta G^{‡}$ = 34.9 kcal/mol) for the formation of the main product and is therefore rather unlikely (Figure S32).

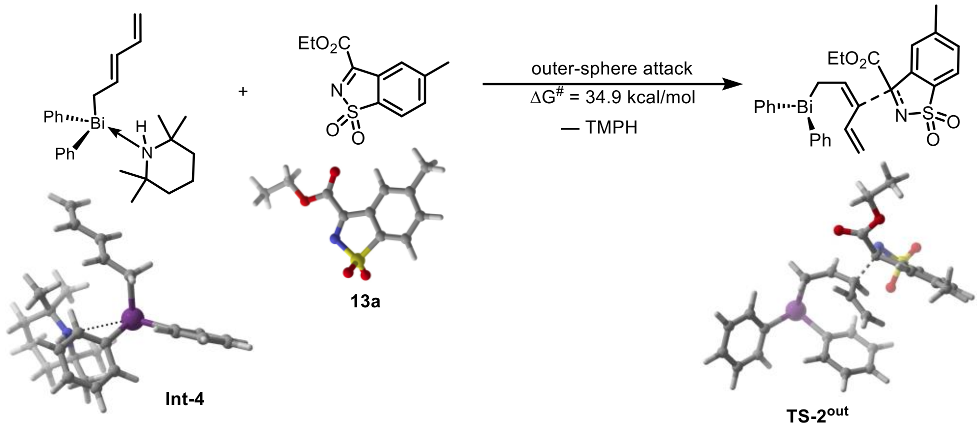

Figure S32. Free energy barrier for an outer-sphere electrophilic attack from substrate **13a** to allylbismuth species.

Additionally, our calculations show that the analogous barrier towards the formation of the linear product via **TS-2'$_{\mathbf{out}}$** accounts for a barrier of $\Delta G^{\ddagger}$ = 22.3 kcal/mol (Figure S33). This barrier is higher than the one that leads to the linear product via **TS-2'** ($\Delta G^{\ddagger}$ = 21.4 kcal/mol in Figure S31), thus suggesting this to be a rather unlikely pathway.

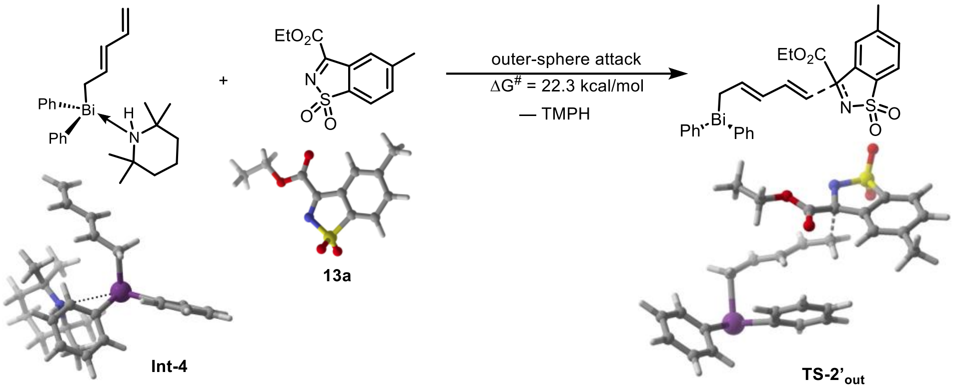

Figure S33. Formation of linear product via an outer-sphere electrophilic attack transition state.

1.2.3 Free radical reaction upon homolysis of allylbismuth species.

We considered that upon formation of allylbismuth species, a radical reactivity scenario is possible if the Bi–C bond of the allyl chain undergoes homolysis to form **alkyl•** and Bi-centered radical species **$BiPh_2$•** (Figure S34). Our calculations predict that such process involves a free energy penalty of 16.5 kcal/mol. Although this scenario cannot be completely excluded, the following step on this pathway, which is the reaction of the electrophilic substrate with the free radical **alkyl•** via **TS-2$^{\mathbf{rad}}$**, leads to an overall high free energy barrier ($\Delta G^{\ddagger}$ = 49.3 kcal/mol), and therefore is a rather unlikely scenario. As reported in the literature, the homolytic cleavage of Bi–X bonds can be reversible when the radicals generated are persistent and do not engage in further reactions on the given time interval.[17-18] In this context, it is worth mentioning that the pair of radicals can easily re-enter the polar reaction pathway by radical recombination to re-generate **Int-4**. Moreover, the calculated spin density on free allyl radical species is almost equally distributed between the central C-atom (iso) and the terminal C-atoms (n). A free radical reaction of such radical will most likely deliver linear: branched with close to 2:1 regioselectivity, which contradicts the experimental findings.

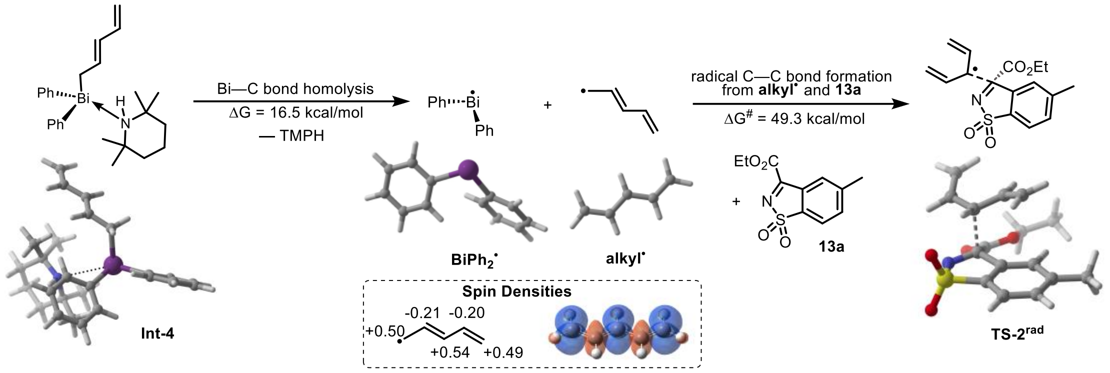

Figure S34. Thermodynamic penalty for the homolysis of allylbismuth species.

1.2.4 Substrate insertion into Bi–C bond of allylbismuth species

We considered the C–C bond formation step occurring via a transition metal-like insertion (Figure S35). The iso-pathway in this hypothetical scenario would involve starting from iso-allyl species **Int-4'**$_{\mathbf{II}}$. The computed free energy barrier to reach transition state **TS-2**$^{\mathbf{ins}}$ from **Int-4'**$_{\mathbf{II}}$ allyl species is considerably high ($\Delta G^{\ddagger}$ = 67.2 kcal/mol) and therefore insertion into Bi–C bond towards the formation of product **13-TMS** is very unlikely to occur.

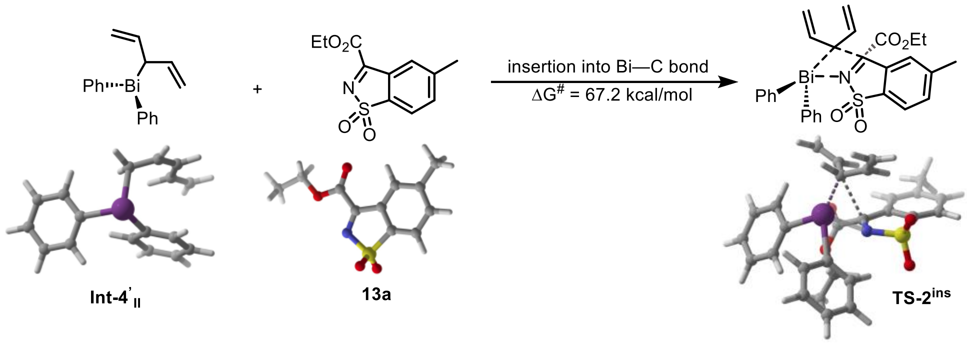

Figure S35. Free energy barrier for substrate **13a** insertion into Bi–C bond of allylbismuth species.

1.3 Study of Bismuth–Olefin Interactions

To further support the experimental findings regarding bismuth–olefin interactions, we performed a computational study on the thermodynamic stability of different bismuth–olefin adducts tested experimentally. Additionally, we gain further insights into the bonding interactions between the olefin unit and the bismuth center by means of NBO and IBO analysis. Furthermore, we provide the predicted IR shifts of the C=C bonds between the free olefin and the coordinated olefin to support the experimental results.

1.3.1 Interaction of $BiMe_2^+$ and $BiPh_2^+$ with olefins.

In Figure S36 and S37 we present the thermodynamic trends for the formation of $BiMe_2SbF_6$ and $BiPh_2SbF_6$ adducts with olefins: cyclopentene, 1,4-pentadiene and allylbenzene.

The formation of an adduct is predicted to be slightly favorable with cyclo-pentene and almost thermoneutral for 1,4-pentadiene and allylbenzene. In addition, we considered as well the arene interaction of allylbenzene to the bismuth center. For such cases, we found (almost) isoenergetic arene-adducts with respect to the adducts with the olefin interacting (see Figure S36 and S37).

Although there is not a strong thermodynamic driving force towards the formation of the adducts, our calculations show that a shift on the infrared spectra corresponding to a change on the olefin ν_s frequency is significant enough to be detected experimentally. Notably, the shifts in the stretching frequencies experimentally detected by IR spectroscopy showed the expected trend derived from our calculations. NBO calculations predict that interactions between the olefin and bismuth center arise from the olefin bond to the lone pair* orbital of bismuth and have an interaction energy of 15–20 kcal/mol. IBO analyses show that the π-electron density of the olefin C=C bond is polarized towards the bismuth atom.

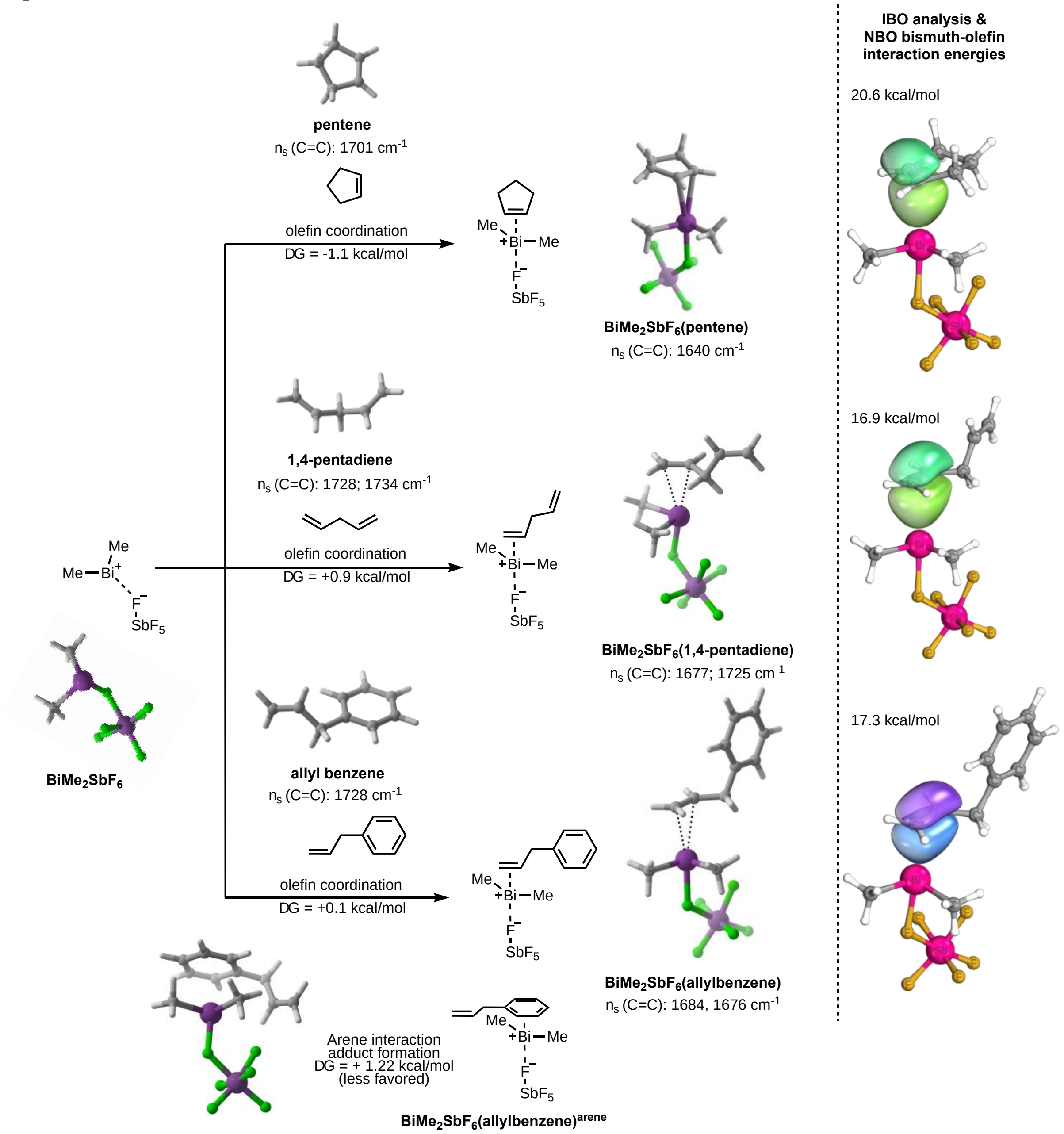

Figure S36. Interaction of $BiMe_2SbF_6$ with olefins.

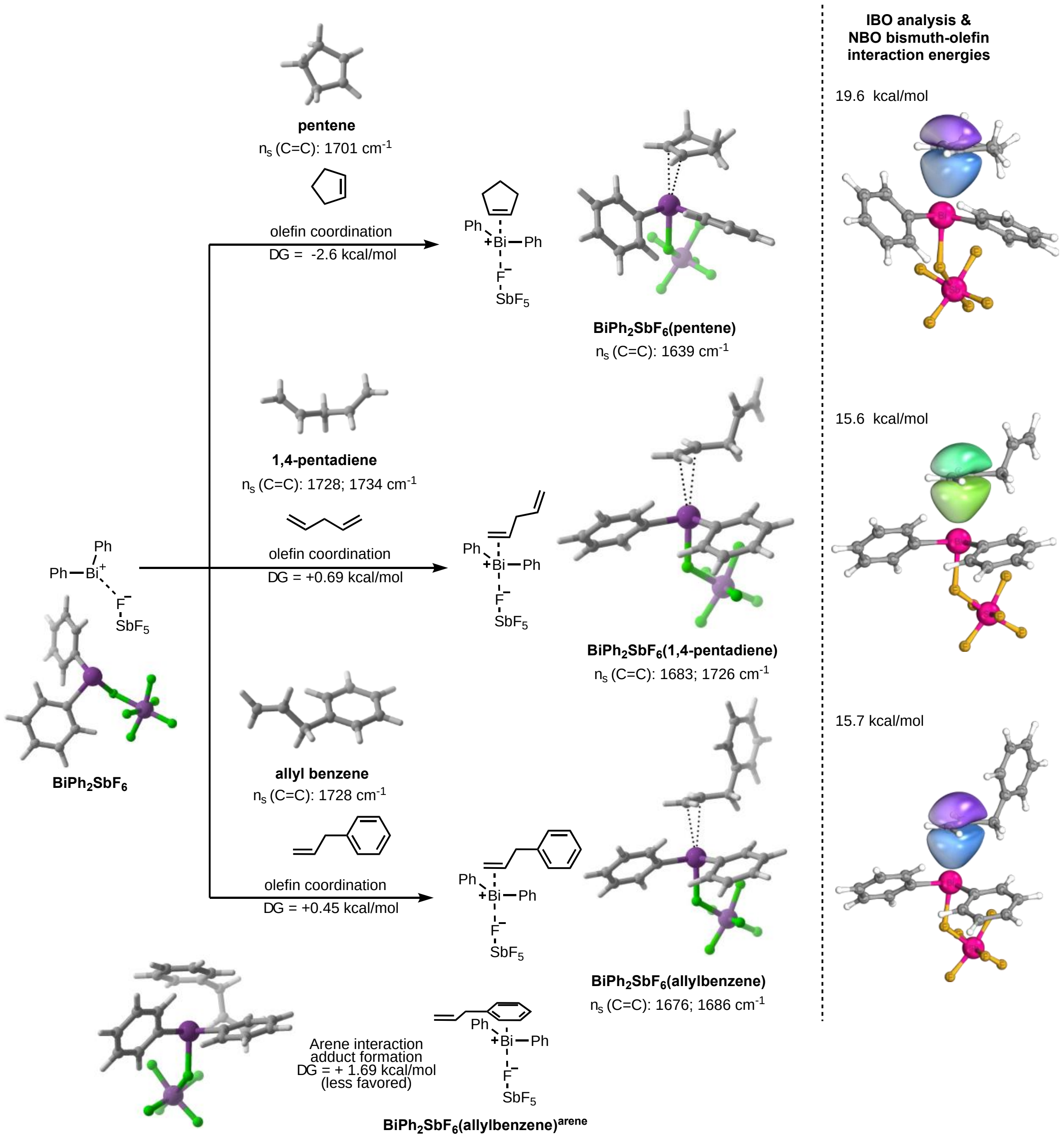

Figure S37. Interaction of $BiPh_2SbF_6$ with olefins.

1.3.2 Frontier Molecular Orbital Analysis and Atomic Orbital Contributions.

We present below the calculated natural atomic orbital (NAO) contributions for the HOMO and LUMO of [$BiMe_2(SbF_6)$(1,4-pentadiene)] (Figure S38), as illustrated in the manuscript (Scheme 4D).

Bi atomic orbitals contribute 50.3% to the HOMO, which is mainly composed of contributions from orbitals: 6s (11.5%), $6p_y$ (16.6%) and $6p_z$ (21.7%). Methyl substituents contribute a total of 37.6%, of which C^2 atom contributes 15.7% with $2p_z$ orbital and C^6 contributes 21.9% with $2p_y$ orbital. The olefin substrate has a contribution of 1.9%, provided mainly by the $2p_x$ orbital of the C^{19} atom. Although the contribution is relatively low, its composition helps rationalize why interactions between bismuth and the olefin are weak. In this context, the olefin interacts through a p_x orbital while

bismuth interacts through orthogonal p_y and p_z orbitals, leading to a relatively poor overlap and therefore weak interaction.

Bi atomic orbitals contribute 41.7% to the LUMO, which is mainly composed of $6p_x$ (41.3%) contribution. The anion $(SbF_6)^-$ contributes 50.4%. Methyl substituents do not contribute significantly (<0.5%). The olefin substrate has a contribution of 4.5%, provided mainly by the $2p_x$ orbital (4.2%) of the C^{19} atom.

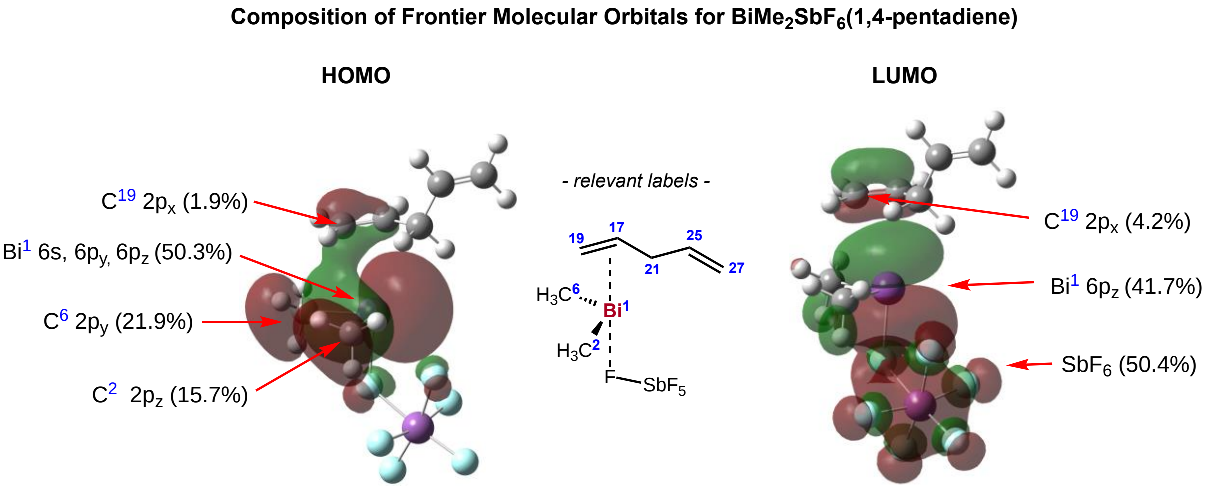

Figure S38. Main contributions of natural atomic orbitals (NAO) to the HOMO and LUMO of $BiMe_2SbF_6$(1,4-pentadiene).

1.3.2.1 Calculated NAO Contributions for HOMO of $BiMe_2SbF_6$(1,4-pentadiene).

Note: All Rydberg NAOs/shells or contributions <= 0.50 % are not printed

NAO#	Center	Label	Type	Composition
2	1(Bi)	s	Val(6s)	11.533 %
13	1(Bi)	py	Val(6p)	16.663 %
18	1(Bi)	pz	Val(6p)	21.734 %
52	2(C)	s	Val(2s)	0.539 %
62	2(C)	pz	Val(2p)	15.713 %
94	5(H)	s	Val(1s)	1.618 %
101	6(C)	s	Val(2s)	0.972 %
108	6(C)	py	Val(2p)	21.940 %
137	8(H)	s	Val(1s)	0.569 %
143	9(H)	s	Val(1s)	1.844 %
365	16(F)	pz	Val(2p)	0.828 %
427	19(C)	px	Val(2p)	1.866 %

Condensed NAO terms to shells:

Atom:	1(Bi)	Shell:	2(6s Val)	11.533 %
Atom:	1(Bi)	Shell:	8(6p Val)	38.451 %
Atom:	2(C)	Shell:	18(2s Val)	0.539 %
Atom:	2(C)	Shell:	22(2p Val)	16.057 %
Atom:	5(H)	Shell:	36(1s Val)	1.618 %
Atom:	6(C)	Shell:	41(2s Val)	0.972 %
Atom:	6(C)	Shell:	45(2p Val)	22.407 %
Atom:	8(H)	Shell:	55(1s Val)	0.569 %
Atom:	9(H)	Shell:	59(1s Val)	1.844 %
Atom:	13(F)	Shell:	106(2p Val)	1.046 %
Atom:	16(F)	Shell:	139(2p Val)	1.457 %
Atom:	19(C)	Shell:	165(2p Val)	1.902 %

Composition of different types of shells (%):
s: 18.071 p: 81.797 d: 0.092 f: 0.031 g: 0.000 h: 0.000

Condensed NAO terms to atoms:
Center Composition

1(Bi)	50.308 %
2(C)	16.634 %
5(H)	1.641 %
6(C)	23.422 %
8(H)	0.581 %
9(H)	1.870 %
13(F)	1.067 %
16(F)	1.465 %
19(C)	1.913 %

Core composition: 0.020 %
Valence composition: 99.403 %
Rydberg composition: 0.569 %

Orbital delocalization index: 33.70

1.3.2.2 NAO Contributions for LUMO of $BiMe_2SbF_6$(1,4-pentadiene).

Note: All Rydberg NAOs/shells or contributions <= 0.50 % are not printed

NAO#	Center	Label	Type	Composition
8	1(Bi)	px	Val(6p)	41.306 %
150	10(Sb)	s	Val(5s)	28.345 %
210	11(F)	pz	Val(2p)	3.367 %
235	12(F)	px	Val(2p)	2.326 %
238	12(F)	py	Val(2p)	1.186 %
266	13(F)	px	Val(2p)	1.325 %
269	13(F)	py	Val(2p)	1.212 %
303	14(F)	pz	Val(2p)	3.115 %
328	15(F)	px	Val(2p)	0.805 %
331	15(F)	py	Val(2p)	1.935 %
355	16(F)	s	Val(2s)	1.411 %
362	16(F)	py	Val(2p)	1.690 %
427	19(C)	px	Val(2p)	4.162 %

Condensed NAO terms to shells:

Atom:	1(Bi)	Shell:	8(6p Val)	41.681 %
Atom:	10(Sb)	Shell:	64(5s Val)	28.345 %
Atom:	11(F)	Shell:	84(2p Val)	3.522 %
Atom:	12(F)	Shell:	95(2p Val)	3.685 %
Atom:	13(F)	Shell:	106(2p Val)	2.747 %
Atom:	14(F)	Shell:	117(2p Val)	3.332 %
Atom:	15(F)	Shell:	128(2p Val)	2.770 %
Atom:	16(F)	Shell:	135(2s Val)	1.411 %
Atom:	16(F)	Shell:	139(2p Val)	2.041 %
Atom:	19(C)	Shell:	165(2p Val)	4.242 %

Composition of different types of shells (%):
s: 33.944 p: 65.711 d: 0.254 f: 0.094 g: 0.000 h: 0.000

Condensed NAO terms to atoms:

Center	Composition
1(Bi)	42.323 %
10(Sb)	29.334 %
11(F)	3.798 %
12(F)	4.009 %
13(F)	3.162 %
14(F)	3.627 %
15(F)	3.022 %
16(F)	3.746 %
19(C)	4.485 %

Core composition: 0.003 %
Valence composition: 97.292 %
Rydberg composition: 2.708 %

Orbital delocalization index: 27.49

1.3.2.3 HOMO of Int-4 and TS-1.

In Figure S39 we present the HOMO for **TS-1** and **Int-3**$_{TMPH}$. While the HOMO of **Int-3**$_{TMPH}$ is centered on the N atom of TMPH participating in the deprotonation, the electron density is then shifted towards the olefin and the B–C bond to be formed in **TS-1**, as depicted by the arrow pushing in Figure S39a.

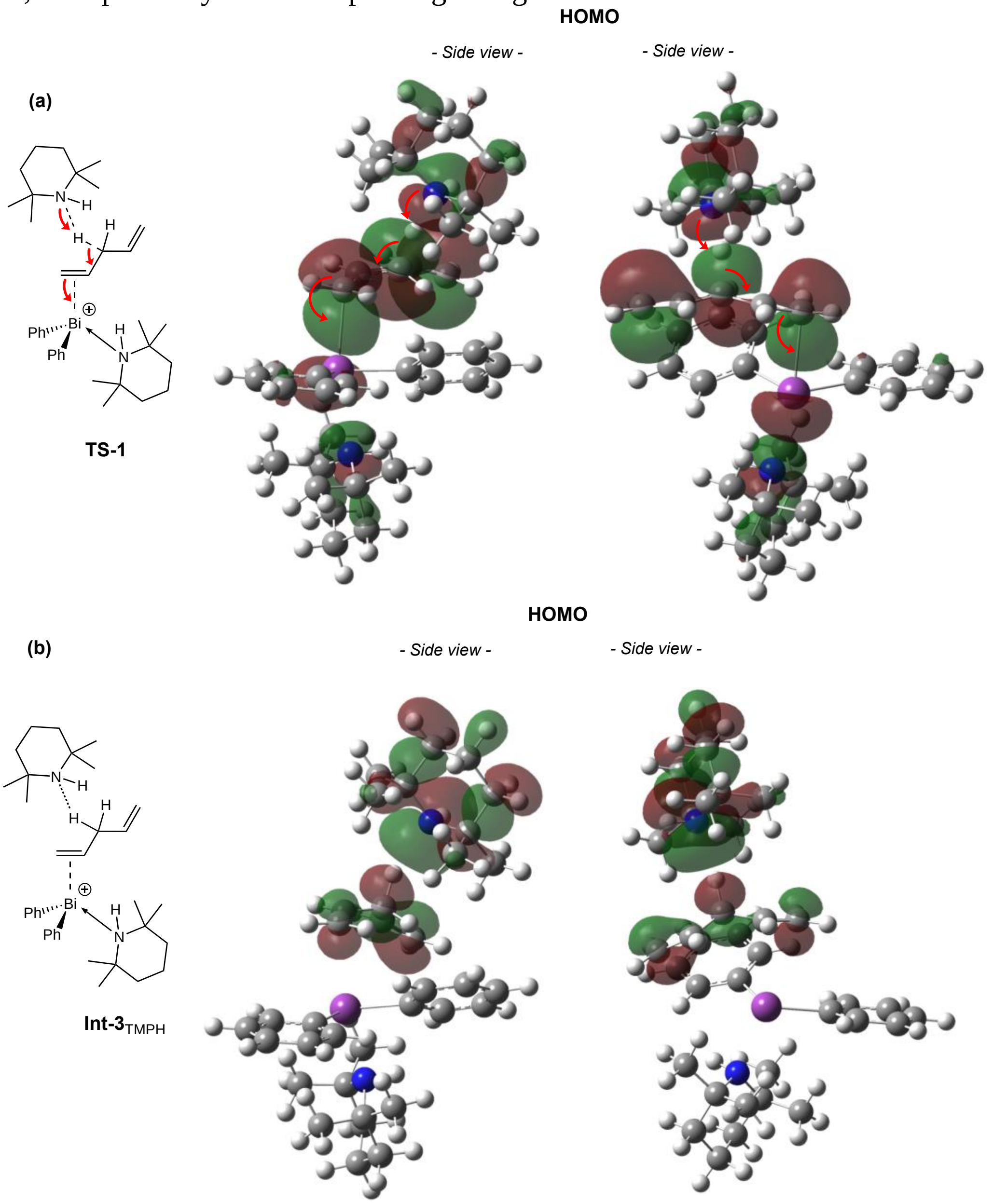

Figure S39. HOMO for **TS-1** (a) and **Int-4** (b).

1.3.3 Activation of substrate 13a by TMSOTf.

We considered that electrophilic substrate **13a** could be activated by reacting with TMSOTf to form **13a-TMS**$^{+}$. Such a process is endergonic (ΔG = 15.3 kcal/mol) and therefore is rather unlikely. Specifically, comparison with the activation by $BiPh_2(C_5H_7)$ (as in **Int-4**$_{13a}$) shows that the pathway involving **13a-TMS**$^{+}$ would be disfavored, because i) the TMS group sterically shields the electrophilic iminium

carbon atom and ii) the critical C–C bond forming step would be an intermolecular reaction.

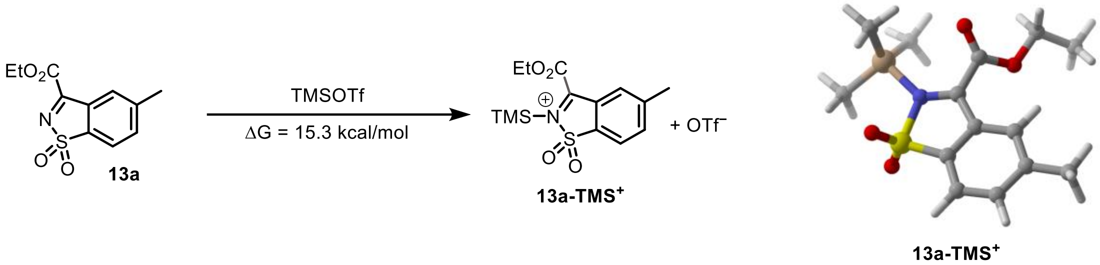

Figure S40. Activation of the substrate by TMSOTf is rather unlikely.

1.4 Role of TMS^+ in the regioselectivity switch for aldehyde substrates.

In the case of aldehyde substrates, the choice of Lewis acid, TMSOTf or $BF_3{\cdot}Et_2O$, allowed the regioselective formation of linear or branched product, respectively. We hypothesized that the Lewis acid would promote a switch in the mechanism from inner-sphere (as shown in ketimine substrates) to outer-sphere, if there is sufficient steric repulsion imposed by the Lewis acid (in this case, TMS^+). Our calculations confirmed this hypothesis, showing a larger energy barrier for the inner sphere mechanism when the aldehyde is activated by TMS^+ (Figure S41), accounting for an overall barrier of +35.5 kcal/mol (linear, **TS-2’** 1a-TMS) and +32.0 kcal/mol (branched, **TS-2** 1a-TMS).

In this context, the outer-sphere mechanism presented lower energy barriers than the inner-sphere mechanism, +20.5 kcal/mol (linear, **TS-2’$_{out}$** 1a-TMS) and +23.0 kcal/mol (branched, **TS-2$_{out}$** 1a-TMS), thus indicating the plausible switch in the C–C bond formation step transition state. Moreover, the predicted regioselectivity for the outer-sphere mechanism indicate that the linear product formation is favored over the branched product by a difference in activation energy of +2.5 kcal/mol, which agrees with the regioselectivity observed in the experiments.

Finally, we corroborated the feasibility of the inner-sphere mechanism for the case of $BF_3{\cdot}Et_2O$ (Figure S42). Our calculations predict an overall barrier of +24.0 kcal/mol (branched, **TS-2** 1a-BF_3) and +27.7 kcal/mol (linear, **TS-2’** 1a-BF_3), thus indicating the preference towards branched product formation over the linear product, which is in agreement with the experimental observations.

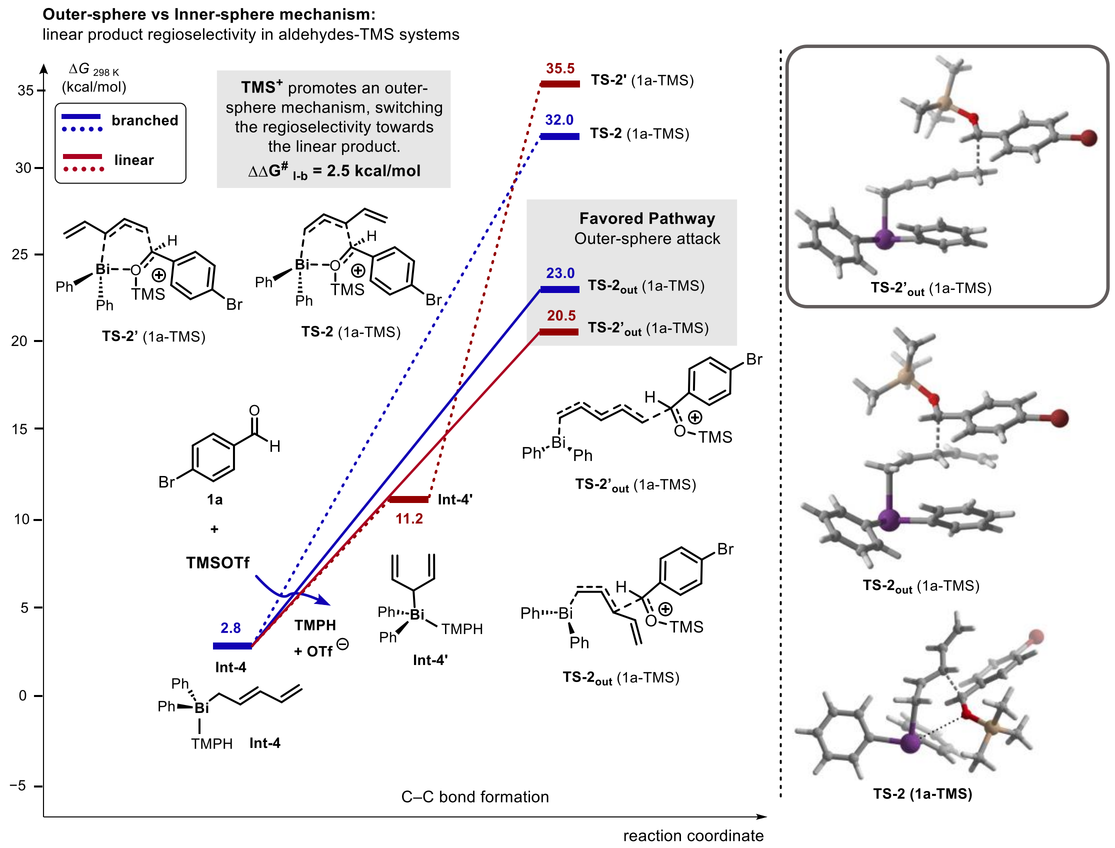

Figure S41. Regioselectivity switch in aldehydes-TMS systems via outer-sphere C–C bond formation transition state.

$BF_3 \cdot Et_2O$ retains inner-sphere mechanism regioselectivity.

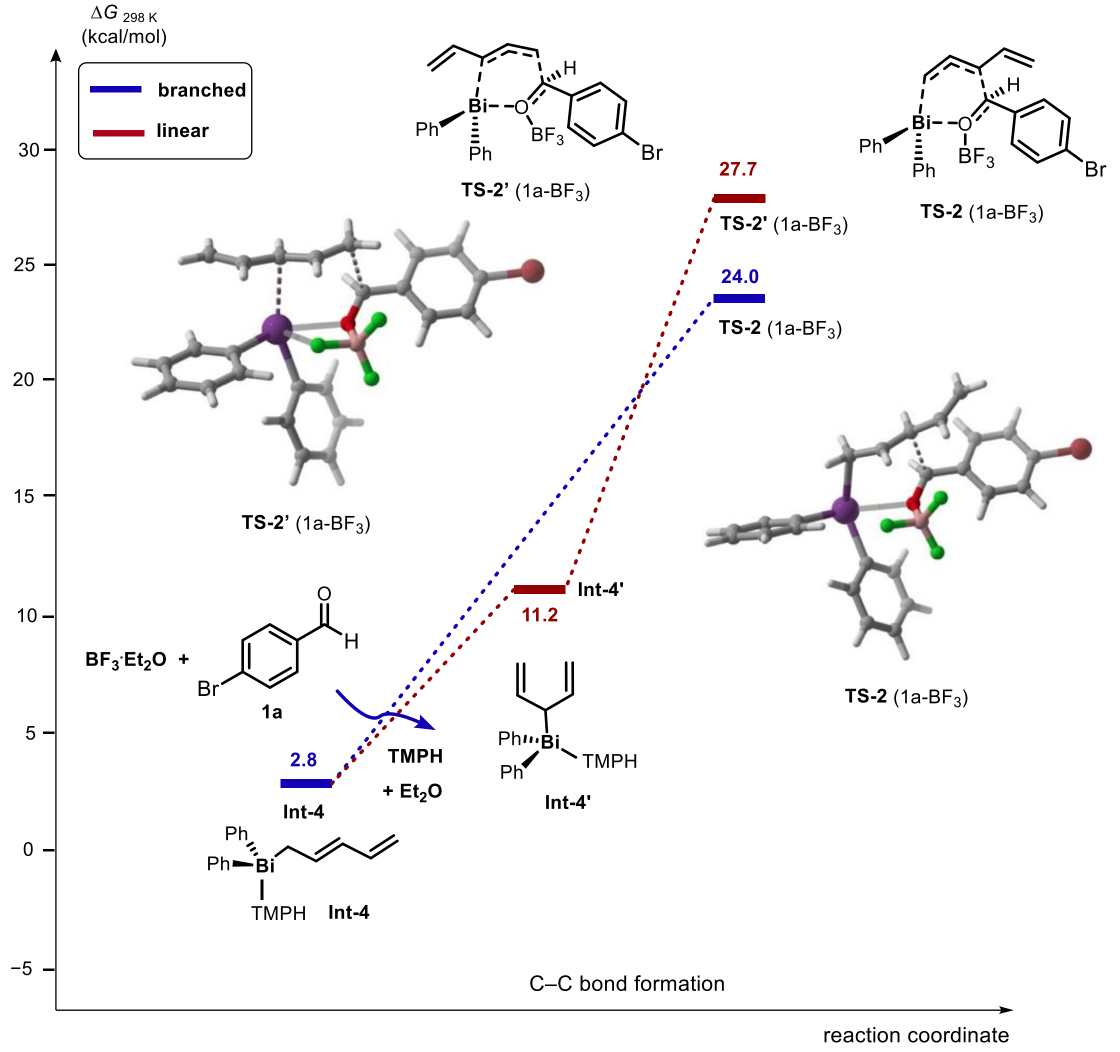

Figure S42. Plausible C–C bond formation step via inner-sphere transition state when $BF_3 \cdot Et_2O$ activates aldehydes substrates.

1.5 Computed values for the optimized structures.

Structure	G_{corr}	G_{298} gas phase	E_0 DCE	G_{298} DCE
TMPH	0.235242	-408.57626	-409.2545194	-409.0192774
1,4-pentadiene	0.083228	-195.03754	-195.3383345	-195.2551065
pentene	0.089178	-195.066011	-195.3696583	-195.2804803
allylbenzene	0.12729	-348.528867	-349.0343663	-348.9070763
$TMPH_2^+$	0.249696	-408.954354	-409.724686	-409.47499
OTf^-	-0.004824	-960.894362	-961.8289155	-961.8337395
TMSOTf	0.098811	-1084.273138	-1085.37946	-1085.280649
13a	0.162064	-1178.617165	-1179.846779	-1179.684715
13-TMS	0.366195	-1496.521765	-1498.298975	-1497.93278
$BiPh_2OTf$	0.155831	-1429.006554	-1430.528418	-1430.372587
Int-1	0.413034	-1837.585717	-1839.802589	-1839.389555
Int-2	0.398521	-876.55812	-877.9579731	-877.5594521
Int-3	0.499465	-1071.595109	-1073.306395	-1072.80693
Int-3_{TMPH}	0.755037	-1480.175261	-1482.573549	-1481.818512
TS-1	0.753102	-1480.164694	-1482.556564	-1481.803462
Int-4_{TMPH2+}	0.757821	-1480.180981	-1482.587808	-1481.829987
Int-4	0.486624	-1071.205969	-1072.848303	-1072.361679
Int-4_{13a}	0.42208	-1841.238096	-1843.425677	-1843.003597
Ts-2	0.42076	-1841.228152	-1843.414754	-1842.993994
Int-5	0.423792	-1841.258678	-1843.449929	-1843.026137
$BiPh_2$-rad.	0.137087	-468.170124	-468.8085967	-468.6715097
Alkyl rad.	0.070500	-194.429780	-194.716260	-194.6457597
Int-4’	0.231716	-662.62024	-663.5682175	-663.3365015
TS-2^{ins}	0.420174	-1841.143257	-1843.334332	-1842.914158
TS-2^{out}	0.419318	-1841.18585	-1843.391834	-1842.972516
TS-2’$_{out}$	0.419184	-1841.203887	-1843.410749	-1842.991565
Int-4’$_{13a}$	0.421276	-1841.23120	-1843.41740	-1842.996100
TS-2’	0.424506	-1841.2244	-1843.4134	-1842.9889
Int-5’	0.425593	-1841.2562	-1843.4487	-1843.0231
13-TMS’	0.368151	-1496.5392	-1498.3205	-1497.9523
Int-4’$_{II}$	0.231716	-662.62024	-663.5682175	-663.3365015
13a-TMS^+	0.267523	-1301.8452	-1303.3748	-1303.1073
$BiMe_2SbF_6$	0.040164	-689.153171	-690.0498033	-690.0096393
$BiMe_2SbF_6$(pentene)	0.149941	-884.222068	-885.4418722	-885.2919312
$BiMe_2SbF_6$(pentadiene)	0.141988	-884.191135	-885.4052603	-885.2632723
$BiMe_2SbF_6$(allylbenzene)	0.185766	-1037.682773	-1039.102264	-1038.916498
$BiPh_2SbF_6$	0.138291	-1072.207409	-1073.602962	-1073.464671
$BiPh_2SbF_6$(pentene)	0.246974	-1267.278835	-1268.996342	-1268.749368

$BiPh_2SbF_6$(pentadiene)	0.241129	-1267.241143	-1268.959807	-1268.718678
$BiPh_2SbF_6$(allylbenzene)	0.285037	-1420.732243	-1422.656069	-1422.371032
$BiMe_2SbF_6$(allylbenzene)^{arene}	0.188682	-1037.686389	-1039.1032	-1038.9146
$BiPh_2SbF_6$(allylbenzene)^{arene}	0.287222	-1420.741584	-1422.6563	-1422.3691
[$TMPH_2^+$][OTf^-]	0.266407	-1369.992675	-1371.575067	-1371.30866
Et_2O	0.106186	-233.345308	-233.717183	-233.610997
$BF_3 \cdot Et_2O$	0.115497	-557.631179	-558.3947473	-558.2792503
1a	0.065896	-2918.364486	-2919.125909	-2919.060013
TS-2' (1a-TMS)	0.423379	-3704.189297	-3706.220538	-3705.797159
TS-2 (1a-TMS)	0.426663	-3704.195581	-3706.229498	-3705.802835
TS-2'$_{out}$ (1a-TMS)	0.422358	-3704.214935	-3706.243426	-3705.821068
TS-2$_{out}$ (1a-TMS)	0.424329	-3704.207155	-3706.241491	-3705.817162
TS-2' (1a-BF_3)	0.332602	-3905.239013	-3907.363647	-3907.031045
TS-2 (1a-BF_3)	0.334649	-3905.247818	-3907.371648	-3907.036999

Table S2. G_{corr} is the thermal correction to Gibbs Free Energy and $G_{298\ gas\ phase}$ is the sum of electronic and thermal free energies at M06-L+GD3/def2svp$^{(C,H,N,O,S,F)}$/LANL2DZ$^{(Si,Bi)}$ level of theory in gas phase (values in hartree); $E_{0\ DCE}$ is the electronic energies obtained at M06-L+GD3/def2tzvp$^{(C,H,N,O,S,F)}$//LANL2DZ$^{(Si,Bi)}$(SMD)//M06L+GD3/def2svp$^{(C,H,N,O,S,F)}$//LANL2DZ$^{(Si,Bi)}$ level of theory in dichloroethane as solvent; SMD, solvation model based on density (values in hartree); $G_{298\ DCE}$ is the sum of $E_{0\ DCE}$ and G_{corr} (values in hartree).

2.0 Summary of computational study

Our computational study supports: i) the favored formation of an allylbimuth species (detected by HRMS-ESI), which is among the low-energy points in our reaction profile; ii) the C−H deprotonation being involved in a rate determining step, iii) the presence for the formation of branched products over linear ones, and v) a polar reaction being preferred over a radical scenario.

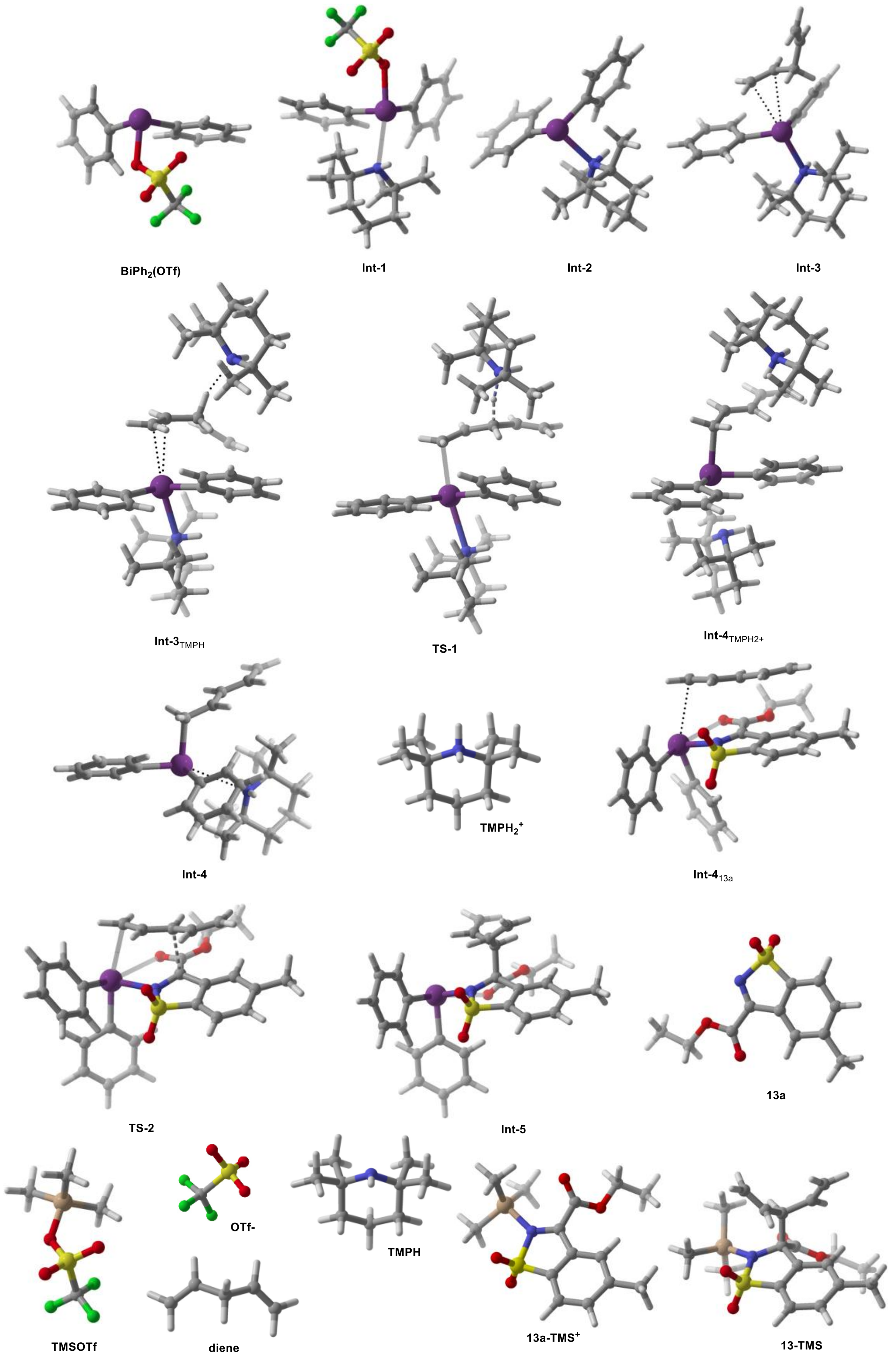

Chart S2. Optimized geometries.

Synthesis of cationic bismuth complexes

1. $[BiPh_2(OTf)]$ (Bi1)

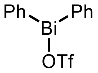

To an oven dried round bottom flask were added $BiPh_3$ (4.40 g, 10.0 mmol, 1.0 equiv.) and anhydrous CH_2Cl_2 (100 mL). The mixture was then cooled to –78 °C, and triflic acid (0.88 mL, 10.0 mmol, 1.0 equiv.) was added dropwise over 5 min. The resulting mixture was stirred at –78 °C for 10 min, and was warmed to room temperature and stirred for 10 h. After completion, the solvent was removed *in vacuo*. The product was obtained (3.10 g, 60% yield) by recrystallization with acetonitrile (10 mL) and diethyl ether (50 mL).

Note: This complex is very moisture sensitive and gradually decomposes under air. If stored in the glovebox, the complex can maintain stable for up to 6 months.

^{1}H NMR (300 MHz, CD_3CN) δ 8.46 (dd, *J* = 8.0, 1.2 Hz, 4H), 7.89 – 7.80 (m, 4H), 7.56 – 7.47 (m, 2H).
^{13}C NMR (75 MHz, CD_3CN) δ 139.2, 133.6, 130.1.
^{19}F NMR (282 MHz, CD_3CN) δ –78.92.
HRMS (ESI) calcd for $C_{12}H_{10}Bi$ $[M–OTf]^+$: 363.0581, found: 363.0564.

2. 5*H*-Dibenzo[*b*,*d*]bismol-5-yl 4-methylbenzenesulfonate (Bi2)

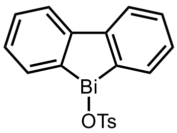

This complex was synthesized according to the following procedure.

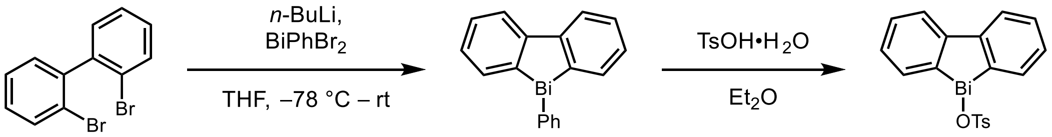

To the solution of 2,2'-dibromobiphenyl (1.08 g, 3.5 mmol, 1.0 equiv.) in anhydrous THF (15 mL) was added *n*-BuLi (2.8 mL, 7.0 mmol, 2.0 equiv., 2.5 M in hexanes) dropwise over 2 min, the mixture was stirred at –78 °C for 1 h. Then a solution of $BiPhBr_2$ (1.54 g, 3.5 mmol, 1.0 equiv.) in anhydrous THF (20 mL) was added at –78 °C. The resulting mixture was allowed to warm to room temperature and stirred overnight. After completion, the mixture was quenched with brine (10 mL) and extracted with EtOAc (2 × 20 mL). The combined organic layers were dried over magnesium sulfate and the solvent was removed by rotary evaporation. The crude residue was purified by column chromatography (gradient elution from 100% hexanes to 50:1 hexanes/EtOAc) to obtain compound 9-phenylbismafluorene as a white solid (521 mg, 34% yield). ^{1}H NMR spectroscopic data were in agreement with the literature.[19]

$TsOH·H_2O$ (226 mg, 1.2 mmol, 1.0 equiv.) was added to the suspension of 9-phenylbismafluorene (521 mg, 1.2 mmol, 1.0 equiv.) in Et_2O (20 mL), the resulting mixture was stirred at room temperature for 16 h. Large amounts of white chunks were precipitated out during the reaction, and the product was collected by vacuum filtration as a white solid (602 mg, 95% yield).

Note: This compound is poorly soluble in non-coordinating solvents, while in coordinating solvents it gradually decomposes, albeit with good solubility. Therefore, this compound was difficult to characterize by NMR spectroscopy and the obtained spectra contained some unidentified impurities.

^{1}H NMR (300 MHz, CD_3OD) δ 8.45 (dd, *J* = 7.2, 1.4 Hz, 2H), 8.28 (dd, *J* = 7.9, 1.1 Hz, 2H), 7.84 (td, *J* = 7.3, 1.2 Hz, 2H), 7.72 – 7.58 (m, 4H), 7.21 (d, *J* = 7.9 Hz, 2H), 2.36 (s, 3H).
^{13}C NMR (100 MHz, CD_3OD) δ 202.8, 164.8, 143.0, 141.9, 138.1, 133.4, 131.8, 131.3, 129.8, 128.8, 127.0, 21.3.
HRMS (ESI) calcd for $C_{19}H_{16}O_3BiS$ $[M+H]^+$: 533.0618, found: 533.0620.
Elemental analysis: Anal. calc. for: [$C_{19}H_{16}O_3BiS$] (532.37 g/mol): C 42.87, H 2.84, found: C 42.41, H 2.85.

3. [$BiPh_2(SbF_6)$]

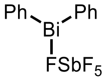

To a solution of $BiPh_2Cl$ (50.0 mg, 0.13 mmol) in *o*-difluorobenzene (2 mL) was added a solution of $AgSbF_6$ (43.1 mg, 0.13 mmol) in *o*-difluorobenzene (2 mL). The reaction mixture was stirred for 2 h, then the precipitate was filtered off and the light yellow solution was layered with *n*-pentane. After being stored at –30 °C for 16 h, a yellow solid was obtained after filtration and drying *in vacuo*. Yield: 570 mg, 0.10 mmol, 77%.

The compound decomposes to benzene in the following circumstances: 1) in noncoordinating solvents (e.g. CD_2Cl_2); 2) exposed to vacuum for long time (>2 h, even with silanized glass). In solution, when exposed to light, the compound decomposes to biphenyl (~20% decomposition after 6 h). The compound is pure and stable in its solid form and in coordinating solvents (e.g. acetonitrile). The characterization data were reported using CD_2Cl_2 as the deuterated solvent, which is consistent with that used in mechanistic studies.

^{1}H NMR (400 MHz, CD_2Cl_2): δ = 7.63 (t, 2H, $^{3}J_{HH}$ = 7.3 Hz, *para*-C_6H_5), 8.08 (t, 4H, $^{3}J_{HH}$ = 7.4 Hz, *meta*-C_6H_5), 8.55 (d, 4H, $^{3}J_{HH}$ = 7.5 Hz, *ortho*-C_6H_5) ppm.
^{13}C NMR (101 MHz, CD_2Cl_2): δ = 131.34 (s, *para*-C_6H_5), 134.79 (s, *meta*-C_6H_5), 138.76 (s, *ortho*-C_6H_5), 214.76 (s, *ipso*-C_6H_5, detected by 2D-^{1}H-^{13}C-HMBC experiments) ppm.
^{19}F NMR (376 MHz, CD_2Cl_2): δ = –122.16 (s) ppm.
Elemental analysis: Anal. calc. for: [$C_{12}H_{10}BiSbF_6$] (597.95 g/mol): C 24.06, H 1.68, found: C 23.98, H 1.75.

Single crystals for X-ray diffraction analysis of [$BiPh_2(SbF_6)$] were obtained by layering a solution of [$BiPh_2(SbF_6)$] in CH_2Cl_2 with *n*-pentane and storing at –30 °C for 32 h. A suitable crystal was coated with perfluorinated polyether oil in a glovebox, transferred to a nylon loop and then to a Bruker D8 Quest diffractometer. The crystal was kept at 100.00 K during data collection. Using Olex2,[20] the structure was solved with the XS solution program[21] using Direct Methods and refined with the XL package[21] using Least Squares minimization.

Crystal Data for [$C_{13}H_{12}BiCl_2F_6Sb$] (*M* = 683.86 g/mol): monoclinic, space group $P2_1/c$ (no. 14), a = 10.3042(13) Å, b = 33.691(7) Å, c = 10.3351(18) Å, β = 90.251(15)°, V = 3587.9(11) Å^3, Z = 8, T = 100.00 K, μ(MoKα) = 11.649 mm^{-1}, *Dcalc* = 2.532 g/cm^3,

100915 reflections measured ($3.94° \leq 2\Theta \leq 57.468°$), 9276 unique ($R_{int}$ = 0.0437, R_{sigma} = 0.0224) which were used in all calculations. The final R_1 was 0.0484 ($I > 2\sigma(I)$) and wR_2 was 0.1064 (all data).

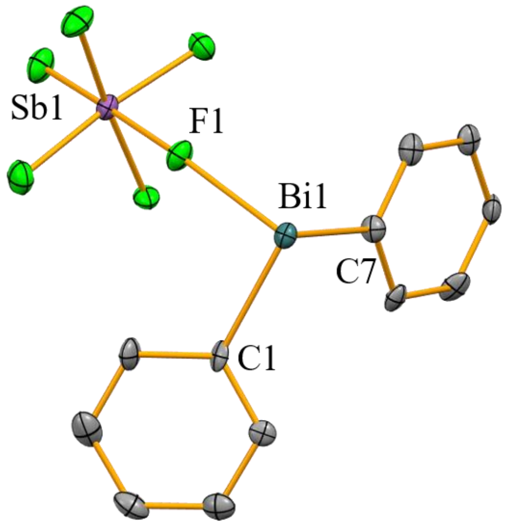

Figure S43. Crystal structure of [$BiPh_2(SbF_6)$]. Displacement ellipsoids are drawn at 50% probability level. Hydrogen atoms and the second molecule inside the asymmetric unit are omitted for clarity. Selected bond lengths [Å] and angles [°]: Bi1–C1 2.241(10), Bi1–C7 2.236(10), Bi1–F1 2.441(6), C1–Bi1–C7 97.1(4), C1–Bi1–F1 89.3(3), C7–Bi1–F1 89.1(3).

[$BiPh_2(SbF_6)$] crystallizes in the monoclinic space group $P2_1/c$ with two molecules in the asymmetric unit. The central bismuth atom in both molecules shows a distorted square pyramidal coordination geometry (τ_5 = 0.21). The apical position is occupied by one phenyl ring, while the second phenyl ring, two fluorine atoms of two SbF_6 units, and one phenyl ring of the second molecule of [$BiPh_2(SbF_6)$] in η^6-mode are in the basal positions. The distance between the bismuth atom and the centroid of the η^6-phenyl ligand is 3.344(10) Å, which is in line with known bismuth–arene compounds with a bismuth–arene distance of 2.6-3.8 Å.[22] The bismuth–carbon bond lengths are 2.236(10)-2.241(10) Å which are only slightly shorter than that in the parent compound $BiPh_3$ (2.247(2)-2.257(2) Å).[23] The bismuth–fluorine distances are identical within limits of error (2.441(6)-2.456(6) Å), so the counterions cannot be directly ascribed to one cation, but are rather shared among two cations. In the related compound [$BiMe_2(SbF_6)$], a similar behavior is observed with bismuth–fluorine distances of 2.451(3) Å and 2.452(3) Å.[24] This leads to the formation of a 1D coordination polymer in the solid state. Since the coordinating fluorine atoms of the SbF_6 moiety are in *cis*-position the polymeric chain with a zig-zag structure, which is also supported by the secondary bismuth-arene interaction. The carbon-bismuth-carbon angles and the carbon-bismuth fluorine angles are very close to, due to s-p-separation, ideal 90 ° with 85.4(3)-97.1(4) °.

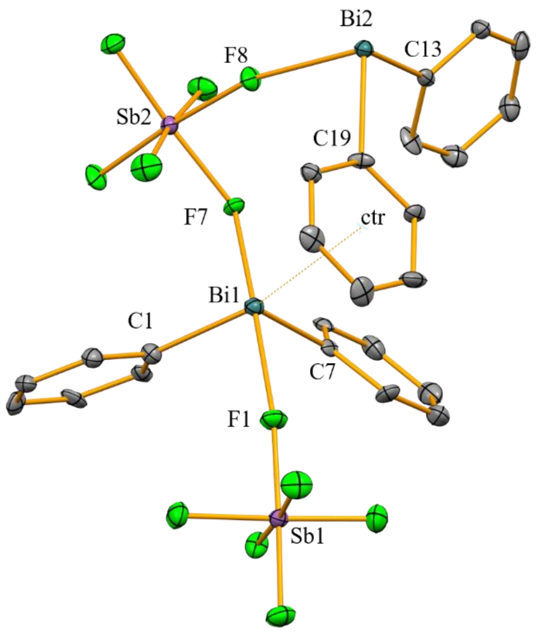

Figure S44. Crystal structure of [$BiPh_2(SbF_6)$]. Displacement ellipsoids are drawn at 50% probability level. Hydrogen atoms are omitted for clarity. Selected bond lengths [Å] and angles [°]: Bi1–C1 2.241(10), Bi1–C7 2.236(10), Bi1–F1 2.441(6) Bi1–F7 2.456(6), Bi1–ctr 3.344(10), Bi2–C13 2.212(10), Bi2–C19 2.230(9), Bi2–F8 2.448(6), Bi2–F2 2.488(6), C1–Bi1–C7 97.1(4), C1–Bi1–F1 89.3(3), C7–Bi1–F1 89.1(3), F1–Bi1–F7 170.3(2), C13–Bi2–C19 96.7(4), C13–Bi2–F8 89.6(3), C19–Bi2–F2 85.9(3), F2–Bi2–F8 167.4(2).

[**Bi3**][25] and [**Bi4**][26] were synthesized according to literature procedures.

Procedures for substrate synthesis and characterization data

N-Sulfonyl ketimines **12a–22a** are known compounds and were synthesized according to modified literature procedures.[27-29]

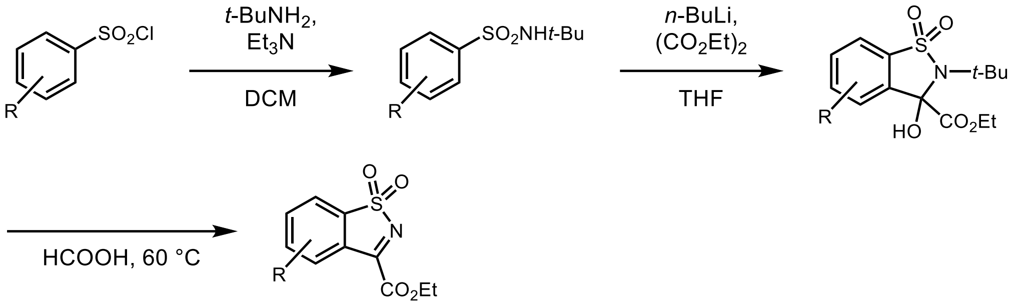

General procedure B1: Arylsulfonyl chloride (10 mmol) was added to a solution of *tert*-butylamine (1.6 mL, 15 mmol, 1.5 equiv.) and triethylamine (2.8 mL, 20 mmol, 2.0 equiv.) in CH_2Cl_2 (20 mL) at 0 °C. The mixture was stirred at room temperature overnight. After completion, the mixture was concentrated *in vacuo* to remove excess amines, and was diluted with CH_2Cl_2 (20 mL), washed with 0.1 N HCl (2 × 5 mL) and brine (3 × 5 mL). The organic layer was dried over magnesium sulfate, concentrated *in vacuo* to give the aryl sulfonamide as a solid.

n-Butyllithium (4.8 mL, 12 mmol, 2.0 equiv., 2.5 M in hexane) was added dropwise over 5 min to the solution of aryl sulfonamide (6 mmol) in anhydrous THF (20 mL) at 0 °C under nitrogen. After stirring at 0 °C for 30 min, the mixture was cooled further to –78 °C and diethyl oxalate (2.4 mL, 18 mmol, 3.0 equiv.) was added. The mixture was allowed to stir at room temperature for 2 h. After completion, the mixture was quenched with 1 N HCl (10 mL) and extracted with ethyl acetate (2 × 5 mL). The organic layers were dried over magnesium sulfate and concentrated *in vacuo*. The crude residue was purified by flash column chromatography.

To the product obtained above, formic acid (8 mL) was added and the mixture was stirred at 60 °C under nitrogen for 12 h. Formic acid was removed *in vacuo* and the resultant solid was dissolved in CH_2Cl_2 and concentrated to remove traces of formic acid. The crude residue was purified by flash column chromatography followed by recrystallization with EtOAc/hexanes to afford the desired product as a solid.

1. Ethyl 6-methylbenzo[d]isothiazole-3-carboxylate 1,1-dioxide (20a, wrh-07-167A)

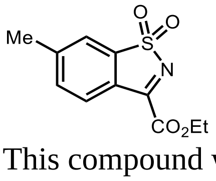

This compound was obtained as a white solid according to general procedure B1.
^{1}H NMR (400 MHz, $CDCl_3$) δ 8.14 (d, J = 7.9 Hz, 1H), 7.74 (s, 1H), 7.54 (d, J = 8.0 Hz, 1H), 4.53 (q, J = 7.1 Hz, 2H), 2.54 (s, 3H), 1.47 (t, J = 7.1 Hz, 3H).
^{13}C NMR (100 MHz, $CDCl_3$) δ 160.7, 160.3, 146.4, 140.9, 135.0, 127.4, 126.1, 123.8, 63.8, 22.0, 14.1.
HRMS (ESI) calcd for $C_{11}H_{12}O_4NS$ $[M+H]^+$: 254.0482, found: 254.0479.
m.p. = 111 – 112 °C

α-Keto esters **7a**–**10a** were synthesized according to the following prodedure.

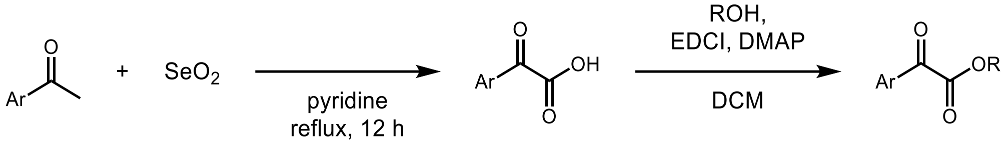

General procedure B2: Under inert atmosphere, the mixture of selenium dioxide (1.11 g, 10 mmol, 2.0 equiv.) and arylethanone (5.0 mmol, 1.0 equiv.) in pyridine (5 mL) was heated at reflux for 12 h. Then the reaction mixture was filtered through a celite pad and the filtrate was concentrated to give the crude product. The resulting residue was treated with 2 N NaOH (10 mL) and then washed with EtOAc (20 mL). The aqueous layer was acidified to pH = 1 with 6 N HCl, then extracted with EtOAc (2 × 20 mL). The combined organic layers were dried over magnesium sulfate and concentrated to give the 2-oxoacetic acid, which was used in the next step without further purification.

The 2-oxoacetic acid obtained in the last step, alcohol (1.0 equiv.) and DMAP (30.5 mg, 5 mol%) were dissolved in CH_2Cl_2 (10 mL), the resulting solution was cooled in ice bath. EDCI (1.15 g, 6.0 mmol, 1.2 equiv.) was added to the solution at 0 °C. The mixture was stirred at room temperature overnight. Then the mixture was washed with H_2O (3 × 10 mL). The organic layer was dried over magnesium sulfate and the solvent was removed under reduced pressure. The crude residue was purified by column chromatography to afford the products.

2. Ethyl 2-(4-bromophenyl)-2-oxoacetate (7a, wrh-08-65B)

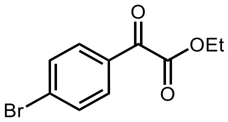

^{1}H NMR (300 MHz, $CDCl_3$) δ 7.96 – 7.85 (m, 2H), 7.71 – 7.61 (m, 2H), 4.45 (q, *J* = 7.2 Hz, 2H), 1.42 (t, *J* = 7.2 Hz, 3H).
^{1}H NMR spectroscopic data were in agreement with the literature.[30]

3. Ethyl 2-oxo-2-(4-(trifluoromethoxy)phenyl)acetate (8a, wrh-08-67A)

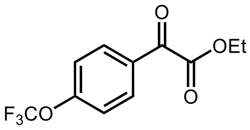

This compound was synthesized as a pale yellow oil according to general procedure B2.
^{1}H NMR (300 MHz, $CDCl_3$) δ 8.17 – 8.05 (m, 2H), 7.37 – 7.30 (m, 2H), 4.46 (q, *J* = 7.2 Hz, 2H), 1.43 (t, *J* = 7.1 Hz, 3H).
^{13}C NMR (76 MHz, $CDCl_3$) δ 184.6, 163.2, 154.0 (q, *J* = 1.8 Hz), 132.4, 130.9, 120.6, 120.4 (q, *J* = 261 Hz), 62.7, 14.2.
^{19}F NMR (282 MHz, $CDCl_3$) δ –57.55.
HRMS (ESI) calcd for $C_{11}H_{10}O_4F_3$ $[M+H]^+$: 263.0526, found: 263.0523.

4. Benzyl 2-oxo-2-phenylacetate (9a, wrh-08-OBn)

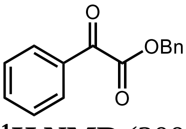

^{1}H NMR (300 MHz, $CDCl_3$) δ 8.03 – 7.91 (m, 2H), 7.65 (ddt, *J* = 7.9, 6.9, 1.3 Hz, 1H), 7.56 – 7.31 (m, 7H), 5.42 (s, 2H).

^{1}H NMR spectroscopic data were in agreement with the literature.[31]

5. (1*R*,3*S*,5*r*,7*r*)-Adamantan-2-yl 2-(4-bromophenyl)-2-oxoacetate (10a, wrh-08-104)

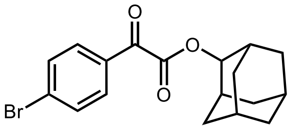

^{1}H NMR (300 MHz, $CDCl_3$) δ 7.93 – 7.83 (m, 2H), 7.72 – 7.62 (m, 2H), 5.28 – 5.24 (m, 1H), 2.21 – 2.13 (m, 2H), 2.09 – 1.99 (m, 2H), 1.98 – 1.74 (m, 8H), 1.66 – 1.56 (m, 2H).
^{1}H NMR spectroscopic data were in agreement with the literature.[32]

Allylarenes **25b**, **26b**, **29b** are commercially available, **27b**,[33] **28b**,[34] **30b**,[34] **32b**–**33b**,[34] **34b**,[35] **35b**[34] were synthesized according to literature procedures.

6. 4-Allyl-1-tosyl-1*H*-indole (32b, wrh-08-27-1)

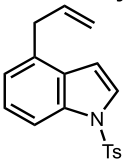

This compound was obtained as a white waxy solid.
^{1}H NMR (300 MHz, $CDCl_3$) δ 7.86 (d, *J* = 8.3 Hz, 1H), 7.82 – 7.69 (m, 2H), 7.56 (d, *J* = 3.7 Hz, 1H), 7.29 – 7.16 (m, 3H), 7.07 – 7.01 (m, 1H), 6.71 (dd, *J* = 3.7, 0.9 Hz, 1H), 6.07 – 5.88 (m, 1H), 5.14 – 5.01 (m, 2H), 3.57 (dt, *J* = 6.6, 1.6 Hz, 2H), 2.34 (s, 3H).
^{13}C NMR (76 MHz, $CDCl_3$) δ 145.0, 136.6, 135.5, 134.9, 133.0, 130.1, 130.0, 127.0, 126.0, 124.8, 123.1, 116.3, 111.8, 107.3, 37.6, 21.7.
HRMS (ESI) calcd for $C_{18}H_{18}O_2NS$ $[M+H]^+$: 312.1053, found: 312.1046.

7. 4-(4,4,5,5-Tetraethyl-1,3,2-dioxaborolan-2-yl)phenyl 4-allylbenzoate (31b, czz-03-71-2)

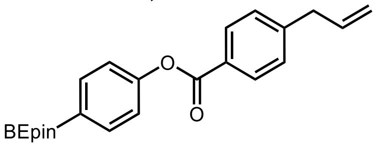

This compound was synthesized according to the following procedure.

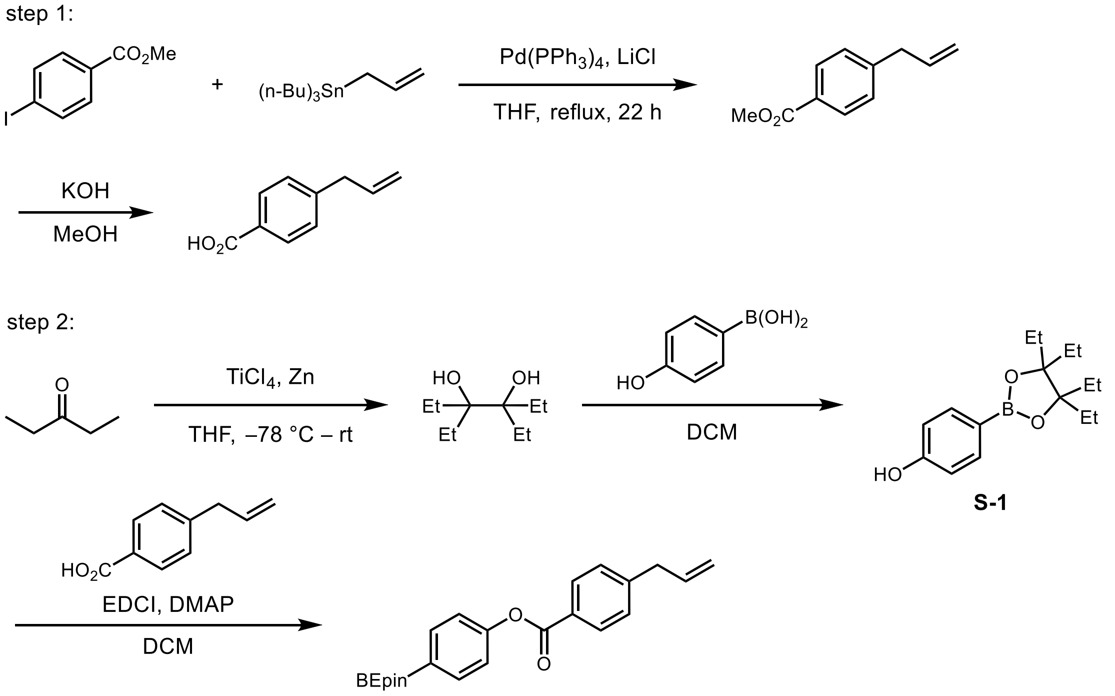

Step 1: A 250 mL three-necked round bottom flask equipped with a reflux condenser was charged with methyl 4-iodobenzoate (2.62 g, 10 mmol, 1.0 equiv.), $Pd(PPh_3)_4$ (1.16 g, 1.0 mmol, 10 mol%), LiCl (2.12 g, 50 mmol, 5.0 equiv.), and anhydrous THF (75 mL) under a flow of nitrogen. Then allyltributylstanane (3.3 mL, 10.5 mmol, 1.05 equiv.) was added to the resulting mixture. After degassing under nitrogen for 15 min, the mixture was heated at reflux (80 °C) for 22 h. The reaction mixture was allowed to cool to room temperature and was quenched with saturated KF solution (100 mL). After stirring at room temperature for 1 h, the insoluble solid materials were filtered out through a pad of Celite. Then the mixture was extracted with EtOAc (2 × 30 mL), the combined organic layers were dried over magnesium sulfate and the solvent was removed by rotary evaporation. The crude residue was purified by column chromatography (30:1 hexanes/EtOAc) to afford methyl 4-allylbenzoate as a colorless oil (1.50 g, 84% yield). ^1H NMR spectroscopic data were in agreement with the literature.[36]

A 25 mL round bottom flask was charged with methyl 4-allylbenzoate (1.37 g, 7.8 mmol, 1.0 equiv.), KOH (1.31 g, 23 mmol, 3.0 equiv.) and MeOH (5 mL). The reaction mixture was stirred at room temperature for 2 h. Then H_2O (6 mL) was added to dilute the mixture, and 6 N HCl was added dropwise until the pH reached 1–2. The resulting mixture was extracted with Et_2O (3 × 10 mL), the combined organic layers were dried over magnesium sulfate and the solvent was removed by rotary evaporation. The crude residue was purified by column chromatography (30:1 CH_2Cl_2/MeOH) to afford 4-allylbenzoic acid as a white solid (950 mg, 75% yield). ^1H NMR spectroscopic data were in agreement with the literature.[37]

Step 2: An oven-dried 250 mL round bottom flask was charged with diethyl ketone (2.1 mL, 20 mmol, 1.0 equiv.) and anhydrous THF (50 mL) under nitrogen atmosphere. Then the mixture was cooled to –78 °C, $TiCl_4$ (3.3 mL, 30 mmol, 1.5 equiv.) was added dropwise over 2 min. After stirring at –78 °C for 30 min, Zn powder (3.92 g, 60 mmol, 3.0 equiv.) was added, and the reaction mixture was stirred at reflux (72 °C) for 3 h. After completion, the mixture was quenched by slow addition of saturated K_2CO_3

solution (25 mL) at 0 °C and was stirred for 30 min. The resulting mixture was filtered through a pad of Celite, and was extracted with EtOAc (2 × 30 mL). The combined organic layers were dried over magnesium sulfate and the solvent was removed by rotary evaporation. The crude product was used in the next step without purification.

A 250 mL round bottom flask was charged with (4-hydroxyphenyl) boronic acid (1.16 g, 8.4 mmol, 1.0 equiv.), Epin (1.47 g, 8.4 mmol, 1.0 equiv.) and anhydrous CH_2Cl_2 (85 mL) under N_2 atmosphere. The reaction mixture was stirred at room temperature for 16 h. Then the mixture was quenched with H_2O (50 mL), and was extracted with CH_2Cl_2 (2 × 50 mL). The combined organic layers were dried over magnesium sulfate and the solvent was removed by rotary evaporation. The crude residue was purified by column chromatography (5:1 hexanes/EtOAc) to afford compound **S-1** as a yellow oil (1.30 g, 56% yield).
^{1}H NMR (300 MHz, $CDCl_3$) δ 7.78 – 7.67 (m, 2H), 6.87 – 6.76 (m, 2H), 5.44 (s, 1H), 1.90 – 1.60 (m, 8H), 0.97 (t, *J* = 7.5 Hz, 12H).
^{13}C NMR (76 MHz, $CDCl_3$) δ 158.4, 136.9, 114.9, 88.8, 77.4, 26.6, 9.0.
HRMS (ESI) calcd for $C_{16}H_{24}O_3B$ $[M–H]^-$: 275.1824, found: 275.1820.

Compound **S-1** (552 mg, 2.0 mmol, 1.0 equiv.), 4-allylbenzoic acid (324 mg, 2.0 mmol, 1.0 equiv.) obtained in *step 1*, DMAP (12.2 mg, 0.1 mmol, 5 mol%) were dissolved in CH_2Cl_2 (7 mL), the resulting solution was cooled in ice bath. Then EDCI (460 mg, 2.4 mmol, 1.2 equiv.) was added to the mixture at 0 °C. The mixture was stirred at room temperature overnight. Then the mixture was washed with brine (3 × 10 mL), the organic layer was dried over magnesium sulfate and the solvent was removed under reduced pressure. The crude residue was purified by column chromatography (40:1 hexanes/EtOAc) to afford the product as a yellow oil (597 mg, 71% yield).
^{1}H NMR (300 MHz, $CDCl_3$) δ 8.19 – 8.08 (m, 2H), 7.96 – 7.85 (m, 2H), 7.34 (d, *J* = 8.3 Hz, 2H), 7.22 (d, *J* = 8.4 Hz, 2H), 5.99 (ddt, *J* = 16.2, 10.8, 6.7 Hz, 1H), 5.19 – 5.06 (m, 2H), 3.49 (d, *J* = 6.7 Hz, 2H), 1.89 – 1.66 (m, 8H), 0.98 (t, *J* = 7.4 Hz, 12H).
^{13}C NMR (76 MHz, $CDCl_3$) δ 165.1, 153.6, 146.5, 136.4, 130.7, 130.5, 129.0, 127.6, 125.9, 121.2, 116.9, 89.0, 40.3, 26.6, 9.0.
HRMS (ESI) calcd for $C_{26}H_{34}O_4B$ $[M+H]^+$: 421.2545, found: 421.2538.

Skipped dienes **38b**–**41b**[38], **42b**[39] were synthesized according to literature procedures.

8. (*E*)-9-Chloronona-1,4-diene (38b, wrh-08-Cl)

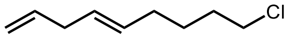

This compound was obtained as a colorless liquid.
^{1}H NMR (300 MHz, $CDCl_3$) δ 5.82 (ddt, *J* = 17.2, 10.1, 6.4 Hz, 1H), 5.54 – 5.34 (m, 2H), 5.09 – 4.93 (m, 2H), 3.53 (t, *J* = 6.7 Hz, 2H), 2.83 – 2.65 (m, 2H), 2.11 – 1.98 (m, 2H), 1.86 – 1.70 (m, 2H), 1.59 – 1.43 (m, 2H).
^{13}C NMR (76 MHz, $CDCl_3$) δ 137.4, 130.9, 128.5, 115.0, 45.1, 36.8, 32.2, 31.9, 26.8.
HRMS (ESI) calcd for $C_9H_{14}Cl$ $[M–H]^+$: 157.0779, found: 157.0778.

9. (*E*)-Octa-4,7-dien-1-yl benzoate (42b, wrh-08-OPh)

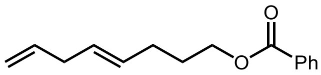

This compound was obtained as a colorless oil.
^{1}H NMR (300 MHz, $CDCl_3$) δ 8.10 – 7.99 (m, 2H), 7.62 – 7.50 (m, 1H), 7.50 – 7.38 (m, 2H), 5.82 (ddt, *J* = 16.7, 10.1, 6.4 Hz, 1H), 5.59 – 5.40 (m, 2H), 5.09 – 4.93 (m,

2H), 4.33 (t, J = 6.5 Hz, 2H), 2.82 – 2.69 (m, 2H), 2.27 – 2.11 (m, 2H), 1.85 (dq, J = 8.5, 6.7 Hz, 2H).
^{13}C NMR (76 MHz, $CDCl_3$) δ 166.8, 137.3, 133.0, 130.6, 130.2, 129.7, 129.0, 128.5, 115.1, 64.6, 36.8, 29.1, 28.6.
HRMS (ESI) calcd for $C_{15}H_{19}O_2$ $[M+H]^+$: 231.1380, found: 231.1377.

X-ray structures

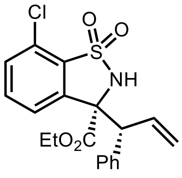

25

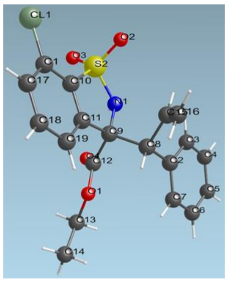

Table S3. Sample and crystal data for Ruihan102023.

**Identification code**	Ruihan102023	
**Chemical formula**	$C_{19}H_{18}ClNO_4S$	
**Formula weight**	391.85 g/mol	
**Temperature**	100(2) K	
**Wavelength**	1.54178 Å	
**Crystal system**	orthorhombic	
**Space group**	P c a 21	
**Unit cell dimensions**	a = 14.1801(3) Å	α = 90°
	b = 9.5824(2) Å	β = 90°
	c = 14.0519(3) Å	γ = 90°
**Volume**	1909.36(7) $Å^3$	
**Z**	4	
**Density (calculated)**	1.363 g/cm^3	
**Absorption coefficient**	3.000 mm^{-1}	
**F(000)**	816	

Table S4. Data collection and structure refinement for Ruihan102023.

**Diffractometer**	Bruker Photon III CPAD Detector
**Radiation source**	Bruker D8 Venture Duo (CuKα, λ = 1.54178 Å)
**Theta range for data collection**	4.61 to 72.25°
**Index ranges**	-17<=h<=17, -11<=k<=11, -17<=l<=16
**Reflections collected**	43594
**Independent reflections**	3750 [R(int) = 0.0640]
**Absorption correction**	multi-scan
**Structure solution technique**	direct methods
**Structure solution program**	SHELXT 2014/5 (Sheldrick, 2014)
**Refinement method**	Full-matrix least-squares on F^2
**Refinement program**	SHELXL-2017/1 (Sheldrick, 2017)
**Function minimized**	$\Sigma w(F_o^2 - F_c^2)^2$

**Data / restraints / parameters**	3750 / 1 / 250	
**Goodness-of-fit on F^2**	1.039	
**Δ/σ_{max}**	0.011	
**Final R indices**	3564 data; $I>2\sigma(I)$	R1 = 0.0212, wR2 = 0.0460
	all data	R1 = 0.0236, wR2 = 0.0468
**Weighting scheme**	$w=1/[\sigma^2(F_o^2)+(0.0188P)^2+0.2037P]$<br>where $P=(F_o^2+2F_c^2)/3$	
**Absolute structure parameter**	0.008(12)	
**Extinction coefficient**	0.0005(1)	
**Largest diff. peak and hole**	0.173 and -0.150 eÅ^{-3}	
**R.M.S. deviation from mean**	0.030 eÅ^{-3}	

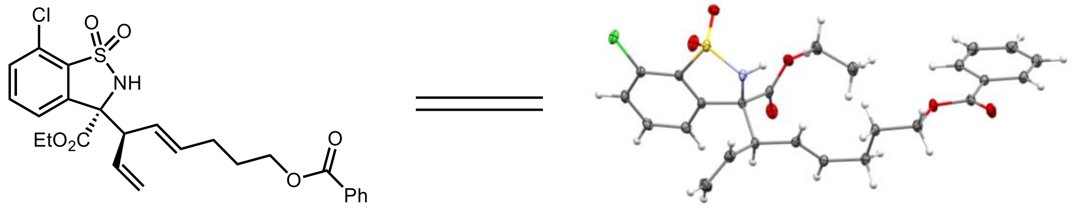

42

Table S5. Sample and crystal data for Ruihan32124.

**Identification code**	Ruihan32124
**Chemical formula**	$C_{25}H_{26}ClNO_6S$
**Formula weight**	503.98 g/mol
**Temperature**	108(2) K
**Wavelength**	0.71073 Å
**Crystal size**	0.040 x 0.080 x 0.130 mm
**Crystal habit**	clear colourless prism
**Crystal system**	triclinic
**Space group**	P -1
**Unit cell dimensions**	a = 8.8672(5) Å α = 78.972(2)°
	b = 14.6366(8) Å β = 79.347(2)°
	c = 19.0659(9) Å γ = 79.765(2)°
**Volume**	2360.8(2) $Å^3$
**Z**	4
**Density (calculated)**	1.418 g/cm^3
**Absorption coefficient**	0.293 mm^{-1}
**F(000)**	1056

Table S6. Data collection and structure refinement for Ruihan32124.

**Diffractometer**	Bruker Venture Duo ImuS
**Radiation source**	microfocus Bruker Photon III CPAD (λ = 0.71073 Å)
**Theta range for data collection**	1.95 to 28.35°
**Index ranges**	-11<=h<=11, -19<=k<=19, -25<=l<=25
**Reflections collected**	113472
**Independent reflections**	11757 [R(int) = 0.0932]
**Coverage of independent reflections**	99.8%
**Absorption correction**	multi-scan
**Max. and min. transmission**	0.9880 and 0.9630
**Refinement method**	Full-matrix least-squares on F^2

**Refinement program**	SHELXL-2019/1 (Sheldrick, 2019)	
**Function minimized**	Σ w(F_o^2 - F_c^2)^2	
**Data / restraints / parameters**	11757 / 0 / 621	
**Goodness-of-fit on F^2**	1.028	
**Final R indices**	8748 data; I>2σ(I)	R1 = 0.0590, wR2 = 0.1436
	all data	R1 = 0.0842, wR2 = 0.1590
**Weighting scheme**	w=1/[$\sigma^2(F_o^2)$+(0.0729P)^2+2.8421P] where P=(F_o^2+2F_c^2)/3	
**Largest diff. peak and hole**	1.126 and -0.495 eÅ^{-3}	
**R.M.S. deviation from mean**	0.085 eÅ^{-3}	

[BiPh$_2$(SbF$_6$)] ═══ 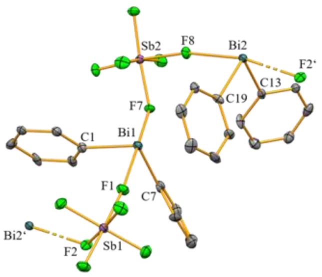

Table S7. Crystal data and structure refinement for JSX31.

**Identification code**	JSX31
**Empirical formula**	$C_{13}H_{12}BiCl_2F_6Sb$
**Formula weight**	683.86
**Temperature/K**	100.00
**Crystal system**	monoclinic
**Space group**	$P2_1/c$
**a/Å**	10.3042(13)
**b/Å**	33.691(7)
**c/Å**	10.3351(18)
**α/°**	90
**β/°**	90.251(15)
**γ/°**	90
**Volume/Å^3**	3587.9(11)
**Z**	8
**ρ_{calc}g/cm^3**	2.532
**μ/mm^{-1}**	11.649
**F(000)**	2496.0
**Crystal size/mm^3**	0.233 × 0.098 × 0.078
**Radiation**	MoKα (λ = 0.71073)
**2Θ range for data collection/°**	3.94 to 57.468
**Index ranges**	$-13 \leq h \leq 13$, $-45 \leq k \leq 45$, $-13 \leq l \leq 13$
**Reflections collected**	100915
**Independent reflections**	9276 [R_{int} = 0.0437, R_{sigma} = 0.0224]
**Data/restraints/parameters**	9276/6/417
**Goodness-of-fit on F^2**	1.360
**Final R indexes [I>=2σ (I)]**	R_1 = 0.0484, wR_2 = 0.1051
**Final R indexes [all data]**	R_1 = 0.0526, wR_2 = 0.1064
**Largest diff. peak/hole / e Å^{-3}**	2.53/-2.62

Copies of NMR spectra

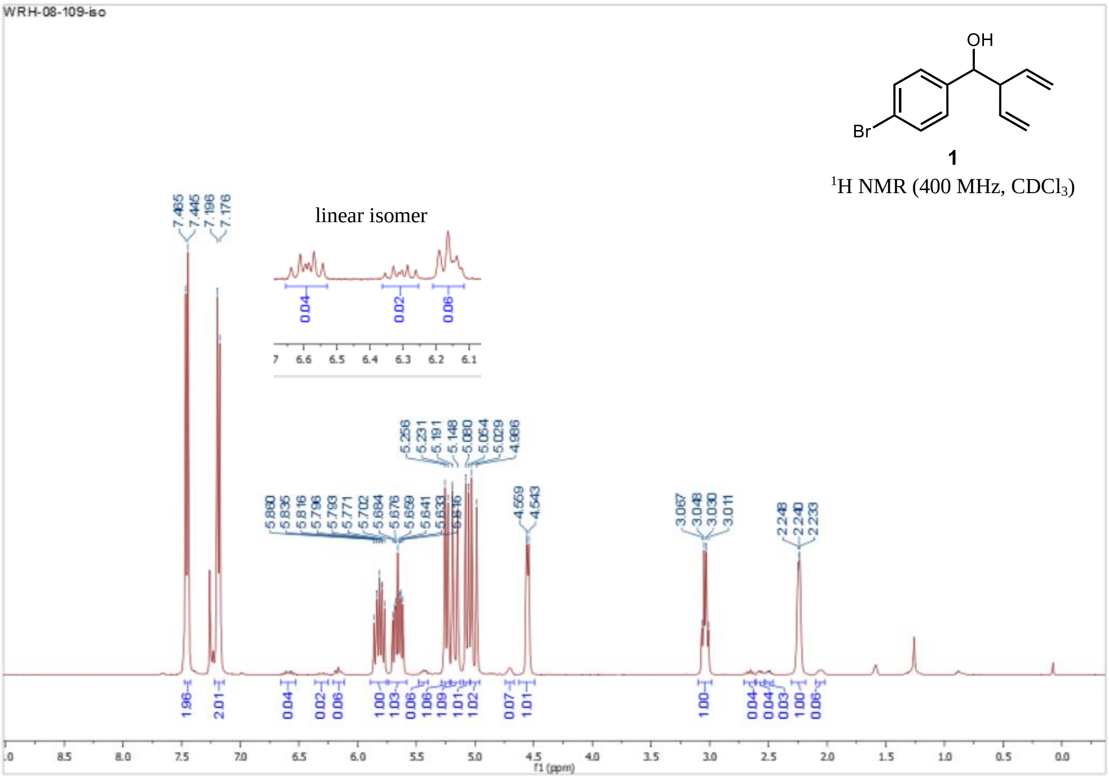

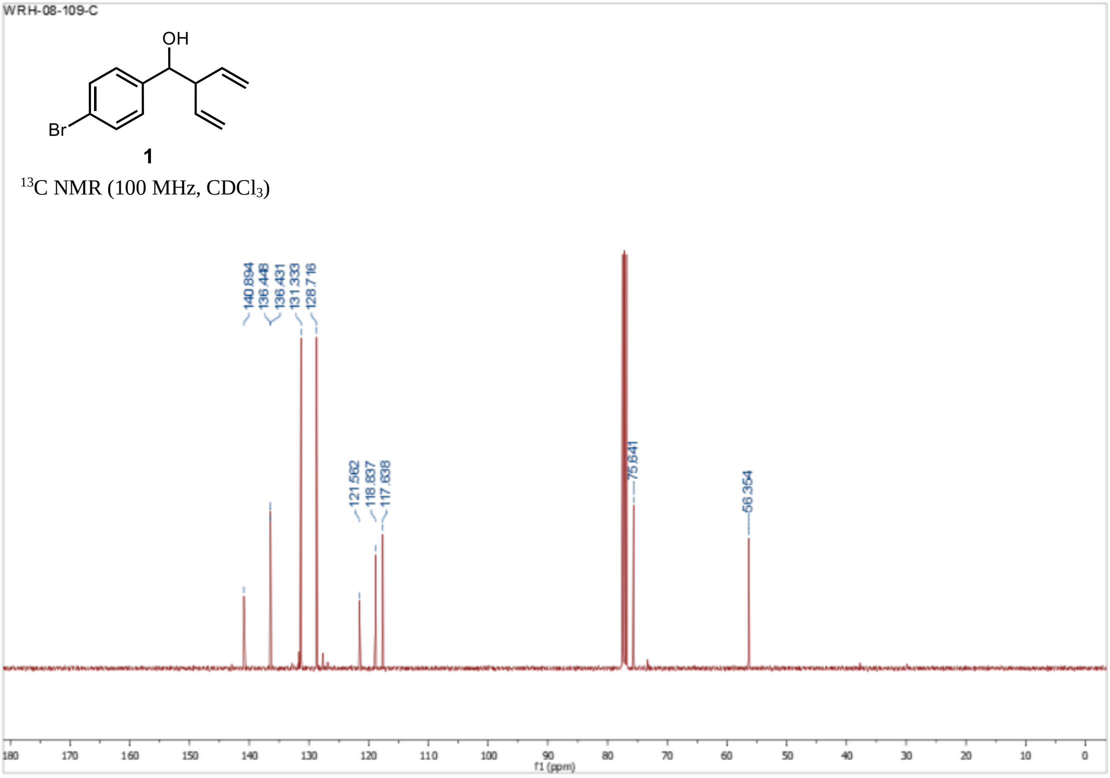

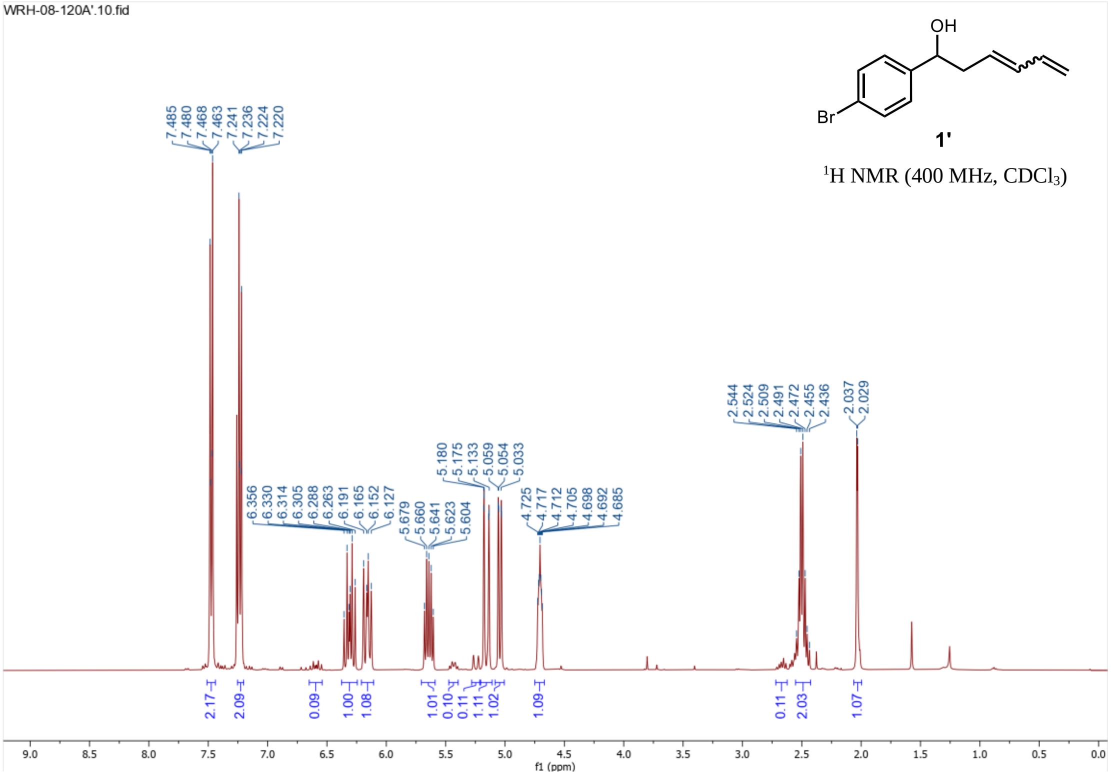
WRH-08-120A'.10.fid
OH
Br
1'
1H NMR (400 MHz, CDCl3)
f1 (ppm)

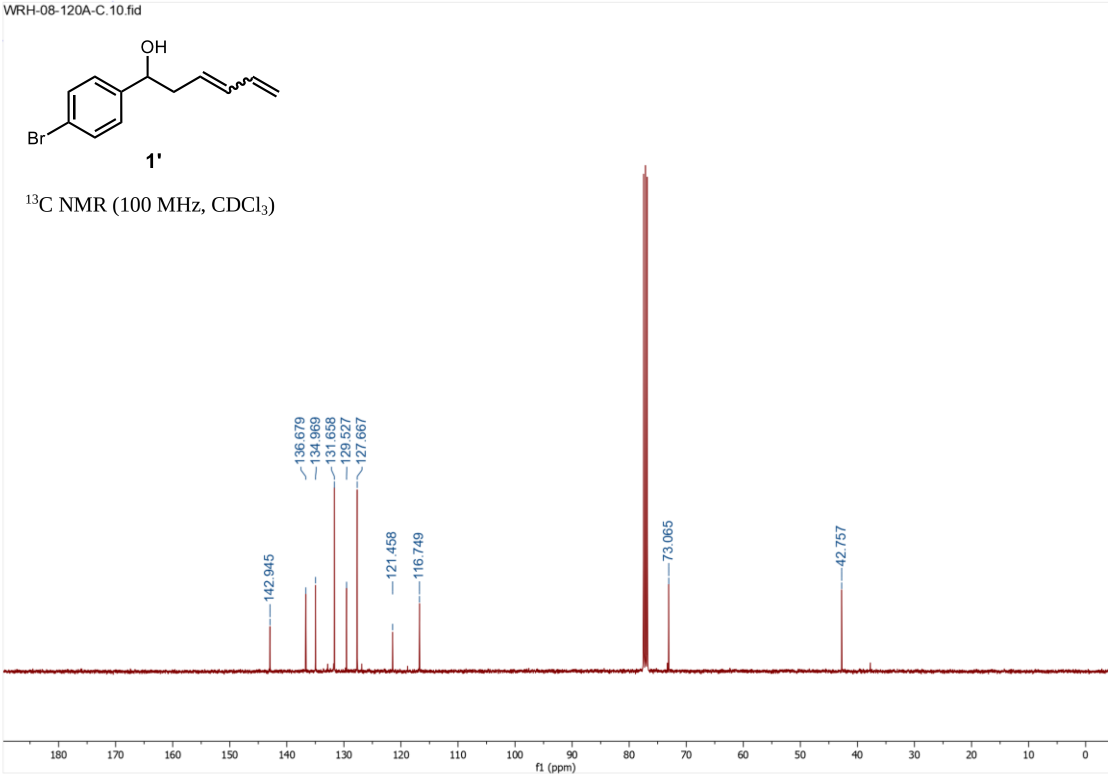
WRH-08-120A-C.10.fid
OH
Br
1'
13C NMR (100 MHz, CDCl3)
f1 (ppm)

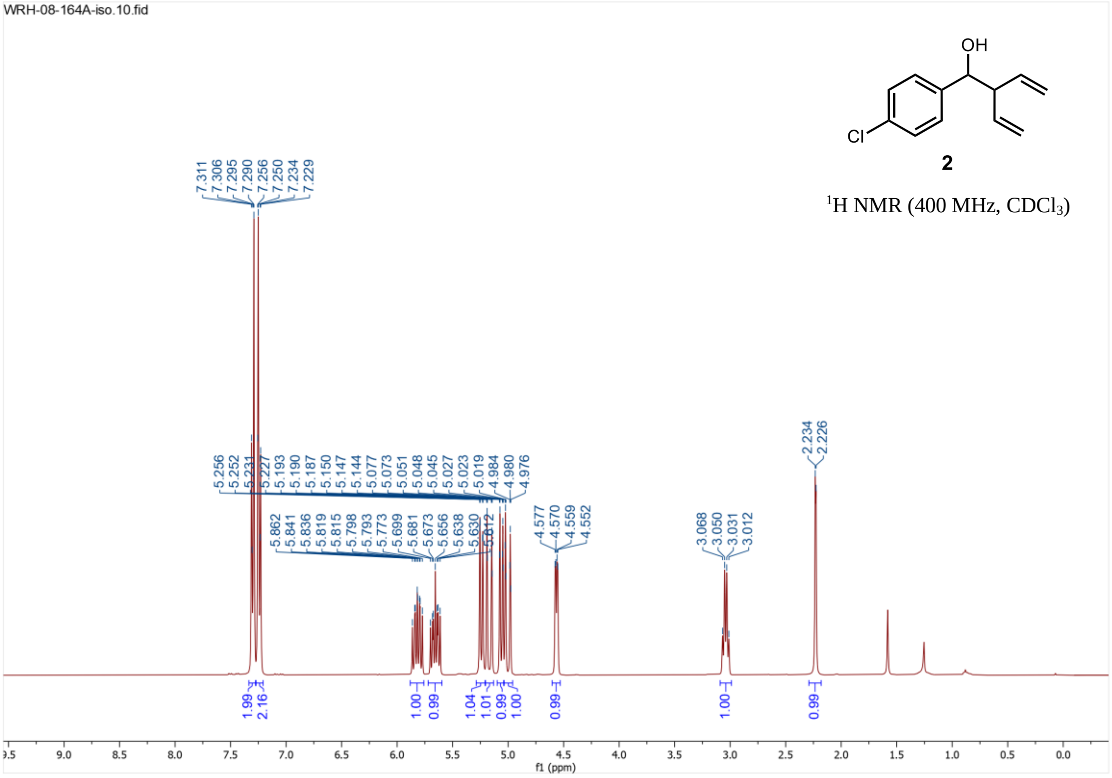
WRH-08-164A-iso.10.fid
OH
Cl
2
1H NMR (400 MHz, CDCl3)
7.311 7.306 7.295 7.290 7.256 7.250 7.234 7.229
5.256 5.252 5.231 5.227 5.193 5.190 5.187 5.150 5.147 5.144 5.077 5.073 5.051 5.048 5.045 5.027 5.023 5.019 4.984 4.980 4.976
5.862 5.841 5.836 5.819 5.815 5.798 5.793 5.773 5.699 5.681 5.673 5.656 5.638 5.630 5.612
4.577 4.570 4.559 4.552
3.068 3.050 3.031 3.012
2.234 2.226
1.99 2.16 1.00 0.99 1.04 1.01 0.99 1.00 0.99 1.00 0.99
f1 (ppm)

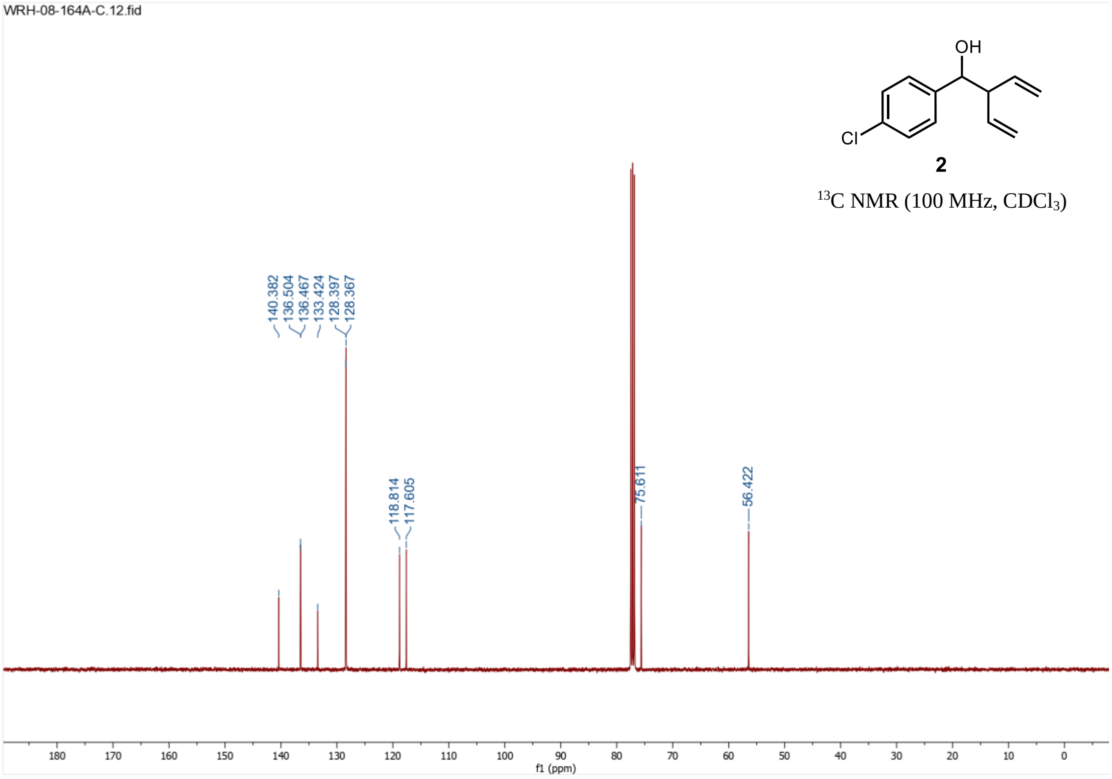
WRH-08-164A-C.12.fid
OH
Cl
2
13C NMR (100 MHz, CDCl3)
140.382 136.504 136.467 133.424 128.397 128.367
118.814 117.605
75.611
56.422
f1 (ppm)

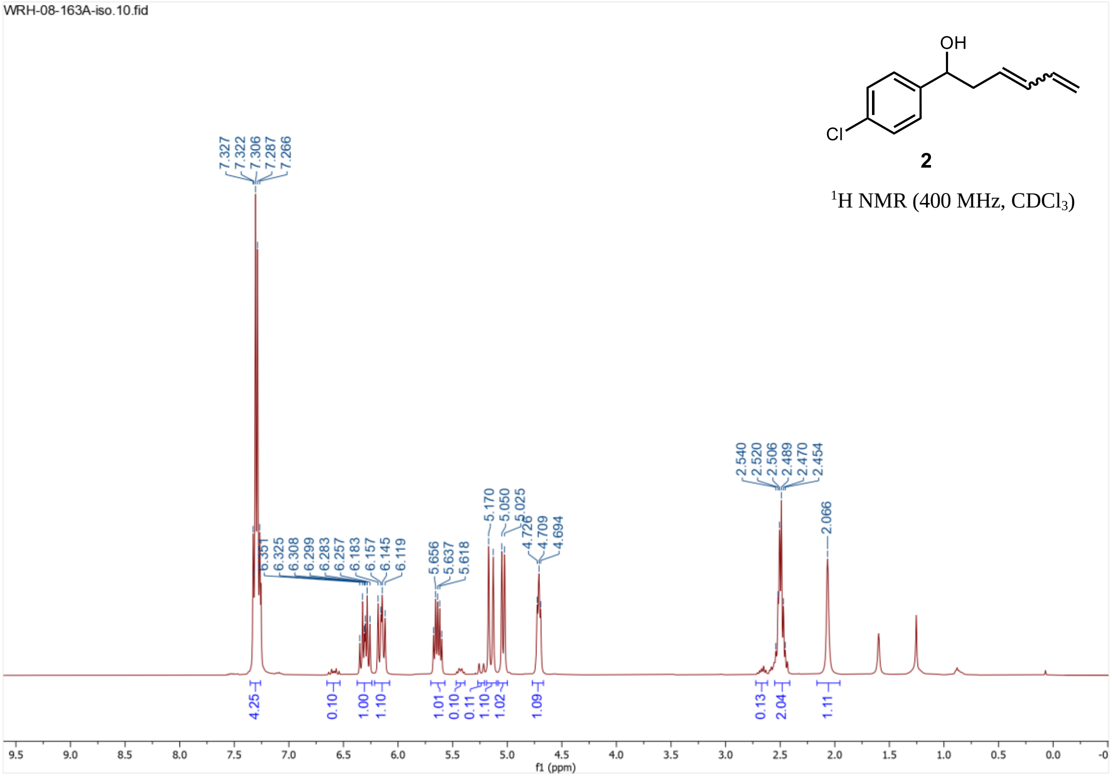
WRH-08-163A-iso.10.fid
OH
Cl
2
1H NMR (400 MHz, CDCl3)
7.327 7.322 7.306 7.287 7.266
6.351 6.325 6.308 6.299 6.283 6.257 6.183 6.157 6.145 6.119
5.656 5.637 5.618
5.170 5.050 5.025
4.726 4.709 4.694
2.540 2.520 2.506 2.489 2.470 2.454
2.066
4.25 0.10 1.00 1.10 1.01 0.10 0.11 1.10 1.02 1.09 0.13 2.04 1.11
f1 (ppm)

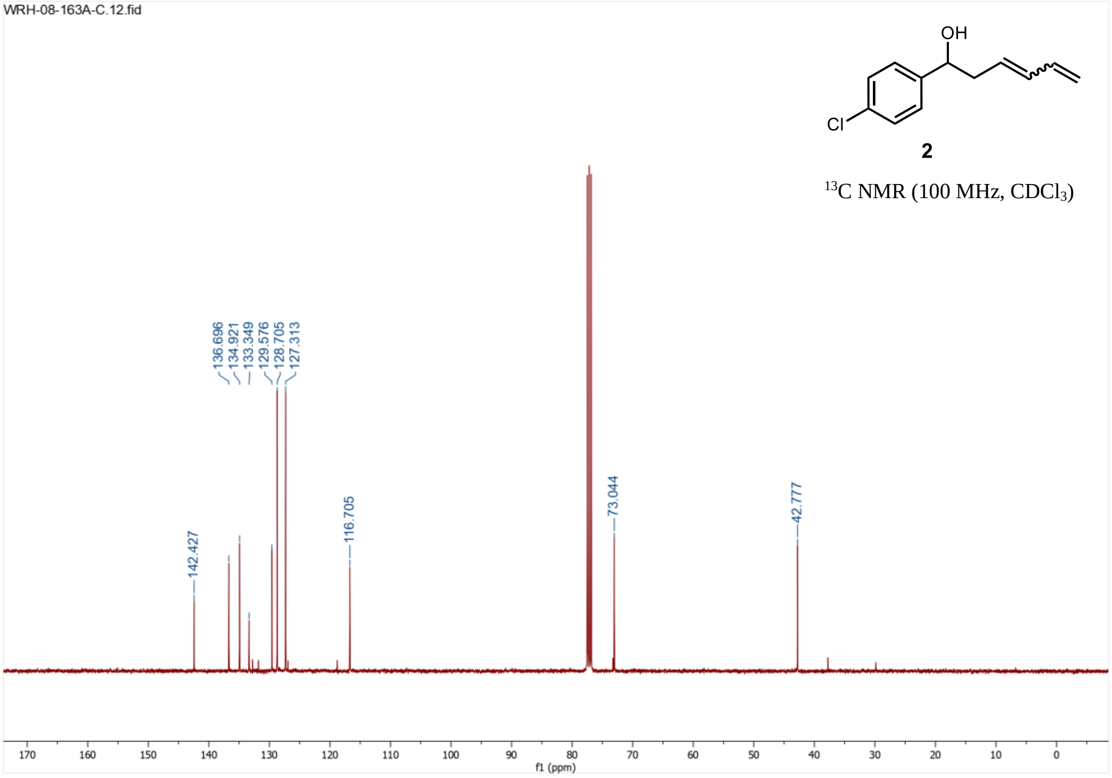
WRH-08-163A-C.12.fid
OH
Cl
2
13C NMR (100 MHz, CDCl3)
136.696 134.921 133.349 129.576 128.705 127.313
142.427
116.705
73.044
42.777
f1 (ppm)

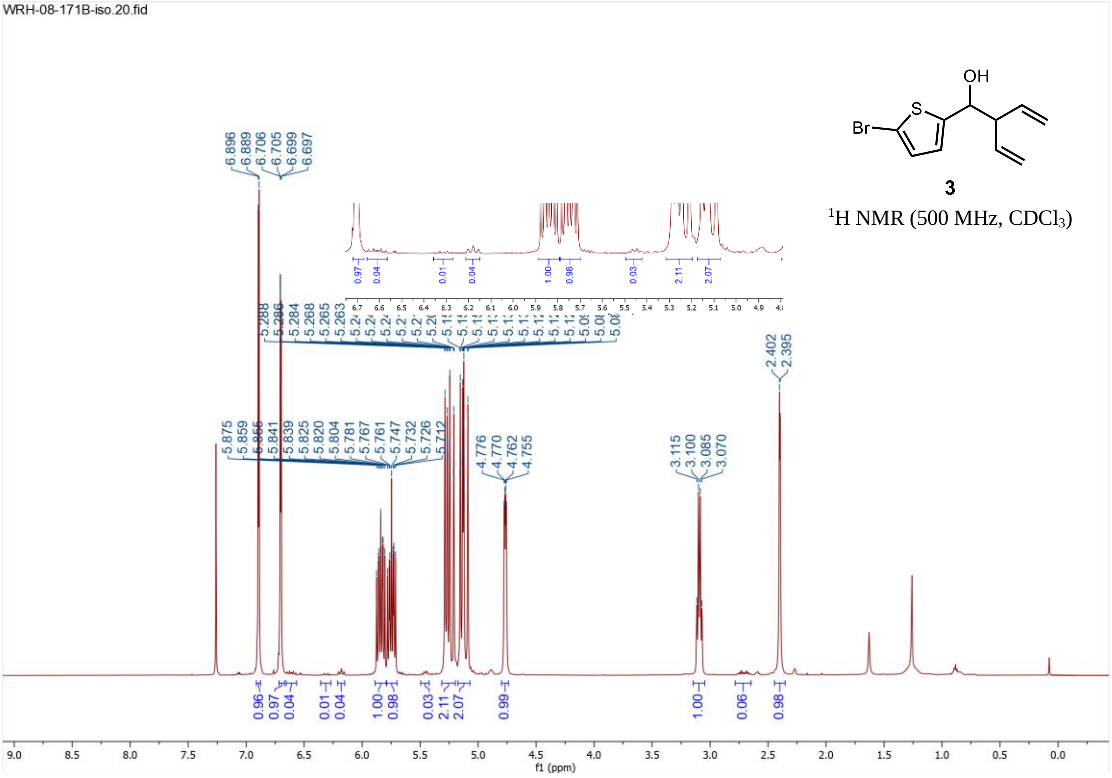
WRH-08-171B-iso.20.fid
OH
S
Br
3
^{1}H NMR (500 MHz, CDCl$_3$)
f1 (ppm)

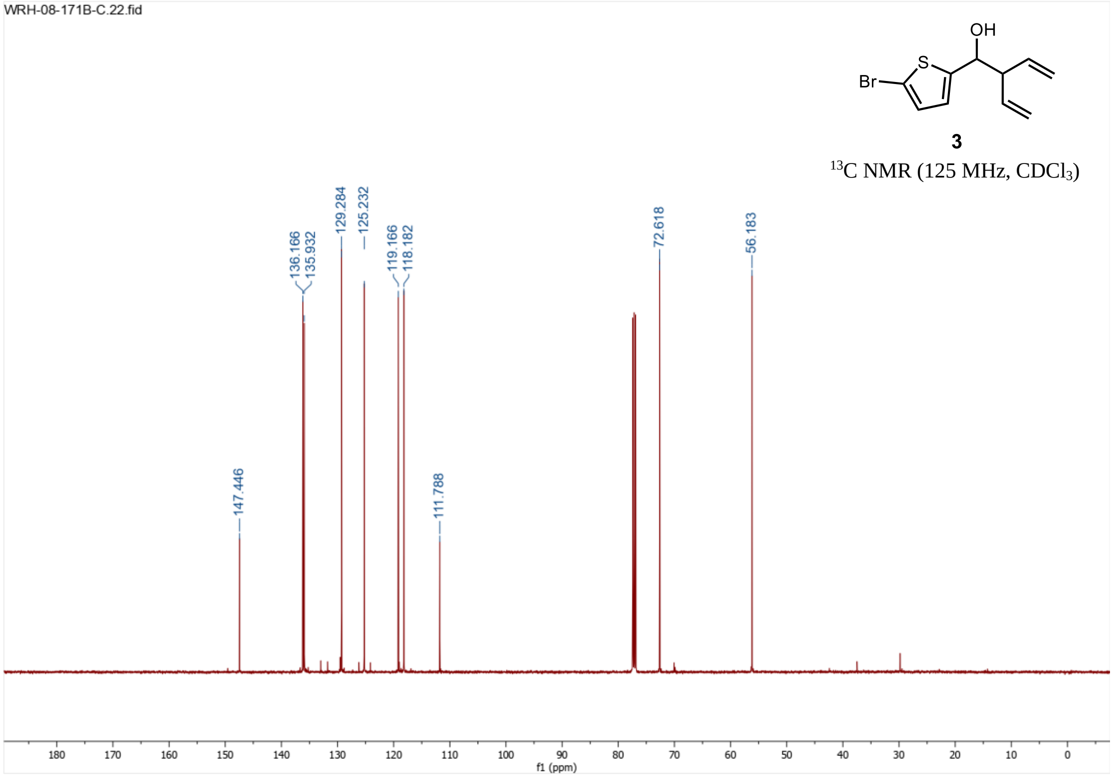
WRH-08-171B-C.22.fid
OH
S
Br
3
^{13}C NMR (125 MHz, CDCl$_3$)
147.446
136.166
135.932
129.284
125.232
119.166
118.182
111.788
72.618
56.183
f1 (ppm)

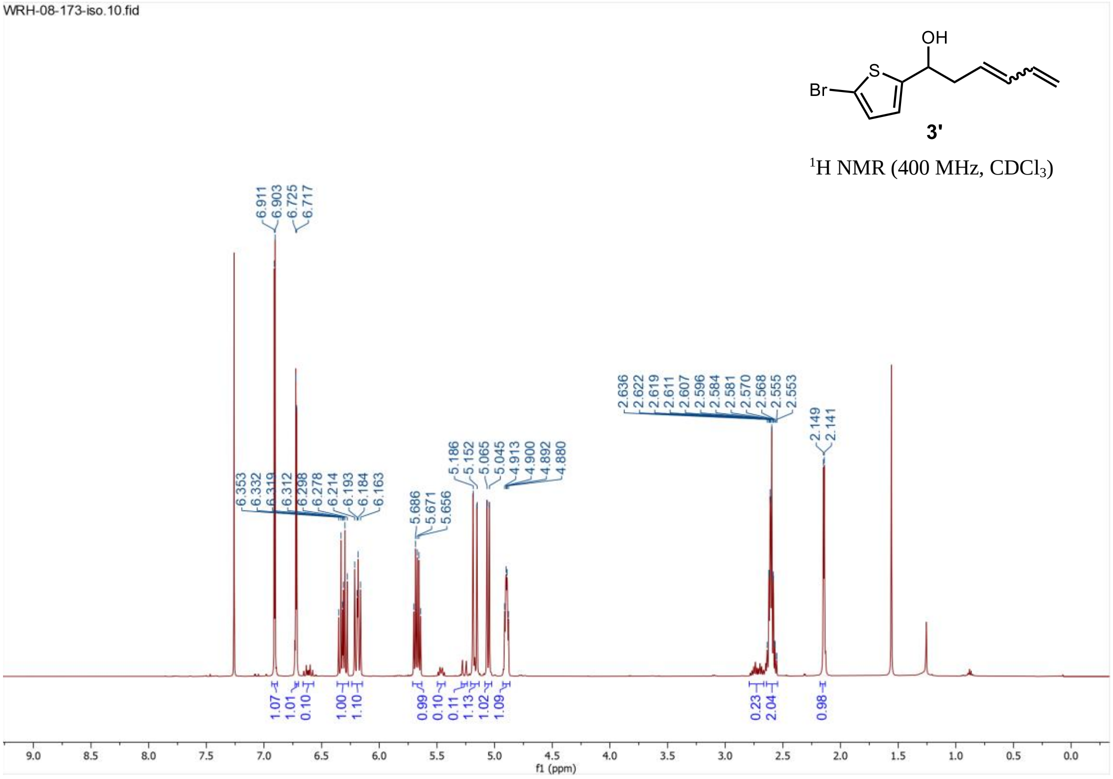
WRH-08-173-iso.10.fid
OH
S
Br
3'
1H NMR (400 MHz, CDCl3)
6.911 6.903 6.725 6.717
6.353 6.332 6.319 6.312 6.298 6.278 6.214 6.193 6.184 6.163
5.686 5.671 5.656
5.186 5.152 5.065 5.045 4.913 4.900 4.892 4.880
2.636 2.622 2.619 2.611 2.607 2.596 2.584 2.581 2.570 2.568 2.555 2.553
2.149 2.141
1.07 1.01 0.10 1.00 1.10 0.99 0.10 0.11 1.13 1.02 1.09 0.23 2.04 0.98
f1 (ppm)

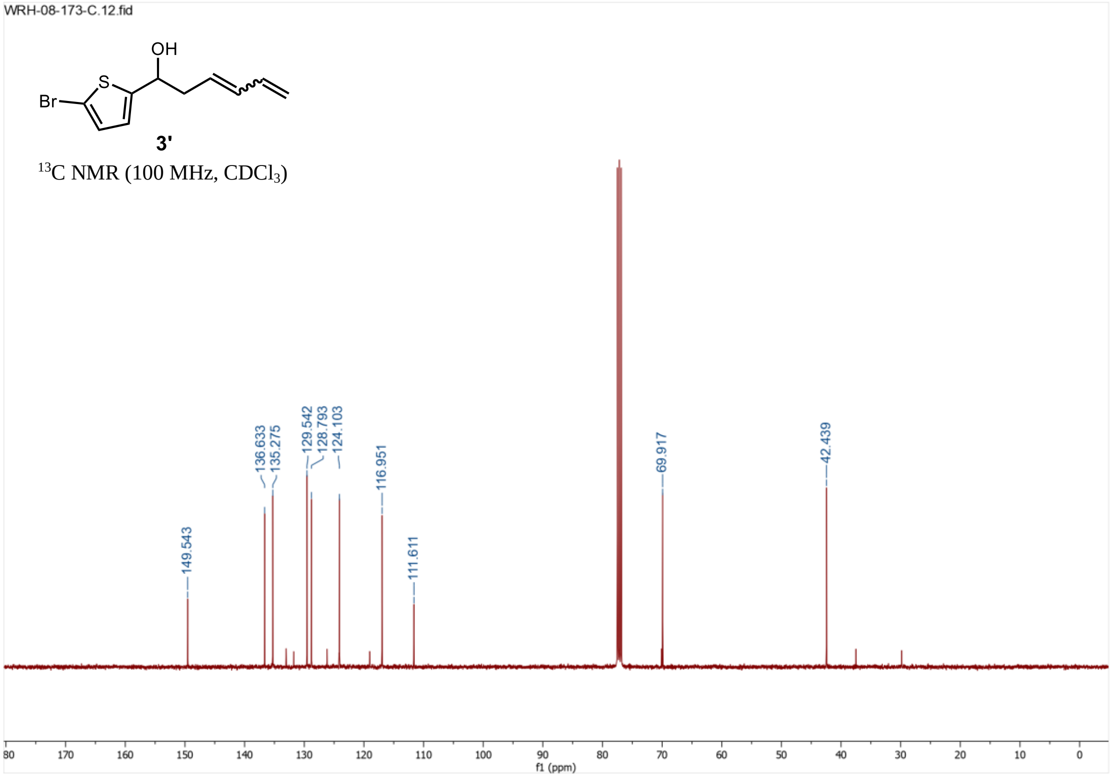
WRH-08-173-C.12.fid
OH
S
Br
3'
13C NMR (100 MHz, CDCl3)
149.543
136.633 135.275
129.542 128.793 124.103
116.951
111.611
69.917
42.439
f1 (ppm)

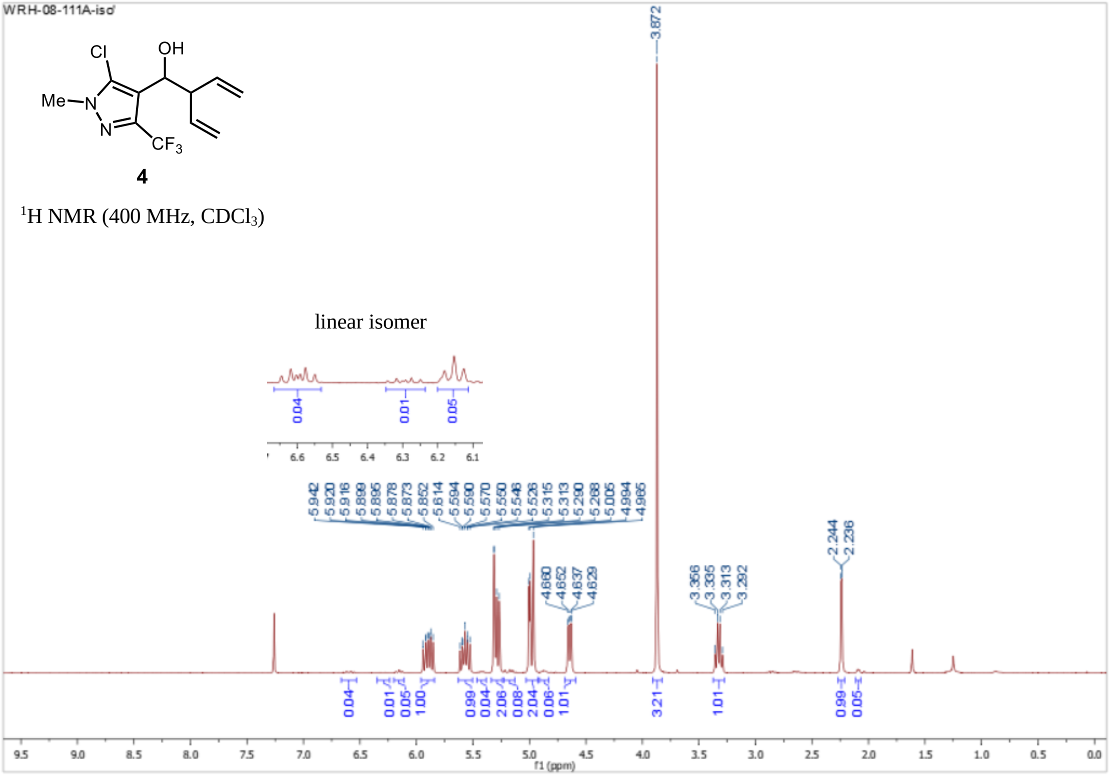
WRH-08-111A-iso
Cl
OH
Me
N
N
CF3
4
1H NMR (400 MHz, CDCl3)
linear isomer
f1 (ppm)

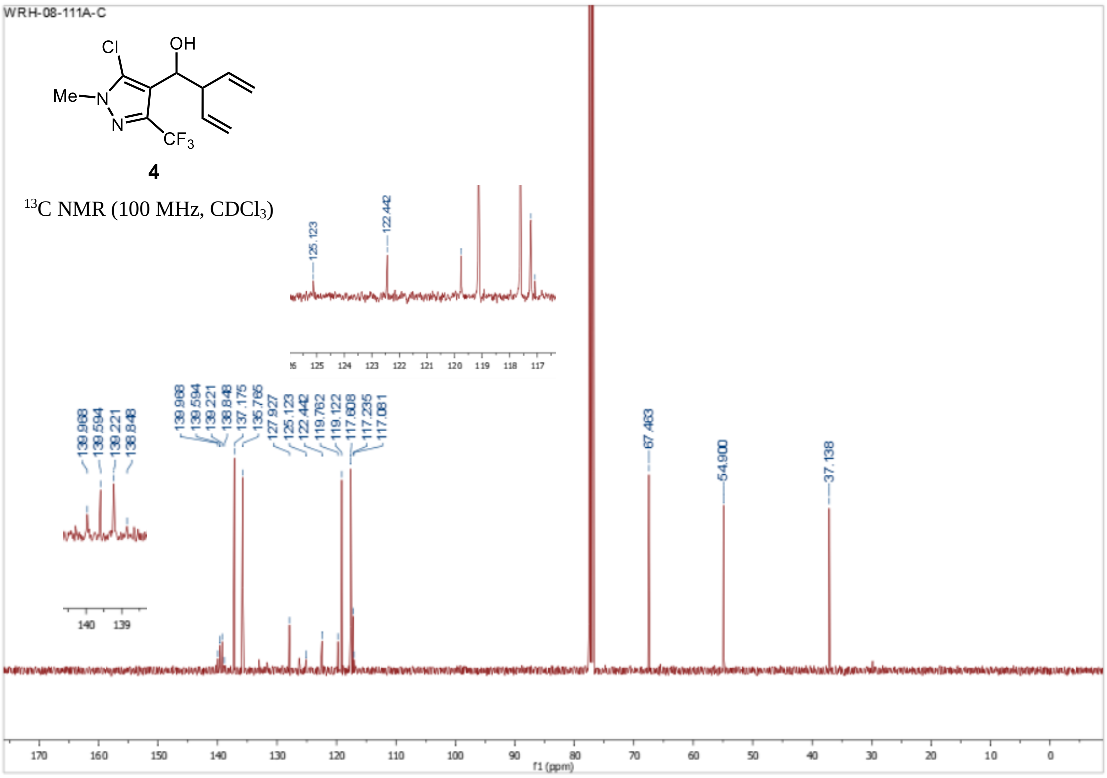
WRH-08-111A-C
Cl
OH
Me
N
N
CF3
4
13C NMR (100 MHz, CDCl3)
f1 (ppm)

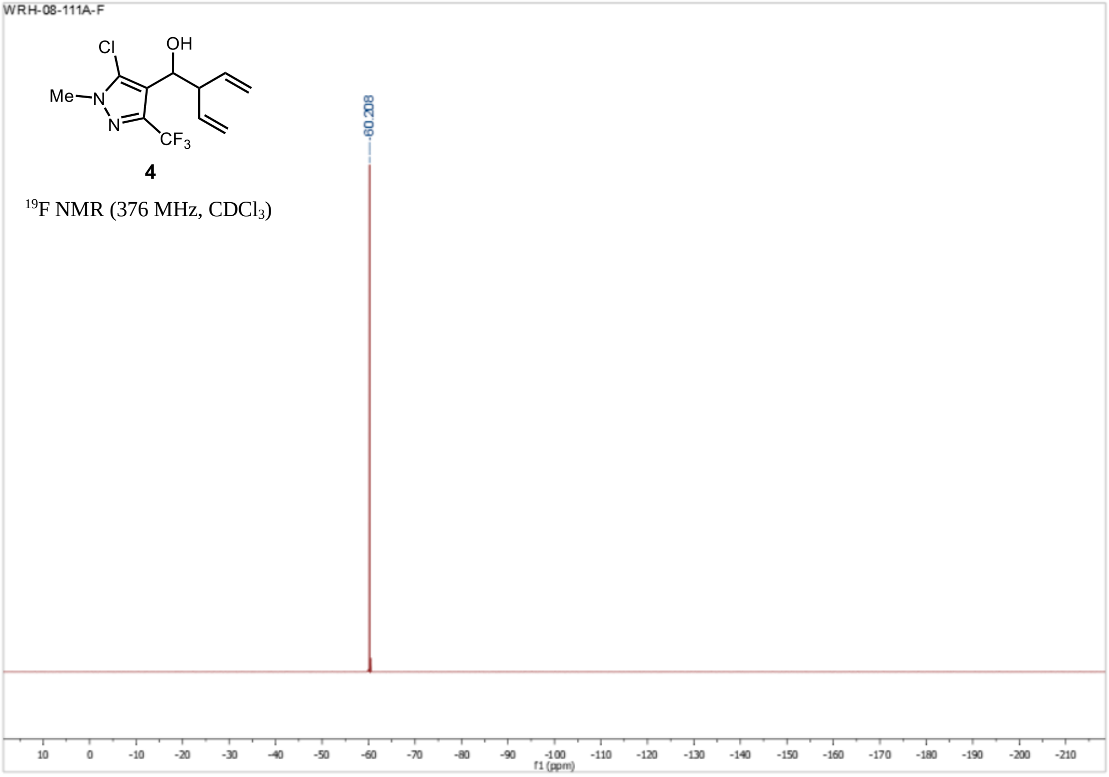

4

^{19}F NMR (376 MHz, $CDCl_3$)

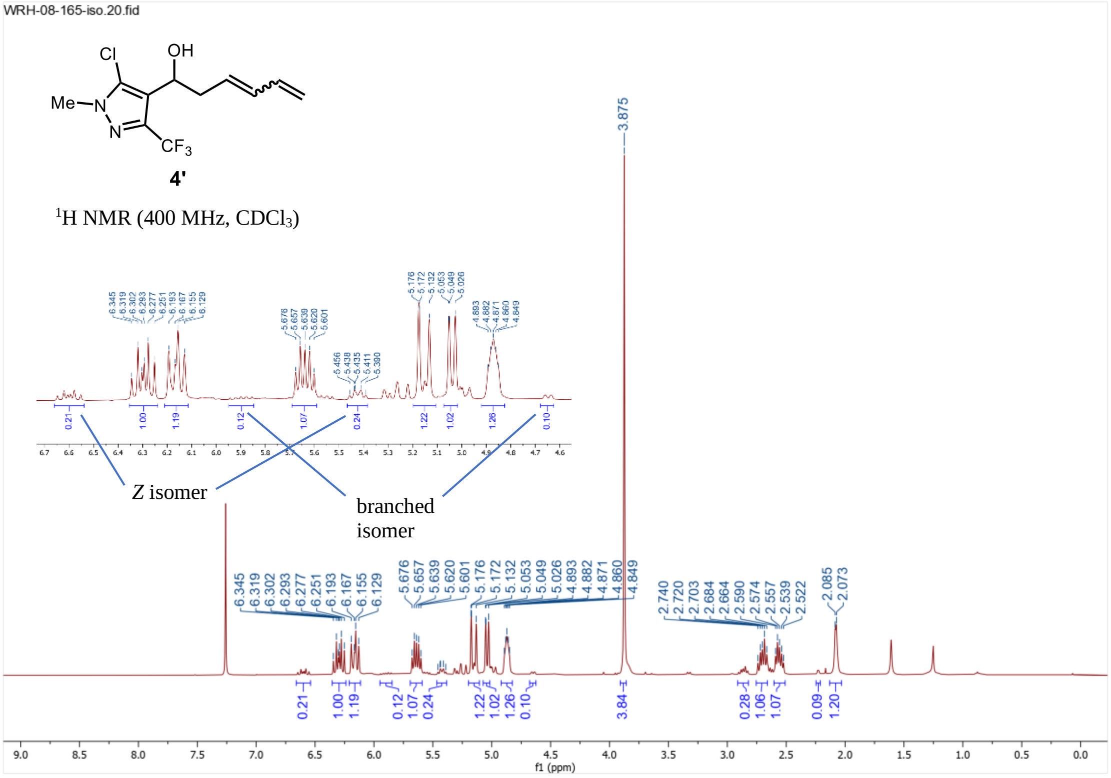

WRH-08-165-iso.20.fid
Cl
OH
Me
N
N
CF3
4'
¹H NMR (400 MHz, CDCl3)
Z isomer
branched isomer
3.875
f1 (ppm)

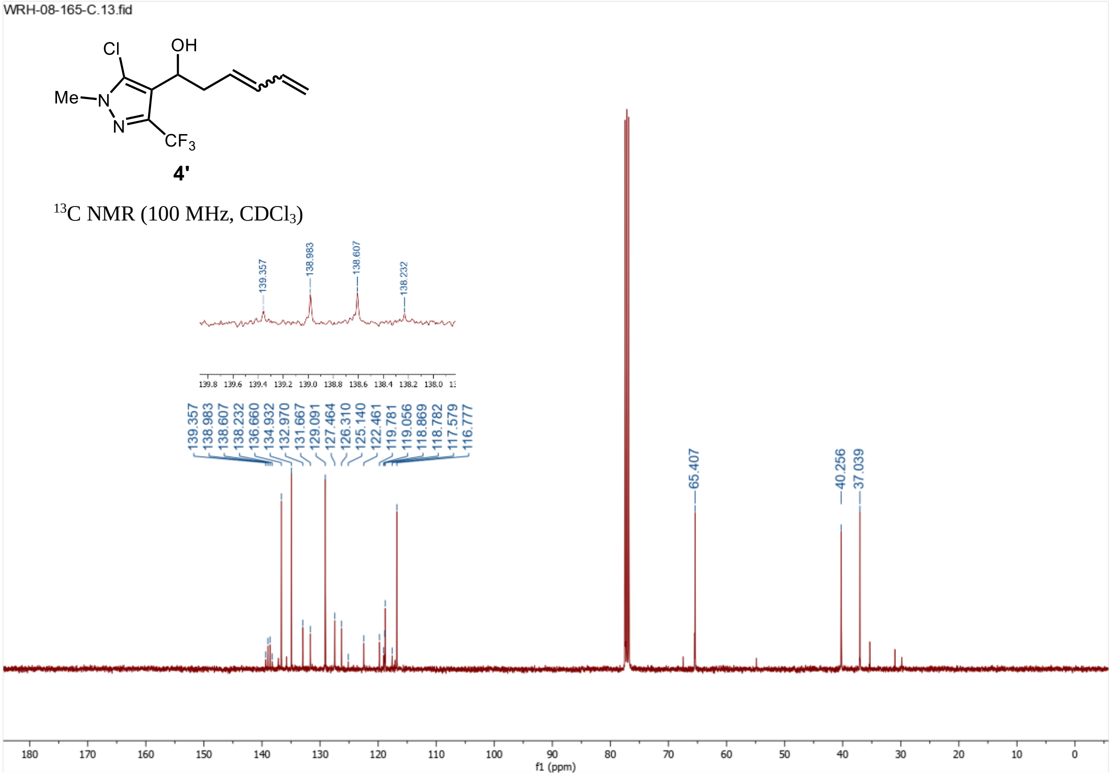

WRH-08-165-C.13.fid
Cl
OH
Me
N
N
CF3
4'
¹³C NMR (100 MHz, CDCl3)
139.357
138.983
138.607
138.232
136.660
134.932
132.970
131.667
129.091
127.464
126.310
125.140
122.461
119.781
119.056
118.869
118.782
117.579
116.777
65.407
40.256
37.039
f1 (ppm)

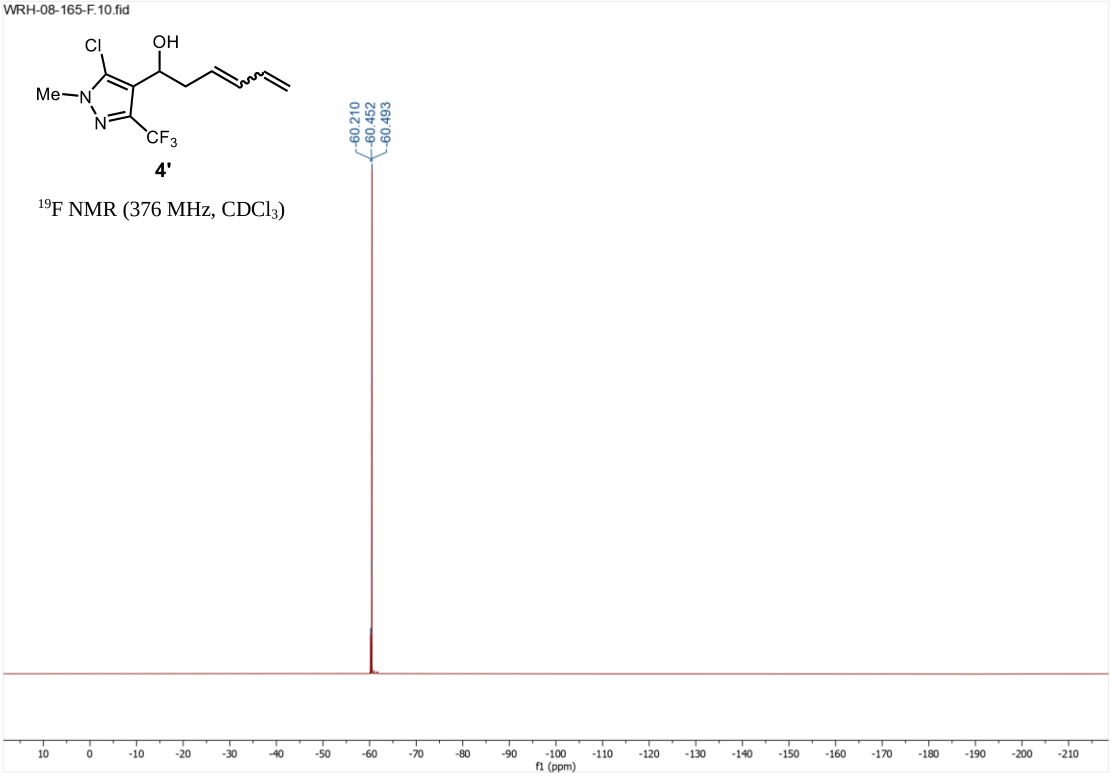
WRH-08-165-F.10.fid
Cl
OH
Me
N
N
CF3
4'
19F NMR (376 MHz, CDCl3)
-60.210
-60.452
-60.493
10 0 -10 -20 -30 -40 -50 -60 -70 -80 -90 -100 -110 -120 -130 -140 -150 -160 -170 -180 -190 -200 -210
f1 (ppm)

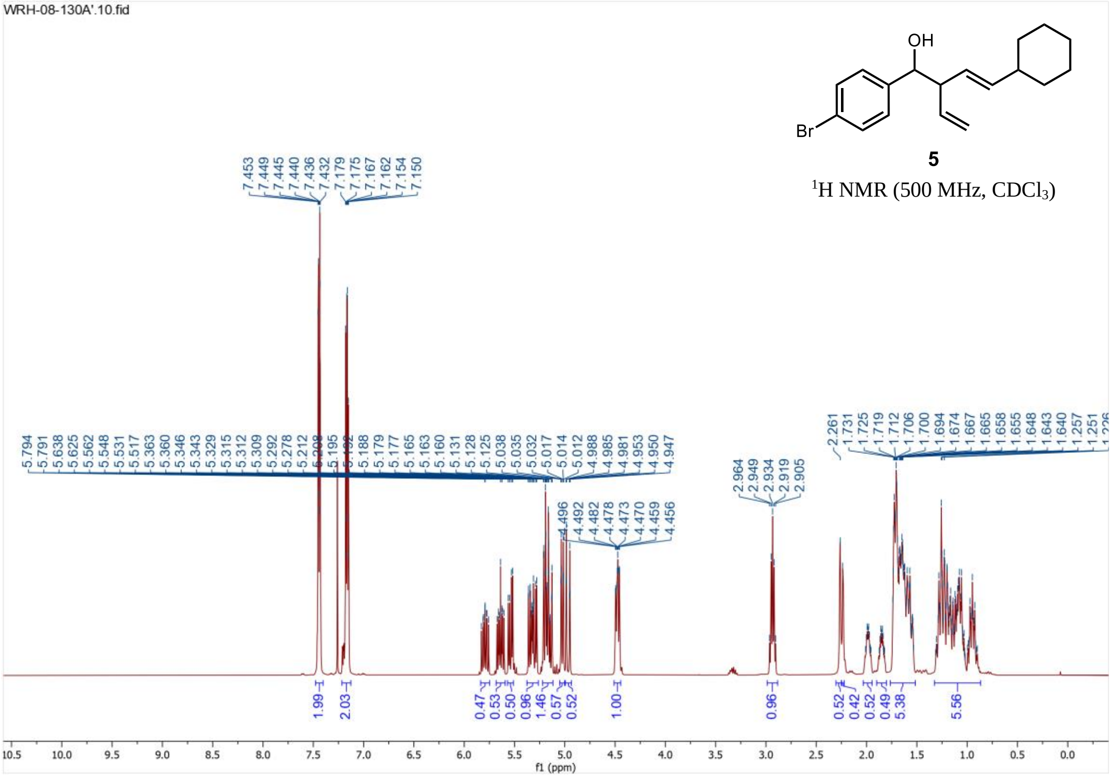
WRH-08-130A'.10.fid
OH
Br
5
1H NMR (500 MHz, CDCl3)
7.453 7.449 7.445 7.440 7.436 7.432 7.179 7.175 7.167 7.162 7.154 7.150
5.794 5.791 5.638 5.625 5.562 5.548 5.531 5.517 5.363 5.360 5.346 5.343 5.329 5.315 5.312 5.309 5.292 5.278 5.212 5.195 5.188 5.179 5.177 5.165 5.163 5.160 5.131 5.128 5.125 5.038 5.035 5.032 5.017 5.014 5.012 4.988 4.985 4.981 4.953 4.950 4.947
4.496 4.492 4.482 4.478 4.473 4.470 4.459 4.456
2.964 2.949 2.934 2.919 2.905
2.261 1.731 1.725 1.719 1.712 1.706 1.700 1.694 1.674 1.667 1.665 1.658 1.655 1.648 1.643 1.640 1.257 1.251
1.99 2.03 0.47 0.53 0.50 0.96 1.46 0.57 0.52 1.00 0.96 0.52 0.42 0.52 0.49 5.38 5.56
f1 (ppm)

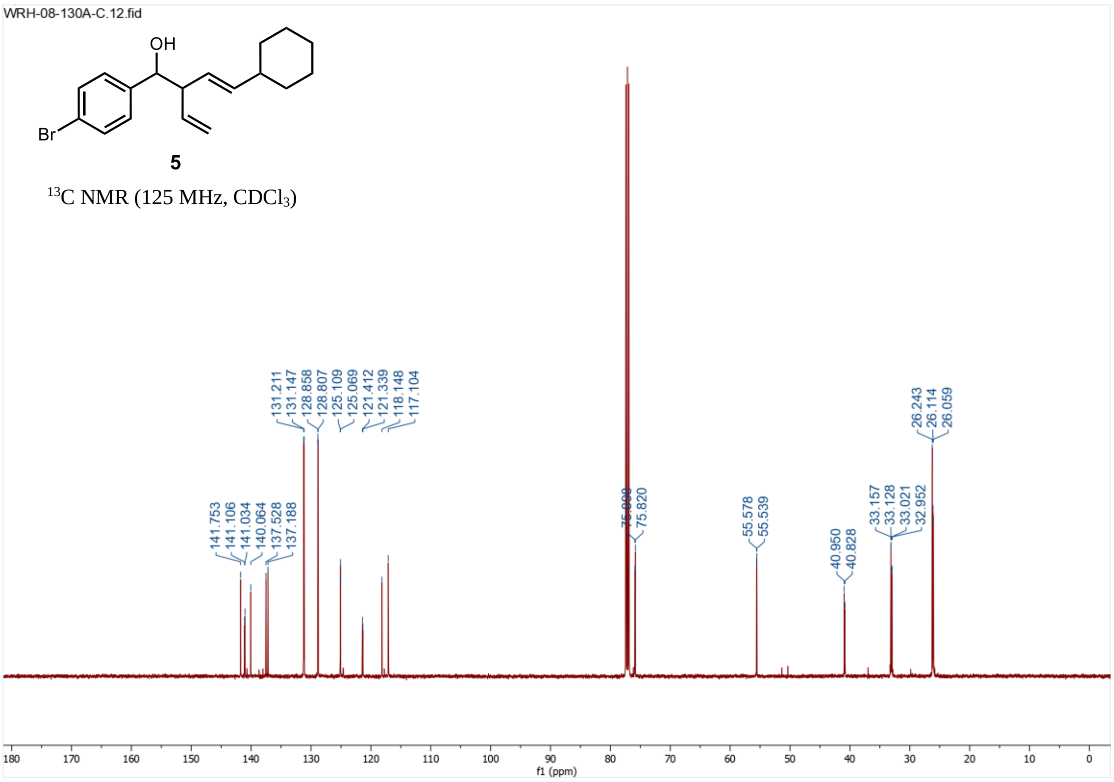
WRH-08-130A-C.12.fid
OH
Br
5
13C NMR (125 MHz, CDCl3)
141.753 141.106 141.034 140.064 137.528 137.188
131.211 131.147 128.858 128.807 125.109 125.069 121.412 121.339 118.148 117.104
75.820
55.578 55.539
40.950 40.828
33.157 33.128 33.021 32.952
26.243 26.114 26.059
f1 (ppm)

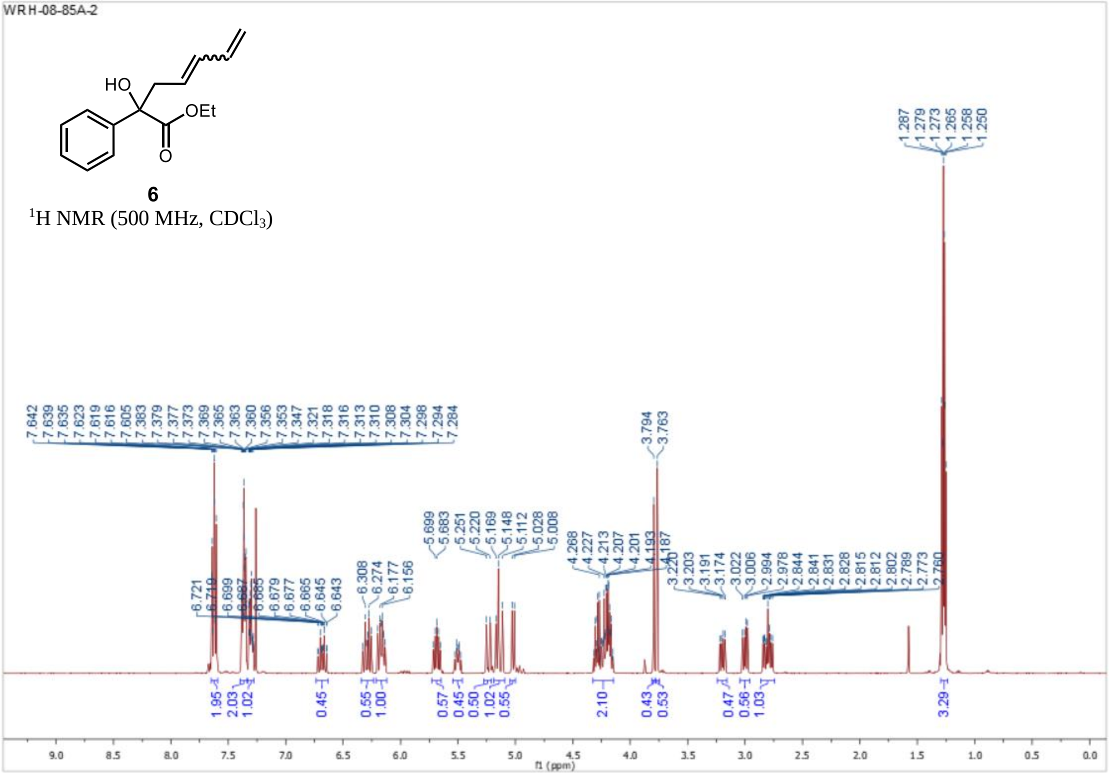
WRH-08-85A-2
HO
OEt
O
6
1H NMR (500 MHz, CDCl3)
f1 (ppm)

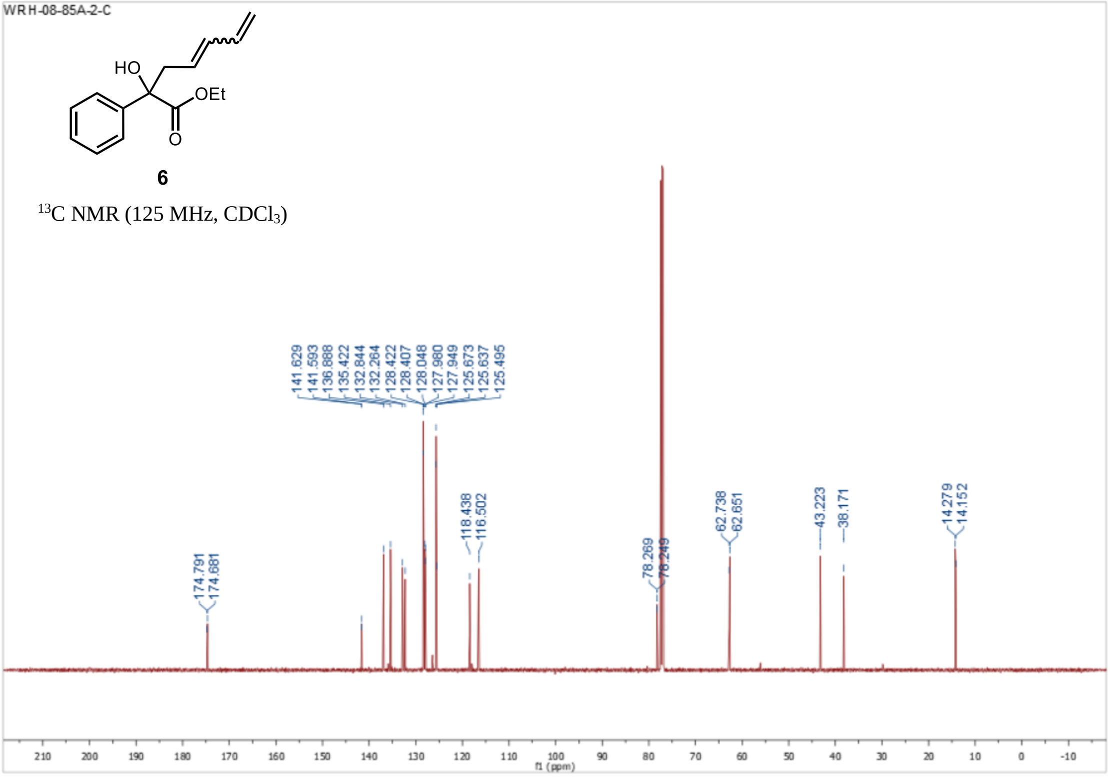
WRH-08-85A-2-C
HO
OEt
O
6
13C NMR (125 MHz, CDCl3)
f1 (ppm)

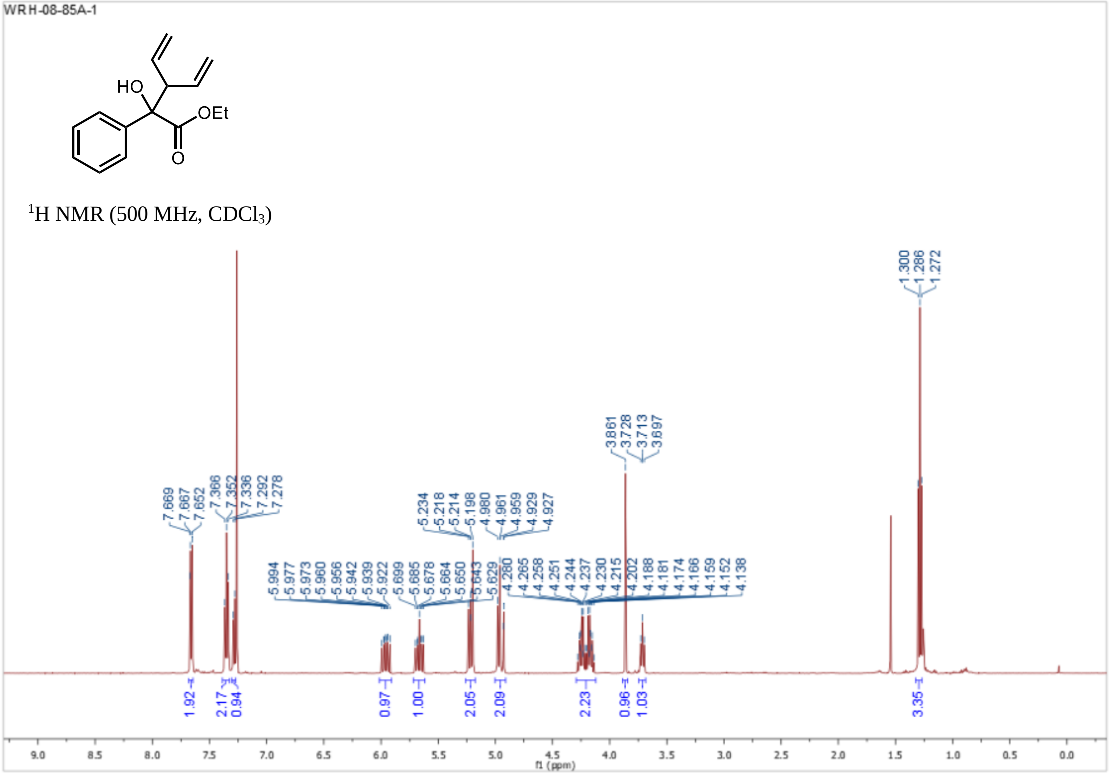
WRH-08-85A-1
HO
OEt
O
^{1}H NMR (500 MHz, CDCl3)
f1 (ppm)

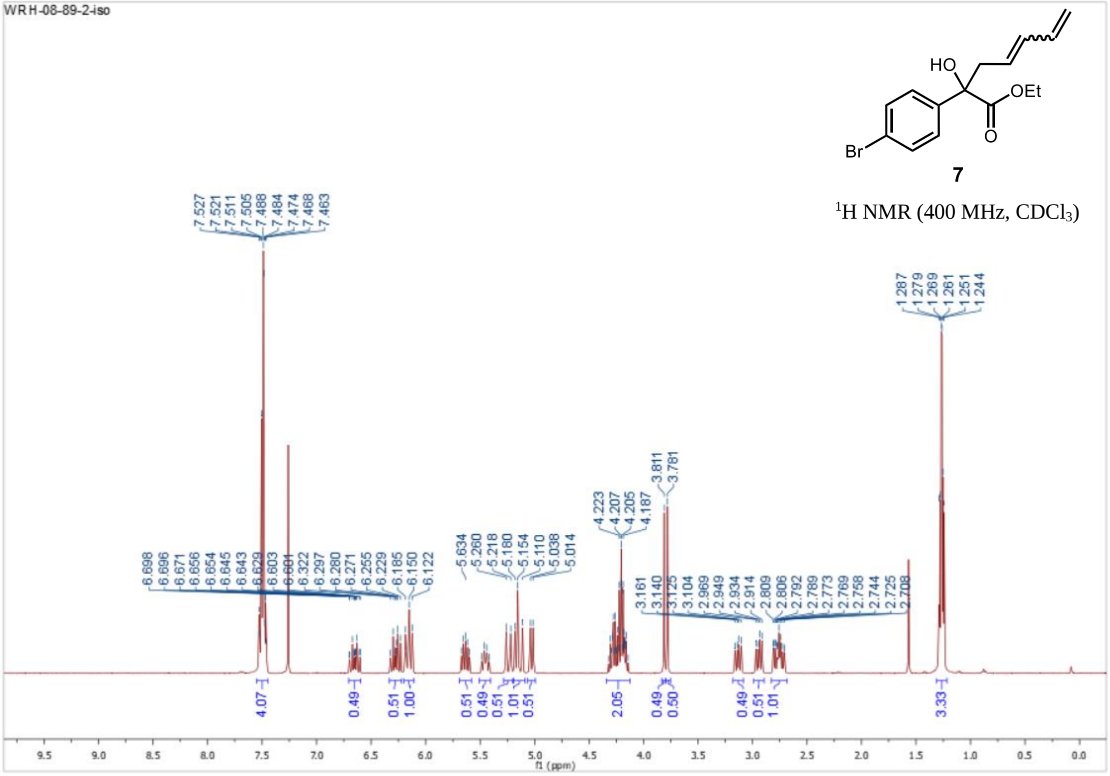
WRH-08-89-2-iso
HO
OEt
O
Br
7
^{1}H NMR (400 MHz, CDCl$_3$)
7.527 7.521 7.511 7.505 7.488 7.484 7.474 7.468 7.463
6.698 6.696 6.671 6.656 6.654 6.645 6.643 6.629 6.603 6.601 6.322 6.297 6.280 6.271 6.255 6.229 6.185 6.150 6.122
5.634 5.260 5.218 5.180 5.154 5.110 5.038 5.014
4.223 4.207 4.205 4.187
3.811 3.781
3.161 3.140 3.125 3.104 2.969 2.949 2.934 2.914 2.809 2.806 2.792 2.789 2.773 2.769 2.758 2.744 2.725 2.708
1.287 1.279 1.269 1.261 1.251 1.244
4.07 0.49 0.51 1.00 0.51 0.49 0.51 1.01 1.01 0.51 2.05 0.49 0.50 0.49 0.51 1.01 3.33
f1 (ppm)

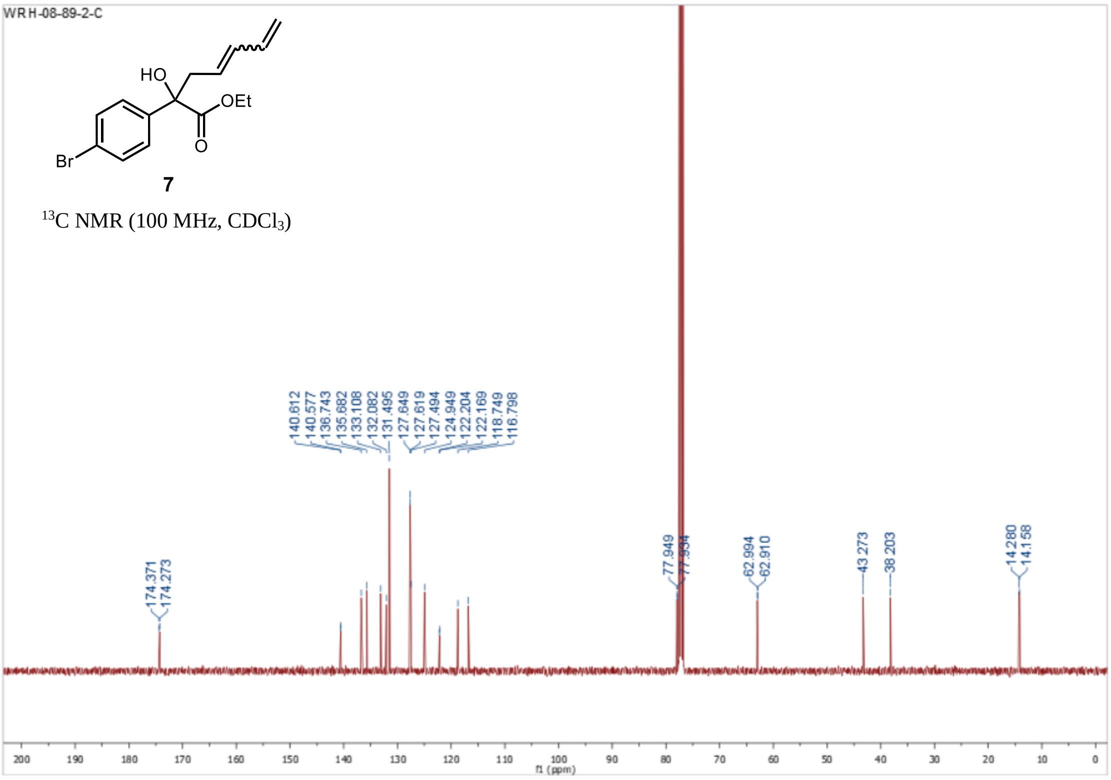
WRH-08-89-2-C
HO
OEt
O
Br
7
^{13}C NMR (100 MHz, CDCl$_3$)
174.371 174.273
140.612 140.577 136.743 135.682 133.108 132.082 131.495 127.649 127.619 127.494 124.949 122.204 122.169 118.749 116.798
77.949 77.934
62.994 62.910
43.273 38.203
14.280 14.158
f1 (ppm)

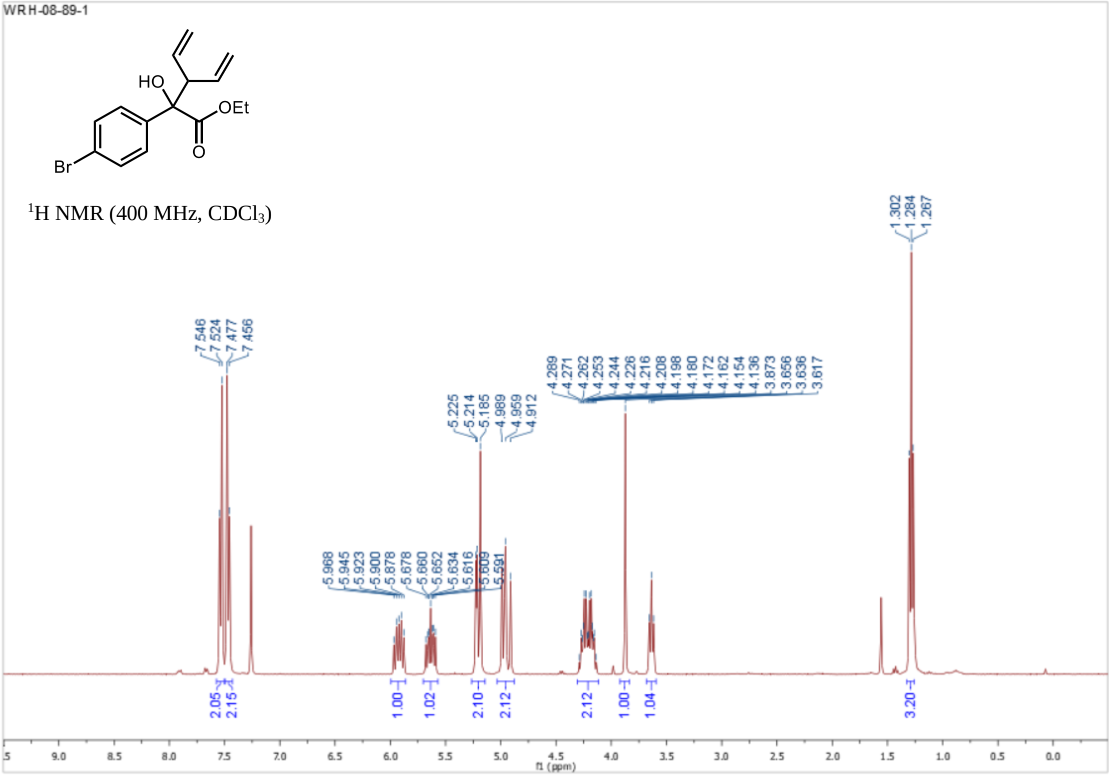
WRH-08-89-1
HO
OEt
O
Br
¹H NMR (400 MHz, CDCl₃)
f1 (ppm)

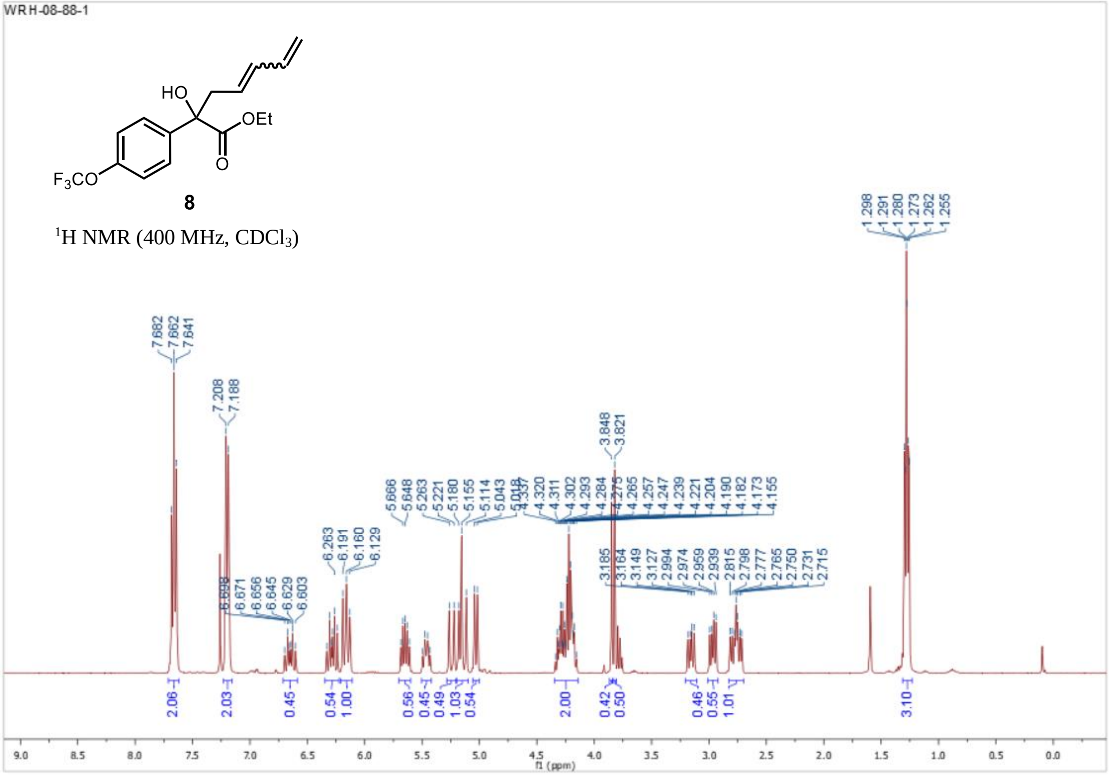
WRH-08-88-1
HO
OEt
O
F_3CO
8
^{1}H NMR (400 MHz, $CDCl_3$)
f1 (ppm)

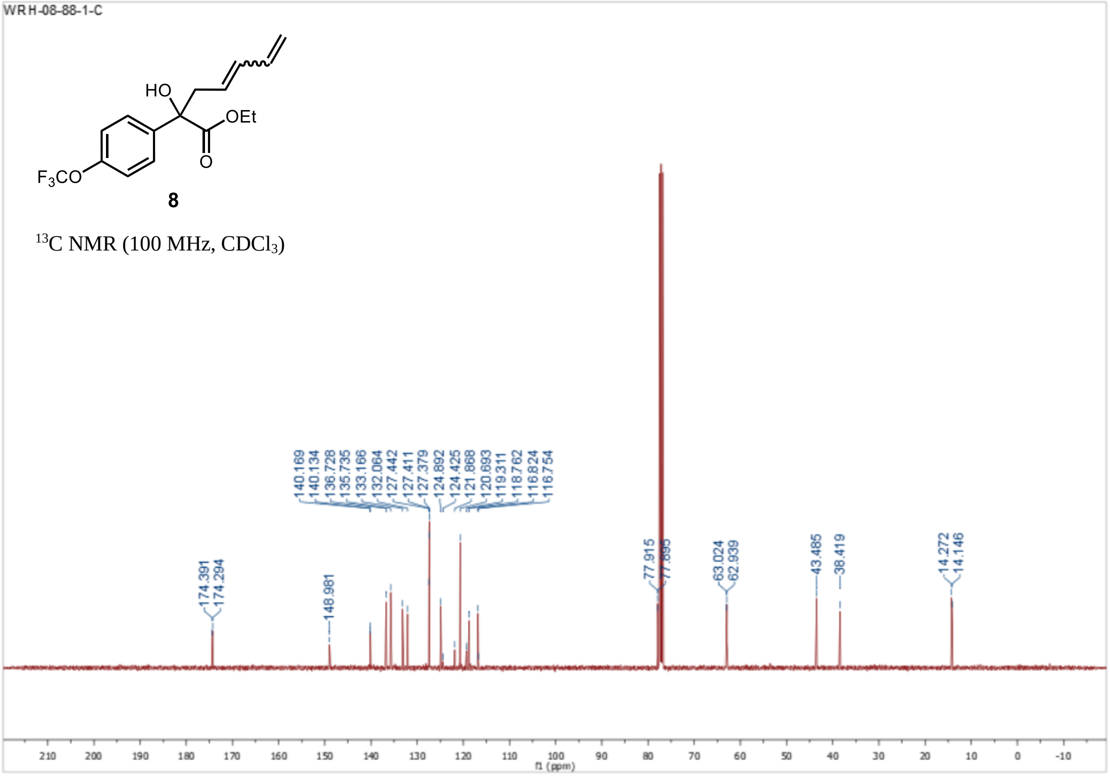
WRH-08-88-1-C
HO
OEt
O
F_3CO
8
^{13}C NMR (100 MHz, $CDCl_3$)
f1 (ppm)

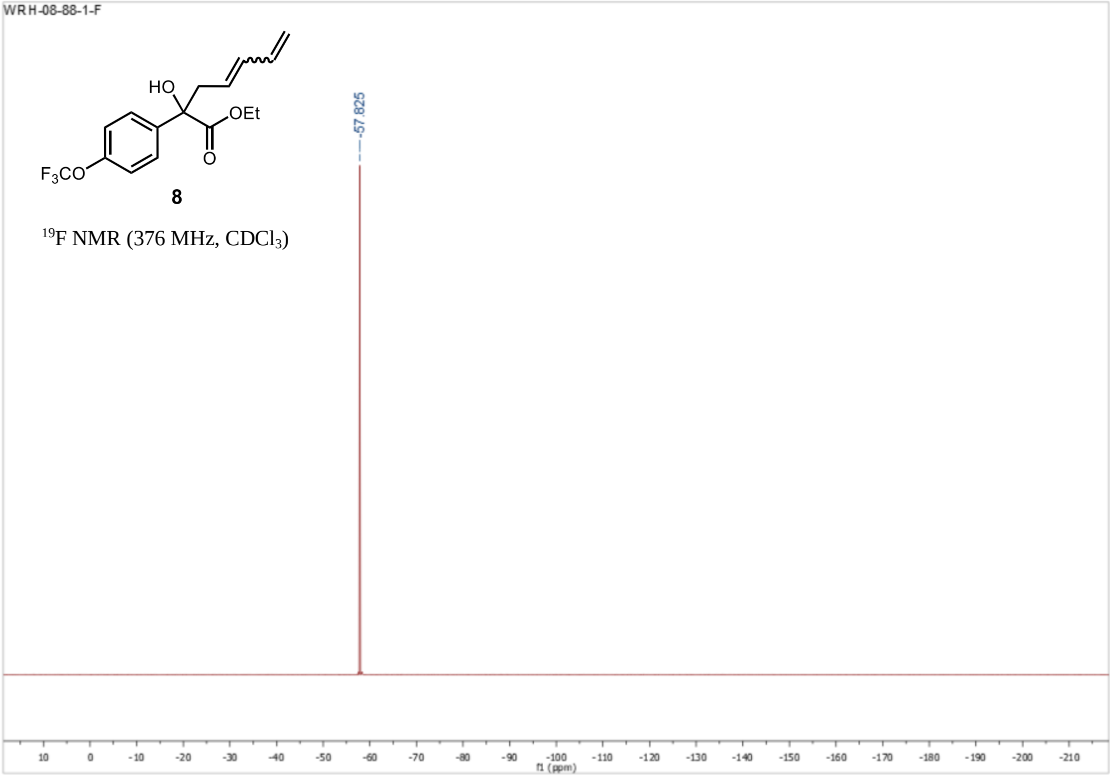
WRH-08-88-1-F
HO
OEt
O
F3CO
8
19F NMR (376 MHz, CDCl3)
-57.825
f1 (ppm)

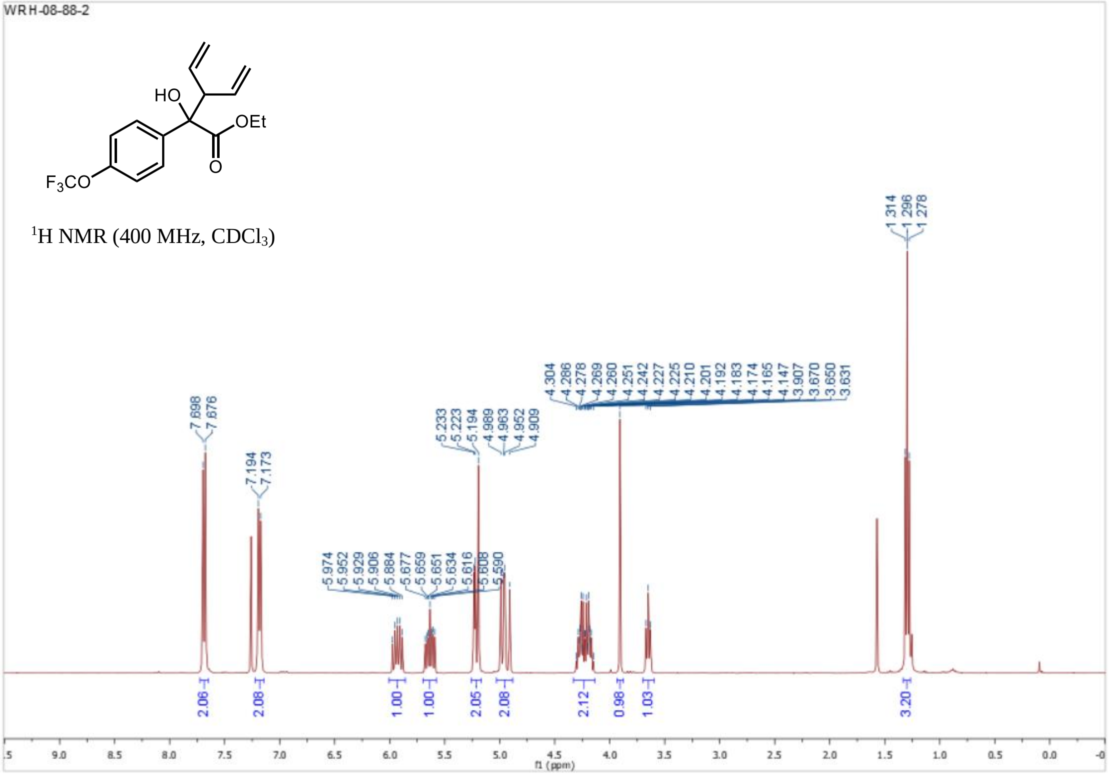
WRH-08-88-2
HO
OEt
O
F3CO
1H NMR (400 MHz, CDCl3)
7.698
7.676
7.194
7.173
5.974
5.952
5.929
5.906
5.884
5.677
5.659
5.651
5.634
5.616
5.608
5.590
5.233
5.223
5.194
4.989
4.963
4.952
4.909
4.304
4.286
4.278
4.269
4.260
4.251
4.242
4.227
4.225
4.210
4.201
4.192
4.183
4.174
4.165
4.147
3.907
3.670
3.650
3.631
1.314
1.296
1.278
2.06
2.08
1.00
1.00
2.05
2.08
2.12
0.98
1.03
3.20
f1 (ppm)

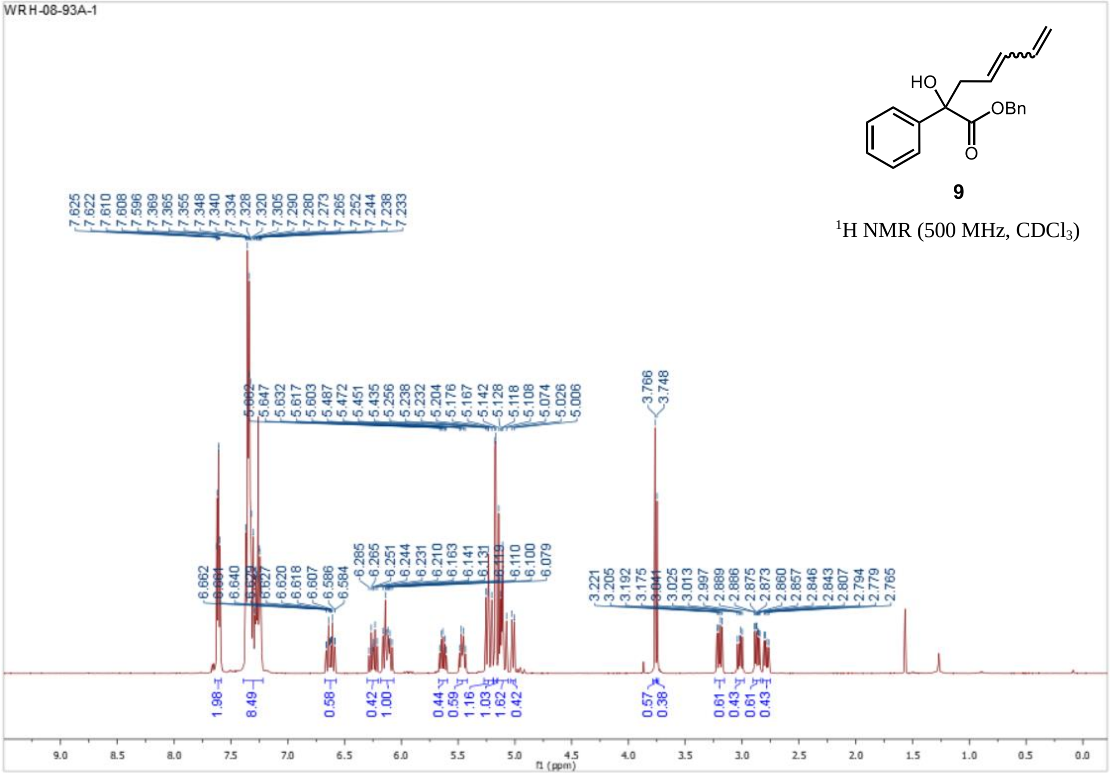
WRH-08-93A-1
HO
OBn
O
9
^{1}H NMR (500 MHz, $CDCl_3$)
f1 (ppm)

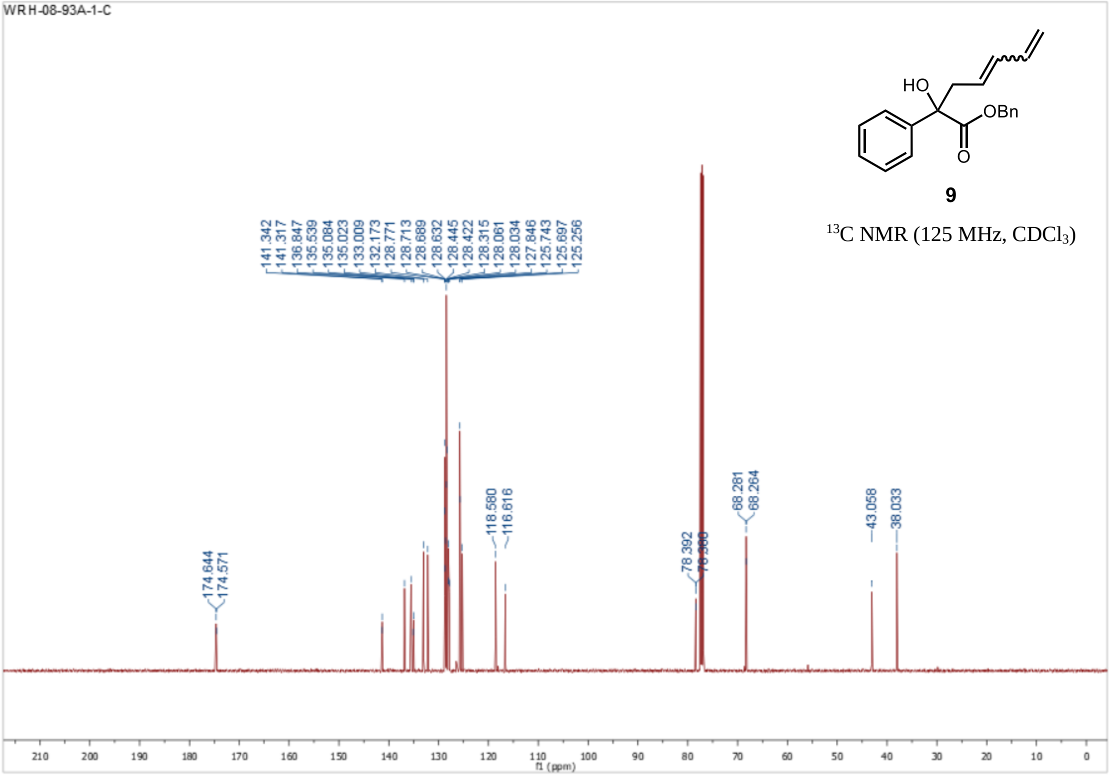
WRH-08-93A-1-C
HO
OBn
O
9
^{13}C NMR (125 MHz, $CDCl_3$)
f1 (ppm)

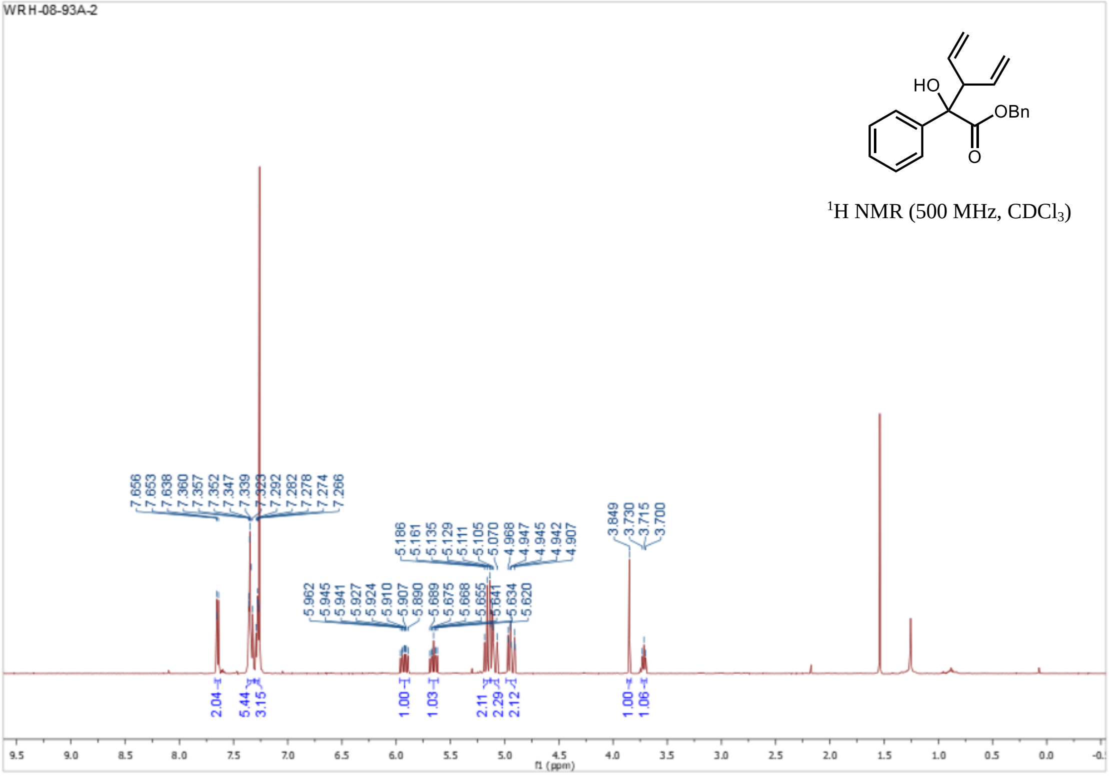
WRH-08-93A-2
HO
OBn
O
^{1}H NMR (500 MHz, CDCl$_3$)
f1 (ppm)

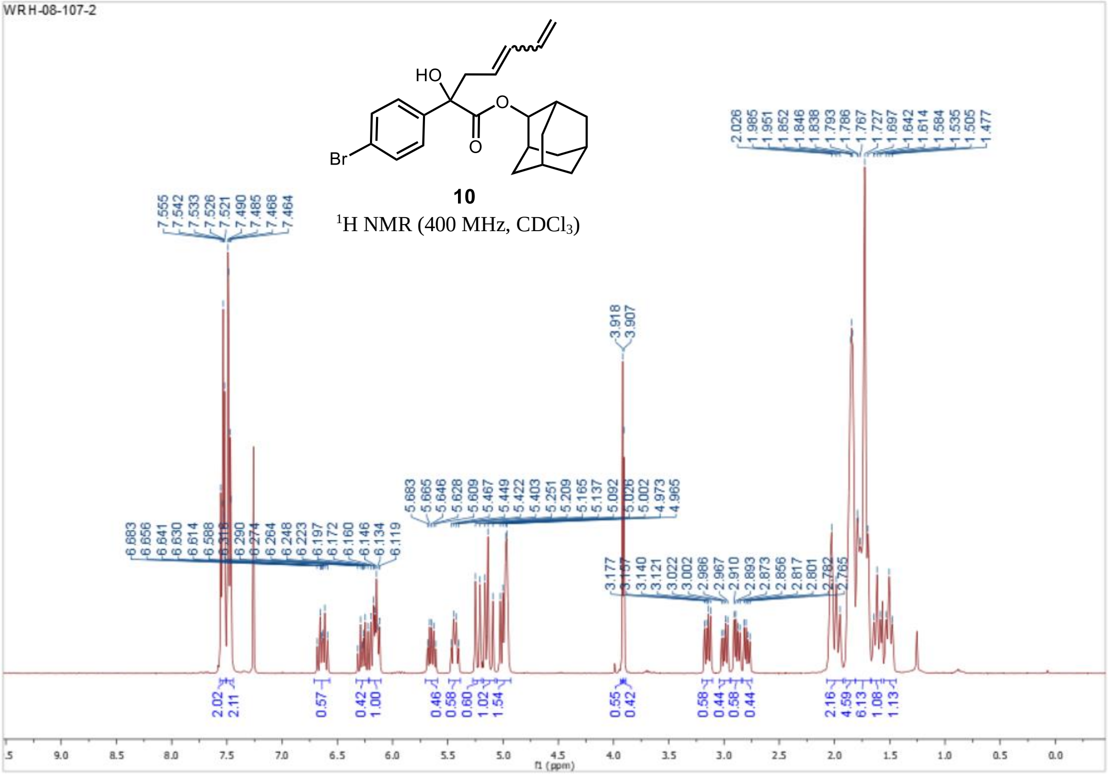
WRH-08-107-2
HO
O
Br
O
10
¹H NMR (400 MHz, CDCl₃)
7.555 7.542 7.533 7.526 7.521 7.490 7.485 7.468 7.464
6.683 6.656 6.641 6.630 6.614 6.588 6.318 6.290 6.274 6.264 6.248 6.223 6.197 6.172 6.160 6.146 6.134 6.119
5.683 5.665 5.646 5.628 5.609 5.467 5.449 5.422 5.403 5.251 5.209 5.165 5.137 5.092 5.026 5.002 4.973 4.965
3.918 3.907
3.177 3.157 3.140 3.121 3.022 3.002 2.986 2.967 2.910 2.893 2.873 2.856 2.817 2.801 2.782 2.765
2.026 1.985 1.951 1.852 1.846 1.838 1.793 1.786 1.767 1.727 1.697 1.642 1.614 1.584 1.535 1.505 1.477
2.02 2.11 0.57 0.42 1.00 0.46 0.58 0.60 1.02 1.54 0.55 0.42 0.58 0.44 0.58 0.44 2.16 4.59 6.13 1.08 1.13
f1 (ppm)

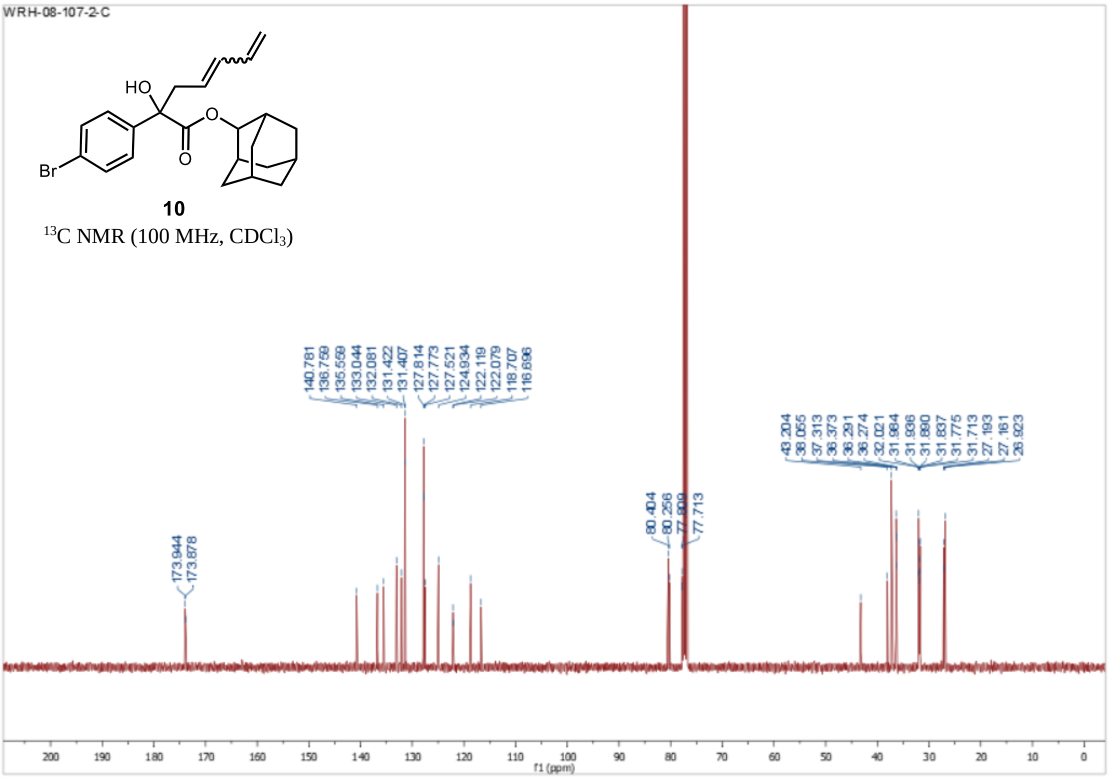
WRH-08-107-2-C
HO
O
Br
O
10
¹³C NMR (100 MHz, CDCl₃)
173.944 173.878
140.781 136.759 135.559 133.044 132.081 131.422 131.407 127.814 127.773 127.521 124.934 122.119 122.079 118.707 116.696
80.404 80.256 77.909 77.713
43.204 38.055 37.313 36.373 36.291 36.274 32.021 31.984 31.936 31.890 31.837 31.775 31.713 27.193 27.161 26.923
f1 (ppm)

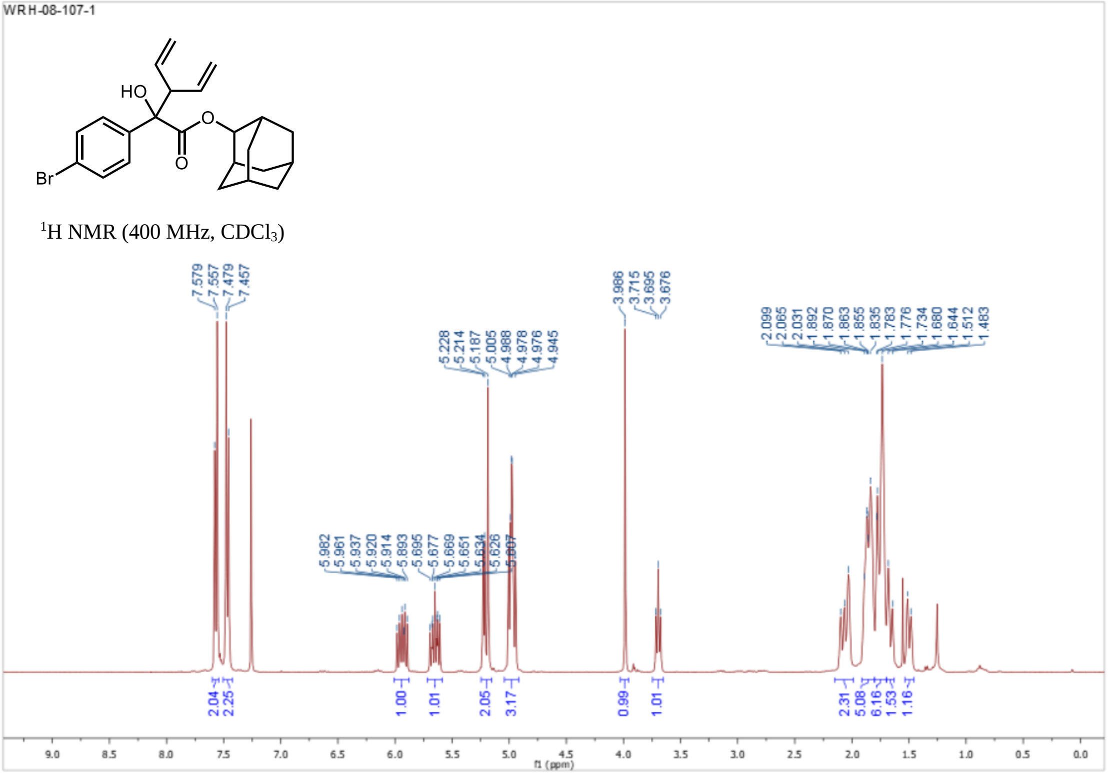
WRH-08-107-1
HO
O
O
Br
¹H NMR (400 MHz, CDCl₃)
f1 (ppm)

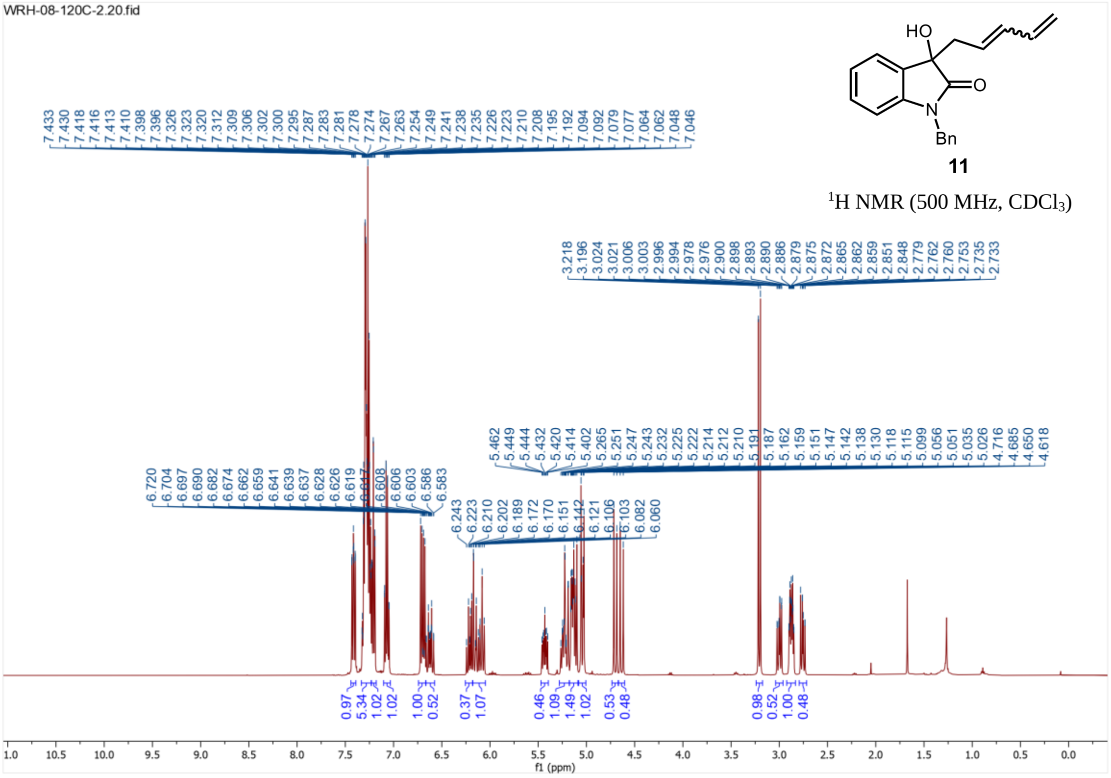
WRH-08-120C-2.20.fid
HO
O
N
Bn
11
¹H NMR (500 MHz, CDCl₃)
7.433 7.430 7.418 7.416 7.413 7.410 7.398 7.396 7.326 7.323 7.320 7.312 7.309 7.306 7.302 7.300 7.295 7.287 7.283 7.281 7.278 7.274 7.267 7.263 7.254 7.249 7.241 7.238 7.235 7.226 7.223 7.210 7.208 7.195 7.192 7.094 7.092 7.079 7.077 7.064 7.062 7.048 7.046
3.218 3.196 3.024 3.021 3.006 3.003 2.996 2.994 2.978 2.976 2.900 2.898 2.893 2.890 2.886 2.879 2.875 2.872 2.865 2.862 2.859 2.851 2.848 2.779 2.762 2.760 2.753 2.735 2.733
5.462 5.449 5.444 5.432 5.420 5.414 5.402 5.265 5.251 5.247 5.243 5.232 5.225 5.222 5.214 5.212 5.210 5.191 5.187 5.162 5.159 5.151 5.147 5.142 5.138 5.130 5.118 5.115 5.099 5.056 5.051 5.035 5.026 4.716 4.685 4.650 4.618
6.720 6.704 6.697 6.690 6.682 6.674 6.662 6.659 6.641 6.639 6.637 6.628 6.626 6.619 6.617 6.608 6.606 6.603 6.586 6.583
6.243 6.223 6.210 6.202 6.189 6.172 6.170 6.151 6.142 6.121 6.106 6.103 6.082 6.060
0.97 5.34 1.02 1.02 1.00 0.52 0.37 1.07 0.46 1.09 1.49 1.02 0.53 0.48 0.98 0.52 1.00 0.48
1.0 10.5 10.0 9.5 9.0 8.5 8.0 7.5 7.0 6.5 6.0 5.5 5.0 4.5 4.0 3.5 3.0 2.5 2.0 1.5 1.0 0.5 0.0
f1 (ppm)

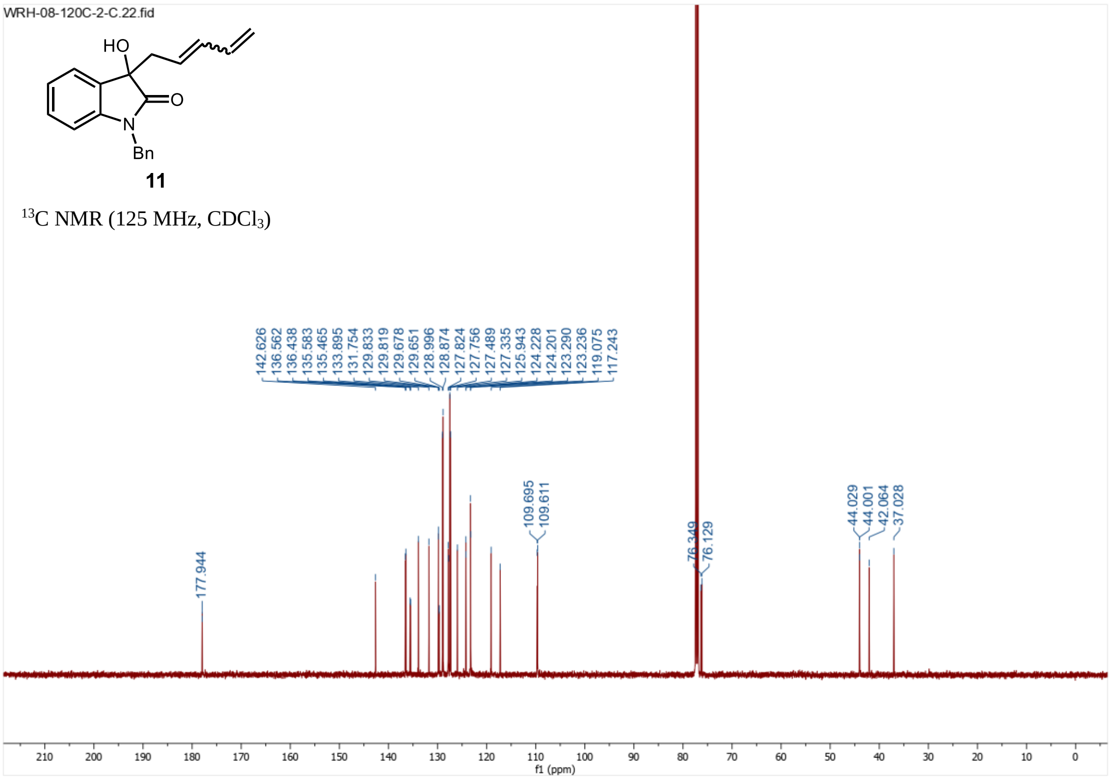
WRH-08-120C-2-C.22.fid
HO
O
N
Bn
11
¹³C NMR (125 MHz, CDCl₃)
142.626 136.562 136.438 135.583 135.465 133.895 131.754 129.833 129.819 129.678 129.651 128.996 128.874 127.824 127.756 127.489 127.335 125.943 124.228 124.201 123.290 123.236 119.075 117.243
177.944
109.695 109.611
76.349 76.129
44.029 44.001 42.064 37.028
210 200 190 180 170 160 150 140 130 120 110 100 90 80 70 60 50 40 30 20 10 0
f1 (ppm)

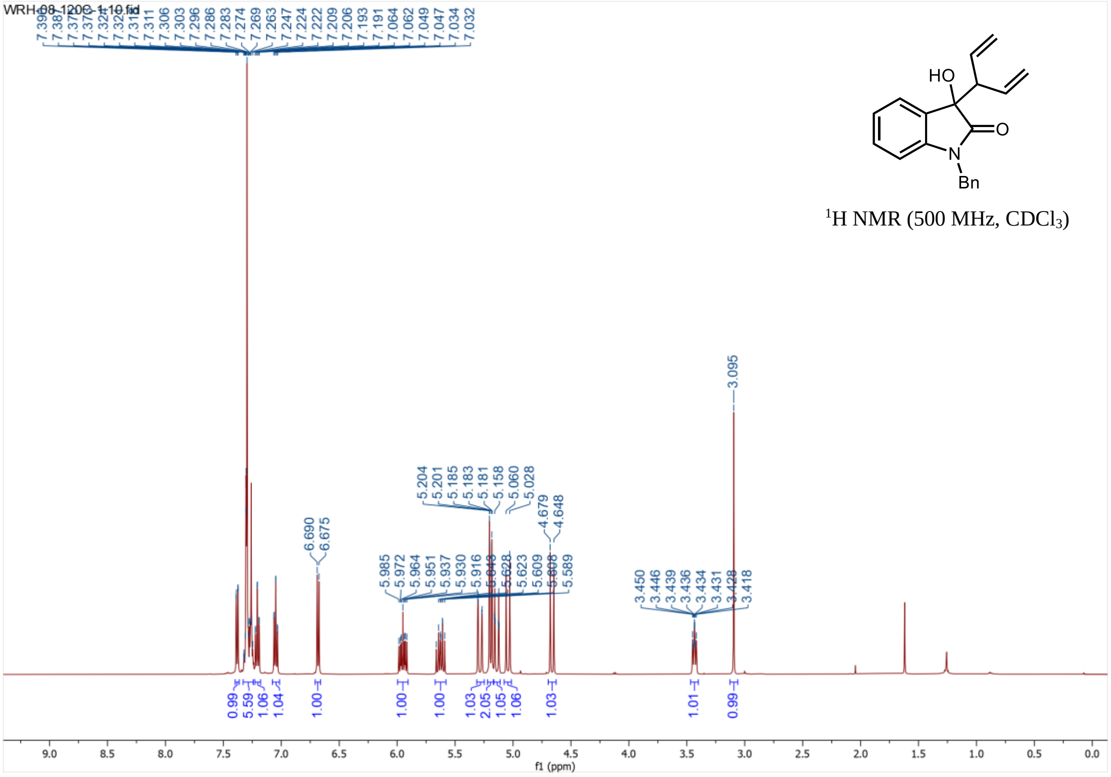

WRH-08-120C-1-10.fid
HO
O
N
Bn
1H NMR (500 MHz, CDCl3)
f1 (ppm)

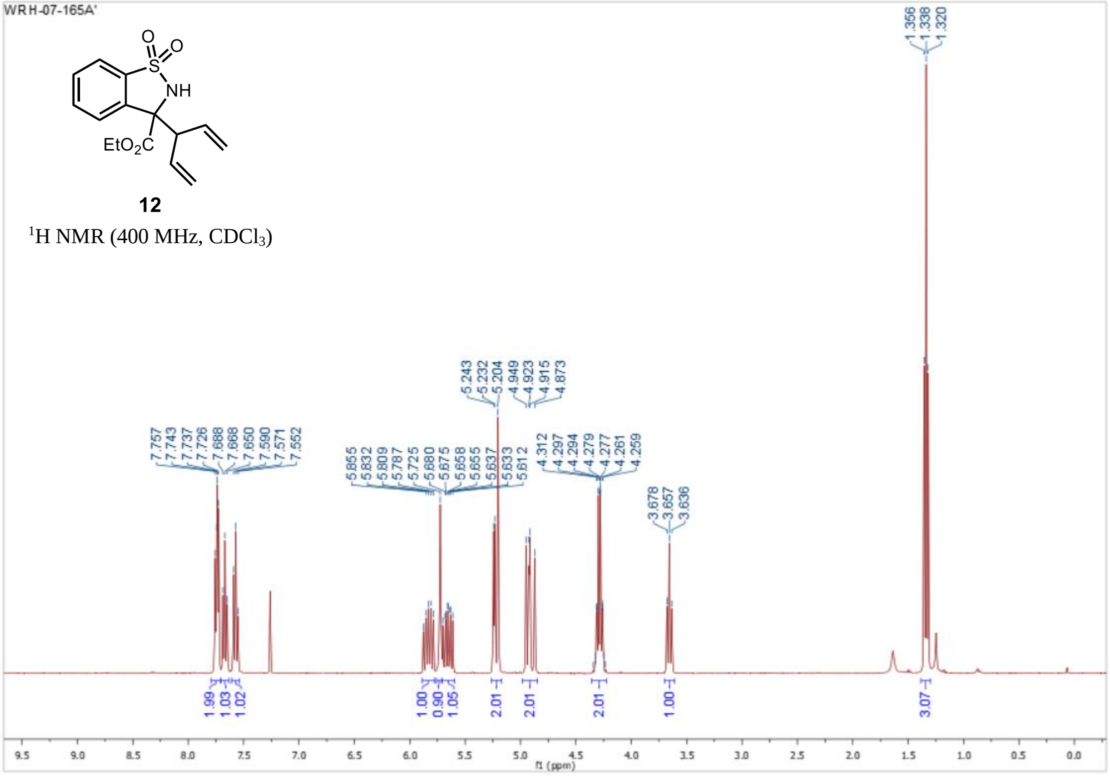
WRH-07-165A'
O
S
O
NH
EtO2C
12
1H NMR (400 MHz, CDCl3)
7.757 7.743 7.737 7.726 7.688 7.668 7.650 7.590 7.571 7.552
5.855 5.832 5.809 5.787 5.725 5.680 5.675 5.658 5.655 5.637 5.633 5.612
5.243 5.232 5.204 4.949 4.923 4.915 4.873
4.312 4.297 4.294 4.279 4.277 4.261 4.259
3.678 3.657 3.636
1.356 1.338 1.320
1.99 1.03 1.02 1.00 0.90 1.05 2.01 2.01 2.01 1.00 3.07
9.5 9.0 8.5 8.0 7.5 7.0 6.5 6.0 5.5 5.0 4.5 4.0 3.5 3.0 2.5 2.0 1.5 1.0 0.5 0.0
f1 (ppm)

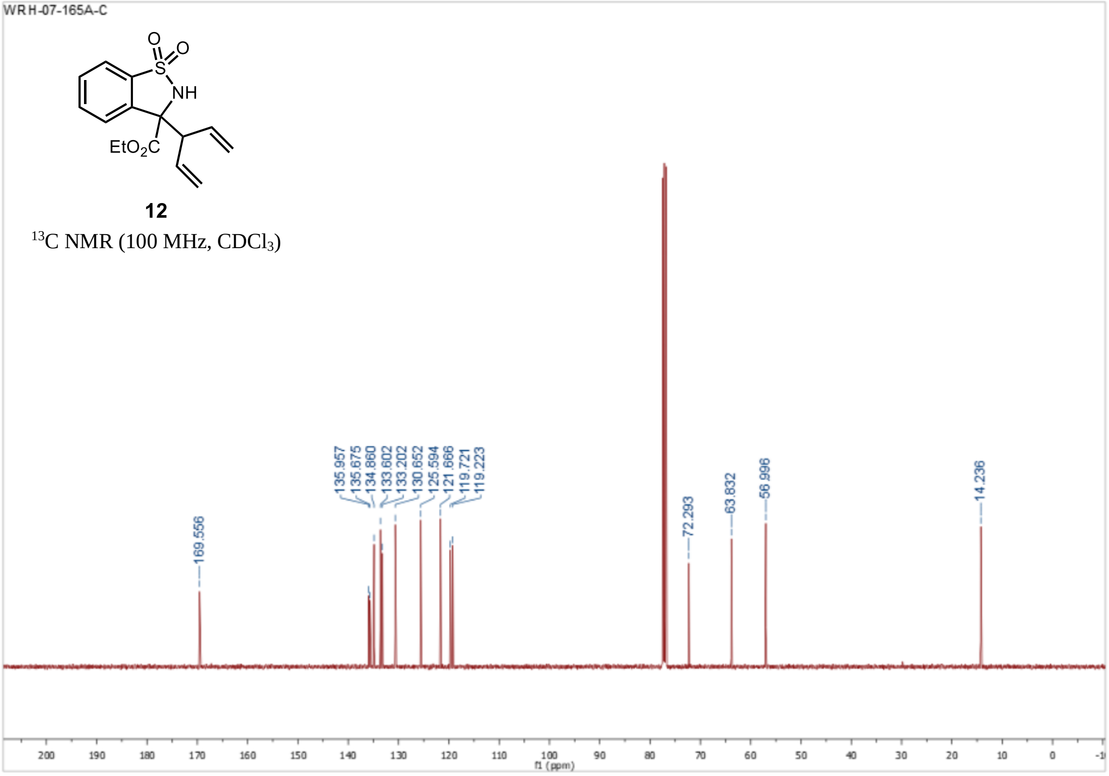
WRH-07-165A-C
O
S
O
NH
EtO2C
12
13C NMR (100 MHz, CDCl3)
169.556
135.957 135.675 134.860 133.602 133.202 130.652 125.594 121.686 119.721 119.223
72.293
63.832
56.996
14.236
200 190 180 170 160 150 140 130 120 110 100 90 80 70 60 50 40 30 20 10 0
f1 (ppm)

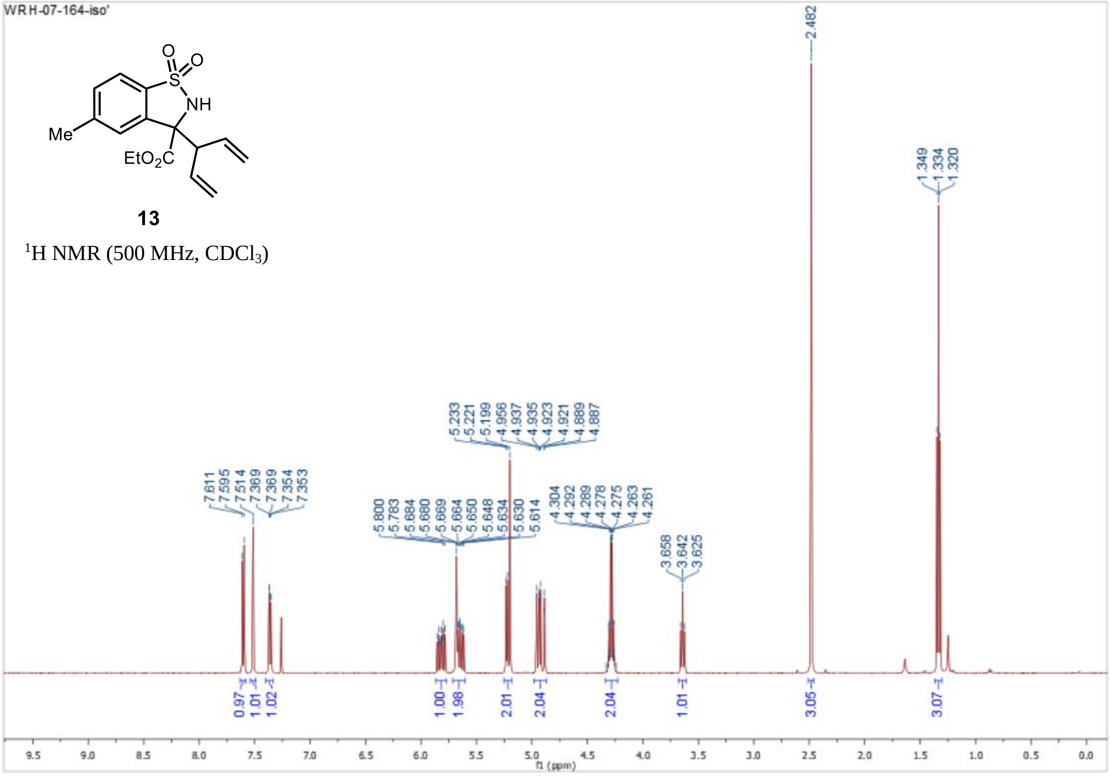

13

^{1}H NMR (500 MHz, $CDCl_3$)

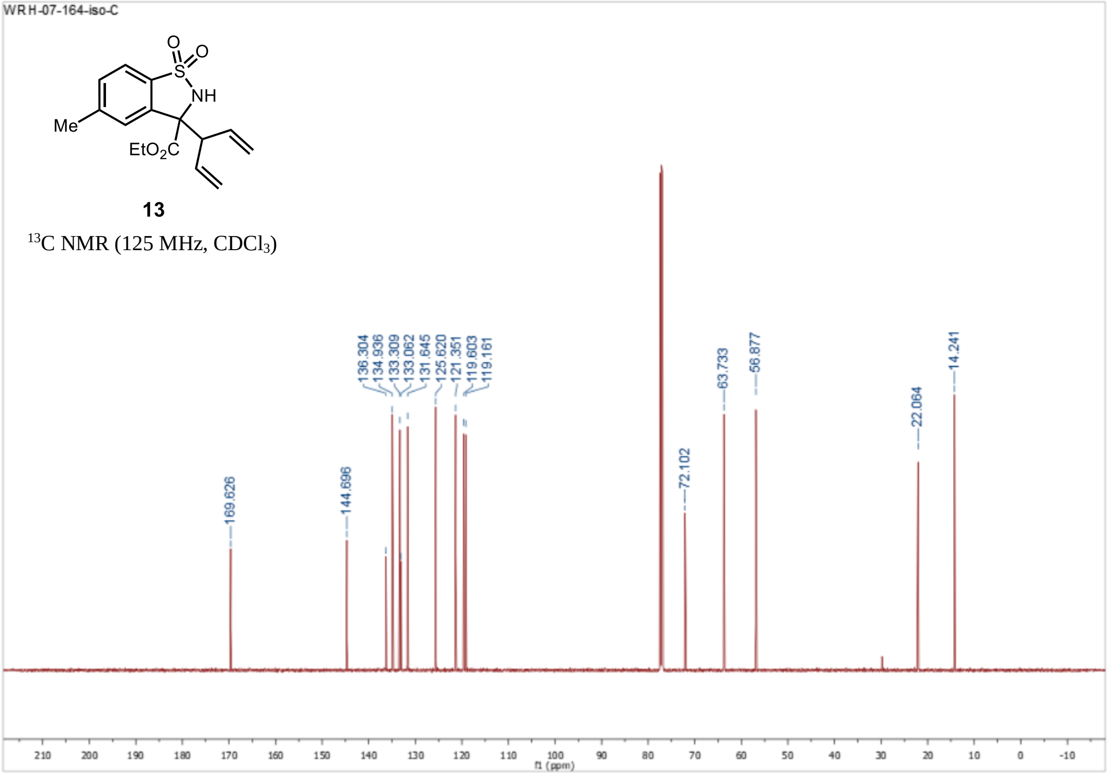

13

^{13}C NMR (125 MHz, $CDCl_3$)

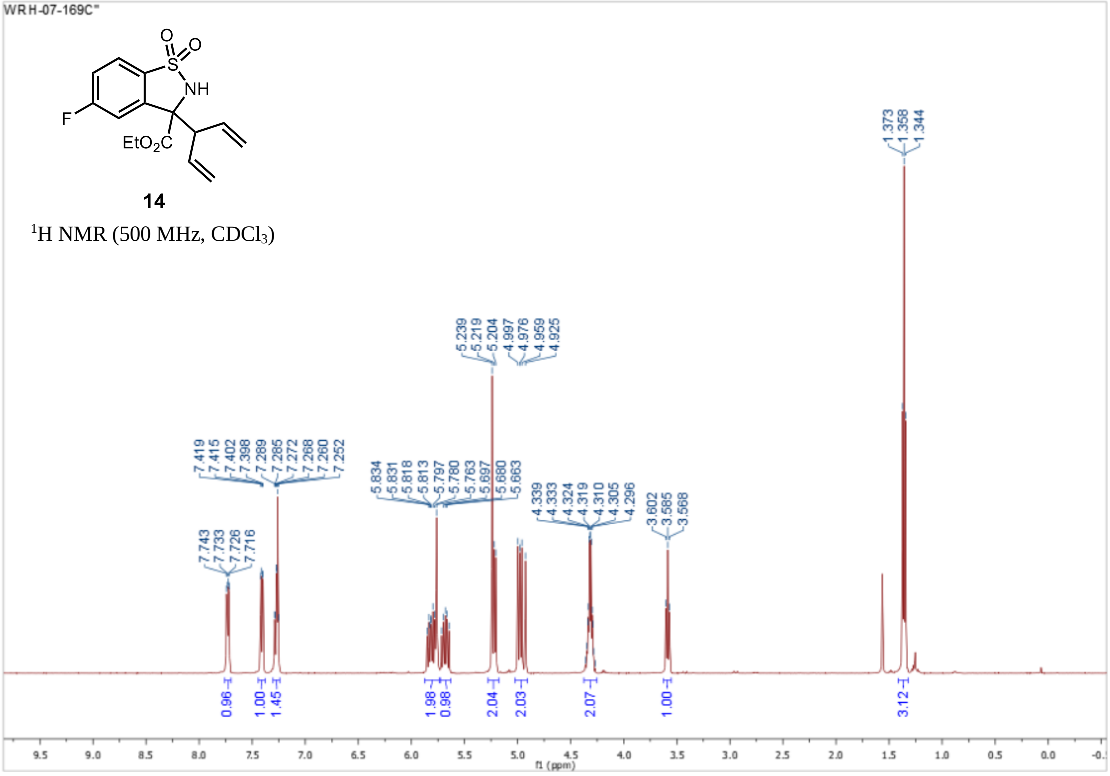
WRH-07-169C"
O
O
S
NH
F
EtO2C
14
1H NMR (500 MHz, CDCl3)
7.743 7.733 7.726 7.716
7.419 7.415 7.402 7.398 7.289 7.285 7.272 7.268 7.260 7.252
5.834 5.831 5.818 5.813 5.797 5.780 5.763 5.697 5.680 5.663
5.239 5.219 5.204 4.997 4.976 4.959 4.925
4.339 4.333 4.324 4.319 4.310 4.305 4.296
3.602 3.585 3.568
1.373 1.358 1.344
0.96 1.00 1.45 1.98 0.98 2.04 2.03 2.07 1.00 3.12
f1 (ppm)

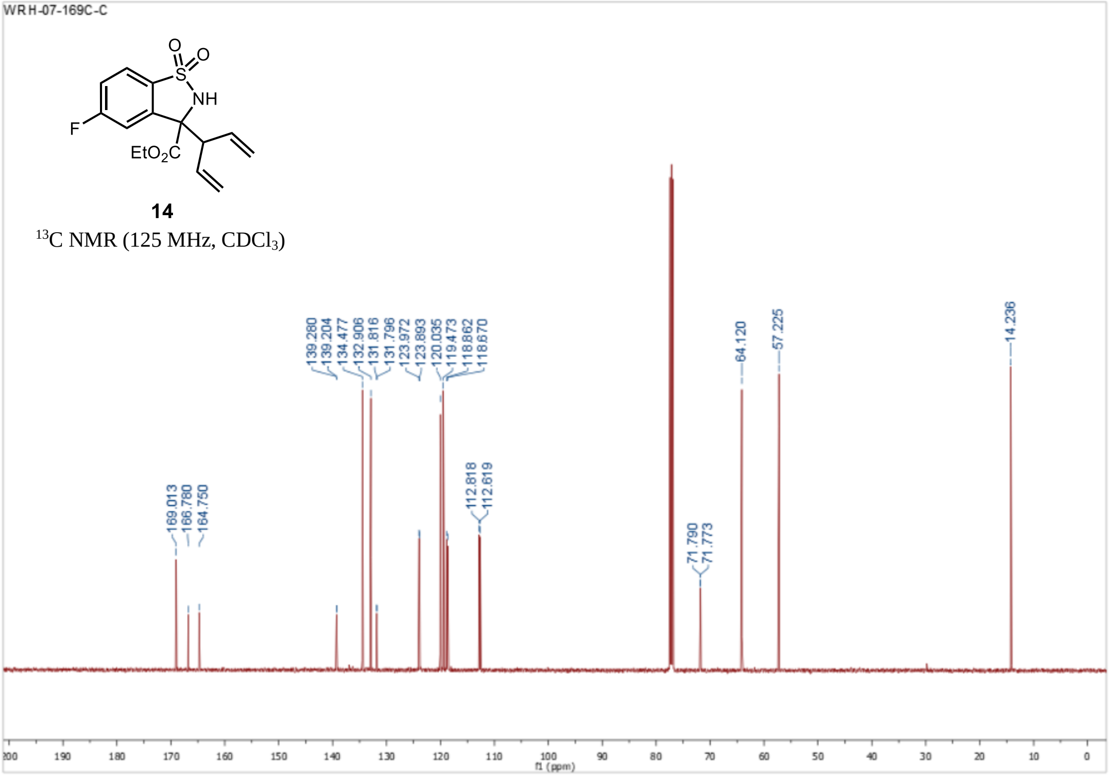
WRH-07-169C-C
O
O
S
NH
F
EtO2C
14
13C NMR (125 MHz, CDCl3)
169.013 166.780 164.750
139.260 139.204 134.477 132.906 131.816 131.796 123.972 123.893 120.035 119.473 118.862 118.670
112.818 112.619
71.790 71.773
64.120
57.225
14.236
f1 (ppm)

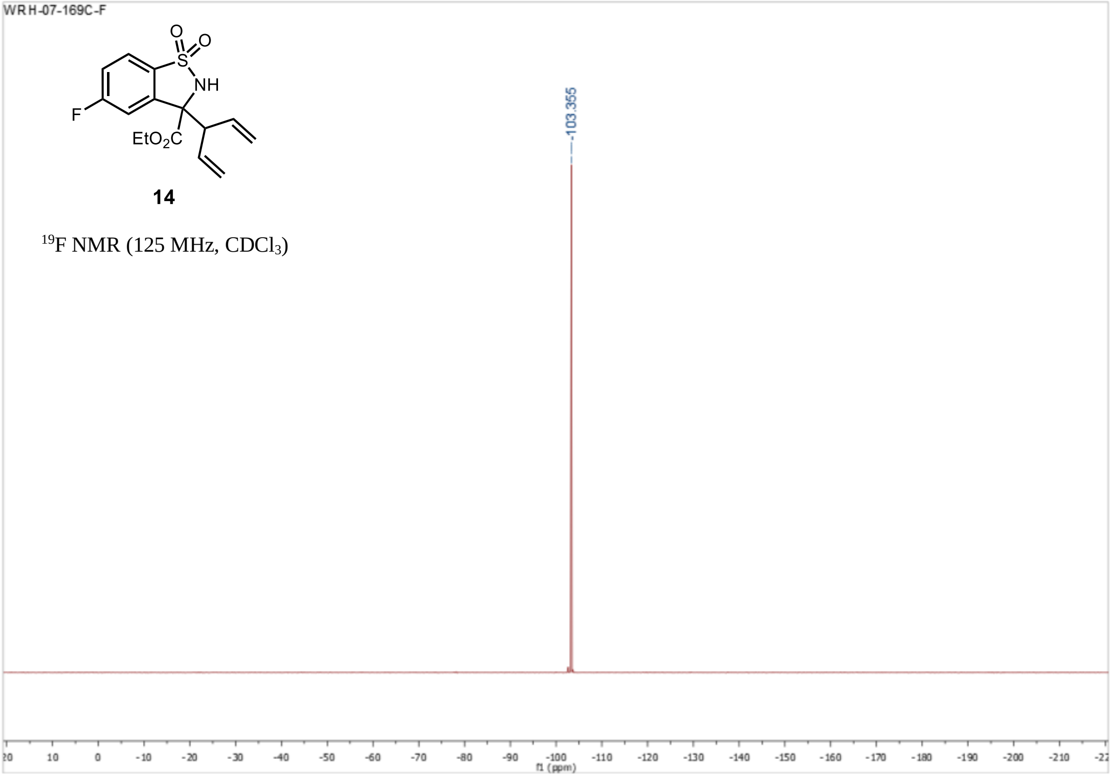

WRH-07-169C-F
14
^{19}F NMR (125 MHz, CDCl$_3$)
-103.355
f1 (ppm)

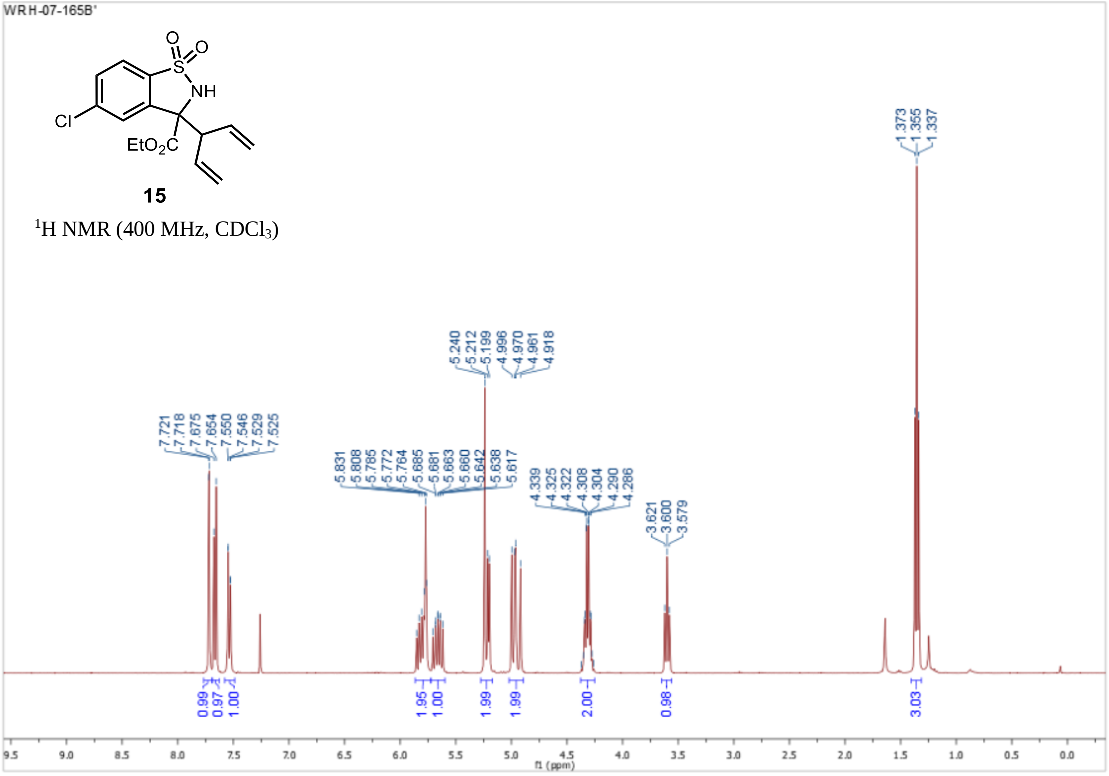

15

^{1}H NMR (400 MHz, $CDCl_3$)

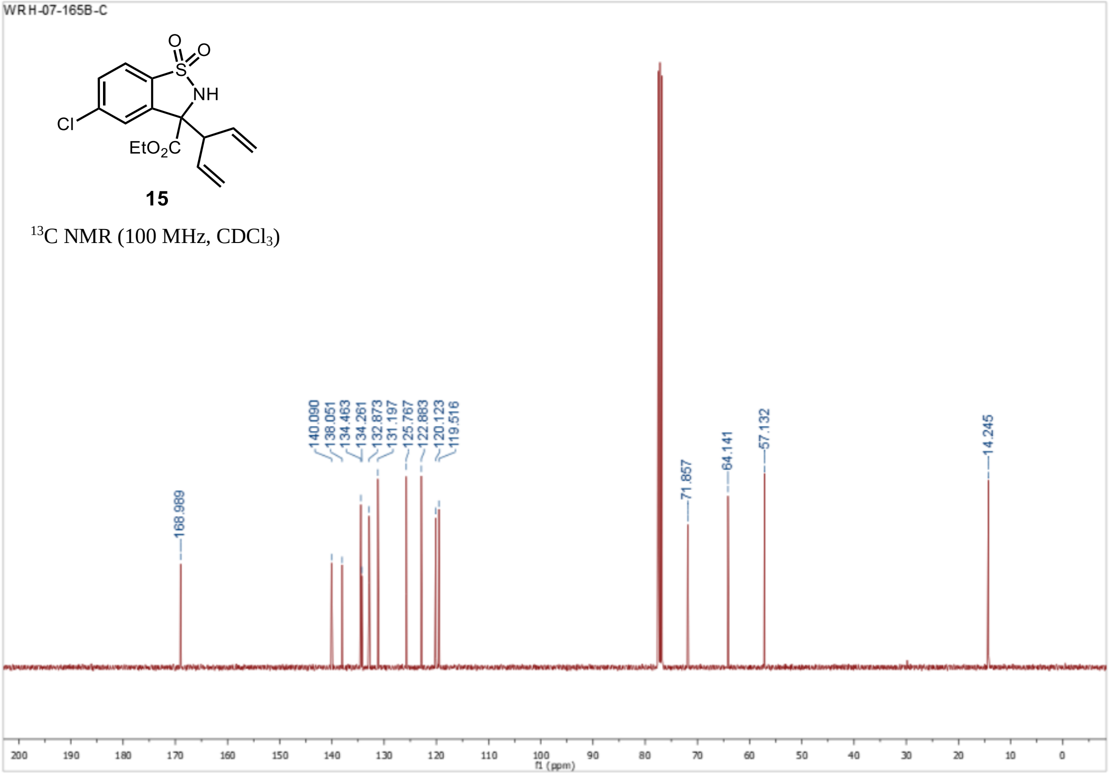

15

^{13}C NMR (100 MHz, $CDCl_3$)

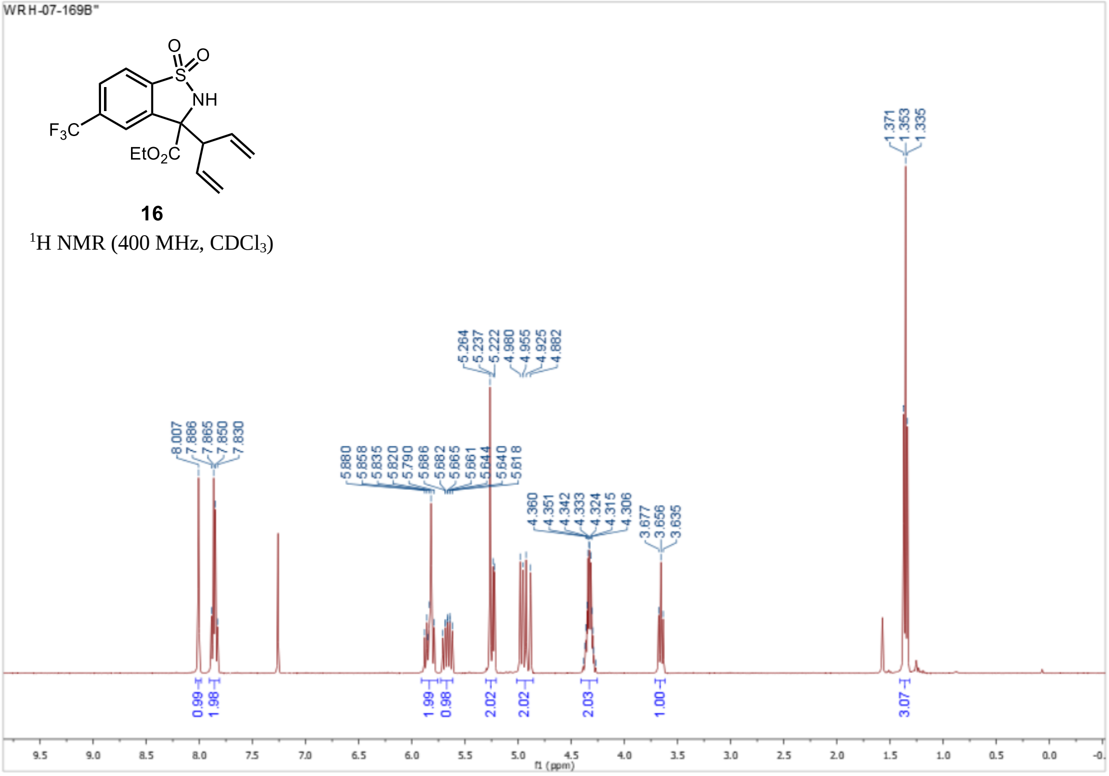
WRH-07-169B"
16
1H NMR (400 MHz, CDCl3)
f1 (ppm)

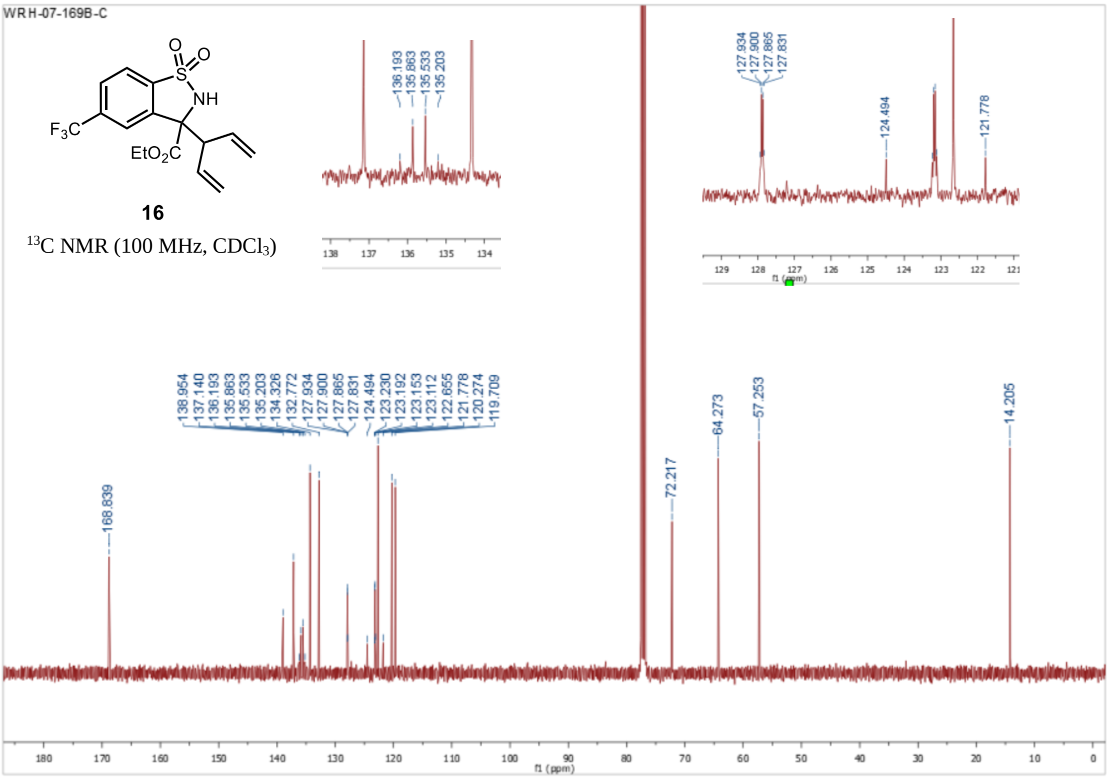
WRH-07-169B-C
16
13C NMR (100 MHz, CDCl3)
f1 (ppm)

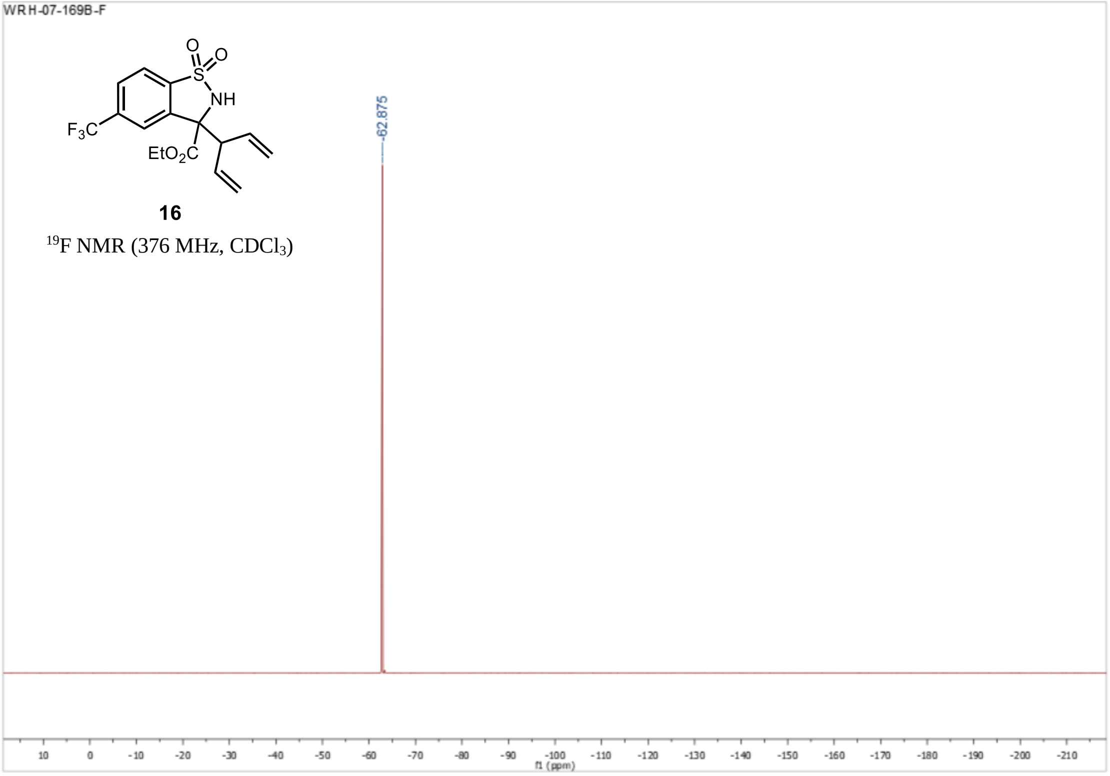
WRH-07-169B-F
F3C
EtO2C
NH
S
O
O
16
19F NMR (376 MHz, CDCl3)
-62.875
f1 (ppm)

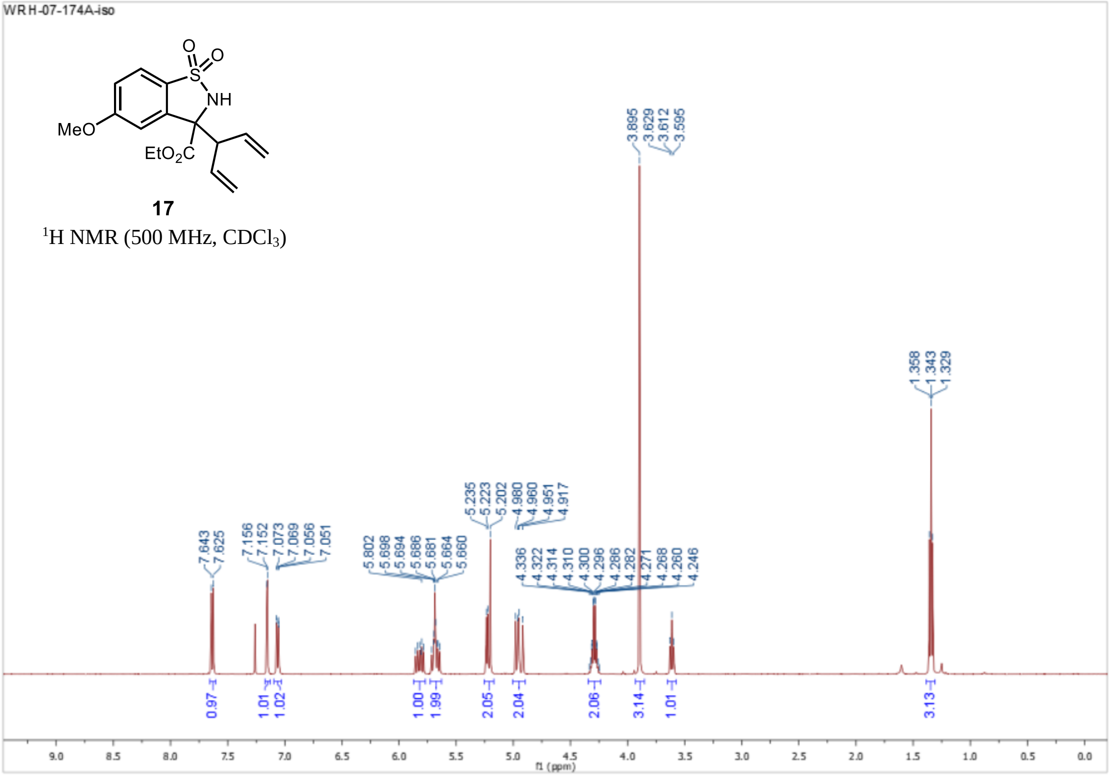
WRH-07-174A-iso
MeO
EtO2C
NH
17
1H NMR (500 MHz, CDCl3)
7.643
7.625
7.156
7.152
7.073
7.069
7.056
7.051
5.802
5.698
5.694
5.686
5.681
5.664
5.660
5.235
5.223
5.202
4.980
4.960
4.951
4.917
4.336
4.322
4.314
4.310
4.300
4.296
4.286
4.282
4.271
4.268
4.260
4.246
3.895
3.629
3.612
3.595
1.358
1.343
1.329
0.97
1.01
1.02
1.00
1.99
2.05
2.04
2.06
3.14
1.01
3.13
f1 (ppm)

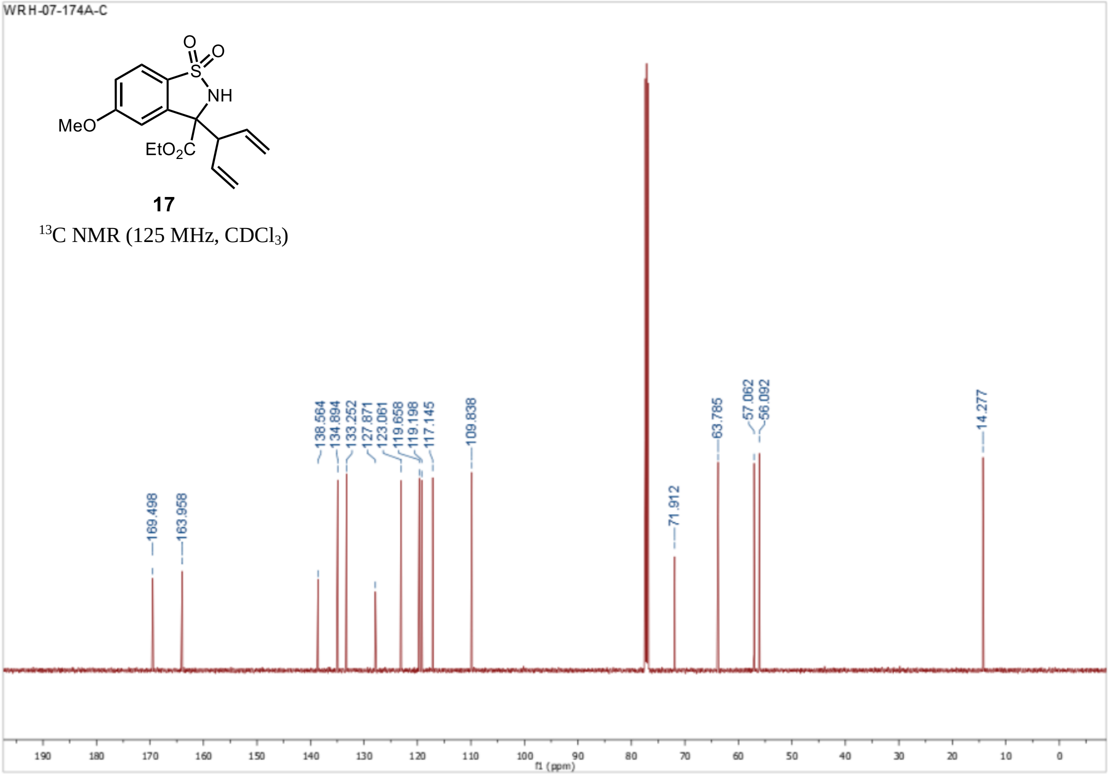
WRH-07-174A-C
MeO
EtO2C
NH
17
13C NMR (125 MHz, CDCl3)
169.498
163.958
138.564
134.894
133.252
127.871
123.061
119.658
119.198
117.145
109.838
71.912
63.785
57.062
56.092
14.277
f1 (ppm)

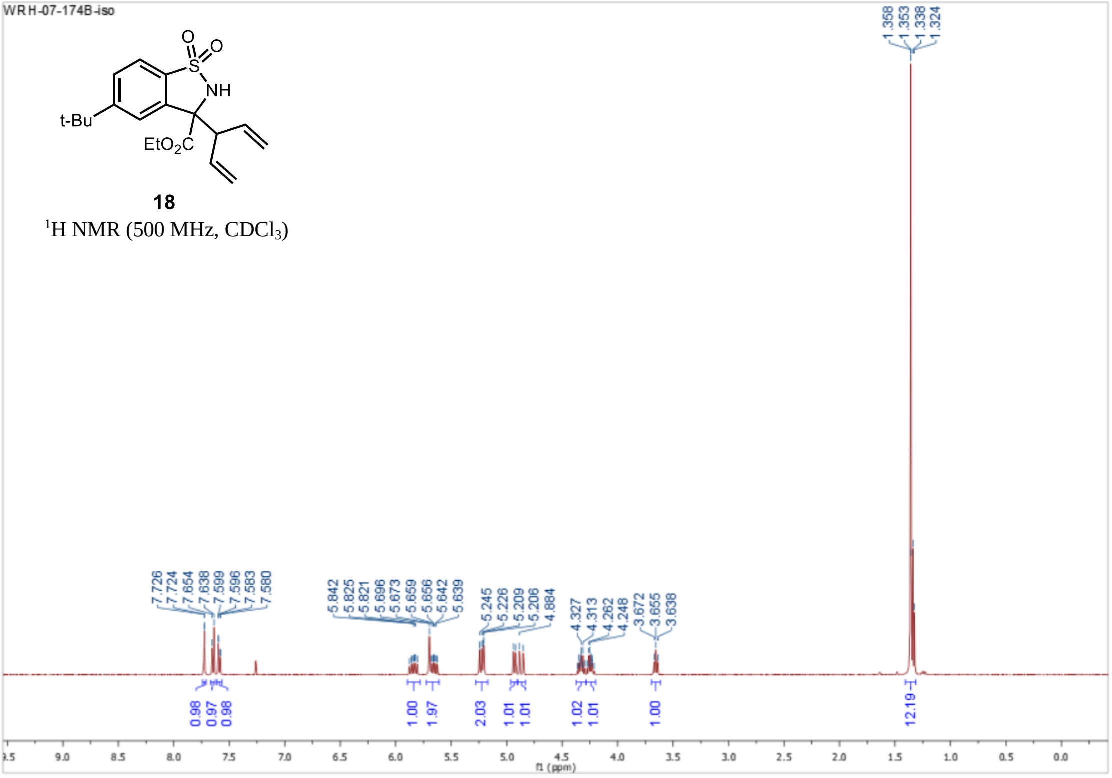
WRH-07-174B-iso
t-Bu
EtO_2C
NH
18
^1H NMR (500 MHz, $CDCl_3$)
7.726 7.724 7.654 7.638 7.599 7.596 7.583 7.580
5.842 5.825 5.821 5.696 5.673 5.659 5.656 5.642 5.639
5.245 5.226 5.209 5.206 4.884
4.327 4.313 4.262 4.248 3.672 3.655 3.638
1.358 1.353 1.338 1.324
0.98 0.97 0.98 1.00 1.97 2.03 1.01 1.01 1.02 1.01 1.00 12.19
f1 (ppm)

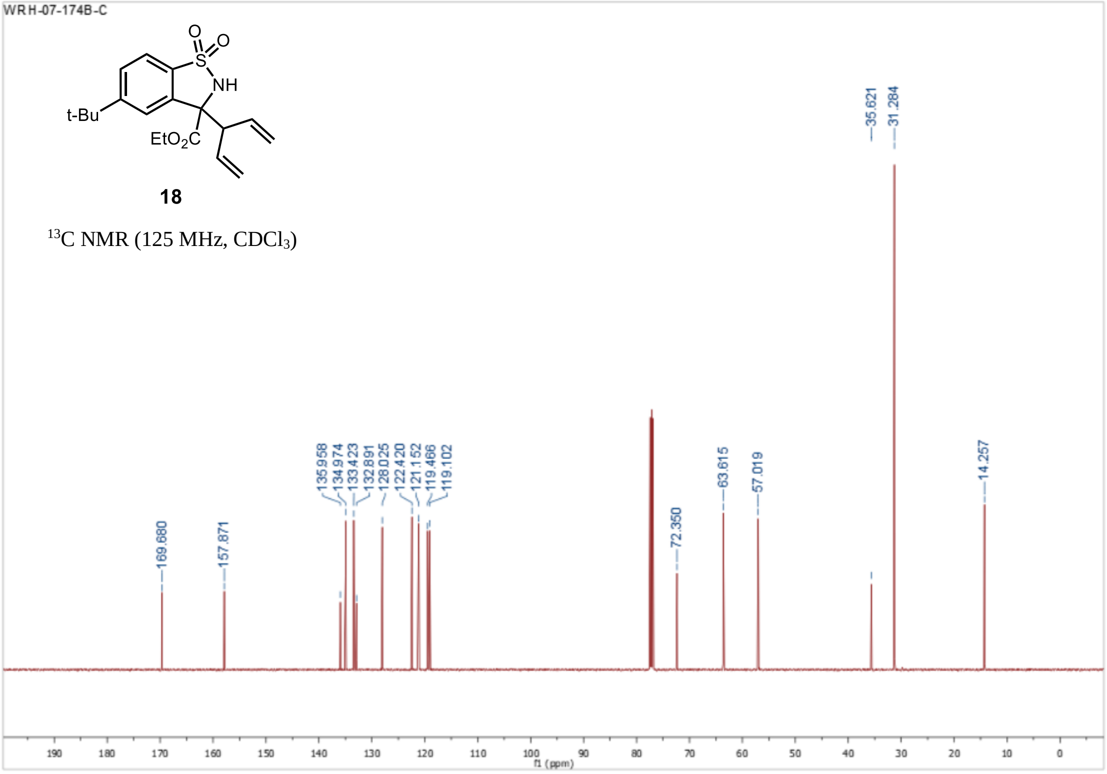
WRH-07-174B-C
t-Bu
EtO_2C
NH
18
^{13}C NMR (125 MHz, $CDCl_3$)
169.680
157.871
135.958 134.974 133.423 132.891 128.025 122.420 121.152 119.466 119.102
72.350
63.615
57.019
35.621
31.284
14.257
f1 (ppm)

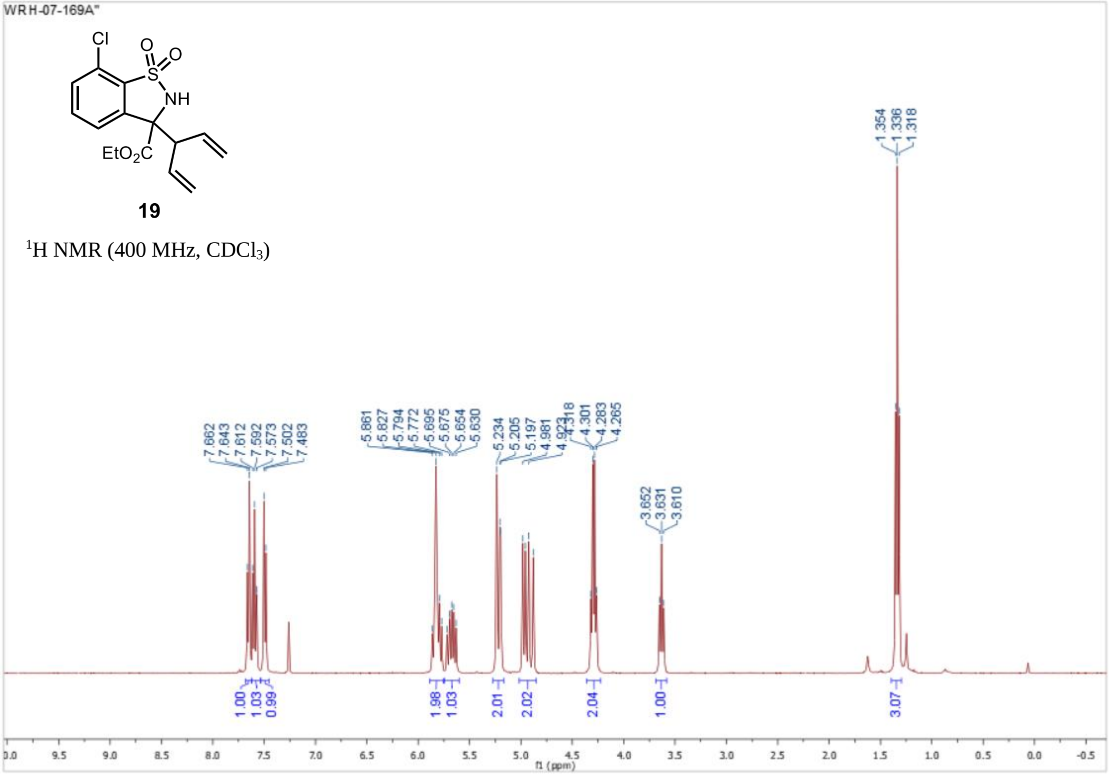
WRH-07-169A"
Cl
O
S
O
NH
EtO2C
19
1H NMR (400 MHz, CDCl3)
f1 (ppm)

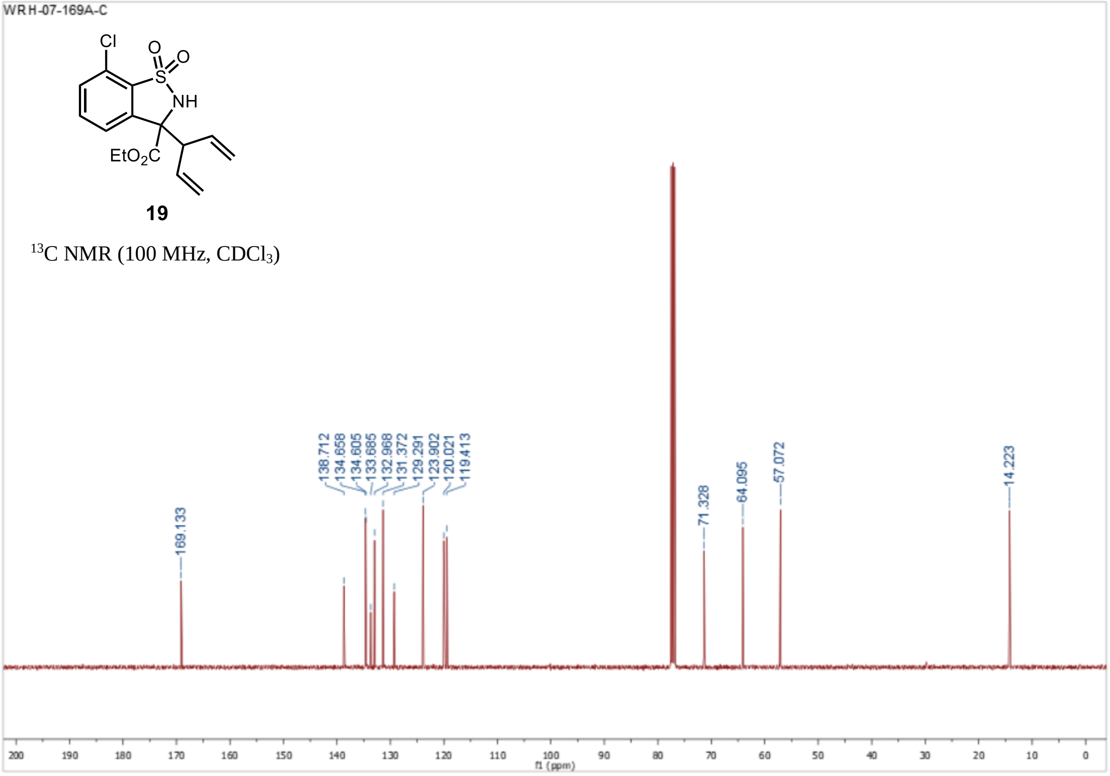
WRH-07-169A-C
Cl
O
S
O
NH
EtO2C
19
13C NMR (100 MHz, CDCl3)
f1 (ppm)

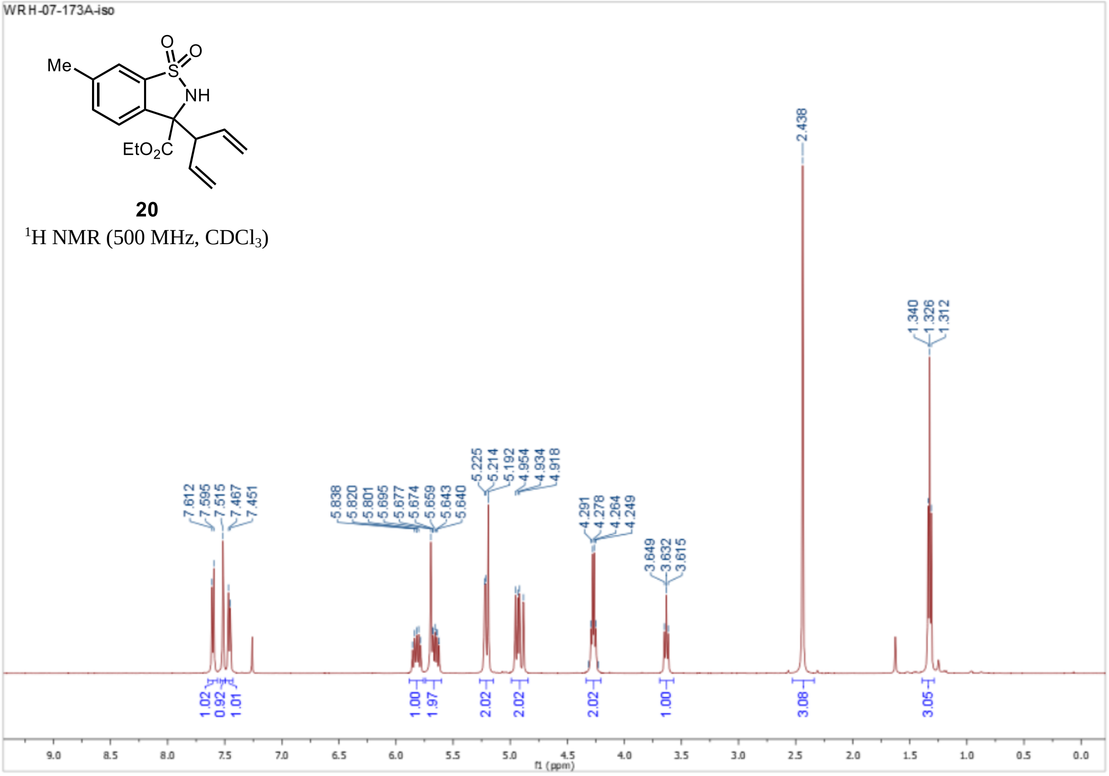
WRH-07-173A-iso
Me
O
O
S
NH
EtO2C
20
1H NMR (500 MHz, CDCl3)
7.612
7.595
7.515
7.467
7.451
5.838
5.820
5.801
5.695
5.677
5.674
5.659
5.643
5.640
5.225
5.214
5.192
4.954
4.934
4.918
4.291
4.278
4.264
4.249
3.649
3.632
3.615
2.438
1.340
1.326
1.312
1.02
0.92
1.01
1.00
1.97
2.02
2.02
2.02
1.00
3.08
3.05
f1 (ppm)

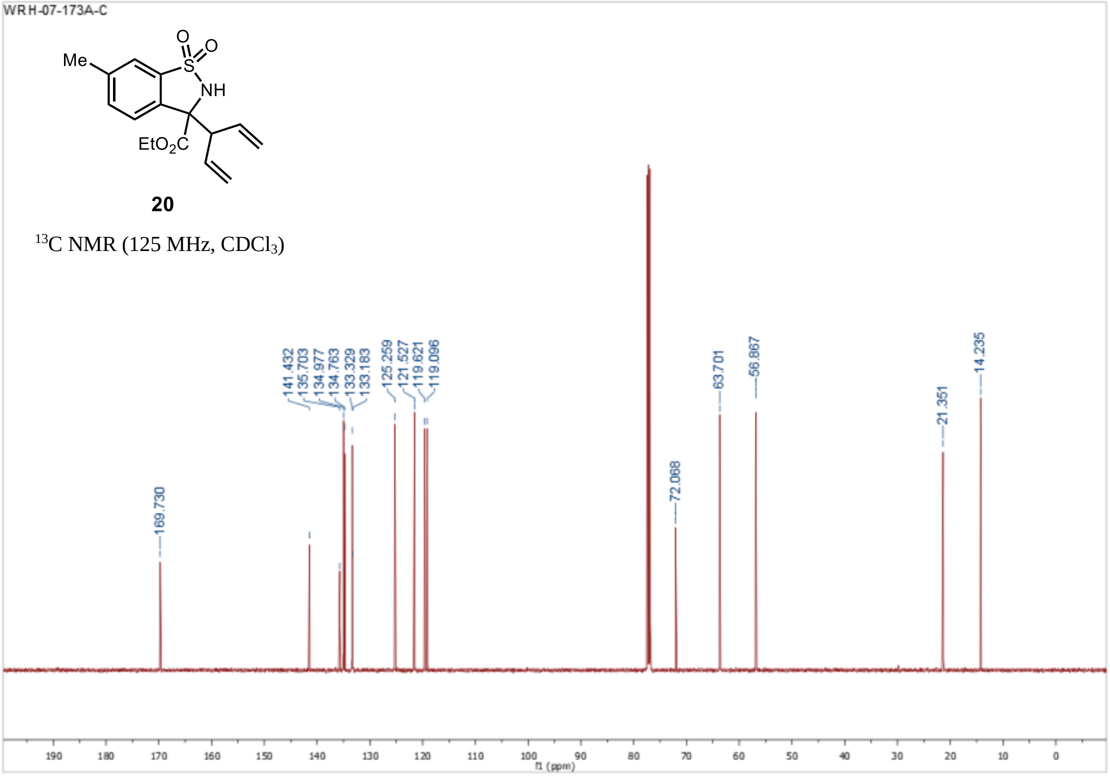
WRH-07-173A-C
Me
O
O
S
NH
EtO2C
20
13C NMR (125 MHz, CDCl3)
169.730
141.432
135.703
134.977
134.763
133.329
133.183
125.259
121.527
119.621
119.096
72.068
63.701
56.867
21.351
14.235
f1 (ppm)

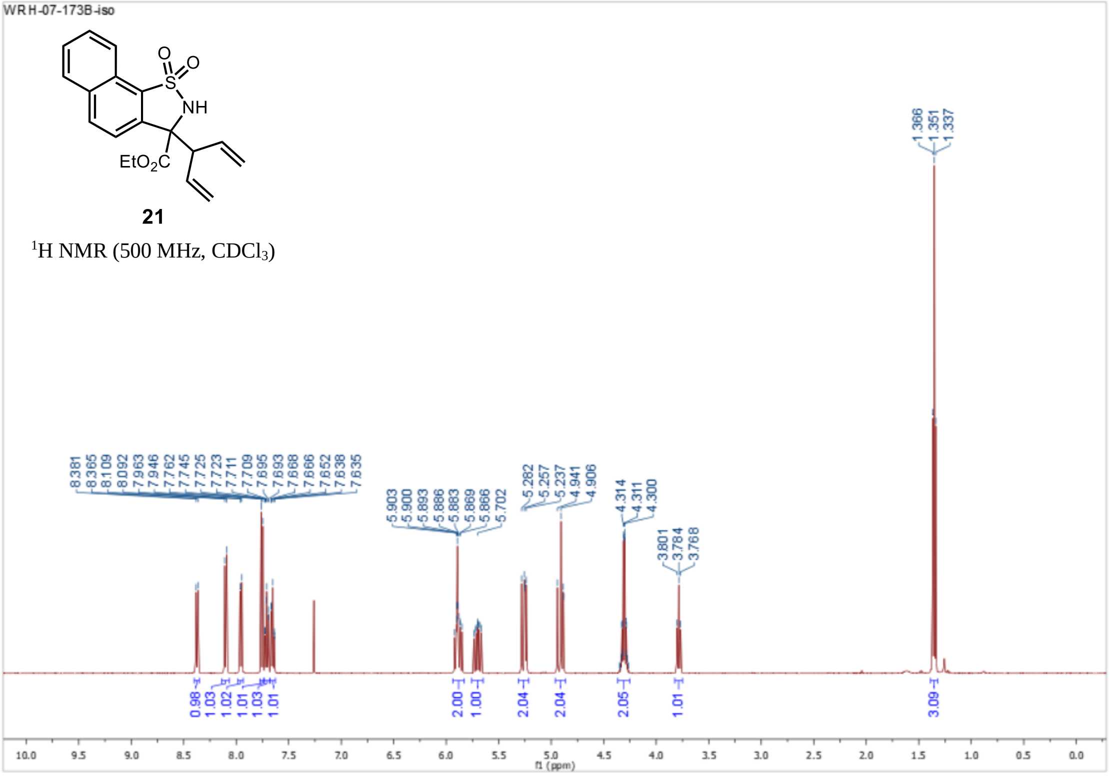
WRH-07-173B-iso
21
1H NMR (500 MHz, CDCl3)

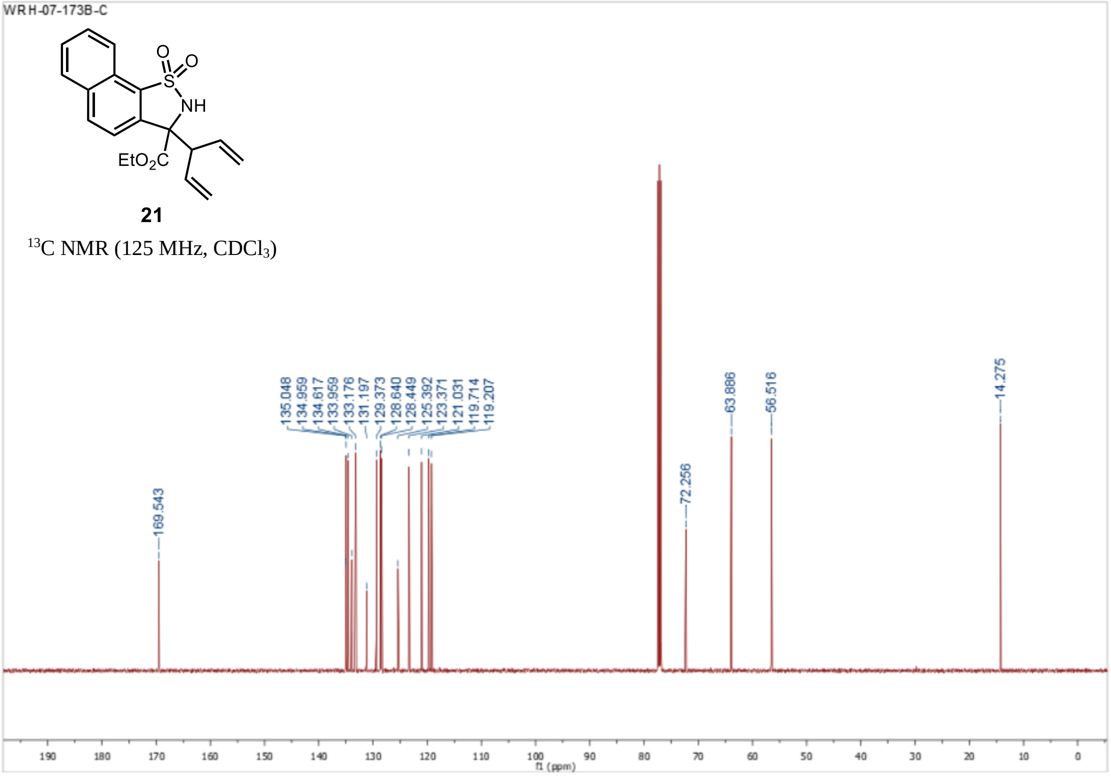
WRH-07-173B-C
21
13C NMR (125 MHz, CDCl3)

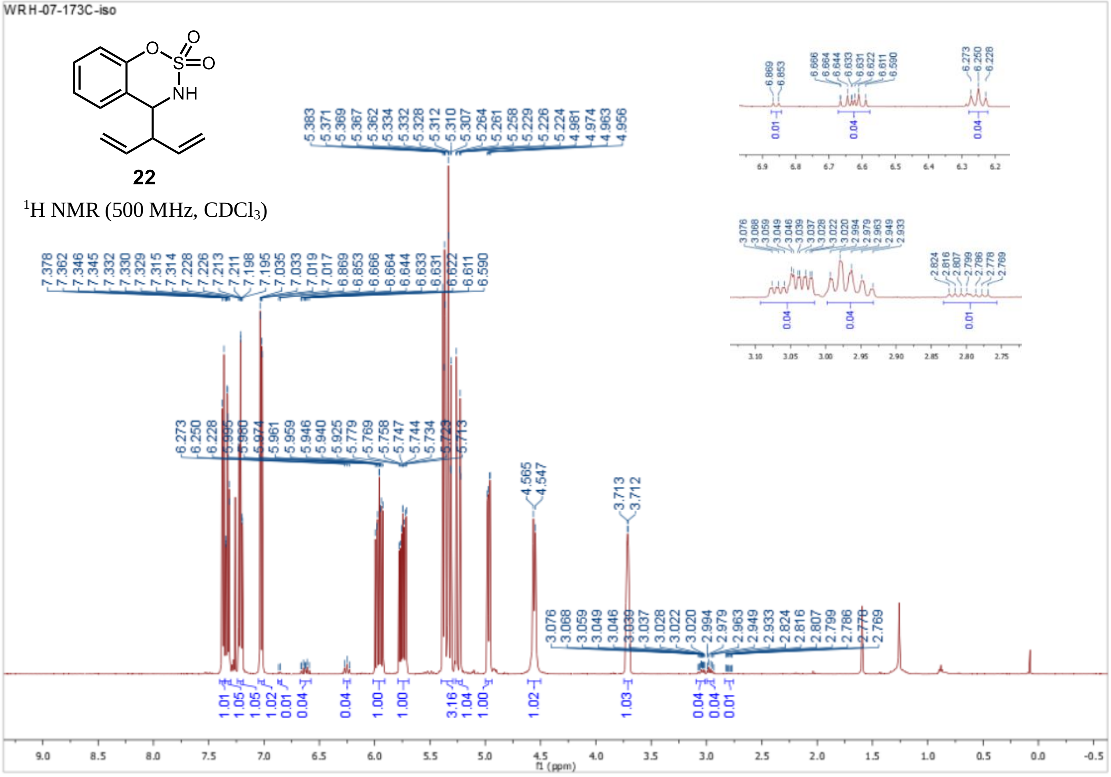
WRH-07-173C-iso
22
1H NMR (500 MHz, CDCl3)
f1 (ppm)

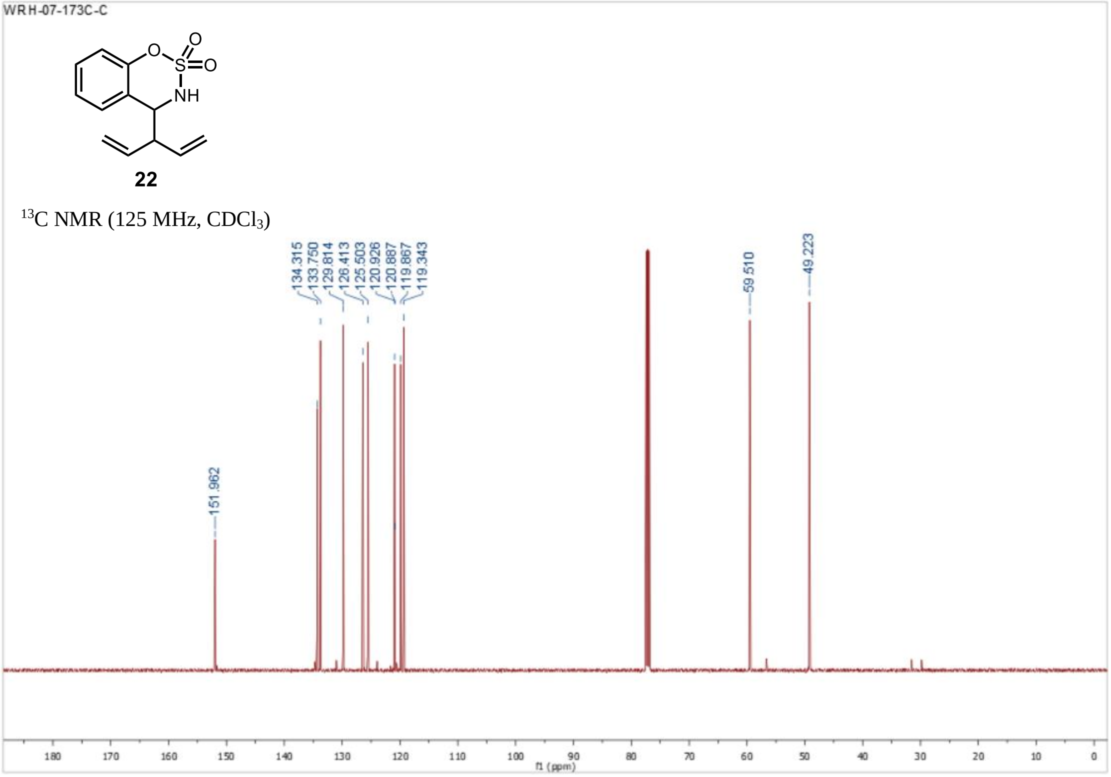
WRH-07-173C-C
22
13C NMR (125 MHz, CDCl3)
151.962
134.315
133.750
129.814
126.413
125.503
120.926
120.887
119.867
119.343
59.510
49.223
f1 (ppm)

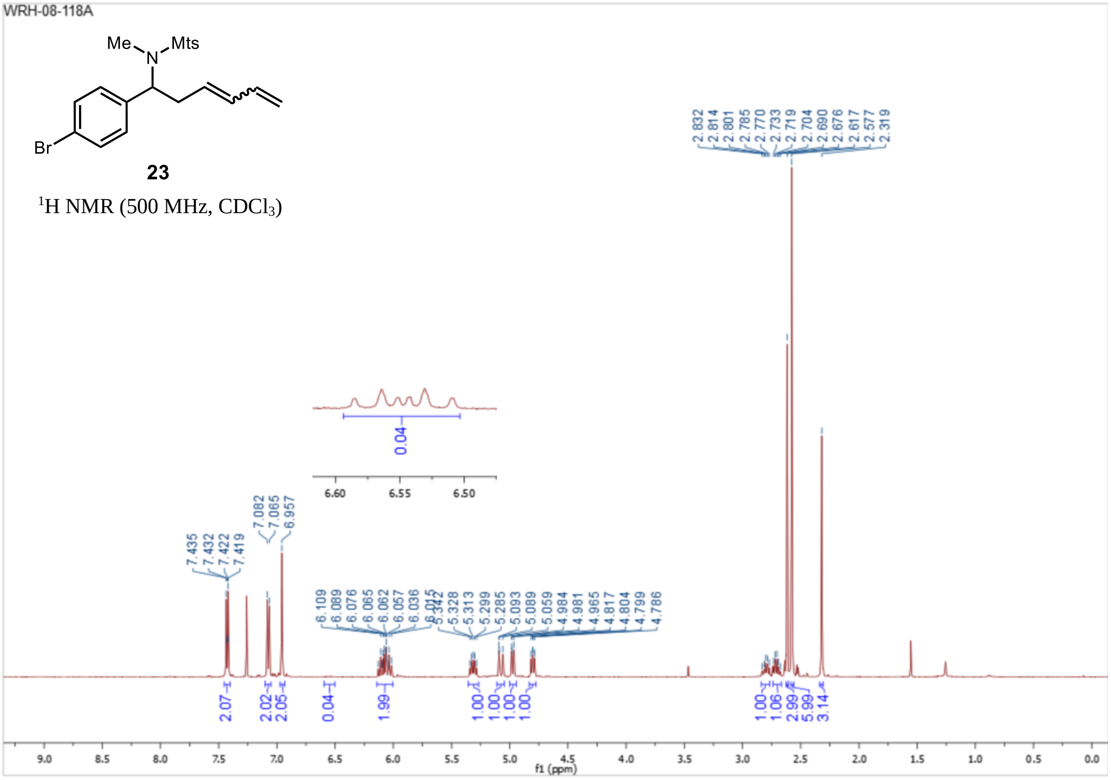
WRH-08-118A
Me
N
Mts
Br
23
1H NMR (500 MHz, CDCl3)
2.832 2.814 2.801 2.785 2.770 2.733 2.719 2.704 2.690 2.676 2.617 2.577 2.319
7.435 7.432 7.422 7.419
7.082 7.065 6.957
6.109 6.089 6.076 6.065 6.062 6.057 6.036 6.015 5.342 5.328 5.313 5.299 5.285 5.093 5.089 5.059 4.984 4.981 4.965 4.817 4.804 4.799 4.786
0.04
6.60 6.55 6.50
2.07 2.02 2.05 0.04 1.99 1.00 1.00 1.00 1.00 1.00 1.06 2.99 5.99 3.14
9.0 8.5 8.0 7.5 7.0 6.5 6.0 5.5 5.0 4.5 4.0 3.5 3.0 2.5 2.0 1.5 1.0 0.5 0.0
f1 (ppm)

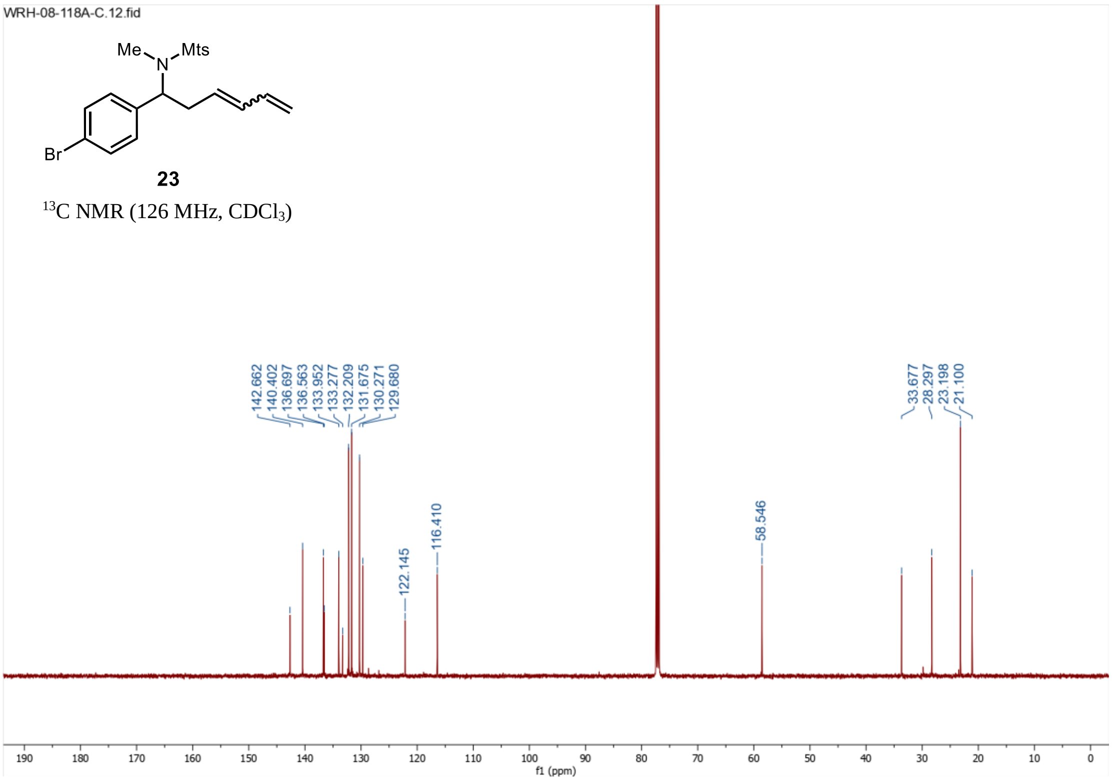
WRH-08-118A-C.12.fid
Me
N
Mts
Br
23
13C NMR (126 MHz, CDCl3)
142.662 140.402 136.697 136.563 133.952 133.277 132.209 131.675 130.271 129.680
122.145
116.410
58.546
33.677 28.297 23.198 21.100
190 180 170 160 150 140 130 120 110 100 90 80 70 60 50 40 30 20 10 0
f1 (ppm)

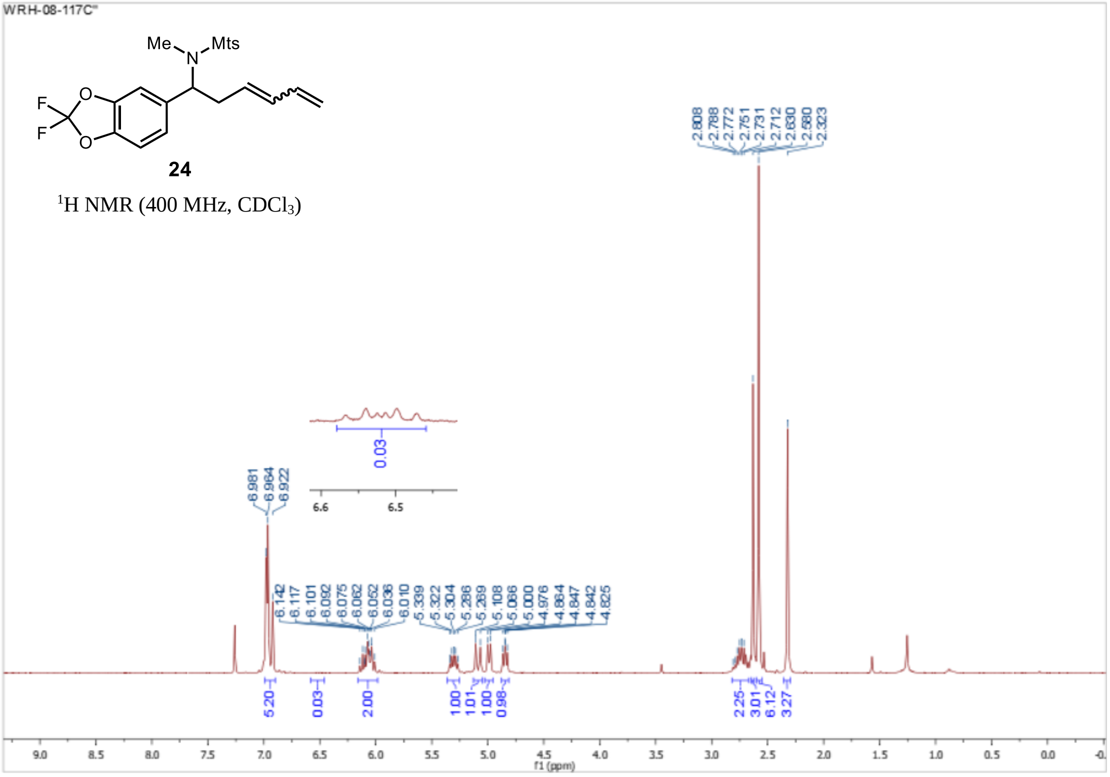
WRH-08-117C
Me
N
Mts
F
F
O
O
24
¹H NMR (400 MHz, CDCl₃)
6.981
6.964
6.922
6.142
6.117
6.101
6.092
6.075
6.062
6.062
6.036
6.010
5.339
5.322
5.304
5.286
5.269
5.108
5.086
5.000
4.976
4.864
4.847
4.842
4.825
2.808
2.788
2.772
2.751
2.731
2.712
2.630
2.580
2.323
0.03
6.6
6.5
5.20
0.03
2.00
1.00
1.01
1.00
0.98
2.25
3.01
6.12
3.27
f1 (ppm)

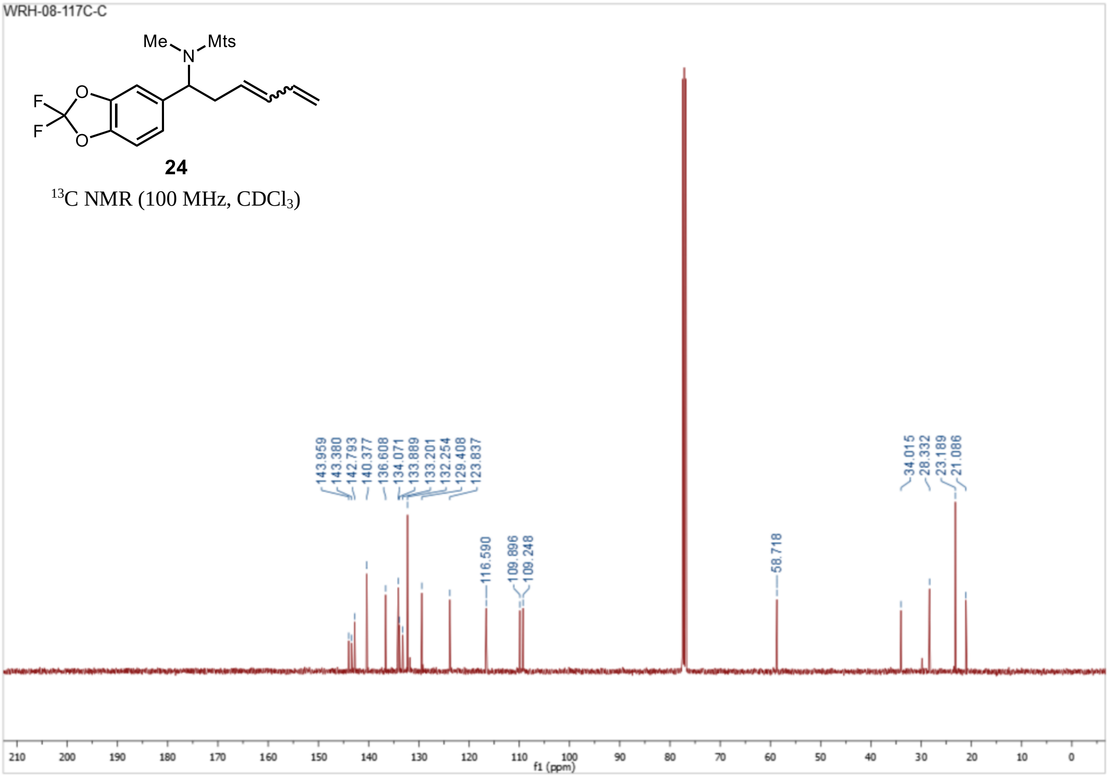
WRH-08-117C-C
Me
N
Mts
F
F
O
O
24
¹³C NMR (100 MHz, CDCl₃)
143.959
143.380
142.793
140.377
136.608
134.071
133.889
133.201
132.254
129.408
123.837
116.590
109.896
109.248
58.718
34.015
28.332
23.189
21.086
f1 (ppm)

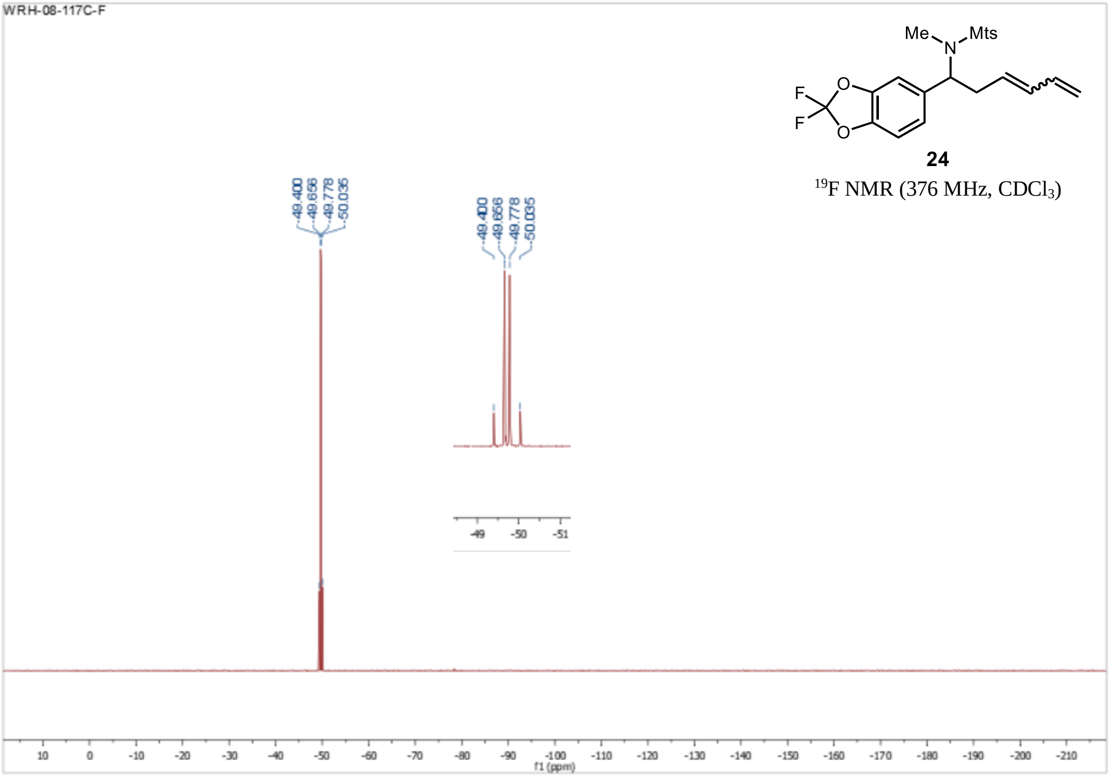

24
^{19}F NMR (376 MHz, $CDCl_3$)

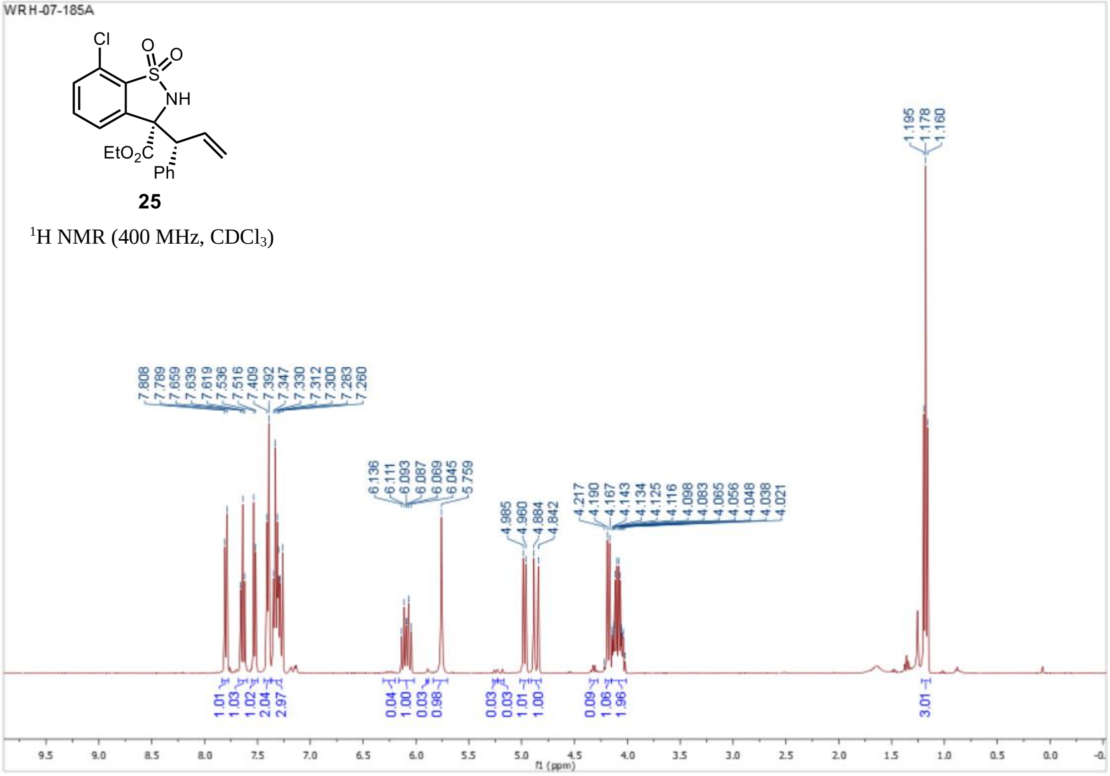
WRH-07-185A
Cl
O
S
O
NH
EtO2C
Ph
25
1H NMR (400 MHz, CDCl3)
f1 (ppm)

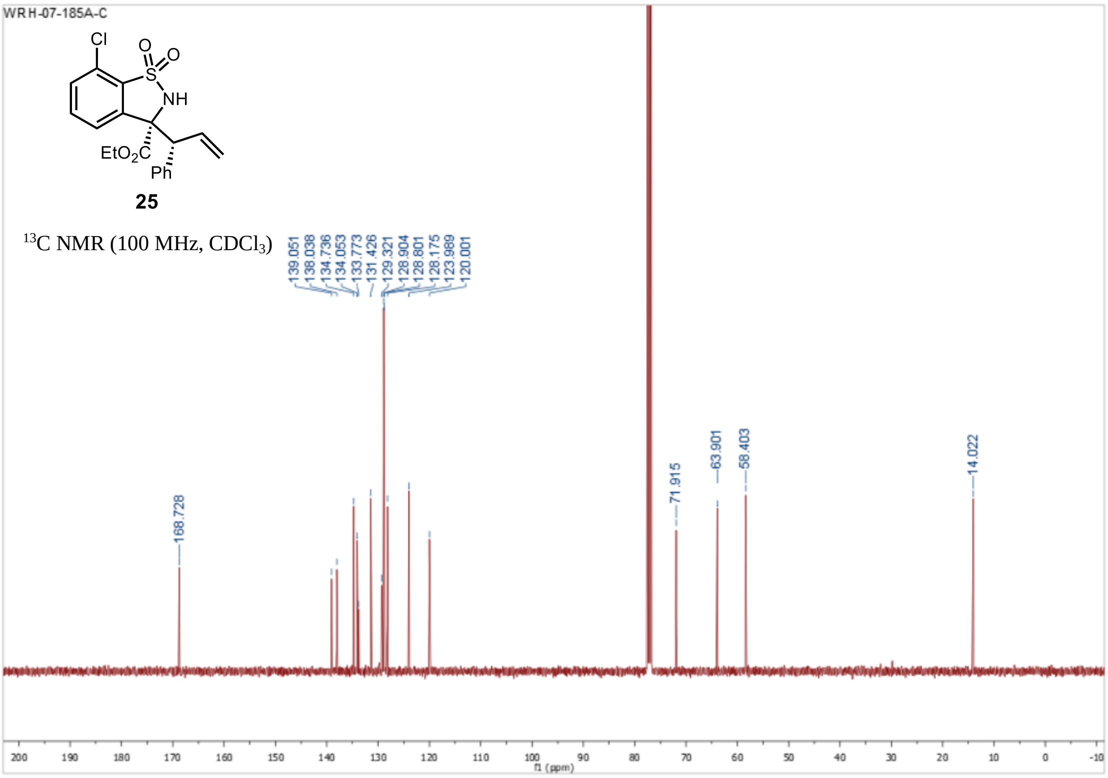
WRH-07-185A-C
Cl
O
S
O
NH
EtO2C
Ph
25
13C NMR (100 MHz, CDCl3)
168.728
71.915
63.901
58.403
14.022
f1 (ppm)

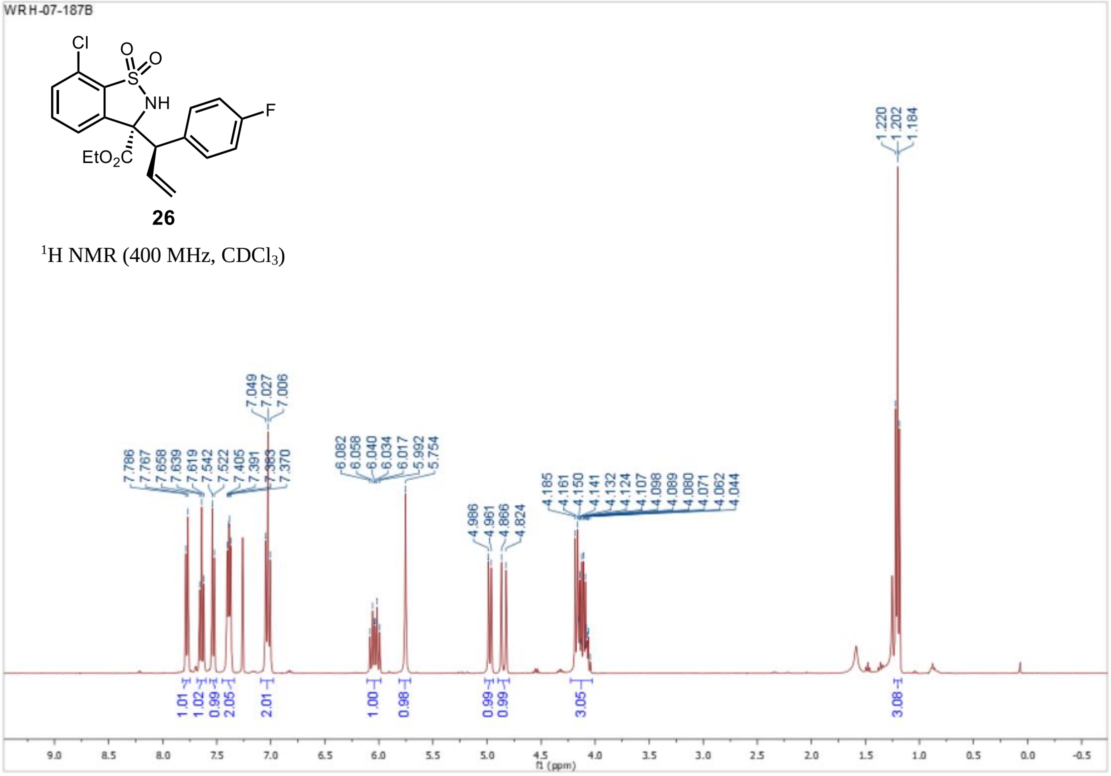
WRH-07-187B
26
^{1}H NMR (400 MHz, CDCl$_3$)
7.786 7.767 7.658 7.639 7.619 7.542 7.522 7.405 7.391 7.383 7.370
7.049 7.027 7.006
6.082 6.058 6.040 6.034 6.017 5.992 5.754
4.986 4.961 4.866 4.824
4.185 4.161 4.150 4.141 4.132 4.124 4.107 4.098 4.089 4.080 4.071 4.062 4.044
1.220 1.202 1.184
1.01 1.02 0.99 2.05 2.01 1.00 0.98 0.99 0.99 3.05 3.08
f1 (ppm)

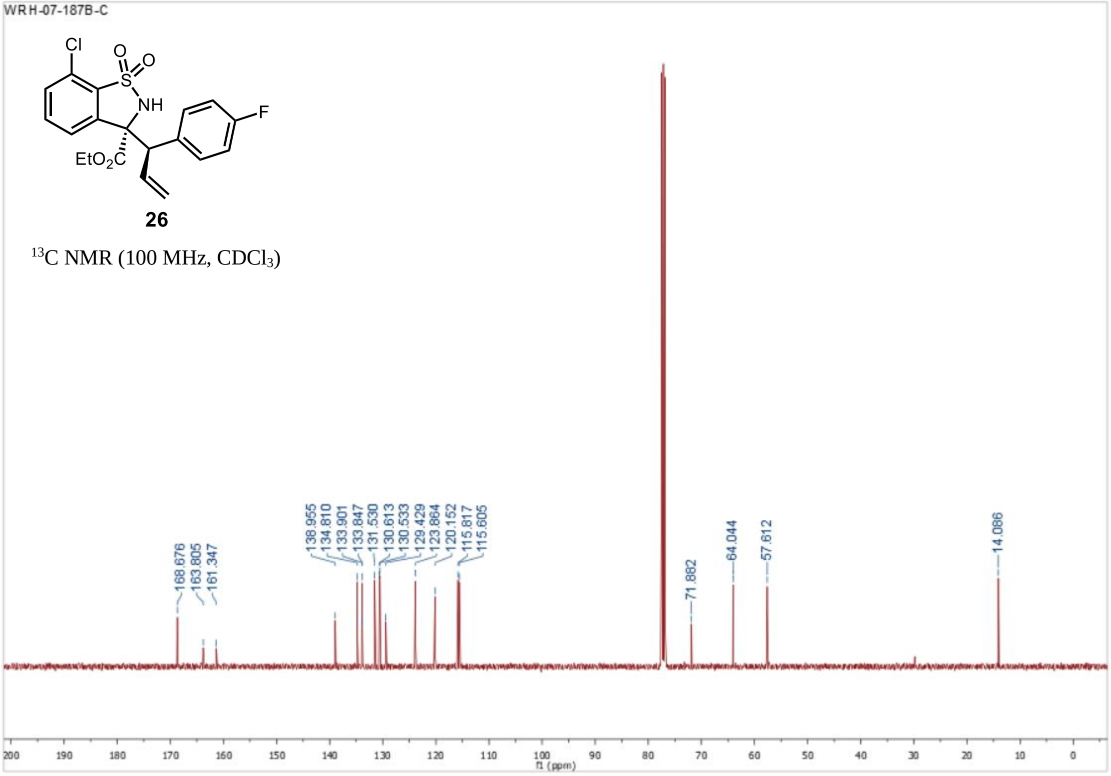
WRH-07-187B-C
26
^{13}C NMR (100 MHz, CDCl$_3$)
168.676 163.805 161.347
138.955 134.810 133.901 133.847 131.530 130.613 130.533 129.429 123.864 120.152 115.817 115.605
71.882 64.044 57.612 14.086
f1 (ppm)

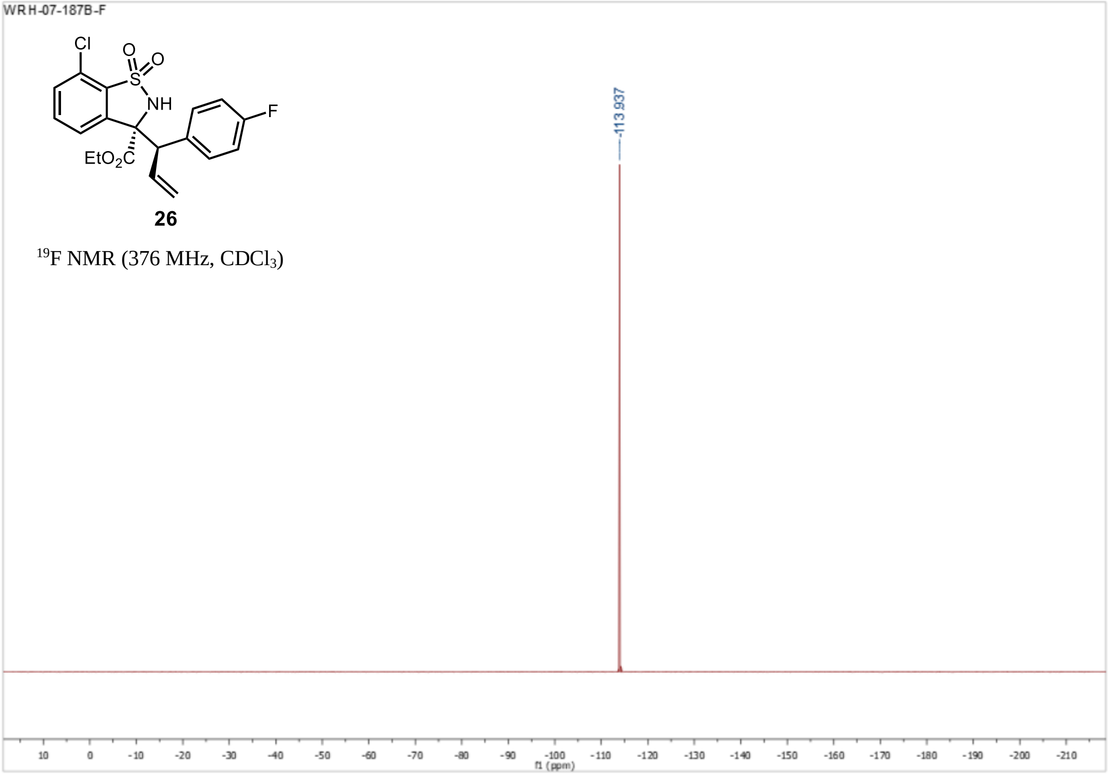
WRH-07-187B-F
Cl
O
S
O
NH
F
EtO2C
26
19F NMR (376 MHz, CDCl3)
-113.937
10 0 -10 -20 -30 -40 -50 -60 -70 -80 -90 -100 -110 -120 -130 -140 -150 -160 -170 -180 -190 -200 -210
f1 (ppm)

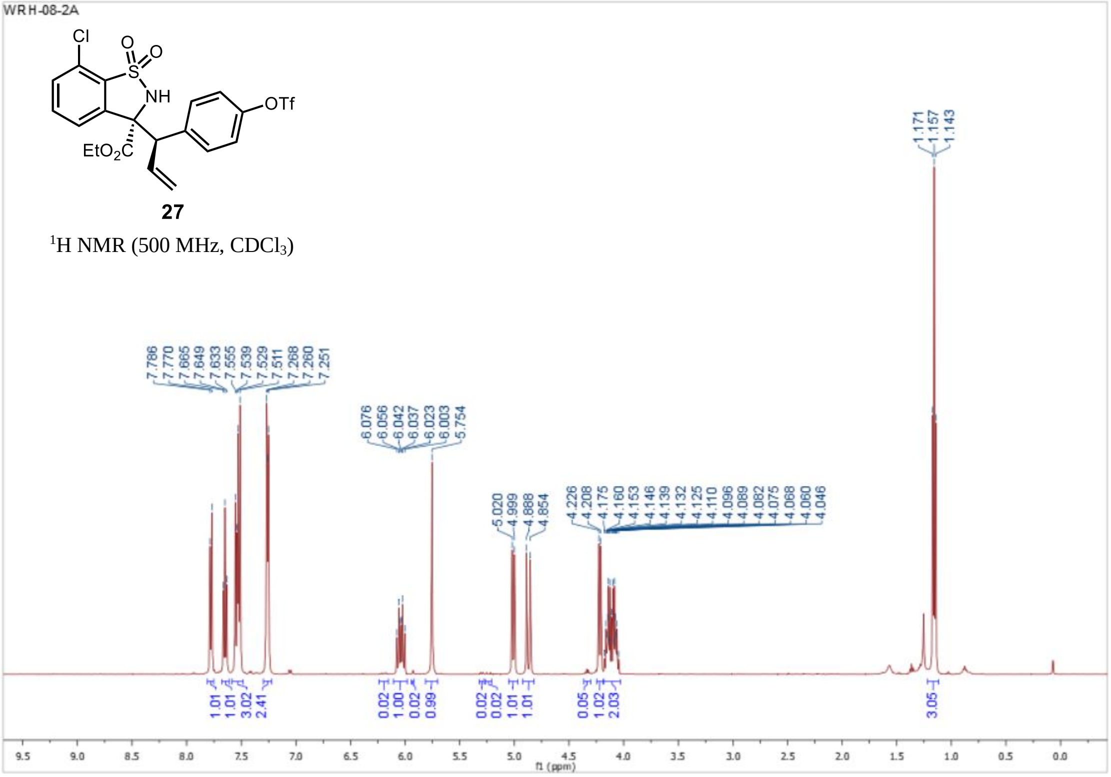
WRH-08-2A
27
¹H NMR (500 MHz, CDCl₃)
f1 (ppm)

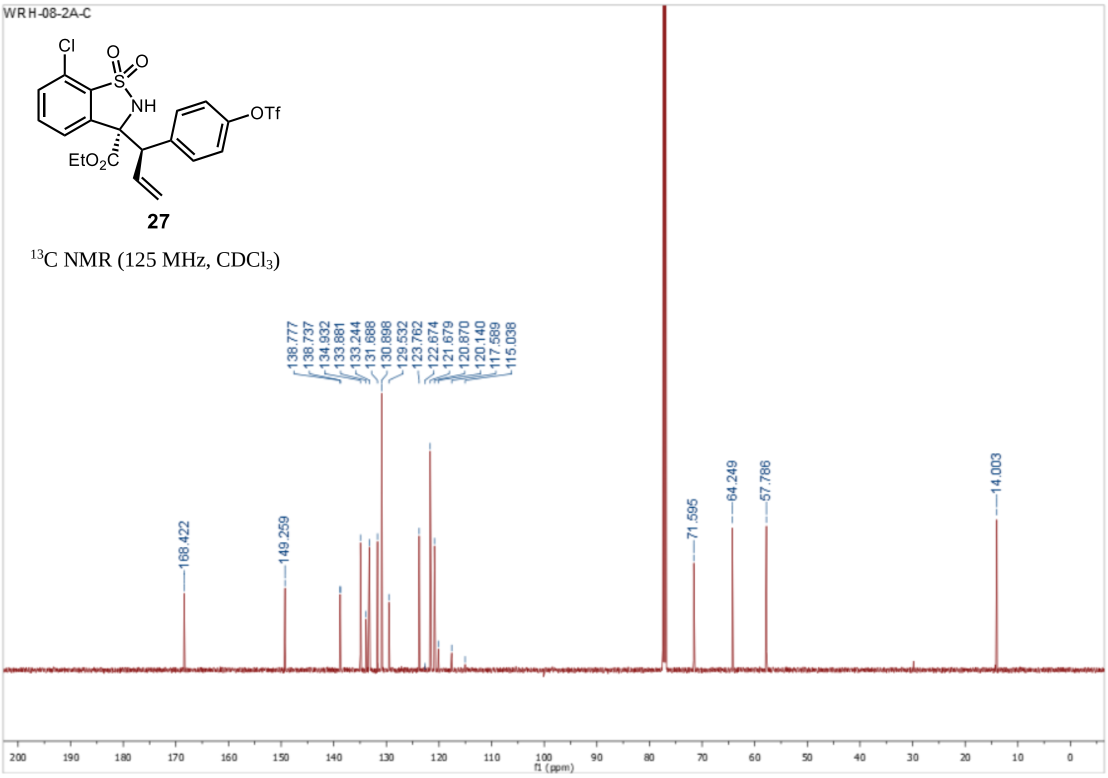
WRH-08-2A-C
27
¹³C NMR (125 MHz, CDCl₃)
f1 (ppm)

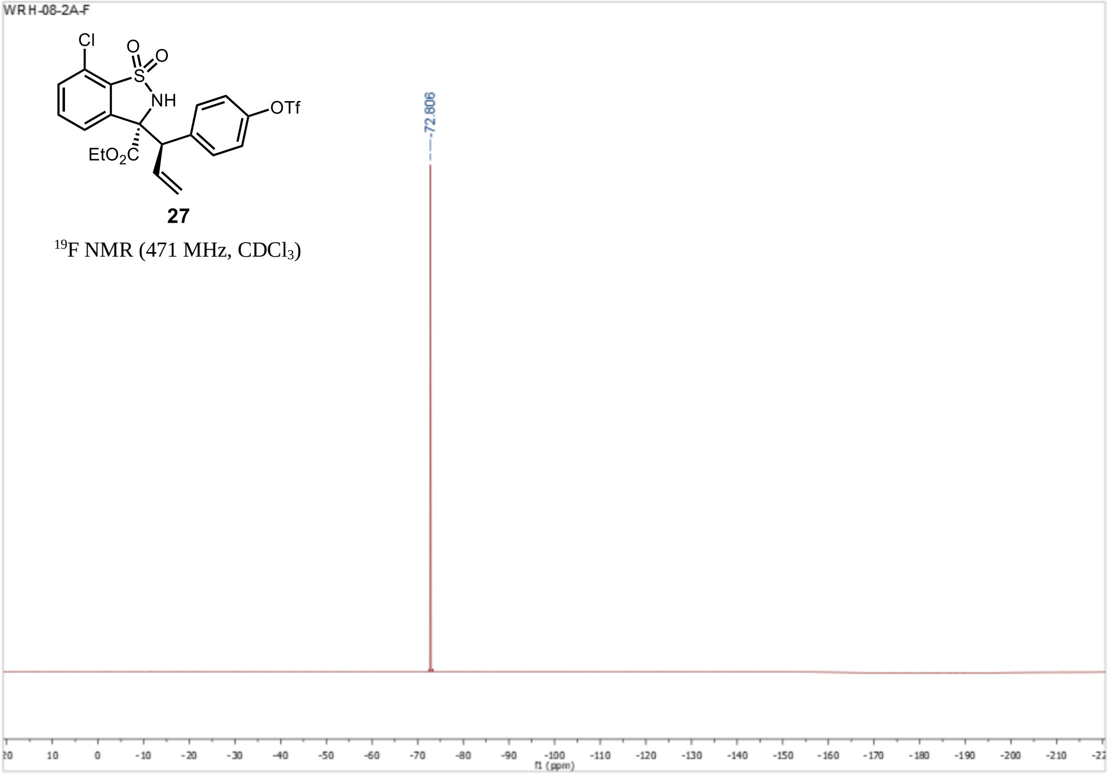

WRH-08-2A-F
Cl
O
S
O
NH
OTf
EtO2C
27
19F NMR (471 MHz, CDCl3)
-72.806
f1 (ppm)

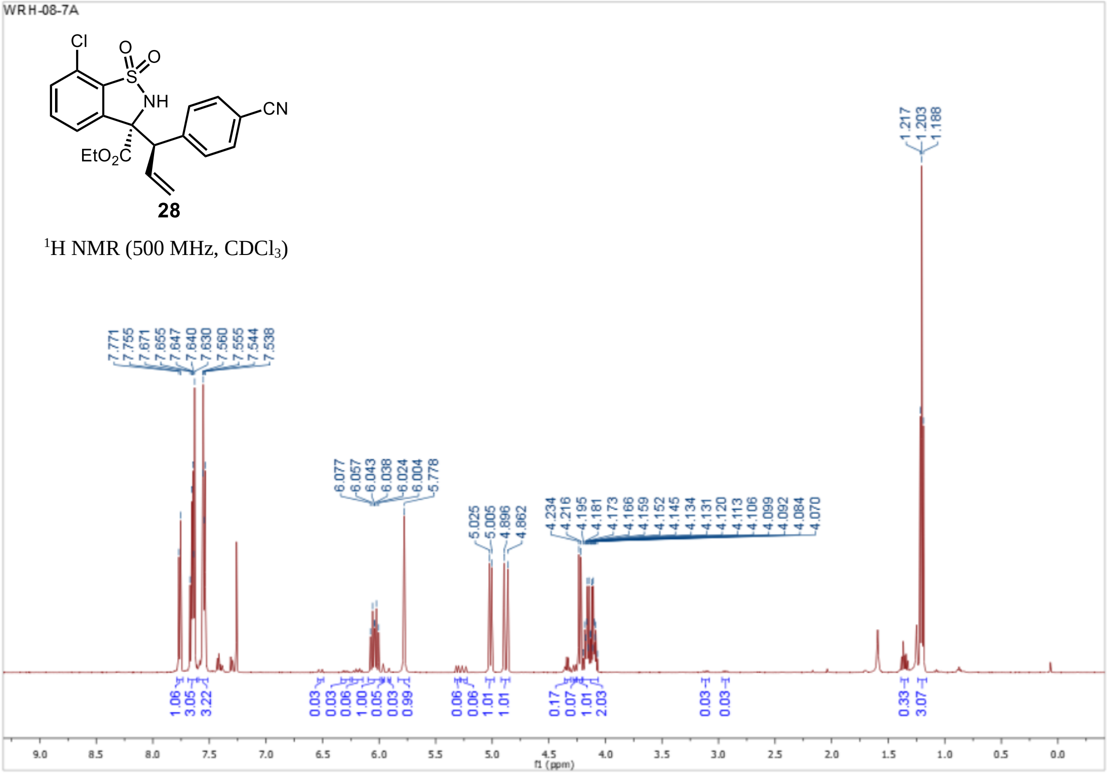

28

^{1}H NMR (500 MHz, $CDCl_3$)

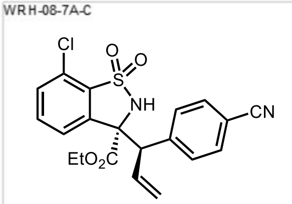

28

^{13}C NMR (125 MHz, $CDCl_3$)

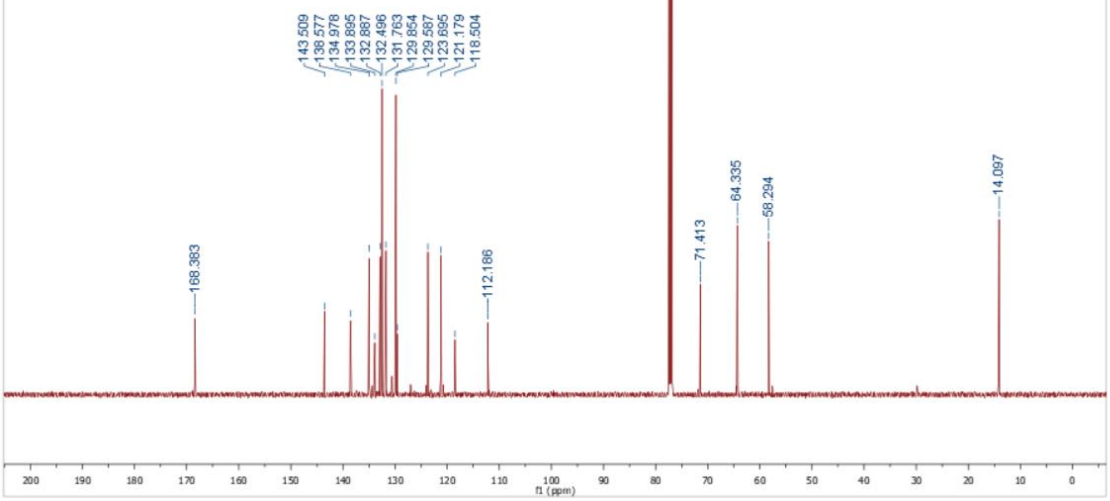

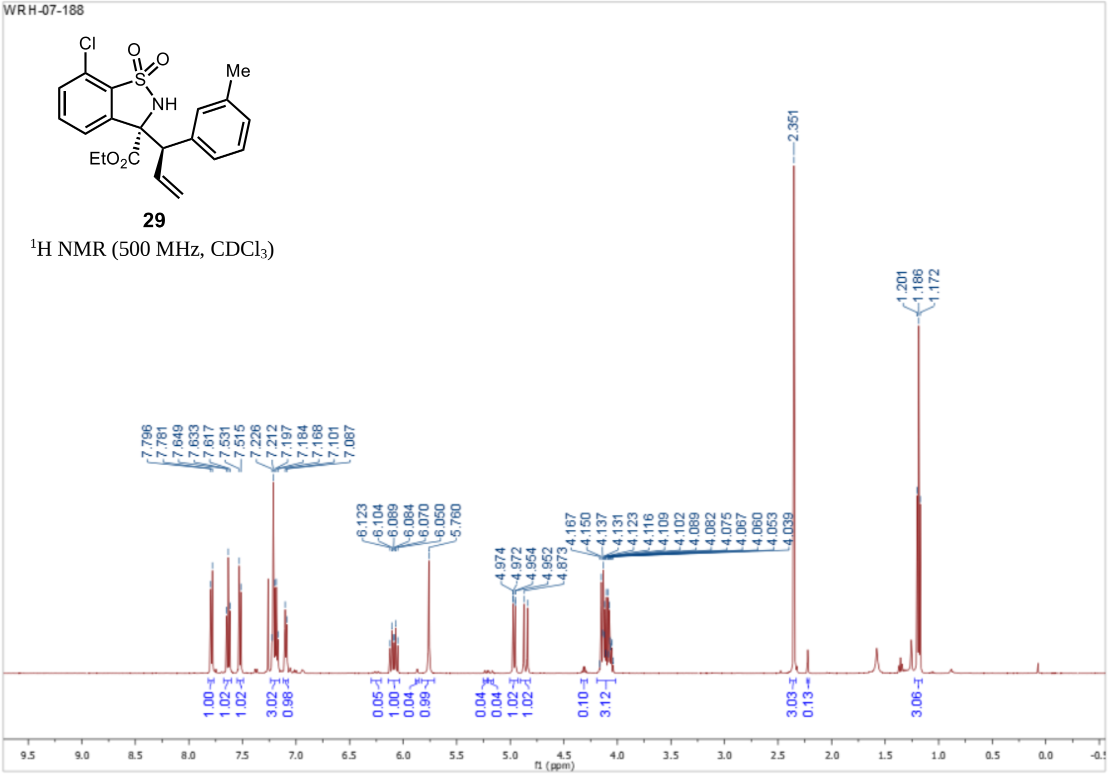
WRH-07-188
Cl
O
O
S
NH
Me
EtO2C
29
1H NMR (500 MHz, CDCl3)
7.796 7.781 7.649 7.633 7.617 7.531 7.515 7.226 7.212 7.197 7.184 7.168 7.101 7.087
6.123 6.104 6.089 6.084 6.070 6.050 5.760
4.974 4.972 4.954 4.952 4.873
4.167 4.150 4.137 4.131 4.123 4.116 4.109 4.102 4.089 4.082 4.075 4.067 4.060 4.053 4.039
2.351
1.201 1.186 1.172
1.00 1.02 1.02 3.02 0.98 0.05 1.00 0.04 0.99 0.04 0.04 1.02 1.02 0.10 3.12 3.03 0.13 3.06
f1 (ppm)

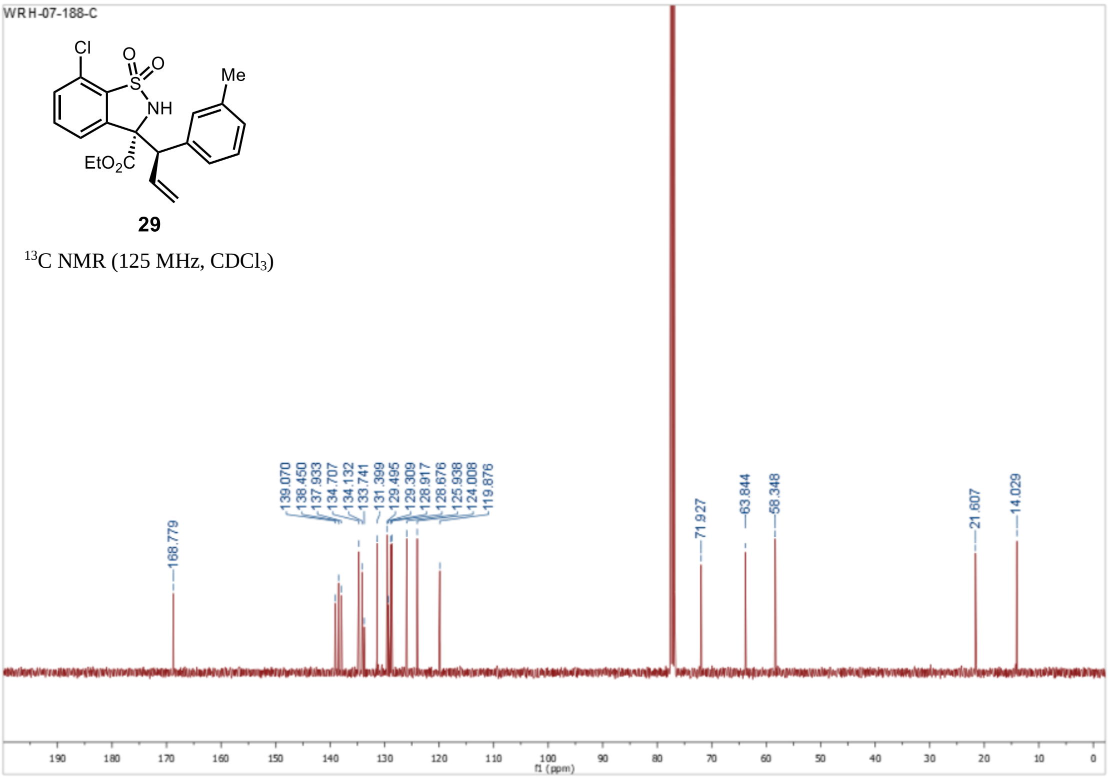
WRH-07-188-C
Cl
O
O
S
NH
Me
EtO2C
29
13C NMR (125 MHz, CDCl3)
168.779
139.070 138.450 137.933 134.707 134.132 133.741 131.399 129.495 129.309 128.917 128.676 125.938 124.008 119.876
71.927
63.844
58.348
21.607
14.029
f1 (ppm)

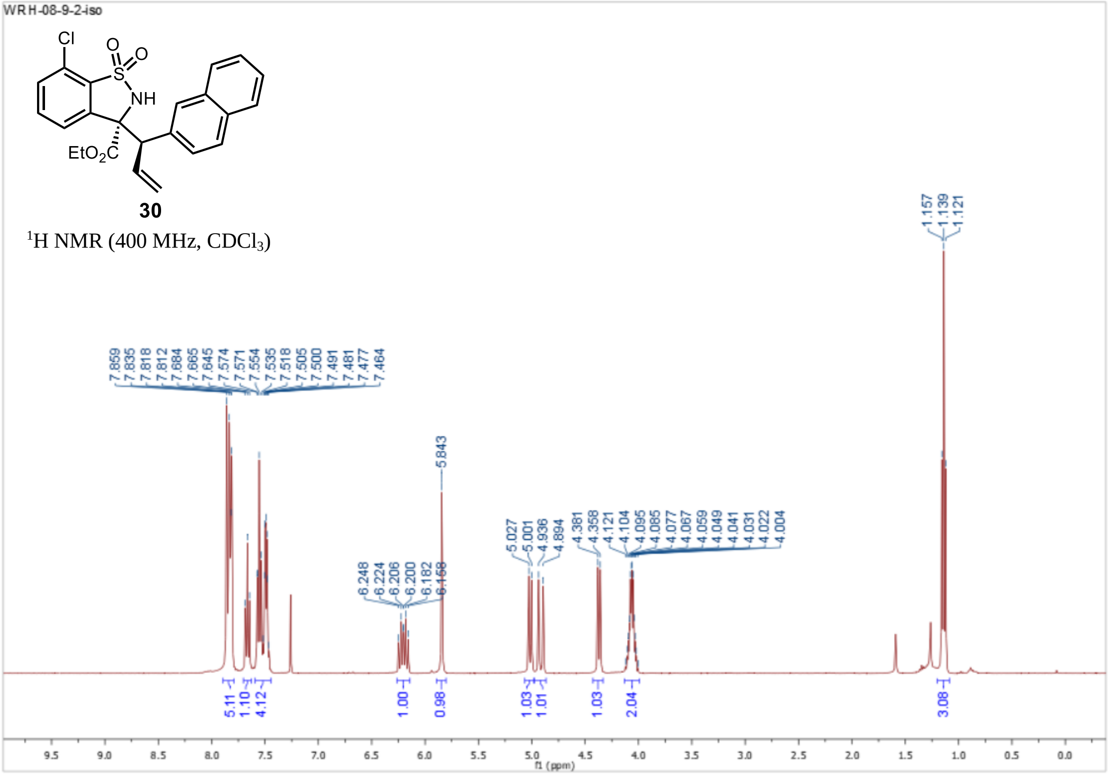

^{1}H NMR (400 MHz, $CDCl_3$)

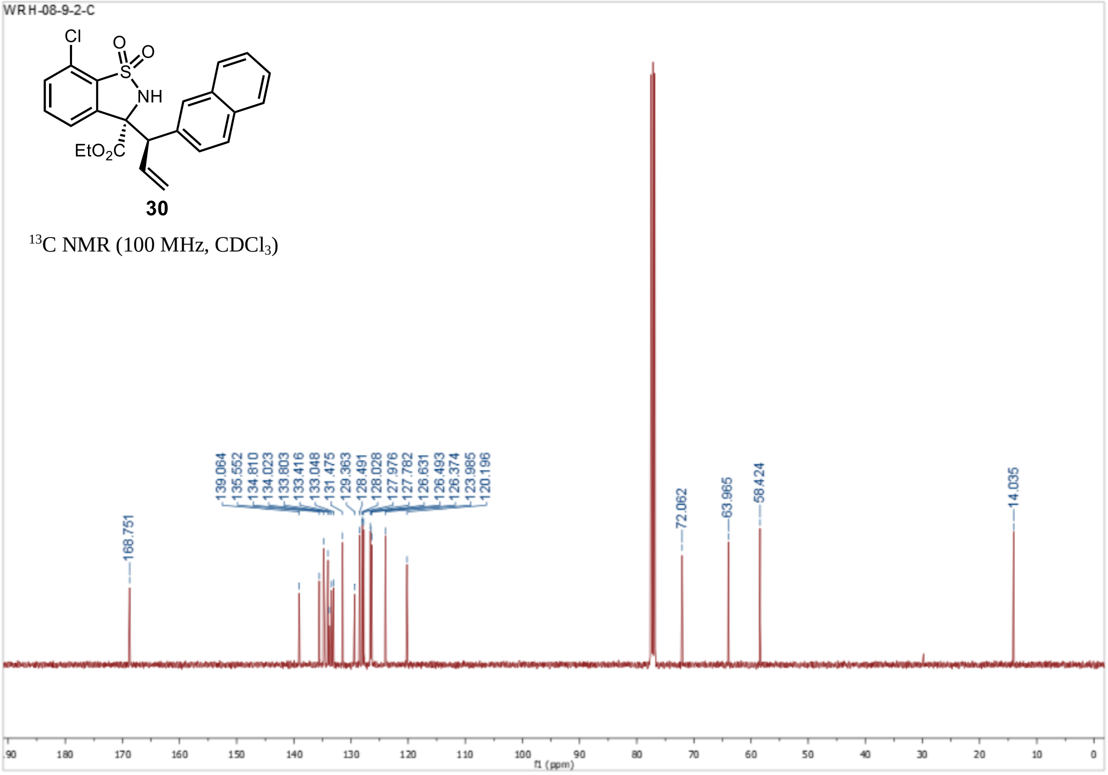

^{13}C NMR (100 MHz, $CDCl_3$)

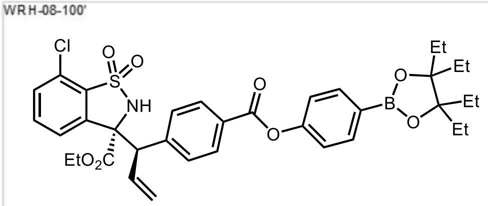

31

^{1}H NMR (500 MHz, $CDCl_3$)

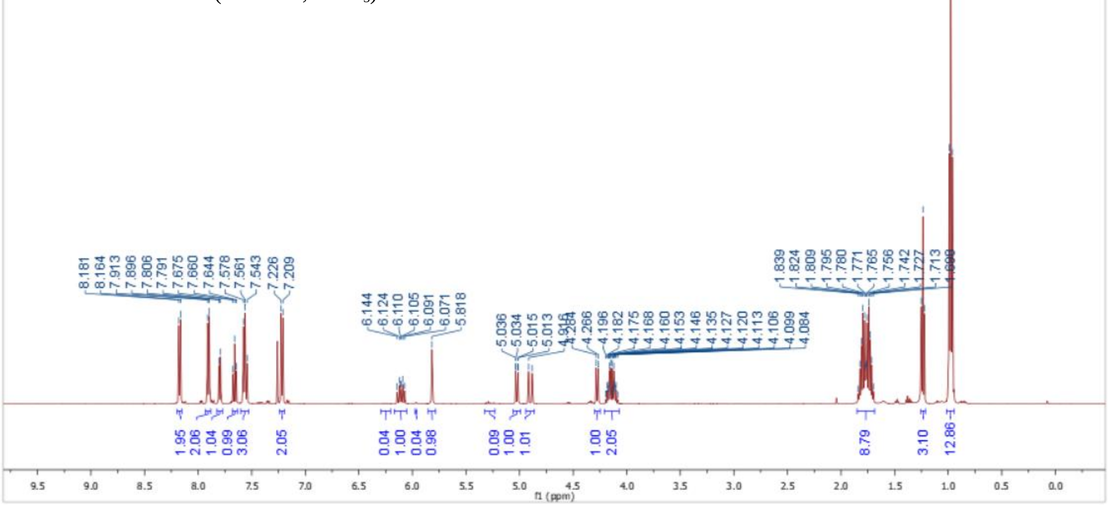

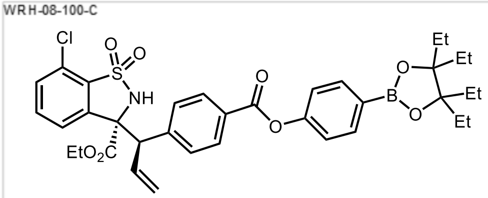

31

^{13}C NMR (125 MHz, $CDCl_3$)

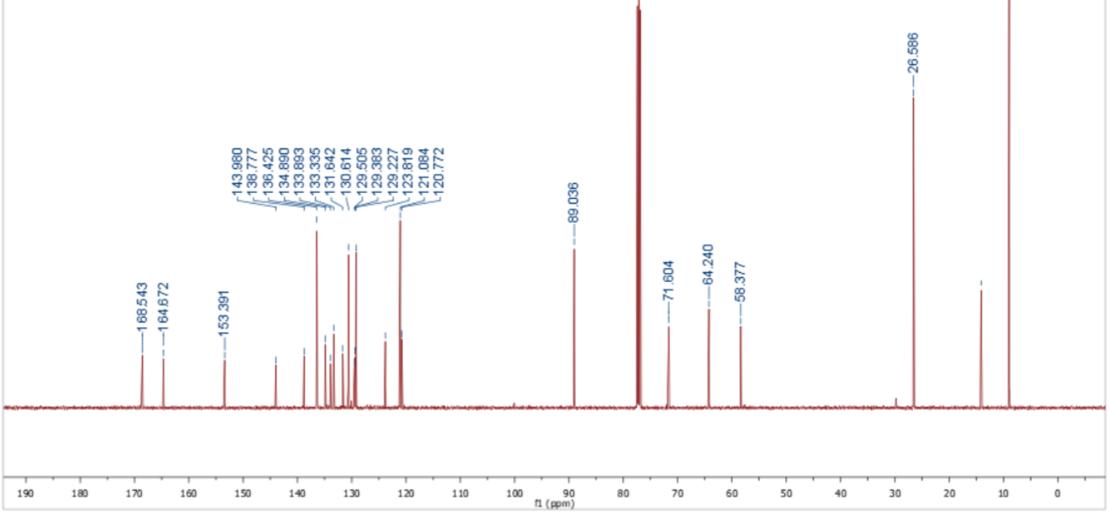

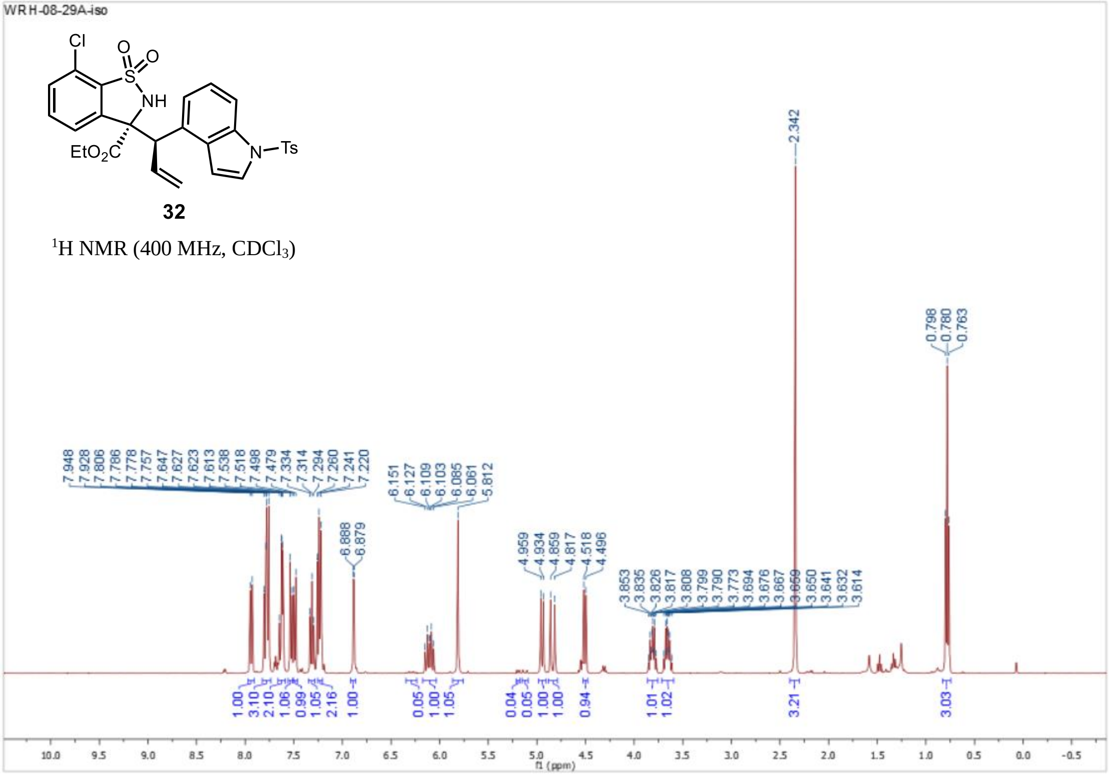

32

^{1}H NMR (400 MHz, $CDCl_3$)

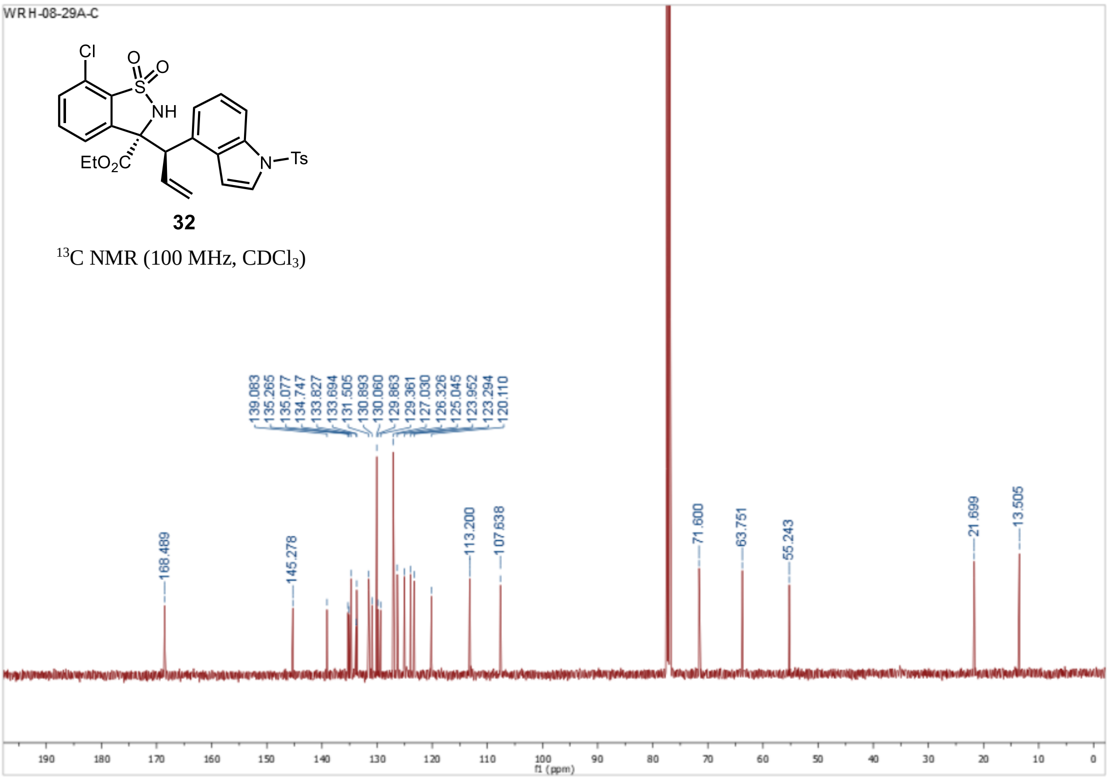

32

^{13}C NMR (100 MHz, $CDCl_3$)

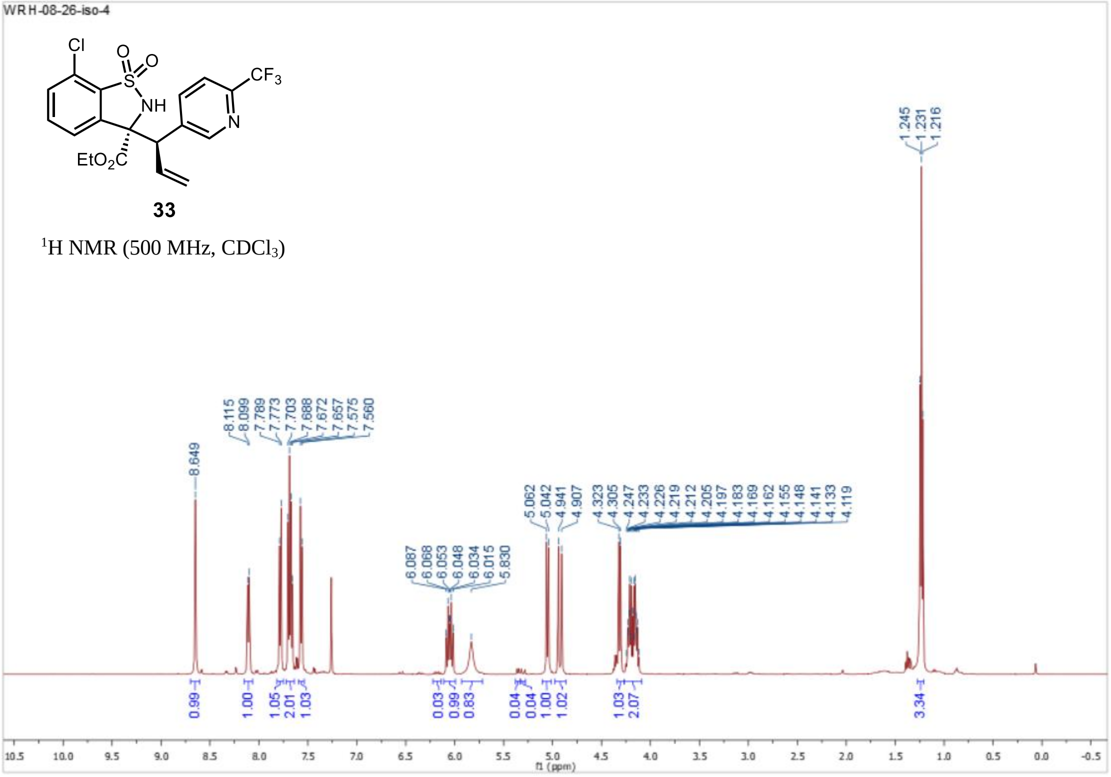
WRH-08-26-iso-4
33
^{1}H NMR (500 MHz, CDCl3)
f1 (ppm)

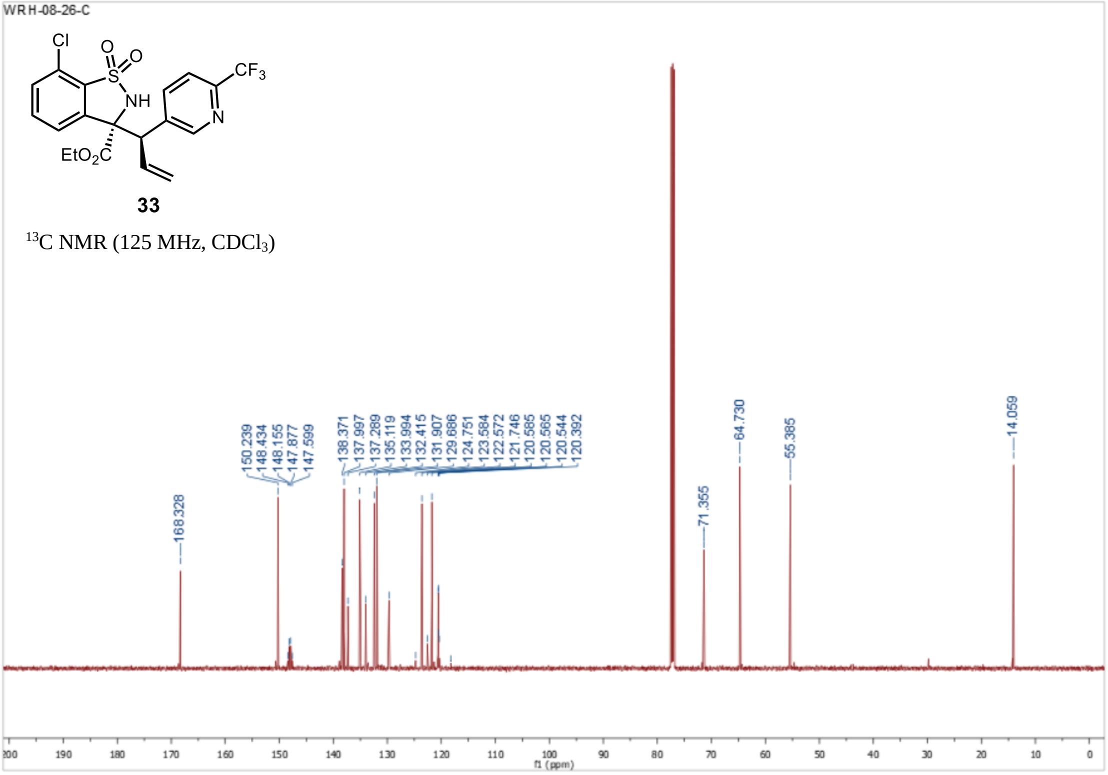
WRH-08-26-C
33
^{13}C NMR (125 MHz, CDCl3)
f1 (ppm)

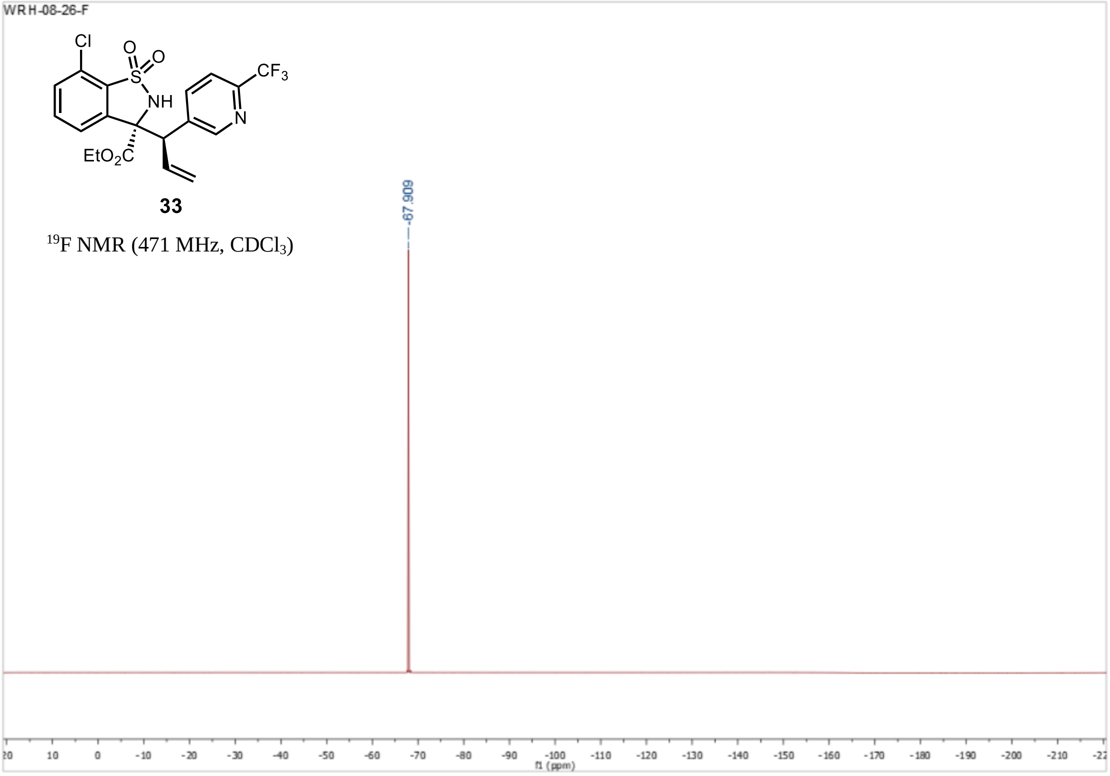
WRH-08-26-F
Cl
O
O
S
NH
CF3
N
EtO2C
33
19F NMR (471 MHz, CDCl3)
-67.909
20 10 0 -10 -20 -30 -40 -50 -60 -70 -80 -90 -100 -110 -120 -130 -140 -150 -160 -170 -180 -190 -200 -210
f1 (ppm)

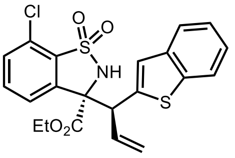

34

^{1}H NMR (400 MHz, $CDCl_3$)

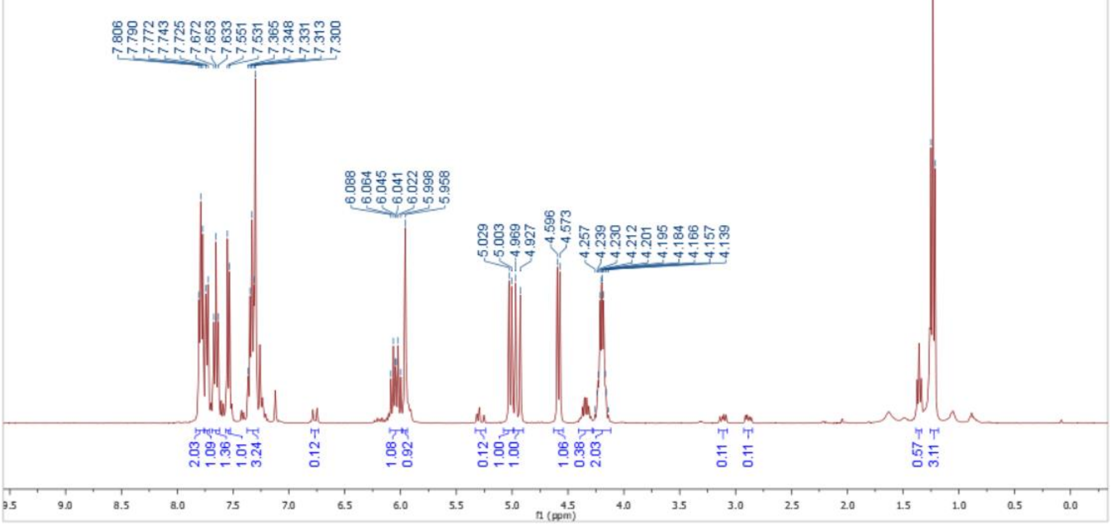

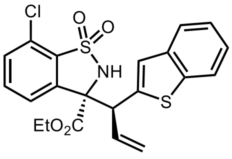

34

^{13}C NMR (100 MHz, $CDCl_3$)

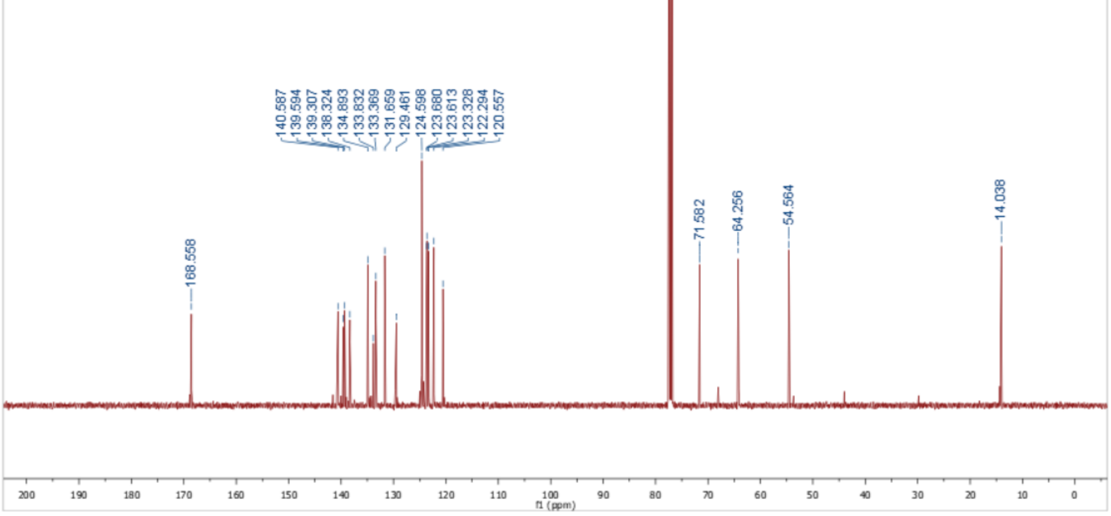

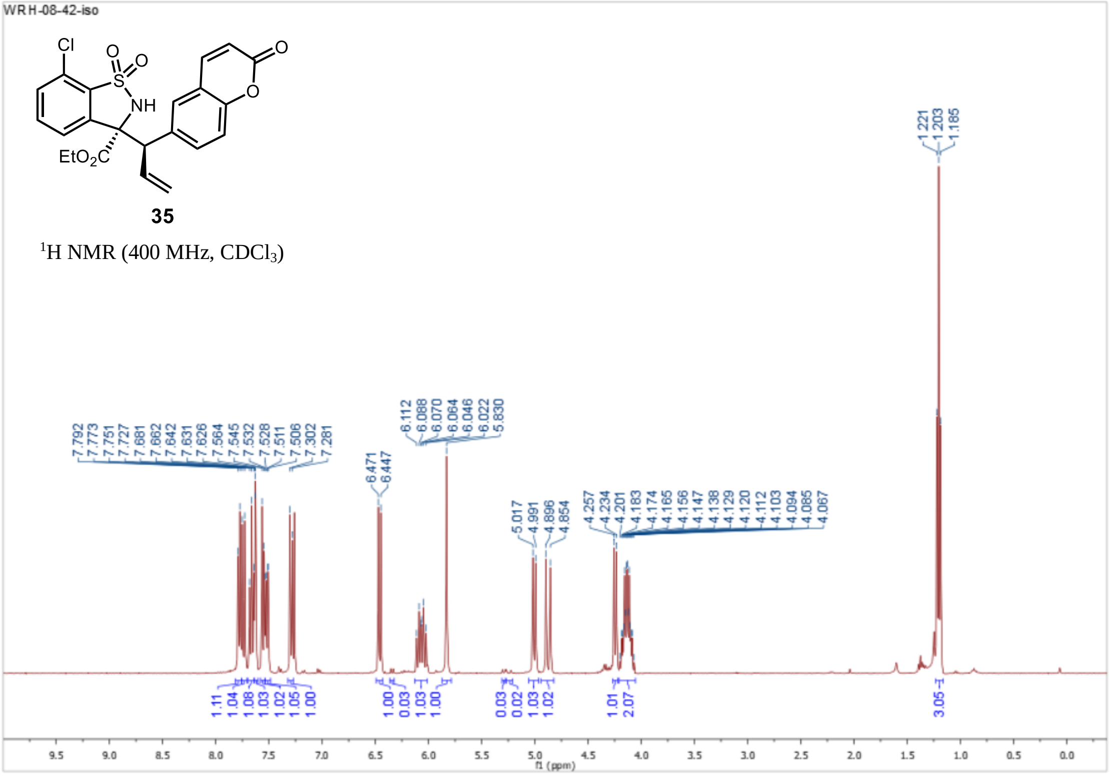
WRH-08-42-iso
35
^{1}H NMR (400 MHz, CDCl$_3$)

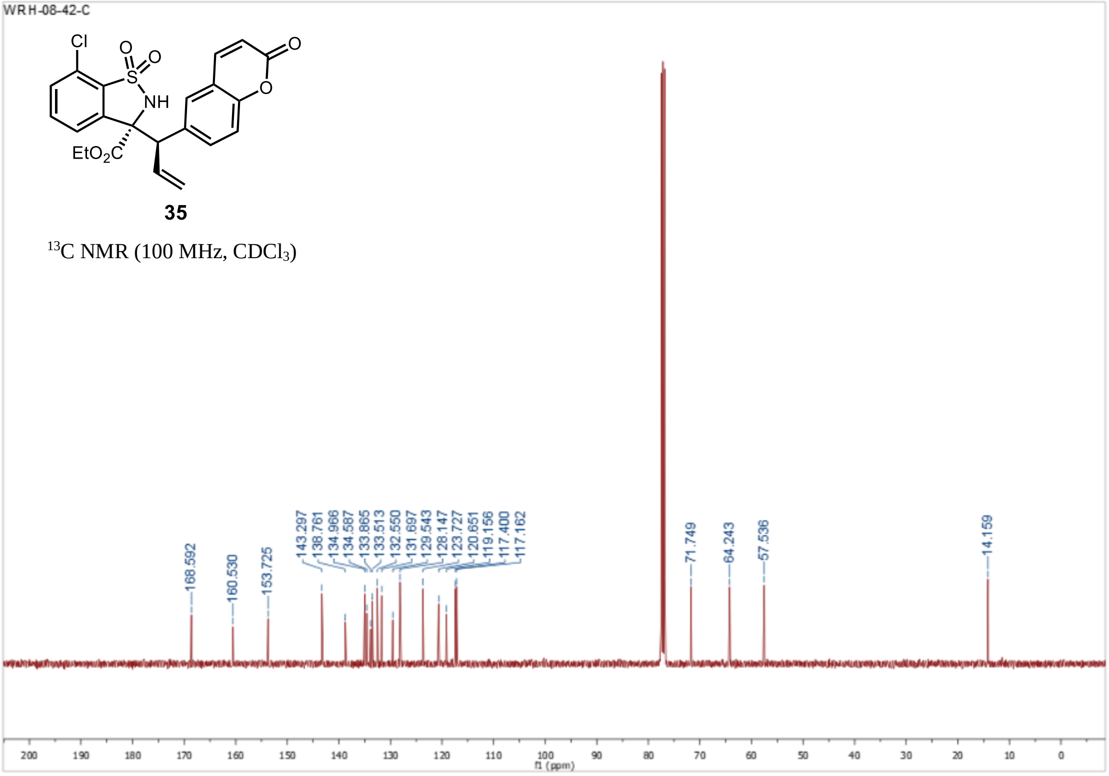
WRH-08-42-C
35
^{13}C NMR (100 MHz, CDCl$_3$)
168.592
160.530
153.725
143.297
138.761
134.966
134.587
133.865
133.513
132.550
131.697
129.543
128.147
123.727
120.651
119.156
117.400
117.162
71.749
64.243
57.536
14.159
f1 (ppm)

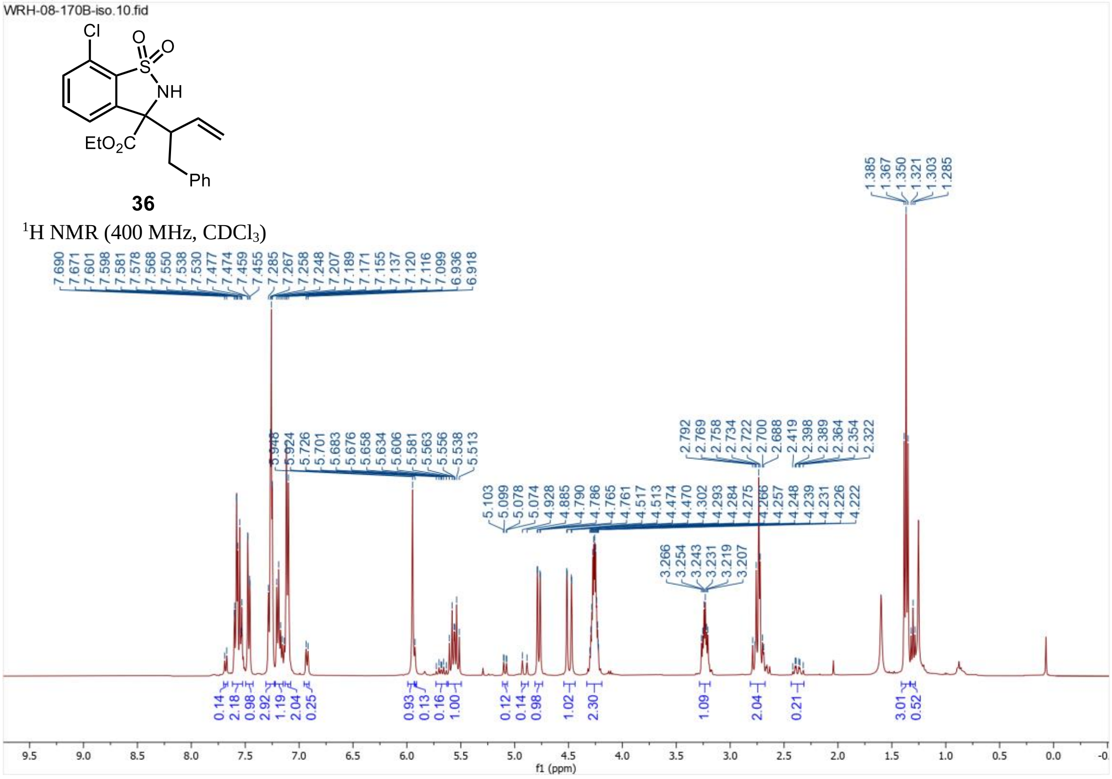

36

^{1}H NMR (400 MHz, $CDCl_3$)

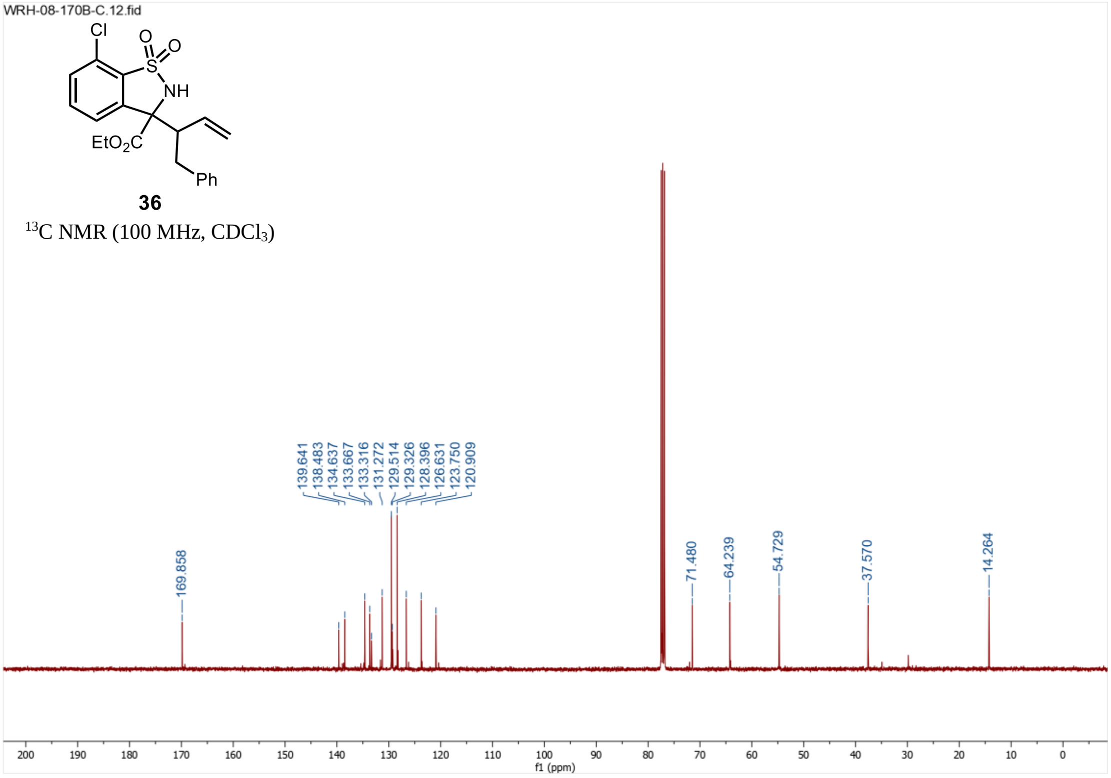

36

^{13}C NMR (100 MHz, $CDCl_3$)

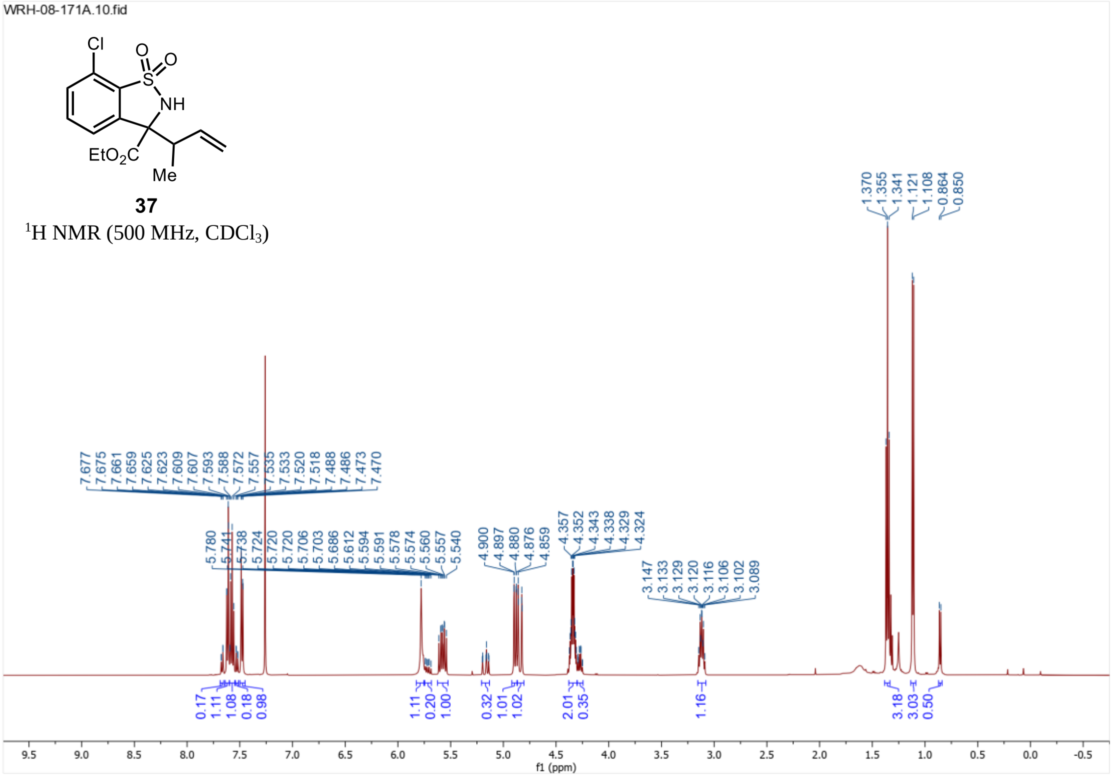

37

^{1}H NMR (500 MHz, $CDCl_3$)

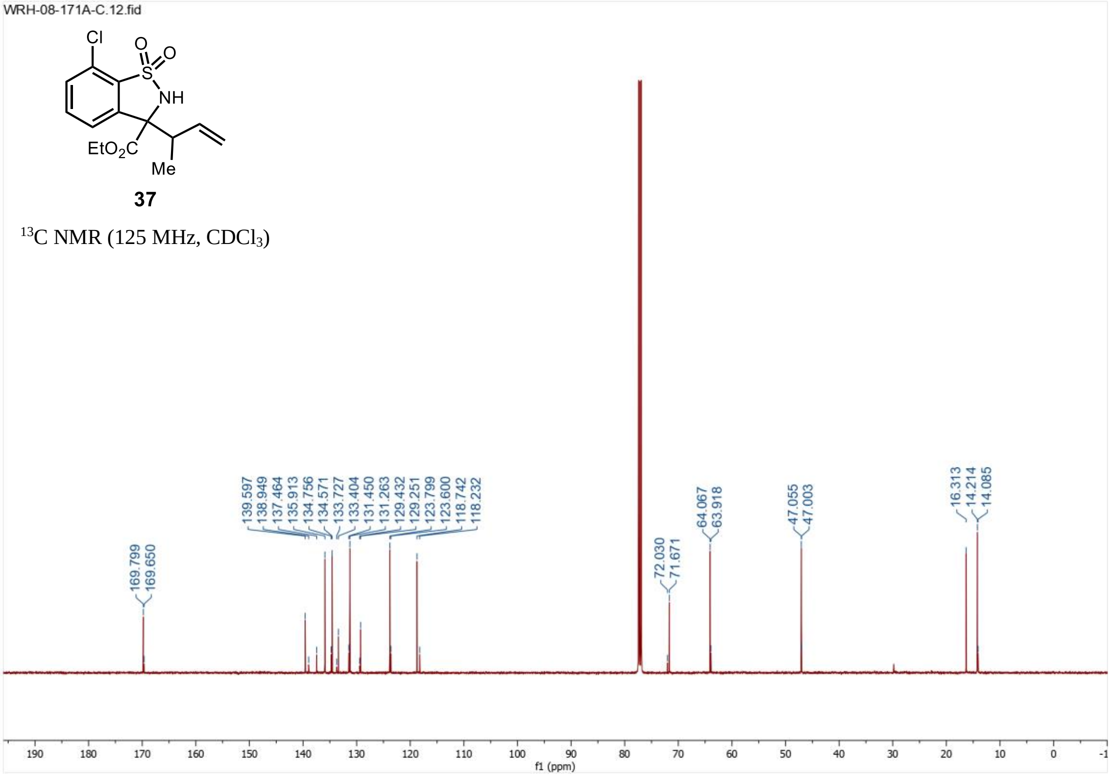

37

^{13}C NMR (125 MHz, $CDCl_3$)

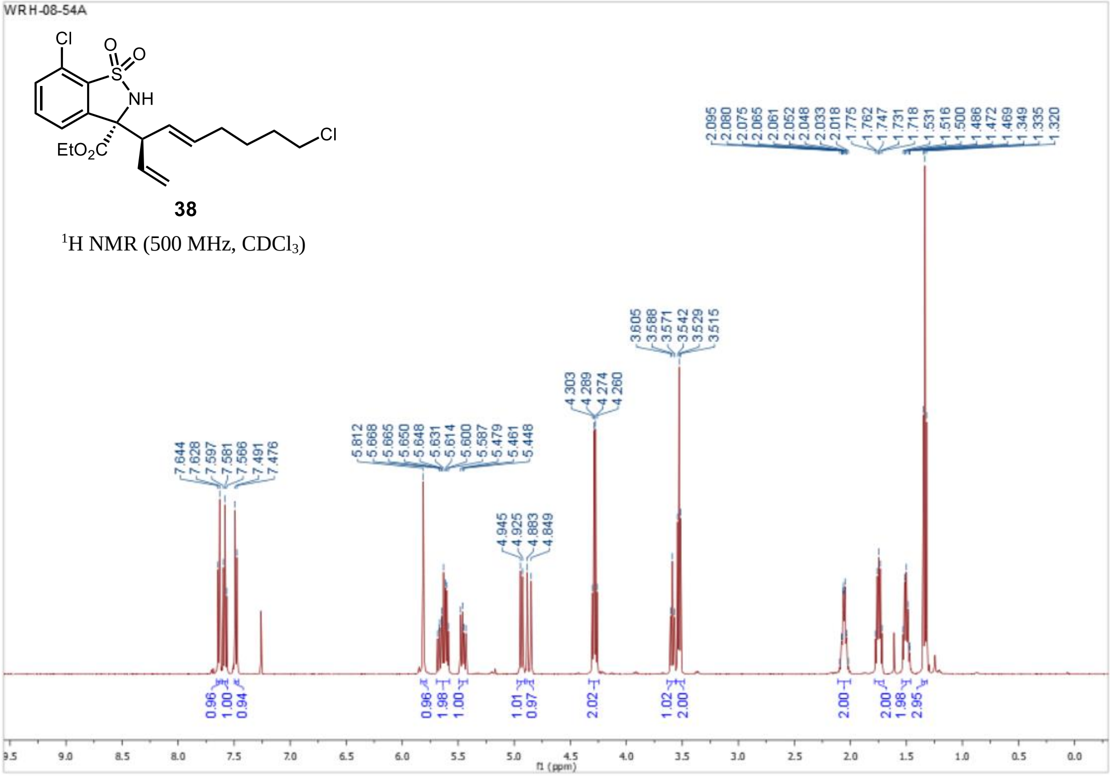
WRH-08-54A
Cl
O
S
O
NH
EtO2C
Cl
38
1H NMR (500 MHz, CDCl3)
2.095 2.080 2.075 2.065 2.061 2.052 2.048 2.033 2.018 1.775 1.762 1.747 1.731 1.718 1.531 1.516 1.500 1.486 1.472 1.469 1.349 1.335 1.320
3.605 3.588 3.571 3.542 3.529 3.515
4.303 4.289 4.274 4.260
5.812 5.668 5.665 5.650 5.648 5.631 5.614 5.600 5.587 5.479 5.461 5.448
7.644 7.628 7.597 7.581 7.566 7.491 7.476
4.945 4.925 4.883 4.849
0.96 1.00 0.94 0.96 1.98 1.00 1.01 0.97 2.02 1.02 2.00 2.00 2.00 1.98 2.95
f1 (ppm)

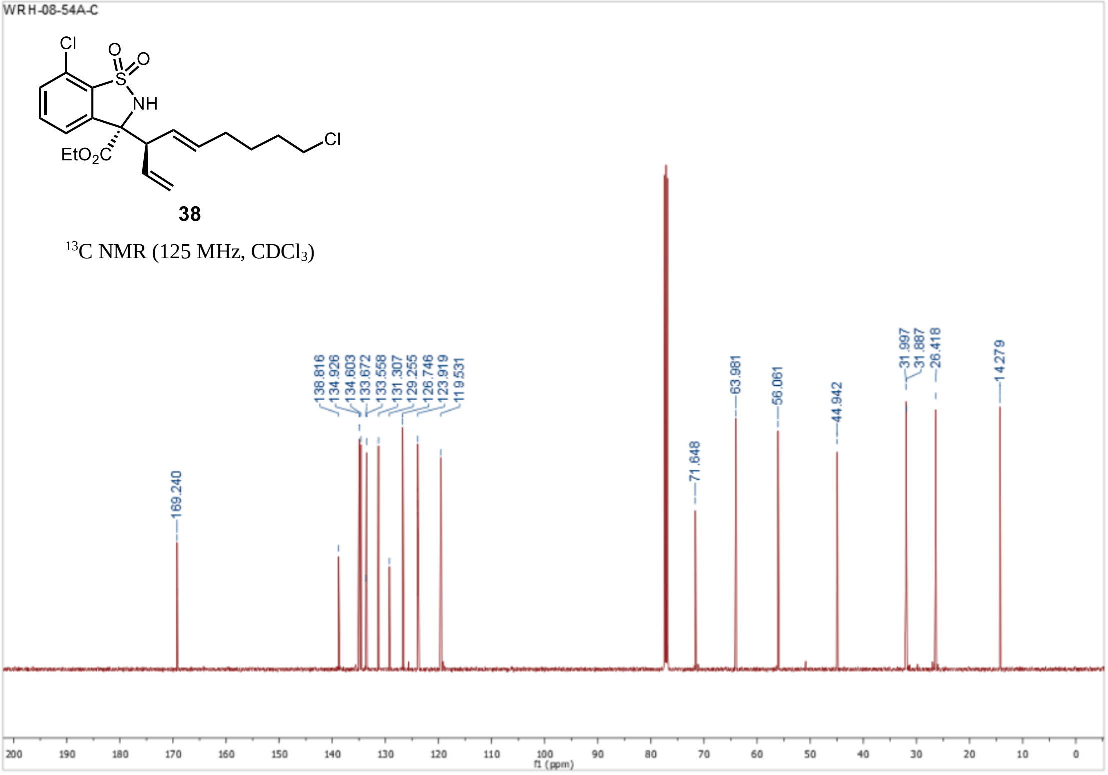
WRH-08-54A-C
Cl
O
S
O
NH
EtO2C
Cl
38
13C NMR (125 MHz, CDCl3)
169.240
138.816 134.926 134.603 133.672 133.558 131.307 129.255 126.746 123.919 119.531
71.648
63.981
56.061
44.942
31.997 31.887 26.418
14.279
f1 (ppm)

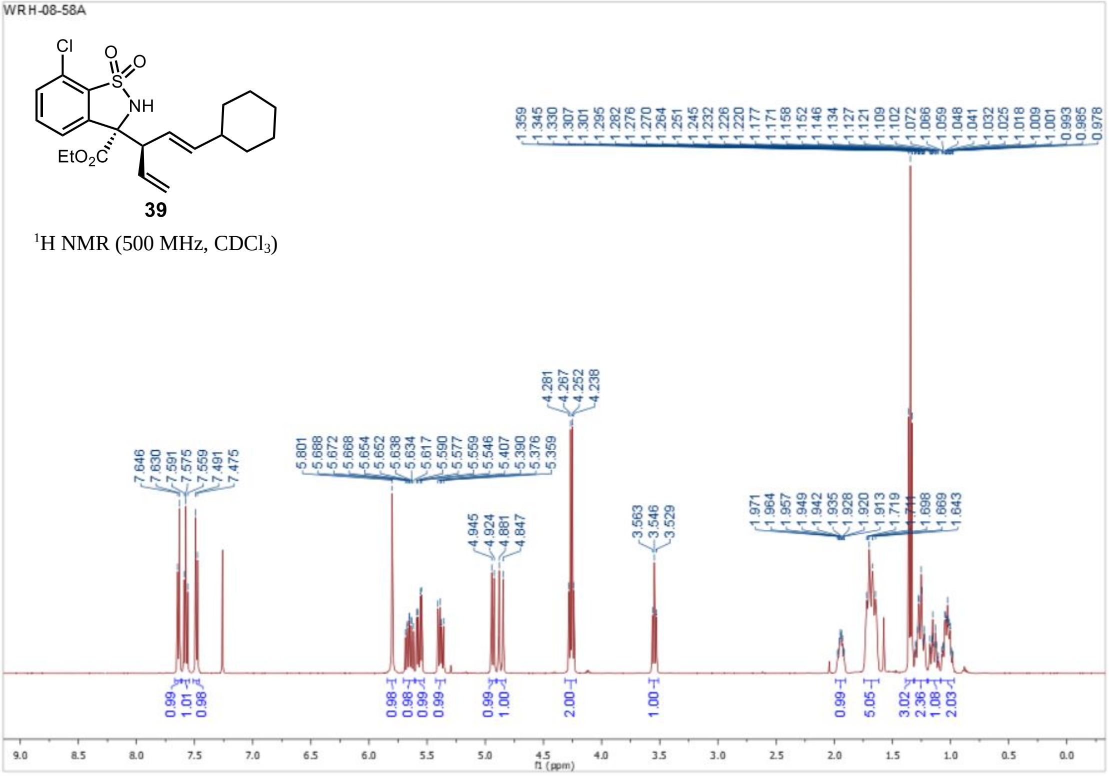
WRH-08-58A
39
^{1}H NMR (500 MHz, CDCl$_3$)
f1 (ppm)

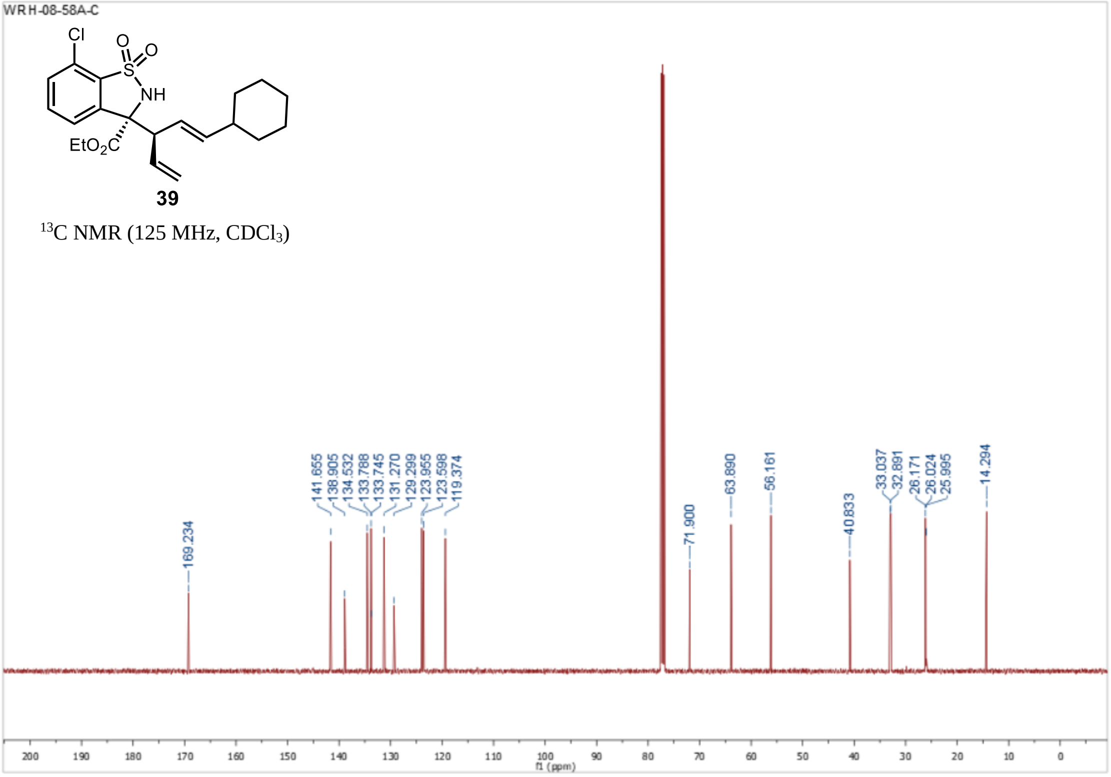
WRH-08-58A-C
39
^{13}C NMR (125 MHz, CDCl$_3$)
169.234
141.655
138.905
134.532
133.788
133.745
131.270
129.299
123.955
123.598
119.374
71.900
63.890
56.161
40.833
33.037
32.891
26.171
26.024
25.995
14.294
f1 (ppm)

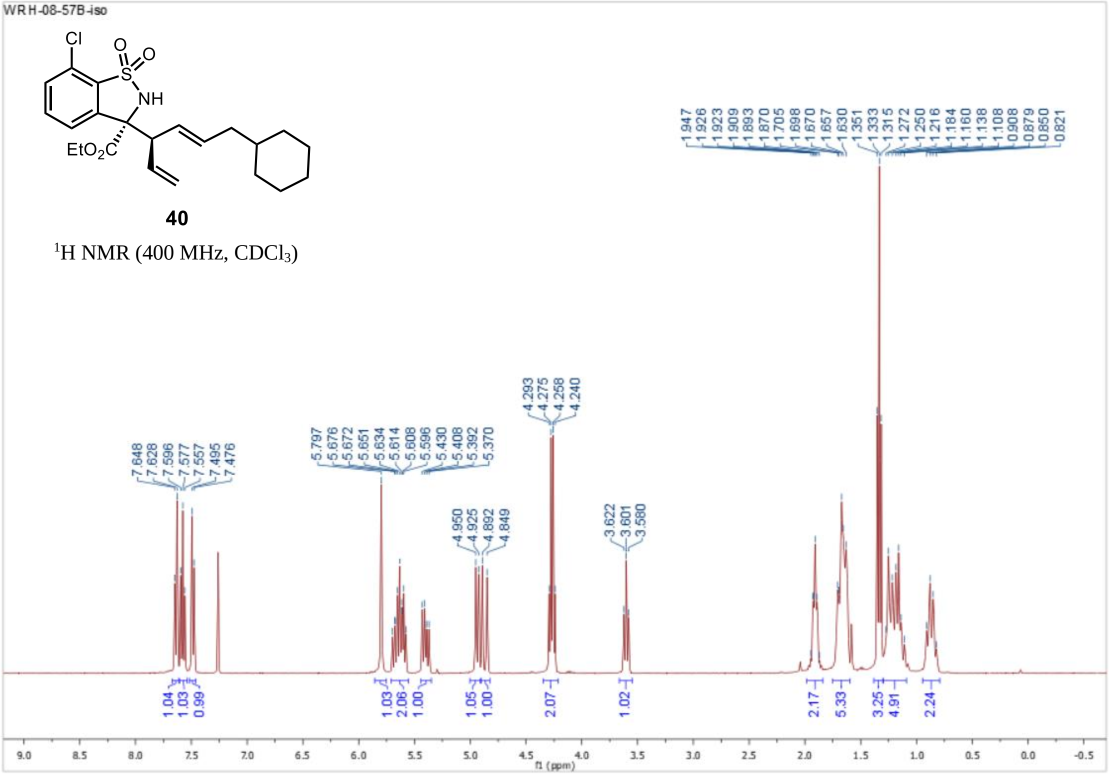

40

^{1}H NMR (400 MHz, $CDCl_3$)

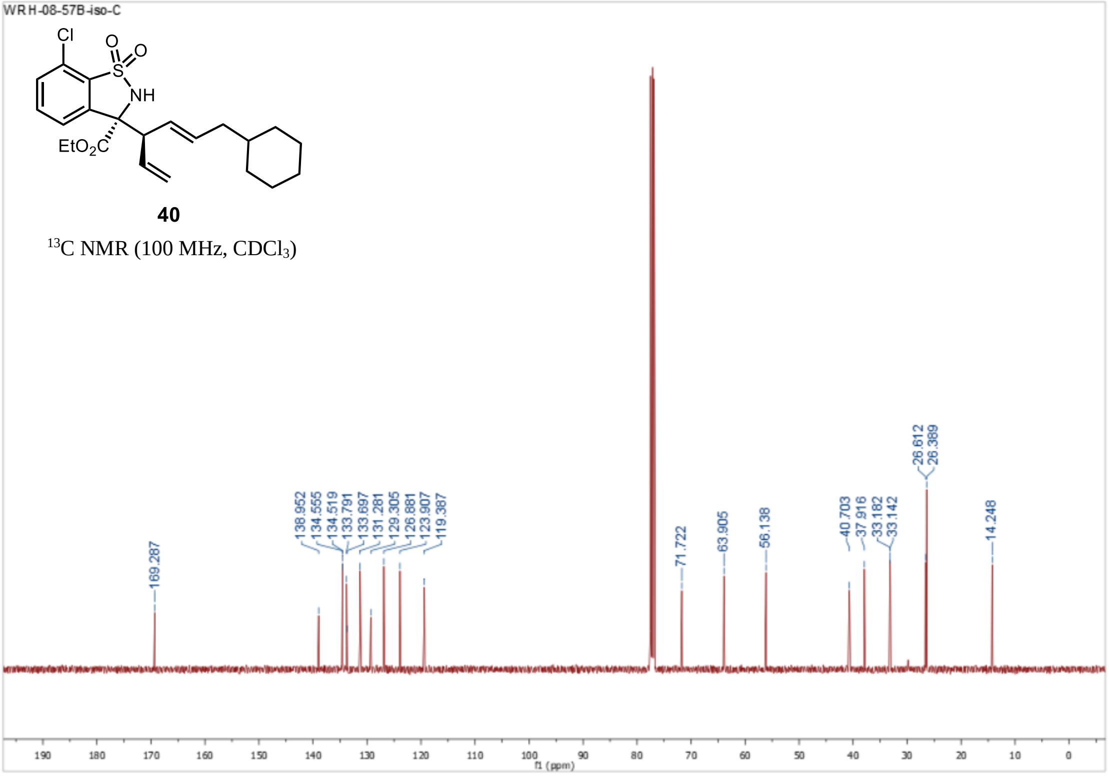

40

^{13}C NMR (100 MHz, $CDCl_3$)

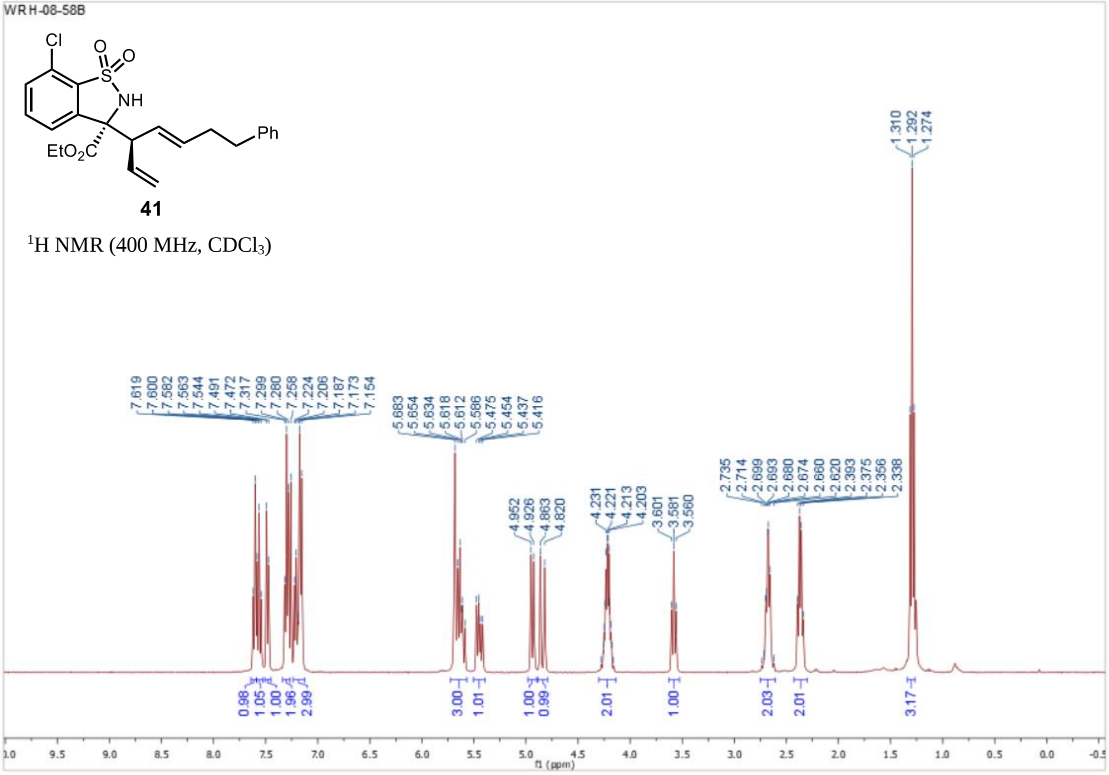
WRH-08-58B
Cl
O
S
O
NH
EtO2C
Ph
41
1H NMR (400 MHz, CDCl3)
f1 (ppm)

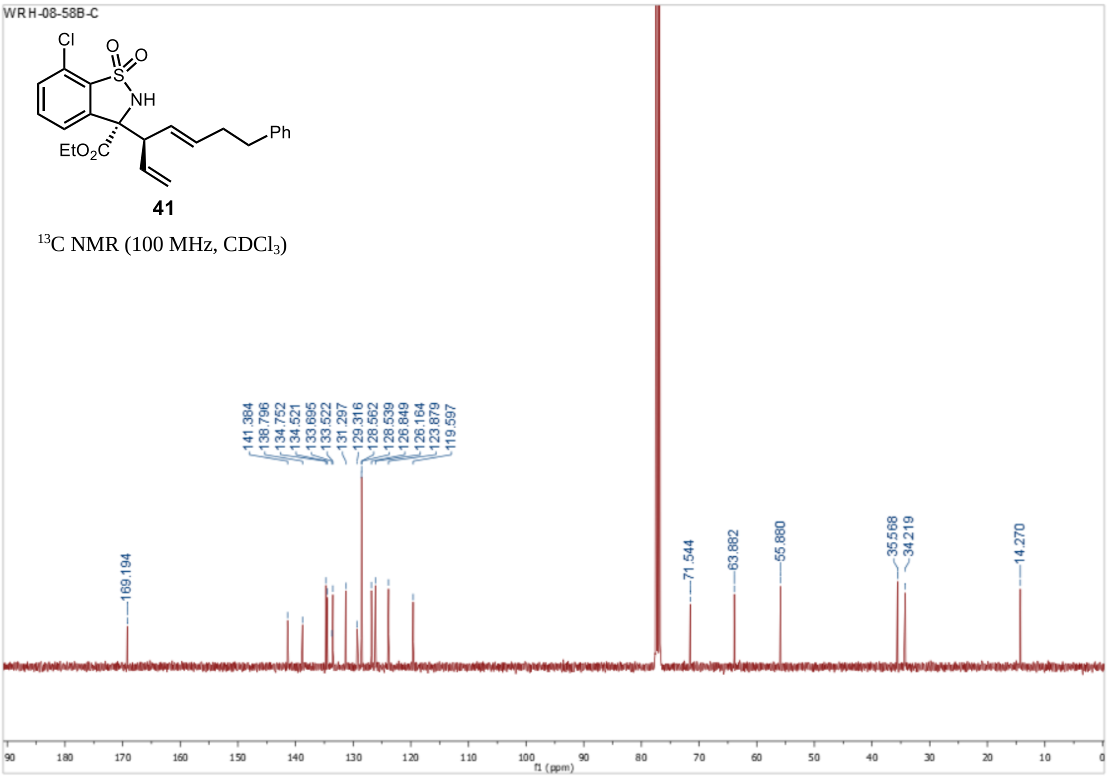
WRH-08-58B-C
Cl
O
S
O
NH
EtO2C
Ph
41
13C NMR (100 MHz, CDCl3)
169.194
71.544
63.882
55.880
35.568
34.219
14.270
f1 (ppm)

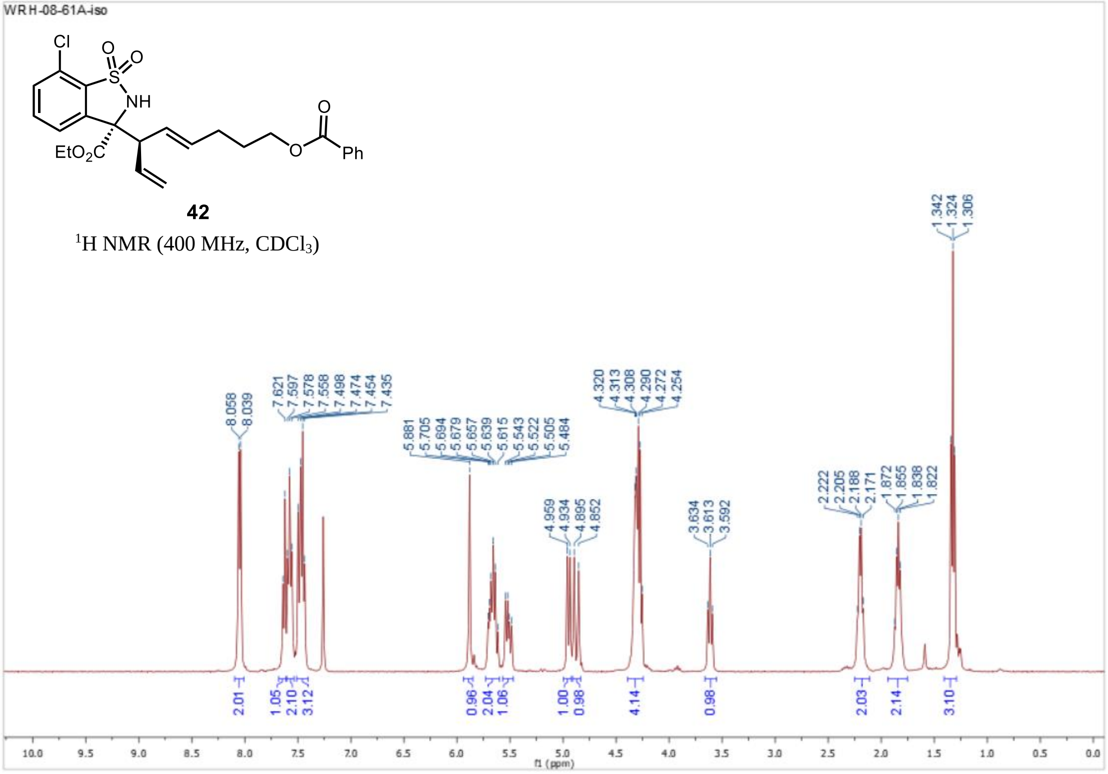
WRH-08-61A-iso
Cl
O
S
O
NH
EtO2C
O
O
Ph
42
1H NMR (400 MHz, CDCl3)
8.058
8.039
7.621
7.597
7.578
7.558
7.498
7.474
7.454
7.435
5.881
5.705
5.694
5.679
5.657
5.639
5.615
5.543
5.522
5.505
5.484
4.959
4.934
4.895
4.852
4.320
4.313
4.308
4.290
4.272
4.254
3.634
3.613
3.592
2.222
2.205
2.188
2.171
1.872
1.855
1.838
1.822
1.342
1.324
1.306
2.01
1.05
2.10
3.12
0.96
2.04
1.06
1.00
0.98
4.14
0.98
2.03
2.14
3.10
f1 (ppm)

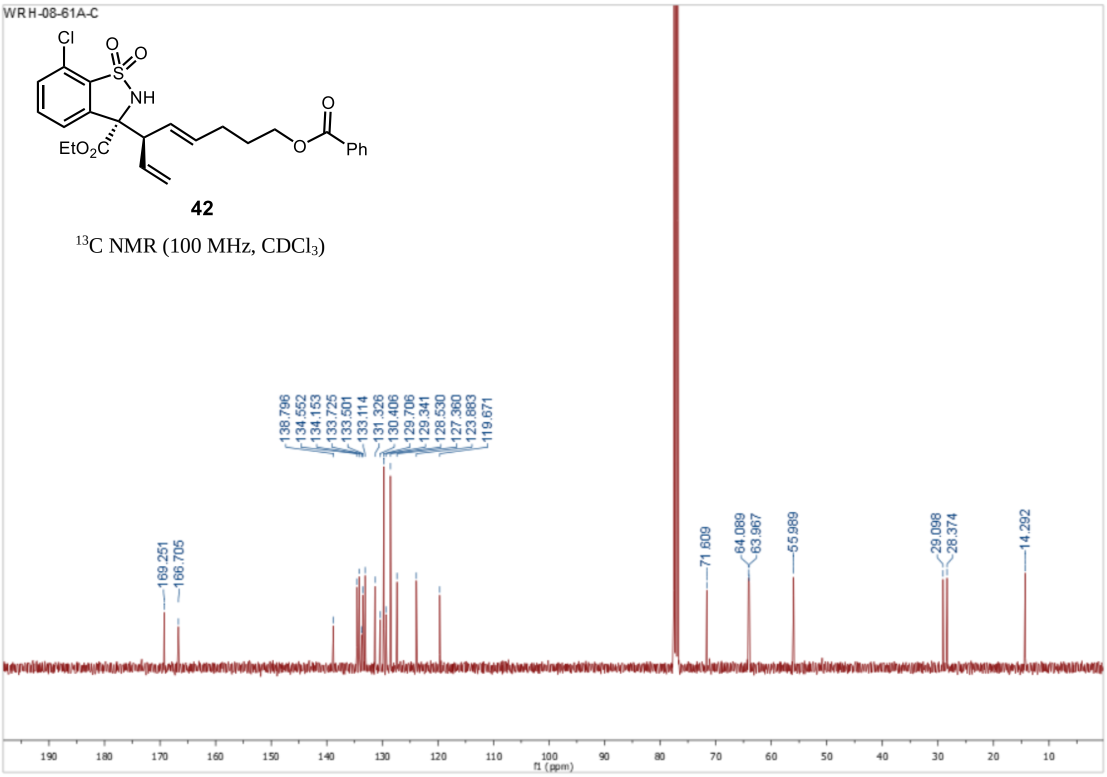
WRH-08-61A-C
Cl
O
S
O
NH
EtO2C
O
O
Ph
42
13C NMR (100 MHz, CDCl3)
169.251
166.705
138.796
134.552
134.153
133.725
133.501
133.114
131.326
130.406
129.706
129.341
128.530
127.360
123.883
119.671
71.609
64.089
63.967
55.989
29.098
28.374
14.292
f1 (ppm)

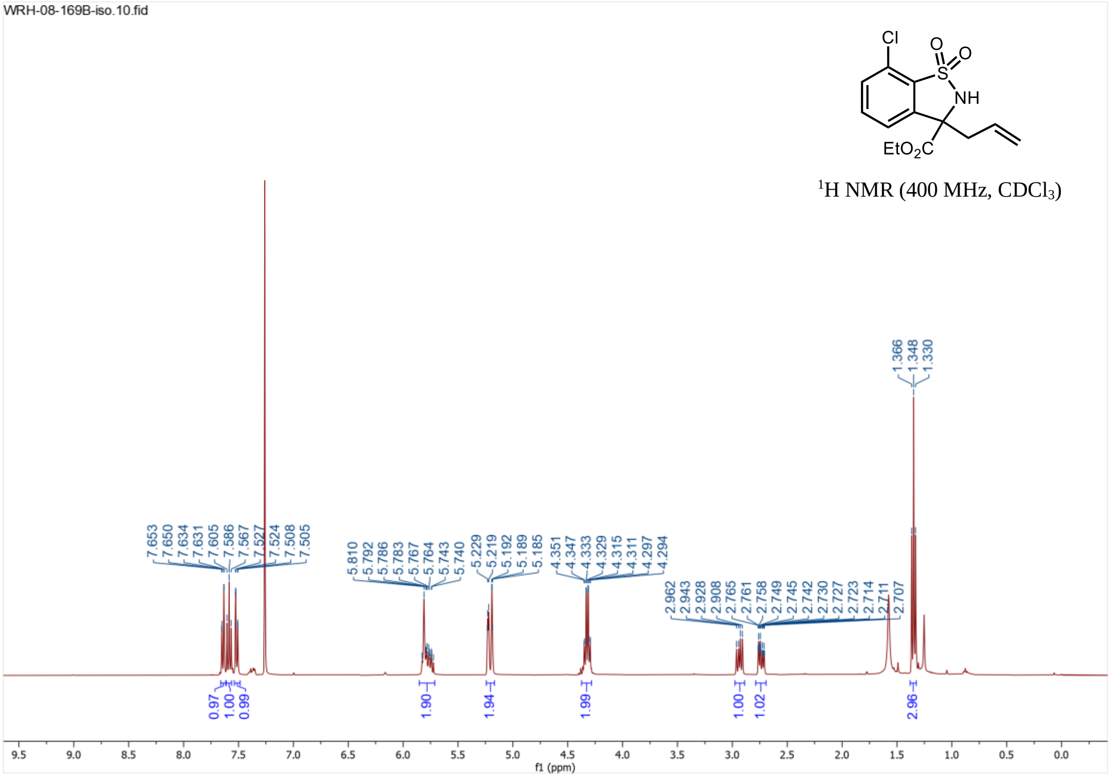
WRH-08-169B-iso.10.fid
Cl
O
O
S
NH
EtO2C
1H NMR (400 MHz, CDCl3)
f1 (ppm)

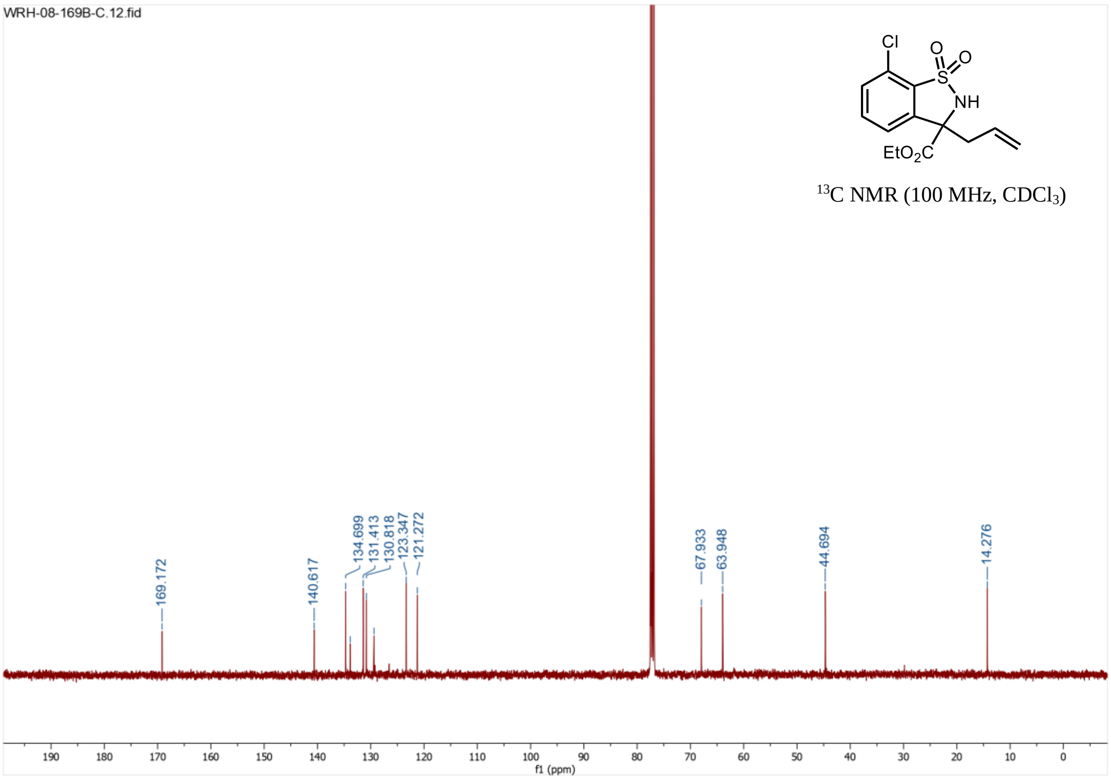
WRH-08-169B-C.12.fid
Cl
O
O
S
NH
EtO2C
13C NMR (100 MHz, CDCl3)
169.172
140.617
134.699
131.413
130.818
123.347
121.272
67.933
63.948
44.694
14.276
f1 (ppm)

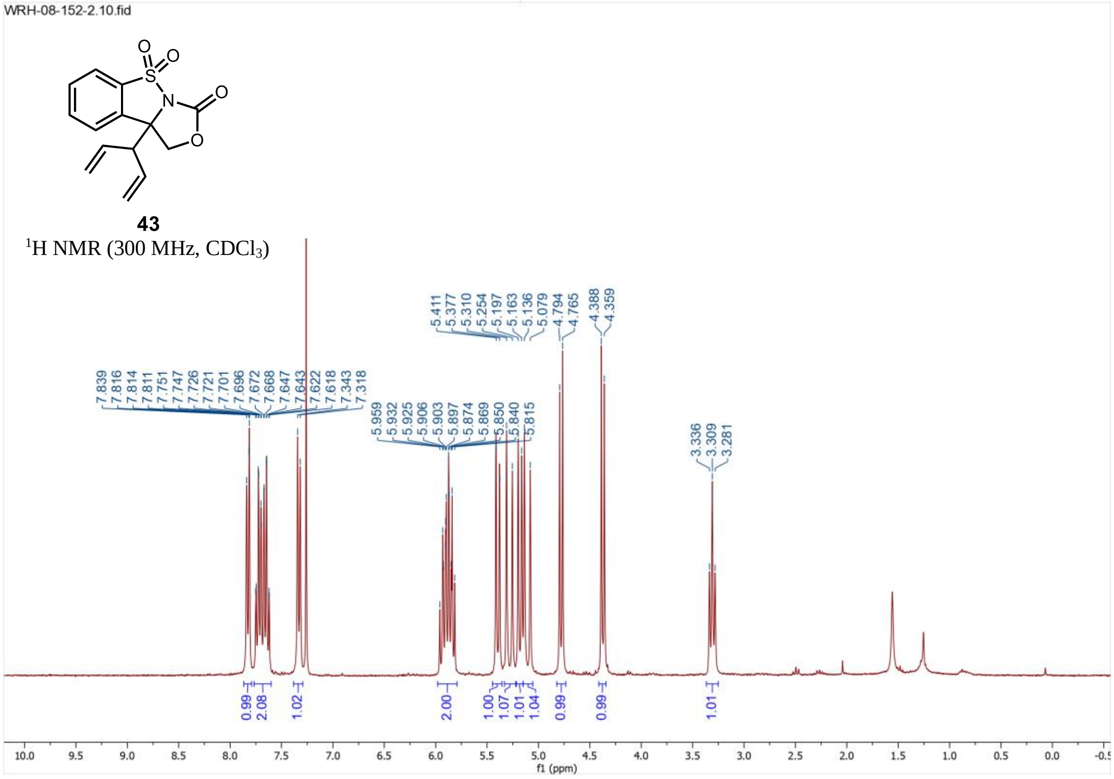

43

^{1}H NMR (300 MHz, $CDCl_3$)

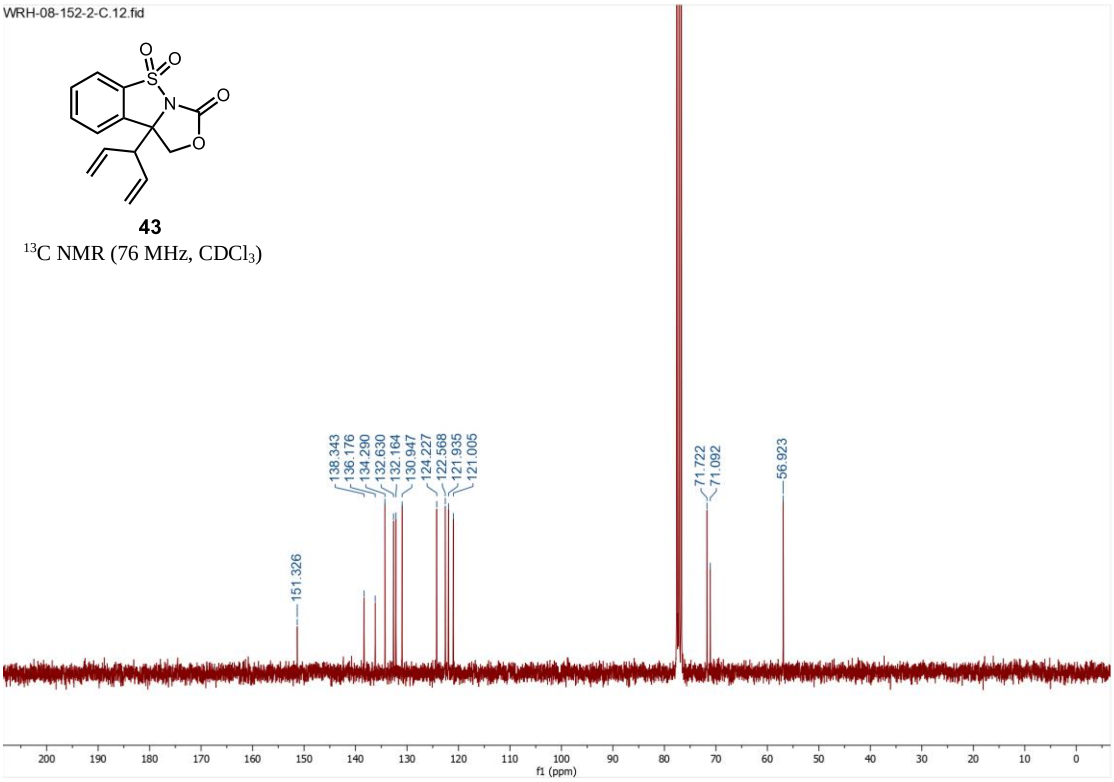

43

^{13}C NMR (76 MHz, $CDCl_3$)

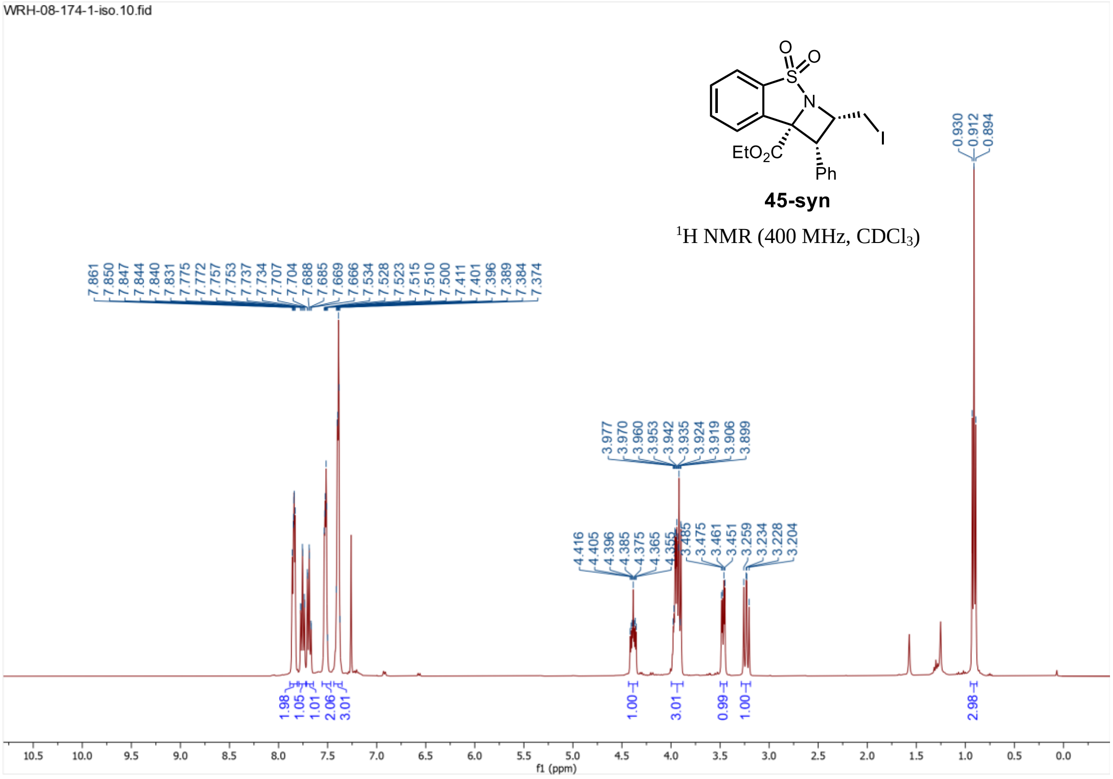
WRH-08-174-1-iso.10.fid
45-syn
¹H NMR (400 MHz, CDCl₃)
f1 (ppm)

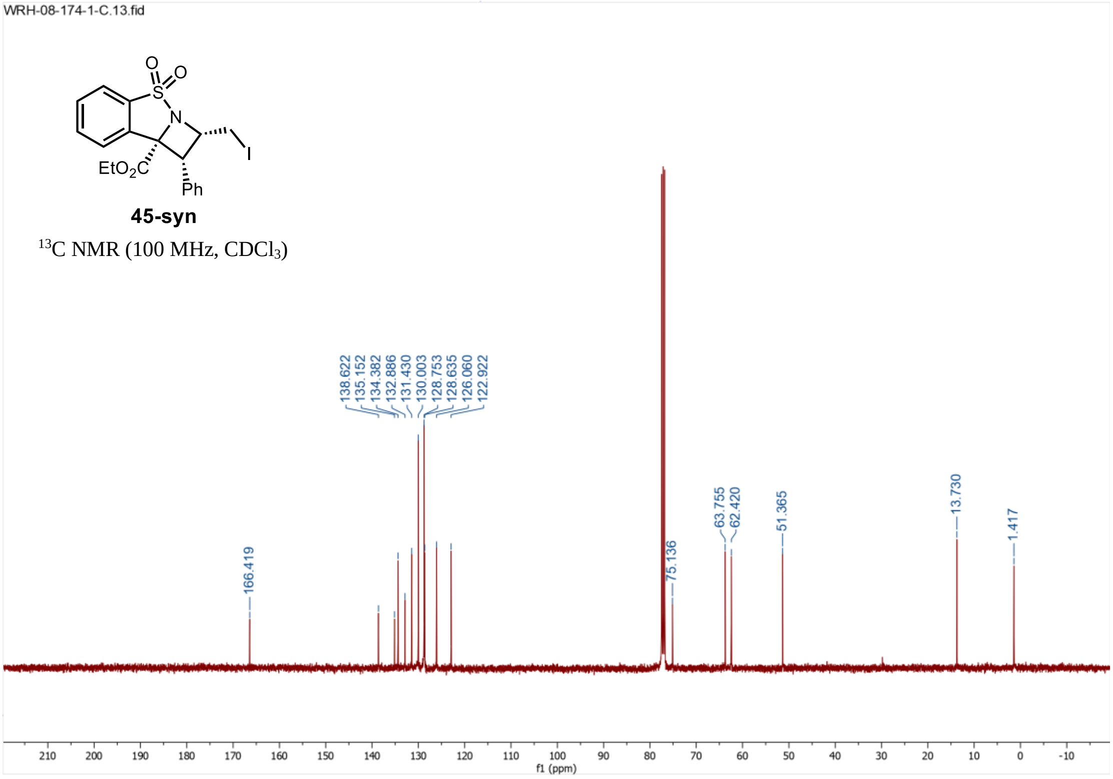
WRH-08-174-1-C.13.fid
45-syn
¹³C NMR (100 MHz, CDCl₃)
f1 (ppm)

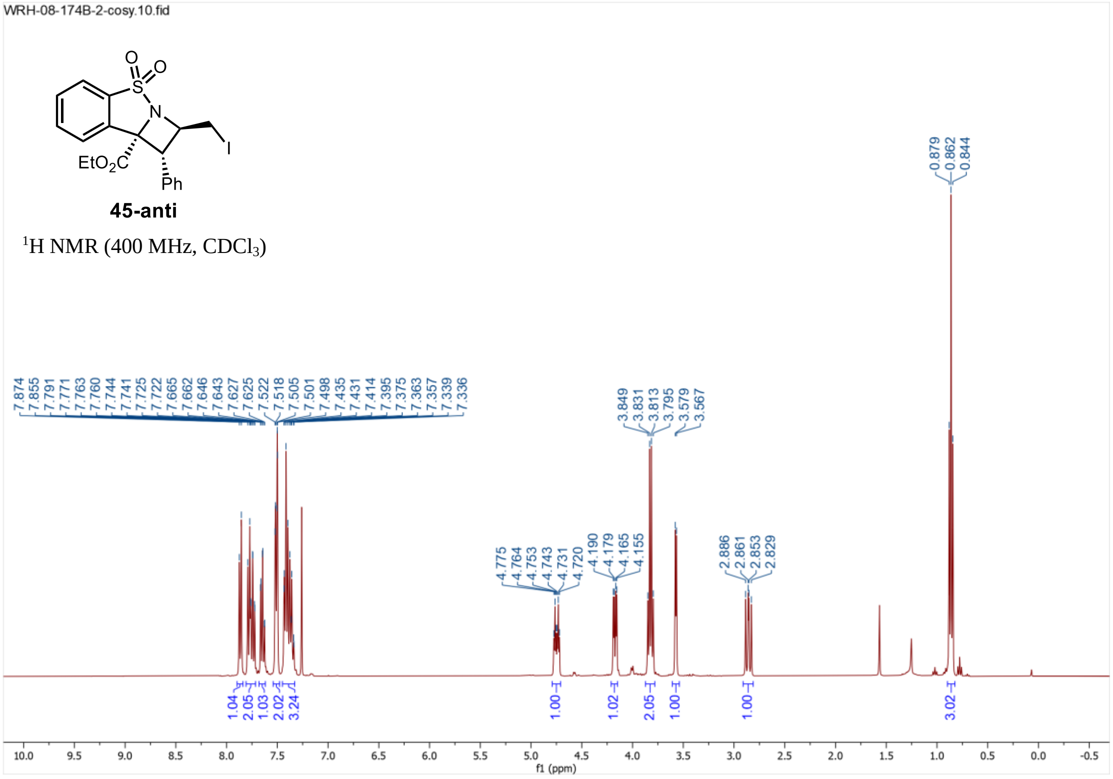
WRH-08-174B-2-cosy.10.fid
45-anti
¹H NMR (400 MHz, CDCl₃)
f1 (ppm)

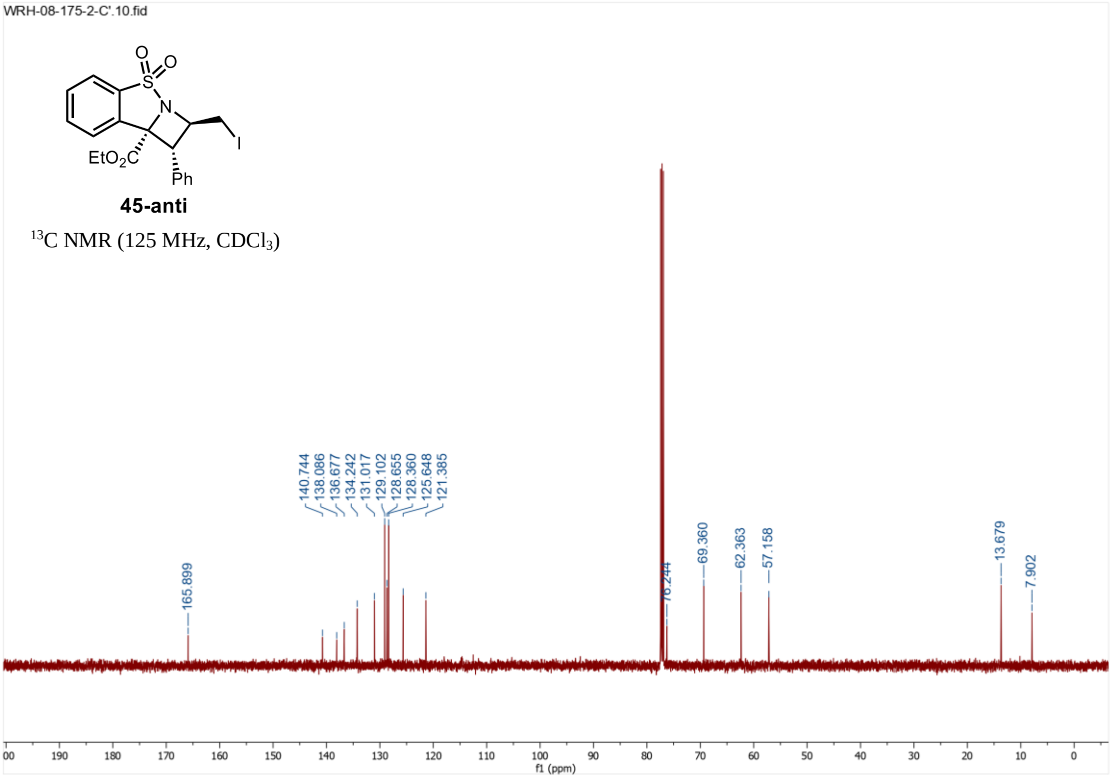
WRH-08-175-2-C'.10.fid
45-anti
¹³C NMR (125 MHz, CDCl₃)
f1 (ppm)

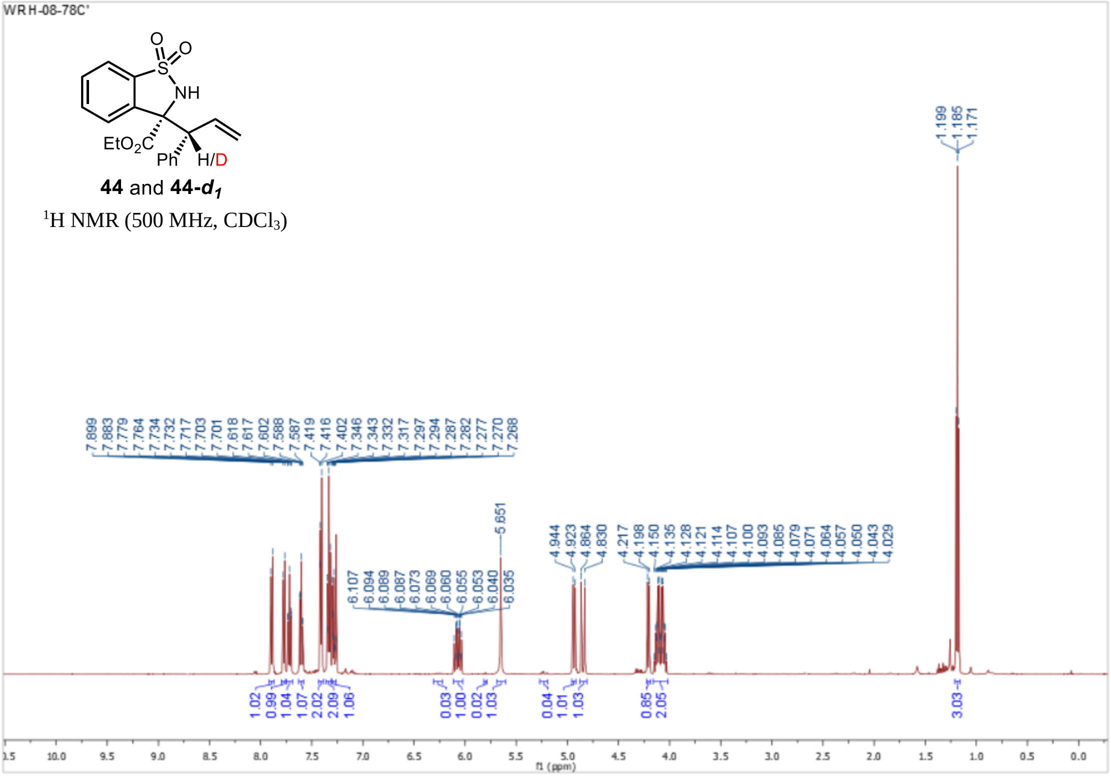
WRH-08-78C'
44 and 44-d1
1H NMR (500 MHz, CDCl3)
f1 (ppm)

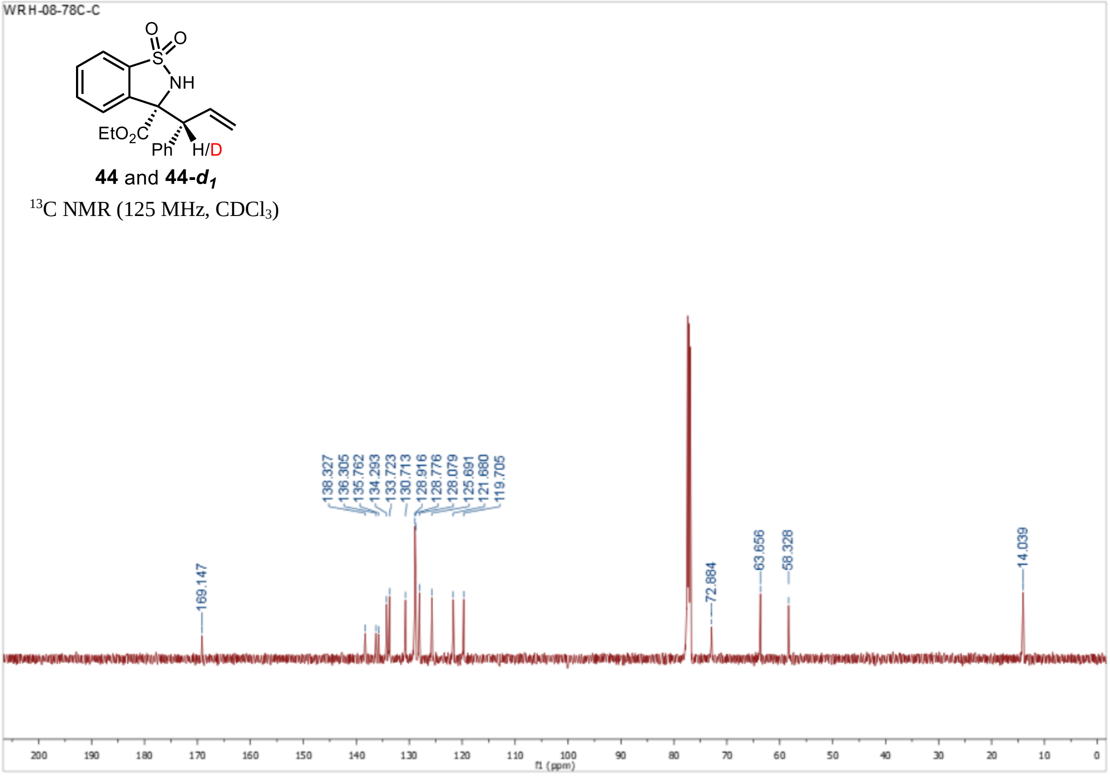
WRH-08-78C-C
44 and 44-d1
13C NMR (125 MHz, CDCl3)
169.147
72.884
63.656
58.328
14.039
f1 (ppm)

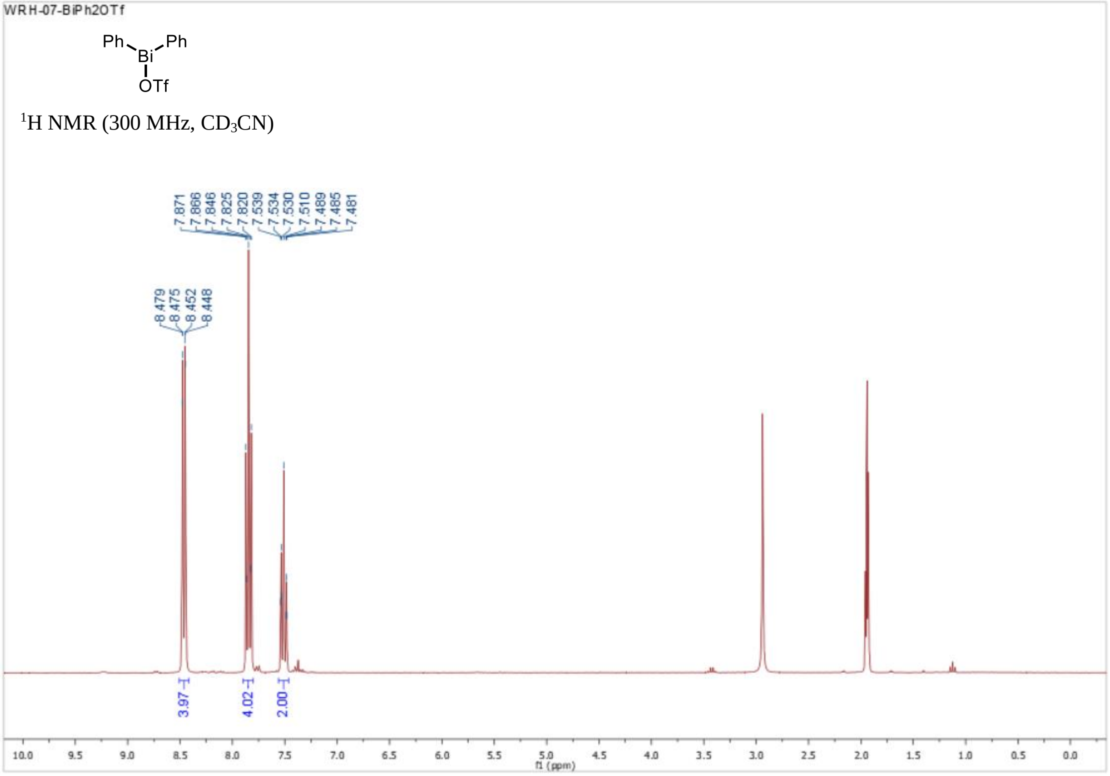
WRH-07-BiPh2OTf
Ph
Bi
Ph
OTf
1H NMR (300 MHz, CD3CN)
7.871
7.866
7.846
7.825
7.820
7.539
7.534
7.530
7.510
7.489
7.485
7.481
8.479
8.475
8.452
8.448
3.97
4.02
2.00
f1 (ppm)

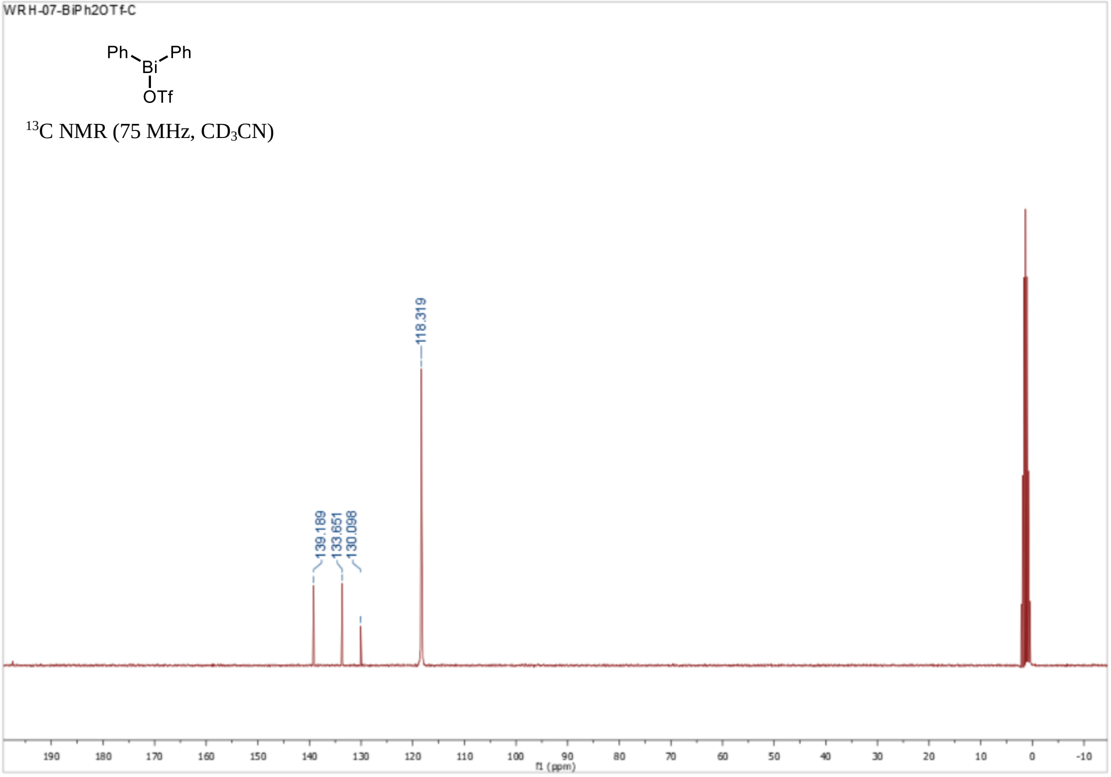
WRH-07-BiPh2OTf-C
Ph
Bi
Ph
OTf
13C NMR (75 MHz, CD3CN)
118.319
139.189
133.651
130.098
f1 (ppm)

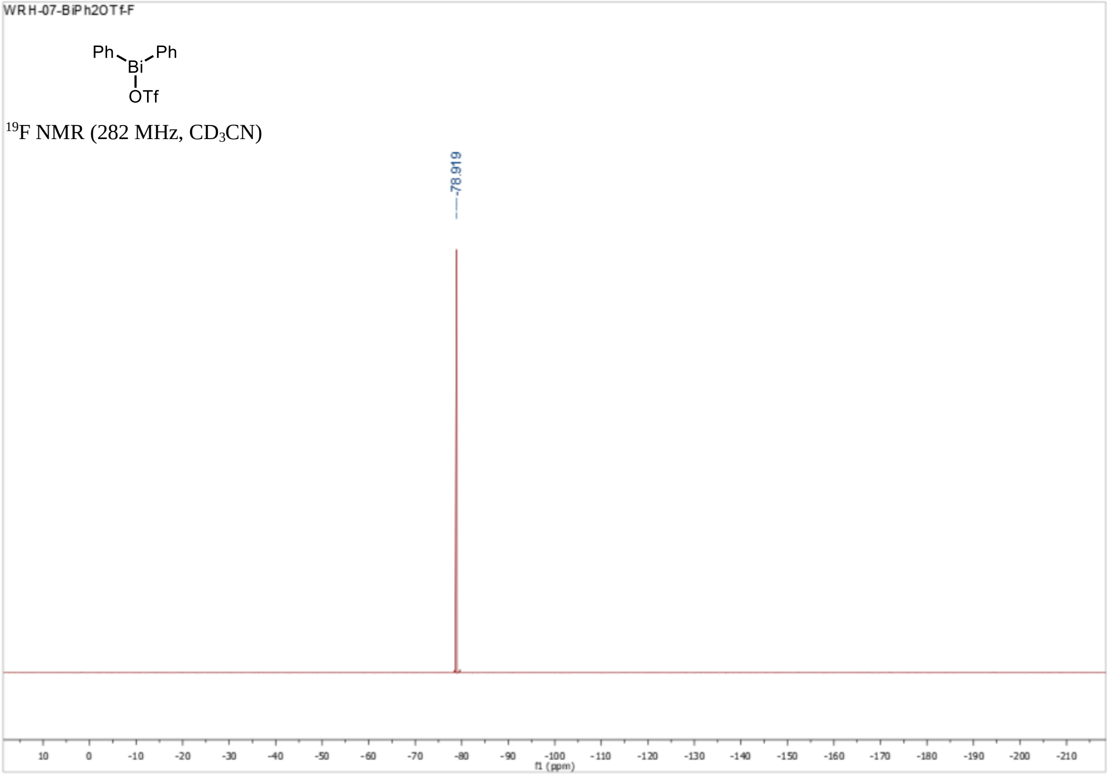
WRH-07-BiPh2OTf-F
Ph
Ph
Bi
OTf
19F NMR (282 MHz, CD3CN)
-78.919
10
0
-10
-20
-30
-40
-50
-60
-70
-80
-90
-100
-110
-120
-130
-140
-150
-160
-170
-180
-190
-200
-210
f1 (ppm)

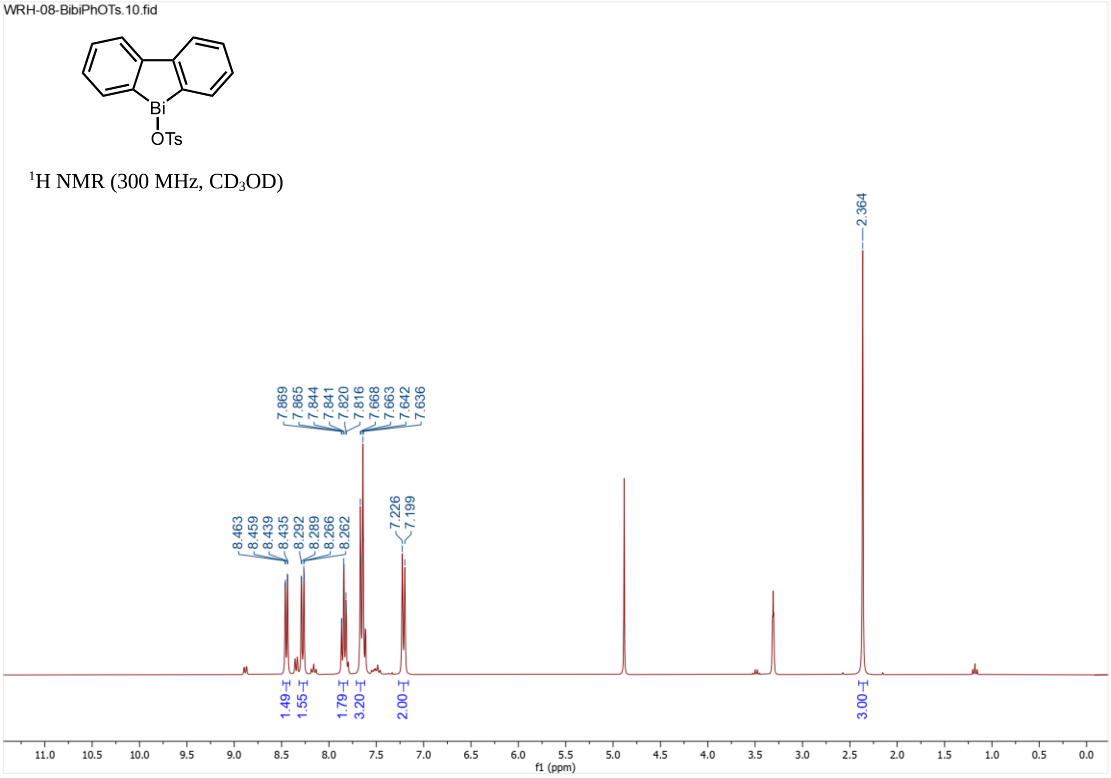
WRH-08-BibiPhOTs.10.fid
Bi
OTs
^{1}H NMR (300 MHz, CD_3OD)
2.364
7.869 7.865 7.844 7.841 7.820 7.816 7.668 7.663 7.642 7.636
8.463 8.459 8.439 8.435 8.292 8.289 8.266 8.262
7.226 7.199
1.49 1.55 1.79 3.20 2.00 3.00
f1 (ppm)

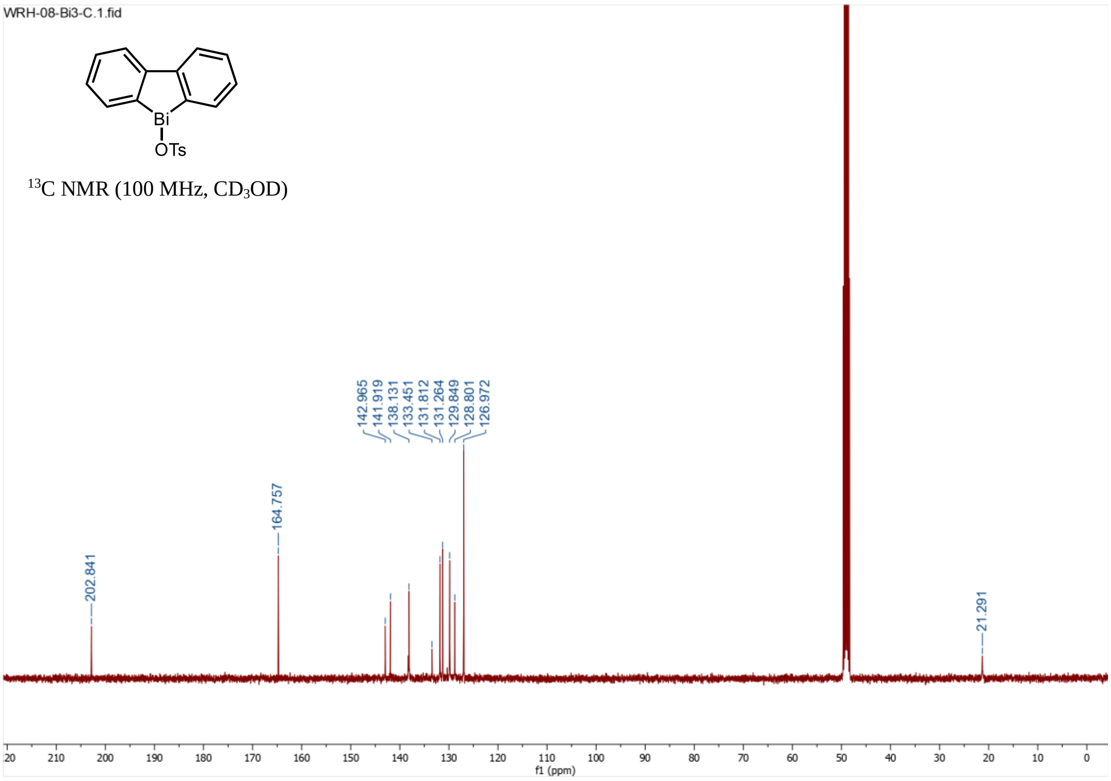
WRH-08-Bi3-C.1.fid
Bi
OTs
^{13}C NMR (100 MHz, CD_3OD)
142.965 141.919 138.131 133.451 131.812 131.264 129.849 128.801 126.972
164.757
202.841
21.291
f1 (ppm)

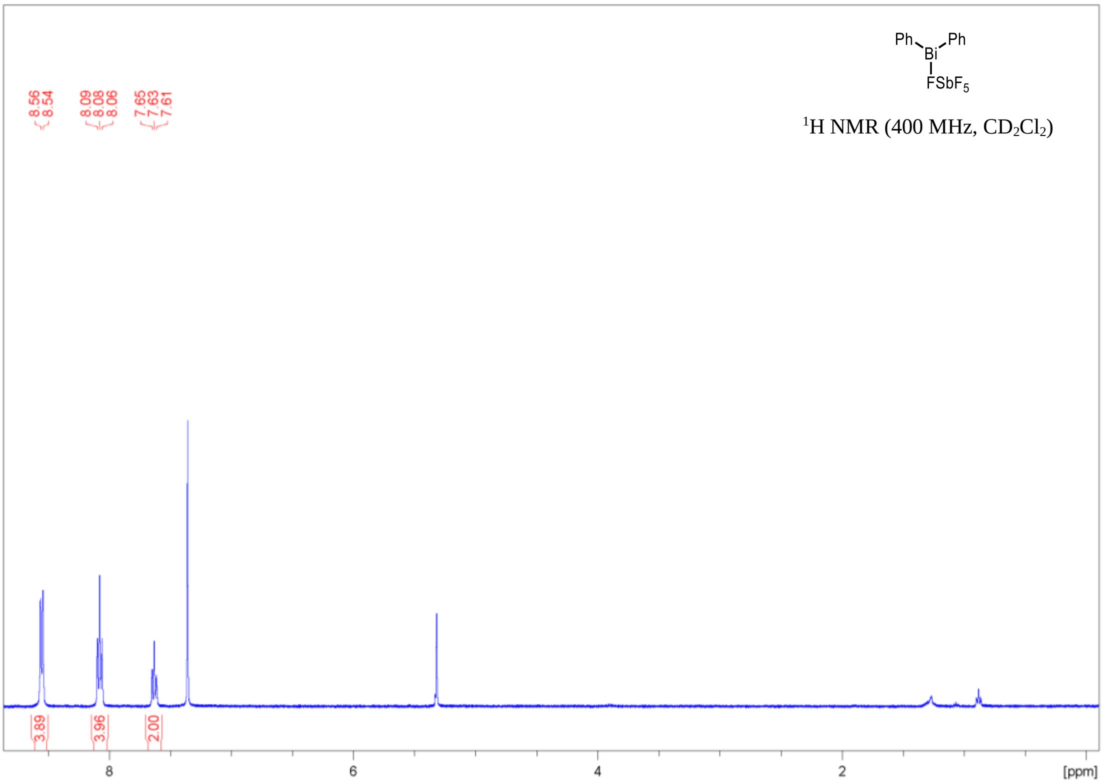
Ph Ph
Bi
FSbF5
1H NMR (400 MHz, CD2Cl2)
8.56
8.54
8.09
8.08
8.06
7.65
7.63
7.61
3.89
3.96
2.00
8
6
4
2
[ppm]

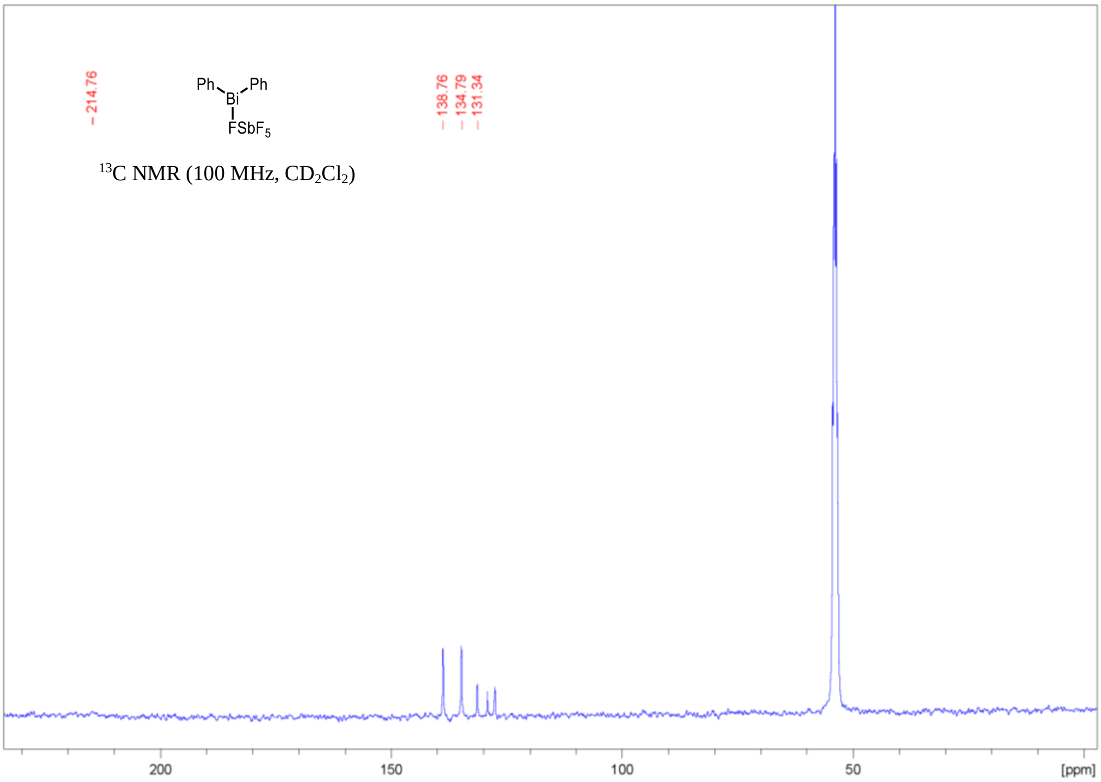
214.76
Ph Ph
Bi
FSbF5
13C NMR (100 MHz, CD2Cl2)
138.76
134.79
131.34
200
150
100
50
[ppm]

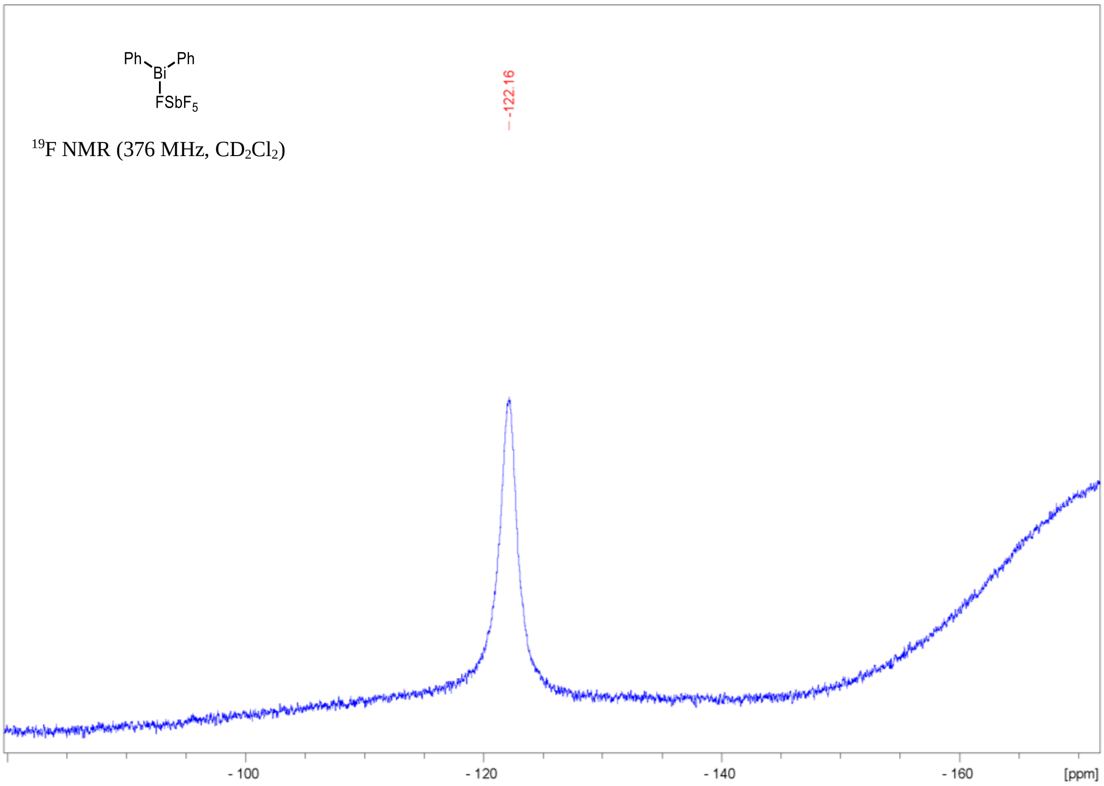

Ph
Bi
Ph
FSbF5
19F NMR (376 MHz, CD2Cl2)
-122.16
- 100
- 120
- 140
- 160
[ppm]

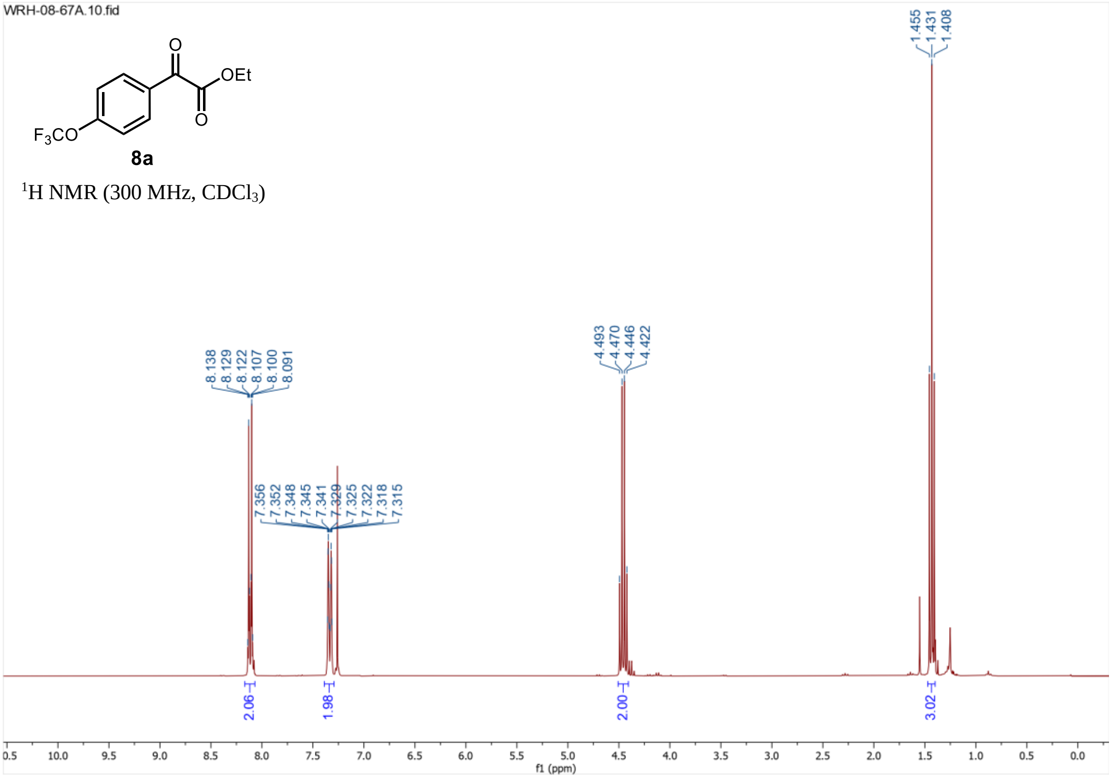
WRH-08-67A.10.fid
OEt
F3CO
8a
1H NMR (300 MHz, CDCl3)
8.138
8.129
8.122
8.107
8.100
8.091
7.356
7.352
7.348
7.345
7.341
7.329
7.325
7.322
7.318
7.315
4.493
4.470
4.446
4.422
1.455
1.431
1.408
2.06
1.98
2.00
3.02
f1 (ppm)

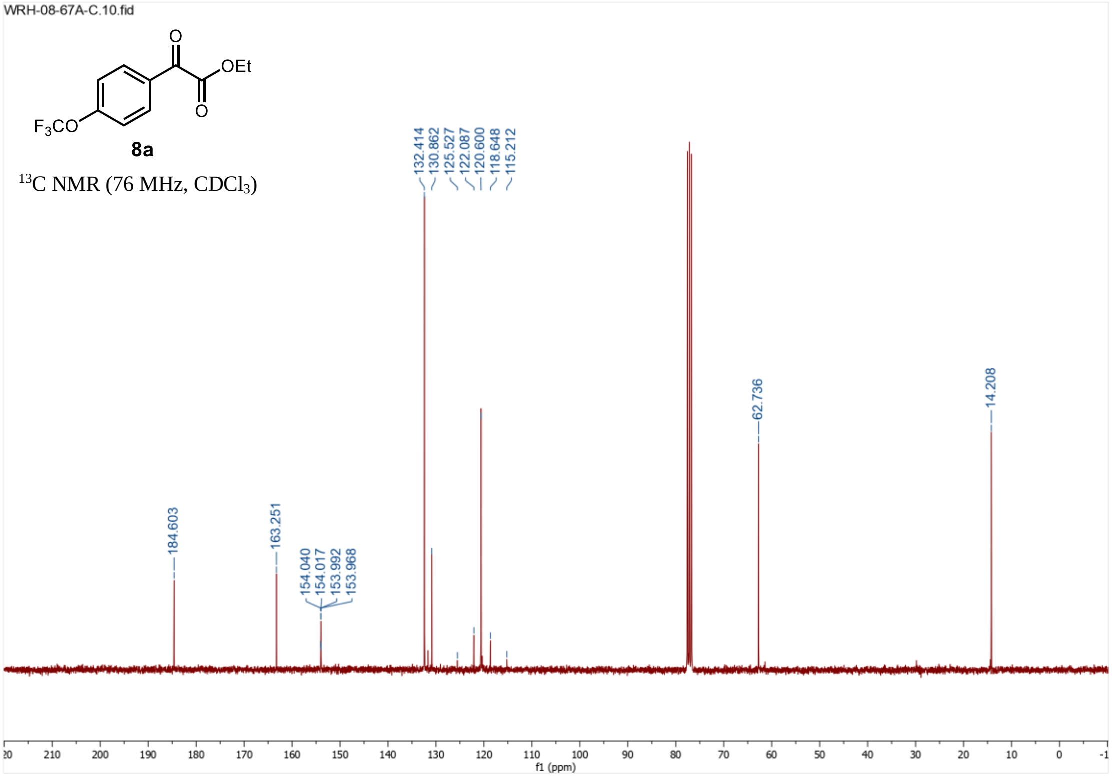
WRH-08-67A-C.10.fid
OEt
F3CO
8a
13C NMR (76 MHz, CDCl3)
132.414
130.862
125.527
122.087
120.600
118.648
115.212
184.603
163.251
154.040
154.017
153.992
153.968
62.736
14.208
f1 (ppm)

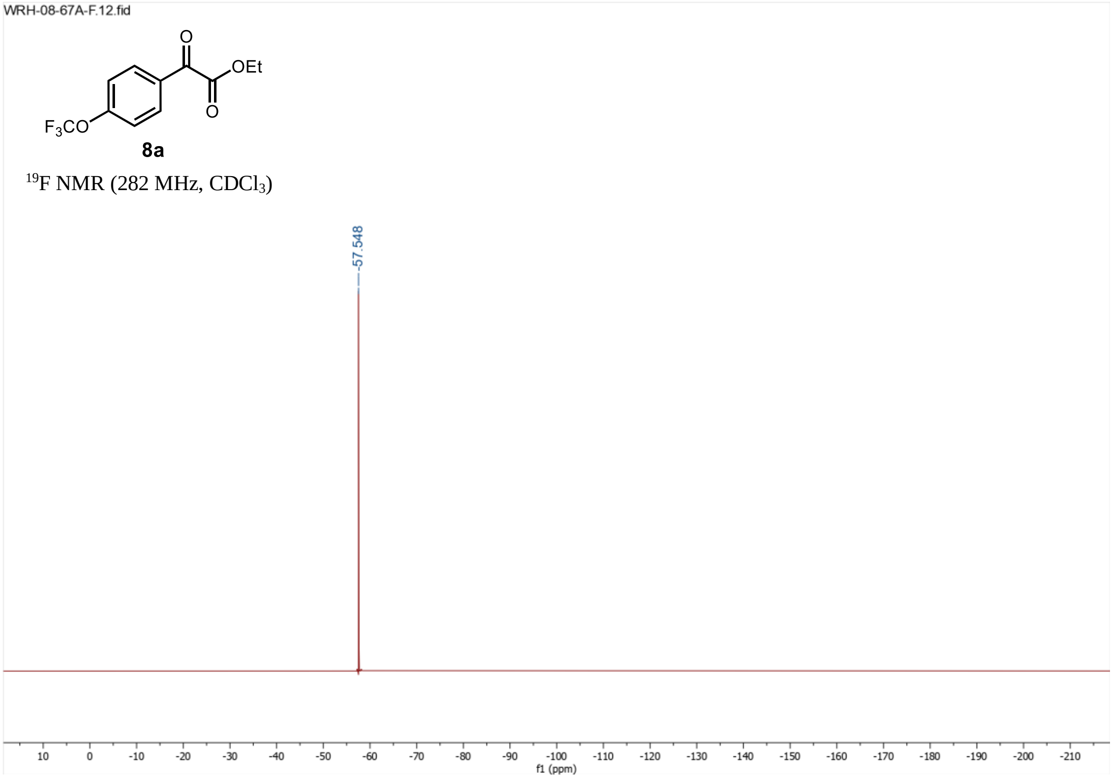
WRH-08-67A-F.12.fid
OEt
F3CO
8a
19F NMR (282 MHz, CDCl3)
-57.548
f1 (ppm)

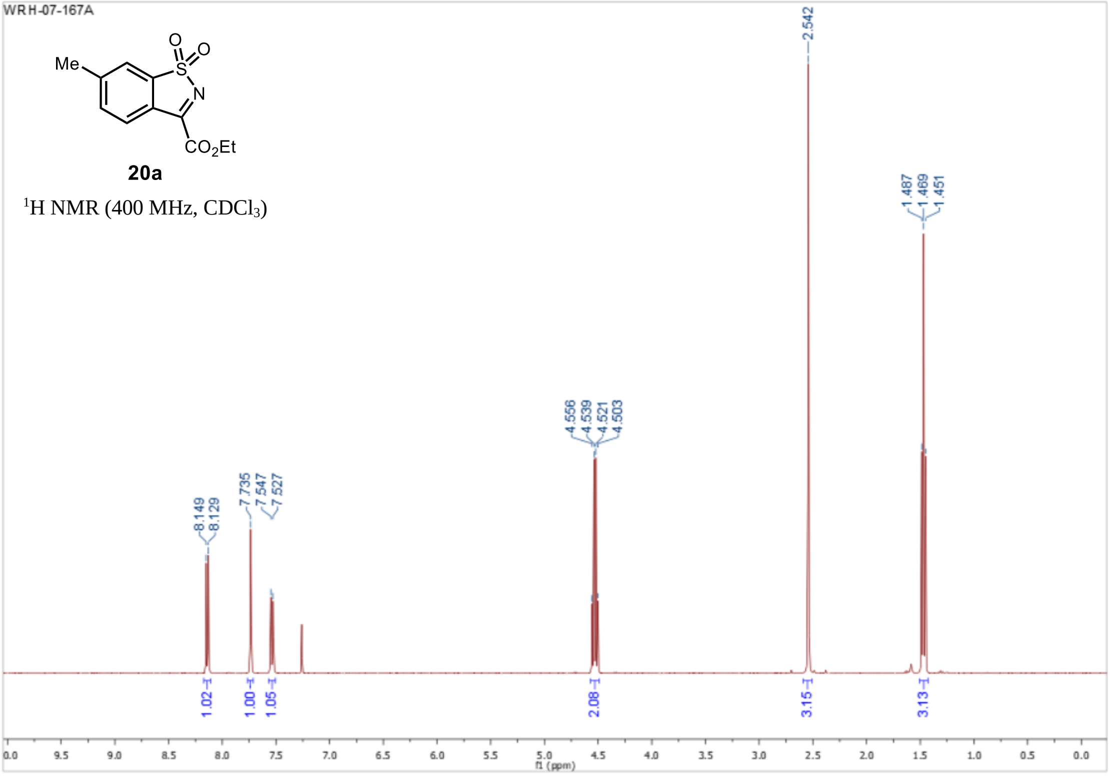
WRH-07-167A
Me
O
O
S
N
CO2Et
20a
1H NMR (400 MHz, CDCl3)
8.149
8.129
7.735
7.547
7.527
4.556
4.539
4.521
4.503
2.542
1.487
1.469
1.451
1.02
1.00
1.05
2.08
3.15
3.13
f1 (ppm)

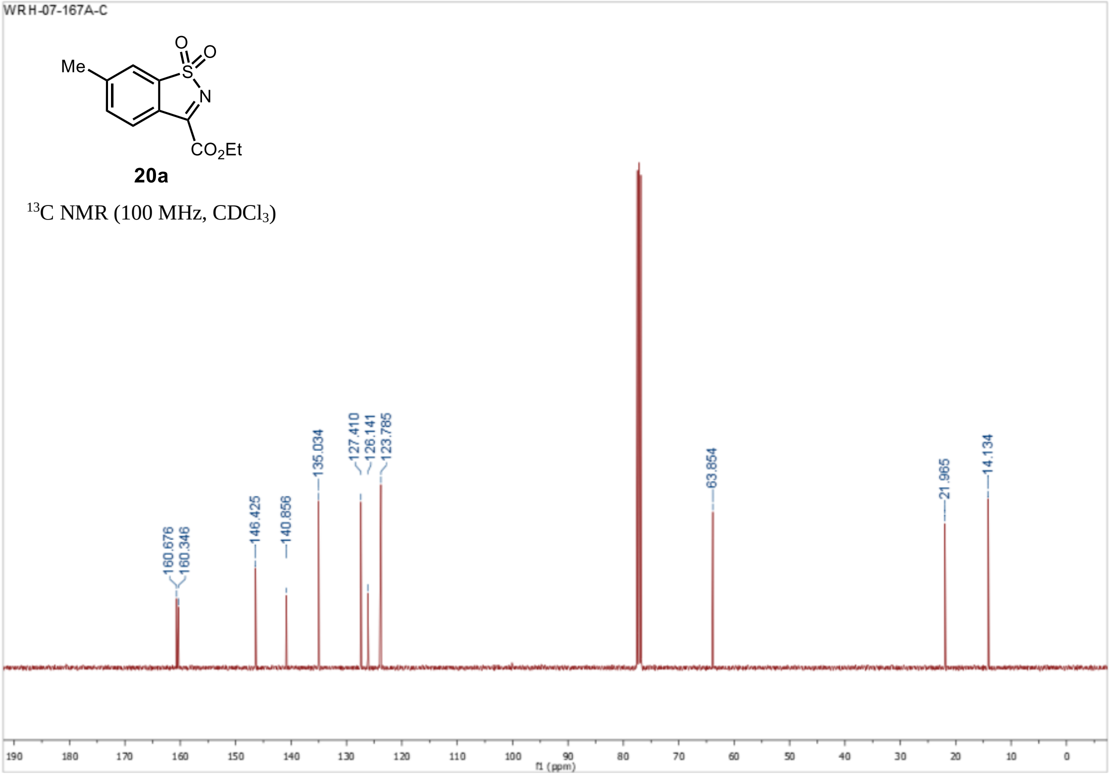
WRH-07-167A-C
Me
O
O
S
N
CO2Et
20a
13C NMR (100 MHz, CDCl3)
160.676
160.346
146.425
140.856
135.034
127.410
126.141
123.785
63.854
21.965
14.134
f1 (ppm)

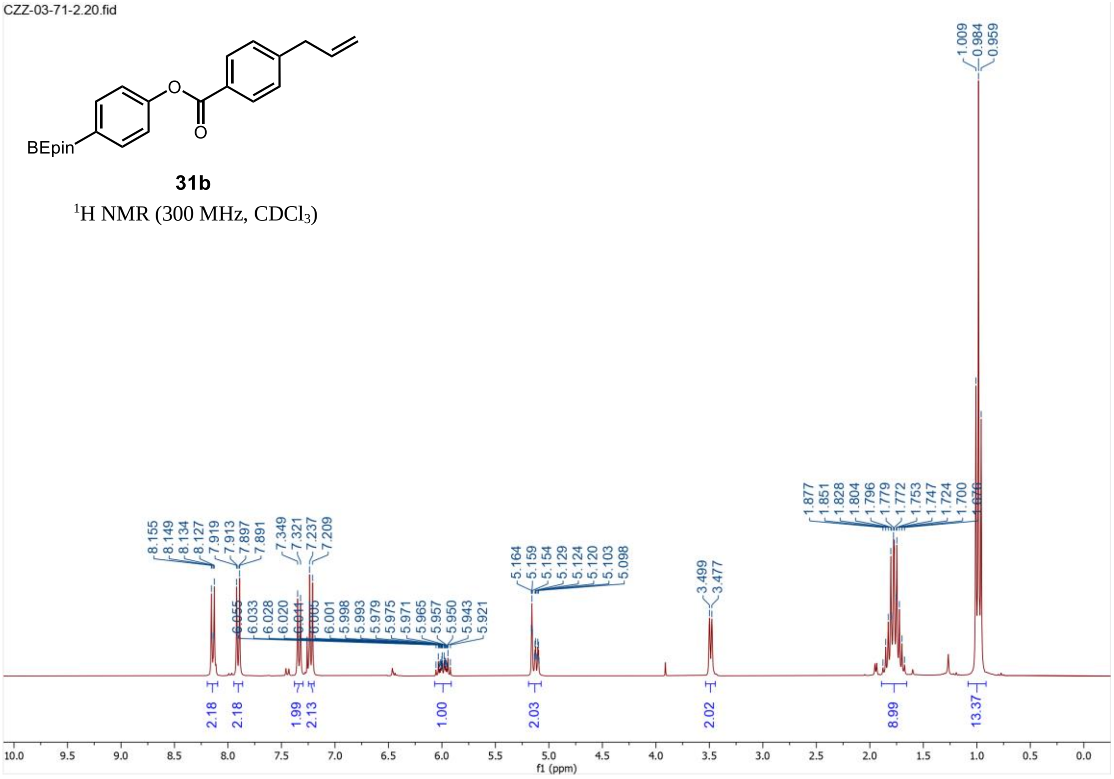
CZZ-03-71-2.20.fid
BEpin
O
O
31b
^{1}H NMR (300 MHz, CDCl$_3$)
8.155 8.149 8.134 8.127 7.919 7.913 7.897 7.891 7.349 7.321 7.237 7.209
6.055 6.033 6.028 6.020 6.011 6.005 6.001 5.998 5.993 5.979 5.975 5.971 5.965 5.957 5.950 5.943 5.921
5.164 5.159 5.154 5.129 5.124 5.120 5.103 5.098
3.499 3.477
1.877 1.851 1.828 1.804 1.796 1.779 1.772 1.753 1.747 1.724 1.700 1.676
1.009 0.984 0.959
2.18 2.18 1.99 2.13 1.00 2.03 2.02 8.99 13.37
f1 (ppm)

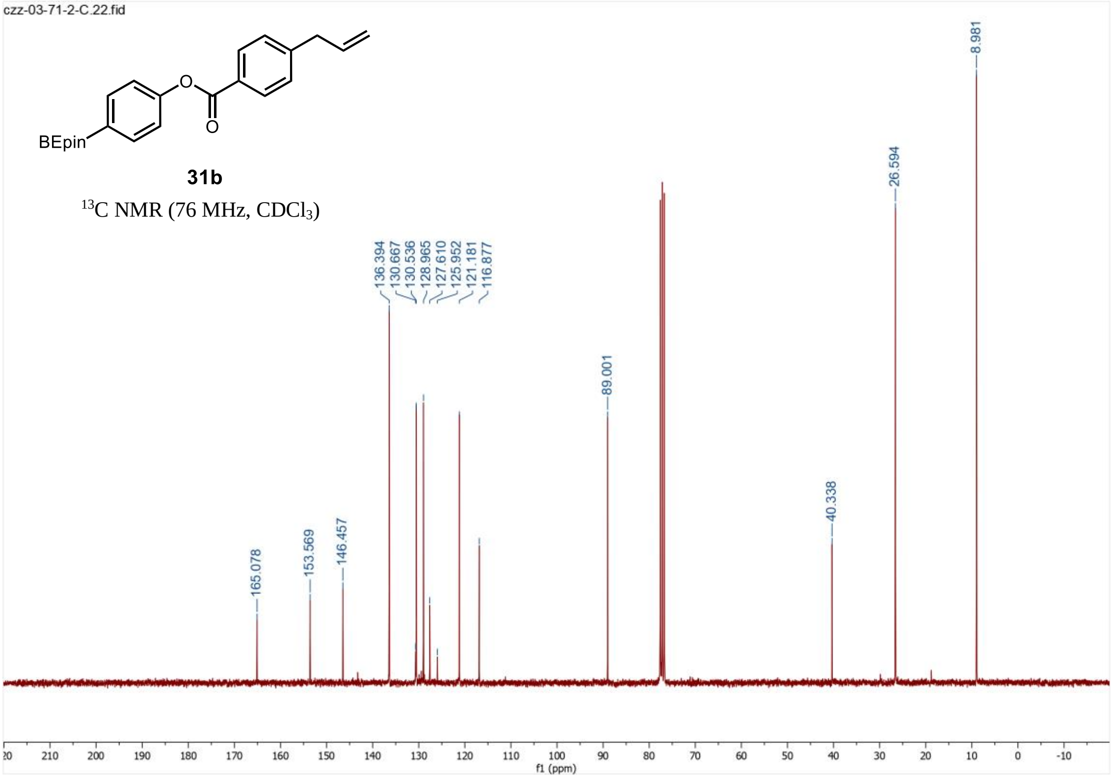
czz-03-71-2-C.22.fid
BEpin
O
O
31b
^{13}C NMR (76 MHz, CDCl$_3$)
165.078
153.569
146.457
136.394 130.667 130.536 128.965 127.610 125.952 121.181 116.877
89.001
40.338
26.594
8.981
f1 (ppm)

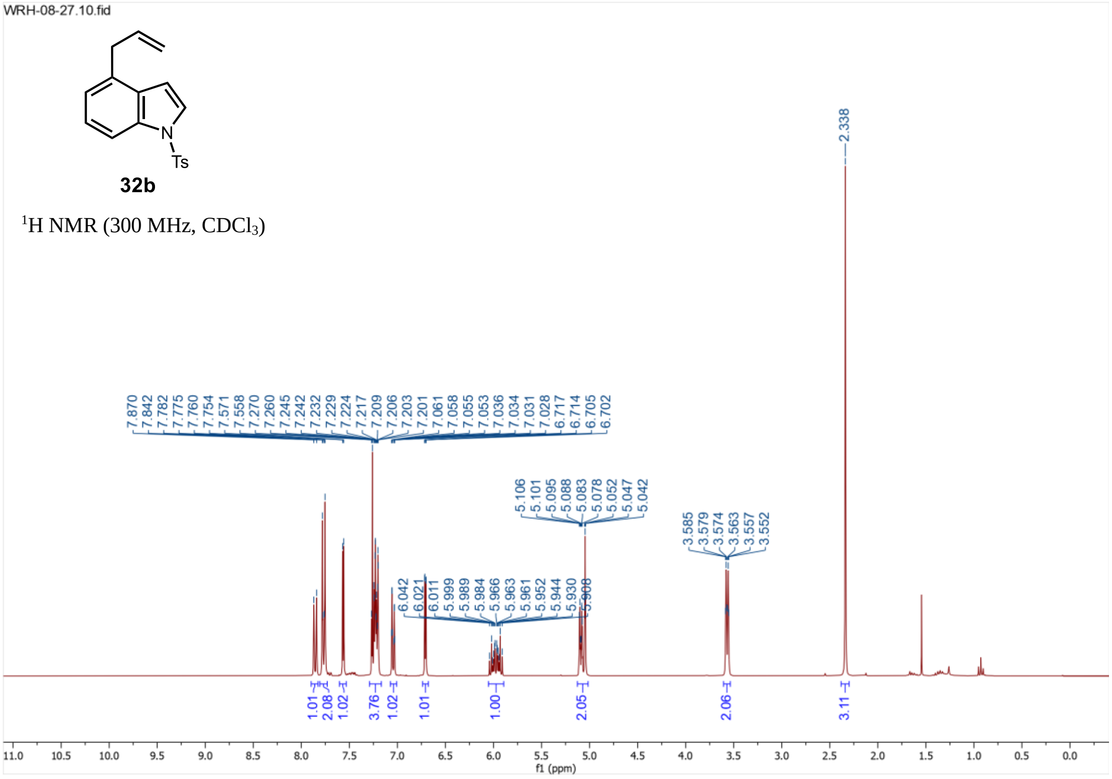

32b

^{1}H NMR (300 MHz, $CDCl_3$)

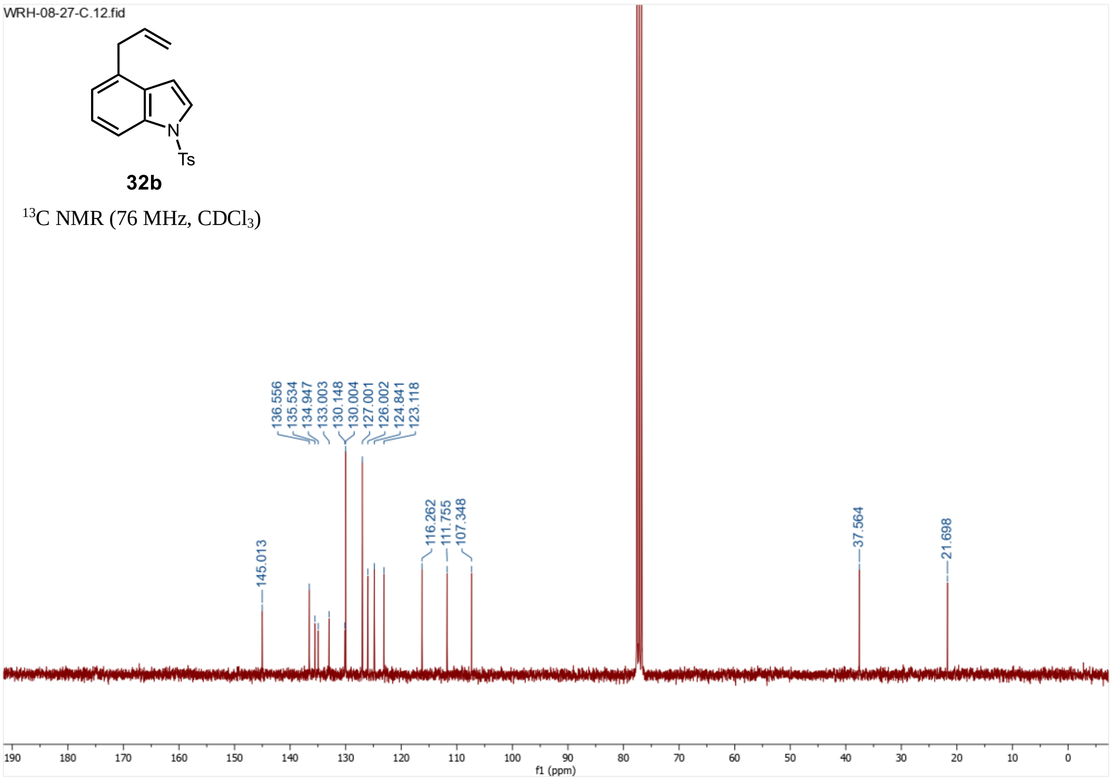

32b

^{13}C NMR (76 MHz, $CDCl_3$)

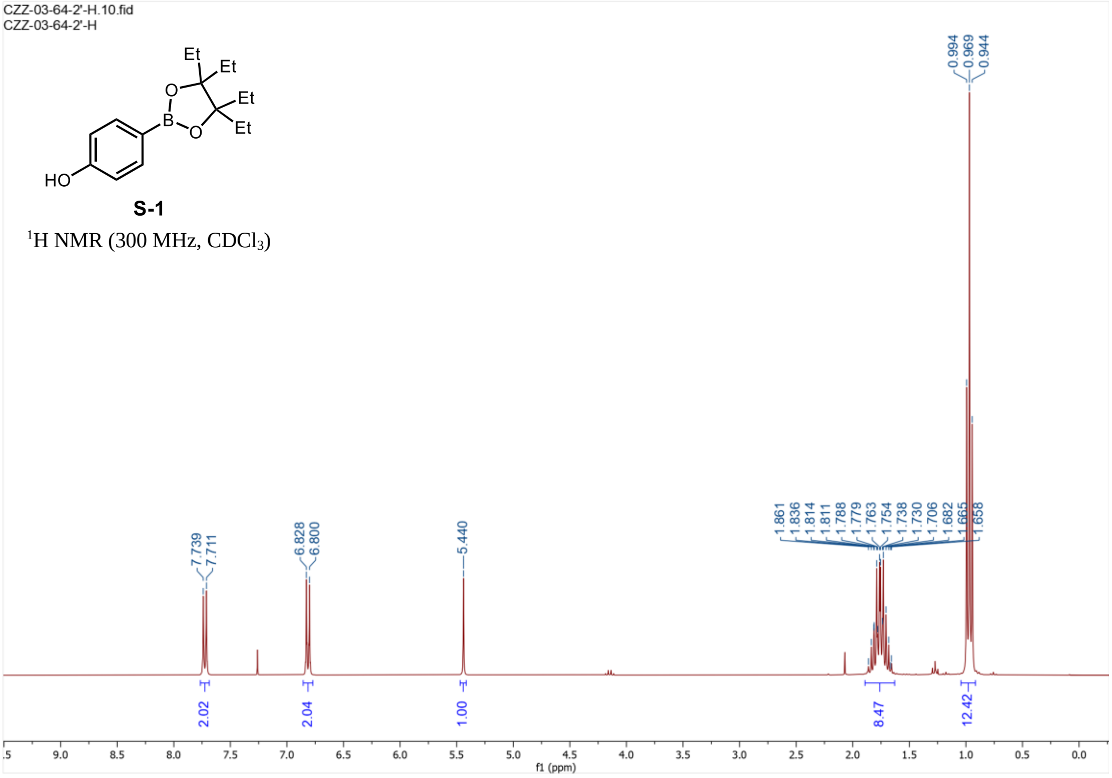
CZZ-03-64-2'-H.10.fid
CZZ-03-64-2'-H
Et
Et
Et
Et
O
B
O
HO
S-1
1H NMR (300 MHz, CDCl3)
7.739
7.711
6.828
6.800
5.440
1.861
1.836
1.814
1.811
1.788
1.779
1.763
1.754
1.738
1.730
1.706
1.682
1.665
1.658
0.994
0.969
0.944
2.02
2.04
1.00
8.47
12.42
f1 (ppm)

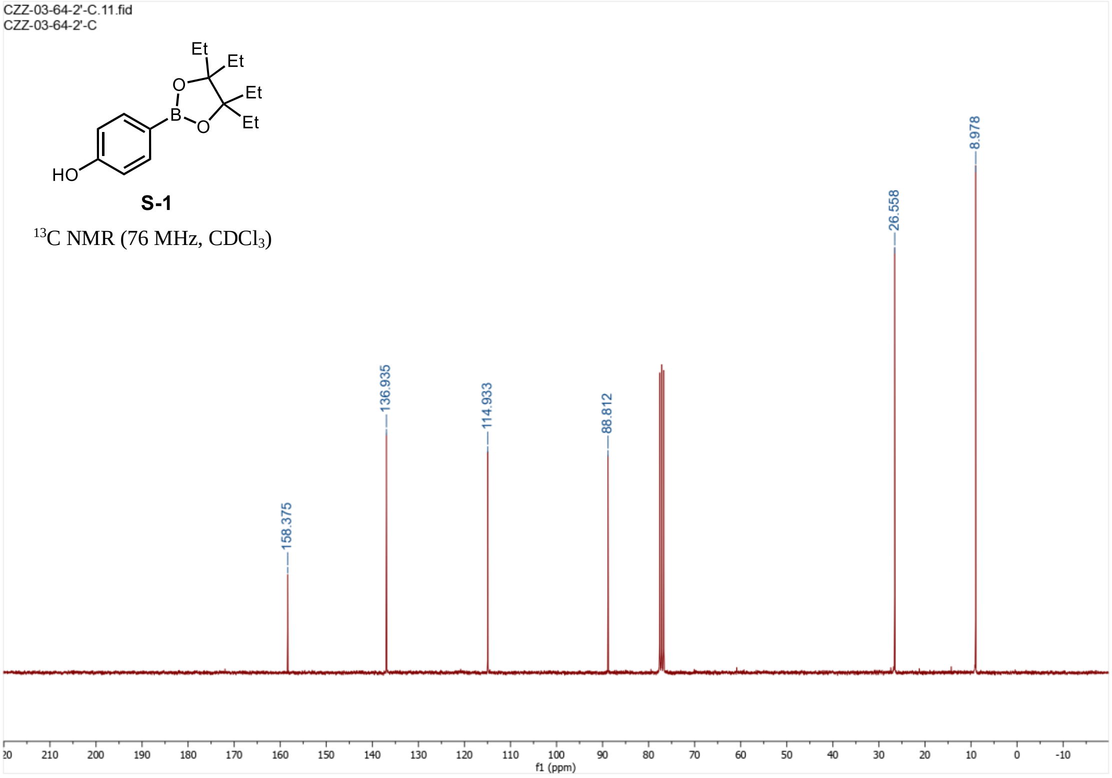
CZZ-03-64-2'-C.11.fid
CZZ-03-64-2'-C
Et
Et
Et
Et
O
B
O
HO
S-1
13C NMR (76 MHz, CDCl3)
158.375
136.935
114.933
88.812
26.558
8.978
f1 (ppm)

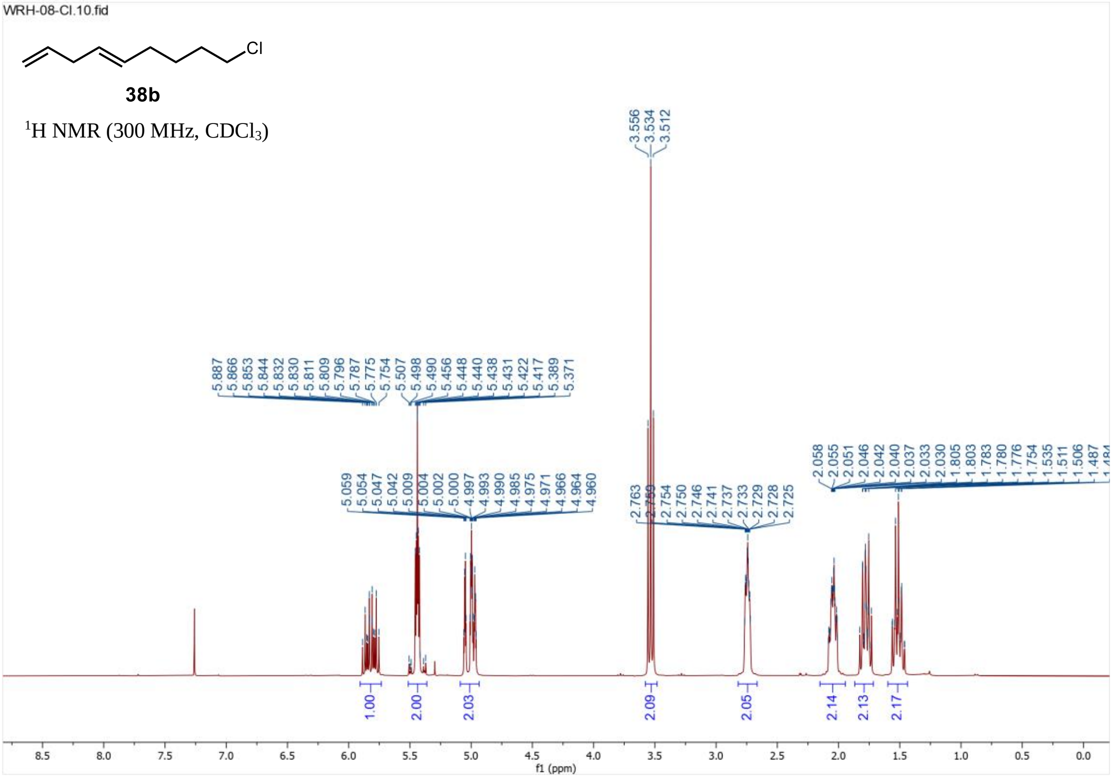
WRH-08-Cl.10.fid
Cl
38b
^{1}H NMR (300 MHz, CDCl$_3$)
f1 (ppm)

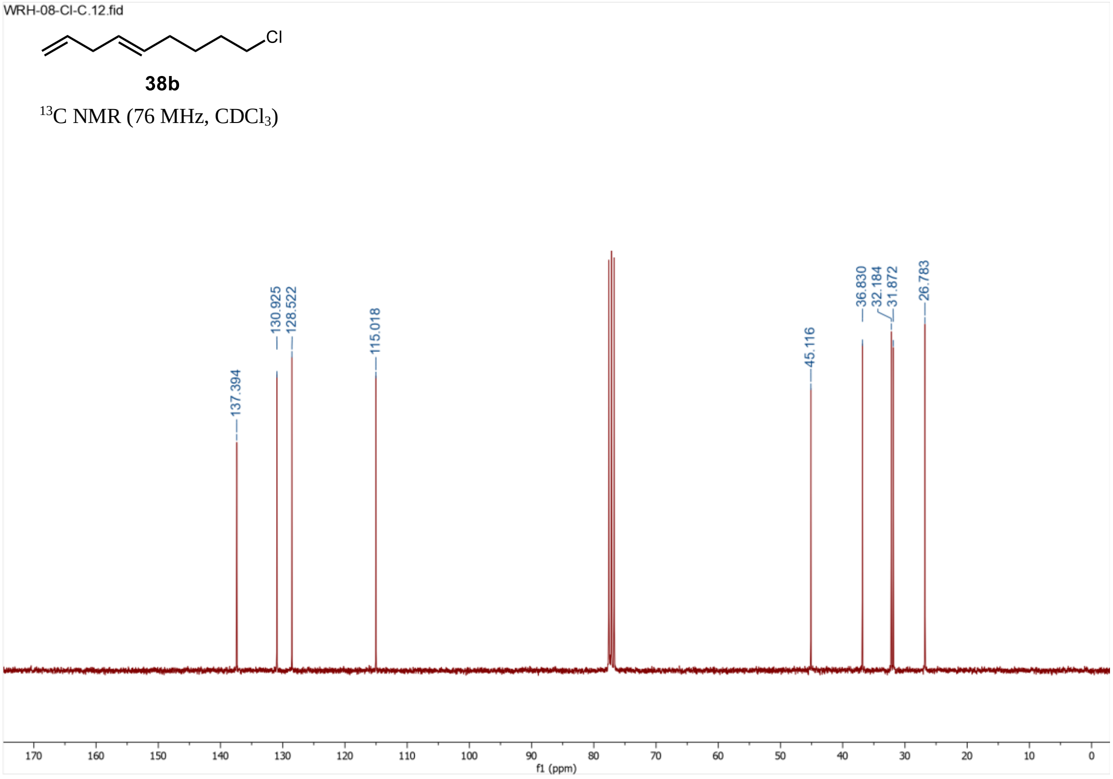
WRH-08-Cl-C.12.fid
Cl
38b
^{13}C NMR (76 MHz, CDCl$_3$)
137.394
130.925
128.522
115.018
45.116
36.830
32.184
31.872
26.783
f1 (ppm)

WRH-08-PhO.20.fid

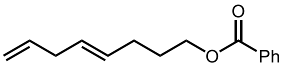

42b

^{1}H NMR (300 MHz, $CDCl_3$)

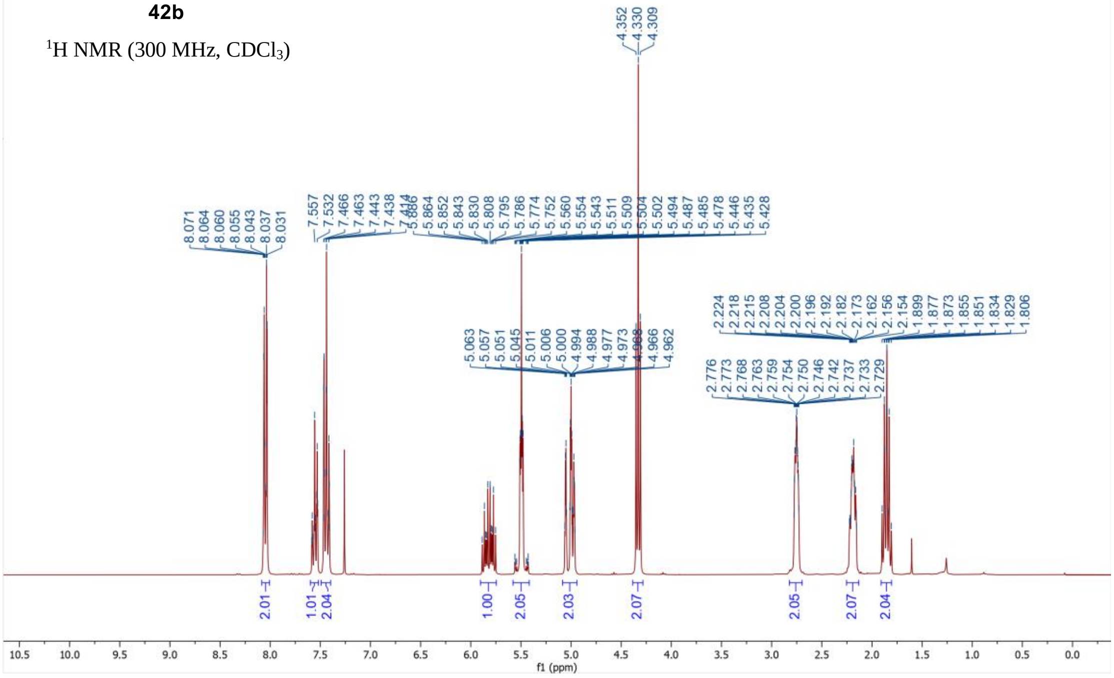

WRH-08-PhO-C.12.fid

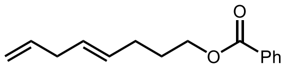

42b

^{13}C NMR (76 MHz, $CDCl_3$)

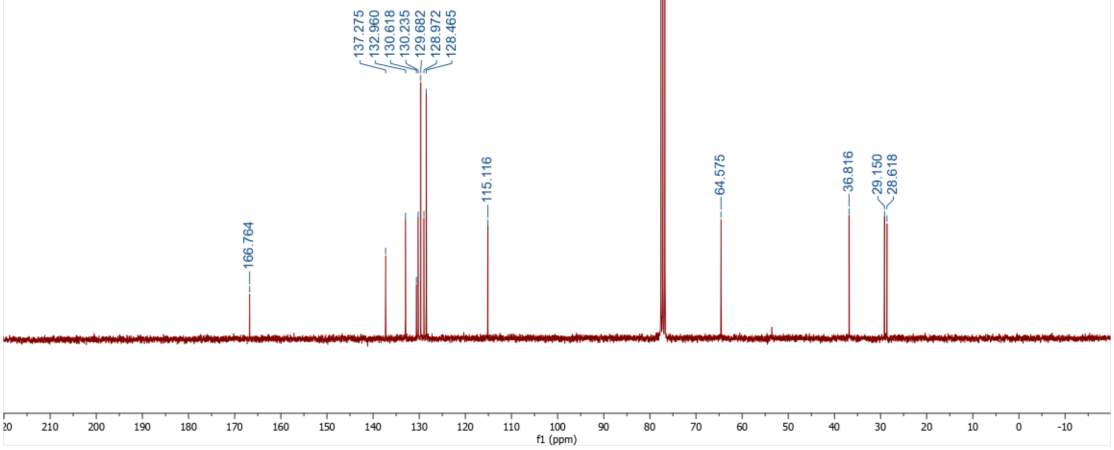

Cartesian coordinates (Å) for DFT calculations

TMPH

C	-1.2745470	-0.2636450	-0.0400110
C	1.2752580	-0.2610810	-0.0402630
H	0.0010780	-1.1159220	-1.3567460
C	-1.2486300	1.2190630	-0.4538530
C	1.2462790	1.2215800	-0.4540760
C	-0.0018540	1.9372620	0.0347010
H	-2.1631910	1.7255870	-0.1013560
H	-1.2780290	1.2722480	-1.5579980
H	2.1598830	1.7299370	-0.1017280
H	1.2753850	1.2748340	-1.5582270
H	-0.0029380	2.9827790	-0.3106510
H	-0.0018080	1.9972640	1.1372670
C	2.3495080	-0.9932680	-0.8352540
H	2.3954190	-2.0529530	-0.5465390
H	2.1467870	-0.9473470	-1.9167750
H	3.3402020	-0.5475210	-0.6681180
C	1.6142040	-0.3947570	1.4453210
H	1.0012760	0.2462650	2.0909520
H	1.4763540	-1.4326790	1.7783790
H	2.6622180	-0.1123020	1.6234610
C	-2.3474920	-0.9980030	-0.8347700
H	-2.1451050	-0.9516810	-1.9163330
H	-2.3911990	-2.0577770	-0.5460290
H	-3.3390500	-0.5542660	-0.6674200
C	-1.6129410	-0.3979390	1.4456450
H	-2.6615480	-0.1178350	1.6239570
H	-1.4726660	-1.4354810	1.7788020
H	-1.0013860	0.2445210	2.0911030
N	0.0010010	-0.9382230	-0.3533450

Diene

C	-2.9666750	1.3465930	-1.8147360
H	-4.0105280	1.0299050	-1.7616020
H	-2.6777790	1.9399580	-2.6878570
C	-2.0868910	1.0257180	-0.8658350
H	-2.4267210	0.4346260	-0.0047050
C	-0.6402180	1.4116320	-0.8582220
H	-0.3961030	2.0024510	-1.7550330
H	-0.0325830	0.4883290	-0.9244960
C	-0.2389800	2.1524630	0.3795310
H	-0.4467640	1.6410530	1.3291450
C	0.3301410	3.3577380	0.4077450
H	0.5502120	3.9098000	-0.5112870
H	0.6019320	3.8431640	1.3475370

Pentene

C	0.66754200	1.06795000	-0.04947600
H	1.29346300	1.96181800	-0.10544700
C	-0.67063200	1.06614200	-0.04907100
H	-1.29870000	1.95832300	-0.10494000
C	1.22412200	-0.31563200	0.10577700
H	2.05810100	-0.53129000	-0.58153500
H	1.64412900	-0.44853500	1.12058200
C	-1.22319100	-0.31884700	0.10569800
H	-1.64360100	-0.45377100	1.12017900
H	-2.05605100	-0.53697000	-0.58254300
C	0.00172100	-1.21268200	-0.13836700
H	0.00214600	-1.54573000	-1.18792200
H	0.00314600	-2.12543600	0.47425400

Allylbenzene

C	-2.41802100	-0.08780300	0.48665500
H	-2.23770900	-0.00595700	1.56673400
C	-3.48309900	0.51908700	-0.03956300
H	-4.18801200	1.08207700	0.57607800

C	-1.41065300	-0.88478700	-0.27690300
H	-1.44881500	-1.93747900	0.05178600
H	-3.69728200	0.47224600	-1.11219500
C	0.00737000	-0.38844500	-0.13196700
C	0.29156600	0.98318700	-0.15639900
C	1.07390100	-1.28469000	0.00677500
C	1.60233000	1.44413700	-0.05175600
H	-0.53197500	1.69646500	-0.25736900
C	2.38671800	-0.82751300	0.11051100
H	0.86959300	-2.35935100	0.03445800
C	2.65584600	0.54001700	0.08144500
H	1.80275500	2.51820200	-0.07277500
H	3.20408000	-1.54459800	0.21920800
H	3.68359900	0.90060100	0.16593000
H	-1.69198000	-0.90134800	-1.34464900

$TMPH_2^+$

C	-1.3504250	-0.2224070	-0.0311730
C	1.3505340	-0.2207670	-0.0305530
H	0.0008250	-0.9585810	-1.4332120
C	-1.2510970	1.2463010	-0.4385920
C	1.2496070	1.2478210	-0.4380160
C	-0.0012850	1.9418100	0.0726710
H	-2.1635110	1.7498560	-0.0873440
H	-1.2831370	1.3135920	-1.5402850
H	2.1612490	1.7524870	-0.0863580
H	1.2820660	1.3151470	-1.5396950
H	-0.0018450	2.9882510	-0.2598940
H	-0.0015650	1.9914550	1.1733450
C	2.3948700	-0.9605110	-0.8501170
H	2.4698670	-2.0207160	-0.5682250
H	2.1989260	-0.8964320	-1.9304440
H	3.3808430	-0.5116680	-0.6778800
C	1.6158700	-0.4110200	1.4528280
H	1.0070440	0.2304570	2.0977450
H	1.4772420	-1.4546490	1.7700970
H	2.6645460	-0.1573140	1.6522980
C	-2.3934720	-0.9634230	-0.8512320
H	-2.1970270	-0.8992070	-1.9314520
H	-2.4674000	-2.0236880	-0.5693010
H	-3.3800480	-0.5156970	-0.6795670
C	-1.6162430	-0.4129640	1.4520930
H	-2.6654170	-0.1610850	1.6509770
H	-1.4759800	-1.4562520	1.7695760
H	-1.0089360	0.2296300	2.0972670
N	0.0005420	-0.8769470	-0.4095430
H	0.0010510	-1.8457300	-0.0687840

OTf^-

O	2.3772610	0.0715560	0.0598540
S	3.8478080	0.0292940	0.1407460
O	4.5148650	-0.9159580	-0.7717220
O	4.5192020	1.3281580	0.3232640
C	4.1180770	-0.7792260	1.7975810
F	3.5738150	-2.0084460	1.8555020
F	5.4249290	-0.9205150	2.0851250
F	3.5751640	-0.0677820	2.8023960

TMSOTf

S	-0.7802230	-0.8457670	0.1331370
O	-0.4242610	-1.3231750	-1.1954060
O	-1.3971430	-1.7005210	1.1210080
C	-1.9335650	0.5861000	-0.1168630
F	-1.3594110	1.4948400	-0.9003760
F	-3.0390550	0.1476100	-0.6987650
F	-2.2370500	1.1431700	1.0437590
O	0.4217350	-0.0533670	0.7725740
Si	2.0496820	0.1721260	0.0598510
C	1.8247520	1.1393830	-1.5258250
H	2.7917800	1.2967660	-2.0252470
H	1.1705760	0.6002510	-2.2245150
H	1.3823260	2.1276660	-1.3400650

C	2.7447600	-1.5440330	-0.2049450
H	3.7828650	-1.5011870	-0.5651430
H	2.7433620	-2.1241920	0.7286400
H	2.1531660	-2.0963060	-0.9476880
C	2.8924780	1.1329100	1.4231820
H	3.9345850	1.3690060	1.1645700
H	2.3745990	2.0821760	1.6177560
H	2.9022660	0.5608820	2.3610650

13a

C	1.6321640	-0.6870780	0.0013040
C	0.7728130	0.4140880	0.0006310
C	1.3062820	1.7029750	-0.0057120
C	2.7001060	1.8757820	-0.0094720
C	3.5290570	0.7433980	-0.0120580
C	3.0074500	-0.5529740	-0.0059930
C	-0.6333920	-0.0513220	0.0024070
H	0.6408810	2.5669490	-0.0103690
H	4.6136040	0.8806290	-0.0219960
H	3.6635580	-1.4255620	-0.0104020
C	3.2857470	3.2516710	0.0129320
H	2.6842390	3.9593930	-0.5718860
H	3.3256370	3.6471510	1.0396230
H	4.3109120	3.2692510	-0.3770950
C	-1.7861100	0.9145860	0.0022370
O	-1.6404860	2.1156480	0.0039680
O	-2.9553880	0.2932770	0.0000770
C	-4.1174950	1.1413780	0.0001110
H	-4.0690630	1.7982480	0.8828200
H	-4.0671590	1.8009430	-0.8804820
C	-5.3303560	0.2612620	-0.0025330
H	-6.2415890	0.8720020	-0.0025460
H	-5.3512460	-0.3828920	-0.8906870
H	-5.3531010	-0.3856410	0.8835750
S	0.6412990	-2.1699210	0.0048970
O	0.7597070	-2.8700890	1.2727360
O	0.7564180	-2.8744550	-1.2608320
N	-0.8572510	-1.3185580	0.0053430

13-TMS

C	0.6853820	-1.7319390	-0.7556800
C	1.1542330	-0.6022350	-0.1064840
C	2.5278990	-0.4933100	0.1271040
C	3.4013150	-1.5018880	-0.2927460
C	2.8801890	-2.6297050	-0.9535970
C	1.5182830	-2.7594300	-1.1881210
C	0.0588620	0.3719210	0.2928030
H	2.9348440	0.3882690	0.6233560
H	3.5630630	-3.4161050	-1.2864870
H	1.1050100	-3.6344640	-1.6939270
C	4.8725430	-1.3900900	-0.0446510
H	5.1409960	-0.4238090	0.4000050
H	5.2256230	-2.1767950	0.6388820
H	5.4502960	-1.5047290	-0.9731950
C	0.2518370	1.7765100	-0.2966690
O	-0.6302310	2.6013040	-0.3103610
O	1.4825040	2.0060180	-0.7466190
C	1.7383570	3.3238490	-1.2665420
H	1.0520910	3.5049110	-2.1076590
H	1.4825840	4.0614240	-0.4895540
C	3.1803710	3.3809660	-1.6725320
H	3.4211900	4.3672860	-2.0879000
H	3.8438660	3.2044100	-0.8152560
H	3.4069460	2.6259490	-2.4365750
S	-1.0690630	-1.7284780	-0.8571790
O	-1.5968150	-2.7409490	0.0567960
O	-1.5532770	-1.7528390	-2.2382000
N	-1.1980350	-0.1673250	-0.2373800
C	-0.0436940	0.5277920	1.8580960
C	-0.4315090	-0.7789230	2.4781850
C	-1.4187240	-0.9353810	3.3590620
H	0.1513550	-1.6562800	2.1683870
H	-2.0246600	-0.0900780	3.7011850

H	-0.8691750	1.2481770	2.0057810
H	-1.6611210	-1.9142050	3.7779060
C	1.1820150	1.1569200	2.4573250
C	1.8494060	0.7196630	3.5262900
H	1.5232390	2.0806440	1.9688480
H	1.5475800	-0.1850030	4.0615050
H	2.7172750	1.2577880	3.9131320
Si	-2.8673240	0.5945050	-0.3456100
C	-4.0704480	-0.8559590	-0.2844310
H	-3.9494850	-1.5490290	-1.1276980
H	-3.9535360	-1.4396650	0.6394260
H	-5.1005770	-0.4687140	-0.3155190
C	-3.0327620	1.4862650	-1.9935060
H	-2.4349840	2.4053430	-2.0372410
H	-2.7092110	0.8188780	-2.8054050
H	-4.0829400	1.7545780	-2.1827580
C	-3.2489600	1.6963370	1.1344260
H	-3.1998540	1.1381370	2.0802080
H	-2.6046640	2.5810240	1.2025010
H	-4.2870080	2.0494600	1.0302050

BiPh$_2$OTf

Bi	0.70967100	-0.05315700	-1.24554700
C	-0.09146800	1.75242000	-0.22155800
C	-1.20771600	2.38785800	-0.78504900
H	-1.66124300	2.00753400	-1.70462100
C	-1.77965200	3.49305400	-0.15706200
H	-2.65691200	3.97360200	-0.59660400
C	-1.23897700	3.97641900	1.03394500
H	-1.69122300	4.83921100	1.52883400
C	0.45478200	2.25372400	0.96679800
C	-0.11940400	3.36023700	1.59209600
H	0.30921400	3.74069900	2.52250700
C	3.65093300	-0.29716200	-0.10209100
C	4.66112300	-0.52537300	0.83414200
H	5.70854800	-0.41303000	0.54386200
C	4.33068600	-0.90293800	2.13428500
H	5.11987400	-1.08728400	2.86690400
C	2.99164000	-1.04995600	2.49827000
H	2.73089100	-1.35115400	3.51582100
C	1.98049000	-0.81540400	1.56706500
H	0.93333000	-0.93678000	1.86017500
C	2.30500500	-0.43405400	0.25803200
O	-0.52053600	-1.43722100	-0.06825700
S	-2.05276500	-1.49061300	-0.32440900
O	-2.41804300	-0.47182600	-1.31005200
O	-2.53719300	-2.85056700	-0.43405400
C	-2.66203400	-0.88568100	1.32385300
F	-2.23085800	0.34632900	1.56474400
F	-3.98765000	-0.88939300	1.32513400
F	-2.22166700	-1.68825800	2.28546500
H	3.93371700	-0.01072200	-1.12141000
H	1.33065500	1.77967900	1.41996600

Int-1

Bi	0.0607710	0.3100720	-0.5880800
C	0.2039040	-0.5322330	1.4825350
C	0.7243120	-1.8226010	1.6482940
H	1.1282040	-2.3697450	0.7910360
C	0.7639610	-2.4100150	2.9130830
H	1.1818140	-3.4129800	3.0310870
C	0.2762120	-1.7195770	4.0220090
H	0.3014780	-2.1830260	5.0112120
C	-0.2579210	0.1646060	2.6046970
C	-0.2292450	-0.4292000	3.8675810
H	-0.5948380	0.1247140	4.7359980
C	-0.9551730	3.0892570	0.8093600
C	-0.8699040	4.4325060	1.1798460
H	-1.6942060	4.8965160	1.7276210
C	0.2624730	5.1759950	0.8537170
H	0.3315610	6.2273740	1.1427590
C	1.3058490	4.5688090	0.1574090
H	2.1997310	5.1427100	-0.0998740

C	1.2215340	3.2257830	-0.2137140
H	2.0589420	2.7667660	-0.7447490
C	0.0889860	2.4651860	0.1118210
O	2.3300300	0.4731180	-0.6718800
S	3.0589930	-0.7205430	-1.3116730
O	2.1800190	-1.8991040	-1.3311910
O	3.8123440	-0.3643340	-2.4998770
C	4.3349190	-1.0607890	-0.0092310
F	3.7570030	-1.3538010	1.1512160
F	5.0792420	-2.0938880	-0.3887290
F	5.1169130	-0.0021250	0.1546620
H	-1.8590860	2.5360930	1.0808720
H	-0.6365740	1.1870210	2.5090090
C	-2.9322070	-2.0394110	-0.3665120
C	-3.8019560	0.3625540	-0.5997660
H	-2.5350150	-0.6650590	-1.7820870
C	-4.2547150	-2.5345040	-0.9744950
C	-5.1169150	-0.2027750	-1.1640870
C	-5.4212610	-1.6086560	-0.6799150
H	-4.4572380	-3.5565320	-0.6152200
H	-4.1278220	-2.6189250	-2.0696670
H	-5.9408770	0.4898690	-0.9261430
H	-5.0424990	-0.2138200	-2.2668330
H	-6.3369470	-1.9834330	-1.1613020
H	-5.6450630	-1.6049580	0.4004240
C	-3.4090080	1.5927850	-1.4082120
H	-2.4938870	2.0667500	-1.0244620
H	-3.2415140	1.3398060	-2.4668920
H	-4.2038790	2.3506950	-1.3777310
C	-3.9550930	0.7435600	0.8727350
H	-4.5813710	0.0421750	1.4371460
H	-2.9776220	0.7772870	1.3760890
H	-4.4243020	1.7333040	0.9656190
C	-1.7749510	-2.8487550	-0.9393310
H	-1.6591820	-2.6902180	-2.0229960
H	-0.8192640	-2.6008620	-0.4555870
H	-1.9430660	-3.9232490	-0.7842000
C	-2.9471700	-2.2064520	1.1508470
H	-2.8267770	-3.2700570	1.4008690
H	-2.1229570	-1.6617670	1.6270940
H	-3.8836750	-1.8779510	1.6163820
N	-2.6913240	-0.6173330	-0.7715520

Int-2

Bi	0.3967140	0.1566330	-1.0657050
C	2.1237670	-0.8761160	-0.1238860
C	2.4329520	-2.1696870	-0.5754290
H	1.7974090	-2.6791630	-1.3083390
C	3.5648270	-2.8305950	-0.1016240
H	3.7943260	-3.8382150	-0.4545770
C	4.4066300	-2.1987860	0.8137190
H	5.2946270	-2.7151370	1.1841030
C	2.9894660	-0.2393450	0.7778270
C	4.1243880	-0.9020390	1.2435750
H	4.7950470	-0.3992490	1.9437430
C	0.5385690	2.0255060	1.5285930
C	0.4956450	3.2237410	2.2423050
H	0.5261160	3.2076100	3.3340570
C	0.4233460	4.4389150	1.5611860
H	0.3925730	5.3765030	2.1197970
C	0.3927140	4.4563950	0.1674510
H	0.3393080	5.4068070	-0.3677910
C	0.4230530	3.2585770	-0.5477710
H	0.3812100	3.2976990	-1.6416890
C	0.4992210	2.0327400	0.1264550
H	0.6165830	1.0821280	2.0804830
H	2.7916500	0.7807640	1.1182750
C	-2.7904800	-0.1047430	-0.3754430
C	-1.4312340	-1.7547030	1.1571960
H	-1.4089580	0.2963620	1.0829880
C	-3.9896200	-0.3654860	0.5447230
C	-2.7366980	-1.9608310	1.9427590
C	-3.9993040	-1.7572080	1.1373040
H	-4.9042780	-0.1629300	-0.0327840

H	-3.9794260	0.3795650	1.3600530
H	-2.6967880	-2.9655920	2.3902510
H	-2.7418220	-1.2576670	2.7938940
H	-4.8799490	-1.8890780	1.7809780
H	-4.0992110	-2.5225510	0.3504230
C	-0.3037320	-1.6292970	2.1728850
H	0.6894660	-1.5530130	1.7158460
H	-0.4569000	-0.7665120	2.8386540
H	-0.2953320	-2.5202910	2.8141250
C	-1.1584960	-2.9177770	0.2155690
H	-2.0194060	-3.1948160	-0.4029440
H	-0.3108380	-2.7093130	-0.4544080
H	-0.8846970	-3.8064460	0.7990270
C	-2.7450670	1.3830690	-0.7051040
H	-2.5125160	2.0006160	0.1758970
H	-2.0226300	1.6323950	-1.4965050
H	-3.7275220	1.7021110	-1.0770580
C	-2.9255870	-0.9071560	-1.6633550
H	-3.7574710	-0.4952780	-2.2496330
H	-2.0322320	-0.8458320	-2.3008020
H	-3.1491830	-1.9667080	-1.5020420
N	-1.4960790	-0.4437630	0.3751280

Int-3

Bi	-0.3574090	0.0596570	-0.4267110
C	-0.2383290	2.2407210	0.0372940
C	0.0092710	3.1169430	-1.0295720
H	0.2094960	2.7367140	-2.0377640
C	-0.0015860	4.4972880	-0.8286750
H	0.1979580	5.1702030	-1.6654530
C	-0.2756240	5.0138260	0.4369820
H	-0.2850090	6.0941010	0.5957560
C	-0.5391230	2.7692360	1.3000170
C	-0.5539250	4.1500170	1.4965920
H	-0.7893290	4.5532870	2.4840820
C	0.2698640	-0.2104700	2.6998480
C	0.2144220	-0.7822230	3.9724870
H	0.8636780	-0.4049720	4.7658440
C	-0.6715400	-1.8273660	4.2302080
H	-0.7162730	-2.2734300	5.2257670
C	-1.4993090	-2.3035620	3.2138840
H	-2.1919570	-3.1247560	3.4108670
C	-1.4382500	-1.7374590	1.9403430
H	-2.0809250	-2.1458470	1.1526200
C	-0.5585900	-0.6807920	1.6713310
H	0.9651610	0.6163920	2.5202270
H	-0.7775210	2.1103260	2.1399840
C	2.1791960	-2.0468520	-0.8302490
C	3.1461890	0.3421580	-0.3809780
H	2.0406310	-0.8746290	0.8378050
C	3.5968590	-2.5854190	-0.5970210
C	4.5214470	-0.3289910	-0.2322760
C	4.6805740	-1.6145020	-1.0122620
H	3.6865670	-3.5452180	-1.1284090
H	3.7122790	-2.8243960	0.4752810
H	5.2870520	0.4118180	-0.5094660
H	4.6879810	-0.5413140	0.8384140
H	5.6722980	-2.0486670	-0.8236670
H	4.6486370	-1.4277680	-2.0983000
C	3.0255440	1.3853400	0.7217960
H	2.1007350	1.9717750	0.6642360
H	3.0920240	0.9237420	1.7186450
H	3.8616260	2.0929670	0.6471840
C	3.0003330	1.0087340	-1.7415990
H	1.9679920	1.3452740	-1.9189980
H	3.6359060	1.9029440	-1.7855800
H	3.2948250	0.3669670	-2.5795800
C	1.1876930	-2.9835440	-0.1484340
H	1.2750330	-2.9573410	0.9486250
H	0.1399530	-2.7729760	-0.4120630
H	1.3819710	-4.0161680	-0.4672480
C	1.8744990	-1.9916350	-2.3228960
H	1.7956270	-3.0171270	-2.7074910
H	0.9182800	-1.4965340	-2.5435290

H	2.6512340	-1.4940630	-2.9128210
N	2.0365110	-0.6773470	-0.1699190
C	-3.6384350	-0.1440010	-0.2680240
H	-3.8504830	-0.4237670	0.7733300
C	-3.2906880	1.1274050	-0.5253080
H	-3.2246790	1.8780410	0.2661040
H	-3.1244610	1.4819970	-1.5492240
C	-3.8230980	-1.2202900	-1.2908240
H	-3.5646120	-0.8453340	-2.2941930
H	-3.1172940	-2.0489030	-1.0770310
C	-5.2193730	-1.7695600	-1.2781860
C	-6.0541040	-1.7425920	-2.3161280
H	-5.5517580	-2.2109110	-0.3302190
H	-7.0603660	-2.1601460	-2.2499380
H	-5.7663950	-1.3068380	-3.2774440

Int-3$_{TMPH}$

C	1.3203010	1.9745890	-0.6229110
H	1.4640710	1.8916360	0.4592120
H	0.9987650	2.9477180	-1.0022760
C	1.6224500	0.9539110	-1.4493290
H	1.5077440	1.1161370	-2.5317010
C	2.1477410	-0.3754390	-1.0462670
H	1.9690110	-0.5641040	0.0262370
H	3.2715140	-0.3439100	-1.1028080
N	5.2757810	-0.5805790	-0.5448030
C	6.1346900	0.4054810	-1.2433760
C	5.3962920	-0.7432200	0.9234870
C	7.5929150	0.2953280	-0.7692990
C	6.0413810	0.0647180	-2.7257750
C	5.6081770	1.8274490	-1.0479450
C	6.8727410	-0.8245860	1.3460310
C	4.6873620	-2.0487730	1.2671120
C	4.6863790	0.3902080	1.6662040
C	7.7143420	0.2891610	0.7454970
H	8.1927140	1.1070050	-1.2125290
H	8.0173540	-0.6452210	-1.1650560
H	5.0084800	0.1662470	-3.0925600
H	6.6782220	0.7302730	-3.3242470
H	6.3694000	-0.9672150	-2.9238590
H	4.5217590	1.8730310	-1.2132830
H	5.8107560	2.2361530	-0.0508290
H	6.0864600	2.5067250	-1.7676840
H	7.2760520	-1.7973060	1.0104510
H	6.9471870	-0.8303480	2.4460540
H	3.6164280	-1.9999500	1.0180830
H	5.1219860	-2.9001580	0.7209450
H	4.7734400	-2.2707550	2.3401910
H	3.6841040	0.5691870	1.2494930
H	4.5671480	0.1249890	2.7269250
H	5.2323010	1.3405580	1.6392660
H	7.4100370	1.2661800	1.1592960
H	8.7671340	0.1691370	1.0410790
H	5.4873160	-1.4908780	-0.9546590
Bi	-1.3050020	0.3104900	-0.5686790
C	-0.3986850	-0.4921840	1.3103050
C	-0.3357560	-1.8866790	1.4428460
C	0.2136770	0.3031100	2.2880040
C	0.3085570	-2.4751320	2.5317650
H	-0.7639180	-2.5423680	0.6748960
C	0.8624380	-0.2841330	3.3754180
H	0.2039240	1.3936210	2.2101690
C	0.9102910	-1.6721310	3.4996520
H	0.3503260	-3.5635760	2.6170480
H	1.3375620	0.3496360	4.1278100
H	1.4220610	-2.1290660	4.3492210
C	-1.9895890	2.3300560	0.1183800
C	-2.3624440	3.1764740	-0.9375770
C	-2.0557680	2.8157660	1.4302420
C	-2.7997080	4.4772640	-0.6883320
H	-2.3190560	2.8283790	-1.9771730
C	-2.4926760	4.1170540	1.6789920
H	-1.7754100	2.1868260	2.2794430
C	-2.8671640	4.9471110	0.6224050

H	-3.0890720	5.1235740	-1.5198630
H	-2.5417010	4.4840160	2.7067050
H	-3.2114950	5.9639840	0.8218210
N	-3.3020950	-1.1597580	-0.0521960
C	-4.3903530	-0.6458010	0.8787930
C	-3.7226380	-1.9053950	-1.3075120
C	-5.4175270	-1.7602560	1.1246110
C	-3.7052130	-0.3123570	2.1949420
C	-5.0709070	0.5987080	0.3215780
C	-4.8051060	-2.9393650	-0.9609530
C	-2.5027490	-2.6606130	-1.8261690
C	-4.2105490	-0.9350530	-2.3758520
C	-5.9530580	-2.3793650	-0.1489580
H	-6.2274800	-1.3449510	1.7438100
H	-4.9442630	-2.5471380	1.7390160
H	-3.0133010	0.5323470	2.0948690
H	-4.4558920	-0.0329460	2.9458700
H	-3.1413760	-1.1690750	2.5951970
H	-4.3522330	1.3272340	-0.0749870
H	-5.8040430	0.3808660	-0.4630750
H	-5.6186370	1.1000490	1.1308230
H	-4.3309300	-3.7597810	-0.3929390
H	-5.1571490	-3.3931600	-1.9002250
H	-1.7175880	-2.0076460	-2.2333850
H	-2.0517860	-3.2963090	-1.0478520
H	-2.8055930	-3.3306590	-2.6419060
H	-3.5098210	-0.0991920	-2.5276040
H	-4.2930790	-1.4599950	-3.3367380
H	-5.1950920	-0.5063260	-2.1608040
H	-6.5260730	-1.6420070	-0.7349610
H	-6.6699600	-3.1777140	0.0889130
H	-2.8049820	-1.8563480	0.5153530
C	1.6573250	-1.5095800	-1.8828580
H	1.5507360	-1.2998940	-2.9570630
C	1.3968340	-2.7456160	-1.4448270
H	1.0961610	-3.5433720	-2.1280300
H	1.5151240	-3.0167060	-0.3907000

TS-1

C	1.2217890	1.2024600	-1.0093130
H	1.3503000	1.4061920	0.0606160
H	1.0379100	2.0855060	-1.6305430
C	1.8379180	0.0913410	-1.5691040
H	1.8545920	0.0382560	-2.6695720
C	2.4495880	-0.9922240	-0.8839860
H	2.1762880	-1.0677750	0.1809160
H	3.7149590	-0.6216770	-0.6568250
N	5.0808340	-0.4892930	-0.3034050
C	5.7658300	0.4635120	-1.2409360
C	5.2468630	-0.3353860	1.1804220
C	7.2329520	0.6145820	-0.8232230
C	5.6661860	-0.1731580	-2.6218290
C	5.0582930	1.8159840	-1.2643040
C	6.7321150	-0.1274950	1.5059310
C	4.7598670	-1.6372080	1.8059040
C	4.3886870	0.8140160	1.7008980
C	7.3938870	0.9388070	0.6516310
H	7.7053250	1.3820440	-1.4562420
H	7.7629010	-0.3285720	-1.0469830
H	4.6230330	-0.2458140	-2.9616840
H	6.2133500	0.4281080	-3.3594750
H	6.1002250	-1.1843720	-2.6379970
H	3.9667830	1.7089870	-1.3316130
H	5.2808980	2.4397760	-0.3913500
H	5.3896380	2.3810100	-2.1457740
H	7.2581730	-1.0868040	1.3535070
H	6.8310530	0.1002210	2.5788910
H	3.6837440	-1.7937270	1.6474790
H	5.2948470	-2.5097100	1.4012440
H	4.9352440	-1.6222990	2.8900390
H	3.3597900	0.7525070	1.3163160
H	4.3343270	0.7594550	2.7968000
H	4.7837010	1.8052320	1.4520330
H	6.9733160	1.9329160	0.8780500

H	8.4604240	1.0144120	0.9067100
H	5.4255180	-1.4235700	-0.5383080
Bi	-1.2322320	0.3000120	-0.7463290
C	-0.3854910	-0.8292930	0.9924810
C	-0.4931930	-2.2257450	0.9561820
C	0.3361720	-0.2421890	2.0403560
C	0.0808130	-3.0176690	1.9529240
H	-1.0108300	-2.7241410	0.1272150
C	0.9075620	-1.0300330	3.0408590
H	0.4755760	0.8425370	2.0850380
C	0.7771170	-2.4189710	3.0014470
H	-0.0102910	-4.1054190	1.9038250
H	1.4603870	-0.5551750	3.8559290
H	1.2239600	-3.0345060	3.7854550
C	-1.6470010	2.3104890	0.1576170
C	-2.0413470	3.2737850	-0.7830880
C	-1.5777860	2.6769450	1.5070320
C	-2.3687670	4.5701960	-0.3851250
H	-2.1078300	3.0211880	-1.8487530
C	-1.9060510	3.9732670	1.9058820
H	-1.2824790	1.9549660	2.2728150
C	-2.3049240	4.9197830	0.9626720
H	-2.6785190	5.3066330	-1.1300930
H	-1.8532770	4.2434160	2.9632170
H	-2.5662480	5.9317670	1.2790940
N	-3.5532340	-0.9651950	-0.0238420
C	-4.4036930	-0.3942600	1.0833970
C	-4.2444590	-1.4952810	-1.2513200
C	-5.5776490	-1.3374960	1.3875570
C	-3.5070570	-0.3104210	2.3099090
C	-4.9183880	1.0008370	0.7403170
C	-5.4478920	-2.3696200	-0.8601070
C	-3.2461690	-2.3788400	-1.9925780
C	-4.6741310	-0.3528740	-2.1658990
C	-6.3679900	-1.7210680	0.1531330
H	-6.2202820	-0.8626990	2.1455990
H	-5.1800170	-2.2535600	1.8608520
H	-2.6936940	0.4116270	2.1661920
H	-4.0880630	0.0143780	3.1835410
H	-3.0547200	-1.2840370	2.5564360
H	-4.1358400	1.6336000	0.3023780
H	-5.7725230	0.9937340	0.0533830
H	-5.2603310	1.4980650	1.6585600
H	-5.0642950	-3.3160850	-0.4380230
H	-5.9906240	-2.6500250	-1.7766680
H	-2.4067050	-1.8132950	-2.4216150
H	-2.8373390	-3.1658510	-1.3395280
H	-3.7445760	-2.8885390	-2.8281920
H	-3.8553720	0.3637880	-2.3342380
H	-4.9647200	-0.7515010	-3.1473640
H	-5.5321300	0.2116140	-1.7846490
H	-6.8676930	-0.8391000	-0.2811920
H	-7.1817450	-2.4106670	0.4197620
H	-3.0939950	-1.7766280	0.4020030
C	2.6275060	-2.2850900	-1.5647200
H	2.6715770	-2.2457530	-2.6624520
C	2.7931880	-3.4693340	-0.9592850
H	2.9572820	-4.3852920	-1.5293340
H	2.7449910	-3.5689810	0.1302260

Int-4$_{TMPH2+}$

C	1.1522860	0.9131630	-1.2425070
H	1.4373660	1.1972080	-0.2181610
H	1.1498060	1.7937620	-1.8994370
C	1.8450680	-0.2385010	-1.7746130
H	1.9207830	-0.3060180	-2.8712530
C	2.3377910	-1.3021660	-1.0685030
H	2.2124540	-1.3058430	0.0254820
H	4.1503540	-0.5891360	-0.4545060
N	5.1410920	-0.4731140	-0.1501560
C	5.8171880	0.5041370	-1.1200790
C	5.1768580	-0.2397640	1.3634300
C	7.2256240	0.7644490	-0.5907340
C	5.8412020	-0.2184060	-2.4580690

C	4.9870220	1.7744240	-1.2359440
C	6.6224790	0.0852550	1.7382230
C	4.7268420	-1.5486240	1.9938500
C	4.1984940	0.8651560	1.7310820
C	7.2536790	1.1636190	0.8748130
H	7.6879870	1.5345300	-1.2257690
H	7.8352670	-0.1451790	-0.7344480
H	4.8250050	-0.3946110	-2.8394160
H	6.3727360	0.3925760	-3.1982560
H	6.3615920	-1.1857440	-2.3984570
H	3.9153960	1.5558140	-1.3558680
H	5.1071910	2.4572740	-0.3883340
H	5.3087340	2.3216910	-2.1310430
H	7.2224290	-0.8388190	1.6622510
H	6.6400800	0.3617450	2.8027860
H	3.6776230	-1.7793290	1.7580250
H	5.3525380	-2.3973130	1.6799820
H	4.8059680	-1.4737410	3.0861280
H	3.2109590	0.6954670	1.2747690
H	4.0548800	0.8599190	2.8192850
H	4.5388010	1.8693930	1.4581680
H	6.7462130	2.1302590	1.0254770
H	8.2911980	1.3317340	1.1938300
H	5.5520780	-1.3985850	-0.3220940
Bi	-1.1477820	0.3053900	-0.9083470
C	-0.3806840	-0.8964560	0.8298190
C	-0.5819890	-2.2822020	0.7953960
C	0.4070790	-0.3665350	1.8605720
C	-0.0222560	-3.1181390	1.7646970
H	-1.1709110	-2.7350830	-0.0113140
C	0.9551280	-1.1948680	2.8420610
H	0.6122360	0.7086520	1.9064610
C	0.7431970	-2.5740800	2.7950250
H	-0.1852170	-4.1974810	1.7140160
H	1.5497720	-0.7608840	3.6518040
H	1.1708810	-3.2231050	3.5630330
C	-1.5284660	2.2930000	0.0688800
C	-1.9484650	3.2998410	-0.8126600
C	-1.4429820	2.5939540	1.4335250
C	-2.2795390	4.5721120	-0.3448060
H	-2.0369250	3.0991870	-1.8876270
C	-1.7761320	3.8650250	1.9045240
H	-1.1362010	1.8337300	2.1570780
C	-2.1973330	4.8550330	1.0176460
H	-2.6103750	5.3415490	-1.0464840
H	-1.7131010	4.0800890	2.9742050
H	-2.4647400	5.8467300	1.3890390
N	-3.7607650	-0.9497250	0.0906530
C	-4.4813250	-0.3250640	1.2412410
C	-4.5572250	-1.4211430	-1.0788960
C	-5.7174270	-1.1536160	1.6271870
C	-3.4959620	-0.3210910	2.4018200
C	-4.8840970	1.1166080	0.9346400
C	-5.8085750	-2.1942620	-0.6263930
C	-3.6643200	-2.3710970	-1.8692320
C	-4.9406860	-0.2484850	-1.9785720
C	-6.6069820	-1.4693370	0.4396860
H	-6.2757310	-0.6241900	2.4161540
H	-5.3764330	-2.1017260	2.0819580
H	-2.6240110	0.3075960	2.1772680
H	-3.9694540	0.0710410	3.3124770
H	-3.1284960	-1.3348650	2.6288720
H	-4.0670250	1.6700950	0.4531330
H	-5.7689550	1.1944670	0.2916680
H	-5.1301830	1.6395340	1.8698430
H	-5.4842090	-3.1710560	-0.2233430
H	-6.4305130	-2.4244970	-1.5066550
H	-2.7857650	-1.8605780	-2.2906540
H	-3.3106400	-3.2053090	-1.2425440
H	-4.2186940	-2.8133770	-2.7084170
H	-4.0712820	0.3938650	-2.1864190
H	-5.3189570	-0.6192370	-2.9415350
H	-5.7250780	0.3876070	-1.5526640
H	-7.0501020	-0.5454490	0.0306210
H	-7.4630460	-2.0836410	0.7555290

H	-3.3104570	-1.7823550	0.4780280
C	2.9605250	-2.4624950	-1.6584260
H	3.0378580	-2.4636390	-2.7546700
C	3.4634170	-3.5121950	-0.9764280
H	3.9154030	-4.3650800	-1.4851650
H	3.3699050	-3.5806790	0.1129240

Int-4

Bi	0.9113690	0.3321390	-0.5002140
C	3.1631010	0.1559210	-0.6381160
C	3.7820590	0.1953420	-1.8946020
H	3.1786170	0.2811160	-2.8058510
C	5.1720070	0.1262710	-2.0155860
H	5.6365000	0.1572020	-3.0048350
C	5.9647780	0.0161910	-0.8747270
H	7.0524140	-0.0388360	-0.9654800
C	3.9752040	0.0437290	0.4993210
C	5.3638530	-0.0248300	0.3840850
H	5.9813630	-0.1115760	1.2821880
C	1.5931000	-2.2095880	1.3178640
C	1.4601720	-3.1222100	2.3661840
H	2.0722570	-4.0279640	2.3787750
C	0.5498150	-2.8805040	3.3947170
H	0.4446360	-3.5948170	4.2148680
C	-0.2218070	-1.7187540	3.3728980
H	-0.9322830	-1.5165040	4.1794050
C	-0.0863700	-0.8097500	2.3216660
H	-0.7089470	0.0907150	2.3178990
C	0.8197530	-1.0423000	1.2793020
H	2.3169510	-2.4176930	0.5217050
H	3.5216700	0.0006030	1.4961530
C	-3.4927690	0.0383120	-0.0496240
C	-2.4363560	-1.9677890	-1.2283820
H	-2.1919200	-1.3383850	0.6801020
C	-4.8342630	-0.7165790	-0.0419700
C	-3.7999740	-2.6787180	-1.1703710
C	-4.9637580	-1.7036870	-1.1877770
H	-5.6666640	0.0069300	-0.0417570
H	-4.9135900	-1.2707840	0.9114280
H	-3.8756340	-3.4054300	-1.9967050
H	-3.8444370	-3.2716680	-0.2382060
H	-5.9194680	-2.2458570	-1.1189820
H	-5.0026630	-1.1691400	-2.1530040
C	-1.3329170	-2.9634500	-0.8909300
H	-0.3356140	-2.5184070	-1.0244840
H	-1.4012700	-3.3092050	0.1528660
H	-1.3928690	-3.8484200	-1.5403870
C	-2.1790110	-1.4351690	-2.6385700
H	-1.2894150	-0.7893070	-2.6552820
H	-2.0051730	-2.2717490	-3.3311400
H	-3.0164300	-0.8563590	-3.0468440
C	-3.2990710	0.7188100	1.2986780
H	-3.2537790	-0.0177330	2.1172270
H	-2.3699560	1.3069750	1.3080500
H	-4.1298380	1.4055770	1.5149640
C	-3.4833460	1.1205480	-1.1293420
H	-4.1874430	1.9240800	-0.8689680
H	-2.4824860	1.5688950	-1.2159240
H	-3.7725680	0.7498500	-2.1204980
N	-2.3474510	-0.8852060	-0.2210420
C	-0.1231230	2.8058580	1.2838420
H	-0.4757210	2.4212460	2.2519560
C	1.1340530	2.2545700	0.7758650
H	1.8350210	1.9626460	1.5716370
H	1.6488850	2.9382820	0.0823510
C	-0.9098350	3.7162490	0.6611970
H	-0.5915950	4.1191990	-0.3103670
C	-2.1645410	4.1935350	1.1870020
H	-2.4546780	3.7963330	2.1692950
C	-2.9870090	5.0630720	0.5730070
H	-2.7412160	5.4840310	-0.4064340
H	-3.9265830	5.3845410	1.0255370

Int-4$_{13a}$

Bi	1.62418700	0.67777400	-0.96495200
C	1.91611300	1.84053700	0.93635100
C	1.49919000	1.34467500	2.18021200
H	1.03248700	0.35408000	2.25264300
C	1.69860200	2.09805300	3.33827900
H	1.37111000	1.70028400	4.30370400
C	2.32160300	3.34902200	3.26851600
H	2.48167300	3.93399800	4.17812600
C	2.53146900	3.10086000	0.87542200
C	2.74093400	3.85037100	2.03574800
H	3.23092200	4.82915600	1.97527100
C	2.96978600	-2.18355700	-1.11647200
C	3.79387100	-3.25993100	-0.78802700
H	3.78647700	-4.16091400	-1.40689100
C	4.61953500	-3.18554100	0.33278200
H	5.26557000	-4.02799500	0.59331000
C	4.61754200	-2.03564800	1.12250300
H	5.26253800	-1.97554800	2.00319700
C	3.78223800	-0.96654400	0.80001900
H	3.77517400	-0.08270700	1.44641900
C	2.94318300	-1.03068400	-0.31986900
H	2.31591500	-2.27096700	-1.99139600
H	2.86063100	3.51894700	-0.08473600
C	-1.75581200	-1.85839500	1.24311900
C	-2.34196900	-0.64741200	0.83838500
C	-3.66911500	-0.39527600	1.21882100
C	-4.37284600	-1.34021600	1.98147300
C	-3.74842800	-2.54061800	2.34980900
C	-2.42733700	-2.81337900	1.98132100
C	-1.41955400	0.12464900	0.03583100
H	-4.14935700	0.54208000	0.94078600
H	-4.30351000	-3.27338300	2.94085300
H	-1.93808600	-3.74539100	2.27288800
C	-5.78183100	-1.04926500	2.39344800
H	-5.82635100	-0.21070900	3.10637700
H	-6.39960300	-0.75504700	1.53097000
H	-6.25920500	-1.91386000	2.87211500
C	-1.63052600	1.43927800	-0.54474300
O	-0.76984300	2.02639700	-1.19308700
O	-2.84610100	1.95768300	-0.30885300
C	-3.12035800	3.22090900	-0.92602500
H	-2.34499000	3.94269000	-0.62425000
H	-3.02953100	3.10840100	-2.02010900
C	-4.49757800	3.64759300	-0.50854100
H	-4.75284700	4.61292500	-0.96301600
H	-5.25611200	2.91669900	-0.82154300
H	-4.56517300	3.75919100	0.58232100
S	-0.10052200	-1.96618400	0.64164300
O	0.00254300	-2.96170400	-0.42308500
O	0.85745000	-2.01190500	1.74039700
N	-0.15981100	-0.38710900	-0.03806000
C	-0.37392600	-0.48172400	-3.38204900
C	-1.40108000	-1.11519200	-2.75321300
H	-1.28491900	-2.17885400	-2.50468200
C	-2.60430600	-0.49015000	-2.33053700
H	-2.76616600	0.55665700	-2.62113600
H	-0.43914700	0.57845200	-3.65284000
H	0.52253400	-1.02371900	-3.69822600
C	-3.71618100	-1.21451700	-1.82425900
H	-3.54332700	-2.28064300	-1.61034900
C	-4.93622300	-0.68910200	-1.53466500
H	-5.74425900	-1.30535700	-1.13183100
H	-5.15728900	0.36848900	-1.72161700

TS-2

Bi	-1.49917000	-1.13509000	0.31211900
C	-2.26708900	0.31446900	1.87515700
C	-1.39776100	0.86299600	2.82386900
H	-0.32148700	0.66891700	2.75604700
C	-1.88041000	1.68776000	3.83937200
H	-1.18784200	2.12132900	4.56582500
C	-3.24466800	1.96728900	3.92326000
H	-3.62322500	2.61748000	4.71595600

C	-3.63527700	0.59430000	1.97387500
C	-4.12303500	1.41884100	2.98994500
H	-5.19225400	1.64020700	3.04694400
C	-3.26166600	-1.01536600	-2.30324500
C	-4.13354400	-0.52617400	-3.27816400
H	-4.52805700	-1.19818600	-4.04470700
C	-4.49676400	0.81867400	-3.26766000
H	-5.17401600	1.20879400	-4.03151400
C	-3.99642500	1.66403500	-2.27693200
H	-4.27954900	2.71962400	-2.26434600
C	-3.13325300	1.17172900	-1.29902000
H	-2.75357400	1.85130100	-0.53121200
C	-2.74681300	-0.17563600	-1.30592400
H	-3.00339900	-2.08086300	-2.32973800
H	-4.34062100	0.18567100	1.24031400
C	1.94291300	2.11059400	-0.27390800
C	2.60778400	0.93954600	0.11094900
C	4.00090500	0.98293800	0.23228100
C	4.69297000	2.18096200	-0.00388600
C	3.98122700	3.33255700	-0.36840000
C	2.59322600	3.30569300	-0.51884700
C	1.66890400	-0.16647100	0.24604700
H	4.55525900	0.08961600	0.51919000
H	4.52466500	4.26387200	-0.54631400
H	2.03568400	4.19711000	-0.81400900
C	6.18472600	2.20231000	0.10887300
H	6.52599300	1.79740500	1.07221400
H	6.64826200	1.57865800	-0.67144400
H	6.59336400	3.21493900	0.00617500
C	1.93312200	-1.43717300	0.92048400
O	1.05476000	-2.20981600	1.26623200
O	3.23982900	-1.70051200	1.08039400
C	3.56064700	-2.98445800	1.63217800
H	3.07860400	-3.08188000	2.61724600
H	3.11691100	-3.76414800	0.99124200
C	5.05513600	-3.08921000	1.70825000
H	5.35054400	-4.06270200	2.11883000
H	5.51260000	-2.98962600	0.71436300
H	5.47678700	-2.30850000	2.35527500
S	0.23154100	1.76183200	-0.52086000
O	-0.01893100	1.61446200	-1.95748000
O	-0.64017500	2.63847600	0.25094600
N	0.34676300	0.22773300	0.20711100
C	-0.07517700	-2.55944900	-1.66871900
C	0.66090100	-1.52851100	-2.16682600
H	0.16397300	-0.80503500	-2.82596900
C	2.02249300	-1.25706800	-1.83575000
H	2.59394200	-2.07123600	-1.37394700
H	0.36804400	-3.32031300	-1.01950100
H	-1.07186100	-2.76972800	-2.05930100
C	2.78135200	-0.28993600	-2.57411300
H	2.19837700	0.45703000	-3.12850500
C	4.12993300	-0.20954000	-2.58340900
H	4.65626100	0.56837900	-3.13992500
H	4.74570800	-0.93413100	-2.04027000

Int-5

Bi	-1.46757800	-0.70847200	-0.94622400
C	-2.08789400	-1.75842000	0.92502700
C	-1.17564900	-2.06786700	1.93738300
H	-0.13871600	-1.73059800	1.85856600
C	-1.58902400	-2.77783600	3.06326200
H	-0.87059100	-3.00526800	3.85509600
C	-2.91695800	-3.18837900	3.18708500
H	-3.23877300	-3.74291100	4.07204500
C	-3.41869500	-2.17086900	1.05557500
C	-3.83372100	-2.88345000	2.18292400
H	-4.87712400	-3.19537500	2.27714400
C	-2.81109500	2.03642800	-1.65124400
C	-3.53306900	3.22103800	-1.50834900
H	-3.54889100	3.95421700	-2.31882900
C	-4.22830200	3.46778700	-0.32556700
H	-4.79044400	4.39733500	-0.20592300
C	-4.20106300	2.53038400	0.70659400

H	-4.73939700	2.72668700	1.63736300
C	-3.47314400	1.34924700	0.56480600
H	-3.43022800	0.63962400	1.39601800
C	-2.76777000	1.09271300	-0.61625200
H	-2.26498500	1.86325300	-2.58728400
H	-4.15848800	-1.92890200	0.28299500
C	1.71934100	1.36467700	1.68017900
C	2.46705700	0.61018900	0.79092200
C	3.83929200	0.47279400	1.00182800
C	4.44258500	1.07368500	2.11336500
C	3.64871900	1.82520200	2.99871800
C	2.28419700	1.98464400	2.78917400
C	1.64776700	0.11227500	-0.39273500
H	4.45004300	-0.10301200	0.30238400
H	4.11976700	2.29776600	3.86506300
H	1.66819300	2.57885700	3.46749800
C	5.90846900	0.91513300	2.36770700
H	6.42194100	0.43639600	1.52459700
H	6.39533400	1.88324700	2.55197600
H	6.09747200	0.29867300	3.25971000
C	1.82474500	-1.39488500	-0.59794000
O	0.93146600	-2.20803500	-0.53349400
O	3.08105700	-1.71376600	-0.90328300
C	3.33678700	-3.10529800	-1.17388600
H	3.03734400	-3.69371000	-0.29348900
H	2.68123400	-3.42053200	-2.00069600
C	4.79248000	-3.24992700	-1.50059800
H	5.03056800	-4.29833100	-1.71848800
H	5.06655800	-2.65221900	-2.38022700
H	5.42428700	-2.92866500	-0.66185300
S	0.03265700	1.48330100	1.15484000
O	-0.23381200	2.84132200	0.67974100
O	-0.87543000	0.94056300	2.16435300
N	0.25554600	0.39141600	-0.09378600
C	1.88453300	-0.53177600	-3.79751400
C	1.41188700	0.34478000	-2.90840800
H	0.40170300	0.75455300	-3.04410900
C	2.15533100	0.84402200	-1.70574500
H	3.22170300	0.58346500	-1.80824900
H	2.89454300	-0.94554100	-3.70919300
H	1.29065200	-0.86092000	-4.65289800
C	2.00980400	2.32750100	-1.56055300
H	0.98073600	2.70001500	-1.48925000
C	3.03116100	3.18076300	-1.48138500
H	2.86922400	4.25389800	-1.36032400
H	4.07109300	2.84047100	-1.52877300

$BiPh_2$-rad

Bi	-0.2159320	-1.0112820	-0.0149700
C	-2.0311350	0.2089640	0.4716220
C	-2.9668950	-0.2961840	1.3886340
H	-2.7847000	-1.2499320	1.8961080
C	-4.1372840	0.4044800	1.6793520
H	-4.8502540	-0.0023400	2.4012590
C	-4.3957720	1.6231260	1.0521390
H	-5.3121240	2.1735750	1.2784480
C	-2.3097480	1.4329950	-0.1576650
C	-3.4801350	2.1339130	0.1315380
H	-3.6807180	3.0847150	-0.3693640
C	2.1406500	0.5983960	-1.3951650
C	3.0131310	1.6515830	-1.6685770
H	3.8181640	1.5168630	-2.3957090
C	2.8567750	2.8750270	-1.0174280
H	3.5391950	3.7014270	-1.2303450
C	1.8266840	3.0372230	-0.0903560
H	1.7036010	3.9912020	0.4291910
C	0.9542370	1.9838190	0.1814960
H	0.1565350	2.1296230	0.9169510
C	1.0934260	0.7486990	-0.4718820
H	2.2820150	-0.3522040	-1.9212640
H	-1.6074210	1.8479100	-0.8877580

Alkyl rad

C	1.3567000	0.5212400	-0.0002270
H	1.7050390	-0.5200850	-0.0001140
C	2.2913880	1.5161180	-0.0003660
H	1.9968090	2.5697110	-0.0004840
H	3.3609900	1.2994570	-0.0003720
C	-0.0406770	0.7190380	-0.0002190
C	-0.9871160	-0.3278810	-0.0000930
C	-2.3421360	-0.1623520	-0.0000920
H	-0.5877170	-1.3507040	0.0000090
H	-3.0249810	-1.0136800	0.0000050
H	-2.7904970	0.8355700	-0.0001920
H	-0.4147190	1.7513980	-0.0003280

Int-4'

Bi	-0.71033600	-0.32583500	-0.32271500
C	-2.84646600	0.28285400	-0.72840100
C	-3.45239500	-0.13332900	-1.92066400
H	-2.86808000	-0.66363800	-2.68149700
C	-4.80802600	0.10307200	-2.16046100
H	-5.26374500	-0.23147200	-3.09621200
C	-5.57931300	0.76054900	-1.20388800
H	-6.64099700	0.94425800	-1.38639700
C	-3.63534100	0.94533200	0.22235000
C	-4.99035600	1.18271500	-0.01099100
H	-5.59132700	1.69745900	0.74356400
C	-0.84676400	2.74532200	0.56625700
C	-0.48943900	3.91338100	1.24284800
H	-0.92814600	4.86909400	0.94391800
C	0.42382300	3.86325800	2.29533300
H	0.70289200	4.77701800	2.82553500
C	0.97828600	2.63867500	2.66693300
H	1.69303900	2.58783900	3.49318700
C	0.62334400	1.47439100	1.98309700
H	1.08740100	0.52851300	2.27803900
C	-0.29657700	1.50740100	0.92642000
H	-1.56660700	2.80986600	-0.25703000
H	-3.19181400	1.28149800	1.16677500
C	-0.48239100	-2.00282200	2.39623800
H	-0.16312600	-1.14732000	3.00630500
C	-1.54248800	-1.70050400	1.41699300
H	-2.29959400	-1.01828700	1.83874900
C	0.12089300	-3.18150000	2.61427500
H	-0.15229200	-4.09284600	2.07586600
C	-2.15178700	-2.82844600	0.68785800
H	-1.46579400	-3.63410800	0.39100400
C	-3.43536100	-2.91970600	0.31240000
H	-3.80225400	-3.77115200	-0.26453600
H	-4.16490300	-2.14348200	0.56570800
H	0.91326200	-3.27759600	3.35949700

TS-2ins

Bi	1.27039900	0.01804700	-1.23821400
C	2.46247100	-1.50475900	-0.00706500
C	2.01287100	-2.57181500	0.77699500
H	0.95136600	-2.68687600	1.01371200
C	2.90998000	-3.51355300	1.28670300
H	2.53672000	-4.34054700	1.89442900
C	4.27503900	-3.40356000	1.01690500
H	4.97578600	-4.13964300	1.41727000
C	3.83734800	-1.41393500	-0.27740700
C	4.74033500	-2.34943000	0.23158700
H	5.80826500	-2.25205700	0.01465900
C	3.02053500	1.61402800	0.99174900
C	3.41504800	2.72392200	1.73785800
H	4.15571300	2.60687200	2.53392400
C	2.85648500	3.97720700	1.48311300
H	3.15675400	4.84237600	2.07980800
C	1.90782700	4.11883700	0.47196400
H	1.45410700	5.09301900	0.27390800
C	1.52301000	3.01171200	-0.28287300
H	0.75716300	3.14512000	-1.05156500
C	2.07149400	1.74370700	-0.03331200
H	3.45138700	0.63548000	1.22786300

H	4.23430200	-0.59217500	-0.89222500
C	-3.18612000	-1.47632800	0.36215600
C	-2.93072100	-0.12008900	0.57989100
C	-3.99529600	0.74349000	0.85789600
C	-5.30375400	0.24372500	0.88947100
C	-5.51668600	-1.12837500	0.66203100
C	-4.46260900	-2.00814600	0.41223300
C	-1.48209400	0.15655700	0.50940400
H	-3.79865300	1.80209200	1.04102900
H	-6.53887000	-1.51607100	0.68969800
H	-4.63819800	-3.07449400	0.25747800
C	-6.46414200	1.14768900	1.15688700
H	-7.07700600	0.77851100	1.99105000
H	-6.14339400	2.16734900	1.39941500
H	-7.13062700	1.20853200	0.28366800
C	-1.00269800	1.39562000	1.23523900
O	-1.56693400	2.45584400	1.11385200
O	-0.04253400	1.26794500	2.15089100
C	0.53700700	0.03914700	2.62999600
H	-0.24527800	-0.72755800	2.74505800
H	1.25273900	-0.33422300	1.88348000
C	1.21602400	0.34632300	3.93212300
H	1.69955000	-0.55865500	4.32127700
H	1.98591400	1.11743300	3.79705900
H	0.50167900	0.70715300	4.68378100
S	-1.64092700	-2.33081500	0.16678500
O	-1.49847500	-2.96931400	-1.13148900
O	-1.36575500	-3.10202700	1.37864800
N	-0.72437900	-0.88644100	0.23916700
C	-0.95040700	1.14078700	-1.83147900
H	-0.22366100	1.56005400	-2.55543500
C	-1.87552100	2.24572900	-1.55097500
C	-1.69238700	3.53013500	-1.90003500
H	-2.82178400	1.99114300	-1.06111900
H	-0.79078300	3.86414700	-2.42402000
C	-1.58914500	-0.03565200	-2.43783700
H	-2.50328200	-0.40345800	-1.95285800
C	-1.15899100	-0.71749600	-3.51357400
H	-0.27805400	-0.40310500	-4.08320200
H	-1.67144100	-1.61739800	-3.85755500
H	-2.43793600	4.29474200	-1.67459700

TS-2out

Bi	-2.78344400	0.02020200	-1.12170000
C	-4.27636500	0.70333900	0.40973000
C	-4.31336400	2.02024100	0.88396800
H	-3.56845700	2.75378100	0.55909600
C	-5.30090800	2.42495000	1.78380200
H	-5.31315000	3.45523900	2.14808700
C	-6.26789100	1.51898600	2.21643000
H	-7.03965300	1.83579000	2.92163300
C	-5.26003100	-0.19662600	0.84452800
C	-6.24740400	0.20736200	1.74412200
H	-7.00304200	-0.50818500	2.07777100
C	-2.32007800	-1.91280800	1.42957600
C	-1.71024800	-2.94936500	2.13561700
H	-2.02766200	-3.16787100	3.15861800
C	-0.68623100	-3.69383000	1.54733800
H	-0.20322300	-4.49702100	2.10967300
C	-0.27299300	-3.40537200	0.24771900
H	0.54500300	-3.95857700	-0.22024500
C	-0.89875300	-2.38436400	-0.46948800
H	-0.53594400	-2.17350200	-1.48136500
C	-1.92110900	-1.62556200	0.11853600
H	-3.10127900	-1.32096700	1.91682500
H	-5.26105800	-1.23418300	0.49234300
C	3.87728400	-1.23963300	-0.48819100
C	3.51605200	-0.14744300	0.29151600
C	3.99260100	-0.04272800	1.59212200
C	4.83089400	-1.04174500	2.11290700
C	5.16291600	-2.13891200	1.30379200
C	4.68919100	-2.25289300	-0.00523400
C	2.56459000	0.74674900	-0.47387600
H	3.72271100	0.81527500	2.21447500

H	5.81633100	-2.91677400	1.70893500
H	4.96208200	-3.10299900	-0.63469200
C	5.35718100	-0.92295700	3.50913500
H	6.06173800	-0.08270200	3.60521500
H	4.54880700	-0.73342600	4.23080800
H	5.88521800	-1.82979100	3.82929300
C	2.64268800	2.24934200	-0.24132100
O	1.71221200	3.00704800	-0.39717500
O	3.86689800	2.63505200	0.10191900
C	4.07453600	4.05273100	0.23016100
H	3.83947100	4.52628900	-0.73544500
H	3.34943900	4.44878900	0.95836200
C	5.49697300	4.27152600	0.64926700
H	5.70338900	5.34408400	0.75288800
H	5.70661800	3.79044000	1.61380000
H	6.19485100	3.86011000	-0.09107900
S	3.05529500	-1.05557300	-2.06800600
O	1.86994300	-1.94484200	-2.02369600
O	3.95729400	-1.21171500	-3.20100700
N	2.60763600	0.49834500	-1.82942000
C	-1.18386400	1.64311400	-0.37783300
C	0.05964500	1.06273600	-0.72078200
H	0.37981900	1.07188800	-1.77282700
C	0.92490400	0.32104200	0.13457400
H	1.03851900	-0.71609100	-0.23154000
H	-1.48036100	2.51821700	-0.97380700
H	-1.40864900	1.74365100	0.69255900
C	0.71596200	0.40944500	1.59026300
H	0.37765400	1.38037600	1.97715500
C	0.96950400	-0.58374300	2.45117900
H	1.32417000	-1.55973400	2.10298200
H	0.82947000	-0.46394000	3.52791200

TS-2’out

Bi	3.55465100	-0.20248600	-1.26862300
C	4.74391400	0.16021200	0.59868500
C	5.15885100	-0.90518600	1.40762200
H	4.88210200	-1.93551500	1.15938500
C	5.92662000	-0.67489400	2.55055300
H	6.23834300	-1.51621100	3.17456900
C	6.29296100	0.62511100	2.89555100
H	6.89274600	0.80622800	3.79040700
C	5.12067300	1.46256300	0.95406800
C	5.88924800	1.69363600	2.09566200
H	6.17063000	2.71529200	2.36288100
C	1.72784300	1.72998000	0.52515700
C	0.60534900	2.50749100	0.81123400
H	0.37833700	2.77525900	1.84595800
C	-0.24065800	2.92088300	-0.21943400
H	-1.12539400	3.52212800	0.00528100
C	0.04058100	2.56025900	-1.53611900
H	-0.62118200	2.87898400	-2.34554200
C	1.15913200	1.77350400	-1.81958400
H	1.34686000	1.48101600	-2.85861800
C	2.01255900	1.34817500	-0.79328200
H	2.37056100	1.39853000	1.34826600
H	4.80219900	2.31724300	0.34743600
C	2.20273800	-1.78079200	-0.14270600
C	0.91963100	-1.92347900	-0.79061100
H	0.80076200	-2.73264100	-1.52400900
C	-0.14533300	-1.07112300	-0.60916400
H	-0.02716600	-0.22932100	0.08620700
H	2.79369400	-2.70634800	-0.10127800
H	2.15649200	-1.29747400	0.84549200
C	-1.38803900	-1.19489000	-1.24623500
H	-1.57313000	-2.08799800	-1.85585700
C	-2.44685400	-0.28862700	-1.03944500
H	-2.11294600	0.70745600	-0.72043200
H	-3.22280700	-0.27678000	-1.81317300
C	-3.50378800	-0.70727200	0.35612900
C	-4.38616100	0.53419800	0.41922200
C	-2.44110900	-0.83181100	1.43469400
N	-4.21151400	-1.83483600	0.09742400
C	-5.63209100	0.25392500	-0.12647000

C	-4.10784200	1.82232100	0.87230200
O	-1.93561000	0.11463700	1.99523500
O	-2.07252700	-2.09303400	1.62498200
S	-5.62040900	-1.45094200	-0.66026600
C	-6.62691200	1.21443200	-0.23223300
C	-5.09678000	2.81490900	0.79630900
H	-3.13147100	2.05303500	1.30321600
C	-0.97458100	-2.29643300	2.52606500
O	-5.41366100	-1.47166900	-2.11705000
O	-6.74213700	-2.20011600	-0.11013900
C	-6.34659000	2.49561200	0.23972800
H	-7.59961700	0.96869400	-0.66415300
C	-4.83350500	4.18924500	1.32780300
H	-0.16522400	-1.59543000	2.26170000
H	-1.29930600	-2.02471200	3.54291600
C	-0.55199900	-3.73099700	2.41932400
H	-7.11476600	3.27176500	0.17810100
H	-5.24652200	4.31190800	2.34129000
H	-3.75897700	4.40346500	1.39595400
H	-5.29686100	4.96649800	0.70505500
H	0.27967600	-3.93735400	3.10519700
H	-1.37862100	-4.40797400	2.66996400
H	-0.22295200	-3.96870500	1.39863300

Int-4’$_{13a}$

Bi	1.42090600	0.73680100	-0.79308300
C	1.32754000	2.03221100	1.04678900
C	0.82103400	1.56087100	2.26596700
H	0.47291400	0.52625400	2.35294400
C	0.78050000	2.39297900	3.38489800
H	0.38337800	2.01086300	4.32939200
C	1.25507800	3.70294200	3.30424600
H	1.23179800	4.34969100	4.18456000
C	1.78964600	3.35133600	0.97503900
C	1.76213400	4.18224700	2.09774100
H	2.13712000	5.20683000	2.02924700
C	3.09220500	-1.98958300	-0.40530500
C	3.96514200	-2.91001400	0.17228700
H	4.16309800	-3.85745100	-0.33602300
C	4.57443300	-2.62249900	1.39161800
H	5.25702500	-3.34350900	1.84821800
C	4.30367400	-1.41023700	2.02416200
H	4.77660100	-1.17834900	2.98198000
C	3.42234600	-0.49484100	1.44716300
H	3.21704600	0.44073500	1.97616200
C	2.79394200	-0.77121500	0.22401100
H	2.62446800	-2.25084300	-1.35430700
H	2.18321300	3.75362800	0.03401700
C	-2.01575000	-2.02000200	1.05017400
C	-2.62787000	-0.84098400	0.60862900
C	-4.00245500	-0.68058100	0.84481300
C	-4.72853400	-1.69018800	1.48060400
C	-4.07389800	-2.87143300	1.88652400
C	-2.70995200	-3.04569500	1.67628000
C	-1.65906000	0.04632000	-0.02002800
H	-4.50628000	0.23007200	0.52123600
H	-4.64735400	-3.65588800	2.38748800
H	-2.19544900	-3.95209000	2.00284000
C	-6.19057300	-1.52266000	1.74710600
H	-6.58731600	-0.60322200	1.29972800
H	-6.77317800	-2.36795100	1.35364100
H	-6.39681900	-1.48090300	2.82730900
C	-1.88156600	1.34699200	-0.63871900
O	-0.97856900	2.01220200	-1.13081900
O	-3.15014500	1.77351100	-0.56482800
C	-3.45921900	2.96552000	-1.29625500
H	-3.00064400	3.83068200	-0.79095800
H	-2.98807500	2.90090000	-2.28971400
C	-4.95318200	3.07585200	-1.38544100
H	-5.23598100	3.97835800	-1.94168300
H	-5.38471800	2.21044600	-1.90740700
H	-5.41309900	3.13575300	-0.38991900
S	-0.27784600	-1.96538500	0.75502500
O	0.13453900	-2.94309100	-0.24951200

O	0.45865400	-1.89796600	2.01144300
N	-0.37209600	-0.39375600	0.03769700
C	-0.11980000	-0.78237900	-2.99361700
C	-1.35317700	-1.31029900	-2.60248800
H	-1.37027700	-2.36268200	-2.28455100
C	-2.50466100	-0.56551900	-2.47843900
H	-2.55246300	0.46267900	-2.84023000
H	-0.10051400	0.26475900	-3.33168100
C	1.04938500	-1.57322100	-3.20329900
H	0.99250500	-2.61914200	-2.87215800
C	2.20659400	-1.10709000	-3.73467200
H	2.29600400	-0.06910400	-4.07437800
H	3.08906000	-1.74105200	-3.84295400
H	-3.45656700	-1.02171900	-2.14020000

TS-2’

Bi	-1.43822500	0.70705400	0.79800900
C	-1.35626700	2.08095000	-0.96995300
C	-0.84512500	1.65626100	-2.20379200
H	-0.48336500	0.62934200	-2.32531200
C	-0.82058800	2.52856300	-3.29255700
H	-0.42363000	2.18542900	-4.25163100
C	-1.31165700	3.82899300	-3.16411500
H	-1.29970100	4.50698000	-4.02126300
C	-1.83498600	3.39082200	-0.84725700
C	-1.82047000	4.26037700	-1.94032200
H	-2.20727200	5.27767400	-1.83401000
C	-3.08848600	-1.98004100	0.35958200
C	-3.95487500	-2.89367700	-0.24015200
H	-4.14484000	-3.85745700	0.23919600
C	-4.57125100	-2.57930900	-1.44992300
H	-5.24815800	-3.29535200	-1.92283800
C	-4.32128100	-1.34798300	-2.05444200
H	-4.80362200	-1.09502500	-3.00227700
C	-3.44521100	-0.44070000	-1.45983500
H	-3.24971500	0.51082400	-1.96335100
C	-2.80900600	-0.74893800	-0.24989400
H	-2.60870400	-2.25158100	1.30438700
H	-2.23166900	3.75420400	0.10797400
C	2.00236500	-1.95741300	-1.12735700
C	2.62683400	-0.81497800	-0.61575000
C	3.99841900	-0.64424800	-0.86298000
C	4.70599600	-1.60347200	-1.59143100
C	4.03841400	-2.74957600	-2.06924900
C	2.67917600	-2.93529000	-1.84355300
C	1.67941200	0.00939400	0.12703600
H	4.51309800	0.23965000	-0.48503400
H	4.59987500	-3.49967300	-2.63238800
H	2.15476200	-3.81589900	-2.22103500
C	6.16386000	-1.42025600	-1.87400300
H	6.56806800	-0.52634000	-1.38332700
H	6.75314600	-2.28507500	-1.53625100
H	6.35312200	-1.31958000	-2.95327000
C	1.88938200	1.32752200	0.70766800
O	0.99359100	1.96211700	1.25323700
O	3.15302900	1.77078900	0.62547800
C	3.46186600	2.94296400	1.38961100
H	3.00470700	3.82009000	0.90429700
H	2.98758100	2.85467300	2.38030600
C	4.95614000	3.04707500	1.48157800
H	5.24462900	3.92626800	2.07124000
H	5.38267200	2.15884700	1.96879500
H	5.41435500	3.14129600	0.48783200
S	0.27864700	-1.93595600	-0.75809200
O	-0.08546200	-2.97821900	0.20004200
O	-0.50433600	-1.81332100	-1.98319300
N	0.38638800	-0.41605300	0.03388600
C	0.11233102	-0.90656037	2.96637127
C	1.28106953	-1.38361544	2.40816490
H	1.27577302	-2.41134923	2.02503402
C	2.39429302	-0.58799615	2.15310859
H	2.47146830	0.39306115	2.63093640
H	0.10451951	0.12569985	3.34569798
C	-1.09559541	-1.66062439	3.07526057

H	-1.06633339	-2.68079880	2.67128050
C	-2.25387554	-1.18596570	3.59033377
H	-2.32113023	-0.16947184	3.99186934
H	-3.15992449	-1.79321381	3.62862880
H	3.32785145	-1.01852547	1.78582548

Int-5

Bi	1.56586000	0.54347800	-1.26171100
C	3.25586900	-0.57329300	-0.30433500
C	3.52698700	-1.89876500	-0.67243300
H	2.86190200	-2.43534600	-1.35419600
C	4.63645300	-2.56134800	-0.14824200
H	4.82694700	-3.59988600	-0.42960900
C	5.49593500	-1.90580000	0.73222200
H	6.36527800	-2.42752700	1.13995600
C	4.13065000	0.08170600	0.57334600
C	5.24492700	-0.58206500	1.08884900
H	5.91735300	-0.06022000	1.77457200
C	1.46379200	1.74078900	1.68099200
C	1.27015900	2.70042700	2.67245600
H	1.26864900	2.40414900	3.72447000
C	1.05900500	4.03392100	2.32218900
H	0.89822700	4.78380400	3.10020700
C	1.04136100	4.40817500	0.97997700
H	0.86978900	5.45142100	0.70367100
C	1.22804200	3.44544200	-0.01294600
H	1.19247600	3.75480100	-1.06392200
C	1.44165500	2.10496700	0.32850600
H	1.61185300	0.69167600	1.96055000
H	3.94939600	1.12006600	0.86782400
C	-1.68247300	-2.47070200	-0.21624600
C	-2.30425700	-1.26988700	0.07950500
C	-3.65339700	-1.26620400	0.43501800
C	-4.36812200	-2.46875100	0.48088600
C	-3.70157600	-3.67061300	0.17099000
C	-2.35429400	-3.68864300	-0.16974400
C	-1.39356200	-0.06349800	-0.02762200
H	-4.14857100	-0.32498100	0.69358800
H	-4.25645600	-4.61160900	0.21711000
H	-1.83252000	-4.62454300	-0.38012500
C	-5.81674400	-2.48640700	0.85177600
H	-6.01722900	-3.19548200	1.66760300
H	-6.17071900	-1.49788700	1.16956200
H	-6.44247100	-2.80446000	0.00364400
C	-1.52929200	0.85556800	1.21545300
O	-1.83073600	2.01664200	1.10496600
O	-1.32380600	0.35920100	2.44016400
C	-0.95885200	-0.98966600	2.77971200
H	-1.83635300	-1.64493900	2.64872100
H	-0.16290800	-1.35446600	2.11742000
C	-0.50961100	-0.97452500	4.21284600
H	-0.24894600	-1.98976900	4.53710700
H	0.37788400	-0.34074300	4.34131100
H	-1.29758900	-0.59126200	4.87429900
S	0.04489000	-2.21329300	-0.47091700
O	0.45165500	-2.38940800	-1.87761900
O	0.79867300	-2.96819800	0.53088900
N	-0.01635800	-0.58089600	-0.11788000
C	-1.75825100	0.75924900	-1.30039000
H	-1.18103000	1.69778700	-1.25428200
C	-3.19857800	1.06683700	-1.52163000
C	-3.79906700	2.24339300	-1.25945500
H	-3.80767200	0.25675800	-1.94416000
H	-3.20508600	3.05353500	-0.82040800
H	-1.38464400	0.16052400	-2.14951500
C	-5.20123500	2.49986500	-1.49888300
C	-5.82206300	3.65854200	-1.22397500
H	-5.28004100	4.50250600	-0.78785500
H	-5.77934300	1.67410800	-1.93449700
H	-6.88535800	3.80408000	-1.42133600

13-TMS’

C	1.15370500	-1.61494400	-0.87945900

C	1.52481700	-0.62321200	0.01427600
C	2.79335400	-0.67232000	0.59290900
C	3.68227700	-1.69987900	0.25411100
C	3.27169500	-2.68261300	-0.66494200
C	2.00284700	-2.65875800	-1.23147800
C	0.43191700	0.40860900	0.27345300
H	3.10029200	0.09637500	1.30649800
H	3.96448900	-3.48571400	-0.93042600
H	1.67569100	-3.43129900	-1.93032200
C	5.05519100	-1.75123200	0.84593800
H	5.82579500	-1.54141700	0.08867100
H	5.18217400	-1.01983300	1.65356600
H	5.28414800	-2.74620500	1.25322700
C	0.98344000	1.80422500	-0.04611000
O	0.67507700	2.47176700	-0.99925700
O	1.85215400	2.19565600	0.89650100
C	2.43046100	3.50564900	0.73377800
H	3.36007500	3.46835800	1.31506600
H	2.68735400	3.64693700	-0.32611900
C	1.50067100	4.58008200	1.22557000
H	1.99265000	5.56021900	1.17739400
H	0.59517400	4.63254500	0.60773400
H	1.20424200	4.40318200	2.26864300
S	-0.53808200	-1.43185400	-1.34302400
O	-1.32157700	-2.44411600	-0.63217700
O	-0.74626800	-1.32549500	-2.78637400
N	-0.64708400	0.09067200	-0.65007300
C	-0.01913400	0.36044700	1.76635700
C	-0.82114700	-0.85465400	2.04955100
C	-2.08834000	-0.85109000	2.50009100
H	-0.35932100	-1.81942700	1.80341000
H	-2.56925500	0.10917600	2.73582300
H	-0.59636100	1.26866300	2.00233200
Si	-2.09056800	1.10555900	-1.12105900
C	-3.53790100	-0.09848000	-1.14722900
H	-3.40507800	-0.87341600	-1.91551900
H	-3.65371800	-0.61433300	-0.18310700
H	-4.47773100	0.42952800	-1.37018400
C	-1.84881800	1.87908700	-2.81416200
H	-1.08204900	2.66253400	-2.80081500
H	-1.53622300	1.10774700	-3.53105100
H	-2.79166800	2.31496300	-3.17722100
C	-2.36600600	2.39935300	0.22494900
H	-2.65125500	1.94519700	1.18456500
H	-1.50293700	3.05856700	0.38997700
H	-3.20245200	3.04695300	-0.08045300
C	-2.89038300	-2.04106900	2.67569700
C	-4.15719500	-2.04545000	3.12070000
H	-2.41426300	-2.98766500	2.39294900
H	-4.73107900	-2.96849500	3.21637900
H	-4.66565300	-1.11762600	3.40028800
H	0.89001800	0.40662100	2.38886000

Int-4’II

Bi	-0.71033600	-0.32583500	-0.32271500
C	-2.84646600	0.28285400	-0.72840100
C	-3.45239500	-0.13332900	-1.92066400
H	-2.86808000	-0.66363800	-2.68149700
C	-4.80802600	0.10307200	-2.16046100
H	-5.26374500	-0.23147200	-3.09621200
C	-5.57931300	0.76054900	-1.20388800
H	-6.64099700	0.94425800	-1.38639700
C	-3.63534100	0.94533200	0.22235000
C	-4.99035600	1.18271500	-0.01099100
H	-5.59132700	1.69745900	0.74356400
C	-0.84676400	2.74532200	0.56625700
C	-0.48943900	3.91338100	1.24284800
H	-0.92814600	4.86909400	0.94391800
C	0.42382300	3.86325800	2.29533300
H	0.70289200	4.77701800	2.82553500
C	0.97828600	2.63867500	2.66693300
H	1.69303900	2.58783900	3.49318700
C	0.62334400	1.47439100	1.98309700
H	1.08740100	0.52851300	2.27803900

C	-0.29657700	1.50740100	0.92642000
H	-1.56660700	2.80986600	-0.25703000
H	-3.19181400	1.28149800	1.16677500
C	-0.48239100	-2.00282200	2.39623800
H	-0.16312600	-1.14732000	3.00630500
C	-1.54248800	-1.70050400	1.41699300
H	-2.29959400	-1.01828700	1.83874900
C	0.12089300	-3.18150000	2.61427500
H	-0.15229200	-4.09284600	2.07586600
C	-2.15178700	-2.82844600	0.68785800
H	-1.46579400	-3.63410800	0.39100400
C	-3.43536100	-2.91970600	0.31240000
H	-3.80225400	-3.77115200	-0.26453600
H	-4.16490300	-2.14348200	0.56570800
H	0.91326200	-3.27759600	3.35949700

$BiMe_2SbF_6$

Bi	-1.77823600	-0.06845800	-0.29745500
C	-1.63286200	1.92479900	0.64622500
H	-0.67500600	1.90991000	1.18827900
H	-1.58290000	2.72115900	-0.10718500
H	-2.45331900	2.10954300	1.35229900
C	-2.78529200	-0.93683600	1.48392700
H	-2.82655600	-2.03322900	1.44028500
H	-2.22543400	-0.63180900	2.37925500
H	-3.81166400	-0.54682600	1.56348500
Sb	1.88232100	-0.02442700	0.02719500
F	1.71599500	1.29903300	1.34158100
F	2.66618500	-1.19742300	1.23897500
F	0.67486100	1.00592000	-1.03866000
F	1.63665900	-1.39214200	-1.22613000
F	3.42845500	0.66196900	-0.73253500
F	0.06440600	-0.65832900	0.71726600

$BiMe_2$(cyclopentene)SbF_6

Bi	1.05200600	0.44546900	-0.20183900
C	1.00672500	-0.34139700	1.87503100
H	-0.03102500	-0.18175100	2.20220700
H	1.19992500	-1.42231300	1.89166800
H	1.69572400	0.18776500	2.54645800
C	1.50067900	2.54646400	0.41319800
H	2.34562400	2.96351200	-0.15378600
H	0.60699700	3.14977100	0.20033000
H	1.71592400	2.61496700	1.48840200
Sb	-2.57121600	-0.25985900	-0.02253700
F	-2.57098600	-0.31501500	1.85125300
F	-3.78099300	1.15727400	-0.03773600
F	-1.06943800	-1.44084700	0.01594500
F	-2.22840200	-0.06330300	-1.85736300
F	-3.81546500	-1.62512500	-0.23106400
F	-1.09076100	1.08265000	0.17458100
C	3.88971900	0.16431100	-0.78438900
H	3.84358500	1.04670500	-1.43065300
C	3.97120600	0.20175800	0.56538000
H	3.97089300	1.11572600	1.16670100
C	4.06228200	-1.23480500	-1.29878200
H	3.38635400	-1.49281800	-2.12823200
H	5.08404700	-1.34305500	-1.70641900
C	4.20356200	-1.16524600	1.12903900
H	5.26792900	-1.25119000	1.41706000
H	3.63510600	-1.37223900	2.04812600
C	3.84900100	-2.08733700	-0.04268100
H	2.78476500	-2.37177200	0.02794500
H	4.41502900	-3.02763400	-0.04913100

$BiMe_2$(pentadiene)SbF_6

Bi	-0.93141600	-0.72267300	-0.24824600
C	-0.83248800	-0.47795100	1.95348000
H	0.24175000	-0.47978600	2.19025800
H	-1.23851100	0.49548100	2.26052200
H	-1.33335800	-1.29247500	2.49240700
C	-1.05105800	-2.95368800	-0.15496200

H	-1.57061900	-3.36892300	-1.03011800
H	-0.01484500	-3.32130100	-0.15695000
H	-1.54516300	-3.29877700	0.76341500
Sb	2.54235800	0.52079800	0.00824700
F	2.61342700	0.04535300	1.82135300
F	3.94463700	-0.63338200	-0.40381800
F	0.87795900	1.38346100	0.37517400
F	2.15284300	0.79068000	-1.80600800
F	3.55497000	2.06737100	0.20153100
F	1.29093900	-1.05038000	-0.17576300
C	-3.88696100	-0.02486700	-0.23694900
H	-4.05653400	-0.05429600	-1.32216500
C	-3.83327000	-1.18439700	0.44397900
H	-3.95576100	-2.15309600	-0.04776700
C	-3.80247800	1.33982400	0.37830200
H	-2.98026400	1.91322600	-0.08607800
H	-3.71193800	-1.19733000	1.53504600
H	-3.55536700	1.24156100	1.45061300
C	-5.07879200	2.11006900	0.21850200
H	-5.95952200	1.68254600	0.71188500
C	-5.19720700	3.24840600	-0.46567100
H	-4.34175900	3.70871600	-0.96791200
H	-6.15028600	3.77336800	-0.54161100

$BiMe_2$(allylbenzene)SbF_6

Bi	0.01426800	1.06010000	-0.27332500
C	0.15127900	0.63300100	1.89812400
H	-0.88319800	0.42909900	2.21054700
H	0.73886500	-0.27485200	2.08780000
H	0.55220200	1.47878900	2.47254900
C	-0.34479200	3.24558400	0.04687200
H	-0.16907400	3.83157800	-0.86562500
H	-1.40411300	3.33751400	0.32709700
H	0.27093400	3.64592600	0.86362900
Sb	-3.08258700	-0.92488900	0.01988500
F	-3.15871400	-0.68969300	1.87758800
F	-4.72525300	-0.07765800	-0.21124100
F	-1.25214500	-1.43068800	0.23321200
F	-2.73845700	-0.88811700	-1.82406700
F	-3.71874400	-2.67071000	0.04170900
F	-2.21513300	0.89321900	-0.00834200
C	3.06147300	0.94010800	-0.37012900
H	3.15941800	0.79685500	-1.45528000
C	2.77081600	2.16807400	0.09675400
H	2.64120600	3.02360000	-0.57151000
C	3.33634600	-0.26589100	0.46910700
H	2.59719900	-1.05377900	0.24334900
H	2.70965000	2.37004600	1.17188000
H	3.20222600	-0.01260500	1.53432500
C	4.72158100	-0.81600100	0.22550100
C	4.91140900	-2.13660900	-0.19384400
C	5.84541900	-0.00194300	0.41740800
C	6.19518300	-2.63653100	-0.40910600
H	4.04266200	-2.78273900	-0.34874800
C	7.12830500	-0.49949000	0.20303100
H	5.71117200	1.03444200	0.74237400
C	7.30659000	-1.81949000	-0.21155400
H	6.32594500	-3.67127400	-0.73340900
H	7.99464400	0.14688000	0.36063500
H	8.31227600	-2.21044300	-0.38049700

$BiPh_2SbF_6$

Bi	-0.75291200	-0.21174400	-1.02080800
C	-1.23955300	1.80225100	-0.23795200
C	-0.23719400	2.59765100	0.33026300
C	-2.55134200	2.28789700	-0.32163800
C	-0.55345200	3.86616500	0.81697100
H	0.78396600	2.22428800	0.42039500
C	-2.85673700	3.56364800	0.15229600
H	-3.35325700	1.67119200	-0.74259700
C	-1.85717300	4.35179800	0.72249900
H	0.22934900	4.47576700	1.27406000
H	-3.88094200	3.93820700	0.08455200

H	-2.09779100	5.34816000	1.10049900
C	-2.34936300	-1.18278800	0.17204000
C	-2.94198200	-2.35913500	-0.31114900
C	-2.75634200	-0.67068500	1.41336500
C	-3.94161500	-3.00001700	0.42056400
H	-2.62814900	-2.79067000	-1.26814700
C	-3.74464200	-1.32015100	2.14979700
H	-2.29313500	0.23752600	1.81174600
C	-4.34066600	-2.47996500	1.65114500
H	-4.40348300	-3.91127000	0.03354700
H	-4.05168300	-0.92068300	3.11923000
H	-5.11801200	-2.98491400	2.22938000
Sb	2.62033400	-0.33451900	0.34386600
F	4.39946300	-0.24075200	-0.17644100
F	2.83922200	-1.46172700	1.80775900
F	2.65945700	1.18181300	1.43926300
F	0.61233300	-0.38930600	0.69982600
F	2.17939500	-1.78809300	-0.76825200
F	1.96436900	0.76558100	-1.07126100

$BiPh_2$(cyclopentene)SbF_6

C	3.46167500	-1.12240100	-0.93579800
H	3.97827400	-0.38368900	-0.31554100
C	2.96624000	-0.88010400	-2.17079700
H	3.05706500	0.06963300	-2.70730100
C	2.43468800	-2.13476900	-2.79928200
H	3.15960300	-2.49450700	-3.55214300
H	1.48961400	-1.99423900	-3.34624400
Bi	0.63231000	0.08394600	-0.64780100
C	0.96252200	-1.05417000	1.24137000
C	0.06577900	-2.07725600	1.57444100
C	2.02835900	-0.76717400	2.10380000
C	0.24278900	-2.80730100	2.75092400
H	-0.79236200	-2.29323300	0.93423200
C	2.20460900	-1.50349900	3.27505900
H	2.73075100	0.03937000	1.87027000
C	1.31346100	-2.52695100	3.59710700
H	-0.46884100	-3.59456100	3.00871200
H	3.03901700	-1.27269700	3.94135100
H	1.44989300	-3.10165000	4.51631300
C	1.72002900	1.88210300	0.10394600
C	2.68555900	2.52103300	-0.68444700
C	1.39912300	2.42286500	1.35638400
C	3.33899300	3.66459700	-0.22228100
H	2.94233400	2.13596600	-1.67745700
C	2.04390600	3.57199300	1.81327900
H	0.63643700	1.94778800	1.98073100
C	3.01757200	4.18975600	1.02797500
H	4.09569300	4.14948500	-0.84393700
H	1.78289400	3.98768200	2.78915000
H	3.52363000	5.08804400	1.38936200
C	2.31896600	-3.08839000	-1.60462200
H	2.45707200	-4.14430500	-1.86976200
C	3.33570600	-2.57224200	-0.58209300
H	3.04500300	-2.75497500	0.46349900
H	1.30513300	-3.01208600	-1.17297900
H	4.32593900	-3.04942000	-0.70513500
Sb	-3.00788000	0.16111400	-0.09726200
F	-4.59240900	-0.59990300	-0.70614900
F	-3.76483500	1.71014900	0.60817500
F	-3.03092400	-0.69590700	1.56508000
F	-1.20414300	0.87101000	0.43478500
F	-2.60875900	1.01629700	-1.72432000
F	-1.91858200	-1.28114400	-0.73813300

$BiPh_2$(pentadiene)SbF_6

Bi	0.77108200	0.14805800	-0.56620000
Sb	-2.86056000	-0.44237800	-0.36311100
F	-2.72755300	-0.45362800	1.50147600
F	-4.03591500	1.00328000	-0.33406100
F	-1.38105500	-1.65537100	-0.40864900
F	-2.64886700	-0.30119900	-2.22220500
F	-4.14348500	-1.78505200	-0.44959700

F	-1.34035100	0.88376300	-0.32571200
C	3.58628900	-1.14408900	-0.92114100
H	3.67240800	-0.97900500	-2.00422000
C	3.78605500	-0.10665900	-0.09013200
H	4.03682300	0.88970700	-0.46372700
C	3.31886400	-2.55497300	-0.49862100
H	2.37518600	-2.91129800	-0.94847800
H	3.74783600	-0.23387700	0.99785800
H	3.18542300	-2.58702400	0.59660600
C	4.43149900	-3.47237600	-0.91116600
H	5.41460500	-3.24684400	-0.47958700
C	4.30358200	-4.50038900	-1.75021000
H	3.34160600	-4.75668900	-2.20455600
H	5.15299400	-5.13470100	-2.01117900
C	0.65322000	-0.39048200	1.57692300
C	0.64807200	-1.74452400	1.93758900
C	0.37640500	0.57873000	2.54801700
C	0.41270100	-2.12041900	3.25867200
H	0.78907000	-2.52418600	1.18298300
C	0.13197800	0.19948200	3.86750200
H	0.31960400	1.63669800	2.27591000
C	0.15787300	-1.14742900	4.22459700
H	0.40559200	-3.17868800	3.52981300
H	-0.09600300	0.96138900	4.61648100
H	-0.04126100	-1.44277700	5.25730500
C	1.29383300	2.29838300	-0.24094300
C	0.68322000	3.23904100	-1.08149400
C	2.19158700	2.74792000	0.73705900
C	0.96452600	4.59914400	-0.94799900
H	-0.03861400	2.92149000	-1.84101000
C	2.47916300	4.10692600	0.86564900
H	2.66563600	2.03858800	1.42358600
C	1.86634700	5.03333300	0.02212900
H	0.47326500	5.32197100	-1.60363600
H	3.17970400	4.44395000	1.63372700
H	2.08866700	6.09790400	0.12550900

$BiPh_2$(allylbenzene)SbF_6

Bi	0.07830100	0.54560100	-0.49500700
Sb	-2.84088700	-1.69753800	-0.42737300
F	-2.95820600	-1.46995500	1.42432200
F	-4.55165200	-1.00555200	-0.68554600
F	-0.97378300	-2.04731500	-0.19819200
F	-2.48150000	-1.64271700	-2.26872000
F	-3.31871800	-3.49413400	-0.41043300
F	-2.14366700	0.19524900	-0.47922300
C	3.18951900	0.72162600	-0.51234800
H	3.31621400	0.81374300	-1.60008700
C	2.77972400	1.79577500	0.18506000
H	2.57903300	2.75446700	-0.30059100
C	3.55899900	-0.60271900	0.07385800
H	2.91484000	-1.39190000	-0.35117500
H	2.67749400	1.76036400	1.27590500
H	3.36335900	-0.59083400	1.15969400
C	-0.03474000	0.23988500	1.69402000
C	0.56450500	-0.89472100	2.25669100
C	-0.86396900	1.04170600	2.48674800
C	0.37213100	-1.19813200	3.60313500
H	1.15826700	-1.57670400	1.64058900
C	-1.06264200	0.73073100	3.83155400
H	-1.38558000	1.89978800	2.05288900
C	-0.43932500	-0.38286500	4.39157900
H	0.83970700	-2.08734700	4.03228400
H	-1.72151600	1.35460900	4.43991600
H	-0.60190900	-0.62869000	5.44348200
C	-0.50641800	2.70371600	-0.45780300
C	-1.37326400	3.15175700	-1.46371600
C	-0.05010900	3.62021800	0.49942200
C	-1.77446900	4.48726200	-1.51241400
H	-1.76356500	2.45556700	-2.21326600
C	-0.44452000	4.95725000	0.44633600
H	0.60874200	3.29342000	1.31088600
C	-1.30629100	5.39167200	-0.56062600
H	-2.45869400	4.82035100	-2.29636500

H	-0.08270300	5.66110800	1.19997300
H	-1.61820900	6.43790400	-0.59981500
C	5.00143400	-0.95846000	-0.19785900
C	5.34206800	-2.13748900	-0.86862400
C	6.02793100	-0.10277100	0.22267400
C	6.67758900	-2.45976000	-1.10763900
H	4.55073400	-2.81412200	-1.20390800
C	7.36223800	-0.42287700	-0.01471500
H	5.77518800	0.82501600	0.74518100
C	7.69101000	-1.60343400	-0.68155400
H	6.92598100	-3.38613000	-1.63048500
H	8.15096700	0.25339400	0.32280600
H	8.73722300	-1.85489100	-0.86888300

$BiMe_2$(allylbenzene)SbF_6^{arene}

Bi	0.4874270	1.0187970	0.0762870
C	0.4786520	0.1557280	2.1189590
H	-0.5507970	-0.1933290	2.2883000
H	1.1487330	-0.7126400	2.1727810
H	0.7469920	0.8999220	2.8815250
C	-0.1137280	3.0330240	0.8358500
H	-0.1664610	3.7764990	0.0286430
H	-1.1092760	2.9282100	1.2912580
H	0.5873510	3.3869840	1.6053960
Sb	-2.4124440	-1.1944810	-0.4924450
F	-2.7452060	-1.4113820	1.3368510
F	-4.0771520	-0.4057630	-0.7638720
F	-0.5872390	-1.6386780	-0.1382160
F	-1.8325490	-0.6972250	-2.2066850
F	-2.8900220	-2.9262900	-0.9670900
F	-1.7096350	0.6272050	0.0216180
C	3.5973560	0.8437690	0.6010510
C	3.3199250	2.2098640	0.7002150
C	3.8359000	0.2429400	-0.6469590
H	3.6210260	0.2254190	1.5043710
C	3.2730160	3.0004820	-0.4546260
H	3.1486720	2.6602360	1.6821040
C	3.8014040	1.0507880	-1.7878340
C	3.5215790	2.4160820	-1.6951920
H	3.0472480	4.0666520	-0.3800890
H	3.9828260	0.6002620	-2.7675150
H	3.4921690	3.0258180	-2.6008910
C	4.0167960	-1.2501950	-0.7321650
H	4.4264290	-1.5134240	-1.7202980
H	4.7515270	-1.5846750	0.0172660
C	2.7090390	-1.9494840	-0.5060800
C	2.4190600	-2.7158210	0.5470430
H	1.9306950	-1.7732570	-1.2607280
H	1.4290650	-3.1607190	0.6649100
H	3.1598390	-2.9248760	1.3265320

$BiPh_2$(allylbenzene)SbF_6^{arene}

Bi	0.5858250	0.9279510	0.3792210
Sb	-2.2370360	-0.6020250	-1.3482820
F	-3.3197610	-1.6780620	-0.2816390
F	-3.5283800	0.7506570	-1.4104270
F	-0.7557490	-1.7415700	-1.0524060
F	-0.9823050	0.6151680	-2.1066510
F	-2.6937610	-1.3702140	-2.9781940
F	-1.5586560	0.2686990	0.3396890
C	3.8579660	0.6014440	0.6709080
C	3.5825340	1.9662040	0.8030030
C	3.8527970	-0.0169790	-0.5867800
H	4.0611510	0.0019040	1.5634670
C	3.3074410	2.7374630	-0.3319630
H	3.5883450	2.4265390	1.7952670
C	3.5932830	0.7723400	-1.7148780
C	3.3236410	2.1348270	-1.5920090
H	3.0827250	3.8019120	-0.2341100
H	3.5778660	0.3043380	-2.7033530
H	3.1106840	2.7297580	-2.4827120
C	3.9614900	-1.5149510	-0.6969130
H	4.4271130	-1.7837990	-1.6592290

H	4.6101850	-1.9137020	0.0978310
C	2.5935000	-2.1261110	-0.6023010
C	2.1636620	-2.9167750	0.3833860
H	1.8890980	-1.8525570	-1.3993340
H	1.1390160	-3.2954780	0.3943250
H	2.8193850	-3.2208720	1.2065460
C	0.6049210	0.7159120	2.5985390
C	-0.1669550	1.5157320	3.4523110
C	1.3986900	-0.3004600	3.1514880
C	-0.1312130	1.3121100	4.8316400
H	-0.8079700	2.3018210	3.0412390
C	1.4389570	-0.5001730	4.5310690
H	1.9872110	-0.9563710	2.4991100
C	0.6749090	0.3095110	5.3712950
H	-0.7393400	1.9379890	5.4893300
H	2.0612730	-1.2941390	4.9511550
H	0.7018290	0.1532150	6.4523450
C	-0.2362580	2.9975730	0.4480020
C	-1.3812170	3.3010710	-0.2993530
C	0.3842800	4.0062230	1.1971920
C	-1.8968340	4.5979750	-0.2903860
H	-1.8908190	2.5255110	-0.8749920
C	-0.1283860	5.3035330	1.1954770
H	1.2677450	3.7835840	1.8069730
C	-1.2696340	5.5993410	0.4493890
H	-2.7973440	4.8221070	-0.8669930
H	0.3596080	6.0833920	1.7855970
H	-1.6745950	6.6141000	0.4503590

[TMPH$_2^+$][OTf$^-$]

S	2.02588700	0.64569900	-0.68729800
F	1.73029100	-0.64873400	1.60743300
F	2.68775500	-1.80764900	0.05658900
F	3.78494400	-0.24490600	1.07074400
O	3.10724600	0.69292600	-1.66010100
O	1.74223800	1.84977900	0.10889500
O	0.77686600	-0.03041200	-1.19133000
N	-1.81940000	0.20824500	-0.84196000
C	-2.04743200	1.30906900	0.18934500
C	-2.12392600	-1.24847500	-0.51192200
C	-1.38639400	-1.55721600	0.79156700
C	2.60002600	-0.58432700	0.58053700
C	-1.29825300	0.86801800	1.44693600
C	-3.53160600	1.54335000	0.43182300
C	-1.55151100	-2.05911800	-1.66680800
C	-1.42107600	2.55837100	-0.41223700
C	-3.62313700	-1.49220500	-0.41050000
C	-1.64642400	-0.54289400	1.89447300
H	-1.66324600	-3.12957700	-1.45125200
H	-0.35169400	2.41614500	-0.61795700
H	-1.50855400	1.60013400	2.24103300
H	-2.29195500	0.46132000	-1.71428200
H	-2.08765800	-1.85791900	-2.60693900
H	-0.30838100	-1.59075200	0.57356800
H	-1.50221900	3.38690100	0.30286400
H	-0.48474500	-1.84693100	-1.81498600
H	-2.69522200	-0.59939000	2.23554100
H	-0.21827400	0.94308900	1.24368500
H	-4.00709600	0.78139800	1.05875600
H	-0.76766800	0.22871400	-1.04342200
H	-4.06878200	-1.13354900	0.52344200
H	-4.09833200	1.61680500	-0.50849900
H	-4.17365300	-1.04013500	-1.24898200
H	-1.67164200	-2.57188900	1.10887200
H	-1.03962100	-0.80363900	2.77331200
H	-3.65242400	2.50270600	0.95174700
H	-1.94034100	2.86735900	-1.33260600
H	-3.80619500	-2.57387400	-0.45529200

TS-2' (1a-TMS)

Bi	2.57134200	0.54281500	0.37274500
C	1.28410900	-0.98051400	1.41138900
C	0.53142400	-0.53345000	2.50804900

H	0.60072800	0.50787100	2.84167900
C	-0.33104100	-1.39700200	3.18326000
H	-0.90638900	-1.03706000	4.03997500
C	-0.44975300	-2.72438400	2.76890100
H	-1.12340700	-3.40370400	3.29626700
C	1.17909900	-2.32285200	1.02487600
C	0.31179200	-3.18813600	1.69519300
H	0.24013400	-4.23335500	1.38448100
C	5.40141500	-0.72516500	0.13958000
C	6.43486100	-1.59159100	-0.22240600
H	7.44001800	-1.43206100	0.17403100
C	6.18461300	-2.65063900	-1.09146900
H	6.99260400	-3.32626200	-1.37945600
C	4.89926400	-2.84253000	-1.59843000
H	4.69909400	-3.66708500	-2.28654600
C	3.86622800	-1.98130000	-1.23016000
H	2.87102000	-2.15229100	-1.65282300
C	4.10308100	-0.91350300	-0.35307900
H	5.62914400	0.10555800	0.81650900
H	1.78323100	-2.71272800	0.20042800
C	-3.30043000	-1.46944000	0.00698400
C	-2.83218700	-0.16306000	-0.23366900
C	-3.75116300	0.81417100	-0.65391600
C	-5.09497300	0.50953400	-0.80268000
C	-5.54435400	-0.79058800	-0.53239800
C	-4.64417500	-1.78359600	-0.12892100
C	-1.41127000	0.07494000	-0.04278400
H	-3.41026800	1.82168900	-0.89669200
H	-4.99992300	-2.79418000	0.07535000
C	1.53919800	0.90151600	-1.84953400
C	0.62288600	-0.17016000	-2.10275500
H	1.06262400	-1.16192000	-2.28131700
C	-0.75169600	-0.06671000	-2.13337000
H	-1.20708500	0.91608400	-2.28156600
H	1.05046000	1.87875500	-1.70978000
H	-5.80135100	1.27078000	-1.13662600
H	-2.59295800	-2.24185500	0.32236800
Br	-7.36324600	-1.20213200	-0.72661700
H	-0.83444000	-0.81235800	0.24372300
O	-0.86301600	1.19442300	0.32245800
Si	-1.34126900	2.85098500	0.80659700
C	0.23448000	3.45800800	1.60475000
H	1.06876600	3.49980000	0.88923700
H	0.09670100	4.48033000	1.98745200
H	0.54622700	2.83941700	2.45772000
C	-1.69500500	3.85856300	-0.73607300
H	-0.89772600	3.74319600	-1.48464100
H	-2.65198800	3.65233200	-1.23179200
H	-1.71013500	4.92428900	-0.46149800
C	-2.74802500	2.67097900	2.02552500
H	-3.70894800	2.40153500	1.56907300
H	-2.51000800	1.90777900	2.78132900
H	-2.89678100	3.61957000	2.56241400
C	2.79809000	0.98521300	-2.61556400
C	3.46333700	2.12438800	-2.84890500
H	3.09898800	3.09130900	-2.48782600
H	4.38909100	2.13234200	-3.42537200
H	3.20161100	0.04047100	-2.99757100
H	-1.34375200	-0.92262100	-2.46113300

TS-2 (1a-TMS)

Bi	-2.95565700	-1.59162600	-0.46035400
C	-1.45082600	-1.57109200	1.19603800
C	-0.73245800	-2.75553700	1.42526000
H	-0.90795600	-3.64606000	0.81105700
C	0.22137700	-2.81990700	2.44159700
H	0.77677800	-3.74548400	2.61019800
C	0.45405400	-1.70673000	3.24985600
H	1.19432700	-1.75901800	4.05127600
C	-1.21642300	-0.46271800	2.01899200
C	-0.27190300	-0.53308000	3.04265200
H	-0.10363400	0.33399400	3.68794300
C	-3.93958900	1.36708400	-1.17827500
C	-4.52699300	2.61333500	-0.95627800

H	-4.66574600	3.30417300	-1.79111100
C	-4.94948800	2.96887600	0.32440600
H	-5.41549600	3.94132700	0.49645400
C	-4.79114400	2.07247200	1.38008600
H	-5.13603300	2.33800900	2.38205500
C	-4.19652200	0.82915000	1.16006700
H	-4.09013700	0.14126800	2.00527000
C	-3.74842800	0.46851100	-0.11949500
H	-3.64561600	1.10547800	-2.20047400
H	-1.75476100	0.47272000	1.84833600
C	3.31473500	1.14302900	-0.33830900
C	2.37188000	0.10472900	-0.27383700
C	2.83554500	-1.21928600	-0.17025500
C	4.19109900	-1.50105700	-0.09912100
C	5.11289600	-0.44701500	-0.14296400
C	4.67577800	0.87591900	-0.26338800
C	0.92506000	0.32556500	-0.34864700
H	2.11108700	-2.03863700	-0.12753400
H	5.40240600	1.68851800	-0.30652200
C	-1.61343800	-0.97504300	-2.39620000
C	-0.82576500	0.19400500	-2.31519200
H	-1.36221300	1.15112200	-2.23905400
C	0.58119000	0.28074800	-2.29188800
H	1.11722900	-0.64540200	-2.53793300
H	-1.08842300	-1.89777500	-2.67866000
H	-2.52500300	-0.82649900	-2.99471500
C	1.19611800	1.52002900	-2.78288500
H	0.58736600	2.42758900	-2.68909300
C	2.42325200	1.59149600	-3.31025100
H	2.83975400	2.53637300	-3.66413300
H	3.05152400	0.70271800	-3.42433700
H	4.54100800	-2.52971900	-0.00390300
H	2.98325700	2.17567900	-0.45306700
Br	6.95220400	-0.81509300	-0.04328500
H	0.35338400	-0.58773600	-0.12496200
O	0.34128200	1.42610800	0.07877500
C	-0.98995500	3.20311600	1.82019800
H	-1.79585000	3.11042000	1.07705300
H	-1.21999500	2.52278800	2.65194500
H	-1.03195400	4.22633300	2.22201500
C	2.00797100	2.48719100	2.31284600
H	1.98570700	1.43236700	2.62398900
H	3.02618400	2.70674100	1.96656900
H	1.83440500	3.09287000	3.21529400
C	1.12386900	4.23398200	-0.16256900
H	2.03175900	4.04065300	-0.75103300
H	0.30298000	4.41834000	-0.87052500
H	1.29116000	5.17472700	0.38298700
Si	0.67737600	2.86641600	1.04172000

TS-2'out (1a-TMS)

Bi	-3.97167800	-0.03886200	-1.02986100
C	-5.12363800	-0.59981500	0.79850800
C	-5.69609500	0.38337500	1.61611500
H	-5.53807400	1.44748900	1.41040300
C	-6.48041400	0.02299200	2.71252000
H	-6.91863100	0.79835100	3.34531600
C	-6.70821000	-1.32307600	2.99611300
H	-7.32500300	-1.60462600	3.85222000
C	-5.36183200	-1.95057200	1.08883200
C	-6.15023100	-2.30884500	2.18309100
H	-6.32847900	-3.36464700	2.39999500
C	-2.01138800	-1.98555900	0.60746100
C	-0.86976300	-2.75990200	0.81962700
H	-0.64973300	-3.13914000	1.82080800
C	-0.01682500	-3.05818500	-0.24509200
H	0.87131600	-3.67351200	-0.07970700
C	-0.30919300	-2.58357700	-1.52316300
H	0.35000200	-2.82160400	-2.36181200
C	-1.44930700	-1.80496300	-1.73329600
H	-1.65505000	-1.43664100	-2.74443600
C	-2.31173200	-1.49769400	-0.67194600
H	-2.66753100	-1.75858200	1.45477200
H	-4.92714600	-2.74052900	0.46704300

C	-2.71048900	1.57859100	0.21167600
C	-1.49336000	1.82541100	-0.49924600
H	-1.50144100	2.62414200	-1.25551000
C	-0.34182100	1.07370300	-0.39819300
H	-0.31474400	0.24476700	0.32174800
H	-3.38533100	2.43978600	0.30039200
H	-2.60154800	1.03227700	1.15932700
C	0.78098900	1.26453800	-1.22544300
H	0.75509900	2.11623100	-1.91820700
C	1.92228500	0.48299400	-1.19240900
H	1.87269400	-0.48922500	-0.69436300
H	2.66466800	0.57793500	-1.98690500
C	3.18540300	1.37613900	0.34241800
C	4.25837900	0.39729200	0.28688400
C	5.46167000	0.67958300	-0.38197400
C	4.10253400	-0.84608400	0.92574200
C	6.48626300	-0.25459600	-0.40883000
C	5.12217400	-1.78423500	0.90684800
H	3.16891500	-1.07443200	1.44946700
C	6.31533000	-1.48707800	0.23457100
H	7.42282300	-0.03428800	-0.92291400
H	5.58584900	1.64679200	-0.87257500
H	5.00450800	-2.74363500	1.41213600
H	2.40040600	1.20332000	1.09824600
O	3.44343300	2.59903500	-0.00834700
Br	7.70015500	-2.75359900	0.20085400
Si	2.64202400	4.10931800	0.53736700
C	1.60507000	3.64670700	2.02778300
H	0.78400600	2.95894000	1.77592500
H	2.20392300	3.19277800	2.83087600
H	1.14063500	4.54928800	2.45236400
C	1.62462900	4.72811300	-0.90847200
H	1.42756100	5.80405700	-0.79181500
H	2.15536900	4.60342700	-1.86347300
H	0.64897300	4.22974000	-0.98509800
C	4.11493400	5.18869200	0.91783900
H	4.74468600	5.32504500	0.02718700
H	3.80387600	6.18746300	1.25548200
H	4.74330400	4.75408500	1.70756400

TS-2out (1a-TMS)

Bi	-3.08195000	-0.04746300	-0.96193900
C	-4.04531300	-0.74733100	0.91945900
C	-4.63928000	0.17989800	1.78742200
H	-4.58164000	1.25566900	1.58916700
C	-5.31998300	-0.25443500	2.92416500
H	-5.77390000	0.47571000	3.59784000
C	-5.42759100	-1.61868600	3.19445500
H	-5.96500500	-1.95833100	4.08223700
C	-4.16400400	-2.11738500	1.19445100
C	-4.85503100	-2.54823000	2.32769100
H	-4.94525300	-3.61738600	2.53222200
C	-0.98708400	-2.17219900	0.18992600
C	0.10516100	-3.03764800	0.14531500
H	0.48581900	-3.47981000	1.06959600
C	0.70986000	-3.34135400	-1.07606500
H	1.56823700	-4.01664800	-1.10754800
C	0.21578700	-2.78388800	-2.25413200
H	0.68421800	-3.02125900	-3.21212200
C	-0.87508800	-1.91340900	-2.21177500
H	-1.23547400	-1.47582500	-3.14882500
C	-1.48774500	-1.60070500	-0.99059600
H	-1.44914200	-1.94533600	1.15740500
H	-3.71923900	-2.86276600	0.52764500
C	4.03900400	0.72305000	-0.08614600
C	2.82259500	0.62723800	0.60269200
C	2.52292700	-0.55103300	1.30252800
C	3.41273700	-1.61491700	1.31655500
C	4.62030100	-1.50682100	0.61551600
C	4.94015200	-0.33616300	-0.07727000
C	1.83897300	1.72471000	0.55467100
H	1.57408500	-0.63639500	1.84300800
H	5.88909400	-0.25923100	-0.60992100
C	-1.56547300	1.35526200	0.40953700

C	-0.58029000	1.76883600	-0.49802500
H	-0.75138500	2.71243600	-1.03855800
C	0.62250800	1.07248600	-0.77649900
H	-2.26002600	2.10947000	0.79906100
H	-1.29800500	0.56725900	1.12697800
H	3.18167500	-2.52703000	1.86908500
H	4.27356600	1.64254700	-0.62471000
Br	5.82145400	-2.95612400	0.61172200
H	1.15121700	1.73868600	1.42161400
O	2.31710100	2.89321000	0.15622900
C	1.30664800	1.30434700	-2.05820600
H	1.17298600	2.29459500	-2.51065000
C	2.09677900	0.40181200	-2.64848100
H	2.25279300	-0.59004100	-2.21059800
H	2.60965900	0.61445000	-3.58808500
H	0.60556700	0.02651400	-0.44229700
Si	1.76292600	4.52278300	0.54292000
C	0.44475700	4.36698400	1.87133000
H	0.07443600	5.36475700	2.14977900
H	-0.43049500	3.78499700	1.54828000
H	0.83428000	3.90809400	2.79166200
C	1.11170400	5.22387000	-1.06889500
H	0.19156900	4.73802500	-1.42180200
H	0.88318900	6.29412400	-0.95967200
H	1.86324600	5.13549700	-1.86639300
C	3.30806300	5.38686900	1.13171200
H	3.71397100	4.91600600	2.03751700
H	4.09307900	5.36054400	0.36338900
H	3.11365900	6.44291700	1.36663600

TS-2' (1a-BF3)

Bi	2.91236000	-1.33736600	0.18885300
C	1.21611000	-1.59488100	-1.24315000
C	0.34976600	-2.67936800	-1.01566600
H	0.52687500	-3.37141200	-0.18174500
C	-0.76182900	-2.88497400	-1.83308200
H	-1.43673900	-3.72500700	-1.64074500
C	-1.01524100	-2.00831300	-2.89100900
H	-1.89455100	-2.15678600	-3.52259100
C	0.97022500	-0.74209300	-2.32810400
C	-0.14391500	-0.94644400	-3.14344100
H	-0.34011500	-0.26134100	-3.97269300
C	4.98378100	0.89112600	-0.54842100
C	5.47885000	2.15221300	-0.88535900
H	6.55861100	2.31581800	-0.94084000
C	4.59518100	3.20169800	-1.13796700
H	4.98298700	4.19258100	-1.38850900
C	3.21788300	2.99036300	-1.06044000
H	2.51264800	3.80621800	-1.23289800
C	2.71880000	1.72614700	-0.74117300
H	1.63270600	1.59862400	-0.68821100
C	3.60030400	0.66692700	-0.48168300
H	5.69371500	0.08383700	-0.33489400
H	1.63160000	0.10645200	-2.53096800
C	-3.70684900	1.30772800	-0.23696500
C	-2.81127500	0.26553800	0.03679000
C	-3.32270000	-0.99498500	0.38612700
C	-4.68918500	-1.21467300	0.49097700
C	-5.56473900	-0.15804900	0.22830200
C	-5.07901600	1.09684500	-0.14488800
C	-1.34479000	0.42106400	-0.11931100
H	-2.63225900	-1.82373600	0.57216400
H	-5.77271400	1.90888900	-0.36641400
C	1.74828000	-0.43379300	2.16041000
C	0.68118500	0.44772300	1.80688500
H	0.96065600	1.49190700	1.58357200
C	-0.67592400	0.15209600	1.73336400
H	-1.00798100	-0.85097100	2.04037600
H	1.42581600	-1.38398400	2.61725200
H	-5.07811900	-2.19706100	0.76616800
H	-3.32899500	2.28225800	-0.55053800
Br	-7.42927300	-0.44202600	0.36503200
H	-0.87941500	-0.49950800	-0.48843200
O	-0.80284600	1.49516900	-0.59217200

B	-0.97918800	2.94001500	-0.07452400
F	0.31156300	3.40140700	0.10232400
F	-1.65564700	2.87661400	1.13984400
F	-1.67374000	3.65024800	-1.02273800
C	2.94864800	0.16793000	2.77745900
C	3.79073100	-0.46292000	3.60533500
H	3.62917900	-1.50084600	3.91304700
H	4.66324900	0.04354900	4.02000000
H	3.15036700	1.20939900	2.49535400
H	-1.32544100	0.98613500	2.02584100

TS-2 (1a-BF3)

0 1

Bi	-2.51822900	-0.71083700	0.56111000
C	-1.54309600	1.14574200	1.37879900
C	-0.62576200	0.94552200	2.42431000
H	-0.34999800	-0.06699000	2.73439600
C	-0.02628200	2.03184000	3.05902000
H	0.69185900	1.85906300	3.86440300
C	-0.34145800	3.33321700	2.66547800
H	0.13135600	4.18490600	3.16061300
C	-1.87821000	2.45565100	1.01583900
C	-1.27340300	3.54342100	1.65038300
H	-1.53857900	4.56092000	1.35158600
C	-5.42711300	0.07995300	-0.44462100
C	-6.43898700	0.68382300	-1.19535000
H	-7.48274300	0.40648900	-1.02576100
C	-6.11744800	1.63364600	-2.16291700
H	-6.90597500	2.10468500	-2.75460900
C	-4.78223200	1.97861200	-2.37443100
H	-4.52118600	2.72336400	-3.13114400
C	-3.77735500	1.37353900	-1.61924900
H	-2.73857600	1.67176400	-1.79989500
C	-4.07900000	0.41514900	-0.63854700
H	-5.71054100	-0.66949700	0.30391700
H	-2.62282700	2.64521400	0.23802600
C	3.05131100	1.28841800	-0.12757400
C	2.50969300	-0.00442600	-0.15535300
C	3.38270300	-1.10075500	-0.21187600
C	4.75847800	-0.90439500	-0.21513100
C	5.27571500	0.39284700	-0.17166000
C	4.42579800	1.49760800	-0.12614700
C	1.03298800	-0.10303200	-0.18191400
H	2.98833200	-2.11540100	-0.26236800
H	4.83453800	2.50860100	-0.09574800
C	-1.57042900	-1.42986100	-1.58819300
C	-0.71035400	-0.46327600	-2.12943200
H	-1.17187200	0.42942100	-2.57671700
C	0.69984100	-0.49685400	-2.09267400
H	1.13864100	-1.49816100	-1.99573700
H	-1.13037700	-2.40115100	-1.33123200
H	-2.56890800	-1.47417100	-2.04156300
C	1.42843400	0.46072000	-2.93021200
H	0.89552600	1.39171800	-3.16799500
C	2.66726200	0.28222500	-3.40298300
H	3.15939000	1.03681800	-4.01908500
H	3.23535800	-0.62837500	-3.18880700
H	5.43339700	-1.76070000	-0.25473300
H	2.37784200	2.15070400	-0.09928300
Br	7.14851400	0.65488500	-0.18559600
H	0.56621400	0.89621300	-0.13681700
O	0.35030300	-0.97886100	0.50720200
B	0.68928400	-2.44199100	0.85719600
F	1.71421900	-2.45471900	1.76149500
F	1.01117800	-3.08757600	-0.33630200
F	-0.50220500	-2.91016400	1.38095500

1a

C	-0.10462000	-1.13607700	-0.00000100
C	-1.48868400	-1.04127300	0.00000000
C	-2.11620000	0.21293600	0.00000200
C	-1.33435300	1.37392600	-0.00000200
C	0.05423200	1.29562000	-0.00000900

C	0.65988700	0.03689400	-0.00000400
H	0.38985300	-2.10876200	0.00000200
H	-2.11599800	-1.93607700	0.00000300
H	-1.82447400	2.35239900	-0.00000300
H	0.66683300	2.19829000	-0.00001700
Br	2.54461400	-0.08586800	0.00000200
C	-3.58792200	0.30564800	0.00001100
H	-3.98178500	1.35868100	0.00004900
O	-4.33624300	-0.64314600	-0.00000900

BF3·Et2O

B	0.30821200	-1.09554800	-0.01692200
F	0.96511000	-1.23964000	1.15133900
F	1.07716600	-1.12138400	-1.13508400
F	-0.87363100	-1.74793500	-0.11584700
O	-0.20002600	0.56537900	0.03057500
C	-1.51673900	0.81997800	-0.52765800
H	-1.50056800	1.84757900	-0.91908100
H	-1.66205200	0.14074000	-1.38071900
C	0.80297600	1.53339300	-0.36275500
H	0.97846400	1.43161000	-1.44688200
H	0.35556100	2.52293500	-0.18118300
C	-2.56665000	0.64863400	0.52940200
H	-3.55935400	0.84666800	0.10479700
H	-2.56390700	-0.37353400	0.92329100
H	-2.40734800	1.34519500	1.36287700
C	2.06583200	1.36169100	0.42668000
H	2.74698700	2.18791200	0.18587000
H	1.86681700	1.38241500	1.50521400
H	2.57423200	0.42164500	0.18814100

Et2O

O	0.00000000	-0.26294500	0.00008900
C	-1.17076800	0.51002700	-0.00000900
H	-1.18922800	1.18109700	0.88559800
H	-1.18912100	1.18112100	-0.88561400
C	1.17075200	0.50999700	-0.00000500
H	1.18911300	1.18101200	-0.88563400
H	1.18926400	1.18115100	0.88552800
C	-2.36371000	-0.40506800	-0.00003500
H	-3.30060300	0.16660300	-0.00000100
H	-2.35780700	-1.05339400	-0.88641000
H	-2.35776600	-1.05335200	0.88636100
C	2.36370700	-0.40506800	-0.00001500
H	3.30057500	0.16669100	-0.00031200
H	2.35799600	-1.05319200	0.88643700
H	2.35768900	-1.05350400	-0.88628500

References

1. Wang, R.; Wang, Y.; Ding, R.; Staub, P. B.; Zhao, C. Z.; Liu, P.; Wang, Y. M., Designed Iron Catalysts for Allylic C-H Functionalization of Propylene and Simple Olefins. *Angew. Chem. Int. Ed.* **2023**, *62* (10), e202216309.
2. Li, Y.; Zhou, K.; Wen, Z.; Cao, S.; Shen, X.; Lei, M.; Gong, L., Copper(II)-Catalyzed Asymmetric Photoredox Reactions: Enantioselective Alkylation of Imines Driven by Visible Light. *J. Am. Chem. Soc.* **2018**, *140* (46), 15850–15858.
3. Minakata, S.; Morino, Y.; Oderaotoshi, Y.; Komatsu, M., Practical and Convenient Synthesis of N-Heterocycles: Stereoselective Cyclization of *N*-Alkenylamides with t-BuOI under Neutral Conditions. *Org. Lett.* **2006**, *8* (15), 3335–3337.
4. Woof, C. R.; Durand, D. J.; Fey, N.; Richards, E.; Webster, R. L., Iron Catalyzed Double Bond Isomerization: Evidence for an Fe^{I}/Fe^{III} Catalytic Cycle. *Chem. Eur. J.* **2021,** *27* (19), 5972–5977.
5. Lichtenberg, C.; Pan, F.; Spaniol, T. P.; Englert, U.; Okuda, J., The bis(allyl)bismuth cation: a reagent for direct allyl transfer by Lewis acid activation and controlled radical polymerization. *Angew. Chem. Int. Ed.* **2012,** *51* (52), 13011–13015.
6. Bauld, N. L.; Aplin, J. T.; Yueh, W.; Endo, S., Cation radical cycloaddition polymerization: Diels–Alder copolymerization. *J. Phys. Org. Chem.* **1998,** *11* (11), 825–830.
7. Frisch, M. J.; Trucks, G. W.; Schlegel, H. B.; Scuseria, G. E.; Robb, M. A.; Cheeseman, J. R.; Scalmani, G.; Barone, V.; Petersson, G. A.; Nakatsuji, H.; Li, X.; Caricato, M.; Marenich, A. V.; Bloino, J.; Janesko, B. G.; Gomperts, R.; Mennucci, B.; Hratchian, H. P.; Ortiz, J. V.; Izmaylov, A. F.; Sonnenberg, J. L.; Williams-Young, D.; Ding, F.; Lipparini, F.; Egidi, F.; Goings, J.; Peng, B.; Petrone, A.; Henderson, T.; Ranasinghe, D.; Zakrzewski, V. G.; Gao, J.; Rega, N.; Zheng, G.; Liang, W.; Hada, M.; Ehara, M.; Toyota, K.; Fukuda, R.; Hasegawa, J.; Ishida, M.; Nakajima, T.; Honda, Y.; Kitao, O.; Nakai, H.; Vreven, T.; Throssell, K.; Montgomery, J. A., Jr.; Peralta, J. E.; Ogliaro, F.; Bearpark, M. J.; Heyd, J. J.; Brothers, E. N.; Kudin, K. N.; Staroverov, V. N.; Keith, T. A.; Kobayashi, R.; Normand, J.; Raghavachari, K.; Rendell, A. P.; Burant, J. C.; Iyengar, S. S.; Tomasi, J.; Cossi, M.; Millam, J. M.; Klene, M.; Adamo, C.; Cammi, R.; Ochterski, J. W.; Martin, R. L.; Morokuma, K.; Farkas, O.; Foresman, J. B.; Fox, D. J. Gaussian16, 2016.
8. Cramer, C. J.; Truhlar, D., Density functional theory for transition metals and transition metal chemistry. *Phys. Chem. Chem. Phys.* **2009,** *11* (46), 10757–10816.
9. Marenich, A. V.; Cramer, C. J.; Truhlar, D., Universal solvation model based on solute electron density and on a continuum model of the solvent defined by the bulk dielectric constant and atomic surface tensions. *J. Phys. Chem. B* **2009,** *113* (18), 6378–6396.
10. Weigend, F., Accurate Coulomb-fitting basis sets for H to Rn. *Phys. Chem. Chem. Phys.* **2006,** *8* (9), 1057–1065.
11. Weigend, F.; Ahlrichs, R., Balanced basis sets of split valence, triple zeta valence and quadruple zeta valence quality for H to Rn: Design and assessment of accuracy. *Phys. Chem. Chem. Phys.* **2005,** *7* (18), 3297–3305.
12. Zhao, Y.; Truhlar, D., Density functionals with broad applicability in chemistry. *Acc. Chem. Res.* **2008,** *41* (2), 157–167.

13. Hay, P. J.; Wadt, W., Ab initio effective core potentials for molecular calculations. Potentials for K to Au including the outermost core orbitals. *J. Chem. Phys.* **1985,** *82* (1), 299–310.
14. Glendening, E. D.; Landis, C. R.; Weinhold, F., NBO 6.0: Natural bond orbital analysis program. *J. Comput. Chem.* **2013,** *34* (16), 1429–1437.
15. Knizia, G., Intrinsic atomic orbitals: An unbiased bridge between quantum theory and chemical concepts. *J. Chem. Theory Comput.* **2013,** *9* (11), 4834–4843.
16. Lu, T.; Chen, F., Multiwfn: A multifunctional wavefunction analyzer. *J. Comput. Chem.* **2012,** *33* (5), 580–592.
17. Yang, X.; Reijerse, E. J.; Bhattacharyya, K.; Leutzsch, M.; Kochius, M.; Nöthling, N.; Busch, J.; Schnegg, A.; Auer, A. A.; Cornella, Radical activation of N–H and O–H bonds at bismuth (II). *J. Am. Chem. Soc.* **2022,** *144* (36), 16535–16544.
18. Ishida, S.; Hirakawa, F.; Furukawa, K.; Yoza, K.; Iwamoto, Persistent Antimony- and Bismuth-Centered Radicals in Solution. *Angew. Chem. Int. Ed.* **2014,** *53* (42), 11172–11176.
19. Inaba, R.; Oka, K.; Iwami, T.; Miyake, Y.; Tajima, K.; Imoto, H.; Naka, K., Systematic Study of Pnictogen-Fused Heterofluorenes. *Inorg. Chem.* **2022,** *61* (19), 7318–7326.
20. V. Dolomanov, L. J. Bourhis, R. J. Gildea, J. A. K. Howard, H. Puschmann, *J. Appl. Crystallogr.* **2009**, 42, 339.
21. G. M. Sheldrick, *Acta Crystallogr. A* **2008**, 64, 112.
22. A. Schier, J. M. Wallis, G. Müller, H. Schmidbaur, *Angew. Chem. Int. Ed.* **1986**, 25, 757; b) G. Becker, J. Egner, M. Meiser, O. Mundt, J. Weidlein, Z. *anorg. allg. Chem.* **1997**, 623, 941; c) W. Frank, V. Reiland, G. J. Reiß, *Angew. Chem. Int. Ed.* **1998**, 37, 2983; d) C. Silvestru, H. J. Breunig, H. Althaus, *Chem. Rev.* **1999**, 99, 3277; e) J. H. Thurston, K. H. Whitmire, *Inorg. Chem.* **2002**, 41, 4194; f) D. Mansfeld, M. Mehring, M. Schürmann, Z. *anorg. allg. Chem.* **2004**, 630, 1795; g) H. Schmidbaur, A. Schier, *Organometallics* **2008**, 27, 2361; h) A. A. Auer, D. Mansfeld, C. Nolde, W. Schneider, M. Schürmann, M. Mehring, *Organometallics* **2009**, 28, 5405; i) T. Dunaj, M. Egorycheva, A. Arebi, K. Dollberg, C. von Hänisch, Z. *anorg. allg. Chem.* **2023**, 649; j) A.-M. Preda, M. Krasowska, L. Wrobel, P. Kitschke, P. C. Andrews, J. G. MacLellan, L. Mertens, M. Korb, T. Rüffer, H. Lang et al., Beilstein *J. Org. Chem.* **2018**, 14, 2125.
23. H.-G. Stammler, B. Neumann, *CCDC 1029501: Experimental Crystal Structure Determination*, Cambridge Crystallographic Data Centre, **2014**.
24. J. Ramler, F. Fantuzzi, F. Geist, A. Hanft, H. Braunschweig, B. Engels, C. Lichtenberg, *Angew. Chem. Int. Ed.* **2021**, 60, 24388.
25. Jurrat, M.; Maggi, L.; Lewis, W.; Ball, L. T., Modular bismacycles for the selective C-H arylation of phenols and naphthols. *Nat. Chem.* **2020,** *12* (3), 260–269.
26. Shimada, S.; Yamazaki, O.; Tanaka, T.; Rao, M. L.; Suzuki, Y.; Tanaka, M., 5,6,7,12-tetrahydrodibenz[c,f][1,5]azabismocines: highly reactive and recoverable organobismuth reagents for cross-coupling reactions with aryl bromides. *Angew. Chem. Int. Ed.* **2003,** *42* (16), 1845–8.
27. Zhang, S.; Li, L.; Hu, Y.; Zha, Z.; Wang, Z.; Loh, T.-P., Bifunctional Amino Sulfonohydrazide Catalyzed Direct Asymmetric Mannich Reaction of Cyclic Ketimines with Ketones: Highly Diastereo- and Enantioselective Construction of Quaternary Carbon Stereocenters. *Org. Lett.* **2015,** *17* (4), 1050–1053.
28. Zhang, H.; Jiang, C.; Tan, J.-P.; Hu, H.-L.; Chen, Y.; Ren, X.; Zhang, H.-S.; Wang, T., Highly Enantioselective Construction of Fully Substituted Stereocenters

Enabled by In Situ Phosphonium-Containing Organocatalysis. *ACS Catal.* **2020,** *10* (10), 5698–5706.
29. Wang, H.; Jiang, T.; Xu, M.-H., Simple Branched Sulfur–Olefins as Chiral Ligands for Rh-Catalyzed Asymmetric Arylation of Cyclic Ketimines: Highly Enantioselective Construction of Tetrasubstituted Carbon Stereocenters. *J. Am. Chem. Soc.* **2013,** *135* (3), 971–974.
30. Hu, S.; Neckers, D. C., Photochemical Reactions of Alkyl Phenylglyoxylates1. *J. Org. Chem.* **1996,** *61* (18), 6407–6415.
31. Roy, S.; Kumar, G.; Chatterjee, I., Photoinduced Diverse Reactivity of Diazo Compounds with Nitrosoarenes. *Org. Lett.* **2021,** *23* (17), 6709–6713.
32. He, Y.; Sun, B.; Lu, X.; Zhou, Y.; Zhang, F.-L., Iridium-Catalyzed Direct Ortho-C–H Amidation of α-Ketoesters with Sulfonyl Azides Using a Transient Directing Group Strategy. *J. Org. Chem.* **2023,** *88* (7), 4345–4351.
33. Qi, X.; Chen, P.; Liu, G., Catalytic Oxidative Trifluoromethoxylation of Allylic C−H Bonds Using a Palladium Catalyst. *Angew. Chem. Int. Ed.* **2017,** *56* (32), 9517–9521.
34. Liu, W.; Ali, S. Z.; Ammann, S. E.; White, M. C., Asymmetric Allylic C–H Alkylation via Palladium(II)/cis-ArSOX Catalysis. *J. Am. Chem. Soc.* **2018,** *140* (34), 10658–10662.
35. Zhang, Y.; Wang, C.; Rothberg, L.; Ng, M.-K., Surface-initiated growth of conjugated polymers for functionalization of electronically active nanoporous networks: synthesis, structure and optical properties. *J. Mater. Chem.* **2006,** *16* (37), 3721–3725.
36. Gomes, P.; Gosmini, C.; Périchon, J., New Chemical Cross-Coupling between Aryl Halides and Allylic Acetates Using a Cobalt Catalyst. *Org. Lett.* **2003,** *5* (7), 1043–1045.
37. Querolle, O.; Dubois, J.; Thoret, S.; Roussi, F.; Guéritte, F.; Guénard, D., Synthesis of C2−C3‘N-Linked Macrocyclic Taxoids. Novel Docetaxel Analogues with High Tubulin Activity. *J. Med. Chem.* **2004,** *47* (24), 5937–5944.
38. Lynd, R. A.; Zweifel, G., Allylation of Vinylalanes. A Convenient Synthesis of Isomerically Pure trans-1,4-Dienes. *Synthesis.* **1974,** *1974* (09), 658–659.
39. Nguyen, N. N. M.; Leclère, M.; Stogaitis, N.; Fallis, A. G., Triquinanes: A “One-Pot” IMDA-Tandem Metathesis Cascade Strategy: Ring-Closing Metathesis (RCM) Dominates Norbornene ROM! *Org. Lett.* **2010,** *12* (8), 1684–1687.
